# Prospective inter- and intra-tracer repeatability analysis of radiomics features in [^68^Ga]Ga-PSMA-11 and [^18^F]F-PSMA-1007 PET scans in metastatic prostate cancer

**DOI:** 10.1259/bjr.20221178

**Published:** 2023-10-24

**Authors:** Jake Kendrick, Roslyn J. Francis, Ghulam Mubashar Hassan, Pejman Rowshanfarzad, Jeremy S.L. Ong, Robert Jeraj, Nathaniel Barry, Tammy Hagan, Martin A. Ebert

**Affiliations:** 1 School of Physics, Mathematics and Computing, University of Western Australia, Perth, Australia; 2 Medical School, University of Western Australia, Crawley, Australia; 3 Department of Nuclear Medicine, Sir Charles Gairdner Hospital, Perth, Australia; 4 Department of Nuclear Medicine, Fiona Stanley Hospital, Murdoch, Australia; 5 Department of Medical Physics and Human Oncology, University of Wisconsin, Madison, United States; 6 Department of Physics, University of Ljubljana, Ljubljana, Slovenia; 7 5D Clinics, Claremont, Australia; 8 Department of Radiation Oncology, Sir Charles Gairdner Hospital, Perth, Australia

## Abstract

**Objective:**

This study aimed to quantify both the intra- and intertracer repeatability of lesion-level radiomics features in [^68^Ga]Ga-prostate-specific membrane antigen (PSMA)-11 and [^18^F]F-PSMA-1007 positron emission tomography (PET) scans.

**Methods:**

Eighteen patients with metastatic prostate cancer (mPCa) were prospectively recruited for the study and randomised to one of three test–retest groups: (i) intratracer [^68^Ga]Ga-PSMA-11 PET, (ii) intratracer [^18^F]F-PSMA-1007 PET or (iii) intertracer between [^68^Ga]Ga-PSMA-11 and [^18^F]F-PSMA-1007 PET. Four conventional PET metrics (standardised uptake value (SUV)_max_, SUV_mean_, SUV_total_ and volume) and 107 radiomics features were extracted from 75 lesions and assessed using the repeatability coefficient (RC) and the ICC. Radiomic feature repeatability was also quantified after the application of 16 filters to the PET image.

**Results:**

Test–retest scans were taken a median of 5 days apart (range: 2–7 days). SUV_mean_ demonstrated the lowest RC limits of the conventional features, with RCs of 7.9%, 14.2% and 24.7% for the [^68^Ga]Ga-PSMA-11 PET, [^18^F]F-PSMA-1007 PET, and intertracer groups, respectively. 69%, 66% and 9% of all radiomics features had good or excellent ICC values (ICC ≥ 0.75) for the same groups. Feature repeatability therefore diminished considerably for the intertracer group relative to intratracer groups.

**Conclusion:**

In this study, robust biomarkers for each tracer group that can be used in subsequent clinical studies were identified. Overall, the repeatability of conventional and radiomic features were found to be substantially lower for the intertracer group relative to both intratracer groups, suggesting that assessing patient response quantitatively should be done using the same radiotracer where possible.

**Advances in knowledge:**

Intertracer biomarker repeatability limits are significantly larger than intratracer limits.

## Introduction

Prostate cancer (PCa) is a leading cause of cancer-related mortality worldwide with a yearly estimated death toll exceeding 370,000.^1^ Advanced PCa in particular carries a poor prognosis, with 5 year survival rates for patients with metastatic disease spread dropping to 35% or below.^2,3^ Prostate-specific membrane antigen (PSMA) is a Type II transmembrane glycoprotein whose expression is significantly upregulated in malignant prostate cells, making it a promising molecular target enabling both diagnostic imaging and PCa-targeted therapeutics.^4–6^ Recent years have seen the development of numerous radioligands that target the PSMA receptor including ^68^Ga-labelled compounds such as [^68^Ga]Ga-PSMA-11 and ^18^F-labelled compounds such as [^18^F]F-PSMA-1007,^7,8^ facilitating positron emission tomography (PET) imaging.^9,10^ PSMA-PET imaging has demonstrated improved diagnostic performance relative to conventional imaging modalities, particularly in the setting of biochemical relapse.^11–13^ Fluorinated PSMA compounds possess several advantages over gallium-labelled tracers that have driven their development in recent years, including a lower positron energy leading to higher theoretical spatial resolution, longer half-life facilitating centralised radioligand production that can be distributed to the required imaging locations, and reduced urinary excretion that does not obscure prostate assessment.^8,14^

Quantitative metrics derived from PSMA-PET imaging have a strong potential to be used in the treatment response setting—longitudinal changes in quantitative imaging biomarkers may evaluate whether treatment is effective or not. Several PSMA-specific progression criteria have been developed to define patient disease progression more concretely, including the PSMA-PET progression (PPP) criteria and the response evaluation criteria in PSMA-imaging (RECIP) 1.0.^15,16^ However, categorising response at the patient level might obscure potentially clinically meaningful changes at the lesion level in diseases such as metastatic PCa (mPCa).

There are many potential candidate imaging biomarkers that can be used to assess response at the lesion level in mPCa. The above response assessment frameworks often use quantitative imaging metrics such as standardised uptake value (SUV) and tumour volume measurements, in combination with other clinical information, for assessment of disease progression. But recent years have seen the development of a new field of quantitative imaging known as ‘radiomics’, characterised by the extraction of large volumes of imaging features from medical images. Radiomics features can be used to non-invasively characterise tumour heterogeneity at the radiographic level, and combinations of these features can be used to build useful predictive models.^17^ This approach to disease characterisation has demonstrated significant potential in the management of mPCa patients, with applications ranging from the detection of metastatic disease to the identification of new imaging biomarkers with potential use in response assessment settings.^18–21^ However, despite their potential, investigations of the repeatability of these radiomics features, which is necessary for determining minimum response assessment thresholds, are limited.

For a reliable assessment of treatment response, it is critical that the intrinsic measurement variability of quantitative biomarkers is adequately characterised in a test–retest setting. Only changes outside of the measured repeatability limits can be attributed to true biological change of the disease. This prospective study aims to characterise both the intertracer (defined as having a test–retest scan with different radiotracers) and intratracer (test–retest scans with the same radiotracer) lesion-level repeatability of both conventional PET quantitative metrics (SUV_max_, SUV_mean_, SUV_total_, and volume) and a more extensive suite of radiomics features in [^68^Ga]Ga-PSMA-11 and [^18^F]F-PSMA-1007 PET scans. To our knowledge, this would be the first estimation of radiomic feature repeatability in a test–retest setting for these tracers.

## Methods

### Study design

Eighteen patients with mPCa referred for PSMA PET/CT imaging due to rising PSA levels were prospectively recruited from the medical and radiation oncology departments at Sir Charles Gairdner Hospital, Perth, Western Australia between July 2020 and October 2021. To be included in the study, there must have been no change in prostate-specific treatment within 3 months of the date of PSMA scan referral. All patients provided written informed consent prior to participating in the study. Following recruitment, patients were randomised to one of four groups representing the four possible radiotracer test–retest combinations. Subject test scans were acquired, which were used for clinical indication for the PSMA-PET scan referral, and then subsequent retest scans were taken a maximum of 1 week after the initial test scan. Routine clinical data related to PCa treatment, including the PSA levels, PSA doubling time, previous and current treatment, were collected at the time of patient enrolment. This study was registered with the Australian New Zealand Clinical Trials Registry (ACTRN12620001110976) on 27 October 2020.

### Imaging protocol

All imaging examinations were performed on the same Siemens Biograph mCT 64 PET/CT scanner (CTI Inc, Knoxville TN). For [^68^Ga]Ga-PSMA-11 scans, 1.7 MBq/Kg was administered intravenously (median activity of 138 MBq, range: 106–210 MBq) and image acquisition began after a median of 79 min (range: 59–104 min) of uptake time post-injection. For the [^18^F]F-PSMA-1007 examinations, 4 MBq/Kg was administered intravenously (median activity of 381 MBq, range: 229–408 MBq) and image acquisition began after a median of 132 min (range: 104–166 min) of uptake time post-injection. Patients were asked to void immediately prior to the commencement of imaging.

PET emission data were acquired from the middle of the thigh to the skull vertex immediately following a low-dose CT acquisition for attenuation correction (120 kVp using CareDose dose modulation) to ensure identical field of view for both modalities. PET images were iteratively reconstructed (2 iterations, 21 subsets) using the point spread function with time of flight, dead time, scatter and random corrections applied, yielding a final PET axial matrix size of 200 × 200 with a voxel resolution of 4.07 × 4.07 × 2 mm^3^. A post-reconstruction gaussian filter of 6 mm was applied. CT images were reconstructed to an axial matrix size of 512 × 512, with a voxel resolution of 1.52 × 1.52 × 5 mm^3^.

### Lesion segmentation

**Figure 1.**
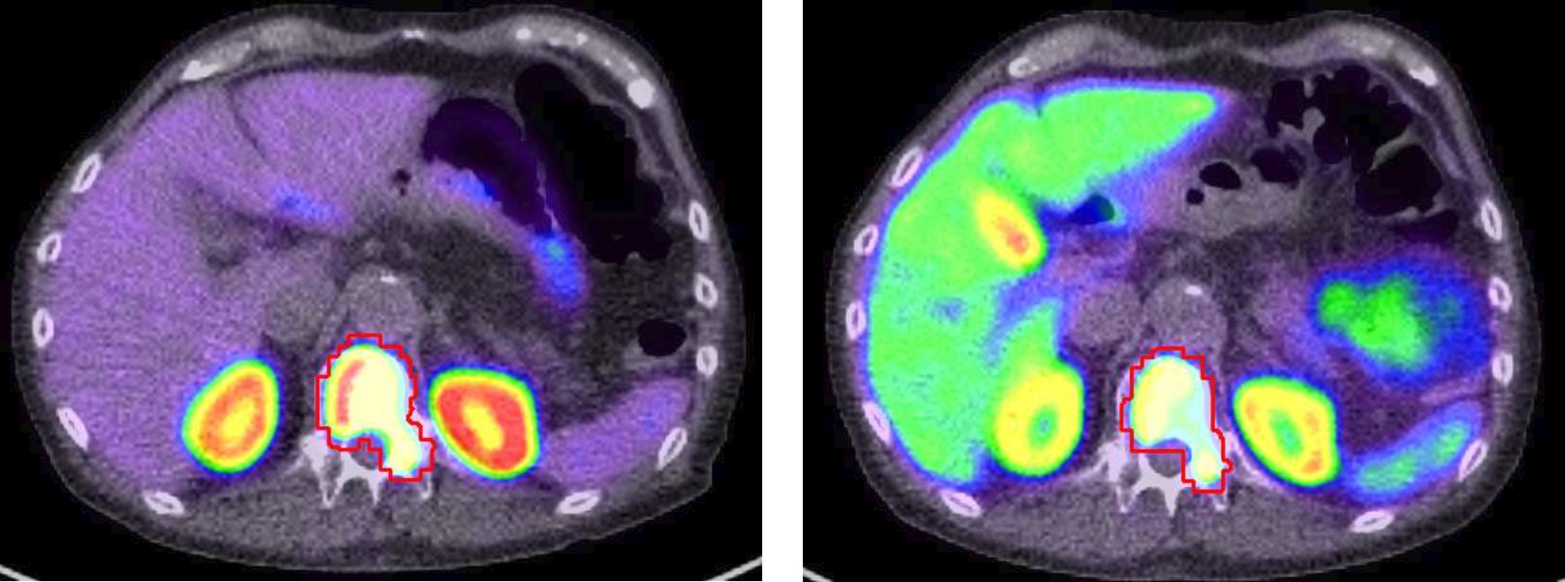
Exemplar PET/CT fusion axial slices taken from the test scan (left) and the retest scan (right) of a single patient using the MIM Encore software. Images show the same osseous lesion in the thoracic spine delineated clearly in red. Patient test scan was acquired using the [68Ga]Ga- PSMA-11 tracer, and retest scan was taken using the [18F]F-PSMA-1007 tracer. PET, positron emission tomography; PSMA, prostate-specific membrane antigen.

Patient lesions in both test and retest scans were segmented using the MIM Encore software (MIM Software Inc., Cleveland, OH) by an expert nuclear medicine physician (JO). Suspected lesion sites were interpreted according to published E-PSMA guidelines,^22^ where sites deemed ‘probably’ or ‘definitely’ positive were delineated for inclusion in the study. The delineation process followed a semi-automated methodology, beginning with a baseline global 3 SUV_bw_ threshold applied to the PET image. This was followed by the manual removal of included physiological uptake areas, and the addition of any pathologic uptake missed during thresholding. Small lesion contours <1.5 cm^3^ in volume as measured on the PET scan were not used in the final analysis.^23,24^ Figure 1 shows an example of a test–retest lesion segmentation.

### Image processing

Binary masks for each segmented lesion were created using Plastimatch v. 1.9.4, an open-source medical image processing package. All PET image voxel values were converted into SUV units normalised to the patient body weight.

Feature extraction was separated into two components: first, conventional PET quantitative parameters (SUV_max_, SUV_mean_, SUV_total_, and volume) were calculated at the lesion level on the original PET images without resampling to avoid the introduction of resampling errors. SUV_total_ is defined as the SUV_mean_ multiplied by the lesion volume. Then, a suite of radiomics features were extracted using the open source PyRadiomics python package^25^ v. 3.0.1 whose feature definitions closely align with the Image Biomarker Standardisation Initiative (IBSI) framework.^26^ Prior to radiomics feature extraction, PET images were resampled to an isotropic voxel spacing of 2 × 2 × 2 mm^3^ using B-spline interpolation, and corresponding binary masks were resampled to the same spacing using nearest neighbour interpolation. Interpolation to isotropic spacing is necessary to ensure that texture features are rotationally invariant and makes comparisons with other studies easier.^27^

A total of 107 radiomics features were extracted from the original PET image, which are broken down into the following feature subcategories: shape features (*n* = 14), first-order statistics (*n* = 18), grey level co-occurrence matrix (GLCM) features (*n* = 24), grey level dependence matrix (GLDM) features (*n* = 14), grey level run length matrix (GLRLM) features (*n* = 16), grey level size zone matrix (GLSZM) features (*n* = 16) and neighbourhood grey tone difference matrix (NGTDM) features (*n* = 5). SUV values in the defined volumes of interest were discretised using a constant bin width of 0.2 for texture feature calculation. The effect of applying various filters to the PET image prior to feature extraction was also investigated. 16 different types of filters were tested, including: exponential, Laplacian of Gaussian (LoG, σ = 2, 3, 4, 5 mm), logarithm, square, square root and eight different wavelet transformations. Shape-based features are unaffected by the application of filters, and thus an additional 1488 features were extracted per lesion.

### Statistical analysis

Repeatability of quantitative metrics were analysed at the lesion level using the within-subject coefficient of variation (wCV), the repeatability coefficient (RC) and the intraclass correlation coefficient (ICC). The ICC values were calculated using a two-way mixed effects model with absolute agreement definition based on single rater measurements.^28^ RCs and wCVs were calculated using the methodology previously described in Lodge and Obuchowski^29,30^ that has been utilised in other PET radiotracer repeatability investigations.^24,31^ RC values define the threshold for biomarker change within which 95% of test–retest variability lies. 95% upper and lower limits of agreement (LOA) are then calculated as follows:

95\% LOA=[B−RC,B+RC],

where B is defined as the mean of the relative differences between test and retest values for that particular biomarker. Relative differences are quantified as described in Pollard et al.^31^

ICC values were interpreted using the Koo and Li classification scheme,^28^ where: ICC ≥0.9 corresponds to ‘excellent’ reliability; 0.75 ≤ ICC <0.9 corresponds to ‘good’ reliability; 0.5 ≤ ICC <0.75 corresponds to ‘moderate’ reliability; and ICC <0.5 corresponds to ‘poor’ reliability. For a robust characterisation of biomarker reliability, the lower end of the calculated ICC 95% confidence interval was used for the classification. Wilcoxon signed-rank tests with Bonferroni corrections applied for multiple testing were used to compare the effect of image filters on ICC distributions using the SciPy package v. 1.7.3. Spearman rank correlation was used to assess correlation between texture features and lesion volume using the SciPy package as well. RC calculations were performed in Python v. 3.9. ICC values with confidence intervals were calculated using the Psych package v. 2.2.5 in R v. 3.3.1.

## Results

### Patient characteristics

In total, 75 lesions were identified from 18 different patients and analysed in this prospective study. Most lesions were in the bones (45/75, 60%), followed by nodal disease (23/75, 30.7%) and local prostate lesions (7/75, 9.3%). There was a median number of 1 lesion per patient (range: 0–19). Following randomisation, five patients were assigned to each possible radiotracer combination, except for the [^18^F]F-PSMA-1007 – [^68^Ga]Ga-PSMA-11 group to which three patients were assigned. Due to the low number of lesions present in this group (*n* = 3), a combined ‘intertracer’ group was created by pooling these lesions together with those of the [^68^Ga]Ga-PSMA-11 – [^18^F]F-PSMA-1007 patients (*n* = 33). Patient retest scans were taken a median of 5 days (range: 2–7 days) after their test scan. A summary of patient demographic variables and pertinent test–retest imaging parameters is provided for each radiotracer combination in Table 1. Two lesions from a patient in the [^68^Ga]Ga-PSMA-11 – [^18^F]F-PSMA-1007 group (a nodal lesion above the diaphragm to the left neck, and a skeletal lesion in the ribs) were visible on the test scan, but not the retest scan, and as such had to be excluded from analysis. These lesions are not included in the 75 total.

**Table 1.** Patient demographic characteristics, lesion types, and pertinent test–retest imaging parameters for all possible radiotracer combinations

Characteristic	All patients (*n* = 18)	Intratracer ^68^Ga-PSMA-11 (*n* = 5)	^68^Ga-PSMA-11 – ^18^F-PSMA-1007 (*n* = 5)	^18^F-PSMA-1007 – ^68^Ga-PSMA-11 (*n* = 3)	Intratracer ^18^F-PSMA-1007 (*n* = 5)
Age (y)	70.5 (65–86)	70 (66–80)	80 (68–86)	68 (65–78)	69 (65–81)
Weight (Kg)	91.5 (59–125)	78 (77–112)	95 (59–108)	80 (70–125)	96 (75–107)
Disease type					
CRPC	6 (33.3%)	3 (60%)	2 (40%)	1 (33.3%)	0 (0%)
CSPC	12 (66.7%)	2 (40%)	3 (60%)	2 (66.7%)	5 (100%)
PSA (ng/ml)	4.75 (0.26–57)	5 (0.41–24)	15 (0.84–57)	2.3 (0.26–54)	3.8 (0.86–10)
PSA doubling time (months)	2 (0.6–35)	1.6 (0.9–13.5)	2 (0.6–35)	1.2 (1–1.8)	5 (1.2–32.6)
Lesion types					
Local prostate	7 (9.3%)	3 (12.5%)	2 (6.1%)	0 (0%)	2 (13.3%)
Lymph node	23 (30.7%)	3 (12.5%)	9 (27.3%)	0 (0%)	11 (73.3%)
Osseous	45 (60.0%)	18 (75.0%)	22 (66.7%)	3 (100%)	2 (13.3%)
Injected activity (MBq)					
Test	188 (106–408)	139 (138–195)	153 (106–179)	320 (275–400)	385 (294–408)
Retest	268 (123–402)	133 (129–185)	379 (229–400)	129 (124–210)	383 (306–402)
Difference a		9 (4–23)			5 (2–12)
Uptake time (min)					
Test	94 (63–166)	64 (63–83)	84 (66–104)	146 (129–166)	125 (104–142)
Retest	117 (59–152)	89 (59–95)	138 (127–152)	79 (61–79)	120 (116–147)
Difference a		11 (3–28)			4 (1–21)

CRPC: castration-resistant prostate cancer, CSPS: castration-sensitive prostate cancer, PSA: prostate-specific antigen.

Continuous data are presented as the median with the range in parentheses, and discrete data are presented as the number of that variable with percentages of the total in parentheses.

a The difference between test and retest uptake time and injected activity is shown only for intratracer scan groups.

### Conventional PET quantitative parameters

Repeatability metrics calculated for conventional PET quantitative parameters at the lesion level are summarised in Table 2. According to the RC, SUV_mean_ showed the highest overall repeatability amongst the groups with symmetric RCs of 7.9%, 14.2% and 24.7% for the intratracer [^68^Ga]Ga-PSMA-11, intratracer [^18^F]F-PSMA-1007, and intertracer groups, respectively. This was followed by the SUV_max_ with symmetric RCs of 16.3%, 27.6% and 43.4% for the respective groups. Volume-based quantitative metrics SUV_total_ and Volume had much larger RC limits than SUV_mean_ and SUV_max_ for all tracer groups. Regardless of the quantitative metric analysed, the intertracer group presented with larger RC limits than the two intratracer groups. ICC values were consistently highest for the volume-based metrics (SUV_total_ and Volume) as compared to SUV_max_ and SUV_mean_ for all tracer groups.

**Table 2.** Repeatability metrics of conventional PET imaging biomarkers SUV_max_, SUV_mean_, SUV_total_, and volume for each tracer combination

Group/Biomarker	wCV (%)	RC (%)	Lower LOA (%)	Upper LOA (%)	ICC*
Intratracer ^68^Ga-PSMA-11					
SUV_max_	5.9	16.3	–20.5	+12.2	0.986 [0.966,0.994]
SUV_mean_	2.8	7.9	–8.6	+7.2	0.995 [0.989,0.998]
SUV_total_	7.1	19.6	–28.4	+10.7	0.998 [0.995,0.999]
Volume	6.9	19.3	–27.4	+11.1	0.998 [0.994,0.999]
Intratracer ^18^F-PSMA-1007					
SUV_max_	9.9	27.6	–25.2	+29.9	0.977 [0.934,0.992]
SUV_mean_	5.1	14.2	–14.8	+13.6	0.97 [0.915,0.99]
SUV_total_	16.2	44.9	–32.7	+57.2	0.982 [0.948,0.994]
Volume	12.6	34.9	–22.0	+47.9	0.995 [0.986,0.998]
Intertracer					
SUV_max_	15.6	43.4	–12.4	+74.3	0.68 [0.157,0.866]
SUV_mean_	8.9	24.7	–9.7	+39.7	0.782 [0.142,0.924]
SUV_total_	20.5	56.9	–13.6	+100.1	0.909 [0.828,0.953]
Volume	15.8	43.7	–14.5	+72.9	0.986 [0.971,0.993]

* 95% confidence intervals are provided in square brackets.

### Radiomics features

**Figure 2.**
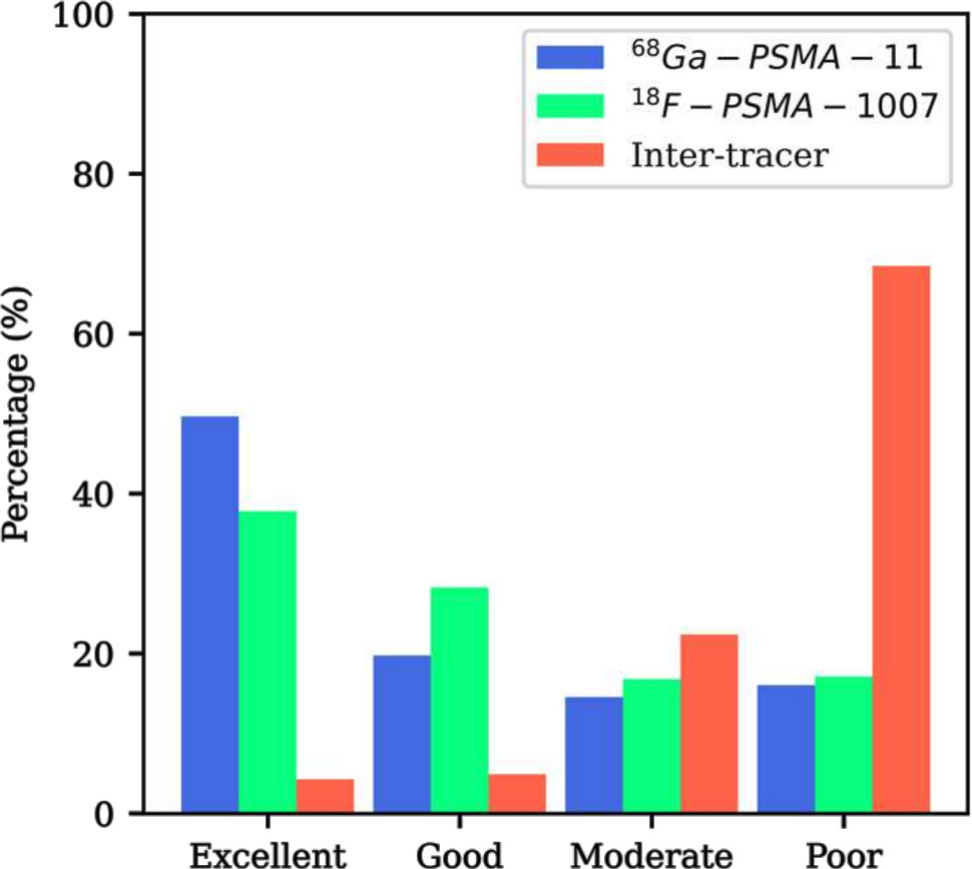
Classification of the ICC values for all radiomics features extracted from original and filtered PET images for all tracer groups. Features are classified using the lower end of the ICC 95% confidence interval as follows: ICC ≥0.9 corresponds to ‘excellent’ reliability; 0.75 ≤ ICC <0.9 corresponds to ‘good’ reliability; 0.5 ≤ ICC <0.75 corresponds to ‘moderate’ reliability; and ICC <0.5 corresponds to ‘poor’ reliability.^28^ ICC, intraclass correlation coefficient; PET, positron emmision tomography.

For both intratracer groups ([^68^Ga]Ga-PSMA-11 and [^18^F]F-PSMA-1007), the total number of features extracted from the unfiltered image rated as excellent or good reliability were similar (69% and 66%, respectively), with [^68^Ga]Ga-PSMA-11 having a higher percentage of excellent features relative to [^18^F]F-PSMA-1007 (50% *vs* 38%). A considerable decrease in feature reliability as measured by the ICC values was found for the intertracer group, where only 9% of all features were classified as either excellent or good. Classifications of all features for the three radiotracer groups are presented in Figure 2. Raw ICC values, RCs, LOAs and wCV percentages for all radiomics features are presented in 1 [Supplementary-material suppl4]-3 for the three tracer groups.

Supplementary Table 1.

Supplementary Table 2.

Supplementary Table 3.

**Figure 3.**
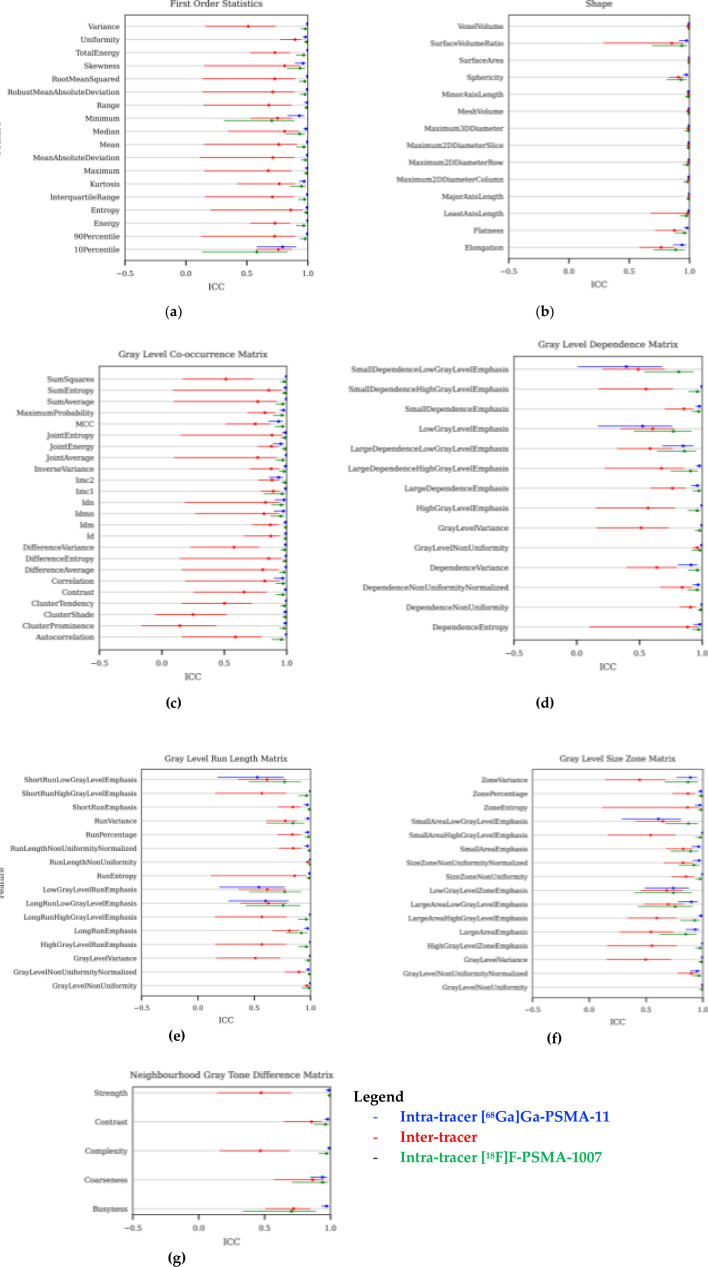
ICC values and their associated 95% confidence intervals plotted for features extracted from the original, unfiltered PET image for all radiotracer combinations. Separate plots are provided for each feature family: (**a**) first-order statistics; (**b**) shape features; (**c**) grey level co-occurrence matrix; (**d**) grey level dependence matrix; (**e**) grey level run length matrix; (**f**) grey level size zone matrix, and; (**g**) neighbourhood grey tone difference matrix. For each feature, intratracer [^68^Ga]Ga- PSMA-11 ICCs are on the top, intertracer in the middle, and intratracer [^18^F]F-PSMA-1007 on the bottom. ICC, intraclass correlation coefficient; PET, positron emmision tomography.

ICC values and their associated confidence intervals for features extracted only from the original, unfiltered image are plotted in Figure 3 for all radiotracer groups. Shape-based features performed much better than other feature families, and were consistently in the top two for the number of features rated as either excellent or good (14/14 = 100% for [^68^Ga]Ga-PSMA-11; 12/14 = 85.7% for [^18^F]F-PSMA-1007; 10/14 = 71.4% for intertracer scans). Among the two intratracer groups, GLCM texture features had the highest percentage of excellent or good feature classifications (24/24 = 100% for [^68^Ga]Ga-PSMA-11; 24/24 = 100% for [^18^F]F-PSMA-1007), however, the intertracer group diverged significantly from this good performance (3/24 = 12.5%). An even larger difference was seen in the first-order features (17/18 = 94.4% for [^68^Ga]Ga-PSMA-11; 16/18 = 88.9% for [^18^F]F-PSMA-1007; 1/18 = 5.6% for intertracer).

**Figure 4.**
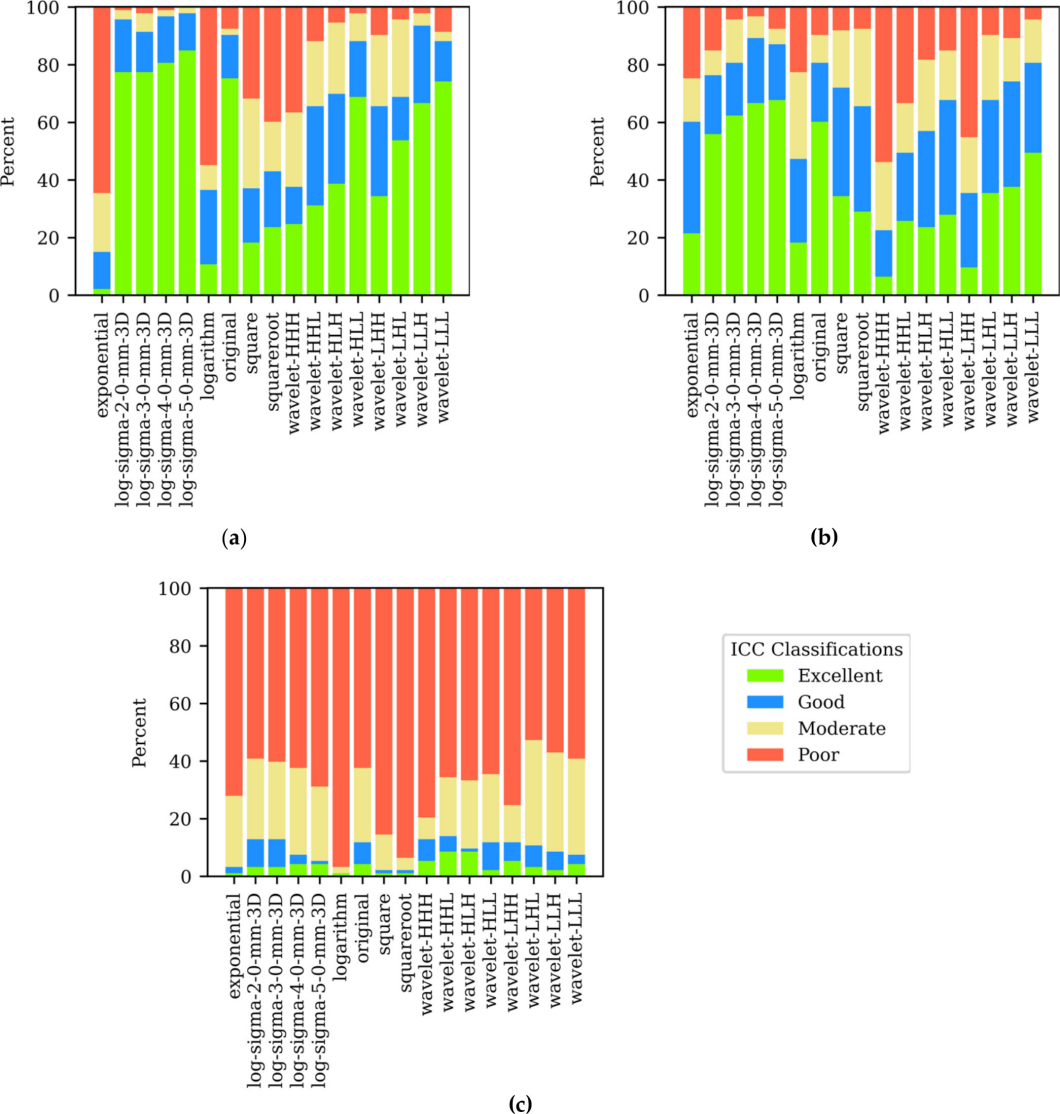
Stacked boxplots showing how the distribution of feature calculations changes as different filters are applied to the original image prior to feature extraction for (**a**) intratracer [^68^Ga]Ga- PSMA-11; (**b**) intratracer [^18^F]F-PSMA-1007; (**c**) intertracer scans. Shape-based features are not included in these plots since they are insensitive to the application of image filters. PSMA, prostate-specific membrane antigen.

The effect of applying 16 different filters to the original PET image on the feature reliability classifications is presented in Figure 4. Of the additional 1488 features extracted, [^68^Ga]Ga-PSMA-11 had the highest percentage of excellent or good features (1009/1488, 67.8%), followed by the [^18^F]F-PSMA-1007 (967/1488, 65.0%), with the intertracer group performing by far the worst (125/1488, 8.4%). None of the filters employed increased ICC values for any of the three tracer groups to statistical significance. Heatmaps showing the change in raw ICC values for the two intratracer groups and the intertracer group with different filtering techniques are shown in Supplementary Figures 1 and 2, respectively.

Supplementary Figure 1.

Supplementary Figure 2.

**Figure 5.**
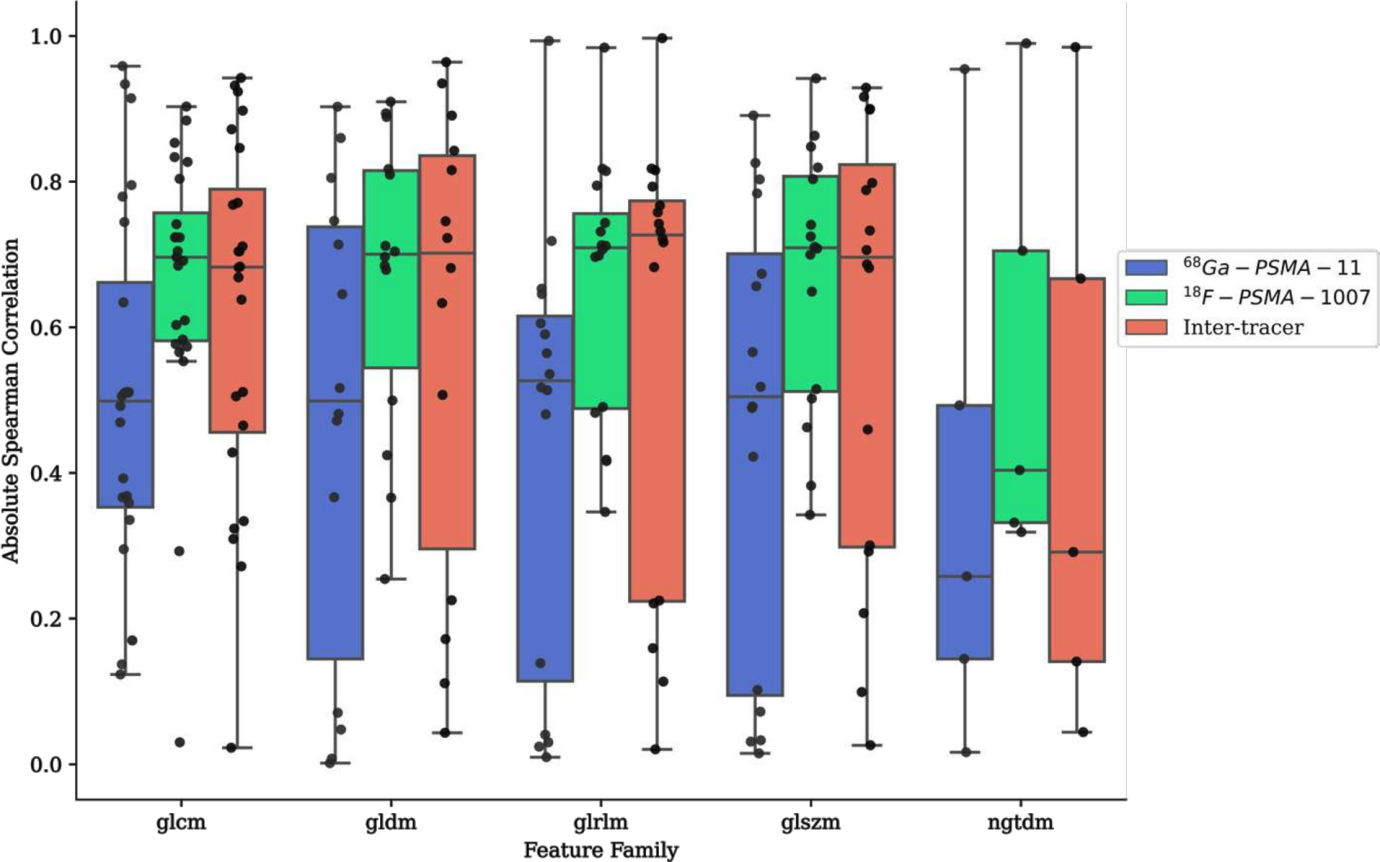
Absolute value of spearman correlation of all texture features with lesion volume, stratified by the texture feature matrix type. Results are presented for all radiotracer groups on texture features extracted from the original image. PSMA, prostate-specific membrane antigen.

Absolute value spearman rank correlation of all texture features extracted from the original image with lesion volume is presented in Figure 5 to assess feature redundancy with tumour volume. Individual texture features exhibited varying degrees of correlation with tumour volume, with some features showing very strong correlation (*glcm_Correlation,* r_s_ = 0.96; *gldm_DependenceNonUniformity*, r_s_ = 0.90), and others showing negligible volume dependence (*ngtdm_Contrast,* r_s_ = 0.04; *gldm_DependenceVariance*, r_s_ = 0.07). Overall, keeping the tracer combination constant, there was minimal difference in the median correlation with volume between texture matrix families with the exception of the NGTDM, which exhibited the lowest volume dependence of the matrix families.

## Discussion

In this prospective study, the repeatability of quantitative metrics extracted from [^68^Ga]Ga-PSMA-11 and [^18^F]F-PSMA-1007 PET scans (both inter- and intratracer) were quantified in a test–retest setting. Previous studies have characterised the repeatability of conventional SUV metrics in several PSMA-targeting tracers,^24,31–33^ though this study is the first to do so for [^18^F]F-PSMA-1007 to our knowledge. A recent study by Werner et al^33^ examined a test–retest cohort of ^18^F-DCFPyL PET images from 21 PCa patients and calculated wCV percentages of 7.3 and 12.1% for SUV_mean_ and SUV_max_ respectively. This is comparable to the wCV values achieved for the intratracer [^18^F]F-PSMA-1007 PET scans in this study (wCV = 5.1 and 9.9% for SUV_mean_ and SUV_max_, respectively). Another earlier study by Jansen et al^24^ analysing test–retest repeatability of lesion-level metrics in ^18^F-DCFPyL PET images determined RC limits of 24.4%, 31% and 28.1% for SUV_mean_, SUV_max_, and volume, respectively, which is again comparable to those demonstrated in the present study for [^18^F]F-PSMA-1007 (RC = 14.2%, 27.6% and 34.9% for SUV_mean_, SUV_max_, and volume, respectively), albeit with a lower RC value for the SUV_mean_ found in this study. The repeatability metrics established for SUV_max_, SUV_mean_ and volume of [^18^F]F-PSMA-1007 lesions in this study therefore appear to be in line with previous investigations of other ^18^F-labelled PSMA ligands. These RC limits can be used to inform treatment response scenarios in clinical practice using [^18^F]F-PSMA-1007 PET images, where percentage SUV or volume changes outside of these bounds reflects either lesion progression or response.

For [^68^Ga]Ga-PSMA-11, Pollard et al^31^ calculated RC limits of 32.5 and 37.9% for SUV_max_ in bone and nodal lesions, respectively, which is greater than what was calculated in this study (RC of 16.3% for all lesion types combined). However, their study included lesions of all sizes and had a substantially greater sample size (136 *vs* 24), with the RC limits subgrouped by lesion type, which makes a direct comparison between the two studies difficult. We also found that regardless of the tracer group, smaller RC limits and wCV (%) values were found for SUV metrics (SUV_max_ and SUV_mean_) relative to the volume-based metrics (SUV_total_ and volume), suggesting that they might be better suited for use in response assessment settings. This is in line with the Werner et al study^33^ and the investigation into the lesion-level repeatability of ^18^F-NaF PET metrics by Lin et al.^23^

An important finding of the present study is that feature repeatability overall diminishes greatly when comparing biomarker values extracted from the same lesion between test–retest scans with different tracers. Intertracer RC limits for SUV_max_ and volume were 43.4 and 43.7%, respectively, which are considerably greater than their intratracer counterparts. This has direct implications for current clinical practice, especially in locations where both tracers investigated in this study are approved for clinical use. These repeatability limits are inconsistent with the use of, for instance, the PPP criteria for determining patient progression, which includes as one of its progression criteria a 30% or greater increase in either size or uptake of 1 or more lesions between baseline and follow-up.^15^ Findings of the present study have shown that a 30% change is insufficient to determine true biological change when comparing intertracer scans. Therefore, it is recommended that in any clinical treatment response scenarios, interpretation of quantitative values across different tracer types must be performed cautiously. If such situations can’t be avoided, then SUV_mean_ values can be used for quantification, which demonstrated the lowest RC limits of all conventional PET quantitative metrics between the different tracers (24.7%). The difference between inter- and intratracer repeatability levels was perhaps not surprising, since both radiotracer compounds used in this study are known to have different biodistributions, pharmacokinetic profiles, and physical and biological half-lives resulting from differences in their molecular composition that can affect compound uptake.^9,14,34^

Radiomics models derived from quantitative imaging biomarkers, despite showing great potential, are yet to be introduced into regular clinical practice. This is partly due to a lack of statistical characterisation of the repeatability of these features, which can be used to identify robust imaging biomarkers for inclusion into radiomics models. It is common, *e.g.* to employ thresholds of calculated ICC values in a test–retest data set to exclude features without a sufficient level of repeatability from the radiomics model building process, which can ensure that models are comprised of robust features and reduce the chances of overfitting.^35–37^ This work aims to facilitate the development of robust radiomics models with the repeatability metrics reported here. Researchers conducting future radiomics analysis using the tracers analysed here can use the raw repeatability data provided to guide them in the selection of robust imaging biomarkers for their predictive tasks. Furthermore, LOAs derived for each of these radiomic features can serve as the minimum threshold of percentage change in each biomarker for use in response assessment settings, which future works can utilise.

The repeatability characteristics of some feature families and individual features are worth highlighting. First-order statistics exhibited high repeatability only for the intratracer groups, which is consistent with the results of a comprehensive systematic review of radiomics feature repeatability and reproducibility by Traverso et al^38^ that identified first-order statistics as being consistently the most stable feature extracted from PET images. However, only one feature (*Uniformity*) from this feature class showed good repeatability in the intertracer analysis. The first-order feature *Kurtosis*, which showed high repeatability in both intratracer groups, has shown prognostic value in overall survival prediction for PCa patients undergoing ^177^Lu-PSMA therapy in a previous study.^20^ Entropy-based features from the GLCM feature family also demonstrated very high levels of repeatability for both intratracer groups. This is important, because a previous work by Khurshid et al^21^ showed that the texture feature *GLCM Entropy* negatively correlates with changes in PSA values after ^177^Lu-PSMA therapy, suggesting that this feature could be used as a surrogate for response assessment. Such features, which have high repeatability as well as significant correlations with relevant clinical endpoints, are of considerable interest, and further studies should elucidate their full predictive potential.

Another finding of this work is that applying filters to the original image prior to quantifying radiomics features does not improve the overall repeatability of those features. This is in contrast to some repeatability studies conducted for other PET tracers such as O-(2-[^18^F]fluoroethyl)-L-tyrosine (FET) PET and ^18^F-fluoro-2-deoxy-D-glucose (FDG) PET^39,40^ that have shown repeatability benefits following LoG filtering. Therefore, it may not be necessary to apply filtering in future mPCa radiomics studies conducted on either [^68^Ga]Ga-PSMA-11 and [^18^F]F-PSMA-1007 PET features, though this should be the subject of further investigation.

This study does have some limitations that should be noted. The study population had a relatively low disease burden meaning that patients with a high disease burden disproportionately contributed to the repeatability results. The sample size of patients was also quite small for each group and came from a single institution. Moreover, subgroup analysis at the lesion level was prevented by the fact that some tracer groups had low numbers of particular lesion types. For example, nodal lesions comprised almost the entirety of the lesions in the [^18^F]F-PSMA-1007 group (11/15, 73.3%), with only two osseous lesions present. Future studies with larger patient cohorts should rectify this issue by having sufficient numbers of all lesion types such that repeatability metrics can be subgrouped by lesion type. There was also a wide variation in tracer uptake time for both intratracer groups, highlighting the difficulty of adherence to a strict imaging protocol in a busy hospital clinic. Although this could have affected the repeatability of quantitative metrics, it is also worth noting that this is more likely to reflect real-world treatment response scenarios, where a precise matching of uptake times between baseline and follow-up imaging is unlikely to occur.

## Conclusion

In this prospective study, the inter- and intratracer repeatability of radiomics features extracted from mPCa lesions delineated in [^68^Ga]Ga-PSMA-11 and [^18^F]F-PSMA-1007 PET scans was quantified. SUV_mean_ was demonstrated to have the lowest RC limits of the conventional quantitative features. For both intratracer groups, high percentages of repeatable radiomics features were found, but substantial reductions in feature repeatability were found for the intertracer group. The application of filters to images did not significantly increase feature ICC values. The repeatability metrics established in this work can serve as the basis for selecting robust features for investigation in future clinical trials. Further studies with greater sample sizes of lesions should be conducted to validate the results of this work.
