# Supplementary material for: Prospective inter- and intra-tracer repeatability analysis of radiomics features in [68Ga]Ga-PSMA-11 and [18F]F-PSMA-1007 PET scans in metastatic prostate cancer
Source: Br J Radiol. 2023 Oct 24;96(1152):20221178. doi: 10.1259/bjr.20221178 (PMC10646662; doi:10.1259/bjr.20221178)
Supplement: Supplementary Table 1. [file bjr.20221178.suppl-03.docx]

**Supplementary Table S1**

Tabulated below are the raw repeatability metrics for the ^68^Ga-PSMA-11 intra-tracer group, including: intraclass correlation coefficients (ICCs), symmetric repeatability coefficients (RCs), within-subject coefficient of variation (wCV) values, upper and lower limits of agreement (LOA).

| **Biomarker** | **wCV (%)** | **Symmetric RC (%)** | **ICC** | **ICC 95% Lower** | **ICC 95% Upper** | **Upper LOA** | **Lower LOA** |
| --- | --- | --- | --- | --- | --- | --- | --- |
| original_shape_Elongation | 4.64 | 12.85 | 0.937 | 0.861 | 0.972 | 11.8 | -13.9 |
| original_shape_Flatness | 4.08 | 11.32 | 0.98 | 0.954 | 0.991 | 10.4 | -12.2 |
| original_shape_LeastAxisLength | 3.77 | 10.45 | 0.993 | 0.982 | 0.997 | 7.4 | -13.5 |
| original_shape_MajorAxisLength | 2.75 | 7.63 | 0.998 | 0.994 | 0.999 | 5.5 | -9.8 |
| original_shape_Maximum2DDiameterColumn | 4.45 | 12.33 | 0.994 | 0.987 | 0.998 | 10.2 | -14.4 |
| original_shape_Maximum2DDiameterRow | 4.50 | 12.47 | 0.995 | 0.989 | 0.998 | 11.1 | -13.8 |
| original_shape_Maximum2DDiameterSlice | 4.02 | 11.15 | 0.994 | 0.985 | 0.997 | 8.8 | -13.5 |
| original_shape_Maximum3DDiameter | 3.49 | 9.67 | 0.995 | 0.988 | 0.998 | 8.4 | -11 |
| original_shape_MeshVolume | 7.75 | 21.49 | 0.996 | 0.992 | 0.998 | 12.7 | -30.3 |
| original_shape_MinorAxisLength | 4.02 | 11.15 | 0.992 | 0.974 | 0.997 | 7.9 | -14.4 |
| original_shape_Sphericity | 1.61 | 4.47 | 0.975 | 0.944 | 0.989 | 4.6 | -4.3 |
| original_shape_SurfaceArea | 5.53 | 15.33 | 0.998 | 0.996 | 0.999 | 9.3 | -21.4 |
| original_shape_SurfaceVolumeRatio | 2.98 | 8.25 | 0.975 | 0.912 | 0.991 | 11 | -5.5 |
| original_shape_VoxelVolume | 7.58 | 21.01 | 0.996 | 0.992 | 0.998 | 12.4 | -29.6 |
| exponential_firstorder_10Percentile | 1.74 | 4.82 | 0.866 | 0.357 | 0.957 | 7.3 | -2.3 |
| exponential_firstorder_90Percentile | 8.28 | 22.96 | 0.81 | 0.532 | 0.921 | 35.7 | -10.2 |
| exponential_firstorder_Energy | 18.11 | 50.19 | 0.832 | 0.648 | 0.924 | 62.8 | -37.6 |
| exponential_firstorder_Entropy | 9.01 | 24.98 | 0.905 | 0.405 | 0.972 | 37 | -13 |
| exponential_firstorder_InterquartileRange | 13.80 | 38.24 | 0.862 | 0.5 | 0.951 | 62.4 | -14 |
| exponential_firstorder_Kurtosis | 12.68 | 35.14 | 0.735 | 0.483 | 0.875 | 38.4 | -31.9 |
| exponential_firstorder_Maximum | 17.53 | 48.59 | 0.753 | 0.449 | 0.892 | 68.8 | -28.4 |
| exponential_firstorder_MeanAbsoluteDeviation | 16.47 | 45.66 | 0.82 | 0.567 | 0.924 | 71.2 | -20.1 |
| exponential_firstorder_Mean | 5.71 | 15.84 | 0.797 | 0.391 | 0.923 | 24.4 | -7.3 |
| exponential_firstorder_Median | 3.20 | 8.88 | 0.788 | 0.061 | 0.936 | 14.7 | -3.1 |
| exponential_firstorder_Minimum | 0.94 | 2.61 | 0.925 | 0.341 | 0.98 | 4.3 | -0.9 |
| exponential_firstorder_Range | 20.93 | 58.01 | 0.757 | 0.458 | 0.893 | 85.5 | -30.5 |
| exponential_firstorder_RobustMeanAbsoluteDeviation | 13.99 | 38.79 | 0.842 | 0.562 | 0.937 | 62.7 | -14.9 |
| exponential_firstorder_RootMeanSquared | 7.87 | 21.82 | 0.77 | 0.458 | 0.902 | 32.5 | -11.1 |
| exponential_firstorder_Skewness | 15.13 | 41.93 | 0.889 | 0.764 | 0.95 | 38.8 | -45.1 |
| exponential_firstorder_TotalEnergy | 18.11 | 50.19 | 0.832 | 0.648 | 0.924 | 62.8 | -37.6 |
| exponential_firstorder_Uniformity | 11.82 | 32.77 | 0.905 | 0.708 | 0.963 | 17.7 | -47.8 |
| exponential_firstorder_Variance | 31.76 | 88.02 | 0.551 | 0.211 | 0.775 | 137.4 | -38.7 |
| exponential_glcm_Autocorrelation | 25.65 | 71.10 | 0.573 | 0.242 | 0.788 | 115.7 | -26.5 |
| exponential_glcm_ClusterProminence | 48.97 | 135.73 | 0.181 | -0.227 | 0.537 | 216.8 | -54.7 |
| exponential_glcm_ClusterShade | 237.25 | 657.63 | 0.319 | -0.08 | 0.633 | 623.9 | -691.3 |
| exponential_glcm_ClusterTendency | 33.91 | 93.99 | 0.55 | 0.209 | 0.775 | 136.8 | -51.2 |
| exponential_glcm_Contrast | 30.42 | 84.33 | 0.513 | 0.163 | 0.752 | 130.6 | -38.1 |
| exponential_glcm_Correlation | 6.92 | 19.18 | 0.944 | 0.876 | 0.975 | 16.5 | -21.8 |
| exponential_glcm_DifferenceAverage | 16.84 | 46.67 | 0.795 | 0.441 | 0.919 | 71.5 | -21.8 |
| exponential_glcm_DifferenceEntropy | 8.65 | 23.98 | 0.903 | 0.383 | 0.971 | 37.5 | -10.5 |
| exponential_glcm_DifferenceVariance | 31.60 | 87.60 | 0.495 | 0.14 | 0.741 | 132.2 | -43 |
| exponential_glcm_Id | 5.02 | 13.92 | 0.909 | 0.348 | 0.974 | 5.6 | -22.2 |
| exponential_glcm_Idm | 6.41 | 17.77 | 0.909 | 0.329 | 0.975 | 7.1 | -28.5 |
| exponential_glcm_Idmn | 0.50 | 1.38 | 0.805 | 0.601 | 0.911 | 1.6 | -1.1 |
| exponential_glcm_Idn | 0.64 | 1.76 | 0.946 | 0.881 | 0.976 | 1.9 | -1.6 |
| exponential_glcm_Imc1 | 8.99 | 24.92 | 0.85 | 0.688 | 0.932 | 21.5 | -28.4 |
| exponential_glcm_Imc2 | 6.23 | 17.28 | 0.928 | 0.842 | 0.968 | 18 | -16.5 |
| exponential_glcm_InverseVariance | 8.35 | 23.14 | 0.856 | 0.699 | 0.935 | 20.3 | -26 |
| exponential_glcm_JointAverage | 12.33 | 34.17 | 0.799 | 0.386 | 0.924 | 55.9 | -12.4 |
| exponential_glcm_JointEnergy | 22.03 | 61.05 | 0.89 | 0.765 | 0.95 | 36 | -86.1 |
| exponential_glcm_JointEntropy | 8.98 | 24.89 | 0.916 | 0.502 | 0.975 | 35.1 | -14.7 |
| exponential_glcm_MCC | 6.20 | 17.19 | 0.947 | 0.883 | 0.977 | 16 | -18.4 |
| exponential_glcm_MaximumProbability | 16.24 | 45.03 | 0.893 | 0.772 | 0.952 | 33.1 | -57 |
| exponential_glcm_SumAverage | 12.33 | 34.17 | 0.799 | 0.386 | 0.924 | 55.9 | -12.4 |
| exponential_glcm_SumEntropy | 7.89 | 21.88 | 0.922 | 0.608 | 0.975 | 29.8 | -14 |
| exponential_glcm_SumSquares | 32.83 | 91.00 | 0.547 | 0.205 | 0.773 | 134.6 | -47.4 |
| exponential_gldm_DependenceEntropy | 3.00 | 8.31 | 0.924 | 0.821 | 0.967 | 10 | -6.6 |
| exponential_gldm_DependenceNonUniformity | 13.79 | 38.24 | 0.985 | 0.967 | 0.994 | 35.3 | -41.2 |
| exponential_gldm_DependenceNonUniformityNormalized | 9.06 | 25.12 | 0.914 | 0.755 | 0.966 | 30.7 | -19.5 |
| exponential_gldm_DependenceVariance | 17.31 | 47.97 | 0.884 | 0.754 | 0.948 | 40.9 | -55.1 |
| exponential_gldm_GrayLevelNonUniformity | 12.68 | 35.14 | 0.833 | 0.651 | 0.925 | 11.6 | -58.6 |
| exponential_gldm_GrayLevelVariance | 30.82 | 85.43 | 0.551 | 0.21 | 0.775 | 130.8 | -40 |
| exponential_gldm_HighGrayLevelEmphasis | 25.85 | 71.65 | 0.571 | 0.238 | 0.786 | 116.4 | -26.9 |
| exponential_gldm_LargeDependenceEmphasis | 14.85 | 41.15 | 0.891 | 0.678 | 0.958 | 14.5 | -67.8 |
| exponential_gldm_LargeDependenceHighGrayLevelEmphasis | 14.48 | 40.14 | 0.879 | 0.74 | 0.946 | 44.5 | -35.8 |
| exponential_gldm_LargeDependenceLowGrayLevelEmphasis | 28.55 | 79.12 | 0.477 | 0.092 | 0.737 | 23.2 | -135.1 |
| exponential_gldm_LowGrayLevelEmphasis | 15.74 | 43.62 | 0.491 | -0.043 | 0.78 | 16.1 | -71.1 |
| exponential_gldm_SmallDependenceEmphasis | 24.48 | 67.85 | 0.743 | 0.148 | 0.91 | 118.4 | -17.3 |
| exponential_gldm_SmallDependenceHighGrayLevelEmphasis | 38.87 | 107.74 | 0.431 | 0.056 | 0.703 | 187.5 | -28 |
| exponential_gldm_SmallDependenceLowGrayLevelEmphasis | 23.93 | 66.34 | 0.451 | -0.073 | 0.758 | 108.3 | -24.4 |
| exponential_glrlm_GrayLevelNonUniformity | 10.08 | 27.93 | 0.961 | 0.876 | 0.985 | 6.4 | -49.4 |
| exponential_glrlm_GrayLevelNonUniformityNormalized | 11.04 | 30.60 | 0.866 | 0.421 | 0.956 | 11 | -50.2 |
| exponential_glrlm_GrayLevelVariance | 28.42 | 78.76 | 0.564 | 0.228 | 0.783 | 128 | -29.5 |
| exponential_glrlm_HighGrayLevelRunEmphasis | 25.14 | 69.69 | 0.593 | 0.269 | 0.799 | 114.6 | -24.7 |
| exponential_glrlm_LongRunEmphasis | 13.59 | 37.68 | 0.814 | 0.586 | 0.918 | 19.8 | -55.6 |
| exponential_glrlm_LongRunHighGrayLevelEmphasis | 19.44 | 53.89 | 0.611 | 0.294 | 0.809 | 82 | -25.8 |
| exponential_glrlm_LongRunLowGrayLevelEmphasis | 25.72 | 71.29 | 0.442 | 0.067 | 0.711 | 25.2 | -117.4 |
| exponential_glrlm_LowGrayLevelRunEmphasis | 13.88 | 38.48 | 0.676 | 0.031 | 0.885 | 13.3 | -63.7 |
| exponential_glrlm_RunEntropy | 3.17 | 8.78 | 0.953 | 0.751 | 0.985 | 12.3 | -5.2 |
| exponential_glrlm_RunLengthNonUniformity | 15.14 | 41.97 | 0.96 | 0.907 | 0.982 | 49.7 | -34.2 |
| exponential_glrlm_RunLengthNonUniformityNormalized | 8.19 | 22.70 | 0.892 | 0.303 | 0.969 | 33.2 | -12.2 |
| exponential_glrlm_RunPercentage | 5.74 | 15.91 | 0.89 | 0.401 | 0.966 | 22.6 | -9.2 |
| exponential_glrlm_RunVariance | 17.60 | 48.78 | 0.758 | 0.513 | 0.888 | 22 | -75.6 |
| exponential_glrlm_ShortRunEmphasis | 4.75 | 13.17 | 0.889 | 0.394 | 0.966 | 19.3 | -7.1 |
| exponential_glrlm_ShortRunHighGrayLevelEmphasis | 26.38 | 73.13 | 0.587 | 0.261 | 0.796 | 123.8 | -22.5 |
| exponential_glrlm_ShortRunLowGrayLevelEmphasis | 10.89 | 30.19 | 0.74 | 0.108 | 0.911 | 12.8 | -47.5 |
| exponential_glszm_GrayLevelNonUniformity | 49.20 | 136.39 | 0.66 | 0.307 | 0.844 | 191 | -81.8 |
| exponential_glszm_GrayLevelNonUniformityNormalized | 29.20 | 80.94 | 0.757 | 0.522 | 0.887 | 83.1 | -78.7 |
| exponential_glszm_GrayLevelVariance | 30.24 | 83.82 | 0.512 | 0.162 | 0.752 | 135 | -32.7 |
| exponential_glszm_HighGrayLevelZoneEmphasis | 31.82 | 88.21 | 0.661 | 0.366 | 0.837 | 109.5 | -66.9 |
| exponential_glszm_LargeAreaEmphasis | 40.59 | 112.52 | 0.274 | -0.119 | 0.6 | 28.2 | -196.8 |
| exponential_glszm_LargeAreaHighGrayLevelEmphasis | 31.61 | 87.61 | 0.613 | 0.296 | 0.811 | 30.3 | -144.9 |
| exponential_glszm_LargeAreaLowGrayLevelEmphasis | 51.37 | 142.40 | 0.087 | -0.301 | 0.459 | 31.6 | -253.2 |
| exponential_glszm_LowGrayLevelZoneEmphasis | 29.74 | 82.42 | 0.698 | 0.375 | 0.863 | 108.6 | -56.3 |
| exponential_glszm_SizeZoneNonUniformity | 42.56 | 117.97 | 0.652 | 0.342 | 0.834 | 175.3 | -60.6 |
| exponential_glszm_SizeZoneNonUniformityNormalized | 32.78 | 90.86 | 0.501 | 0.136 | 0.747 | 98.2 | -83.5 |
| exponential_glszm_SmallAreaEmphasis | 64.79 | 179.60 | 0.572 | 0.124 | 0.807 | 226.1 | -133.1 |
| exponential_glszm_SmallAreaHighGrayLevelEmphasis | 79.37 | 220.01 | 0.578 | 0.248 | 0.79 | 271.5 | -168.5 |
| exponential_glszm_SmallAreaLowGrayLevelEmphasis | 73.24 | 203.01 | 0 | -0.298 | 0.347 | 270.4 | -135.6 |
| exponential_glszm_ZoneEntropy | 15.45 | 42.83 | 0.894 | 0.422 | 0.967 | 58.5 | -27.2 |
| exponential_glszm_ZonePercentage | 35.39 | 98.11 | 0.718 | 0.115 | 0.9 | 160.9 | -35.3 |
| exponential_glszm_ZoneVariance | 33.02 | 91.54 | 0.212 | -0.188 | 0.557 | 21.1 | -162 |
| exponential_ngtdm_Busyness | 31.14 | 86.31 | 0.321 | -0.043 | 0.624 | 22.9 | -149.7 |
| exponential_ngtdm_Coarseness | 10.63 | 29.46 | 0.873 | 0.696 | 0.946 | 39.3 | -19.6 |
| exponential_ngtdm_Complexity | 36.80 | 101.99 | 0.37 | -0.013 | 0.664 | 168.8 | -35.2 |
| exponential_ngtdm_Contrast | 25.65 | 71.11 | 0.619 | 0.305 | 0.814 | 82.5 | -59.7 |
| exponential_ngtdm_Strength | 33.52 | 92.92 | 0.596 | 0.273 | 0.801 | 155.3 | -30.6 |
| log-sigma-2-0-mm-3D_firstorder_10Percentile | 5.70 | 15.80 | 0.991 | 0.979 | 0.996 | 16 | -15.6 |
| log-sigma-2-0-mm-3D_firstorder_90Percentile | 57.91 | 160.51 | 0.977 | 0.901 | 0.992 | 137.9 | -183.1 |
| log-sigma-2-0-mm-3D_firstorder_Energy | 11.55 | 32.01 | 0.992 | 0.981 | 0.997 | 24.2 | -39.8 |
| log-sigma-2-0-mm-3D_firstorder_Entropy | 2.19 | 6.06 | 0.989 | 0.974 | 0.995 | 5.6 | -6.6 |
| log-sigma-2-0-mm-3D_firstorder_InterquartileRange | 6.46 | 17.91 | 0.986 | 0.969 | 0.994 | 19.6 | -16.2 |
| log-sigma-2-0-mm-3D_firstorder_Kurtosis | 9.48 | 26.28 | 0.967 | 0.926 | 0.985 | 21.7 | -30.8 |
| log-sigma-2-0-mm-3D_firstorder_Maximum | 17.36 | 48.11 | 0.947 | 0.878 | 0.977 | 37.3 | -58.9 |
| log-sigma-2-0-mm-3D_firstorder_MeanAbsoluteDeviation | 6.24 | 17.31 | 0.992 | 0.981 | 0.996 | 15.9 | -18.7 |
| log-sigma-2-0-mm-3D_firstorder_Mean | 6.37 | 17.65 | 0.983 | 0.948 | 0.993 | 23.2 | -12.1 |
| log-sigma-2-0-mm-3D_firstorder_Median | 42.42 | 117.59 | 0.963 | 0.841 | 0.987 | 132.3 | -102.9 |
| log-sigma-2-0-mm-3D_firstorder_Minimum | 7.95 | 22.03 | 0.99 | 0.972 | 0.996 | 17.8 | -26.3 |
| log-sigma-2-0-mm-3D_firstorder_Range | 9.10 | 25.21 | 0.989 | 0.965 | 0.996 | 20.6 | -29.8 |
| log-sigma-2-0-mm-3D_firstorder_RobustMeanAbsoluteDeviation | 6.24 | 17.30 | 0.988 | 0.974 | 0.995 | 17.5 | -17.1 |
| log-sigma-2-0-mm-3D_firstorder_RootMeanSquared | 6.09 | 16.88 | 0.993 | 0.984 | 0.997 | 17.3 | -16.5 |
| log-sigma-2-0-mm-3D_firstorder_Skewness | 15.92 | 44.11 | 0.971 | 0.931 | 0.988 | 39.1 | -49.2 |
| log-sigma-2-0-mm-3D_firstorder_TotalEnergy | 11.55 | 32.01 | 0.992 | 0.981 | 0.997 | 24.2 | -39.8 |
| log-sigma-2-0-mm-3D_firstorder_Uniformity | 5.11 | 14.15 | 0.986 | 0.967 | 0.994 | 14.9 | -13.4 |
| log-sigma-2-0-mm-3D_firstorder_Variance | 13.51 | 37.44 | 0.989 | 0.974 | 0.995 | 33 | -41.9 |
| log-sigma-2-0-mm-3D_glcm_Autocorrelation | 17.14 | 47.52 | 0.981 | 0.95 | 0.992 | 37 | -58 |
| log-sigma-2-0-mm-3D_glcm_ClusterProminence | 28.92 | 80.17 | 0.969 | 0.925 | 0.987 | 66.2 | -94.2 |
| log-sigma-2-0-mm-3D_glcm_ClusterShade | 32.65 | 90.51 | 0.98 | 0.947 | 0.992 | 75.6 | -105.5 |
| log-sigma-2-0-mm-3D_glcm_ClusterTendency | 13.71 | 38.01 | 0.989 | 0.971 | 0.995 | 32.5 | -43.5 |
| log-sigma-2-0-mm-3D_glcm_Contrast | 13.19 | 36.57 | 0.986 | 0.969 | 0.994 | 33.5 | -39.7 |
| log-sigma-2-0-mm-3D_glcm_Correlation | 1.94 | 5.39 | 0.981 | 0.955 | 0.992 | 4.3 | -6.5 |
| log-sigma-2-0-mm-3D_glcm_DifferenceAverage | 6.16 | 17.07 | 0.99 | 0.977 | 0.996 | 16.3 | -17.8 |
| log-sigma-2-0-mm-3D_glcm_DifferenceEntropy | 3.53 | 9.79 | 0.99 | 0.977 | 0.996 | 9 | -10.6 |
| log-sigma-2-0-mm-3D_glcm_DifferenceVariance | 15.00 | 41.58 | 0.989 | 0.973 | 0.995 | 36.1 | -47.1 |
| log-sigma-2-0-mm-3D_glcm_Id | 3.32 | 9.21 | 0.991 | 0.979 | 0.996 | 8.8 | -9.7 |
| log-sigma-2-0-mm-3D_glcm_Idm | 4.95 | 13.73 | 0.99 | 0.977 | 0.995 | 12.8 | -14.7 |
| log-sigma-2-0-mm-3D_glcm_Idmn | 0.18 | 0.50 | 0.973 | 0.937 | 0.988 | 0.4 | -0.6 |
| log-sigma-2-0-mm-3D_glcm_Idn | 0.43 | 1.20 | 0.975 | 0.935 | 0.99 | 0.9 | -1.5 |
| log-sigma-2-0-mm-3D_glcm_Imc1 | 3.68 | 10.20 | 0.984 | 0.963 | 0.993 | 11.3 | -9.1 |
| log-sigma-2-0-mm-3D_glcm_Imc2 | 0.98 | 2.73 | 0.982 | 0.958 | 0.992 | 2.8 | -2.7 |
| log-sigma-2-0-mm-3D_glcm_InverseVariance | 4.74 | 13.13 | 0.984 | 0.964 | 0.993 | 11.9 | -14.3 |
| log-sigma-2-0-mm-3D_glcm_JointAverage | 8.86 | 24.56 | 0.988 | 0.962 | 0.995 | 19.1 | -30 |
| log-sigma-2-0-mm-3D_glcm_JointEnergy | 8.70 | 24.12 | 0.971 | 0.935 | 0.987 | 27.1 | -21.1 |
| log-sigma-2-0-mm-3D_glcm_JointEntropy | 2.18 | 6.05 | 0.989 | 0.975 | 0.995 | 5.2 | -6.9 |
| log-sigma-2-0-mm-3D_glcm_MCC | 1.74 | 4.82 | 0.953 | 0.893 | 0.98 | 5.7 | -4 |
| log-sigma-2-0-mm-3D_glcm_MaximumProbability | 17.20 | 47.66 | 0.908 | 0.803 | 0.959 | 52.8 | -42.5 |
| log-sigma-2-0-mm-3D_glcm_SumAverage | 8.86 | 24.56 | 0.988 | 0.962 | 0.995 | 19.1 | -30 |
| log-sigma-2-0-mm-3D_glcm_SumEntropy | 1.93 | 5.35 | 0.99 | 0.976 | 0.995 | 4.6 | -6.1 |
| log-sigma-2-0-mm-3D_glcm_SumSquares | 13.55 | 37.56 | 0.988 | 0.972 | 0.995 | 32.5 | -42.6 |
| log-sigma-2-0-mm-3D_gldm_DependenceEntropy | 1.76 | 4.87 | 0.967 | 0.919 | 0.986 | 3.9 | -5.9 |
| log-sigma-2-0-mm-3D_gldm_DependenceNonUniformity | 8.67 | 24.03 | 0.999 | 0.996 | 0.999 | 17.1 | -30.9 |
| log-sigma-2-0-mm-3D_gldm_DependenceNonUniformityNormalized | 9.31 | 25.82 | 0.905 | 0.795 | 0.957 | 27.5 | -24.1 |
| log-sigma-2-0-mm-3D_gldm_DependenceVariance | 27.95 | 77.48 | 0.841 | 0.672 | 0.928 | 82.4 | -72.6 |
| log-sigma-2-0-mm-3D_gldm_GrayLevelNonUniformity | 10.53 | 29.20 | 0.993 | 0.984 | 0.997 | 21.4 | -37 |
| log-sigma-2-0-mm-3D_gldm_GrayLevelVariance | 13.38 | 37.08 | 0.989 | 0.972 | 0.995 | 32.6 | -41.6 |
| log-sigma-2-0-mm-3D_gldm_HighGrayLevelEmphasis | 16.49 | 45.70 | 0.982 | 0.952 | 0.993 | 35.9 | -55.5 |
| log-sigma-2-0-mm-3D_gldm_LargeDependenceEmphasis | 13.43 | 37.23 | 0.98 | 0.955 | 0.991 | 36.4 | -38.1 |
| log-sigma-2-0-mm-3D_gldm_LargeDependenceHighGrayLevelEmphasis | 23.84 | 66.08 | 0.926 | 0.839 | 0.967 | 56.5 | -75.7 |
| log-sigma-2-0-mm-3D_gldm_LargeDependenceLowGrayLevelEmphasis | 41.74 | 115.71 | 0.988 | 0.973 | 0.995 | 127.6 | -103.8 |
| log-sigma-2-0-mm-3D_gldm_LowGrayLevelEmphasis | 23.96 | 66.42 | 0.955 | 0.901 | 0.98 | 76.9 | -56 |
| log-sigma-2-0-mm-3D_gldm_SmallDependenceEmphasis | 10.50 | 29.11 | 0.956 | 0.893 | 0.982 | 34.8 | -23.4 |
| log-sigma-2-0-mm-3D_gldm_SmallDependenceHighGrayLevelEmphasis | 22.78 | 63.15 | 0.969 | 0.93 | 0.986 | 59.9 | -66.5 |
| log-sigma-2-0-mm-3D_gldm_SmallDependenceLowGrayLevelEmphasis | 19.10 | 52.95 | 0.863 | 0.712 | 0.938 | 57.3 | -48.6 |
| log-sigma-2-0-mm-3D_glrlm_GrayLevelNonUniformity | 9.64 | 26.72 | 0.994 | 0.987 | 0.998 | 19.1 | -34.3 |
| log-sigma-2-0-mm-3D_glrlm_GrayLevelNonUniformityNormalized | 5.42 | 15.03 | 0.981 | 0.957 | 0.992 | 15.8 | -14.3 |
| log-sigma-2-0-mm-3D_glrlm_GrayLevelVariance | 14.14 | 39.20 | 0.989 | 0.974 | 0.996 | 34.3 | -44.1 |
| log-sigma-2-0-mm-3D_glrlm_HighGrayLevelRunEmphasis | 16.38 | 45.41 | 0.982 | 0.952 | 0.993 | 35.7 | -55.2 |
| log-sigma-2-0-mm-3D_glrlm_LongRunEmphasis | 2.97 | 8.23 | 0.982 | 0.96 | 0.992 | 7.4 | -9.1 |
| log-sigma-2-0-mm-3D_glrlm_LongRunHighGrayLevelEmphasis | 16.93 | 46.93 | 0.975 | 0.94 | 0.989 | 36.3 | -57.6 |
| log-sigma-2-0-mm-3D_glrlm_LongRunLowGrayLevelEmphasis | 25.85 | 71.66 | 0.98 | 0.955 | 0.991 | 82.3 | -61 |
| log-sigma-2-0-mm-3D_glrlm_LowGrayLevelRunEmphasis | 22.75 | 63.05 | 0.952 | 0.893 | 0.979 | 73 | -53.1 |
| log-sigma-2-0-mm-3D_glrlm_RunEntropy | 1.66 | 4.62 | 0.985 | 0.966 | 0.994 | 3.9 | -5.4 |
| log-sigma-2-0-mm-3D_glrlm_RunLengthNonUniformity | 6.65 | 18.42 | 0.997 | 0.994 | 0.999 | 10.5 | -26.4 |
| log-sigma-2-0-mm-3D_glrlm_RunLengthNonUniformityNormalized | 1.45 | 4.03 | 0.98 | 0.954 | 0.991 | 4.4 | -3.7 |
| log-sigma-2-0-mm-3D_glrlm_RunPercentage | 0.85 | 2.36 | 0.98 | 0.955 | 0.991 | 2.6 | -2.1 |
| log-sigma-2-0-mm-3D_glrlm_RunVariance | 12.06 | 33.42 | 0.979 | 0.952 | 0.991 | 30.7 | -36.1 |
| log-sigma-2-0-mm-3D_glrlm_ShortRunEmphasis | 0.66 | 1.82 | 0.981 | 0.957 | 0.992 | 1.9 | -1.7 |
| log-sigma-2-0-mm-3D_glrlm_ShortRunHighGrayLevelEmphasis | 16.52 | 45.79 | 0.983 | 0.953 | 0.993 | 36.2 | -55.4 |
| log-sigma-2-0-mm-3D_glrlm_ShortRunLowGrayLevelEmphasis | 21.74 | 60.26 | 0.944 | 0.876 | 0.975 | 70 | -50.5 |
| log-sigma-2-0-mm-3D_glszm_GrayLevelNonUniformity | 10.21 | 28.29 | 0.995 | 0.988 | 0.998 | 25.9 | -30.7 |
| log-sigma-2-0-mm-3D_glszm_GrayLevelNonUniformityNormalized | 8.51 | 23.59 | 0.959 | 0.908 | 0.982 | 25.5 | -21.6 |
| log-sigma-2-0-mm-3D_glszm_GrayLevelVariance | 16.79 | 46.55 | 0.988 | 0.966 | 0.995 | 41.1 | -52 |
| log-sigma-2-0-mm-3D_glszm_HighGrayLevelZoneEmphasis | 15.88 | 44.02 | 0.983 | 0.955 | 0.993 | 35.7 | -52.4 |
| log-sigma-2-0-mm-3D_glszm_LargeAreaEmphasis | 25.24 | 69.96 | 0.904 | 0.793 | 0.957 | 60 | -79.9 |
| log-sigma-2-0-mm-3D_glszm_LargeAreaHighGrayLevelEmphasis | 25.57 | 70.87 | 0.902 | 0.79 | 0.956 | 51.6 | -90.1 |
| log-sigma-2-0-mm-3D_glszm_LargeAreaLowGrayLevelEmphasis | 37.79 | 104.75 | 0.948 | 0.884 | 0.977 | 107.3 | -102.2 |
| log-sigma-2-0-mm-3D_glszm_LowGrayLevelZoneEmphasis | 15.90 | 44.07 | 0.939 | 0.867 | 0.973 | 45.7 | -42.5 |
| log-sigma-2-0-mm-3D_glszm_SizeZoneNonUniformity | 19.52 | 54.11 | 0.99 | 0.978 | 0.996 | 59.6 | -48.7 |
| log-sigma-2-0-mm-3D_glszm_SizeZoneNonUniformityNormalized | 12.00 | 33.27 | 0.898 | 0.74 | 0.958 | 43 | -23.6 |
| log-sigma-2-0-mm-3D_glszm_SmallAreaEmphasis | 8.11 | 22.49 | 0.921 | 0.777 | 0.969 | 28.7 | -16.3 |
| log-sigma-2-0-mm-3D_glszm_SmallAreaHighGrayLevelEmphasis | 20.99 | 58.19 | 0.975 | 0.943 | 0.989 | 57.4 | -59 |
| log-sigma-2-0-mm-3D_glszm_SmallAreaLowGrayLevelEmphasis | 36.44 | 101.00 | 0.304 | -0.1 | 0.624 | 101.6 | -100.4 |
| log-sigma-2-0-mm-3D_glszm_ZoneEntropy | 3.12 | 8.65 | 0.963 | 0.91 | 0.985 | 6.8 | -10.5 |
| log-sigma-2-0-mm-3D_glszm_ZonePercentage | 11.90 | 32.99 | 0.969 | 0.925 | 0.987 | 37.3 | -28.7 |
| log-sigma-2-0-mm-3D_glszm_ZoneVariance | 30.04 | 83.26 | 0.909 | 0.803 | 0.959 | 74.4 | -92.1 |
| log-sigma-2-0-mm-3D_ngtdm_Busyness | 18.97 | 52.57 | 0.917 | 0.821 | 0.963 | 56 | -49.2 |
| log-sigma-2-0-mm-3D_ngtdm_Coarseness | 8.27 | 22.93 | 0.937 | 0.845 | 0.974 | 28.7 | -17.1 |
| log-sigma-2-0-mm-3D_ngtdm_Complexity | 22.24 | 61.65 | 0.99 | 0.977 | 0.996 | 56.1 | -67.2 |
| log-sigma-2-0-mm-3D_ngtdm_Contrast | 10.37 | 28.74 | 0.971 | 0.936 | 0.987 | 31 | -26.5 |
| log-sigma-2-0-mm-3D_ngtdm_Strength | 22.32 | 61.87 | 0.988 | 0.973 | 0.995 | 58.7 | -65 |
| log-sigma-3-0-mm-3D_firstorder_10Percentile | 4.85 | 13.44 | 0.993 | 0.985 | 0.997 | 13.1 | -13.8 |
| log-sigma-3-0-mm-3D_firstorder_90Percentile | 88.75 | 245.99 | 0.977 | 0.933 | 0.991 | 216.3 | -275.7 |
| log-sigma-3-0-mm-3D_firstorder_Energy | 11.78 | 32.65 | 0.994 | 0.986 | 0.998 | 24 | -41.3 |
| log-sigma-3-0-mm-3D_firstorder_Entropy | 2.05 | 5.68 | 0.988 | 0.972 | 0.995 | 5.1 | -6.2 |
| log-sigma-3-0-mm-3D_firstorder_InterquartileRange | 6.18 | 17.14 | 0.99 | 0.978 | 0.996 | 18.5 | -15.8 |
| log-sigma-3-0-mm-3D_firstorder_Kurtosis | 7.53 | 20.88 | 0.955 | 0.901 | 0.98 | 17.2 | -24.5 |
| log-sigma-3-0-mm-3D_firstorder_Maximum | 45.92 | 127.27 | 0.944 | 0.872 | 0.976 | 104.3 | -150.3 |
| log-sigma-3-0-mm-3D_firstorder_MeanAbsoluteDeviation | 6.73 | 18.66 | 0.993 | 0.983 | 0.997 | 16.6 | -20.7 |
| log-sigma-3-0-mm-3D_firstorder_Mean | 9.62 | 26.66 | 0.987 | 0.969 | 0.995 | 32 | -21.4 |
| log-sigma-3-0-mm-3D_firstorder_Median | 12.15 | 33.69 | 0.96 | 0.87 | 0.985 | 40.2 | -27.2 |
| log-sigma-3-0-mm-3D_firstorder_Minimum | 6.18 | 17.14 | 0.993 | 0.972 | 0.997 | 12.9 | -21.4 |
| log-sigma-3-0-mm-3D_firstorder_Range | 7.67 | 21.26 | 0.991 | 0.958 | 0.997 | 16.5 | -26 |
| log-sigma-3-0-mm-3D_firstorder_RobustMeanAbsoluteDeviation | 6.67 | 18.48 | 0.991 | 0.979 | 0.996 | 17.5 | -19.4 |
| log-sigma-3-0-mm-3D_firstorder_RootMeanSquared | 6.08 | 16.86 | 0.994 | 0.985 | 0.997 | 16.8 | -16.9 |
| log-sigma-3-0-mm-3D_firstorder_Skewness | 23.27 | 64.51 | 0.976 | 0.944 | 0.99 | 62.2 | -66.8 |
| log-sigma-3-0-mm-3D_firstorder_TotalEnergy | 11.78 | 32.65 | 0.994 | 0.986 | 0.998 | 24 | -41.3 |
| log-sigma-3-0-mm-3D_firstorder_Uniformity | 5.61 | 15.55 | 0.98 | 0.955 | 0.991 | 16.1 | -15 |
| log-sigma-3-0-mm-3D_firstorder_Variance | 13.72 | 38.02 | 0.991 | 0.977 | 0.996 | 33.1 | -42.9 |
| log-sigma-3-0-mm-3D_glcm_Autocorrelation | 15.70 | 43.52 | 0.986 | 0.954 | 0.995 | 29.5 | -57.5 |
| log-sigma-3-0-mm-3D_glcm_ClusterProminence | 28.85 | 79.95 | 0.975 | 0.935 | 0.99 | 65.8 | -94.1 |
| log-sigma-3-0-mm-3D_glcm_ClusterShade | 35.67 | 98.86 | 0.981 | 0.948 | 0.993 | 75.1 | -122.6 |
| log-sigma-3-0-mm-3D_glcm_ClusterTendency | 14.24 | 39.48 | 0.991 | 0.976 | 0.997 | 33.3 | -45.7 |
| log-sigma-3-0-mm-3D_glcm_Contrast | 13.50 | 37.43 | 0.988 | 0.973 | 0.995 | 34.2 | -40.7 |
| log-sigma-3-0-mm-3D_glcm_Correlation | 1.76 | 4.88 | 0.984 | 0.956 | 0.994 | 3.6 | -6.2 |
| log-sigma-3-0-mm-3D_glcm_DifferenceAverage | 6.43 | 17.81 | 0.992 | 0.981 | 0.996 | 16.8 | -18.8 |
| log-sigma-3-0-mm-3D_glcm_DifferenceEntropy | 3.07 | 8.50 | 0.99 | 0.977 | 0.996 | 7.7 | -9.3 |
| log-sigma-3-0-mm-3D_glcm_DifferenceVariance | 14.91 | 41.32 | 0.989 | 0.971 | 0.995 | 35.9 | -46.8 |
| log-sigma-3-0-mm-3D_glcm_Id | 3.35 | 9.29 | 0.989 | 0.976 | 0.995 | 8.9 | -9.7 |
| log-sigma-3-0-mm-3D_glcm_Idm | 5.00 | 13.86 | 0.987 | 0.971 | 0.994 | 12.9 | -14.8 |
| log-sigma-3-0-mm-3D_glcm_Idmn | 0.10 | 0.29 | 0.985 | 0.886 | 0.996 | 0.1 | -0.4 |
| log-sigma-3-0-mm-3D_glcm_Idn | 0.27 | 0.75 | 0.986 | 0.882 | 0.996 | 0.4 | -1.1 |
| log-sigma-3-0-mm-3D_glcm_Imc1 | 3.39 | 9.40 | 0.988 | 0.972 | 0.995 | 10.8 | -8 |
| log-sigma-3-0-mm-3D_glcm_Imc2 | 0.72 | 2.00 | 0.979 | 0.953 | 0.991 | 2.1 | -1.9 |
| log-sigma-3-0-mm-3D_glcm_InverseVariance | 5.49 | 15.23 | 0.987 | 0.971 | 0.994 | 15.5 | -14.9 |
| log-sigma-3-0-mm-3D_glcm_JointAverage | 8.16 | 22.63 | 0.991 | 0.957 | 0.997 | 15.2 | -30 |
| log-sigma-3-0-mm-3D_glcm_JointEnergy | 8.94 | 24.78 | 0.947 | 0.883 | 0.977 | 28.4 | -21.2 |
| log-sigma-3-0-mm-3D_glcm_JointEntropy | 2.05 | 5.69 | 0.987 | 0.97 | 0.995 | 4.7 | -6.7 |
| log-sigma-3-0-mm-3D_glcm_MCC | 2.18 | 6.06 | 0.938 | 0.863 | 0.972 | 6.5 | -5.6 |
| log-sigma-3-0-mm-3D_glcm_MaximumProbability | 11.05 | 30.64 | 0.968 | 0.929 | 0.986 | 30 | -31.3 |
| log-sigma-3-0-mm-3D_glcm_SumAverage | 8.16 | 22.63 | 0.991 | 0.957 | 0.997 | 15.2 | -30 |
| log-sigma-3-0-mm-3D_glcm_SumEntropy | 1.83 | 5.06 | 0.988 | 0.972 | 0.995 | 4.2 | -5.9 |
| log-sigma-3-0-mm-3D_glcm_SumSquares | 14.07 | 39.00 | 0.991 | 0.976 | 0.996 | 33.3 | -44.7 |
| log-sigma-3-0-mm-3D_gldm_DependenceEntropy | 1.31 | 3.64 | 0.977 | 0.91 | 0.992 | 2.4 | -4.9 |
| log-sigma-3-0-mm-3D_gldm_DependenceNonUniformity | 8.65 | 23.98 | 0.998 | 0.996 | 0.999 | 17.9 | -30.1 |
| log-sigma-3-0-mm-3D_gldm_DependenceNonUniformityNormalized | 7.19 | 19.92 | 0.951 | 0.891 | 0.978 | 22.4 | -17.4 |
| log-sigma-3-0-mm-3D_gldm_DependenceVariance | 17.31 | 47.98 | 0.963 | 0.918 | 0.984 | 49.4 | -46.6 |
| log-sigma-3-0-mm-3D_gldm_GrayLevelNonUniformity | 10.22 | 28.32 | 0.994 | 0.985 | 0.997 | 20.3 | -36.4 |
| log-sigma-3-0-mm-3D_gldm_GrayLevelVariance | 13.79 | 38.23 | 0.991 | 0.977 | 0.996 | 33.3 | -43.1 |
| log-sigma-3-0-mm-3D_gldm_HighGrayLevelEmphasis | 14.89 | 41.28 | 0.986 | 0.954 | 0.995 | 28.5 | -54 |
| log-sigma-3-0-mm-3D_gldm_LargeDependenceEmphasis | 10.13 | 28.09 | 0.984 | 0.964 | 0.993 | 24.8 | -31.4 |
| log-sigma-3-0-mm-3D_gldm_LargeDependenceHighGrayLevelEmphasis | 19.30 | 53.49 | 0.984 | 0.961 | 0.993 | 37.1 | -69.9 |
| log-sigma-3-0-mm-3D_gldm_LargeDependenceLowGrayLevelEmphasis | 57.30 | 158.83 | 0.741 | 0.492 | 0.878 | 169.3 | -148.4 |
| log-sigma-3-0-mm-3D_gldm_LowGrayLevelEmphasis | 28.41 | 78.75 | 0.847 | 0.681 | 0.931 | 93.4 | -64.1 |
| log-sigma-3-0-mm-3D_gldm_SmallDependenceEmphasis | 8.13 | 22.54 | 0.97 | 0.918 | 0.988 | 27.6 | -17.5 |
| log-sigma-3-0-mm-3D_gldm_SmallDependenceHighGrayLevelEmphasis | 18.06 | 50.05 | 0.986 | 0.961 | 0.995 | 42.4 | -57.7 |
| log-sigma-3-0-mm-3D_gldm_SmallDependenceLowGrayLevelEmphasis | 25.00 | 69.29 | 0.793 | 0.57 | 0.906 | 85.6 | -53 |
| log-sigma-3-0-mm-3D_glrlm_GrayLevelNonUniformity | 9.72 | 26.93 | 0.994 | 0.987 | 0.998 | 19.1 | -34.8 |
| log-sigma-3-0-mm-3D_glrlm_GrayLevelNonUniformityNormalized | 5.85 | 16.22 | 0.978 | 0.95 | 0.99 | 16.7 | -15.7 |
| log-sigma-3-0-mm-3D_glrlm_GrayLevelVariance | 13.90 | 38.53 | 0.991 | 0.976 | 0.996 | 33.4 | -43.6 |
| log-sigma-3-0-mm-3D_glrlm_HighGrayLevelRunEmphasis | 14.64 | 40.58 | 0.986 | 0.954 | 0.995 | 27.9 | -53.3 |
| log-sigma-3-0-mm-3D_glrlm_LongRunEmphasis | 2.15 | 5.95 | 0.978 | 0.95 | 0.99 | 5.4 | -6.5 |
| log-sigma-3-0-mm-3D_glrlm_LongRunHighGrayLevelEmphasis | 14.72 | 40.80 | 0.986 | 0.955 | 0.995 | 27.3 | -54.3 |
| log-sigma-3-0-mm-3D_glrlm_LongRunLowGrayLevelEmphasis | 31.48 | 87.26 | 0.817 | 0.627 | 0.916 | 101.8 | -72.7 |
| log-sigma-3-0-mm-3D_glrlm_LowGrayLevelRunEmphasis | 26.91 | 74.59 | 0.866 | 0.716 | 0.94 | 89.1 | -60.1 |
| log-sigma-3-0-mm-3D_glrlm_RunEntropy | 1.53 | 4.25 | 0.987 | 0.97 | 0.995 | 3.5 | -5 |
| log-sigma-3-0-mm-3D_glrlm_RunLengthNonUniformity | 7.27 | 20.16 | 0.997 | 0.993 | 0.999 | 12.2 | -28.1 |
| log-sigma-3-0-mm-3D_glrlm_RunLengthNonUniformityNormalized | 1.21 | 3.35 | 0.974 | 0.941 | 0.989 | 3.7 | -3 |
| log-sigma-3-0-mm-3D_glrlm_RunPercentage | 0.62 | 1.73 | 0.979 | 0.952 | 0.991 | 2 | -1.5 |
| log-sigma-3-0-mm-3D_glrlm_RunVariance | 11.21 | 31.07 | 0.976 | 0.947 | 0.99 | 25.8 | -36.4 |
| log-sigma-3-0-mm-3D_glrlm_ShortRunEmphasis | 0.52 | 1.45 | 0.972 | 0.938 | 0.988 | 1.6 | -1.3 |
| log-sigma-3-0-mm-3D_glrlm_ShortRunHighGrayLevelEmphasis | 14.77 | 40.93 | 0.986 | 0.954 | 0.995 | 28.4 | -53.4 |
| log-sigma-3-0-mm-3D_glrlm_ShortRunLowGrayLevelEmphasis | 25.57 | 70.89 | 0.876 | 0.731 | 0.945 | 85.5 | -56.3 |
| log-sigma-3-0-mm-3D_glszm_GrayLevelNonUniformity | 9.48 | 26.26 | 0.999 | 0.998 | 1 | 24.9 | -27.6 |
| log-sigma-3-0-mm-3D_glszm_GrayLevelNonUniformityNormalized | 6.87 | 19.04 | 0.981 | 0.955 | 0.992 | 22 | -16.1 |
| log-sigma-3-0-mm-3D_glszm_GrayLevelVariance | 13.32 | 36.91 | 0.988 | 0.967 | 0.995 | 29.8 | -44 |
| log-sigma-3-0-mm-3D_glszm_HighGrayLevelZoneEmphasis | 12.05 | 33.40 | 0.987 | 0.951 | 0.995 | 20.6 | -46.3 |
| log-sigma-3-0-mm-3D_glszm_LargeAreaEmphasis | 19.80 | 54.89 | 0.971 | 0.935 | 0.987 | 42.7 | -67.1 |
| log-sigma-3-0-mm-3D_glszm_LargeAreaHighGrayLevelEmphasis | 21.31 | 59.06 | 0.942 | 0.863 | 0.975 | 32.9 | -85.3 |
| log-sigma-3-0-mm-3D_glszm_LargeAreaLowGrayLevelEmphasis | 44.93 | 124.55 | 0.874 | 0.735 | 0.943 | 128.5 | -120.6 |
| log-sigma-3-0-mm-3D_glszm_LowGrayLevelZoneEmphasis | 15.93 | 44.15 | 0.957 | 0.904 | 0.981 | 55.3 | -33 |
| log-sigma-3-0-mm-3D_glszm_SizeZoneNonUniformity | 16.92 | 46.89 | 0.996 | 0.992 | 0.998 | 48.2 | -45.5 |
| log-sigma-3-0-mm-3D_glszm_SizeZoneNonUniformityNormalized | 7.90 | 21.89 | 0.946 | 0.85 | 0.978 | 27.6 | -16.2 |
| log-sigma-3-0-mm-3D_glszm_SmallAreaEmphasis | 5.16 | 14.30 | 0.947 | 0.848 | 0.979 | 17.9 | -10.7 |
| log-sigma-3-0-mm-3D_glszm_SmallAreaHighGrayLevelEmphasis | 12.41 | 34.40 | 0.987 | 0.958 | 0.995 | 24.7 | -44.1 |
| log-sigma-3-0-mm-3D_glszm_SmallAreaLowGrayLevelEmphasis | 40.68 | 112.77 | 0.706 | 0.436 | 0.861 | 125 | -100.5 |
| log-sigma-3-0-mm-3D_glszm_ZoneEntropy | 2.15 | 5.96 | 0.966 | 0.878 | 0.988 | 4 | -7.9 |
| log-sigma-3-0-mm-3D_glszm_ZonePercentage | 9.52 | 26.37 | 0.979 | 0.946 | 0.991 | 30.7 | -22 |
| log-sigma-3-0-mm-3D_glszm_ZoneVariance | 27.96 | 77.51 | 0.975 | 0.943 | 0.989 | 64.1 | -91 |
| log-sigma-3-0-mm-3D_ngtdm_Busyness | 16.67 | 46.20 | 0.931 | 0.848 | 0.969 | 53 | -39.4 |
| log-sigma-3-0-mm-3D_ngtdm_Coarseness | 7.78 | 21.56 | 0.943 | 0.851 | 0.977 | 28 | -15.2 |
| log-sigma-3-0-mm-3D_ngtdm_Complexity | 20.73 | 57.46 | 0.979 | 0.947 | 0.991 | 49.3 | -65.6 |
| log-sigma-3-0-mm-3D_ngtdm_Contrast | 9.38 | 25.99 | 0.972 | 0.936 | 0.988 | 30.9 | -21.1 |
| log-sigma-3-0-mm-3D_ngtdm_Strength | 18.84 | 52.24 | 0.968 | 0.923 | 0.986 | 46.9 | -57.6 |
| log-sigma-4-0-mm-3D_firstorder_10Percentile | 4.21 | 11.66 | 0.995 | 0.988 | 0.998 | 10.2 | -13.1 |
| log-sigma-4-0-mm-3D_firstorder_90Percentile | 118.98 | 329.80 | 0.972 | 0.935 | 0.988 | 365.3 | -294.3 |
| log-sigma-4-0-mm-3D_firstorder_Energy | 12.35 | 34.22 | 0.996 | 0.989 | 0.998 | 24.1 | -44.4 |
| log-sigma-4-0-mm-3D_firstorder_Entropy | 2.11 | 5.84 | 0.986 | 0.967 | 0.994 | 5 | -6.7 |
| log-sigma-4-0-mm-3D_firstorder_InterquartileRange | 5.94 | 16.46 | 0.993 | 0.983 | 0.997 | 15.5 | -17.4 |
| log-sigma-4-0-mm-3D_firstorder_Kurtosis | 5.72 | 15.86 | 0.957 | 0.904 | 0.981 | 13.1 | -18.6 |
| log-sigma-4-0-mm-3D_firstorder_Maximum | 351.72 | 974.91 | 0.953 | 0.893 | 0.979 | 1048.5 | -901.3 |
| log-sigma-4-0-mm-3D_firstorder_MeanAbsoluteDeviation | 6.89 | 19.09 | 0.993 | 0.983 | 0.997 | 16.4 | -21.8 |
| log-sigma-4-0-mm-3D_firstorder_Mean | 43.54 | 120.69 | 0.991 | 0.979 | 0.996 | 134 | -107.4 |
| log-sigma-4-0-mm-3D_firstorder_Median | 13.66 | 37.87 | 0.973 | 0.921 | 0.989 | 46.6 | -29.2 |
| log-sigma-4-0-mm-3D_firstorder_Minimum | 4.81 | 13.34 | 0.995 | 0.976 | 0.998 | 9.7 | -17 |
| log-sigma-4-0-mm-3D_firstorder_Range | 6.64 | 18.40 | 0.993 | 0.962 | 0.998 | 14 | -22.8 |
| log-sigma-4-0-mm-3D_firstorder_RobustMeanAbsoluteDeviation | 6.91 | 19.16 | 0.992 | 0.982 | 0.997 | 17 | -21.4 |
| log-sigma-4-0-mm-3D_firstorder_RootMeanSquared | 6.12 | 16.97 | 0.994 | 0.987 | 0.997 | 16.2 | -17.8 |
| log-sigma-4-0-mm-3D_firstorder_Skewness | 16.53 | 45.82 | 0.982 | 0.956 | 0.992 | 37 | -54.6 |
| log-sigma-4-0-mm-3D_firstorder_TotalEnergy | 12.35 | 34.22 | 0.996 | 0.989 | 0.998 | 24.1 | -44.4 |
| log-sigma-4-0-mm-3D_firstorder_Uniformity | 7.20 | 19.96 | 0.974 | 0.942 | 0.989 | 21.8 | -18.1 |
| log-sigma-4-0-mm-3D_firstorder_Variance | 13.68 | 37.91 | 0.994 | 0.982 | 0.998 | 32.2 | -43.6 |
| log-sigma-4-0-mm-3D_glcm_Autocorrelation | 13.91 | 38.54 | 0.988 | 0.961 | 0.995 | 26.1 | -51 |
| log-sigma-4-0-mm-3D_glcm_ClusterProminence | 28.81 | 79.86 | 0.989 | 0.973 | 0.995 | 65 | -94.7 |
| log-sigma-4-0-mm-3D_glcm_ClusterShade | 47.91 | 132.81 | 0.99 | 0.972 | 0.996 | 101.9 | -163.7 |
| log-sigma-4-0-mm-3D_glcm_ClusterTendency | 14.41 | 39.93 | 0.995 | 0.983 | 0.998 | 33.1 | -46.8 |
| log-sigma-4-0-mm-3D_glcm_Contrast | 12.77 | 35.39 | 0.99 | 0.976 | 0.996 | 31.6 | -39.2 |
| log-sigma-4-0-mm-3D_glcm_Correlation | 1.79 | 4.96 | 0.985 | 0.958 | 0.994 | 3.6 | -6.3 |
| log-sigma-4-0-mm-3D_glcm_DifferenceAverage | 6.19 | 17.17 | 0.993 | 0.984 | 0.997 | 15.4 | -18.9 |
| log-sigma-4-0-mm-3D_glcm_DifferenceEntropy | 2.86 | 7.92 | 0.991 | 0.978 | 0.996 | 7 | -8.8 |
| log-sigma-4-0-mm-3D_glcm_DifferenceVariance | 13.72 | 38.03 | 0.991 | 0.974 | 0.996 | 33.1 | -43 |
| log-sigma-4-0-mm-3D_glcm_Id | 3.93 | 10.90 | 0.987 | 0.971 | 0.994 | 11.8 | -10.1 |
| log-sigma-4-0-mm-3D_glcm_Idm | 6.24 | 17.29 | 0.985 | 0.965 | 0.993 | 18.5 | -16 |
| log-sigma-4-0-mm-3D_glcm_Idmn | 0.10 | 0.29 | 0.988 | 0.947 | 0.996 | 0.2 | -0.4 |
| log-sigma-4-0-mm-3D_glcm_Idn | 0.25 | 0.69 | 0.991 | 0.963 | 0.997 | 0.4 | -0.9 |
| log-sigma-4-0-mm-3D_glcm_Imc1 | 4.04 | 11.20 | 0.985 | 0.966 | 0.993 | 12.8 | -9.6 |
| log-sigma-4-0-mm-3D_glcm_Imc2 | 0.91 | 2.53 | 0.949 | 0.887 | 0.977 | 2.8 | -2.3 |
| log-sigma-4-0-mm-3D_glcm_InverseVariance | 5.32 | 14.76 | 0.985 | 0.967 | 0.994 | 15.9 | -13.6 |
| log-sigma-4-0-mm-3D_glcm_JointAverage | 7.08 | 19.62 | 0.994 | 0.963 | 0.998 | 13.1 | -26.1 |
| log-sigma-4-0-mm-3D_glcm_JointEnergy | 10.69 | 29.62 | 0.953 | 0.894 | 0.98 | 36.1 | -23.2 |
| log-sigma-4-0-mm-3D_glcm_JointEntropy | 1.98 | 5.48 | 0.986 | 0.963 | 0.994 | 4.2 | -6.7 |
| log-sigma-4-0-mm-3D_glcm_MCC | 1.39 | 3.87 | 0.981 | 0.957 | 0.992 | 3.9 | -3.8 |
| log-sigma-4-0-mm-3D_glcm_MaximumProbability | 12.97 | 35.95 | 0.947 | 0.877 | 0.977 | 44.7 | -27.2 |
| log-sigma-4-0-mm-3D_glcm_SumAverage | 7.08 | 19.62 | 0.994 | 0.963 | 0.998 | 13.1 | -26.1 |
| log-sigma-4-0-mm-3D_glcm_SumEntropy | 1.73 | 4.78 | 0.988 | 0.97 | 0.995 | 3.8 | -5.8 |
| log-sigma-4-0-mm-3D_glcm_SumSquares | 14.15 | 39.22 | 0.994 | 0.982 | 0.998 | 32.9 | -45.6 |
| log-sigma-4-0-mm-3D_gldm_DependenceEntropy | 1.08 | 3.01 | 0.983 | 0.907 | 0.995 | 1.7 | -4.3 |
| log-sigma-4-0-mm-3D_gldm_DependenceNonUniformity | 11.15 | 30.90 | 0.997 | 0.992 | 0.999 | 21.7 | -40.1 |
| log-sigma-4-0-mm-3D_gldm_DependenceNonUniformityNormalized | 8.36 | 23.18 | 0.932 | 0.852 | 0.97 | 22.6 | -23.8 |
| log-sigma-4-0-mm-3D_gldm_DependenceVariance | 22.56 | 62.53 | 0.958 | 0.9 | 0.982 | 71 | -54 |
| log-sigma-4-0-mm-3D_gldm_GrayLevelNonUniformity | 10.05 | 27.86 | 0.993 | 0.984 | 0.997 | 21.1 | -34.6 |
| log-sigma-4-0-mm-3D_gldm_GrayLevelVariance | 13.91 | 38.56 | 0.994 | 0.982 | 0.998 | 33 | -44.1 |
| log-sigma-4-0-mm-3D_gldm_HighGrayLevelEmphasis | 13.21 | 36.62 | 0.988 | 0.961 | 0.996 | 25.4 | -47.9 |
| log-sigma-4-0-mm-3D_gldm_LargeDependenceEmphasis | 14.41 | 39.94 | 0.973 | 0.939 | 0.988 | 41.8 | -38 |
| log-sigma-4-0-mm-3D_gldm_LargeDependenceHighGrayLevelEmphasis | 16.78 | 46.52 | 0.989 | 0.975 | 0.995 | 36.2 | -56.8 |
| log-sigma-4-0-mm-3D_gldm_LargeDependenceLowGrayLevelEmphasis | 48.19 | 133.58 | 0.837 | 0.663 | 0.926 | 142.2 | -125 |
| log-sigma-4-0-mm-3D_gldm_LowGrayLevelEmphasis | 20.79 | 57.63 | 0.887 | 0.76 | 0.949 | 68.2 | -47 |
| log-sigma-4-0-mm-3D_gldm_SmallDependenceEmphasis | 8.73 | 24.19 | 0.961 | 0.914 | 0.983 | 26.1 | -22.3 |
| log-sigma-4-0-mm-3D_gldm_SmallDependenceHighGrayLevelEmphasis | 20.38 | 56.50 | 0.985 | 0.949 | 0.994 | 47.5 | -65.5 |
| log-sigma-4-0-mm-3D_gldm_SmallDependenceLowGrayLevelEmphasis | 20.56 | 56.99 | 0.807 | 0.59 | 0.913 | 72.7 | -41.3 |
| log-sigma-4-0-mm-3D_glrlm_GrayLevelNonUniformity | 9.49 | 26.31 | 0.994 | 0.987 | 0.998 | 19.4 | -33.3 |
| log-sigma-4-0-mm-3D_glrlm_GrayLevelNonUniformityNormalized | 7.04 | 19.51 | 0.976 | 0.946 | 0.989 | 21.1 | -17.9 |
| log-sigma-4-0-mm-3D_glrlm_GrayLevelVariance | 13.94 | 38.64 | 0.994 | 0.982 | 0.998 | 33.1 | -44.1 |
| log-sigma-4-0-mm-3D_glrlm_HighGrayLevelRunEmphasis | 12.95 | 35.90 | 0.988 | 0.96 | 0.996 | 24.7 | -47.1 |
| log-sigma-4-0-mm-3D_glrlm_LongRunEmphasis | 2.07 | 5.73 | 0.972 | 0.937 | 0.988 | 5.8 | -5.6 |
| log-sigma-4-0-mm-3D_glrlm_LongRunHighGrayLevelEmphasis | 12.43 | 34.46 | 0.989 | 0.966 | 0.996 | 23.2 | -45.7 |
| log-sigma-4-0-mm-3D_glrlm_LongRunLowGrayLevelEmphasis | 23.86 | 66.13 | 0.882 | 0.75 | 0.947 | 76.7 | -55.5 |
| log-sigma-4-0-mm-3D_glrlm_LowGrayLevelRunEmphasis | 19.42 | 53.84 | 0.902 | 0.789 | 0.956 | 64.5 | -43.2 |
| log-sigma-4-0-mm-3D_glrlm_RunEntropy | 1.51 | 4.18 | 0.988 | 0.971 | 0.995 | 3.3 | -5 |
| log-sigma-4-0-mm-3D_glrlm_RunLengthNonUniformity | 7.76 | 21.51 | 0.997 | 0.992 | 0.999 | 12.9 | -30.1 |
| log-sigma-4-0-mm-3D_glrlm_RunLengthNonUniformityNormalized | 1.23 | 3.40 | 0.964 | 0.92 | 0.984 | 3.4 | -3.4 |
| log-sigma-4-0-mm-3D_glrlm_RunPercentage | 0.66 | 1.82 | 0.97 | 0.933 | 0.987 | 1.8 | -1.8 |
| log-sigma-4-0-mm-3D_glrlm_RunVariance | 14.75 | 40.88 | 0.973 | 0.94 | 0.988 | 42.2 | -39.6 |
| log-sigma-4-0-mm-3D_glrlm_ShortRunEmphasis | 0.51 | 1.40 | 0.966 | 0.924 | 0.985 | 1.4 | -1.4 |
| log-sigma-4-0-mm-3D_glrlm_ShortRunHighGrayLevelEmphasis | 13.12 | 36.37 | 0.988 | 0.959 | 0.995 | 25.3 | -47.5 |
| log-sigma-4-0-mm-3D_glrlm_ShortRunLowGrayLevelEmphasis | 18.03 | 49.98 | 0.913 | 0.812 | 0.961 | 60.7 | -39.3 |
| log-sigma-4-0-mm-3D_glszm_GrayLevelNonUniformity | 7.60 | 21.06 | 0.998 | 0.996 | 0.999 | 15.5 | -26.6 |
| log-sigma-4-0-mm-3D_glszm_GrayLevelNonUniformityNormalized | 7.92 | 21.94 | 0.972 | 0.937 | 0.988 | 23.5 | -20.4 |
| log-sigma-4-0-mm-3D_glszm_GrayLevelVariance | 15.23 | 42.21 | 0.994 | 0.98 | 0.998 | 37.6 | -46.8 |
| log-sigma-4-0-mm-3D_glszm_HighGrayLevelZoneEmphasis | 13.14 | 36.41 | 0.987 | 0.954 | 0.995 | 26.1 | -46.7 |
| log-sigma-4-0-mm-3D_glszm_LargeAreaEmphasis | 25.11 | 69.61 | 0.909 | 0.803 | 0.959 | 68 | -71.3 |
| log-sigma-4-0-mm-3D_glszm_LargeAreaHighGrayLevelEmphasis | 19.42 | 53.82 | 0.993 | 0.984 | 0.997 | 39.9 | -67.7 |
| log-sigma-4-0-mm-3D_glszm_LargeAreaLowGrayLevelEmphasis | 41.57 | 115.23 | 0.902 | 0.79 | 0.956 | 122.4 | -108.1 |
| log-sigma-4-0-mm-3D_glszm_LowGrayLevelZoneEmphasis | 12.41 | 34.40 | 0.937 | 0.863 | 0.972 | 45.3 | -23.4 |
| log-sigma-4-0-mm-3D_glszm_SizeZoneNonUniformity | 17.37 | 48.16 | 0.996 | 0.991 | 0.998 | 43.4 | -52.9 |
| log-sigma-4-0-mm-3D_glszm_SizeZoneNonUniformityNormalized | 7.32 | 20.29 | 0.959 | 0.908 | 0.982 | 22.6 | -18 |
| log-sigma-4-0-mm-3D_glszm_SmallAreaEmphasis | 4.64 | 12.87 | 0.949 | 0.886 | 0.977 | 14.1 | -11.6 |
| log-sigma-4-0-mm-3D_glszm_SmallAreaHighGrayLevelEmphasis | 17.95 | 49.76 | 0.987 | 0.954 | 0.995 | 40.5 | -59 |
| log-sigma-4-0-mm-3D_glszm_SmallAreaLowGrayLevelEmphasis | 32.09 | 88.95 | 0.736 | 0.486 | 0.876 | 105.6 | -72.3 |
| log-sigma-4-0-mm-3D_glszm_ZoneEntropy | 1.42 | 3.92 | 0.982 | 0.917 | 0.994 | 2.4 | -5.4 |
| log-sigma-4-0-mm-3D_glszm_ZonePercentage | 8.65 | 23.97 | 0.965 | 0.921 | 0.985 | 25.4 | -22.5 |
| log-sigma-4-0-mm-3D_glszm_ZoneVariance | 38.24 | 106.00 | 0.902 | 0.79 | 0.956 | 106.8 | -105.2 |
| log-sigma-4-0-mm-3D_ngtdm_Busyness | 15.88 | 44.03 | 0.963 | 0.918 | 0.984 | 49.2 | -38.8 |
| log-sigma-4-0-mm-3D_ngtdm_Coarseness | 7.14 | 19.78 | 0.944 | 0.852 | 0.977 | 26.4 | -13.1 |
| log-sigma-4-0-mm-3D_ngtdm_Complexity | 19.72 | 54.66 | 0.992 | 0.974 | 0.997 | 46.9 | -62.4 |
| log-sigma-4-0-mm-3D_ngtdm_Contrast | 8.50 | 23.56 | 0.971 | 0.935 | 0.987 | 26.2 | -20.9 |
| log-sigma-4-0-mm-3D_ngtdm_Strength | 18.61 | 51.57 | 0.989 | 0.97 | 0.995 | 48.5 | -54.6 |
| log-sigma-5-0-mm-3D_firstorder_10Percentile | 4.03 | 11.18 | 0.995 | 0.989 | 0.998 | 9.5 | -12.9 |
| log-sigma-5-0-mm-3D_firstorder_90Percentile | 24.61 | 68.22 | 0.97 | 0.932 | 0.987 | 71.1 | -65.3 |
| log-sigma-5-0-mm-3D_firstorder_Energy | 12.91 | 35.80 | 0.996 | 0.991 | 0.999 | 24 | -47.6 |
| log-sigma-5-0-mm-3D_firstorder_Entropy | 1.96 | 5.42 | 0.988 | 0.971 | 0.995 | 4.4 | -6.4 |
| log-sigma-5-0-mm-3D_firstorder_InterquartileRange | 7.63 | 21.14 | 0.991 | 0.979 | 0.996 | 19.3 | -23 |
| log-sigma-5-0-mm-3D_firstorder_Kurtosis | 4.71 | 13.05 | 0.96 | 0.91 | 0.982 | 11 | -15.1 |
| log-sigma-5-0-mm-3D_firstorder_Maximum | 74.92 | 207.67 | 0.965 | 0.921 | 0.984 | 231.9 | -183.5 |
| log-sigma-5-0-mm-3D_firstorder_MeanAbsoluteDeviation | 6.89 | 19.11 | 0.994 | 0.982 | 0.998 | 15.8 | -22.4 |
| log-sigma-5-0-mm-3D_firstorder_Mean | 31.37 | 86.96 | 0.992 | 0.982 | 0.997 | 76.4 | -97.5 |
| log-sigma-5-0-mm-3D_firstorder_Median | 15.23 | 42.21 | 0.985 | 0.965 | 0.993 | 47.4 | -37 |
| log-sigma-5-0-mm-3D_firstorder_Minimum | 4.39 | 12.18 | 0.996 | 0.98 | 0.999 | 8.2 | -16.2 |
| log-sigma-5-0-mm-3D_firstorder_Range | 6.35 | 17.60 | 0.994 | 0.975 | 0.998 | 13.5 | -21.7 |
| log-sigma-5-0-mm-3D_firstorder_RobustMeanAbsoluteDeviation | 7.10 | 19.69 | 0.993 | 0.982 | 0.997 | 16.8 | -22.6 |
| log-sigma-5-0-mm-3D_firstorder_RootMeanSquared | 6.14 | 17.02 | 0.995 | 0.987 | 0.998 | 15.3 | -18.7 |
| log-sigma-5-0-mm-3D_firstorder_Skewness | 18.25 | 50.60 | 0.985 | 0.962 | 0.993 | 39.2 | -62 |
| log-sigma-5-0-mm-3D_firstorder_TotalEnergy | 12.91 | 35.80 | 0.996 | 0.991 | 0.999 | 24 | -47.6 |
| log-sigma-5-0-mm-3D_firstorder_Uniformity | 6.77 | 18.77 | 0.982 | 0.959 | 0.992 | 21.6 | -16 |
| log-sigma-5-0-mm-3D_firstorder_Variance | 13.47 | 37.33 | 0.997 | 0.987 | 0.999 | 30.5 | -44.2 |
| log-sigma-5-0-mm-3D_glcm_Autocorrelation | 13.46 | 37.30 | 0.992 | 0.974 | 0.997 | 24.9 | -49.7 |
| log-sigma-5-0-mm-3D_glcm_ClusterProminence | 28.04 | 77.71 | 0.994 | 0.985 | 0.997 | 60.1 | -95.3 |
| log-sigma-5-0-mm-3D_glcm_ClusterShade | 62.45 | 173.10 | 0.993 | 0.984 | 0.997 | 133.2 | -213 |
| log-sigma-5-0-mm-3D_glcm_ClusterTendency | 14.07 | 39.01 | 0.997 | 0.987 | 0.999 | 30.6 | -47.5 |
| log-sigma-5-0-mm-3D_glcm_Contrast | 11.76 | 32.60 | 0.992 | 0.98 | 0.997 | 27.5 | -37.7 |
| log-sigma-5-0-mm-3D_glcm_Correlation | 1.89 | 5.23 | 0.984 | 0.954 | 0.994 | 3.7 | -6.7 |
| log-sigma-5-0-mm-3D_glcm_DifferenceAverage | 5.73 | 15.89 | 0.994 | 0.984 | 0.997 | 13.3 | -18.5 |
| log-sigma-5-0-mm-3D_glcm_DifferenceEntropy | 2.76 | 7.66 | 0.991 | 0.979 | 0.996 | 6.5 | -8.8 |
| log-sigma-5-0-mm-3D_glcm_DifferenceVariance | 12.59 | 34.89 | 0.994 | 0.979 | 0.998 | 29.5 | -40.3 |
| log-sigma-5-0-mm-3D_glcm_Id | 3.40 | 9.42 | 0.989 | 0.975 | 0.995 | 11.1 | -7.8 |
| log-sigma-5-0-mm-3D_glcm_Idm | 5.13 | 14.21 | 0.987 | 0.971 | 0.994 | 16.5 | -11.9 |
| log-sigma-5-0-mm-3D_glcm_Idmn | 0.08 | 0.23 | 0.993 | 0.98 | 0.997 | 0.2 | -0.3 |
| log-sigma-5-0-mm-3D_glcm_Idn | 0.21 | 0.59 | 0.995 | 0.986 | 0.998 | 0.4 | -0.7 |
| log-sigma-5-0-mm-3D_glcm_Imc1 | 3.56 | 9.87 | 0.985 | 0.967 | 0.994 | 11.1 | -8.7 |
| log-sigma-5-0-mm-3D_glcm_Imc2 | 0.72 | 1.99 | 0.97 | 0.934 | 0.987 | 2.2 | -1.8 |
| log-sigma-5-0-mm-3D_glcm_InverseVariance | 4.42 | 12.25 | 0.989 | 0.975 | 0.995 | 13 | -11.5 |
| log-sigma-5-0-mm-3D_glcm_JointAverage | 6.76 | 18.75 | 0.995 | 0.967 | 0.999 | 12.3 | -25.2 |
| log-sigma-5-0-mm-3D_glcm_JointEnergy | 10.76 | 29.83 | 0.964 | 0.91 | 0.985 | 38.1 | -21.5 |
| log-sigma-5-0-mm-3D_glcm_JointEntropy | 1.94 | 5.39 | 0.987 | 0.961 | 0.995 | 4 | -6.8 |
| log-sigma-5-0-mm-3D_glcm_MCC | 1.48 | 4.10 | 0.981 | 0.957 | 0.992 | 3.5 | -4.7 |
| log-sigma-5-0-mm-3D_glcm_MaximumProbability | 14.20 | 39.37 | 0.907 | 0.79 | 0.959 | 47.8 | -31 |
| log-sigma-5-0-mm-3D_glcm_SumAverage | 6.76 | 18.75 | 0.995 | 0.967 | 0.999 | 12.3 | -25.2 |
| log-sigma-5-0-mm-3D_glcm_SumEntropy | 1.69 | 4.68 | 0.989 | 0.968 | 0.995 | 3.5 | -5.9 |
| log-sigma-5-0-mm-3D_glcm_SumSquares | 13.74 | 38.09 | 0.997 | 0.987 | 0.999 | 30.2 | -46 |
| log-sigma-5-0-mm-3D_gldm_DependenceEntropy | 1.39 | 3.84 | 0.984 | 0.962 | 0.993 | 3.1 | -4.6 |
| log-sigma-5-0-mm-3D_gldm_DependenceNonUniformity | 11.28 | 31.27 | 0.996 | 0.991 | 0.998 | 20.6 | -41.9 |
| log-sigma-5-0-mm-3D_gldm_DependenceNonUniformityNormalized | 7.59 | 21.04 | 0.949 | 0.887 | 0.977 | 19 | -23.1 |
| log-sigma-5-0-mm-3D_gldm_DependenceVariance | 19.72 | 54.66 | 0.921 | 0.819 | 0.965 | 64.3 | -45 |
| log-sigma-5-0-mm-3D_gldm_GrayLevelNonUniformity | 8.68 | 24.05 | 0.994 | 0.986 | 0.997 | 18.3 | -29.8 |
| log-sigma-5-0-mm-3D_gldm_GrayLevelVariance | 13.37 | 37.05 | 0.997 | 0.987 | 0.999 | 30 | -44.1 |
| log-sigma-5-0-mm-3D_gldm_HighGrayLevelEmphasis | 12.74 | 35.31 | 0.993 | 0.974 | 0.997 | 24 | -46.6 |
| log-sigma-5-0-mm-3D_gldm_LargeDependenceEmphasis | 12.97 | 35.96 | 0.965 | 0.923 | 0.985 | 40.6 | -31.3 |
| log-sigma-5-0-mm-3D_gldm_LargeDependenceHighGrayLevelEmphasis | 15.93 | 44.14 | 0.995 | 0.986 | 0.998 | 35.3 | -53 |
| log-sigma-5-0-mm-3D_gldm_LargeDependenceLowGrayLevelEmphasis | 54.17 | 150.15 | 0.951 | 0.892 | 0.979 | 172 | -128.3 |
| log-sigma-5-0-mm-3D_gldm_LowGrayLevelEmphasis | 21.51 | 59.61 | 0.95 | 0.89 | 0.978 | 73.5 | -45.7 |
| log-sigma-5-0-mm-3D_gldm_SmallDependenceEmphasis | 9.82 | 27.21 | 0.966 | 0.924 | 0.985 | 27 | -27.4 |
| log-sigma-5-0-mm-3D_gldm_SmallDependenceHighGrayLevelEmphasis | 19.80 | 54.88 | 0.992 | 0.969 | 0.997 | 45.4 | -64.4 |
| log-sigma-5-0-mm-3D_gldm_SmallDependenceLowGrayLevelEmphasis | 23.15 | 64.17 | 0.898 | 0.781 | 0.954 | 66 | -62.3 |
| log-sigma-5-0-mm-3D_glrlm_GrayLevelNonUniformity | 8.39 | 23.24 | 0.995 | 0.989 | 0.998 | 17 | -29.5 |
| log-sigma-5-0-mm-3D_glrlm_GrayLevelNonUniformityNormalized | 6.63 | 18.36 | 0.983 | 0.962 | 0.993 | 20.9 | -15.8 |
| log-sigma-5-0-mm-3D_glrlm_GrayLevelVariance | 13.40 | 37.15 | 0.997 | 0.987 | 0.999 | 30.2 | -44.1 |
| log-sigma-5-0-mm-3D_glrlm_HighGrayLevelRunEmphasis | 12.47 | 34.57 | 0.992 | 0.973 | 0.997 | 23.4 | -45.8 |
| log-sigma-5-0-mm-3D_glrlm_LongRunEmphasis | 1.94 | 5.37 | 0.974 | 0.941 | 0.988 | 5.8 | -4.9 |
| log-sigma-5-0-mm-3D_glrlm_LongRunHighGrayLevelEmphasis | 12.36 | 34.27 | 0.993 | 0.975 | 0.997 | 23.1 | -45.4 |
| log-sigma-5-0-mm-3D_glrlm_LongRunLowGrayLevelEmphasis | 24.74 | 68.57 | 0.955 | 0.9 | 0.98 | 83.1 | -54 |
| log-sigma-5-0-mm-3D_glrlm_LowGrayLevelRunEmphasis | 20.03 | 55.51 | 0.954 | 0.898 | 0.98 | 69.3 | -41.7 |
| log-sigma-5-0-mm-3D_glrlm_RunEntropy | 1.44 | 3.98 | 0.99 | 0.974 | 0.996 | 3.1 | -4.8 |
| log-sigma-5-0-mm-3D_glrlm_RunLengthNonUniformity | 7.91 | 21.94 | 0.996 | 0.992 | 0.998 | 12.8 | -31.1 |
| log-sigma-5-0-mm-3D_glrlm_RunLengthNonUniformityNormalized | 1.20 | 3.31 | 0.965 | 0.923 | 0.985 | 2.9 | -3.7 |
| log-sigma-5-0-mm-3D_glrlm_RunPercentage | 0.64 | 1.77 | 0.971 | 0.935 | 0.987 | 1.6 | -1.9 |
| log-sigma-5-0-mm-3D_glrlm_RunVariance | 12.32 | 34.16 | 0.975 | 0.944 | 0.989 | 37.1 | -31.3 |
| log-sigma-5-0-mm-3D_glrlm_ShortRunEmphasis | 0.50 | 1.38 | 0.966 | 0.923 | 0.985 | 1.2 | -1.5 |
| log-sigma-5-0-mm-3D_glrlm_ShortRunHighGrayLevelEmphasis | 12.60 | 34.94 | 0.992 | 0.973 | 0.997 | 23.7 | -46.2 |
| log-sigma-5-0-mm-3D_glrlm_ShortRunLowGrayLevelEmphasis | 18.59 | 51.53 | 0.957 | 0.902 | 0.981 | 65.1 | -38 |
| log-sigma-5-0-mm-3D_glszm_GrayLevelNonUniformity | 6.13 | 16.99 | 0.996 | 0.991 | 0.998 | 11.5 | -22.5 |
| log-sigma-5-0-mm-3D_glszm_GrayLevelNonUniformityNormalized | 7.33 | 20.32 | 0.975 | 0.944 | 0.989 | 23.3 | -17.3 |
| log-sigma-5-0-mm-3D_glszm_GrayLevelVariance | 14.32 | 39.70 | 0.994 | 0.981 | 0.998 | 32.6 | -46.8 |
| log-sigma-5-0-mm-3D_glszm_HighGrayLevelZoneEmphasis | 11.90 | 32.97 | 0.992 | 0.971 | 0.997 | 23.6 | -42.3 |
| log-sigma-5-0-mm-3D_glszm_LargeAreaEmphasis | 23.89 | 66.21 | 0.857 | 0.702 | 0.935 | 69.2 | -63.2 |
| log-sigma-5-0-mm-3D_glszm_LargeAreaHighGrayLevelEmphasis | 20.19 | 55.97 | 0.992 | 0.981 | 0.996 | 45 | -66.9 |
| log-sigma-5-0-mm-3D_glszm_LargeAreaLowGrayLevelEmphasis | 42.77 | 118.56 | 0.974 | 0.943 | 0.989 | 139.2 | -97.9 |
| log-sigma-5-0-mm-3D_glszm_LowGrayLevelZoneEmphasis | 11.90 | 32.99 | 0.965 | 0.921 | 0.985 | 40.2 | -25.8 |
| log-sigma-5-0-mm-3D_glszm_SizeZoneNonUniformity | 18.88 | 52.34 | 0.994 | 0.987 | 0.998 | 45.4 | -59.3 |
| log-sigma-5-0-mm-3D_glszm_SizeZoneNonUniformityNormalized | 10.10 | 27.99 | 0.952 | 0.894 | 0.979 | 29.4 | -26.6 |
| log-sigma-5-0-mm-3D_glszm_SmallAreaEmphasis | 6.90 | 19.11 | 0.913 | 0.813 | 0.961 | 20.2 | -18.1 |
| log-sigma-5-0-mm-3D_glszm_SmallAreaHighGrayLevelEmphasis | 17.38 | 48.16 | 0.991 | 0.968 | 0.997 | 40.6 | -55.7 |
| log-sigma-5-0-mm-3D_glszm_SmallAreaLowGrayLevelEmphasis | 34.86 | 96.64 | 0.941 | 0.871 | 0.974 | 93.8 | -99.5 |
| log-sigma-5-0-mm-3D_glszm_ZoneEntropy | 2.07 | 5.74 | 0.98 | 0.952 | 0.991 | 4.8 | -6.7 |
| log-sigma-5-0-mm-3D_glszm_ZonePercentage | 9.89 | 27.43 | 0.971 | 0.935 | 0.987 | 27.5 | -27.3 |
| log-sigma-5-0-mm-3D_glszm_ZoneVariance | 32.62 | 90.43 | 0.861 | 0.709 | 0.937 | 96.4 | -84.4 |
| log-sigma-5-0-mm-3D_ngtdm_Busyness | 16.52 | 45.80 | 0.936 | 0.861 | 0.972 | 51.1 | -40.5 |
| log-sigma-5-0-mm-3D_ngtdm_Coarseness | 7.46 | 20.67 | 0.933 | 0.81 | 0.973 | 28.1 | -13.3 |
| log-sigma-5-0-mm-3D_ngtdm_Complexity | 17.26 | 47.83 | 0.992 | 0.978 | 0.997 | 37.3 | -58.4 |
| log-sigma-5-0-mm-3D_ngtdm_Contrast | 7.25 | 20.11 | 0.988 | 0.973 | 0.995 | 21.3 | -18.9 |
| log-sigma-5-0-mm-3D_ngtdm_Strength | 17.09 | 47.36 | 0.989 | 0.971 | 0.996 | 43.2 | -51.5 |
| logarithm_firstorder_10Percentile | 10.23 | 28.36 | 0.512 | -0.054 | 0.799 | 8.6 | -48.2 |
| logarithm_firstorder_90Percentile | 11.40 | 31.61 | 0.621 | 0.038 | 0.853 | 11 | -52.2 |
| logarithm_firstorder_Energy | 21.03 | 58.29 | 0.61 | 0.293 | 0.809 | 11.5 | -105.1 |
| logarithm_firstorder_Entropy | 2.48 | 6.88 | 0.911 | 0.342 | 0.975 | 2.8 | -11 |
| logarithm_firstorder_InterquartileRange | 13.02 | 36.09 | 0.746 | 0.221 | 0.906 | 14.2 | -58 |
| logarithm_firstorder_Kurtosis | 2.99 | 8.28 | 0.877 | 0.74 | 0.945 | 7.9 | -8.7 |
| logarithm_firstorder_Maximum | 11.10 | 30.77 | 0.642 | 0.108 | 0.857 | 9.6 | -51.9 |
| logarithm_firstorder_MeanAbsoluteDeviation | 12.89 | 35.72 | 0.73 | 0.19 | 0.9 | 13.2 | -58.3 |
| logarithm_firstorder_Mean | 10.96 | 30.38 | 0.575 | 0.006 | 0.828 | 10.5 | -50.3 |
| logarithm_firstorder_Median | 10.98 | 30.44 | 0.57 | 0.015 | 0.823 | 10.9 | -50 |
| logarithm_firstorder_Minimum | 10.65 | 29.51 | 0.528 | -0.071 | 0.814 | 12.1 | -47 |
| logarithm_firstorder_Range | 11.85 | 32.85 | 0.703 | 0.229 | 0.882 | 9.7 | -56 |
| logarithm_firstorder_RobustMeanAbsoluteDeviation | 13.40 | 37.14 | 0.736 | 0.185 | 0.904 | 14.6 | -59.7 |
| logarithm_firstorder_RootMeanSquared | 11.03 | 30.58 | 0.584 | 0.014 | 0.833 | 10.6 | -50.6 |
| logarithm_firstorder_Skewness | 349.67 | 969.24 | 0.848 | 0.647 | 0.934 | 941.1 | -997.4 |
| logarithm_firstorder_TotalEnergy | 21.03 | 58.29 | 0.61 | 0.293 | 0.809 | 11.5 | -105.1 |
| logarithm_firstorder_Uniformity | 12.54 | 34.76 | 0.957 | 0.799 | 0.986 | 55.1 | -14.4 |
| logarithm_firstorder_Variance | 23.18 | 64.25 | 0.469 | 0.081 | 0.732 | 21 | -107.5 |
| logarithm_glcm_Autocorrelation | 22.83 | 63.27 | 0.41 | 0.038 | 0.689 | 21.2 | -105.3 |
| logarithm_glcm_ClusterProminence | 37.95 | 105.18 | 0.108 | -0.271 | 0.471 | 25.4 | -185 |
| logarithm_glcm_ClusterShade | 2549.64 | 7067.23 | 0.307 | -0.07 | 0.618 | 6363.7 | -7770.8 |
| logarithm_glcm_ClusterTendency | 23.20 | 64.30 | 0.481 | 0.096 | 0.739 | 19.9 | -108.7 |
| logarithm_glcm_Contrast | 23.51 | 65.17 | 0.495 | 0.051 | 0.761 | 24.6 | -105.7 |
| logarithm_glcm_Correlation | 2.64 | 7.32 | 0.963 | 0.906 | 0.985 | 5.6 | -9.1 |
| logarithm_glcm_DifferenceAverage | 12.73 | 35.29 | 0.737 | 0.124 | 0.909 | 14.3 | -56.3 |
| logarithm_glcm_DifferenceEntropy | 2.94 | 8.16 | 0.9 | 0.308 | 0.971 | 3.4 | -12.9 |
| logarithm_glcm_DifferenceVariance | 23.32 | 64.63 | 0.458 | 0.05 | 0.729 | 24.1 | -105.1 |
| logarithm_glcm_Id | 9.99 | 27.69 | 0.957 | 0.776 | 0.986 | 42.4 | -13 |
| logarithm_glcm_Idm | 14.85 | 41.17 | 0.967 | 0.904 | 0.987 | 57.8 | -24.5 |
| logarithm_glcm_Idmn | 0.16 | 0.44 | 0.962 | 0.902 | 0.985 | 0.3 | -0.5 |
| logarithm_glcm_Idn | 0.32 | 0.90 | 0.972 | 0.925 | 0.989 | 0.7 | -1.1 |
| logarithm_glcm_Imc1 | 6.54 | 18.13 | 0.956 | 0.814 | 0.985 | 12.3 | -24 |
| logarithm_glcm_Imc2 | 0.40 | 1.11 | 0.903 | 0.792 | 0.957 | 1 | -1.2 |
| logarithm_glcm_InverseVariance | 13.72 | 38.04 | 0.969 | 0.911 | 0.988 | 53.8 | -22.3 |
| logarithm_glcm_JointAverage | 12.41 | 34.40 | 0.69 | 0.17 | 0.879 | 12.8 | -56 |
| logarithm_glcm_JointEnergy | 10.38 | 28.77 | 0.923 | 0.783 | 0.97 | 42.7 | -14.9 |
| logarithm_glcm_JointEntropy | 1.59 | 4.41 | 0.983 | 0.88 | 0.995 | 2.4 | -6.4 |
| logarithm_glcm_MCC | 3.69 | 10.23 | 0.84 | 0.632 | 0.931 | 7.8 | -12.7 |
| logarithm_glcm_MaximumProbability | 11.64 | 32.25 | 0.938 | 0.732 | 0.979 | 48.5 | -16 |
| logarithm_glcm_SumAverage | 12.41 | 34.40 | 0.69 | 0.17 | 0.879 | 12.8 | -56 |
| logarithm_glcm_SumEntropy | 2.15 | 5.97 | 0.932 | 0.488 | 0.98 | 2.5 | -9.4 |
| logarithm_glcm_SumSquares | 23.18 | 64.25 | 0.475 | 0.084 | 0.736 | 20.4 | -108 |
| logarithm_gldm_DependenceEntropy | 1.53 | 4.25 | 0.923 | 0.2 | 0.981 | 1.1 | -7.4 |
| logarithm_gldm_DependenceNonUniformity | 11.85 | 32.83 | 0.981 | 0.954 | 0.992 | 19 | -46.6 |
| logarithm_gldm_DependenceNonUniformityNormalized | 7.72 | 21.39 | 0.868 | 0.685 | 0.944 | 16.1 | -26.7 |
| logarithm_gldm_DependenceVariance | 26.70 | 74.01 | 0.887 | 0.759 | 0.949 | 88.2 | -59.8 |
| logarithm_gldm_GrayLevelNonUniformity | 13.01 | 36.07 | 0.94 | 0.863 | 0.974 | 47.9 | -24.2 |
| logarithm_gldm_GrayLevelVariance | 23.16 | 64.20 | 0.469 | 0.08 | 0.732 | 20.9 | -107.5 |
| logarithm_gldm_HighGrayLevelEmphasis | 22.92 | 63.52 | 0.406 | 0.036 | 0.686 | 21.4 | -105.6 |
| logarithm_gldm_LargeDependenceEmphasis | 11.17 | 30.95 | 0.948 | 0.885 | 0.977 | 37.4 | -24.5 |
| logarithm_gldm_LargeDependenceHighGrayLevelEmphasis | 21.00 | 58.22 | 0.555 | 0.182 | 0.784 | 23.5 | -93 |
| logarithm_gldm_LargeDependenceLowGrayLevelEmphasis | 35.99 | 99.75 | 0.836 | 0.662 | 0.925 | 111.1 | -88.4 |
| logarithm_gldm_LowGrayLevelEmphasis | 30.25 | 83.86 | 0.712 | 0.444 | 0.863 | 97.7 | -70 |
| logarithm_gldm_SmallDependenceEmphasis | 3.99 | 11.06 | 0.935 | 0.841 | 0.972 | 8.9 | -13.2 |
| logarithm_gldm_SmallDependenceHighGrayLevelEmphasis | 27.59 | 76.48 | 0.366 | 0.001 | 0.657 | 32.5 | -120.5 |
| logarithm_gldm_SmallDependenceLowGrayLevelEmphasis | 28.77 | 79.75 | 0.657 | 0.359 | 0.835 | 97.8 | -61.7 |
| logarithm_glrlm_GrayLevelNonUniformity | 12.89 | 35.72 | 0.941 | 0.864 | 0.974 | 47.4 | -24 |
| logarithm_glrlm_GrayLevelNonUniformityNormalized | 12.55 | 34.79 | 0.956 | 0.802 | 0.985 | 55.1 | -14.5 |
| logarithm_glrlm_GrayLevelVariance | 23.24 | 64.41 | 0.468 | 0.08 | 0.731 | 21.1 | -107.7 |
| logarithm_glrlm_HighGrayLevelRunEmphasis | 22.99 | 63.71 | 0.406 | 0.036 | 0.685 | 21.6 | -105.8 |
| logarithm_glrlm_LongRunEmphasis | 0.73 | 2.04 | 0.952 | 0.893 | 0.979 | 2.4 | -1.7 |
| logarithm_glrlm_LongRunHighGrayLevelEmphasis | 22.48 | 62.31 | 0.413 | 0.042 | 0.69 | 20.6 | -104 |
| logarithm_glrlm_LongRunLowGrayLevelEmphasis | 30.57 | 84.72 | 0.727 | 0.47 | 0.871 | 98.5 | -71 |
| logarithm_glrlm_LowGrayLevelRunEmphasis | 30.16 | 83.61 | 0.716 | 0.451 | 0.865 | 97.7 | -69.5 |
| logarithm_glrlm_RunEntropy | 2.34 | 6.48 | 0.908 | 0.308 | 0.974 | 2.5 | -10.5 |
| logarithm_glrlm_RunLengthNonUniformity | 7.73 | 21.44 | 0.996 | 0.992 | 0.998 | 12.5 | -30.4 |
| logarithm_glrlm_RunLengthNonUniformityNormalized | 0.45 | 1.26 | 0.951 | 0.887 | 0.979 | 1 | -1.5 |
| logarithm_glrlm_RunPercentage | 0.24 | 0.66 | 0.952 | 0.89 | 0.979 | 0.5 | -0.8 |
| logarithm_glrlm_RunVariance | 23.67 | 65.61 | 0.949 | 0.887 | 0.978 | 80.7 | -50.5 |
| logarithm_glrlm_ShortRunEmphasis | 0.18 | 0.49 | 0.953 | 0.892 | 0.98 | 0.4 | -0.6 |
| logarithm_glrlm_ShortRunHighGrayLevelEmphasis | 23.13 | 64.11 | 0.404 | 0.034 | 0.684 | 21.9 | -106.3 |
| logarithm_glrlm_ShortRunLowGrayLevelEmphasis | 30.06 | 83.34 | 0.713 | 0.446 | 0.864 | 97.6 | -69.1 |
| logarithm_glszm_GrayLevelNonUniformity | 11.81 | 32.75 | 0.951 | 0.884 | 0.979 | 43.1 | -22.4 |
| logarithm_glszm_GrayLevelNonUniformityNormalized | 13.00 | 36.04 | 0.938 | 0.819 | 0.976 | 56.3 | -15.8 |
| logarithm_glszm_GrayLevelVariance | 24.27 | 67.29 | 0.467 | 0.079 | 0.731 | 23.8 | -110.8 |
| logarithm_glszm_HighGrayLevelZoneEmphasis | 24.40 | 67.64 | 0.401 | 0.032 | 0.682 | 25.2 | -110.1 |
| logarithm_glszm_LargeAreaEmphasis | 9.07 | 25.15 | 0.972 | 0.936 | 0.988 | 30.1 | -20.2 |
| logarithm_glszm_LargeAreaHighGrayLevelEmphasis | 20.53 | 56.91 | 0.508 | 0.134 | 0.754 | 20.6 | -93.2 |
| logarithm_glszm_LargeAreaLowGrayLevelEmphasis | 33.71 | 93.44 | 0.867 | 0.72 | 0.94 | 107.1 | -79.8 |
| logarithm_glszm_LowGrayLevelZoneEmphasis | 29.40 | 81.49 | 0.769 | 0.54 | 0.892 | 99.1 | -63.9 |
| logarithm_glszm_SizeZoneNonUniformity | 11.25 | 31.19 | 0.985 | 0.963 | 0.994 | 18.7 | -43.6 |
| logarithm_glszm_SizeZoneNonUniformityNormalized | 4.70 | 13.03 | 0.918 | 0.803 | 0.965 | 10.6 | -15.5 |
| logarithm_glszm_SmallAreaEmphasis | 2.12 | 5.88 | 0.94 | 0.862 | 0.974 | 4.9 | -6.8 |
| logarithm_glszm_SmallAreaHighGrayLevelEmphasis | 27.43 | 76.04 | 0.381 | 0.014 | 0.668 | 33 | -119.1 |
| logarithm_glszm_SmallAreaLowGrayLevelEmphasis | 29.79 | 82.59 | 0.726 | 0.468 | 0.871 | 103.5 | -61.7 |
| logarithm_glszm_ZoneEntropy | 2.01 | 5.57 | 0.906 | 0.272 | 0.974 | 2.2 | -9 |
| logarithm_glszm_ZonePercentage | 2.93 | 8.11 | 0.949 | 0.876 | 0.978 | 6.6 | -9.6 |
| logarithm_glszm_ZoneVariance | 31.80 | 88.16 | 0.952 | 0.894 | 0.979 | 107 | -69.3 |
| logarithm_ngtdm_Busyness | 21.30 | 59.03 | 0.96 | 0.911 | 0.983 | 90.7 | -27.4 |
| logarithm_ngtdm_Coarseness | 7.22 | 20.02 | 0.92 | 0.778 | 0.968 | 28.1 | -12 |
| logarithm_ngtdm_Complexity | 29.34 | 81.32 | 0.244 | -0.125 | 0.57 | 24 | -138.6 |
| logarithm_ngtdm_Contrast | 17.98 | 49.83 | 0.778 | 0.332 | 0.916 | 25.7 | -74 |
| logarithm_ngtdm_Strength | 20.50 | 56.82 | 0.719 | 0.077 | 0.903 | 21.9 | -91.8 |
| original_firstorder_10Percentile | 4.96 | 13.74 | 0.792 | 0.58 | 0.904 | 13.9 | -13.5 |
| original_firstorder_90Percentile | 4.08 | 11.31 | 0.995 | 0.987 | 0.998 | 9.1 | -13.5 |
| original_firstorder_Energy | 8.91 | 24.71 | 0.998 | 0.995 | 0.999 | 13.6 | -35.8 |
| original_firstorder_Entropy | 1.66 | 4.60 | 0.993 | 0.981 | 0.997 | 3.7 | -5.5 |
| original_firstorder_InterquartileRange | 6.10 | 16.91 | 0.995 | 0.988 | 0.998 | 14.8 | -19 |
| original_firstorder_Kurtosis | 5.00 | 13.87 | 0.968 | 0.929 | 0.986 | 10 | -17.8 |
| original_firstorder_Maximum | 4.34 | 12.03 | 0.995 | 0.972 | 0.998 | 7.9 | -16.1 |
| original_firstorder_MeanAbsoluteDeviation | 6.04 | 16.75 | 0.995 | 0.987 | 0.998 | 13.1 | -20.4 |
| original_firstorder_Mean | 3.03 | 8.41 | 0.995 | 0.988 | 0.998 | 7.7 | -9.1 |
| original_firstorder_Median | 3.37 | 9.33 | 0.984 | 0.963 | 0.993 | 10 | -8.6 |
| original_firstorder_Minimum | 6.24 | 17.30 | 0.93 | 0.832 | 0.971 | 21.1 | -13.5 |
| original_firstorder_Range | 5.85 | 16.22 | 0.995 | 0.967 | 0.998 | 11.1 | -21.4 |
| original_firstorder_RobustMeanAbsoluteDeviation | 6.45 | 17.87 | 0.994 | 0.986 | 0.998 | 14.7 | -21.1 |
| original_firstorder_RootMeanSquared | 3.22 | 8.93 | 0.996 | 0.99 | 0.998 | 7.7 | -10.2 |
| original_firstorder_Skewness | 14.62 | 40.53 | 0.96 | 0.894 | 0.984 | 29.1 | -52 |
| original_firstorder_TotalEnergy | 8.91 | 24.71 | 0.998 | 0.995 | 0.999 | 13.6 | -35.8 |
| original_firstorder_Uniformity | 5.46 | 15.13 | 0.981 | 0.957 | 0.992 | 17.5 | -12.8 |
| original_firstorder_Variance | 11.67 | 32.34 | 0.996 | 0.988 | 0.999 | 24.4 | -40.3 |
| original_glcm_Autocorrelation | 8.52 | 23.62 | 0.996 | 0.99 | 0.999 | 18.8 | -28.4 |
| original_glcm_ClusterProminence | 25.29 | 70.10 | 0.989 | 0.974 | 0.995 | 49.4 | -90.8 |
| original_glcm_ClusterShade | 33.09 | 91.73 | 0.989 | 0.971 | 0.995 | 62.1 | -121.3 |
| original_glcm_ClusterTendency | 12.80 | 35.47 | 0.996 | 0.987 | 0.999 | 26.3 | -44.6 |
| original_glcm_Contrast | 10.09 | 27.97 | 0.991 | 0.978 | 0.996 | 23.9 | -32.1 |
| original_glcm_Correlation | 2.49 | 6.91 | 0.966 | 0.897 | 0.987 | 4.8 | -9 |
| original_glcm_DifferenceAverage | 5.05 | 13.98 | 0.994 | 0.985 | 0.997 | 12.2 | -15.8 |
| original_glcm_DifferenceEntropy | 1.93 | 5.35 | 0.994 | 0.985 | 0.998 | 4.4 | -6.3 |
| original_glcm_DifferenceVariance | 10.60 | 29.38 | 0.991 | 0.978 | 0.996 | 24.1 | -34.7 |
| original_glcm_Id | 3.58 | 9.92 | 0.992 | 0.982 | 0.996 | 10.1 | -9.7 |
| original_glcm_Idm | 5.93 | 16.43 | 0.989 | 0.975 | 0.995 | 15.9 | -16.9 |
| original_glcm_Idmn | 0.14 | 0.40 | 0.973 | 0.896 | 0.99 | 0.3 | -0.5 |
| original_glcm_Idn | 0.32 | 0.89 | 0.979 | 0.903 | 0.993 | 0.5 | -1.2 |
| original_glcm_Imc1 | 4.58 | 12.68 | 0.99 | 0.978 | 0.996 | 13.9 | -11.4 |
| original_glcm_Imc2 | 1.17 | 3.25 | 0.935 | 0.857 | 0.971 | 3.2 | -3.3 |
| original_glcm_InverseVariance | 6.21 | 17.20 | 0.99 | 0.976 | 0.995 | 18.2 | -16.2 |
| original_glcm_JointAverage | 4.23 | 11.71 | 0.996 | 0.988 | 0.998 | 9.8 | -13.7 |
| original_glcm_JointEnergy | 8.41 | 23.31 | 0.951 | 0.888 | 0.978 | 30.4 | -16.2 |
| original_glcm_JointEntropy | 1.66 | 4.61 | 0.989 | 0.96 | 0.996 | 3.2 | -6 |
| original_glcm_MCC | 2.32 | 6.44 | 0.934 | 0.854 | 0.971 | 5.5 | -7.4 |
| original_glcm_MaximumProbability | 9.74 | 26.99 | 0.975 | 0.944 | 0.989 | 26.3 | -27.7 |
| original_glcm_SumAverage | 4.23 | 11.71 | 0.996 | 0.988 | 0.998 | 9.8 | -13.7 |
| original_glcm_SumEntropy | 1.59 | 4.40 | 0.991 | 0.973 | 0.997 | 3.3 | -5.5 |
| original_glcm_SumSquares | 12.26 | 33.98 | 0.996 | 0.987 | 0.998 | 25.6 | -42.4 |
| original_gldm_DependenceEntropy | 1.31 | 3.62 | 0.981 | 0.93 | 0.993 | 2.4 | -4.8 |
| original_gldm_DependenceNonUniformity | 9.06 | 25.10 | 0.997 | 0.993 | 0.999 | 18.2 | -32 |
| original_gldm_DependenceNonUniformityNormalized | 5.57 | 15.43 | 0.966 | 0.923 | 0.985 | 17.2 | -13.7 |
| original_gldm_DependenceVariance | 15.16 | 42.01 | 0.91 | 0.806 | 0.96 | 36.4 | -47.6 |
| original_gldm_GrayLevelNonUniformity | 8.37 | 23.19 | 0.995 | 0.989 | 0.998 | 16.9 | -29.5 |
| original_gldm_GrayLevelVariance | 11.58 | 32.09 | 0.996 | 0.988 | 0.999 | 24.2 | -40 |
| original_gldm_HighGrayLevelEmphasis | 8.77 | 24.31 | 0.997 | 0.99 | 0.999 | 19.4 | -29.2 |
| original_gldm_LargeDependenceEmphasis | 9.21 | 25.53 | 0.96 | 0.911 | 0.982 | 21.5 | -29.5 |
| original_gldm_LargeDependenceHighGrayLevelEmphasis | 11.73 | 32.51 | 0.977 | 0.949 | 0.99 | 23.4 | -41.6 |
| original_gldm_LargeDependenceLowGrayLevelEmphasis | 46.91 | 130.02 | 0.848 | 0.682 | 0.931 | 120.4 | -139.6 |
| original_gldm_LowGrayLevelEmphasis | 30.98 | 85.87 | 0.525 | 0.169 | 0.762 | 89.9 | -81.8 |
| original_gldm_SmallDependenceEmphasis | 5.62 | 15.57 | 0.977 | 0.945 | 0.99 | 18.7 | -12.5 |
| original_gldm_SmallDependenceHighGrayLevelEmphasis | 17.51 | 48.54 | 0.996 | 0.99 | 0.999 | 46.5 | -50.6 |
| original_gldm_SmallDependenceLowGrayLevelEmphasis | 29.07 | 80.58 | 0.395 | 0.01 | 0.682 | 94.3 | -66.9 |
| original_glrlm_GrayLevelNonUniformity | 8.09 | 22.42 | 0.996 | 0.99 | 0.998 | 16.3 | -28.5 |
| original_glrlm_GrayLevelNonUniformityNormalized | 5.48 | 15.19 | 0.98 | 0.955 | 0.991 | 17.6 | -12.8 |
| original_glrlm_GrayLevelVariance | 11.71 | 32.46 | 0.996 | 0.988 | 0.999 | 24.5 | -40.4 |
| original_glrlm_HighGrayLevelRunEmphasis | 8.93 | 24.76 | 0.997 | 0.99 | 0.999 | 19.8 | -29.7 |
| original_glrlm_LongRunEmphasis | 1.24 | 3.43 | 0.974 | 0.941 | 0.988 | 3 | -3.9 |
| original_glrlm_LongRunHighGrayLevelEmphasis | 7.59 | 21.04 | 0.996 | 0.989 | 0.999 | 15.9 | -26.2 |
| original_glrlm_LongRunLowGrayLevelEmphasis | 32.03 | 88.77 | 0.6 | 0.273 | 0.804 | 91.5 | -86.1 |
| original_glrlm_LowGrayLevelRunEmphasis | 30.71 | 85.11 | 0.541 | 0.19 | 0.771 | 89.5 | -80.7 |
| original_glrlm_RunEntropy | 1.34 | 3.73 | 0.992 | 0.975 | 0.997 | 2.7 | -4.8 |
| original_glrlm_RunLengthNonUniformity | 7.40 | 20.50 | 0.997 | 0.992 | 0.998 | 12.3 | -28.7 |
| original_glrlm_RunLengthNonUniformityNormalized | 0.77 | 2.14 | 0.973 | 0.94 | 0.988 | 2.4 | -1.9 |
| original_glrlm_RunPercentage | 0.40 | 1.11 | 0.975 | 0.944 | 0.989 | 1.2 | -1 |
| original_glrlm_RunVariance | 11.91 | 33.01 | 0.977 | 0.947 | 0.99 | 26.9 | -39.1 |
| original_glrlm_ShortRunEmphasis | 0.32 | 0.88 | 0.971 | 0.936 | 0.987 | 1 | -0.8 |
| original_glrlm_ShortRunHighGrayLevelEmphasis | 9.31 | 25.80 | 0.997 | 0.99 | 0.999 | 20.9 | -30.7 |
| original_glrlm_ShortRunLowGrayLevelEmphasis | 30.53 | 84.64 | 0.527 | 0.172 | 0.763 | 89.3 | -79.9 |
| original_glszm_GrayLevelNonUniformity | 6.21 | 17.21 | 0.996 | 0.991 | 0.998 | 13.5 | -20.9 |
| original_glszm_GrayLevelNonUniformityNormalized | 7.57 | 20.98 | 0.949 | 0.886 | 0.977 | 23.7 | -18.2 |
| original_glszm_GrayLevelVariance | 14.18 | 39.31 | 0.995 | 0.985 | 0.998 | 29.7 | -48.9 |
| original_glszm_HighGrayLevelZoneEmphasis | 12.42 | 34.43 | 0.997 | 0.986 | 0.999 | 28.8 | -40.1 |
| original_glszm_LargeAreaEmphasis | 15.70 | 43.52 | 0.931 | 0.849 | 0.969 | 38 | -49.1 |
| original_glszm_LargeAreaHighGrayLevelEmphasis | 13.60 | 37.69 | 0.982 | 0.959 | 0.992 | 28 | -47.4 |
| original_glszm_LargeAreaLowGrayLevelEmphasis | 45.46 | 126.01 | 0.898 | 0.781 | 0.954 | 117.9 | -134.1 |
| original_glszm_LowGrayLevelZoneEmphasis | 29.42 | 81.54 | 0.739 | 0.489 | 0.877 | 90.6 | -72.5 |
| original_glszm_SizeZoneNonUniformity | 9.50 | 26.34 | 0.997 | 0.994 | 0.999 | 24.2 | -28.5 |
| original_glszm_SizeZoneNonUniformityNormalized | 6.47 | 17.94 | 0.966 | 0.911 | 0.986 | 22.3 | -13.6 |
| original_glszm_SmallAreaEmphasis | 3.84 | 10.65 | 0.961 | 0.899 | 0.984 | 13.1 | -8.2 |
| original_glszm_SmallAreaHighGrayLevelEmphasis | 17.76 | 49.22 | 0.996 | 0.987 | 0.998 | 46.8 | -51.6 |
| original_glszm_SmallAreaLowGrayLevelEmphasis | 30.89 | 85.61 | 0.608 | 0.285 | 0.808 | 100.5 | -70.8 |
| original_glszm_ZoneEntropy | 2.00 | 5.54 | 0.973 | 0.927 | 0.989 | 4.3 | -6.8 |
| original_glszm_ZonePercentage | 4.95 | 13.72 | 0.981 | 0.956 | 0.992 | 15.9 | -11.6 |
| original_glszm_ZoneVariance | 25.58 | 70.91 | 0.89 | 0.766 | 0.951 | 63.1 | -78.7 |
| original_ngtdm_Busyness | 12.22 | 33.86 | 0.969 | 0.931 | 0.987 | 35 | -32.7 |
| original_ngtdm_Coarseness | 6.65 | 18.44 | 0.939 | 0.846 | 0.974 | 24.1 | -12.8 |
| original_ngtdm_Complexity | 14.87 | 41.21 | 0.991 | 0.974 | 0.996 | 30.5 | -51.9 |
| original_ngtdm_Contrast | 9.02 | 25.00 | 0.979 | 0.953 | 0.991 | 27.7 | -22.3 |
| original_ngtdm_Strength | 13.48 | 37.35 | 0.986 | 0.963 | 0.995 | 29.5 | -45.2 |
| square_firstorder_10Percentile | 18.67 | 51.75 | 0.885 | 0.556 | 0.96 | 76.8 | -26.7 |
| square_firstorder_90Percentile | 12.85 | 35.61 | 0.923 | 0.772 | 0.97 | 56.3 | -14.9 |
| square_firstorder_Energy | 26.36 | 73.06 | 0.858 | 0.697 | 0.936 | 105.7 | -40.4 |
| square_firstorder_Entropy | 10.98 | 30.45 | 0.932 | 0.438 | 0.981 | 42.4 | -18.5 |
| square_firstorder_InterquartileRange | 13.86 | 38.42 | 0.913 | 0.677 | 0.969 | 61.3 | -15.5 |
| square_firstorder_Kurtosis | 7.06 | 19.58 | 0.995 | 0.979 | 0.998 | 12.2 | -27 |
| square_firstorder_Maximum | 14.60 | 40.48 | 0.91 | 0.759 | 0.964 | 57.4 | -23.5 |
| square_firstorder_MeanAbsoluteDeviation | 14.53 | 40.27 | 0.936 | 0.789 | 0.976 | 60.1 | -20.4 |
| square_firstorder_Mean | 13.45 | 37.29 | 0.916 | 0.682 | 0.97 | 59.9 | -14.7 |
| square_firstorder_Median | 14.68 | 40.70 | 0.824 | 0.236 | 0.943 | 67.1 | -14.3 |
| square_firstorder_Minimum | 19.48 | 54.00 | 0.956 | 0.716 | 0.986 | 86 | -22 |
| square_firstorder_Range | 15.08 | 41.81 | 0.911 | 0.761 | 0.964 | 58.4 | -25.2 |
| square_firstorder_RobustMeanAbsoluteDeviation | 13.61 | 37.73 | 0.917 | 0.744 | 0.968 | 59.1 | -16.4 |
| square_firstorder_RootMeanSquared | 13.76 | 38.15 | 0.931 | 0.758 | 0.975 | 59.3 | -17 |
| square_firstorder_Skewness | 9.92 | 27.50 | 0.986 | 0.937 | 0.995 | 19.3 | -35.7 |
| square_firstorder_TotalEnergy | 26.36 | 73.06 | 0.858 | 0.697 | 0.936 | 105.7 | -40.4 |
| square_firstorder_Uniformity | 13.80 | 38.26 | 0.801 | 0.397 | 0.925 | 13.6 | -62.9 |
| square_firstorder_Variance | 27.60 | 76.51 | 0.853 | 0.688 | 0.934 | 112.5 | -40.5 |
| square_glcm_Autocorrelation | 22.89 | 63.44 | 0.84 | 0.662 | 0.928 | 101.8 | -25.1 |
| square_glcm_ClusterProminence | 45.39 | 125.81 | 0.658 | 0.361 | 0.835 | 185.7 | -65.9 |
| square_glcm_ClusterShade | 39.85 | 110.45 | 0.77 | 0.543 | 0.893 | 150.7 | -70.2 |
| square_glcm_ClusterTendency | 27.64 | 76.61 | 0.856 | 0.694 | 0.935 | 113.2 | -40 |
| square_glcm_Contrast | 23.80 | 65.96 | 0.84 | 0.637 | 0.93 | 106.7 | -25.2 |
| square_glcm_Correlation | 3.30 | 9.16 | 0.965 | 0.915 | 0.985 | 7.3 | -11 |
| square_glcm_DifferenceAverage | 15.05 | 41.72 | 0.917 | 0.627 | 0.972 | 66.1 | -17.4 |
| square_glcm_DifferenceEntropy | 6.83 | 18.93 | 0.962 | 0.569 | 0.99 | 28.8 | -9.1 |
| square_glcm_DifferenceVariance | 23.08 | 63.97 | 0.857 | 0.671 | 0.938 | 99.8 | -28.2 |
| square_glcm_Id | 5.52 | 15.30 | 0.93 | 0.373 | 0.981 | 4.6 | -26 |
| square_glcm_Idm | 7.04 | 19.53 | 0.935 | 0.394 | 0.983 | 5.8 | -33.3 |
| square_glcm_Idmn | 0.45 | 1.24 | 0.813 | 0.579 | 0.918 | 0.9 | -1.6 |
| square_glcm_Idn | 1.05 | 2.90 | 0.841 | 0.6 | 0.934 | 2 | -3.8 |
| square_glcm_Imc1 | 6.33 | 17.55 | 0.947 | 0.883 | 0.977 | 16.8 | -18.3 |
| square_glcm_Imc2 | 4.11 | 11.38 | 0.949 | 0.881 | 0.978 | 13.3 | -9.5 |
| square_glcm_InverseVariance | 12.95 | 35.88 | 0.822 | 0.636 | 0.919 | 34 | -37.8 |
| square_glcm_JointAverage | 11.52 | 31.93 | 0.914 | 0.666 | 0.97 | 52 | -11.8 |
| square_glcm_JointEnergy | 23.32 | 64.65 | 0.698 | 0.4 | 0.859 | 22.2 | -107.1 |
| square_glcm_JointEntropy | 9.55 | 26.46 | 0.94 | 0.537 | 0.983 | 36.6 | -16.4 |
| square_glcm_MCC | 3.66 | 10.14 | 0.959 | 0.91 | 0.982 | 9.6 | -10.7 |
| square_glcm_MaximumProbability | 21.23 | 58.84 | 0.643 | 0.22 | 0.844 | 20.7 | -96.9 |
| square_glcm_SumAverage | 11.52 | 31.93 | 0.914 | 0.666 | 0.97 | 52 | -11.8 |
| square_glcm_SumEntropy | 8.51 | 23.60 | 0.943 | 0.592 | 0.983 | 32.3 | -15 |
| square_glcm_SumSquares | 26.84 | 74.40 | 0.855 | 0.69 | 0.935 | 111.7 | -37.1 |
| square_gldm_DependenceEntropy | 2.62 | 7.27 | 0.95 | 0.836 | 0.981 | 9.5 | -5 |
| square_gldm_DependenceNonUniformity | 12.53 | 34.74 | 0.963 | 0.915 | 0.984 | 40.4 | -29.1 |
| square_gldm_DependenceNonUniformityNormalized | 10.96 | 30.38 | 0.758 | 0.174 | 0.916 | 44.6 | -16.2 |
| square_gldm_DependenceVariance | 20.82 | 57.72 | 0.673 | 0.309 | 0.853 | 24.6 | -90.8 |
| square_gldm_GrayLevelNonUniformity | 15.88 | 44.02 | 0.758 | 0.506 | 0.889 | 11.2 | -76.9 |
| square_gldm_GrayLevelVariance | 27.40 | 75.94 | 0.854 | 0.689 | 0.934 | 114.3 | -37.5 |
| square_gldm_HighGrayLevelEmphasis | 22.80 | 63.19 | 0.842 | 0.667 | 0.929 | 101.1 | -25.3 |
| square_gldm_LargeDependenceEmphasis | 17.38 | 48.18 | 0.805 | 0.503 | 0.92 | 13.1 | -83.3 |
| square_gldm_LargeDependenceHighGrayLevelEmphasis | 16.99 | 47.09 | 0.907 | 0.79 | 0.959 | 58.4 | -35.7 |
| square_gldm_LargeDependenceLowGrayLevelEmphasis | 24.63 | 68.27 | 0.494 | 0.104 | 0.749 | 14 | -122.5 |
| square_gldm_LowGrayLevelEmphasis | 10.91 | 30.23 | 0.767 | 0.086 | 0.925 | 11.5 | -49 |
| square_gldm_SmallDependenceEmphasis | 17.90 | 49.61 | 0.909 | 0.367 | 0.974 | 78.1 | -21.1 |
| square_gldm_SmallDependenceHighGrayLevelEmphasis | 33.85 | 93.84 | 0.831 | 0.647 | 0.923 | 141 | -46.6 |
| square_gldm_SmallDependenceLowGrayLevelEmphasis | 17.59 | 48.77 | 0.653 | 0.021 | 0.874 | 78.5 | -19.1 |
| square_glrlm_GrayLevelNonUniformity | 9.52 | 26.38 | 0.944 | 0.829 | 0.978 | 3.2 | -49.6 |
| square_glrlm_GrayLevelNonUniformityNormalized | 11.66 | 32.32 | 0.854 | 0.584 | 0.943 | 11.7 | -52.9 |
| square_glrlm_GrayLevelVariance | 25.94 | 71.91 | 0.866 | 0.712 | 0.94 | 108.4 | -35.4 |
| square_glrlm_HighGrayLevelRunEmphasis | 21.34 | 59.16 | 0.861 | 0.702 | 0.938 | 94 | -24.3 |
| square_glrlm_LongRunEmphasis | 14.14 | 39.18 | 0.774 | 0.492 | 0.902 | 21.1 | -57.3 |
| square_glrlm_LongRunHighGrayLevelEmphasis | 19.93 | 55.25 | 0.864 | 0.707 | 0.939 | 82.6 | -27.9 |
| square_glrlm_LongRunLowGrayLevelEmphasis | 21.91 | 60.74 | 0.55 | 0.175 | 0.781 | 22.3 | -99.2 |
| square_glrlm_LowGrayLevelRunEmphasis | 9.73 | 26.97 | 0.845 | 0.25 | 0.952 | 11.3 | -42.7 |
| square_glrlm_RunEntropy | 2.84 | 7.88 | 0.973 | 0.864 | 0.991 | 10.7 | -5 |
| square_glrlm_RunLengthNonUniformity | 11.68 | 32.38 | 0.984 | 0.964 | 0.993 | 38.6 | -26.2 |
| square_glrlm_RunLengthNonUniformityNormalized | 8.00 | 22.18 | 0.902 | 0.442 | 0.97 | 31 | -13.3 |
| square_glrlm_RunPercentage | 5.87 | 16.28 | 0.868 | 0.426 | 0.957 | 22.6 | -10 |
| square_glrlm_RunVariance | 17.28 | 47.90 | 0.757 | 0.418 | 0.898 | 12 | -83.8 |
| square_glrlm_ShortRunEmphasis | 5.74 | 15.92 | 0.879 | 0.6 | 0.955 | 20.7 | -11.1 |
| square_glrlm_ShortRunHighGrayLevelEmphasis | 23.23 | 64.39 | 0.86 | 0.7 | 0.937 | 101.7 | -27.1 |
| square_glrlm_ShortRunLowGrayLevelEmphasis | 8.03 | 22.27 | 0.907 | 0.662 | 0.967 | 13.9 | -30.7 |
| square_glszm_GrayLevelNonUniformity | 13.44 | 37.26 | 0.983 | 0.959 | 0.993 | 50 | -24.5 |
| square_glszm_GrayLevelNonUniformityNormalized | 13.58 | 37.64 | 0.986 | 0.969 | 0.994 | 28.8 | -46.5 |
| square_glszm_GrayLevelVariance | 25.89 | 71.76 | 0.871 | 0.716 | 0.943 | 104.2 | -39.3 |
| square_glszm_HighGrayLevelZoneEmphasis | 19.76 | 54.76 | 0.901 | 0.776 | 0.957 | 75.3 | -34.3 |
| square_glszm_LargeAreaEmphasis | 29.22 | 80.98 | 0.917 | 0.801 | 0.965 | 13.9 | -148 |
| square_glszm_LargeAreaHighGrayLevelEmphasis | 22.13 | 61.33 | 0.991 | 0.976 | 0.996 | 33.6 | -89.1 |
| square_glszm_LargeAreaLowGrayLevelEmphasis | 36.58 | 101.39 | 0.82 | 0.612 | 0.92 | 19.2 | -183.6 |
| square_glszm_LowGrayLevelZoneEmphasis | 25.18 | 69.79 | 0.941 | 0.864 | 0.975 | 88.5 | -51.1 |
| square_glszm_SizeZoneNonUniformity | 31.17 | 86.41 | 0.948 | 0.877 | 0.978 | 110.5 | -62.3 |
| square_glszm_SizeZoneNonUniformityNormalized | 19.07 | 52.85 | 0.86 | 0.704 | 0.937 | 56.6 | -49.1 |
| square_glszm_SmallAreaEmphasis | 44.17 | 122.43 | 0.81 | 0.613 | 0.912 | 119.7 | -125.2 |
| square_glszm_SmallAreaHighGrayLevelEmphasis | 58.86 | 163.15 | 0.898 | 0.768 | 0.955 | 173.4 | -152.9 |
| square_glszm_SmallAreaLowGrayLevelEmphasis | 65.93 | 182.74 | 0.88 | 0.745 | 0.946 | 210.9 | -154.5 |
| square_glszm_ZoneEntropy | 4.90 | 13.59 | 0.984 | 0.934 | 0.994 | 17.7 | -9.5 |
| square_glszm_ZonePercentage | 16.34 | 45.30 | 0.916 | 0.346 | 0.977 | 75 | -15.6 |
| square_glszm_ZoneVariance | 31.99 | 88.68 | 0.885 | 0.735 | 0.95 | 18.5 | -158.8 |
| square_ngtdm_Busyness | 25.39 | 70.38 | 0.97 | 0.933 | 0.987 | 42.4 | -98.4 |
| square_ngtdm_Coarseness | 5.76 | 15.96 | 0.968 | 0.911 | 0.987 | 22.1 | -9.9 |
| square_ngtdm_Complexity | 31.99 | 88.66 | 0.758 | 0.52 | 0.887 | 134.9 | -42.4 |
| square_ngtdm_Contrast | 24.91 | 69.05 | 0.822 | 0.437 | 0.934 | 106.1 | -32 |
| square_ngtdm_Strength | 28.86 | 80.00 | 0.927 | 0.822 | 0.969 | 102 | -58 |
| squareroot_firstorder_10Percentile | 6.48 | 17.95 | 0.606 | -0.059 | 0.86 | 5.4 | -30.5 |
| squareroot_firstorder_90Percentile | 7.93 | 21.97 | 0.827 | 0.212 | 0.946 | 8.2 | -35.7 |
| squareroot_firstorder_Energy | 15.57 | 43.17 | 0.86 | 0.7 | 0.937 | 9.2 | -77.1 |
| squareroot_firstorder_Entropy | 2.12 | 5.88 | 0.953 | 0.562 | 0.987 | 2.7 | -9.1 |
| squareroot_firstorder_InterquartileRange | 9.86 | 27.32 | 0.901 | 0.538 | 0.968 | 12.6 | -42.1 |
| squareroot_firstorder_Kurtosis | 4.07 | 11.29 | 0.914 | 0.814 | 0.962 | 9.6 | -13 |
| squareroot_firstorder_Maximum | 7.63 | 21.15 | 0.843 | 0.382 | 0.947 | 6.4 | -35.9 |
| squareroot_firstorder_MeanAbsoluteDeviation | 9.63 | 26.69 | 0.897 | 0.468 | 0.967 | 10.9 | -42.5 |
| squareroot_firstorder_Mean | 7.26 | 20.11 | 0.758 | 0.1 | 0.92 | 7.3 | -33 |
| squareroot_firstorder_Median | 7.22 | 20.00 | 0.731 | 0.097 | 0.908 | 7.7 | -32.3 |
| squareroot_firstorder_Minimum | 6.90 | 19.13 | 0.609 | -0.042 | 0.858 | 8.4 | -29.9 |
| squareroot_firstorder_Range | 8.68 | 24.07 | 0.875 | 0.516 | 0.956 | 7.5 | -40.7 |
| squareroot_firstorder_RobustMeanAbsoluteDeviation | 10.24 | 28.39 | 0.898 | 0.473 | 0.968 | 12.8 | -44 |
| squareroot_firstorder_RootMeanSquared | 7.36 | 20.40 | 0.778 | 0.131 | 0.928 | 7.4 | -33.4 |
| squareroot_firstorder_Skewness | 27.93 | 77.41 | 0.919 | 0.796 | 0.966 | 56.2 | -98.6 |
| squareroot_firstorder_TotalEnergy | 15.57 | 43.17 | 0.86 | 0.7 | 0.937 | 9.2 | -77.1 |
| squareroot_firstorder_Uniformity | 9.77 | 27.07 | 0.968 | 0.876 | 0.989 | 42.3 | -11.9 |
| squareroot_firstorder_Variance | 17.84 | 49.45 | 0.809 | 0.45 | 0.926 | 18.5 | -80.4 |
| squareroot_glcm_Autocorrelation | 16.74 | 46.41 | 0.755 | 0.357 | 0.902 | 17.9 | -75 |
| squareroot_glcm_ClusterProminence | 32.04 | 88.82 | 0.518 | 0.167 | 0.756 | 27.4 | -150.2 |
| squareroot_glcm_ClusterShade | 68.05 | 188.62 | 0.643 | 0.316 | 0.831 | 97.2 | -280.1 |
| squareroot_glcm_ClusterTendency | 18.20 | 50.45 | 0.818 | 0.478 | 0.929 | 18.3 | -82.6 |
| squareroot_glcm_Contrast | 17.85 | 49.47 | 0.797 | 0.309 | 0.928 | 21.8 | -77.2 |
| squareroot_glcm_Correlation | 2.58 | 7.14 | 0.965 | 0.905 | 0.986 | 5.3 | -9 |
| squareroot_glcm_DifferenceAverage | 9.34 | 25.88 | 0.892 | 0.386 | 0.967 | 11.8 | -39.9 |
| squareroot_glcm_DifferenceEntropy | 2.42 | 6.72 | 0.954 | 0.581 | 0.987 | 3.1 | -10.3 |
| squareroot_glcm_DifferenceVariance | 17.55 | 48.64 | 0.771 | 0.325 | 0.913 | 20.8 | -76.5 |
| squareroot_glcm_Id | 7.29 | 20.20 | 0.975 | 0.907 | 0.991 | 29.3 | -11.1 |
| squareroot_glcm_Idm | 11.65 | 32.30 | 0.978 | 0.945 | 0.991 | 42.4 | -22.2 |
| squareroot_glcm_Idmn | 0.15 | 0.40 | 0.97 | 0.912 | 0.988 | 0.3 | -0.5 |
| squareroot_glcm_Idn | 0.31 | 0.86 | 0.977 | 0.926 | 0.991 | 0.6 | -1.1 |
| squareroot_glcm_Imc1 | 5.73 | 15.88 | 0.977 | 0.925 | 0.991 | 11.8 | -19.9 |
| squareroot_glcm_Imc2 | 0.73 | 2.01 | 0.906 | 0.797 | 0.958 | 1.8 | -2.2 |
| squareroot_glcm_InverseVariance | 11.53 | 31.97 | 0.977 | 0.922 | 0.992 | 44.7 | -19.3 |
| squareroot_glcm_JointAverage | 8.71 | 24.15 | 0.865 | 0.378 | 0.956 | 9.9 | -38.4 |
| squareroot_glcm_JointEnergy | 11.09 | 30.74 | 0.931 | 0.816 | 0.972 | 44.3 | -17.2 |
| squareroot_glcm_JointEntropy | 1.73 | 4.79 | 0.981 | 0.892 | 0.994 | 2.8 | -6.8 |
| squareroot_glcm_MCC | 3.37 | 9.33 | 0.867 | 0.719 | 0.94 | 8.1 | -10.6 |
| squareroot_glcm_MaximumProbability | 11.43 | 31.68 | 0.961 | 0.867 | 0.986 | 45 | -18.4 |
| squareroot_glcm_SumAverage | 8.71 | 24.15 | 0.865 | 0.378 | 0.956 | 9.9 | -38.4 |
| squareroot_glcm_SumEntropy | 1.89 | 5.25 | 0.959 | 0.608 | 0.989 | 2.3 | -8.2 |
| squareroot_glcm_SumSquares | 18.05 | 50.03 | 0.813 | 0.452 | 0.928 | 18.6 | -81.5 |
| squareroot_gldm_DependenceEntropy | 1.31 | 3.64 | 0.949 | 0.281 | 0.988 | 0.9 | -6.3 |
| squareroot_gldm_DependenceNonUniformity | 12.00 | 33.27 | 0.986 | 0.968 | 0.994 | 22.7 | -43.8 |
| squareroot_gldm_DependenceNonUniformityNormalized | 7.89 | 21.86 | 0.91 | 0.806 | 0.96 | 19.9 | -23.8 |
| squareroot_gldm_DependenceVariance | 25.36 | 70.29 | 0.912 | 0.81 | 0.961 | 74.3 | -66.2 |
| squareroot_gldm_GrayLevelNonUniformity | 10.71 | 29.67 | 0.965 | 0.919 | 0.985 | 36.3 | -23 |
| squareroot_gldm_GrayLevelVariance | 17.88 | 49.57 | 0.809 | 0.45 | 0.926 | 18.7 | -80.5 |
| squareroot_gldm_HighGrayLevelEmphasis | 16.88 | 46.78 | 0.75 | 0.365 | 0.898 | 18.2 | -75.4 |
| squareroot_gldm_LargeDependenceEmphasis | 11.76 | 32.60 | 0.951 | 0.892 | 0.979 | 34.9 | -30.3 |
| squareroot_gldm_LargeDependenceHighGrayLevelEmphasis | 16.99 | 47.08 | 0.804 | 0.388 | 0.927 | 18.8 | -75.3 |
| squareroot_gldm_LargeDependenceLowGrayLevelEmphasis | 29.29 | 81.18 | 0.956 | 0.902 | 0.981 | 96 | -66.3 |
| squareroot_gldm_LowGrayLevelEmphasis | 25.65 | 71.08 | 0.851 | 0.69 | 0.933 | 87 | -55.2 |
| squareroot_gldm_SmallDependenceEmphasis | 5.01 | 13.89 | 0.954 | 0.897 | 0.98 | 13.4 | -14.3 |
| squareroot_gldm_SmallDependenceHighGrayLevelEmphasis | 23.00 | 63.76 | 0.732 | 0.359 | 0.888 | 35.5 | -92.1 |
| squareroot_gldm_SmallDependenceLowGrayLevelEmphasis | 25.37 | 70.32 | 0.765 | 0.534 | 0.89 | 87.3 | -53.3 |
| squareroot_glrlm_GrayLevelNonUniformity | 10.58 | 29.32 | 0.965 | 0.92 | 0.985 | 35.8 | -22.8 |
| squareroot_glrlm_GrayLevelNonUniformityNormalized | 9.84 | 27.27 | 0.966 | 0.881 | 0.988 | 42.3 | -12.2 |
| squareroot_glrlm_GrayLevelVariance | 18.00 | 49.89 | 0.809 | 0.45 | 0.926 | 19 | -80.8 |
| squareroot_glrlm_HighGrayLevelRunEmphasis | 16.98 | 47.07 | 0.751 | 0.366 | 0.898 | 18.5 | -75.6 |
| squareroot_glrlm_LongRunEmphasis | 0.93 | 2.57 | 0.963 | 0.917 | 0.984 | 2.7 | -2.4 |
| squareroot_glrlm_LongRunHighGrayLevelEmphasis | 16.27 | 45.10 | 0.754 | 0.367 | 0.9 | 16.6 | -73.6 |
| squareroot_glrlm_LongRunLowGrayLevelEmphasis | 25.81 | 71.55 | 0.871 | 0.728 | 0.942 | 87.4 | -55.7 |
| squareroot_glrlm_LowGrayLevelRunEmphasis | 25.71 | 71.26 | 0.853 | 0.694 | 0.933 | 87.3 | -55.3 |
| squareroot_glrlm_RunEntropy | 1.91 | 5.30 | 0.951 | 0.499 | 0.987 | 2.2 | -8.5 |
| squareroot_glrlm_RunLengthNonUniformity | 7.80 | 21.62 | 0.996 | 0.992 | 0.998 | 12.9 | -30.3 |
| squareroot_glrlm_RunLengthNonUniformityNormalized | 0.63 | 1.74 | 0.958 | 0.907 | 0.982 | 1.7 | -1.8 |
| squareroot_glrlm_RunPercentage | 0.31 | 0.87 | 0.962 | 0.915 | 0.983 | 0.8 | -0.9 |
| squareroot_glrlm_RunVariance | 21.26 | 58.93 | 0.967 | 0.925 | 0.985 | 63.9 | -54 |
| squareroot_glrlm_ShortRunEmphasis | 0.25 | 0.68 | 0.958 | 0.907 | 0.981 | 0.7 | -0.7 |
| squareroot_glrlm_ShortRunHighGrayLevelEmphasis | 17.20 | 47.69 | 0.75 | 0.366 | 0.898 | 19.2 | -76.2 |
| squareroot_glrlm_ShortRunLowGrayLevelEmphasis | 25.69 | 71.20 | 0.849 | 0.685 | 0.931 | 87.2 | -55.2 |
| squareroot_glszm_GrayLevelNonUniformity | 9.19 | 25.47 | 0.974 | 0.94 | 0.989 | 31.1 | -19.9 |
| squareroot_glszm_GrayLevelNonUniformityNormalized | 10.56 | 29.27 | 0.945 | 0.861 | 0.977 | 43.8 | -14.8 |
| squareroot_glszm_GrayLevelVariance | 19.13 | 53.03 | 0.809 | 0.455 | 0.926 | 22.2 | -83.8 |
| squareroot_glszm_HighGrayLevelZoneEmphasis | 18.73 | 51.93 | 0.752 | 0.373 | 0.898 | 23.6 | -80.3 |
| squareroot_glszm_LargeAreaEmphasis | 9.89 | 27.41 | 0.976 | 0.946 | 0.99 | 29.2 | -25.7 |
| squareroot_glszm_LargeAreaHighGrayLevelEmphasis | 15.49 | 42.93 | 0.79 | 0.386 | 0.92 | 15.6 | -70.3 |
| squareroot_glszm_LargeAreaLowGrayLevelEmphasis | 28.36 | 78.60 | 0.947 | 0.884 | 0.977 | 94.3 | -62.9 |
| squareroot_glszm_LowGrayLevelZoneEmphasis | 26.79 | 74.24 | 0.87 | 0.727 | 0.941 | 91.1 | -57.4 |
| squareroot_glszm_SizeZoneNonUniformity | 12.92 | 35.81 | 0.987 | 0.97 | 0.995 | 26.6 | -45 |
| squareroot_glszm_SizeZoneNonUniformityNormalized | 5.68 | 15.74 | 0.937 | 0.862 | 0.972 | 15.4 | -16.1 |
| squareroot_glszm_SmallAreaEmphasis | 2.70 | 7.48 | 0.949 | 0.887 | 0.977 | 7.4 | -7.5 |
| squareroot_glszm_SmallAreaHighGrayLevelEmphasis | 22.67 | 62.83 | 0.743 | 0.37 | 0.893 | 34.9 | -90.7 |
| squareroot_glszm_SmallAreaLowGrayLevelEmphasis | 26.74 | 74.11 | 0.843 | 0.674 | 0.928 | 91.7 | -56.5 |
| squareroot_glszm_ZoneEntropy | 1.71 | 4.75 | 0.942 | 0.426 | 0.985 | 1.9 | -7.6 |
| squareroot_glszm_ZonePercentage | 3.70 | 10.26 | 0.965 | 0.922 | 0.985 | 9.9 | -10.6 |
| squareroot_glszm_ZoneVariance | 29.13 | 80.73 | 0.974 | 0.941 | 0.988 | 86.1 | -75.4 |
| squareroot_ngtdm_Busyness | 15.17 | 42.06 | 0.926 | 0.837 | 0.967 | 64.6 | -19.5 |
| squareroot_ngtdm_Coarseness | 6.49 | 17.99 | 0.942 | 0.846 | 0.976 | 23.9 | -12 |
| squareroot_ngtdm_Complexity | 22.68 | 62.87 | 0.591 | 0.244 | 0.802 | 21 | -104.8 |
| squareroot_ngtdm_Contrast | 14.69 | 40.72 | 0.89 | 0.613 | 0.96 | 27 | -54.4 |
| squareroot_ngtdm_Strength | 16.09 | 44.61 | 0.878 | 0.441 | 0.96 | 19.3 | -69.9 |
| wavelet-LLH_firstorder_10Percentile | 5.91 | 16.38 | 0.987 | 0.972 | 0.995 | 19.7 | -13.1 |
| wavelet-LLH_firstorder_90Percentile | 8.43 | 23.36 | 0.977 | 0.949 | 0.99 | 22.4 | -24.3 |
| wavelet-LLH_firstorder_Energy | 14.87 | 41.22 | 0.993 | 0.984 | 0.997 | 34.4 | -48.1 |
| wavelet-LLH_firstorder_Entropy | 2.54 | 7.03 | 0.987 | 0.971 | 0.994 | 7.3 | -6.7 |
| wavelet-LLH_firstorder_InterquartileRange | 10.47 | 29.02 | 0.958 | 0.906 | 0.981 | 31.6 | -26.4 |
| wavelet-LLH_firstorder_Kurtosis | 13.92 | 38.58 | 0.596 | 0.271 | 0.801 | 32.1 | -45.1 |
| wavelet-LLH_firstorder_Maximum | 11.17 | 30.97 | 0.966 | 0.924 | 0.985 | 29.7 | -32.2 |
| wavelet-LLH_firstorder_MeanAbsoluteDeviation | 6.99 | 19.37 | 0.983 | 0.961 | 0.992 | 20.6 | -18.2 |
| wavelet-LLH_firstorder_Mean | 10.24 | 28.38 | 0.969 | 0.927 | 0.987 | 35 | -21.8 |
| wavelet-LLH_firstorder_Median | 13.99 | 38.77 | 0.876 | 0.707 | 0.947 | 50.3 | -27.3 |
| wavelet-LLH_firstorder_Minimum | 9.10 | 25.24 | 0.99 | 0.977 | 0.996 | 24.6 | -25.9 |
| wavelet-LLH_firstorder_Range | 9.87 | 27.36 | 0.987 | 0.97 | 0.994 | 26.4 | -28.4 |
| wavelet-LLH_firstorder_RobustMeanAbsoluteDeviation | 8.00 | 22.16 | 0.976 | 0.947 | 0.99 | 24.7 | -19.6 |
| wavelet-LLH_firstorder_RootMeanSquared | 7.32 | 20.30 | 0.985 | 0.966 | 0.993 | 21.1 | -19.5 |
| wavelet-LLH_firstorder_Skewness | 83.74 | 232.11 | 0.853 | 0.694 | 0.933 | 221.8 | -242.4 |
| wavelet-LLH_firstorder_TotalEnergy | 14.87 | 41.22 | 0.993 | 0.984 | 0.997 | 34.4 | -48.1 |
| wavelet-LLH_firstorder_Uniformity | 7.85 | 21.77 | 0.982 | 0.96 | 0.992 | 21 | -22.5 |
| wavelet-LLH_firstorder_Variance | 15.48 | 42.91 | 0.971 | 0.935 | 0.987 | 43.3 | -42.5 |
| wavelet-LLH_glcm_Autocorrelation | 18.23 | 50.53 | 0.99 | 0.977 | 0.996 | 47.9 | -53.1 |
| wavelet-LLH_glcm_ClusterProminence | 35.13 | 97.36 | 0.894 | 0.775 | 0.953 | 91.8 | -102.9 |
| wavelet-LLH_glcm_ClusterShade | 124.09 | 343.96 | 0.937 | 0.859 | 0.973 | 355.1 | -332.9 |
| wavelet-LLH_glcm_ClusterTendency | 15.46 | 42.85 | 0.971 | 0.935 | 0.987 | 42.3 | -43.4 |
| wavelet-LLH_glcm_Contrast | 14.31 | 39.66 | 0.971 | 0.934 | 0.987 | 42 | -37.3 |
| wavelet-LLH_glcm_Correlation | 1.70 | 4.72 | 0.942 | 0.866 | 0.975 | 3.8 | -5.6 |
| wavelet-LLH_glcm_DifferenceAverage | 7.18 | 19.89 | 0.986 | 0.968 | 0.994 | 21.8 | -18 |
| wavelet-LLH_glcm_DifferenceEntropy | 4.12 | 11.43 | 0.988 | 0.973 | 0.995 | 11.8 | -11.1 |
| wavelet-LLH_glcm_DifferenceVariance | 16.00 | 44.34 | 0.963 | 0.918 | 0.984 | 43.6 | -45.1 |
| wavelet-LLH_glcm_Id | 3.34 | 9.26 | 0.982 | 0.958 | 0.992 | 7.9 | -10.7 |
| wavelet-LLH_glcm_Idm | 4.70 | 13.02 | 0.98 | 0.954 | 0.991 | 10.7 | -15.3 |
| wavelet-LLH_glcm_Idmn | 0.24 | 0.66 | 0.91 | 0.806 | 0.96 | 0.6 | -0.7 |
| wavelet-LLH_glcm_Idn | 0.62 | 1.73 | 0.933 | 0.852 | 0.97 | 1.5 | -2 |
| wavelet-LLH_glcm_Imc1 | 4.32 | 11.98 | 0.966 | 0.924 | 0.985 | 13.9 | -10 |
| wavelet-LLH_glcm_Imc2 | 1.37 | 3.79 | 0.956 | 0.899 | 0.981 | 4.4 | -3.2 |
| wavelet-LLH_glcm_InverseVariance | 3.99 | 11.05 | 0.984 | 0.961 | 0.993 | 9.3 | -12.8 |
| wavelet-LLH_glcm_JointAverage | 9.61 | 26.64 | 0.989 | 0.975 | 0.995 | 25.1 | -28.2 |
| wavelet-LLH_glcm_JointEnergy | 12.22 | 33.87 | 0.973 | 0.939 | 0.988 | 32.4 | -35.3 |
| wavelet-LLH_glcm_JointEntropy | 2.56 | 7.10 | 0.989 | 0.974 | 0.995 | 6.9 | -7.3 |
| wavelet-LLH_glcm_MCC | 1.68 | 4.66 | 0.933 | 0.853 | 0.97 | 4.7 | -4.6 |
| wavelet-LLH_glcm_MaximumProbability | 19.89 | 55.14 | 0.902 | 0.79 | 0.956 | 50.4 | -59.9 |
| wavelet-LLH_glcm_SumAverage | 9.61 | 26.64 | 0.989 | 0.975 | 0.995 | 25.1 | -28.2 |
| wavelet-LLH_glcm_SumEntropy | 1.99 | 5.51 | 0.99 | 0.977 | 0.996 | 5.5 | -5.5 |
| wavelet-LLH_glcm_SumSquares | 15.23 | 42.21 | 0.971 | 0.935 | 0.987 | 42.1 | -42.4 |
| wavelet-LLH_gldm_DependenceEntropy | 1.04 | 2.87 | 0.985 | 0.955 | 0.994 | 2.1 | -3.7 |
| wavelet-LLH_gldm_DependenceNonUniformity | 9.78 | 27.10 | 0.996 | 0.991 | 0.998 | 23.1 | -31.1 |
| wavelet-LLH_gldm_DependenceNonUniformityNormalized | 6.73 | 18.65 | 0.953 | 0.871 | 0.981 | 23.2 | -14.1 |
| wavelet-LLH_gldm_DependenceVariance | 21.14 | 58.58 | 0.876 | 0.738 | 0.944 | 53.8 | -63.4 |
| wavelet-LLH_gldm_GrayLevelNonUniformity | 11.50 | 31.88 | 0.992 | 0.981 | 0.996 | 22.6 | -41.2 |
| wavelet-LLH_gldm_GrayLevelVariance | 15.43 | 42.76 | 0.971 | 0.935 | 0.987 | 43 | -42.5 |
| wavelet-LLH_gldm_HighGrayLevelEmphasis | 17.70 | 49.07 | 0.99 | 0.976 | 0.995 | 47.1 | -51 |
| wavelet-LLH_gldm_LargeDependenceEmphasis | 12.62 | 34.97 | 0.95 | 0.89 | 0.978 | 29 | -41 |
| wavelet-LLH_gldm_LargeDependenceHighGrayLevelEmphasis | 19.65 | 54.47 | 0.989 | 0.975 | 0.995 | 47.4 | -61.6 |
| wavelet-LLH_gldm_LargeDependenceLowGrayLevelEmphasis | 45.75 | 126.82 | 0.961 | 0.913 | 0.983 | 122.8 | -130.8 |
| wavelet-LLH_gldm_LowGrayLevelEmphasis | 28.74 | 79.67 | 0.936 | 0.859 | 0.972 | 80.8 | -78.5 |
| wavelet-LLH_gldm_SmallDependenceEmphasis | 13.11 | 36.33 | 0.974 | 0.942 | 0.989 | 40 | -32.6 |
| wavelet-LLH_gldm_SmallDependenceHighGrayLevelEmphasis | 27.98 | 77.56 | 0.981 | 0.957 | 0.992 | 77.2 | -77.9 |
| wavelet-LLH_gldm_SmallDependenceLowGrayLevelEmphasis | 17.11 | 47.43 | 0.921 | 0.829 | 0.965 | 54.5 | -40.4 |
| wavelet-LLH_glrlm_GrayLevelNonUniformity | 10.77 | 29.86 | 0.994 | 0.987 | 0.997 | 21.4 | -38.3 |
| wavelet-LLH_glrlm_GrayLevelNonUniformityNormalized | 7.38 | 20.45 | 0.985 | 0.965 | 0.993 | 20 | -20.9 |
| wavelet-LLH_glrlm_GrayLevelVariance | 16.31 | 45.20 | 0.973 | 0.94 | 0.988 | 44.7 | -45.7 |
| wavelet-LLH_glrlm_HighGrayLevelRunEmphasis | 17.83 | 49.43 | 0.989 | 0.976 | 0.995 | 47.4 | -51.5 |
| wavelet-LLH_glrlm_LongRunEmphasis | 4.49 | 12.44 | 0.972 | 0.936 | 0.988 | 10.4 | -14.4 |
| wavelet-LLH_glrlm_LongRunHighGrayLevelEmphasis | 16.80 | 46.56 | 0.989 | 0.974 | 0.995 | 42.5 | -50.6 |
| wavelet-LLH_glrlm_LongRunLowGrayLevelEmphasis | 31.07 | 86.12 | 0.951 | 0.893 | 0.979 | 85.9 | -86.4 |
| wavelet-LLH_glrlm_LowGrayLevelRunEmphasis | 27.32 | 75.73 | 0.933 | 0.853 | 0.97 | 76.8 | -74.6 |
| wavelet-LLH_glrlm_RunEntropy | 1.65 | 4.58 | 0.987 | 0.969 | 0.994 | 4.1 | -5 |
| wavelet-LLH_glrlm_RunLengthNonUniformity | 8.55 | 23.71 | 0.996 | 0.991 | 0.998 | 16.6 | -30.8 |
| wavelet-LLH_glrlm_RunLengthNonUniformityNormalized | 2.26 | 6.26 | 0.972 | 0.938 | 0.988 | 7 | -5.5 |
| wavelet-LLH_glrlm_RunPercentage | 1.40 | 3.89 | 0.967 | 0.925 | 0.986 | 4.5 | -3.3 |
| wavelet-LLH_glrlm_RunVariance | 14.97 | 41.50 | 0.969 | 0.924 | 0.987 | 32.1 | -50.9 |
| wavelet-LLH_glrlm_ShortRunEmphasis | 1.20 | 3.33 | 0.968 | 0.928 | 0.986 | 3.6 | -3.1 |
| wavelet-LLH_glrlm_ShortRunHighGrayLevelEmphasis | 18.39 | 50.97 | 0.989 | 0.976 | 0.995 | 49.1 | -52.9 |
| wavelet-LLH_glrlm_ShortRunLowGrayLevelEmphasis | 26.01 | 72.08 | 0.926 | 0.838 | 0.967 | 73.5 | -70.6 |
| wavelet-LLH_glszm_GrayLevelNonUniformity | 14.46 | 40.09 | 0.996 | 0.991 | 0.998 | 37.1 | -43.1 |
| wavelet-LLH_glszm_GrayLevelNonUniformityNormalized | 13.89 | 38.51 | 0.915 | 0.817 | 0.962 | 37.2 | -39.9 |
| wavelet-LLH_glszm_GrayLevelVariance | 19.86 | 55.04 | 0.98 | 0.953 | 0.991 | 52.6 | -57.5 |
| wavelet-LLH_glszm_HighGrayLevelZoneEmphasis | 18.60 | 51.55 | 0.988 | 0.972 | 0.995 | 46.6 | -56.5 |
| wavelet-LLH_glszm_LargeAreaEmphasis | 29.31 | 81.23 | 0.925 | 0.831 | 0.967 | 62 | -100.5 |
| wavelet-LLH_glszm_LargeAreaHighGrayLevelEmphasis | 30.62 | 84.87 | 0.969 | 0.93 | 0.986 | 63.9 | -105.8 |
| wavelet-LLH_glszm_LargeAreaLowGrayLevelEmphasis | 42.83 | 118.71 | 0.941 | 0.87 | 0.974 | 104.2 | -133.2 |
| wavelet-LLH_glszm_LowGrayLevelZoneEmphasis | 18.31 | 50.77 | 0.939 | 0.867 | 0.973 | 52.5 | -49 |
| wavelet-LLH_glszm_SizeZoneNonUniformity | 24.59 | 68.17 | 0.993 | 0.983 | 0.997 | 61.7 | -74.6 |
| wavelet-LLH_glszm_SizeZoneNonUniformityNormalized | 14.02 | 38.85 | 0.936 | 0.859 | 0.972 | 34.5 | -43.2 |
| wavelet-LLH_glszm_SmallAreaEmphasis | 12.90 | 35.76 | 0.911 | 0.808 | 0.961 | 30.9 | -40.7 |
| wavelet-LLH_glszm_SmallAreaHighGrayLevelEmphasis | 27.36 | 75.83 | 0.979 | 0.952 | 0.991 | 65.3 | -86.3 |
| wavelet-LLH_glszm_SmallAreaLowGrayLevelEmphasis | 32.20 | 89.24 | 0.606 | 0.282 | 0.807 | 92.6 | -85.9 |
| wavelet-LLH_glszm_ZoneEntropy | 4.19 | 11.62 | 0.968 | 0.928 | 0.986 | 12.3 | -11 |
| wavelet-LLH_glszm_ZonePercentage | 15.46 | 42.84 | 0.977 | 0.945 | 0.99 | 49.4 | -36.3 |
| wavelet-LLH_glszm_ZoneVariance | 32.91 | 91.21 | 0.892 | 0.768 | 0.952 | 66.6 | -115.8 |
| wavelet-LLH_ngtdm_Busyness | 23.22 | 64.37 | 0.829 | 0.648 | 0.922 | 63.8 | -64.9 |
| wavelet-LLH_ngtdm_Coarseness | 10.55 | 29.25 | 0.919 | 0.821 | 0.964 | 35 | -23.5 |
| wavelet-LLH_ngtdm_Complexity | 23.67 | 65.60 | 0.954 | 0.898 | 0.98 | 64.3 | -66.9 |
| wavelet-LLH_ngtdm_Contrast | 14.31 | 39.65 | 0.949 | 0.882 | 0.978 | 46.5 | -32.8 |
| wavelet-LLH_ngtdm_Strength | 26.81 | 74.31 | 0.974 | 0.942 | 0.989 | 75.9 | -72.7 |
| wavelet-LHL_firstorder_10Percentile | 6.46 | 17.92 | 0.987 | 0.972 | 0.995 | 17.1 | -18.7 |
| wavelet-LHL_firstorder_90Percentile | 19.21 | 53.25 | 0.969 | 0.931 | 0.987 | 46.4 | -60.1 |
| wavelet-LHL_firstorder_Energy | 17.64 | 48.90 | 0.977 | 0.948 | 0.99 | 38.9 | -58.9 |
| wavelet-LHL_firstorder_Entropy | 4.95 | 13.72 | 0.99 | 0.976 | 0.995 | 13.2 | -14.2 |
| wavelet-LHL_firstorder_InterquartileRange | 9.26 | 25.66 | 0.979 | 0.953 | 0.991 | 26.6 | -24.7 |
| wavelet-LHL_firstorder_Kurtosis | 13.33 | 36.94 | 0.854 | 0.693 | 0.934 | 32.4 | -41.5 |
| wavelet-LHL_firstorder_Maximum | 15.45 | 42.82 | 0.967 | 0.927 | 0.986 | 43.7 | -41.9 |
| wavelet-LHL_firstorder_MeanAbsoluteDeviation | 8.53 | 23.65 | 0.982 | 0.96 | 0.992 | 22.8 | -24.5 |
| wavelet-LHL_firstorder_Mean | 8.06 | 22.35 | 0.975 | 0.945 | 0.989 | 24.2 | -20.5 |
| wavelet-LHL_firstorder_Median | 50.44 | 139.82 | 0.597 | 0.269 | 0.802 | 125.2 | -154.4 |
| wavelet-LHL_firstorder_Minimum | 14.53 | 40.28 | 0.934 | 0.856 | 0.971 | 35.9 | -44.7 |
| wavelet-LHL_firstorder_Range | 12.69 | 35.18 | 0.971 | 0.936 | 0.987 | 32.9 | -37.5 |
| wavelet-LHL_firstorder_RobustMeanAbsoluteDeviation | 8.77 | 24.32 | 0.982 | 0.959 | 0.992 | 24.3 | -24.3 |
| wavelet-LHL_firstorder_RootMeanSquared | 7.79 | 21.59 | 0.984 | 0.963 | 0.993 | 20.9 | -22.3 |
| wavelet-LHL_firstorder_Skewness | 32.77 | 90.83 | 0.799 | 0.595 | 0.907 | 84 | -97.6 |
| wavelet-LHL_firstorder_TotalEnergy | 17.64 | 48.90 | 0.977 | 0.948 | 0.99 | 38.9 | -58.9 |
| wavelet-LHL_firstorder_Uniformity | 7.78 | 21.57 | 0.975 | 0.943 | 0.989 | 22.6 | -20.5 |
| wavelet-LHL_firstorder_Variance | 17.65 | 48.92 | 0.959 | 0.909 | 0.982 | 45.9 | -52 |
| wavelet-LHL_glcm_Autocorrelation | 31.07 | 86.13 | 0.801 | 0.597 | 0.908 | 79 | -93.3 |
| wavelet-LHL_glcm_ClusterProminence | 36.94 | 102.38 | 0.856 | 0.699 | 0.935 | 91.9 | -112.8 |
| wavelet-LHL_glcm_ClusterShade | 156.12 | 432.75 | 0.82 | 0.631 | 0.917 | 454.3 | -411.1 |
| wavelet-LHL_glcm_ClusterTendency | 17.79 | 49.31 | 0.962 | 0.916 | 0.983 | 46 | -52.6 |
| wavelet-LHL_glcm_Contrast | 18.14 | 50.27 | 0.913 | 0.812 | 0.961 | 48.9 | -51.7 |
| wavelet-LHL_glcm_Correlation | 4.01 | 11.10 | 0.855 | 0.698 | 0.934 | 9.8 | -12.4 |
| wavelet-LHL_glcm_DifferenceAverage | 9.71 | 26.91 | 0.969 | 0.931 | 0.987 | 27.4 | -26.5 |
| wavelet-LHL_glcm_DifferenceEntropy | 4.96 | 13.76 | 0.988 | 0.973 | 0.995 | 13 | -14.5 |
| wavelet-LHL_glcm_DifferenceVariance | 17.78 | 49.27 | 0.924 | 0.836 | 0.966 | 44.2 | -54.3 |
| wavelet-LHL_glcm_Id | 4.25 | 11.78 | 0.987 | 0.972 | 0.995 | 10.8 | -12.7 |
| wavelet-LHL_glcm_Idm | 5.78 | 16.03 | 0.987 | 0.972 | 0.995 | 14.6 | -17.4 |
| wavelet-LHL_glcm_Idmn | 0.35 | 0.98 | 0.828 | 0.646 | 0.921 | 1.1 | -0.9 |
| wavelet-LHL_glcm_Idn | 0.58 | 1.61 | 0.946 | 0.881 | 0.976 | 1.6 | -1.6 |
| wavelet-LHL_glcm_Imc1 | 6.84 | 18.97 | 0.912 | 0.809 | 0.961 | 19.4 | -18.5 |
| wavelet-LHL_glcm_Imc2 | 3.40 | 9.42 | 0.932 | 0.852 | 0.97 | 8.9 | -9.9 |
| wavelet-LHL_glcm_InverseVariance | 5.59 | 15.48 | 0.973 | 0.94 | 0.988 | 15.4 | -15.6 |
| wavelet-LHL_glcm_JointAverage | 16.68 | 46.22 | 0.925 | 0.837 | 0.967 | 42.6 | -49.9 |
| wavelet-LHL_glcm_JointEnergy | 13.50 | 37.42 | 0.955 | 0.9 | 0.98 | 40.8 | -34 |
| wavelet-LHL_glcm_JointEntropy | 4.81 | 13.32 | 0.989 | 0.976 | 0.995 | 12.3 | -14.3 |
| wavelet-LHL_glcm_MCC | 3.53 | 9.80 | 0.862 | 0.712 | 0.938 | 9.1 | -10.5 |
| wavelet-LHL_glcm_MaximumProbability | 12.32 | 34.16 | 0.974 | 0.942 | 0.989 | 38.5 | -29.8 |
| wavelet-LHL_glcm_SumAverage | 16.68 | 46.22 | 0.925 | 0.837 | 0.967 | 42.6 | -49.9 |
| wavelet-LHL_glcm_SumEntropy | 4.08 | 11.32 | 0.989 | 0.974 | 0.995 | 10.3 | -12.3 |
| wavelet-LHL_glcm_SumSquares | 17.39 | 48.21 | 0.956 | 0.903 | 0.981 | 45.3 | -51.1 |
| wavelet-LHL_gldm_DependenceEntropy | 1.46 | 4.06 | 0.979 | 0.934 | 0.992 | 2.9 | -5.2 |
| wavelet-LHL_gldm_DependenceNonUniformity | 11.35 | 31.45 | 0.993 | 0.985 | 0.997 | 25.4 | -37.5 |
| wavelet-LHL_gldm_DependenceNonUniformityNormalized | 8.93 | 24.76 | 0.958 | 0.906 | 0.981 | 27.3 | -22.2 |
| wavelet-LHL_gldm_DependenceVariance | 20.56 | 56.98 | 0.964 | 0.92 | 0.984 | 57.3 | -56.7 |
| wavelet-LHL_gldm_GrayLevelNonUniformity | 9.42 | 26.12 | 0.998 | 0.995 | 0.999 | 18.6 | -33.7 |
| wavelet-LHL_gldm_GrayLevelVariance | 16.98 | 47.06 | 0.959 | 0.909 | 0.982 | 44.7 | -49.4 |
| wavelet-LHL_gldm_HighGrayLevelEmphasis | 30.33 | 84.08 | 0.807 | 0.608 | 0.911 | 77 | -91.2 |
| wavelet-LHL_gldm_LargeDependenceEmphasis | 13.19 | 36.55 | 0.983 | 0.963 | 0.993 | 31.8 | -41.3 |
| wavelet-LHL_gldm_LargeDependenceHighGrayLevelEmphasis | 27.08 | 75.06 | 0.816 | 0.624 | 0.916 | 64.8 | -85.3 |
| wavelet-LHL_gldm_LargeDependenceLowGrayLevelEmphasis | 54.16 | 150.13 | 0.846 | 0.68 | 0.93 | 140.3 | -160 |
| wavelet-LHL_gldm_LowGrayLevelEmphasis | 35.49 | 98.38 | 0.8 | 0.595 | 0.908 | 104.1 | -92.7 |
| wavelet-LHL_gldm_SmallDependenceEmphasis | 14.10 | 39.09 | 0.986 | 0.948 | 0.995 | 48.2 | -30 |
| wavelet-LHL_gldm_SmallDependenceHighGrayLevelEmphasis | 37.92 | 105.11 | 0.797 | 0.59 | 0.906 | 107.3 | -102.9 |
| wavelet-LHL_gldm_SmallDependenceLowGrayLevelEmphasis | 20.67 | 57.29 | 0.815 | 0.622 | 0.915 | 71.4 | -43.2 |
| wavelet-LHL_glrlm_GrayLevelNonUniformity | 8.65 | 23.96 | 0.997 | 0.994 | 0.999 | 16.5 | -31.4 |
| wavelet-LHL_glrlm_GrayLevelNonUniformityNormalized | 7.97 | 22.11 | 0.977 | 0.949 | 0.99 | 22.5 | -21.7 |
| wavelet-LHL_glrlm_GrayLevelVariance | 17.98 | 49.85 | 0.96 | 0.911 | 0.982 | 48.1 | -51.6 |
| wavelet-LHL_glrlm_HighGrayLevelRunEmphasis | 30.27 | 83.92 | 0.807 | 0.608 | 0.911 | 77 | -90.9 |
| wavelet-LHL_glrlm_LongRunEmphasis | 5.48 | 15.20 | 0.989 | 0.975 | 0.995 | 14.2 | -16.2 |
| wavelet-LHL_glrlm_LongRunHighGrayLevelEmphasis | 27.71 | 76.81 | 0.817 | 0.625 | 0.916 | 68.8 | -84.8 |
| wavelet-LHL_glrlm_LongRunLowGrayLevelEmphasis | 39.54 | 109.61 | 0.872 | 0.729 | 0.942 | 113.4 | -105.8 |
| wavelet-LHL_glrlm_LowGrayLevelRunEmphasis | 34.05 | 94.39 | 0.817 | 0.626 | 0.916 | 100.8 | -88 |
| wavelet-LHL_glrlm_RunEntropy | 2.24 | 6.21 | 0.985 | 0.966 | 0.993 | 5.4 | -7 |
| wavelet-LHL_glrlm_RunLengthNonUniformity | 10.07 | 27.91 | 0.996 | 0.991 | 0.998 | 21.5 | -34.3 |
| wavelet-LHL_glrlm_RunLengthNonUniformityNormalized | 4.08 | 11.32 | 0.981 | 0.956 | 0.992 | 12.7 | -9.9 |
| wavelet-LHL_glrlm_RunPercentage | 2.28 | 6.31 | 0.984 | 0.964 | 0.993 | 7.1 | -5.6 |
| wavelet-LHL_glrlm_RunVariance | 9.62 | 26.66 | 0.989 | 0.976 | 0.995 | 24.4 | -28.9 |
| wavelet-LHL_glrlm_ShortRunEmphasis | 2.36 | 6.54 | 0.979 | 0.953 | 0.991 | 7.2 | -5.9 |
| wavelet-LHL_glrlm_ShortRunHighGrayLevelEmphasis | 31.48 | 87.26 | 0.807 | 0.608 | 0.911 | 81 | -93.5 |
| wavelet-LHL_glrlm_ShortRunLowGrayLevelEmphasis | 31.26 | 86.65 | 0.817 | 0.626 | 0.916 | 94.2 | -79.1 |
| wavelet-LHL_glszm_GrayLevelNonUniformity | 16.89 | 46.83 | 0.989 | 0.976 | 0.995 | 53.2 | -40.5 |
| wavelet-LHL_glszm_GrayLevelNonUniformityNormalized | 11.05 | 30.62 | 0.973 | 0.939 | 0.988 | 33.8 | -27.5 |
| wavelet-LHL_glszm_GrayLevelVariance | 26.13 | 72.42 | 0.968 | 0.929 | 0.986 | 68.1 | -76.8 |
| wavelet-LHL_glszm_HighGrayLevelZoneEmphasis | 31.94 | 88.53 | 0.81 | 0.614 | 0.913 | 84.1 | -92.9 |
| wavelet-LHL_glszm_LargeAreaEmphasis | 31.50 | 87.31 | 0.925 | 0.836 | 0.967 | 69.6 | -105 |
| wavelet-LHL_glszm_LargeAreaHighGrayLevelEmphasis | 32.20 | 89.25 | 0.92 | 0.824 | 0.965 | 63.8 | -114.7 |
| wavelet-LHL_glszm_LargeAreaLowGrayLevelEmphasis | 51.73 | 143.38 | 0.563 | 0.221 | 0.783 | 127.7 | -159.1 |
| wavelet-LHL_glszm_LowGrayLevelZoneEmphasis | 24.81 | 68.78 | 0.79 | 0.577 | 0.903 | 72 | -65.6 |
| wavelet-LHL_glszm_SizeZoneNonUniformity | 19.23 | 53.31 | 0.976 | 0.945 | 0.989 | 58.1 | -48.5 |
| wavelet-LHL_glszm_SizeZoneNonUniformityNormalized | 13.89 | 38.49 | 0.939 | 0.867 | 0.973 | 40.1 | -36.9 |
| wavelet-LHL_glszm_SmallAreaEmphasis | 29.80 | 82.60 | 0.883 | 0.72 | 0.95 | 98 | -67.2 |
| wavelet-LHL_glszm_SmallAreaHighGrayLevelEmphasis | 41.22 | 114.27 | 0.814 | 0.62 | 0.914 | 119.9 | -108.6 |
| wavelet-LHL_glszm_SmallAreaLowGrayLevelEmphasis | 50.82 | 140.86 | 0.546 | 0.197 | 0.774 | 152.3 | -129.4 |
| wavelet-LHL_glszm_ZoneEntropy | 8.25 | 22.87 | 0.969 | 0.932 | 0.987 | 24.1 | -21.6 |
| wavelet-LHL_glszm_ZonePercentage | 19.38 | 53.72 | 0.989 | 0.967 | 0.995 | 65.1 | -42.3 |
| wavelet-LHL_glszm_ZoneVariance | 28.59 | 79.26 | 0.962 | 0.915 | 0.983 | 66.5 | -92 |
| wavelet-LHL_ngtdm_Busyness | 33.17 | 91.94 | 0.644 | 0.339 | 0.828 | 86.1 | -97.8 |
| wavelet-LHL_ngtdm_Coarseness | 10.10 | 28.00 | 0.874 | 0.725 | 0.944 | 35.3 | -20.7 |
| wavelet-LHL_ngtdm_Complexity | 33.03 | 91.57 | 0.95 | 0.889 | 0.978 | 91.3 | -91.8 |
| wavelet-LHL_ngtdm_Contrast | 21.27 | 58.95 | 0.885 | 0.755 | 0.948 | 56.8 | -61.1 |
| wavelet-LHL_ngtdm_Strength | 31.00 | 85.93 | 0.941 | 0.87 | 0.974 | 91 | -80.9 |
| wavelet-LHH_firstorder_10Percentile | 13.74 | 38.08 | 0.971 | 0.936 | 0.987 | 35.6 | -40.6 |
| wavelet-LHH_firstorder_90Percentile | 10.49 | 29.07 | 0.964 | 0.919 | 0.984 | 27.6 | -30.5 |
| wavelet-LHH_firstorder_Energy | 21.93 | 60.79 | 0.979 | 0.952 | 0.991 | 52.6 | -69 |
| wavelet-LHH_firstorder_Entropy | 3.71 | 10.28 | 0.972 | 0.937 | 0.988 | 9.2 | -11.4 |
| wavelet-LHH_firstorder_InterquartileRange | 9.49 | 26.32 | 0.981 | 0.957 | 0.992 | 24.4 | -28.2 |
| wavelet-LHH_firstorder_Kurtosis | 10.26 | 28.45 | 0.867 | 0.72 | 0.94 | 32.2 | -24.7 |
| wavelet-LHH_firstorder_Maximum | 14.71 | 40.78 | 0.944 | 0.877 | 0.975 | 42.1 | -39.5 |
| wavelet-LHH_firstorder_MeanAbsoluteDeviation | 9.86 | 27.33 | 0.978 | 0.951 | 0.99 | 26.7 | -28 |
| wavelet-LHH_firstorder_Mean | 23.53 | 65.21 | 0.958 | 0.887 | 0.983 | 78.6 | -51.8 |
| wavelet-LHH_firstorder_Median | 198.29 | 549.63 | 0.858 | 0.69 | 0.937 | 536 | -563.3 |
| wavelet-LHH_firstorder_Minimum | 16.22 | 44.95 | 0.971 | 0.935 | 0.987 | 42 | -47.9 |
| wavelet-LHH_firstorder_Range | 13.34 | 36.99 | 0.965 | 0.922 | 0.985 | 36.6 | -37.4 |
| wavelet-LHH_firstorder_RobustMeanAbsoluteDeviation | 9.72 | 26.94 | 0.979 | 0.953 | 0.991 | 25.8 | -28.1 |
| wavelet-LHH_firstorder_RootMeanSquared | 10.07 | 27.91 | 0.974 | 0.942 | 0.989 | 28 | -27.9 |
| wavelet-LHH_firstorder_Skewness | 31.12 | 86.27 | 0.761 | 0.527 | 0.889 | 101.2 | -71.4 |
| wavelet-LHH_firstorder_TotalEnergy | 21.93 | 60.79 | 0.979 | 0.952 | 0.991 | 52.6 | -69 |
| wavelet-LHH_firstorder_Uniformity | 2.97 | 8.25 | 0.963 | 0.918 | 0.984 | 8.9 | -7.6 |
| wavelet-LHH_firstorder_Variance | 20.34 | 56.37 | 0.943 | 0.874 | 0.975 | 55.9 | -56.9 |
| wavelet-LHH_glcm_Autocorrelation | 21.72 | 60.19 | 0.913 | 0.81 | 0.961 | 51 | -69.4 |
| wavelet-LHH_glcm_ClusterProminence | 21.76 | 60.31 | 0.881 | 0.748 | 0.947 | 56.2 | -64.5 |
| wavelet-LHH_glcm_ClusterShade | 99.50 | 275.81 | 0.908 | 0.801 | 0.959 | 271.9 | -279.7 |
| wavelet-LHH_glcm_ClusterTendency | 8.52 | 23.62 | 0.951 | 0.891 | 0.978 | 21.6 | -25.7 |
| wavelet-LHH_glcm_Contrast | 9.21 | 25.52 | 0.932 | 0.851 | 0.97 | 23.2 | -27.8 |
| wavelet-LHH_glcm_Correlation | 6.35 | 17.61 | 0.719 | 0.456 | 0.867 | 18.3 | -16.9 |
| wavelet-LHH_glcm_DifferenceAverage | 6.99 | 19.39 | 0.961 | 0.913 | 0.983 | 17.3 | -21.5 |
| wavelet-LHH_glcm_DifferenceEntropy | 4.77 | 13.21 | 0.941 | 0.87 | 0.974 | 11.6 | -14.8 |
| wavelet-LHH_glcm_DifferenceVariance | 8.13 | 22.53 | 0.911 | 0.808 | 0.96 | 20.6 | -24.4 |
| wavelet-LHH_glcm_Id | 1.23 | 3.41 | 0.967 | 0.926 | 0.986 | 3.7 | -3.1 |
| wavelet-LHH_glcm_Idm | 1.42 | 3.94 | 0.966 | 0.925 | 0.985 | 4.2 | -3.7 |
| wavelet-LHH_glcm_Idmn | 0.66 | 1.82 | 0.814 | 0.618 | 0.915 | 1.5 | -2.1 |
| wavelet-LHH_glcm_Idn | 0.84 | 2.34 | 0.694 | 0.417 | 0.854 | 2 | -2.6 |
| wavelet-LHH_glcm_Imc1 | 9.64 | 26.72 | 0.693 | 0.414 | 0.854 | 29.1 | -24.3 |
| wavelet-LHH_glcm_Imc2 | 6.03 | 16.71 | 0.851 | 0.689 | 0.932 | 17.1 | -16.3 |
| wavelet-LHH_glcm_InverseVariance | 5.88 | 16.29 | 0.947 | 0.883 | 0.977 | 14.2 | -18.4 |
| wavelet-LHH_glcm_JointAverage | 11.88 | 32.92 | 0.873 | 0.729 | 0.943 | 27.8 | -38 |
| wavelet-LHH_glcm_JointEnergy | 6.40 | 17.75 | 0.954 | 0.899 | 0.98 | 19.2 | -16.3 |
| wavelet-LHH_glcm_JointEntropy | 4.43 | 12.28 | 0.969 | 0.932 | 0.987 | 10.8 | -13.8 |
| wavelet-LHH_glcm_MCC | 6.32 | 17.52 | 0.751 | 0.51 | 0.884 | 17.9 | -17.2 |
| wavelet-LHH_glcm_MaximumProbability | 5.77 | 15.99 | 0.94 | 0.869 | 0.974 | 17.6 | -14.4 |
| wavelet-LHH_glcm_SumAverage | 11.88 | 32.92 | 0.873 | 0.729 | 0.943 | 27.8 | -38 |
| wavelet-LHH_glcm_SumEntropy | 3.76 | 10.41 | 0.971 | 0.935 | 0.987 | 9.2 | -11.6 |
| wavelet-LHH_glcm_SumSquares | 8.17 | 22.66 | 0.948 | 0.884 | 0.977 | 20.5 | -24.8 |
| wavelet-LHH_gldm_DependenceEntropy | 1.86 | 5.15 | 0.823 | 0.637 | 0.919 | 4.8 | -5.5 |
| wavelet-LHH_gldm_DependenceNonUniformity | 8.52 | 23.61 | 0.993 | 0.984 | 0.997 | 15.9 | -31.3 |
| wavelet-LHH_gldm_DependenceNonUniformityNormalized | 4.53 | 12.57 | 0.91 | 0.806 | 0.96 | 13.5 | -11.6 |
| wavelet-LHH_gldm_DependenceVariance | 6.67 | 18.48 | 0.943 | 0.875 | 0.975 | 18.5 | -18.4 |
| wavelet-LHH_gldm_GrayLevelNonUniformity | 7.57 | 20.97 | 0.997 | 0.993 | 0.999 | 13 | -29 |
| wavelet-LHH_gldm_GrayLevelVariance | 7.37 | 20.44 | 0.946 | 0.88 | 0.976 | 18.6 | -22.3 |
| wavelet-LHH_gldm_HighGrayLevelEmphasis | 21.47 | 59.51 | 0.913 | 0.809 | 0.961 | 50.1 | -69 |
| wavelet-LHH_gldm_LargeDependenceEmphasis | 4.28 | 11.85 | 0.975 | 0.944 | 0.989 | 11.5 | -12.2 |
| wavelet-LHH_gldm_LargeDependenceHighGrayLevelEmphasis | 21.28 | 58.99 | 0.906 | 0.797 | 0.958 | 49.1 | -68.8 |
| wavelet-LHH_gldm_LargeDependenceLowGrayLevelEmphasis | 29.14 | 80.76 | 0.826 | 0.643 | 0.92 | 92.3 | -69.2 |
| wavelet-LHH_gldm_LowGrayLevelEmphasis | 26.59 | 73.70 | 0.746 | 0.502 | 0.881 | 86 | -61.4 |
| wavelet-LHH_gldm_SmallDependenceEmphasis | 17.85 | 49.47 | 0.685 | 0.402 | 0.85 | 49.2 | -49.8 |
| wavelet-LHH_gldm_SmallDependenceHighGrayLevelEmphasis | 33.36 | 92.48 | 0.873 | 0.732 | 0.943 | 78.8 | -106.2 |
| wavelet-LHH_gldm_SmallDependenceLowGrayLevelEmphasis | 21.14 | 58.61 | 0.761 | 0.503 | 0.891 | 71.4 | -45.8 |
| wavelet-LHH_glrlm_GrayLevelNonUniformity | 7.73 | 21.42 | 0.997 | 0.992 | 0.999 | 13.6 | -29.3 |
| wavelet-LHH_glrlm_GrayLevelNonUniformityNormalized | 3.18 | 8.80 | 0.969 | 0.932 | 0.987 | 9.5 | -8.1 |
| wavelet-LHH_glrlm_GrayLevelVariance | 8.75 | 24.26 | 0.937 | 0.861 | 0.972 | 22 | -26.6 |
| wavelet-LHH_glrlm_HighGrayLevelRunEmphasis | 21.10 | 58.48 | 0.918 | 0.817 | 0.964 | 48.7 | -68.3 |
| wavelet-LHH_glrlm_LongRunEmphasis | 6.32 | 17.53 | 0.966 | 0.924 | 0.985 | 18 | -17.1 |
| wavelet-LHH_glrlm_LongRunHighGrayLevelEmphasis | 19.86 | 55.05 | 0.939 | 0.866 | 0.973 | 45.9 | -64.2 |
| wavelet-LHH_glrlm_LongRunLowGrayLevelEmphasis | 29.97 | 83.07 | 0.857 | 0.7 | 0.935 | 95 | -71.1 |
| wavelet-LHH_glrlm_LowGrayLevelRunEmphasis | 25.72 | 71.29 | 0.792 | 0.579 | 0.904 | 83.9 | -58.7 |
| wavelet-LHH_glrlm_RunEntropy | 1.93 | 5.35 | 0.965 | 0.918 | 0.985 | 4.3 | -6.3 |
| wavelet-LHH_glrlm_RunLengthNonUniformity | 9.74 | 27.00 | 0.986 | 0.968 | 0.994 | 18.7 | -35.3 |
| wavelet-LHH_glrlm_RunLengthNonUniformityNormalized | 3.87 | 10.71 | 0.975 | 0.945 | 0.989 | 11 | -10.4 |
| wavelet-LHH_glrlm_RunPercentage | 2.61 | 7.24 | 0.978 | 0.95 | 0.99 | 7.4 | -7.1 |
| wavelet-LHH_glrlm_RunVariance | 6.83 | 18.92 | 0.974 | 0.942 | 0.989 | 18.3 | -19.5 |
| wavelet-LHH_glrlm_ShortRunEmphasis | 2.85 | 7.91 | 0.969 | 0.931 | 0.987 | 8.2 | -7.6 |
| wavelet-LHH_glrlm_ShortRunHighGrayLevelEmphasis | 22.47 | 62.27 | 0.934 | 0.851 | 0.971 | 52 | -72.5 |
| wavelet-LHH_glrlm_ShortRunLowGrayLevelEmphasis | 23.84 | 66.09 | 0.77 | 0.527 | 0.895 | 79.7 | -52.4 |
| wavelet-LHH_glszm_GrayLevelNonUniformity | 28.13 | 77.96 | 0.925 | 0.836 | 0.967 | 80.8 | -75.2 |
| wavelet-LHH_glszm_GrayLevelNonUniformityNormalized | 13.63 | 37.77 | 0.871 | 0.728 | 0.942 | 42.5 | -33 |
| wavelet-LHH_glszm_GrayLevelVariance | 27.94 | 77.44 | 0.899 | 0.784 | 0.955 | 64.5 | -90.4 |
| wavelet-LHH_glszm_HighGrayLevelZoneEmphasis | 22.43 | 62.17 | 0.929 | 0.843 | 0.969 | 46.9 | -77.5 |
| wavelet-LHH_glszm_LargeAreaEmphasis | 37.14 | 102.94 | 0.962 | 0.915 | 0.983 | 87.7 | -118.2 |
| wavelet-LHH_glszm_LargeAreaHighGrayLevelEmphasis | 31.00 | 85.92 | 0.963 | 0.918 | 0.984 | 60.6 | -111.3 |
| wavelet-LHH_glszm_LargeAreaLowGrayLevelEmphasis | 54.44 | 150.90 | 0.93 | 0.847 | 0.969 | 144.6 | -157.2 |
| wavelet-LHH_glszm_LowGrayLevelZoneEmphasis | 18.99 | 52.65 | 0.737 | 0.458 | 0.88 | 65 | -40.3 |
| wavelet-LHH_glszm_SizeZoneNonUniformity | 21.87 | 60.62 | 0.857 | 0.701 | 0.935 | 58.4 | -62.9 |
| wavelet-LHH_glszm_SizeZoneNonUniformityNormalized | 25.39 | 70.38 | 0.499 | 0.134 | 0.746 | 69.9 | -70.9 |
| wavelet-LHH_glszm_SmallAreaEmphasis | 78.49 | 217.55 | 0.165 | -0.205 | 0.512 | 172.2 | -262.9 |
| wavelet-LHH_glszm_SmallAreaHighGrayLevelEmphasis | 80.24 | 222.41 | 0.261 | -0.146 | 0.594 | 170.1 | -274.7 |
| wavelet-LHH_glszm_SmallAreaLowGrayLevelEmphasis | 86.13 | 238.74 | 0.061 | -0.342 | 0.446 | 206.1 | -271.4 |
| wavelet-LHH_glszm_ZoneEntropy | 19.60 | 54.32 | 0.83 | 0.649 | 0.922 | 53.5 | -55.1 |
| wavelet-LHH_glszm_ZonePercentage | 30.40 | 84.27 | 0.773 | 0.548 | 0.895 | 90.5 | -78 |
| wavelet-LHH_glszm_ZoneVariance | 28.08 | 77.84 | 0.968 | 0.929 | 0.986 | 64.4 | -91.3 |
| wavelet-LHH_ngtdm_Busyness | 41.28 | 114.42 | 0.911 | 0.807 | 0.96 | 123.9 | -104.9 |
| wavelet-LHH_ngtdm_Coarseness | 10.25 | 28.41 | 0.924 | 0.811 | 0.968 | 36.9 | -19.9 |
| wavelet-LHH_ngtdm_Complexity | 33.51 | 92.89 | 0.894 | 0.773 | 0.952 | 77.1 | -108.7 |
| wavelet-LHH_ngtdm_Contrast | 29.07 | 80.57 | 0.839 | 0.661 | 0.927 | 93.8 | -67.3 |
| wavelet-LHH_ngtdm_Strength | 37.63 | 104.30 | 0.829 | 0.647 | 0.922 | 97.8 | -110.8 |
| wavelet-HLL_firstorder_10Percentile | 8.86 | 24.56 | 0.984 | 0.963 | 0.993 | 21.4 | -27.8 |
| wavelet-HLL_firstorder_90Percentile | 18.67 | 51.76 | 0.969 | 0.921 | 0.987 | 46 | -57.5 |
| wavelet-HLL_firstorder_Energy | 19.71 | 54.64 | 0.993 | 0.983 | 0.997 | 39.9 | -69.4 |
| wavelet-HLL_firstorder_Entropy | 3.93 | 10.90 | 0.981 | 0.956 | 0.992 | 9.5 | -12.2 |
| wavelet-HLL_firstorder_InterquartileRange | 9.22 | 25.56 | 0.976 | 0.946 | 0.99 | 26.5 | -24.6 |
| wavelet-HLL_firstorder_Kurtosis | 15.23 | 42.21 | 0.928 | 0.842 | 0.968 | 35.9 | -48.6 |
| wavelet-HLL_firstorder_Maximum | 24.88 | 68.97 | 0.908 | 0.792 | 0.96 | 53.9 | -84.1 |
| wavelet-HLL_firstorder_MeanAbsoluteDeviation | 9.02 | 25.00 | 0.99 | 0.974 | 0.996 | 21.1 | -28.9 |
| wavelet-HLL_firstorder_Mean | 25.55 | 70.82 | 0.963 | 0.917 | 0.984 | 82.7 | -59 |
| wavelet-HLL_firstorder_Median | 41.70 | 115.59 | 0.728 | 0.461 | 0.873 | 122.2 | -109 |
| wavelet-HLL_firstorder_Minimum | 12.08 | 33.49 | 0.981 | 0.958 | 0.992 | 28.9 | -38 |
| wavelet-HLL_firstorder_Range | 13.10 | 36.31 | 0.979 | 0.944 | 0.991 | 28.6 | -44.1 |
| wavelet-HLL_firstorder_RobustMeanAbsoluteDeviation | 8.13 | 22.53 | 0.989 | 0.975 | 0.995 | 20.9 | -24.2 |
| wavelet-HLL_firstorder_RootMeanSquared | 10.54 | 29.23 | 0.986 | 0.968 | 0.994 | 25.9 | -32.6 |
| wavelet-HLL_firstorder_Skewness | 138.37 | 383.55 | 0.9 | 0.785 | 0.955 | 411.5 | -355.6 |
| wavelet-HLL_firstorder_TotalEnergy | 19.71 | 54.64 | 0.993 | 0.983 | 0.997 | 39.9 | -69.4 |
| wavelet-HLL_firstorder_Uniformity | 9.79 | 27.14 | 0.966 | 0.924 | 0.985 | 27.1 | -27.1 |
| wavelet-HLL_firstorder_Variance | 20.60 | 57.09 | 0.992 | 0.976 | 0.997 | 47.1 | -67.1 |
| wavelet-HLL_glcm_Autocorrelation | 25.27 | 70.06 | 0.973 | 0.94 | 0.988 | 59.2 | -80.9 |
| wavelet-HLL_glcm_ClusterProminence | 40.92 | 113.41 | 0.991 | 0.978 | 0.996 | 92.3 | -134.6 |
| wavelet-HLL_glcm_ClusterShade | 177.92 | 493.16 | 0.924 | 0.835 | 0.966 | 494.8 | -491.5 |
| wavelet-HLL_glcm_ClusterTendency | 21.58 | 59.82 | 0.991 | 0.975 | 0.997 | 48.9 | -70.7 |
| wavelet-HLL_glcm_Contrast | 21.30 | 59.04 | 0.985 | 0.963 | 0.993 | 50.7 | -67.3 |
| wavelet-HLL_glcm_Correlation | 2.62 | 7.26 | 0.951 | 0.887 | 0.979 | 5.8 | -8.7 |
| wavelet-HLL_glcm_DifferenceAverage | 10.19 | 28.24 | 0.983 | 0.959 | 0.993 | 23.7 | -32.7 |
| wavelet-HLL_glcm_DifferenceEntropy | 5.93 | 16.44 | 0.973 | 0.939 | 0.988 | 14.8 | -18.1 |
| wavelet-HLL_glcm_DifferenceVariance | 23.95 | 66.39 | 0.976 | 0.946 | 0.99 | 59.1 | -73.7 |
| wavelet-HLL_glcm_Id | 4.28 | 11.85 | 0.983 | 0.961 | 0.993 | 12.9 | -10.8 |
| wavelet-HLL_glcm_Idm | 6.39 | 17.71 | 0.982 | 0.96 | 0.992 | 18.8 | -16.7 |
| wavelet-HLL_glcm_Idmn | 0.30 | 0.83 | 0.922 | 0.831 | 0.966 | 0.8 | -0.9 |
| wavelet-HLL_glcm_Idn | 0.57 | 1.58 | 0.961 | 0.914 | 0.983 | 1.4 | -1.8 |
| wavelet-HLL_glcm_Imc1 | 4.48 | 12.42 | 0.973 | 0.941 | 0.988 | 12.2 | -12.6 |
| wavelet-HLL_glcm_Imc2 | 2.01 | 5.57 | 0.939 | 0.865 | 0.973 | 5.3 | -5.8 |
| wavelet-HLL_glcm_InverseVariance | 7.12 | 19.74 | 0.958 | 0.906 | 0.981 | 22.2 | -17.3 |
| wavelet-HLL_glcm_JointAverage | 13.40 | 37.13 | 0.979 | 0.951 | 0.991 | 31.5 | -42.7 |
| wavelet-HLL_glcm_JointEnergy | 16.50 | 45.73 | 0.941 | 0.87 | 0.974 | 45 | -46.5 |
| wavelet-HLL_glcm_JointEntropy | 4.09 | 11.34 | 0.978 | 0.95 | 0.99 | 9.8 | -12.9 |
| wavelet-HLL_glcm_MCC | 2.70 | 7.48 | 0.821 | 0.634 | 0.918 | 7.3 | -7.6 |
| wavelet-HLL_glcm_MaximumProbability | 21.09 | 58.45 | 0.97 | 0.932 | 0.987 | 50.5 | -66.4 |
| wavelet-HLL_glcm_SumAverage | 13.40 | 37.13 | 0.979 | 0.951 | 0.991 | 31.5 | -42.7 |
| wavelet-HLL_glcm_SumEntropy | 3.51 | 9.74 | 0.978 | 0.945 | 0.991 | 7.8 | -11.6 |
| wavelet-HLL_glcm_SumSquares | 21.41 | 59.35 | 0.991 | 0.974 | 0.996 | 48.9 | -69.8 |
| wavelet-HLL_gldm_DependenceEntropy | 1.90 | 5.27 | 0.965 | 0.918 | 0.985 | 4.3 | -6.2 |
| wavelet-HLL_gldm_DependenceNonUniformity | 9.74 | 27.01 | 0.996 | 0.991 | 0.998 | 20.7 | -33.3 |
| wavelet-HLL_gldm_DependenceNonUniformityNormalized | 8.41 | 23.30 | 0.919 | 0.824 | 0.964 | 25.6 | -21 |
| wavelet-HLL_gldm_DependenceVariance | 24.31 | 67.39 | 0.948 | 0.886 | 0.977 | 55.8 | -79 |
| wavelet-HLL_gldm_GrayLevelNonUniformity | 13.16 | 36.48 | 0.994 | 0.987 | 0.997 | 28 | -45 |
| wavelet-HLL_gldm_GrayLevelVariance | 20.44 | 56.67 | 0.991 | 0.975 | 0.997 | 47.2 | -66.1 |
| wavelet-HLL_gldm_HighGrayLevelEmphasis | 24.62 | 68.25 | 0.974 | 0.942 | 0.989 | 57.7 | -78.8 |
| wavelet-HLL_gldm_LargeDependenceEmphasis | 16.23 | 44.98 | 0.977 | 0.949 | 0.99 | 40.8 | -49.2 |
| wavelet-HLL_gldm_LargeDependenceHighGrayLevelEmphasis | 28.90 | 80.12 | 0.986 | 0.969 | 0.994 | 66.1 | -94.1 |
| wavelet-HLL_gldm_LargeDependenceLowGrayLevelEmphasis | 45.44 | 125.97 | 0.923 | 0.833 | 0.966 | 139.5 | -112.4 |
| wavelet-HLL_gldm_LowGrayLevelEmphasis | 30.42 | 84.33 | 0.855 | 0.698 | 0.934 | 98.4 | -70.3 |
| wavelet-HLL_gldm_SmallDependenceEmphasis | 12.47 | 34.56 | 0.962 | 0.916 | 0.983 | 32.2 | -36.9 |
| wavelet-HLL_gldm_SmallDependenceHighGrayLevelEmphasis | 32.56 | 90.25 | 0.994 | 0.986 | 0.997 | 75.9 | -104.6 |
| wavelet-HLL_gldm_SmallDependenceLowGrayLevelEmphasis | 16.92 | 46.91 | 0.858 | 0.704 | 0.936 | 48.8 | -45 |
| wavelet-HLL_glrlm_GrayLevelNonUniformity | 11.98 | 33.20 | 0.995 | 0.989 | 0.998 | 25.4 | -41 |
| wavelet-HLL_glrlm_GrayLevelNonUniformityNormalized | 9.43 | 26.13 | 0.965 | 0.923 | 0.985 | 27.3 | -24.9 |
| wavelet-HLL_glrlm_GrayLevelVariance | 21.23 | 58.84 | 0.99 | 0.971 | 0.996 | 48.9 | -68.8 |
| wavelet-HLL_glrlm_HighGrayLevelRunEmphasis | 24.58 | 68.12 | 0.975 | 0.943 | 0.989 | 57.5 | -78.7 |
| wavelet-HLL_glrlm_LongRunEmphasis | 5.61 | 15.55 | 0.96 | 0.912 | 0.983 | 14.9 | -16.2 |
| wavelet-HLL_glrlm_LongRunHighGrayLevelEmphasis | 23.57 | 65.34 | 0.973 | 0.939 | 0.988 | 54.4 | -76.3 |
| wavelet-HLL_glrlm_LongRunLowGrayLevelEmphasis | 33.84 | 93.80 | 0.924 | 0.835 | 0.966 | 108.4 | -79.2 |
| wavelet-HLL_glrlm_LowGrayLevelRunEmphasis | 28.89 | 80.07 | 0.873 | 0.733 | 0.943 | 93.6 | -66.5 |
| wavelet-HLL_glrlm_RunEntropy | 2.38 | 6.60 | 0.975 | 0.942 | 0.989 | 5.6 | -7.6 |
| wavelet-HLL_glrlm_RunLengthNonUniformity | 8.64 | 23.96 | 0.997 | 0.993 | 0.999 | 14.4 | -33.5 |
| wavelet-HLL_glrlm_RunLengthNonUniformityNormalized | 2.14 | 5.93 | 0.985 | 0.966 | 0.993 | 5.3 | -6.6 |
| wavelet-HLL_glrlm_RunPercentage | 1.52 | 4.22 | 0.982 | 0.958 | 0.992 | 4 | -4.5 |
| wavelet-HLL_glrlm_RunVariance | 21.00 | 58.21 | 0.962 | 0.915 | 0.983 | 49.1 | -67.3 |
| wavelet-HLL_glrlm_ShortRunEmphasis | 1.26 | 3.50 | 0.981 | 0.958 | 0.992 | 3.1 | -3.9 |
| wavelet-HLL_glrlm_ShortRunHighGrayLevelEmphasis | 25.05 | 69.44 | 0.975 | 0.944 | 0.989 | 58.6 | -80.3 |
| wavelet-HLL_glrlm_ShortRunLowGrayLevelEmphasis | 27.10 | 75.13 | 0.854 | 0.696 | 0.934 | 88.1 | -62.1 |
| wavelet-HLL_glszm_GrayLevelNonUniformity | 14.47 | 40.10 | 0.995 | 0.988 | 0.998 | 30.3 | -49.9 |
| wavelet-HLL_glszm_GrayLevelNonUniformityNormalized | 15.71 | 43.55 | 0.875 | 0.736 | 0.944 | 46.2 | -40.9 |
| wavelet-HLL_glszm_GrayLevelVariance | 27.49 | 76.21 | 0.978 | 0.947 | 0.99 | 67.6 | -84.8 |
| wavelet-HLL_glszm_HighGrayLevelZoneEmphasis | 24.51 | 67.94 | 0.981 | 0.957 | 0.992 | 57.5 | -78.4 |
| wavelet-HLL_glszm_LargeAreaEmphasis | 34.46 | 95.51 | 0.864 | 0.715 | 0.939 | 90.9 | -100.2 |
| wavelet-HLL_glszm_LargeAreaHighGrayLevelEmphasis | 33.62 | 93.19 | 0.995 | 0.989 | 0.998 | 77.8 | -108.6 |
| wavelet-HLL_glszm_LargeAreaLowGrayLevelEmphasis | 46.66 | 129.34 | 0.815 | 0.623 | 0.915 | 139.4 | -119.3 |
| wavelet-HLL_glszm_LowGrayLevelZoneEmphasis | 19.03 | 52.75 | 0.948 | 0.885 | 0.977 | 63.6 | -41.9 |
| wavelet-HLL_glszm_SizeZoneNonUniformity | 24.99 | 69.28 | 0.986 | 0.969 | 0.994 | 57.6 | -80.9 |
| wavelet-HLL_glszm_SizeZoneNonUniformityNormalized | 13.61 | 37.73 | 0.944 | 0.878 | 0.975 | 38.2 | -37.3 |
| wavelet-HLL_glszm_SmallAreaEmphasis | 12.62 | 34.97 | 0.938 | 0.863 | 0.972 | 32.2 | -37.8 |
| wavelet-HLL_glszm_SmallAreaHighGrayLevelEmphasis | 29.48 | 81.72 | 0.987 | 0.969 | 0.994 | 65.3 | -98.2 |
| wavelet-HLL_glszm_SmallAreaLowGrayLevelEmphasis | 37.03 | 102.65 | 0.453 | 0.075 | 0.719 | 98.4 | -106.9 |
| wavelet-HLL_glszm_ZoneEntropy | 3.99 | 11.07 | 0.968 | 0.921 | 0.987 | 8.7 | -13.4 |
| wavelet-HLL_glszm_ZonePercentage | 14.93 | 41.37 | 0.967 | 0.925 | 0.985 | 37.4 | -45.4 |
| wavelet-HLL_glszm_ZoneVariance | 41.66 | 115.47 | 0.884 | 0.754 | 0.948 | 105.2 | -125.8 |
| wavelet-HLL_ngtdm_Busyness | 28.22 | 78.22 | 0.921 | 0.829 | 0.965 | 82.4 | -74 |
| wavelet-HLL_ngtdm_Coarseness | 15.15 | 41.98 | 0.824 | 0.614 | 0.922 | 51.6 | -32.3 |
| wavelet-HLL_ngtdm_Complexity | 31.66 | 87.76 | 0.98 | 0.944 | 0.992 | 72.3 | -103.3 |
| wavelet-HLL_ngtdm_Contrast | 18.14 | 50.29 | 0.891 | 0.768 | 0.951 | 49.7 | -50.9 |
| wavelet-HLL_ngtdm_Strength | 26.75 | 74.13 | 0.988 | 0.972 | 0.995 | 67.1 | -81.2 |
| wavelet-HLH_firstorder_10Percentile | 12.60 | 34.91 | 0.98 | 0.956 | 0.991 | 32.2 | -37.7 |
| wavelet-HLH_firstorder_90Percentile | 10.65 | 29.51 | 0.976 | 0.947 | 0.99 | 27.8 | -31.3 |
| wavelet-HLH_firstorder_Energy | 19.74 | 54.71 | 0.981 | 0.955 | 0.992 | 44.6 | -64.8 |
| wavelet-HLH_firstorder_Entropy | 3.57 | 9.89 | 0.973 | 0.939 | 0.988 | 8.9 | -10.9 |
| wavelet-HLH_firstorder_InterquartileRange | 9.31 | 25.80 | 0.961 | 0.91 | 0.983 | 29.9 | -21.7 |
| wavelet-HLH_firstorder_Kurtosis | 13.09 | 36.28 | 0.964 | 0.919 | 0.984 | 33.9 | -38.6 |
| wavelet-HLH_firstorder_Maximum | 14.08 | 39.02 | 0.974 | 0.942 | 0.989 | 37.3 | -40.7 |
| wavelet-HLH_firstorder_MeanAbsoluteDeviation | 9.19 | 25.47 | 0.985 | 0.965 | 0.993 | 25.6 | -25.3 |
| wavelet-HLH_firstorder_Mean | 109.19 | 302.65 | 0.938 | 0.857 | 0.973 | 346.6 | -258.7 |
| wavelet-HLH_firstorder_Median | 62.12 | 172.18 | 0.802 | 0.573 | 0.912 | 176.2 | -168.2 |
| wavelet-HLH_firstorder_Minimum | 14.27 | 39.55 | 0.957 | 0.904 | 0.981 | 39.2 | -39.9 |
| wavelet-HLH_firstorder_Range | 12.73 | 35.29 | 0.976 | 0.945 | 0.989 | 34.1 | -36.5 |
| wavelet-HLH_firstorder_RobustMeanAbsoluteDeviation | 9.08 | 25.17 | 0.972 | 0.936 | 0.988 | 28.3 | -22 |
| wavelet-HLH_firstorder_RootMeanSquared | 9.83 | 27.24 | 0.984 | 0.964 | 0.993 | 26.4 | -28.1 |
| wavelet-HLH_firstorder_Skewness | 45.23 | 125.37 | 0.865 | 0.716 | 0.939 | 104 | -146.7 |
| wavelet-HLH_firstorder_TotalEnergy | 19.74 | 54.71 | 0.981 | 0.955 | 0.992 | 44.6 | -64.8 |
| wavelet-HLH_firstorder_Uniformity | 2.64 | 7.31 | 0.973 | 0.94 | 0.988 | 7.6 | -7 |
| wavelet-HLH_firstorder_Variance | 19.70 | 54.62 | 0.979 | 0.953 | 0.991 | 52 | -57.2 |
| wavelet-HLH_glcm_Autocorrelation | 17.84 | 49.46 | 0.947 | 0.883 | 0.977 | 43.6 | -55.4 |
| wavelet-HLH_glcm_ClusterProminence | 22.61 | 62.69 | 0.903 | 0.791 | 0.957 | 54.8 | -70.6 |
| wavelet-HLH_glcm_ClusterShade | 33.57 | 93.06 | 0.935 | 0.853 | 0.972 | 87.6 | -98.5 |
| wavelet-HLH_glcm_ClusterTendency | 8.15 | 22.59 | 0.961 | 0.913 | 0.983 | 19 | -26.1 |
| wavelet-HLH_glcm_Contrast | 9.55 | 26.47 | 0.939 | 0.864 | 0.973 | 23.1 | -29.8 |
| wavelet-HLH_glcm_Correlation | 4.98 | 13.80 | 0.806 | 0.607 | 0.911 | 13.7 | -13.9 |
| wavelet-HLH_glcm_DifferenceAverage | 6.69 | 18.54 | 0.955 | 0.9 | 0.98 | 16.9 | -20.2 |
| wavelet-HLH_glcm_DifferenceEntropy | 5.10 | 14.14 | 0.937 | 0.859 | 0.972 | 11.9 | -16.4 |
| wavelet-HLH_glcm_DifferenceVariance | 8.90 | 24.68 | 0.925 | 0.833 | 0.967 | 20.7 | -28.7 |
| wavelet-HLH_glcm_Id | 1.56 | 4.34 | 0.958 | 0.906 | 0.981 | 4.6 | -4 |
| wavelet-HLH_glcm_Idm | 1.77 | 4.91 | 0.957 | 0.904 | 0.981 | 5.3 | -4.5 |
| wavelet-HLH_glcm_Idmn | 0.68 | 1.89 | 0.826 | 0.637 | 0.921 | 1.6 | -2.2 |
| wavelet-HLH_glcm_Idn | 0.88 | 2.43 | 0.711 | 0.445 | 0.863 | 2.1 | -2.8 |
| wavelet-HLH_glcm_Imc1 | 10.24 | 28.37 | 0.229 | -0.18 | 0.572 | 29.8 | -27 |
| wavelet-HLH_glcm_Imc2 | 3.75 | 10.39 | 0.912 | 0.811 | 0.961 | 10.6 | -10.2 |
| wavelet-HLH_glcm_InverseVariance | 5.28 | 14.64 | 0.94 | 0.869 | 0.974 | 15 | -14.3 |
| wavelet-HLH_glcm_JointAverage | 9.67 | 26.81 | 0.92 | 0.826 | 0.964 | 23.6 | -30 |
| wavelet-HLH_glcm_JointEnergy | 6.89 | 19.11 | 0.95 | 0.889 | 0.978 | 20.9 | -17.3 |
| wavelet-HLH_glcm_JointEntropy | 4.43 | 12.27 | 0.968 | 0.928 | 0.986 | 10.5 | -14.1 |
| wavelet-HLH_glcm_MCC | 4.35 | 12.04 | 0.836 | 0.661 | 0.925 | 12.5 | -11.6 |
| wavelet-HLH_glcm_MaximumProbability | 6.72 | 18.63 | 0.908 | 0.802 | 0.959 | 19.4 | -17.8 |
| wavelet-HLH_glcm_SumAverage | 9.67 | 26.81 | 0.92 | 0.826 | 0.964 | 23.6 | -30 |
| wavelet-HLH_glcm_SumEntropy | 3.52 | 9.76 | 0.973 | 0.94 | 0.988 | 8.3 | -11.2 |
| wavelet-HLH_glcm_SumSquares | 8.16 | 22.61 | 0.958 | 0.905 | 0.981 | 19.1 | -26.1 |
| wavelet-HLH_gldm_DependenceEntropy | 1.34 | 3.73 | 0.891 | 0.767 | 0.951 | 3.2 | -4.3 |
| wavelet-HLH_gldm_DependenceNonUniformity | 8.61 | 23.86 | 0.995 | 0.989 | 0.998 | 14.8 | -32.9 |
| wavelet-HLH_gldm_DependenceNonUniformityNormalized | 5.46 | 15.13 | 0.832 | 0.654 | 0.923 | 14.7 | -15.6 |
| wavelet-HLH_gldm_DependenceVariance | 8.99 | 24.92 | 0.911 | 0.808 | 0.96 | 25.3 | -24.5 |
| wavelet-HLH_gldm_GrayLevelNonUniformity | 7.37 | 20.43 | 0.996 | 0.991 | 0.998 | 12.1 | -28.7 |
| wavelet-HLH_gldm_GrayLevelVariance | 7.24 | 20.08 | 0.955 | 0.901 | 0.98 | 17.6 | -22.6 |
| wavelet-HLH_gldm_HighGrayLevelEmphasis | 17.32 | 48.01 | 0.948 | 0.884 | 0.977 | 41.5 | -54.5 |
| wavelet-HLH_gldm_LargeDependenceEmphasis | 6.59 | 18.28 | 0.979 | 0.952 | 0.991 | 17.5 | -19.1 |
| wavelet-HLH_gldm_LargeDependenceHighGrayLevelEmphasis | 18.93 | 52.46 | 0.876 | 0.738 | 0.944 | 47 | -58 |
| wavelet-HLH_gldm_LargeDependenceLowGrayLevelEmphasis | 22.70 | 62.92 | 0.854 | 0.695 | 0.934 | 68.1 | -57.7 |
| wavelet-HLH_gldm_LowGrayLevelEmphasis | 22.05 | 61.12 | 0.846 | 0.681 | 0.93 | 68.3 | -53.9 |
| wavelet-HLH_gldm_SmallDependenceEmphasis | 12.75 | 35.34 | 0.878 | 0.741 | 0.945 | 31.5 | -39.2 |
| wavelet-HLH_gldm_SmallDependenceHighGrayLevelEmphasis | 23.53 | 65.22 | 0.965 | 0.914 | 0.985 | 49.1 | -81.4 |
| wavelet-HLH_gldm_SmallDependenceLowGrayLevelEmphasis | 22.41 | 62.13 | 0.79 | 0.578 | 0.903 | 69.2 | -55 |
| wavelet-HLH_glrlm_GrayLevelNonUniformity | 8.04 | 22.28 | 0.997 | 0.993 | 0.999 | 15.2 | -29.4 |
| wavelet-HLH_glrlm_GrayLevelNonUniformityNormalized | 3.17 | 8.79 | 0.971 | 0.936 | 0.987 | 9.7 | -7.9 |
| wavelet-HLH_glrlm_GrayLevelVariance | 9.05 | 25.09 | 0.953 | 0.895 | 0.98 | 21.5 | -28.6 |
| wavelet-HLH_glrlm_HighGrayLevelRunEmphasis | 17.47 | 48.43 | 0.953 | 0.893 | 0.979 | 40.8 | -56.1 |
| wavelet-HLH_glrlm_LongRunEmphasis | 6.47 | 17.93 | 0.972 | 0.938 | 0.988 | 17.8 | -18.1 |
| wavelet-HLH_glrlm_LongRunHighGrayLevelEmphasis | 18.74 | 51.95 | 0.88 | 0.747 | 0.946 | 46.6 | -57.3 |
| wavelet-HLH_glrlm_LongRunLowGrayLevelEmphasis | 22.04 | 61.09 | 0.841 | 0.671 | 0.928 | 67.1 | -55 |
| wavelet-HLH_glrlm_LowGrayLevelRunEmphasis | 22.21 | 61.57 | 0.86 | 0.706 | 0.937 | 69.5 | -53.6 |
| wavelet-HLH_glrlm_RunEntropy | 2.00 | 5.54 | 0.953 | 0.863 | 0.981 | 3.9 | -7.1 |
| wavelet-HLH_glrlm_RunLengthNonUniformity | 9.96 | 27.60 | 0.995 | 0.988 | 0.998 | 21 | -34.2 |
| wavelet-HLH_glrlm_RunLengthNonUniformityNormalized | 4.02 | 11.14 | 0.96 | 0.911 | 0.982 | 12.2 | -10.1 |
| wavelet-HLH_glrlm_RunPercentage | 2.48 | 6.87 | 0.974 | 0.941 | 0.989 | 7.5 | -6.2 |
| wavelet-HLH_glrlm_RunVariance | 6.91 | 19.15 | 0.983 | 0.963 | 0.993 | 17.6 | -20.7 |
| wavelet-HLH_glrlm_ShortRunEmphasis | 2.93 | 8.13 | 0.966 | 0.925 | 0.985 | 8.8 | -7.4 |
| wavelet-HLH_glrlm_ShortRunHighGrayLevelEmphasis | 17.90 | 49.61 | 0.975 | 0.942 | 0.989 | 41.4 | -57.8 |
| wavelet-HLH_glrlm_ShortRunLowGrayLevelEmphasis | 23.04 | 63.86 | 0.854 | 0.692 | 0.934 | 72.9 | -54.8 |
| wavelet-HLH_glszm_GrayLevelNonUniformity | 30.57 | 84.73 | 0.774 | 0.549 | 0.895 | 75 | -94.5 |
| wavelet-HLH_glszm_GrayLevelNonUniformityNormalized | 15.51 | 42.98 | 0.833 | 0.655 | 0.924 | 48.5 | -37.4 |
| wavelet-HLH_glszm_GrayLevelVariance | 31.00 | 85.94 | 0.92 | 0.812 | 0.966 | 71.6 | -100.3 |
| wavelet-HLH_glszm_HighGrayLevelZoneEmphasis | 17.91 | 49.63 | 0.949 | 0.883 | 0.978 | 40.9 | -58.3 |
| wavelet-HLH_glszm_LargeAreaEmphasis | 35.21 | 97.60 | 0.932 | 0.852 | 0.97 | 94.8 | -100.4 |
| wavelet-HLH_glszm_LargeAreaHighGrayLevelEmphasis | 30.00 | 83.16 | 0.932 | 0.852 | 0.97 | 75.2 | -91.2 |
| wavelet-HLH_glszm_LargeAreaLowGrayLevelEmphasis | 47.01 | 130.30 | 0.873 | 0.732 | 0.943 | 130.9 | -129.7 |
| wavelet-HLH_glszm_LowGrayLevelZoneEmphasis | 14.59 | 40.45 | 0.888 | 0.761 | 0.95 | 42.1 | -38.8 |
| wavelet-HLH_glszm_SizeZoneNonUniformity | 20.07 | 55.62 | 0.781 | 0.538 | 0.902 | 40.6 | -70.6 |
| wavelet-HLH_glszm_SizeZoneNonUniformityNormalized | 18.03 | 49.98 | 0.782 | 0.564 | 0.899 | 50.9 | -49.1 |
| wavelet-HLH_glszm_SmallAreaEmphasis | 83.91 | 232.58 | 0.364 | -0.027 | 0.662 | 184 | -281.2 |
| wavelet-HLH_glszm_SmallAreaHighGrayLevelEmphasis | 83.36 | 231.06 | 0.596 | 0.267 | 0.802 | 173.6 | -288.5 |
| wavelet-HLH_glszm_SmallAreaLowGrayLevelEmphasis | 90.93 | 252.03 | 0.461 | 0.087 | 0.723 | 209.7 | -294.3 |
| wavelet-HLH_glszm_ZoneEntropy | 16.20 | 44.90 | 0.87 | 0.727 | 0.941 | 39.2 | -50.6 |
| wavelet-HLH_glszm_ZonePercentage | 28.63 | 79.36 | 0.837 | 0.663 | 0.926 | 72.2 | -86.5 |
| wavelet-HLH_glszm_ZoneVariance | 32.83 | 90.99 | 0.944 | 0.877 | 0.975 | 64.3 | -117.7 |
| wavelet-HLH_ngtdm_Busyness | 36.89 | 102.25 | 0.917 | 0.82 | 0.963 | 111.6 | -92.9 |
| wavelet-HLH_ngtdm_Coarseness | 11.99 | 33.23 | 0.893 | 0.752 | 0.954 | 42.3 | -24.1 |
| wavelet-HLH_ngtdm_Complexity | 29.58 | 82.00 | 0.954 | 0.879 | 0.981 | 60.3 | -103.7 |
| wavelet-HLH_ngtdm_Contrast | 28.50 | 78.99 | 0.808 | 0.608 | 0.912 | 93.4 | -64.6 |
| wavelet-HLH_ngtdm_Strength | 35.24 | 97.68 | 0.93 | 0.844 | 0.969 | 86.3 | -109.1 |
| wavelet-HHL_firstorder_10Percentile | 16.53 | 45.82 | 0.963 | 0.917 | 0.984 | 37.1 | -54.6 |
| wavelet-HHL_firstorder_90Percentile | 9.87 | 27.35 | 0.976 | 0.945 | 0.989 | 24.8 | -29.8 |
| wavelet-HHL_firstorder_Energy | 24.27 | 67.28 | 0.979 | 0.951 | 0.991 | 50.2 | -84.4 |
| wavelet-HHL_firstorder_Entropy | 3.87 | 10.74 | 0.972 | 0.93 | 0.988 | 7.9 | -13.5 |
| wavelet-HHL_firstorder_InterquartileRange | 12.45 | 34.50 | 0.947 | 0.883 | 0.977 | 32 | -37 |
| wavelet-HHL_firstorder_Kurtosis | 16.34 | 45.30 | 0.925 | 0.838 | 0.967 | 42.1 | -48.5 |
| wavelet-HHL_firstorder_Maximum | 17.33 | 48.03 | 0.946 | 0.881 | 0.976 | 42.9 | -53.1 |
| wavelet-HHL_firstorder_MeanAbsoluteDeviation | 11.66 | 32.33 | 0.97 | 0.932 | 0.987 | 28 | -36.7 |
| wavelet-HHL_firstorder_Mean | 112.48 | 311.77 | 0.918 | 0.822 | 0.964 | 293.3 | -330.2 |
| wavelet-HHL_firstorder_Median | 4054.55 | 11238.63 | 0.829 | 0.648 | 0.922 | 12434 | -10043.3 |
| wavelet-HHL_firstorder_Minimum | 23.91 | 66.28 | 0.923 | 0.806 | 0.968 | 46.5 | -86.1 |
| wavelet-HHL_firstorder_Range | 18.48 | 51.23 | 0.965 | 0.919 | 0.985 | 40.5 | -62 |
| wavelet-HHL_firstorder_RobustMeanAbsoluteDeviation | 11.74 | 32.54 | 0.952 | 0.893 | 0.979 | 29.3 | -35.8 |
| wavelet-HHL_firstorder_RootMeanSquared | 11.55 | 32.00 | 0.976 | 0.946 | 0.989 | 27.5 | -36.5 |
| wavelet-HHL_firstorder_Skewness | 54.70 | 151.61 | 0.747 | 0.502 | 0.881 | 177.2 | -126 |
| wavelet-HHL_firstorder_TotalEnergy | 24.27 | 67.28 | 0.979 | 0.951 | 0.991 | 50.2 | -84.4 |
| wavelet-HHL_firstorder_Uniformity | 3.26 | 9.05 | 0.96 | 0.898 | 0.983 | 10.9 | -7.2 |
| wavelet-HHL_firstorder_Variance | 24.03 | 66.61 | 0.961 | 0.913 | 0.983 | 56.5 | -76.7 |
| wavelet-HHL_glcm_Autocorrelation | 27.74 | 76.89 | 0.874 | 0.725 | 0.944 | 63.8 | -90 |
| wavelet-HHL_glcm_ClusterProminence | 22.94 | 63.57 | 0.95 | 0.889 | 0.978 | 51.7 | -75.4 |
| wavelet-HHL_glcm_ClusterShade | 191.46 | 530.71 | 0.944 | 0.876 | 0.975 | 461.4 | -600 |
| wavelet-HHL_glcm_ClusterTendency | 9.17 | 25.41 | 0.968 | 0.928 | 0.986 | 19.6 | -31.2 |
| wavelet-HHL_glcm_Contrast | 10.52 | 29.15 | 0.95 | 0.89 | 0.978 | 26.7 | -31.6 |
| wavelet-HHL_glcm_Correlation | 6.07 | 16.83 | 0.839 | 0.653 | 0.928 | 13.2 | -20.4 |
| wavelet-HHL_glcm_DifferenceAverage | 7.19 | 19.92 | 0.964 | 0.919 | 0.984 | 18.6 | -21.2 |
| wavelet-HHL_glcm_DifferenceEntropy | 4.72 | 13.09 | 0.954 | 0.898 | 0.98 | 11.6 | -14.6 |
| wavelet-HHL_glcm_DifferenceVariance | 9.75 | 27.03 | 0.934 | 0.855 | 0.971 | 24.3 | -29.8 |
| wavelet-HHL_glcm_Id | 1.56 | 4.33 | 0.966 | 0.924 | 0.985 | 4.5 | -4.2 |
| wavelet-HHL_glcm_Idm | 1.80 | 5.00 | 0.966 | 0.924 | 0.985 | 5.2 | -4.8 |
| wavelet-HHL_glcm_Idmn | 0.50 | 1.38 | 0.898 | 0.781 | 0.955 | 1.2 | -1.6 |
| wavelet-HHL_glcm_Idn | 0.54 | 1.50 | 0.92 | 0.826 | 0.965 | 1.3 | -1.7 |
| wavelet-HHL_glcm_Imc1 | 8.43 | 23.37 | 0.758 | 0.523 | 0.887 | 19.1 | -27.6 |
| wavelet-HHL_glcm_Imc2 | 5.08 | 14.07 | 0.889 | 0.704 | 0.955 | 10.1 | -18.1 |
| wavelet-HHL_glcm_InverseVariance | 5.75 | 15.95 | 0.932 | 0.851 | 0.97 | 15.8 | -16.1 |
| wavelet-HHL_glcm_JointAverage | 15.23 | 42.22 | 0.826 | 0.638 | 0.921 | 35.3 | -49.2 |
| wavelet-HHL_glcm_JointEnergy | 6.87 | 19.05 | 0.96 | 0.908 | 0.982 | 21.8 | -16.3 |
| wavelet-HHL_glcm_JointEntropy | 4.71 | 13.04 | 0.971 | 0.932 | 0.987 | 10.5 | -15.6 |
| wavelet-HHL_glcm_MCC | 5.65 | 15.65 | 0.837 | 0.618 | 0.93 | 11.5 | -19.8 |
| wavelet-HHL_glcm_MaximumProbability | 6.13 | 16.99 | 0.935 | 0.858 | 0.971 | 19.2 | -14.8 |
| wavelet-HHL_glcm_SumAverage | 15.23 | 42.22 | 0.826 | 0.638 | 0.921 | 35.3 | -49.2 |
| wavelet-HHL_glcm_SumEntropy | 3.93 | 10.90 | 0.97 | 0.924 | 0.988 | 8.3 | -13.5 |
| wavelet-HHL_glcm_SumSquares | 9.14 | 25.33 | 0.965 | 0.922 | 0.985 | 20.5 | -30.1 |
| wavelet-HHL_gldm_DependenceEntropy | 1.06 | 2.93 | 0.944 | 0.823 | 0.978 | 2 | -3.9 |
| wavelet-HHL_gldm_DependenceNonUniformity | 8.56 | 23.73 | 0.996 | 0.99 | 0.998 | 16 | -31.4 |
| wavelet-HHL_gldm_DependenceNonUniformityNormalized | 4.47 | 12.38 | 0.945 | 0.878 | 0.976 | 13.3 | -11.5 |
| wavelet-HHL_gldm_DependenceVariance | 8.80 | 24.40 | 0.901 | 0.788 | 0.956 | 23.4 | -25.4 |
| wavelet-HHL_gldm_GrayLevelNonUniformity | 7.69 | 21.31 | 0.997 | 0.992 | 0.999 | 14.5 | -28.1 |
| wavelet-HHL_gldm_GrayLevelVariance | 7.96 | 22.07 | 0.965 | 0.922 | 0.985 | 17.5 | -26.7 |
| wavelet-HHL_gldm_HighGrayLevelEmphasis | 27.39 | 75.92 | 0.876 | 0.729 | 0.945 | 63.3 | -88.5 |
| wavelet-HHL_gldm_LargeDependenceEmphasis | 6.92 | 19.17 | 0.964 | 0.919 | 0.984 | 17.8 | -20.6 |
| wavelet-HHL_gldm_LargeDependenceHighGrayLevelEmphasis | 27.27 | 75.58 | 0.86 | 0.697 | 0.938 | 61.8 | -89.3 |
| wavelet-HHL_gldm_LargeDependenceLowGrayLevelEmphasis | 38.25 | 106.04 | 0.604 | 0.279 | 0.806 | 118.9 | -93.2 |
| wavelet-HHL_gldm_LowGrayLevelEmphasis | 34.96 | 96.90 | 0.634 | 0.325 | 0.822 | 111.7 | -82.1 |
| wavelet-HHL_gldm_SmallDependenceEmphasis | 15.99 | 44.31 | 0.859 | 0.704 | 0.936 | 48.5 | -40.1 |
| wavelet-HHL_gldm_SmallDependenceHighGrayLevelEmphasis | 38.11 | 105.63 | 0.83 | 0.65 | 0.922 | 102.4 | -108.9 |
| wavelet-HHL_gldm_SmallDependenceLowGrayLevelEmphasis | 27.05 | 74.98 | 0.728 | 0.461 | 0.873 | 92.8 | -57.1 |
| wavelet-HHL_glrlm_GrayLevelNonUniformity | 6.44 | 17.85 | 0.996 | 0.991 | 0.998 | 12 | -23.7 |
| wavelet-HHL_glrlm_GrayLevelNonUniformityNormalized | 3.78 | 10.48 | 0.955 | 0.883 | 0.981 | 12.7 | -8.2 |
| wavelet-HHL_glrlm_GrayLevelVariance | 9.28 | 25.74 | 0.961 | 0.914 | 0.983 | 20.5 | -30.9 |
| wavelet-HHL_glrlm_HighGrayLevelRunEmphasis | 26.67 | 73.93 | 0.887 | 0.749 | 0.95 | 61.3 | -86.5 |
| wavelet-HHL_glrlm_LongRunEmphasis | 8.38 | 23.22 | 0.936 | 0.859 | 0.971 | 22.9 | -23.5 |
| wavelet-HHL_glrlm_LongRunHighGrayLevelEmphasis | 26.05 | 72.21 | 0.892 | 0.762 | 0.952 | 58.9 | -85.5 |
| wavelet-HHL_glrlm_LongRunLowGrayLevelEmphasis | 38.24 | 106.00 | 0.588 | 0.258 | 0.797 | 120.3 | -91.7 |
| wavelet-HHL_glrlm_LowGrayLevelRunEmphasis | 34.48 | 95.59 | 0.695 | 0.42 | 0.855 | 110.4 | -80.8 |
| wavelet-HHL_glrlm_RunEntropy | 1.62 | 4.49 | 0.962 | 0.749 | 0.988 | 2.4 | -6.6 |
| wavelet-HHL_glrlm_RunLengthNonUniformity | 8.63 | 23.91 | 0.99 | 0.978 | 0.996 | 16.9 | -30.9 |
| wavelet-HHL_glrlm_RunLengthNonUniformityNormalized | 4.81 | 13.34 | 0.962 | 0.916 | 0.983 | 14.5 | -12.2 |
| wavelet-HHL_glrlm_RunPercentage | 3.23 | 8.96 | 0.967 | 0.925 | 0.985 | 9.5 | -8.4 |
| wavelet-HHL_glrlm_RunVariance | 8.35 | 23.16 | 0.973 | 0.938 | 0.988 | 19.6 | -26.7 |
| wavelet-HHL_glrlm_ShortRunEmphasis | 4.17 | 11.56 | 0.944 | 0.876 | 0.975 | 11.7 | -11.4 |
| wavelet-HHL_glrlm_ShortRunHighGrayLevelEmphasis | 29.18 | 80.88 | 0.918 | 0.816 | 0.964 | 68.4 | -93.4 |
| wavelet-HHL_glrlm_ShortRunLowGrayLevelEmphasis | 32.23 | 89.34 | 0.731 | 0.478 | 0.873 | 104.5 | -74.2 |
| wavelet-HHL_glszm_GrayLevelNonUniformity | 28.15 | 78.02 | 0.93 | 0.847 | 0.969 | 77.4 | -78.7 |
| wavelet-HHL_glszm_GrayLevelNonUniformityNormalized | 16.42 | 45.53 | 0.83 | 0.65 | 0.922 | 49.6 | -41.5 |
| wavelet-HHL_glszm_GrayLevelVariance | 31.11 | 86.24 | 0.876 | 0.737 | 0.944 | 79.6 | -92.9 |
| wavelet-HHL_glszm_HighGrayLevelZoneEmphasis | 25.58 | 70.92 | 0.921 | 0.829 | 0.965 | 61.1 | -80.8 |
| wavelet-HHL_glszm_LargeAreaEmphasis | 27.64 | 76.62 | 0.987 | 0.97 | 0.994 | 66.4 | -86.9 |
| wavelet-HHL_glszm_LargeAreaHighGrayLevelEmphasis | 40.17 | 111.35 | 0.886 | 0.758 | 0.949 | 89.3 | -133.4 |
| wavelet-HHL_glszm_LargeAreaLowGrayLevelEmphasis | 40.51 | 112.29 | 0.741 | 0.493 | 0.878 | 115.5 | -109 |
| wavelet-HHL_glszm_LowGrayLevelZoneEmphasis | 24.29 | 67.33 | 0.711 | 0.445 | 0.863 | 79.7 | -55 |
| wavelet-HHL_glszm_SizeZoneNonUniformity | 14.29 | 39.61 | 0.877 | 0.74 | 0.945 | 37.2 | -42.1 |
| wavelet-HHL_glszm_SizeZoneNonUniformityNormalized | 16.24 | 45.00 | 0.877 | 0.739 | 0.944 | 47.3 | -42.7 |
| wavelet-HHL_glszm_SmallAreaEmphasis | 77.76 | 215.53 | 0.425 | 0.041 | 0.702 | 211.7 | -219.4 |
| wavelet-HHL_glszm_SmallAreaHighGrayLevelEmphasis | 79.07 | 219.18 | 0.044 | -0.356 | 0.432 | 228.4 | -210 |
| wavelet-HHL_glszm_SmallAreaLowGrayLevelEmphasis | 90.94 | 252.08 | 0.394 | 0.003 | 0.682 | 236.8 | -267.3 |
| wavelet-HHL_glszm_ZoneEntropy | 12.37 | 34.29 | 0.932 | 0.851 | 0.97 | 33.1 | -35.5 |
| wavelet-HHL_glszm_ZonePercentage | 20.65 | 57.23 | 0.891 | 0.767 | 0.951 | 60.9 | -53.5 |
| wavelet-HHL_glszm_ZoneVariance | 31.38 | 86.98 | 0.989 | 0.974 | 0.995 | 70 | -103.9 |
| wavelet-HHL_ngtdm_Busyness | 37.63 | 104.30 | 0.898 | 0.782 | 0.955 | 112.7 | -95.9 |
| wavelet-HHL_ngtdm_Coarseness | 10.42 | 28.89 | 0.903 | 0.789 | 0.957 | 35 | -22.8 |
| wavelet-HHL_ngtdm_Complexity | 28.01 | 77.64 | 0.942 | 0.872 | 0.974 | 68.5 | -86.7 |
| wavelet-HHL_ngtdm_Contrast | 22.02 | 61.02 | 0.869 | 0.725 | 0.941 | 68.2 | -53.9 |
| wavelet-HHL_ngtdm_Strength | 29.58 | 81.99 | 0.807 | 0.609 | 0.911 | 80.9 | -83.1 |
| wavelet-HHH_firstorder_10Percentile | 11.18 | 31.00 | 0.939 | 0.867 | 0.973 | 28.9 | -33.1 |
| wavelet-HHH_firstorder_90Percentile | 14.35 | 39.78 | 0.964 | 0.92 | 0.984 | 35.8 | -43.8 |
| wavelet-HHH_firstorder_Energy | 22.36 | 61.97 | 0.982 | 0.958 | 0.992 | 50.4 | -73.6 |
| wavelet-HHH_firstorder_Entropy | 0.14 | 0.40 | 0.614 | 0.299 | 0.811 | 0.3 | -0.5 |
| wavelet-HHH_firstorder_InterquartileRange | 10.83 | 30.03 | 0.952 | 0.893 | 0.979 | 28.9 | -31.2 |
| wavelet-HHH_firstorder_Kurtosis | 15.29 | 42.39 | 0.783 | 0.565 | 0.899 | 45.7 | -39 |
| wavelet-HHH_firstorder_Maximum | 17.64 | 48.91 | 0.977 | 0.947 | 0.99 | 48.5 | -49.3 |
| wavelet-HHH_firstorder_MeanAbsoluteDeviation | 10.29 | 28.52 | 0.978 | 0.95 | 0.99 | 26.9 | -30.2 |
| wavelet-HHH_firstorder_Mean | 179.74 | 498.23 | 0.83 | 0.651 | 0.922 | 540.7 | -455.7 |
| wavelet-HHH_firstorder_Median | 6505.83 | 18033.25 | 0.767 | 0.538 | 0.892 | 16183.9 | -19882.6 |
| wavelet-HHH_firstorder_Minimum | 16.27 | 45.11 | 0.966 | 0.924 | 0.985 | 46.1 | -44.2 |
| wavelet-HHH_firstorder_Range | 14.79 | 41.00 | 0.975 | 0.943 | 0.989 | 41.4 | -40.6 |
| wavelet-HHH_firstorder_RobustMeanAbsoluteDeviation | 9.66 | 26.77 | 0.971 | 0.936 | 0.987 | 25.8 | -27.8 |
| wavelet-HHH_firstorder_RootMeanSquared | 10.98 | 30.43 | 0.979 | 0.953 | 0.991 | 28.8 | -32 |
| wavelet-HHH_firstorder_Skewness | 34.95 | 96.88 | 0.733 | 0.479 | 0.874 | 108.2 | -85.5 |
| wavelet-HHH_firstorder_TotalEnergy | 22.36 | 61.97 | 0.982 | 0.958 | 0.992 | 50.4 | -73.6 |
| wavelet-HHH_firstorder_Uniformity | 0.20 | 0.55 | 0.614 | 0.298 | 0.811 | 0.6 | -0.5 |
| wavelet-HHH_firstorder_Variance | 21.97 | 60.89 | 0.969 | 0.931 | 0.986 | 57.4 | -64.3 |
| wavelet-HHH_glcm_Autocorrelation | 1.84 | 5.10 | 0.775 | 0.551 | 0.895 | 4.4 | -5.8 |
| wavelet-HHH_glcm_ClusterProminence | 1.94 | 5.39 | 0.636 | 0.326 | 0.823 | 5.2 | -5.6 |
| wavelet-HHH_glcm_ClusterShade | 221.69 | 614.50 | 0.785 | 0.57 | 0.9 | 658.6 | -570.4 |
| wavelet-HHH_glcm_ClusterTendency | 1.97 | 5.47 | 0.598 | 0.27 | 0.803 | 5.3 | -5.7 |
| wavelet-HHH_glcm_Contrast | 3.63 | 10.07 | 0.635 | 0.324 | 0.823 | 10.4 | -9.7 |
| wavelet-HHH_glcm_Correlation | 8.11 | 22.48 | 0.622 | 0.306 | 0.816 | 22 | -23 |
| wavelet-HHH_glcm_DifferenceAverage | 3.63 | 10.07 | 0.635 | 0.324 | 0.823 | 10.4 | -9.7 |
| wavelet-HHH_glcm_DifferenceEntropy | 1.40 | 3.87 | 0.816 | 0.624 | 0.915 | 4.1 | -3.6 |
| wavelet-HHH_glcm_DifferenceVariance | 1.88 | 5.20 | 0.807 | 0.608 | 0.911 | 5.5 | -4.9 |
| wavelet-HHH_glcm_Id | 0.75 | 2.08 | 0.635 | 0.324 | 0.823 | 2 | -2.1 |
| wavelet-HHH_glcm_Idm | 0.75 | 2.08 | 0.635 | 0.324 | 0.823 | 2 | -2.1 |
| wavelet-HHH_glcm_Idmn | 0.27 | 0.74 | 0.635 | 0.324 | 0.823 | 0.7 | -0.8 |
| wavelet-HHH_glcm_Idn | 0.47 | 1.30 | 0.635 | 0.324 | 0.823 | 1.3 | -1.3 |
| wavelet-HHH_glcm_Imc1 | 12.37 | 34.29 | 0.816 | 0.624 | 0.915 | 33.2 | -35.4 |
| wavelet-HHH_glcm_Imc2 | 6.58 | 18.25 | 0.738 | 0.488 | 0.877 | 17.3 | -19.2 |
| wavelet-HHH_glcm_InverseVariance | 3.63 | 10.07 | 0.635 | 0.324 | 0.823 | 10.4 | -9.7 |
| wavelet-HHH_glcm_JointAverage | 0.94 | 2.60 | 0.768 | 0.539 | 0.892 | 2.3 | -2.9 |
| wavelet-HHH_glcm_JointEnergy | 1.53 | 4.24 | 0.818 | 0.628 | 0.916 | 4.1 | -4.4 |
| wavelet-HHH_glcm_JointEntropy | 0.70 | 1.93 | 0.828 | 0.647 | 0.921 | 2 | -1.8 |
| wavelet-HHH_glcm_MCC | 6.97 | 19.32 | 0.75 | 0.508 | 0.883 | 18.3 | -20.3 |
| wavelet-HHH_glcm_MaximumProbability | 3.20 | 8.88 | 0.737 | 0.487 | 0.877 | 9.7 | -8.1 |
| wavelet-HHH_glcm_SumAverage | 0.94 | 2.60 | 0.768 | 0.539 | 0.892 | 2.3 | -2.9 |
| wavelet-HHH_glcm_SumEntropy | 0.51 | 1.42 | 0.909 | 0.804 | 0.959 | 1.5 | -1.4 |
| wavelet-HHH_glcm_SumSquares | 0.34 | 0.95 | 0.652 | 0.35 | 0.832 | 0.9 | -1 |
| wavelet-HHH_gldm_DependenceEntropy | 0.95 | 2.64 | 0.771 | 0.545 | 0.894 | 2.5 | -2.8 |
| wavelet-HHH_gldm_DependenceNonUniformity | 7.96 | 22.07 | 0.997 | 0.993 | 0.999 | 13.8 | -30.3 |
| wavelet-HHH_gldm_DependenceNonUniformityNormalized | 3.47 | 9.63 | 0.647 | 0.342 | 0.829 | 10 | -9.3 |
| wavelet-HHH_gldm_DependenceVariance | 7.67 | 21.27 | 0.26 | -0.148 | 0.594 | 22.7 | -19.8 |
| wavelet-HHH_gldm_GrayLevelNonUniformity | 7.57 | 20.99 | 0.996 | 0.992 | 0.998 | 12.5 | -29.5 |
| wavelet-HHH_gldm_GrayLevelVariance | 0.20 | 0.56 | 0.614 | 0.298 | 0.811 | 0.5 | -0.6 |
| wavelet-HHH_gldm_HighGrayLevelEmphasis | 1.39 | 3.85 | 0.803 | 0.602 | 0.91 | 3.5 | -4.2 |
| wavelet-HHH_gldm_LargeDependenceEmphasis | 3.38 | 9.38 | 0.954 | 0.896 | 0.98 | 7.8 | -10.9 |
| wavelet-HHH_gldm_LargeDependenceHighGrayLevelEmphasis | 4.79 | 13.28 | 0.95 | 0.877 | 0.979 | 10.4 | -16.2 |
| wavelet-HHH_gldm_LargeDependenceLowGrayLevelEmphasis | 4.98 | 13.79 | 0.862 | 0.71 | 0.937 | 13.6 | -13.9 |
| wavelet-HHH_gldm_LowGrayLevelEmphasis | 1.39 | 3.87 | 0.803 | 0.602 | 0.91 | 4.2 | -3.5 |
| wavelet-HHH_gldm_SmallDependenceEmphasis | 15.14 | 41.98 | 0.599 | 0.272 | 0.804 | 44.4 | -39.5 |
| wavelet-HHH_gldm_SmallDependenceHighGrayLevelEmphasis | 13.97 | 38.71 | 0.721 | 0.461 | 0.868 | 43.7 | -33.7 |
| wavelet-HHH_gldm_SmallDependenceLowGrayLevelEmphasis | 19.34 | 53.62 | 0.35 | -0.049 | 0.654 | 53.8 | -53.4 |
| wavelet-HHH_glrlm_GrayLevelNonUniformity | 6.91 | 19.14 | 0.997 | 0.993 | 0.999 | 11.6 | -26.6 |
| wavelet-HHH_glrlm_GrayLevelNonUniformityNormalized | 0.26 | 0.71 | 0.846 | 0.68 | 0.93 | 0.8 | -0.6 |
| wavelet-HHH_glrlm_GrayLevelVariance | 0.26 | 0.73 | 0.846 | 0.68 | 0.93 | 0.7 | -0.8 |
| wavelet-HHH_glrlm_HighGrayLevelRunEmphasis | 1.25 | 3.48 | 0.824 | 0.638 | 0.919 | 3.6 | -3.4 |
| wavelet-HHH_glrlm_LongRunEmphasis | 4.07 | 11.27 | 0.967 | 0.92 | 0.986 | 8.9 | -13.6 |
| wavelet-HHH_glrlm_LongRunHighGrayLevelEmphasis | 4.25 | 11.78 | 0.973 | 0.927 | 0.989 | 8.6 | -14.9 |
| wavelet-HHH_glrlm_LongRunLowGrayLevelEmphasis | 5.19 | 14.39 | 0.935 | 0.858 | 0.971 | 12.8 | -16 |
| wavelet-HHH_glrlm_LowGrayLevelRunEmphasis | 1.31 | 3.62 | 0.824 | 0.638 | 0.919 | 3.5 | -3.7 |
| wavelet-HHH_glrlm_RunEntropy | 1.38 | 3.82 | 0.966 | 0.921 | 0.985 | 3.2 | -4.4 |
| wavelet-HHH_glrlm_RunLengthNonUniformity | 7.15 | 19.81 | 0.997 | 0.994 | 0.999 | 13.5 | -26.1 |
| wavelet-HHH_glrlm_RunLengthNonUniformityNormalized | 3.25 | 9.01 | 0.929 | 0.844 | 0.968 | 10.3 | -7.8 |
| wavelet-HHH_glrlm_RunPercentage | 2.00 | 5.54 | 0.947 | 0.879 | 0.977 | 6.6 | -4.5 |
| wavelet-HHH_glrlm_RunVariance | 4.99 | 13.82 | 0.978 | 0.932 | 0.991 | 10.3 | -17.3 |
| wavelet-HHH_glrlm_ShortRunEmphasis | 3.14 | 8.71 | 0.863 | 0.713 | 0.938 | 9.7 | -7.8 |
| wavelet-HHH_glrlm_ShortRunHighGrayLevelEmphasis | 3.76 | 10.42 | 0.88 | 0.743 | 0.946 | 11.9 | -8.9 |
| wavelet-HHH_glrlm_ShortRunLowGrayLevelEmphasis | 4.30 | 11.92 | 0.776 | 0.552 | 0.896 | 12.3 | -11.5 |
| wavelet-HHH_glszm_GrayLevelNonUniformity | 32.38 | 89.74 | 0.904 | 0.794 | 0.957 | 92.1 | -87.4 |
| wavelet-HHH_glszm_GrayLevelNonUniformityNormalized | 8.17 | 22.65 | 0.559 | 0.215 | 0.781 | 24.4 | -20.9 |
| wavelet-HHH_glszm_GrayLevelVariance | 10.93 | 30.29 | 0.559 | 0.215 | 0.781 | 28.4 | -32.2 |
| wavelet-HHH_glszm_HighGrayLevelZoneEmphasis | 17.17 | 47.59 | 0.358 | -0.04 | 0.659 | 50.4 | -44.8 |
| wavelet-HHH_glszm_LargeAreaEmphasis | 36.13 | 100.14 | 0.993 | 0.985 | 0.997 | 83.4 | -116.9 |
| wavelet-HHH_glszm_LargeAreaHighGrayLevelEmphasis | 35.36 | 98.00 | 0.994 | 0.986 | 0.997 | 80.6 | -115.4 |
| wavelet-HHH_glszm_LargeAreaLowGrayLevelEmphasis | 37.08 | 102.78 | 0.993 | 0.984 | 0.997 | 86.5 | -119 |
| wavelet-HHH_glszm_LowGrayLevelZoneEmphasis | 15.92 | 44.12 | 0.358 | -0.04 | 0.659 | 40.7 | -47.6 |
| wavelet-HHH_glszm_SizeZoneNonUniformity | 23.41 | 64.89 | 0.885 | 0.75 | 0.949 | 55.9 | -73.9 |
| wavelet-HHH_glszm_SizeZoneNonUniformityNormalized | 17.89 | 49.59 | 0.183 | -0.184 | 0.524 | 39.8 | -59.4 |
| wavelet-HHH_glszm_SmallAreaEmphasis | 94.78 | 262.71 | 0.505 | 0.142 | 0.75 | 268.2 | -257.3 |
| wavelet-HHH_glszm_SmallAreaHighGrayLevelEmphasis | 100.07 | 277.37 | 0.373 | -0.022 | 0.669 | 295.8 | -259 |
| wavelet-HHH_glszm_SmallAreaLowGrayLevelEmphasis | 100.70 | 279.13 | 0.435 | 0.052 | 0.708 | 272.5 | -285.8 |
| wavelet-HHH_glszm_ZoneEntropy | 23.06 | 63.91 | 0.211 | -0.197 | 0.559 | 71.6 | -56.3 |
| wavelet-HHH_glszm_ZonePercentage | 31.64 | 87.70 | 0.656 | 0.357 | 0.834 | 96.7 | -78.7 |
| wavelet-HHH_glszm_ZoneVariance | 85.91 | 238.14 | 0.996 | 0.99 | 0.998 | 255.7 | -220.6 |
| wavelet-HHH_ngtdm_Busyness | 11.63 | 32.25 | 0.994 | 0.987 | 0.997 | 26.6 | -37.9 |
| wavelet-HHH_ngtdm_Coarseness | 8.25 | 22.87 | 0.945 | 0.852 | 0.978 | 30.8 | -15 |
| wavelet-HHH_ngtdm_Complexity | 4.51 | 12.50 | 0.529 | 0.174 | 0.764 | 13.2 | -11.8 |
| wavelet-HHH_ngtdm_Contrast | 4.53 | 12.57 | 0.54 | 0.189 | 0.77 | 13.2 | -12 |
| wavelet-HHH_ngtdm_Strength | 8.30 | 23.01 | 0.944 | 0.851 | 0.977 | 30.9 | -15.1 |
| wavelet-LLL_firstorder_10Percentile | 5.19 | 14.37 | 0.757 | 0.519 | 0.886 | 14.5 | -14.2 |
| wavelet-LLL_firstorder_90Percentile | 3.94 | 10.91 | 0.995 | 0.987 | 0.998 | 8.7 | -13.2 |
| wavelet-LLL_firstorder_Energy | 8.93 | 24.77 | 0.998 | 0.995 | 0.999 | 13.6 | -35.9 |
| wavelet-LLL_firstorder_Entropy | 1.28 | 3.55 | 0.991 | 0.971 | 0.997 | 2.6 | -4.5 |
| wavelet-LLL_firstorder_InterquartileRange | 6.37 | 17.67 | 0.994 | 0.987 | 0.998 | 15.4 | -19.9 |
| wavelet-LLL_firstorder_Kurtosis | 4.75 | 13.17 | 0.964 | 0.92 | 0.984 | 9.3 | -17 |
| wavelet-LLL_firstorder_Maximum | 4.25 | 11.77 | 0.995 | 0.968 | 0.998 | 7.5 | -16 |
| wavelet-LLL_firstorder_MeanAbsoluteDeviation | 6.05 | 16.77 | 0.995 | 0.987 | 0.998 | 13 | -20.5 |
| wavelet-LLL_firstorder_Mean | 3.03 | 8.39 | 0.995 | 0.988 | 0.998 | 7.7 | -9.1 |
| wavelet-LLL_firstorder_Median | 3.29 | 9.12 | 0.985 | 0.965 | 0.993 | 9.8 | -8.4 |
| wavelet-LLL_firstorder_Minimum | 5.77 | 15.99 | 0.931 | 0.831 | 0.971 | 19.6 | -12.4 |
| wavelet-LLL_firstorder_Range | 5.73 | 15.88 | 0.995 | 0.962 | 0.999 | 10.5 | -21.2 |
| wavelet-LLL_firstorder_RobustMeanAbsoluteDeviation | 6.48 | 17.97 | 0.994 | 0.986 | 0.998 | 14.7 | -21.3 |
| wavelet-LLL_firstorder_RootMeanSquared | 3.20 | 8.88 | 0.996 | 0.99 | 0.998 | 7.6 | -10.2 |
| wavelet-LLL_firstorder_Skewness | 14.27 | 39.56 | 0.961 | 0.895 | 0.984 | 28 | -51.1 |
| wavelet-LLL_firstorder_TotalEnergy | 8.93 | 24.77 | 0.998 | 0.995 | 0.999 | 13.6 | -35.9 |
| wavelet-LLL_firstorder_Uniformity | 4.80 | 13.30 | 0.983 | 0.956 | 0.993 | 17.2 | -9.4 |
| wavelet-LLL_firstorder_Variance | 11.64 | 32.26 | 0.996 | 0.988 | 0.999 | 24.3 | -40.2 |
| wavelet-LLL_glcm_Autocorrelation | 9.05 | 25.09 | 0.996 | 0.989 | 0.999 | 20.5 | -29.6 |
| wavelet-LLL_glcm_ClusterProminence | 25.17 | 69.78 | 0.989 | 0.974 | 0.995 | 48.8 | -90.7 |
| wavelet-LLL_glcm_ClusterShade | 33.09 | 91.73 | 0.989 | 0.972 | 0.995 | 61.3 | -122.2 |
| wavelet-LLL_glcm_ClusterTendency | 12.81 | 35.51 | 0.996 | 0.988 | 0.999 | 26.2 | -44.8 |
| wavelet-LLL_glcm_Contrast | 10.10 | 28.00 | 0.991 | 0.979 | 0.996 | 23.6 | -32.4 |
| wavelet-LLL_glcm_Correlation | 2.42 | 6.72 | 0.968 | 0.905 | 0.988 | 4.7 | -8.7 |
| wavelet-LLL_glcm_DifferenceAverage | 5.05 | 14.00 | 0.994 | 0.985 | 0.997 | 12 | -16 |
| wavelet-LLL_glcm_DifferenceEntropy | 1.42 | 3.92 | 0.994 | 0.983 | 0.997 | 3.1 | -4.8 |
| wavelet-LLL_glcm_DifferenceVariance | 10.62 | 29.45 | 0.992 | 0.978 | 0.997 | 23.9 | -35 |
| wavelet-LLL_glcm_Id | 4.85 | 13.45 | 0.99 | 0.977 | 0.996 | 13.2 | -13.7 |
| wavelet-LLL_glcm_Idm | 9.54 | 26.44 | 0.983 | 0.961 | 0.992 | 24 | -28.9 |
| wavelet-LLL_glcm_Idmn | 0.14 | 0.38 | 0.974 | 0.868 | 0.991 | 0.2 | -0.5 |
| wavelet-LLL_glcm_Idn | 0.31 | 0.85 | 0.98 | 0.885 | 0.994 | 0.5 | -1.2 |
| wavelet-LLL_glcm_Imc1 | 3.54 | 9.82 | 0.99 | 0.976 | 0.995 | 10.2 | -9.5 |
| wavelet-LLL_glcm_Imc2 | 0.27 | 0.76 | 0.843 | 0.674 | 0.928 | 0.7 | -0.9 |
| wavelet-LLL_glcm_InverseVariance | 9.36 | 25.96 | 0.977 | 0.948 | 0.99 | 26.6 | -25.3 |
| wavelet-LLL_glcm_JointAverage | 4.46 | 12.35 | 0.996 | 0.989 | 0.998 | 10.6 | -14.1 |
| wavelet-LLL_glcm_JointEnergy | 8.33 | 23.09 | 0.938 | 0.828 | 0.975 | 32.4 | -13.8 |
| wavelet-LLL_glcm_JointEntropy | 1.44 | 3.99 | 0.99 | 0.947 | 0.997 | 2.5 | -5.5 |
| wavelet-LLL_glcm_MCC | 3.08 | 8.54 | 0.887 | 0.759 | 0.949 | 9.2 | -7.8 |
| wavelet-LLL_glcm_MaximumProbability | 7.94 | 22.00 | 0.957 | 0.875 | 0.983 | 28.6 | -15.4 |
| wavelet-LLL_glcm_SumAverage | 4.46 | 12.35 | 0.996 | 0.989 | 0.998 | 10.6 | -14.1 |
| wavelet-LLL_glcm_SumEntropy | 1.36 | 3.78 | 0.989 | 0.963 | 0.996 | 2.6 | -4.9 |
| wavelet-LLL_glcm_SumSquares | 12.28 | 34.05 | 0.996 | 0.987 | 0.999 | 25.5 | -42.6 |
| wavelet-LLL_gldm_DependenceEntropy | 1.35 | 3.75 | 0.977 | 0.882 | 0.992 | 2.2 | -5.3 |
| wavelet-LLL_gldm_DependenceNonUniformity | 8.22 | 22.78 | 0.997 | 0.994 | 0.999 | 17.2 | -28.3 |
| wavelet-LLL_gldm_DependenceNonUniformityNormalized | 5.23 | 14.49 | 0.935 | 0.851 | 0.972 | 17.5 | -11.4 |
| wavelet-LLL_gldm_DependenceVariance | 23.97 | 66.44 | 0.956 | 0.902 | 0.981 | 55.8 | -77.1 |
| wavelet-LLL_gldm_GrayLevelNonUniformity | 6.89 | 19.10 | 0.996 | 0.991 | 0.998 | 14.4 | -23.8 |
| wavelet-LLL_gldm_GrayLevelVariance | 11.63 | 32.23 | 0.996 | 0.988 | 0.999 | 24.2 | -40.3 |
| wavelet-LLL_gldm_HighGrayLevelEmphasis | 9.32 | 25.83 | 0.996 | 0.989 | 0.999 | 21.2 | -30.5 |
| wavelet-LLL_gldm_LargeDependenceEmphasis | 7.87 | 21.82 | 0.969 | 0.93 | 0.987 | 18.2 | -25.4 |
| wavelet-LLL_gldm_LargeDependenceHighGrayLevelEmphasis | 12.58 | 34.88 | 0.973 | 0.938 | 0.988 | 27.1 | -42.6 |
| wavelet-LLL_gldm_LargeDependenceLowGrayLevelEmphasis | 37.04 | 102.67 | 0.681 | 0.397 | 0.847 | 92.1 | -113.3 |
| wavelet-LLL_gldm_LowGrayLevelEmphasis | 29.07 | 80.57 | 0.705 | 0.433 | 0.86 | 82.4 | -78.7 |
| wavelet-LLL_gldm_SmallDependenceEmphasis | 3.08 | 8.53 | 0.956 | 0.897 | 0.981 | 10.2 | -6.9 |
| wavelet-LLL_gldm_SmallDependenceHighGrayLevelEmphasis | 13.14 | 36.43 | 0.998 | 0.994 | 0.999 | 33.4 | -39.5 |
| wavelet-LLL_gldm_SmallDependenceLowGrayLevelEmphasis | 28.28 | 78.40 | 0.692 | 0.413 | 0.853 | 88.6 | -68.2 |
| wavelet-LLL_glrlm_GrayLevelNonUniformity | 6.86 | 19.03 | 0.996 | 0.991 | 0.998 | 14.4 | -23.7 |
| wavelet-LLL_glrlm_GrayLevelNonUniformityNormalized | 4.86 | 13.46 | 0.982 | 0.955 | 0.992 | 17.3 | -9.6 |
| wavelet-LLL_glrlm_GrayLevelVariance | 11.67 | 32.36 | 0.996 | 0.988 | 0.999 | 24.3 | -40.5 |
| wavelet-LLL_glrlm_HighGrayLevelRunEmphasis | 9.40 | 26.04 | 0.997 | 0.989 | 0.999 | 21.4 | -30.7 |
| wavelet-LLL_glrlm_LongRunEmphasis | 0.49 | 1.36 | 0.964 | 0.915 | 0.984 | 1.1 | -1.6 |
| wavelet-LLL_glrlm_LongRunHighGrayLevelEmphasis | 9.04 | 25.06 | 0.996 | 0.988 | 0.998 | 20.2 | -29.9 |
| wavelet-LLL_glrlm_LongRunLowGrayLevelEmphasis | 29.36 | 81.37 | 0.708 | 0.439 | 0.862 | 82.5 | -80.2 |
| wavelet-LLL_glrlm_LowGrayLevelRunEmphasis | 28.91 | 80.14 | 0.708 | 0.439 | 0.862 | 82.2 | -78.1 |
| wavelet-LLL_glrlm_RunEntropy | 1.26 | 3.49 | 0.99 | 0.968 | 0.996 | 2.5 | -4.5 |
| wavelet-LLL_glrlm_RunLengthNonUniformity | 7.51 | 20.82 | 0.997 | 0.992 | 0.998 | 12.5 | -29.2 |
| wavelet-LLL_glrlm_RunLengthNonUniformityNormalized | 0.30 | 0.83 | 0.965 | 0.918 | 0.985 | 1 | -0.7 |
| wavelet-LLL_glrlm_RunPercentage | 0.16 | 0.43 | 0.965 | 0.919 | 0.985 | 0.5 | -0.3 |
| wavelet-LLL_glrlm_RunVariance | 21.61 | 59.91 | 0.957 | 0.903 | 0.981 | 49.4 | -70.4 |
| wavelet-LLL_glrlm_ShortRunEmphasis | 0.12 | 0.32 | 0.965 | 0.919 | 0.985 | 0.4 | -0.3 |
| wavelet-LLL_glrlm_ShortRunHighGrayLevelEmphasis | 9.49 | 26.31 | 0.997 | 0.99 | 0.999 | 21.7 | -30.9 |
| wavelet-LLL_glrlm_ShortRunLowGrayLevelEmphasis | 28.82 | 79.88 | 0.708 | 0.439 | 0.862 | 82.2 | -77.5 |
| wavelet-LLL_glszm_GrayLevelNonUniformity | 6.80 | 18.86 | 0.996 | 0.99 | 0.998 | 14.9 | -22.9 |
| wavelet-LLL_glszm_GrayLevelNonUniformityNormalized | 5.88 | 16.29 | 0.968 | 0.927 | 0.986 | 19.8 | -12.8 |
| wavelet-LLL_glszm_GrayLevelVariance | 12.21 | 33.85 | 0.996 | 0.987 | 0.999 | 25.2 | -42.5 |
| wavelet-LLL_glszm_HighGrayLevelZoneEmphasis | 10.62 | 29.44 | 0.997 | 0.989 | 0.999 | 24.6 | -34.3 |
| wavelet-LLL_glszm_LargeAreaEmphasis | 6.24 | 17.30 | 0.968 | 0.929 | 0.986 | 14.7 | -19.9 |
| wavelet-LLL_glszm_LargeAreaHighGrayLevelEmphasis | 10.61 | 29.41 | 0.988 | 0.969 | 0.995 | 23.1 | -35.7 |
| wavelet-LLL_glszm_LargeAreaLowGrayLevelEmphasis | 32.33 | 89.60 | 0.812 | 0.618 | 0.914 | 84.4 | -94.8 |
| wavelet-LLL_glszm_LowGrayLevelZoneEmphasis | 27.88 | 77.28 | 0.744 | 0.498 | 0.88 | 83 | -71.5 |
| wavelet-LLL_glszm_SizeZoneNonUniformity | 8.54 | 23.67 | 0.997 | 0.994 | 0.999 | 18.2 | -29.2 |
| wavelet-LLL_glszm_SizeZoneNonUniformityNormalized | 3.89 | 10.77 | 0.944 | 0.87 | 0.976 | 12.7 | -8.8 |
| wavelet-LLL_glszm_SmallAreaEmphasis | 1.80 | 5.00 | 0.945 | 0.875 | 0.976 | 5.9 | -4.1 |
| wavelet-LLL_glszm_SmallAreaHighGrayLevelEmphasis | 13.21 | 36.60 | 0.998 | 0.992 | 0.999 | 32.8 | -40.5 |
| wavelet-LLL_glszm_SmallAreaLowGrayLevelEmphasis | 29.54 | 81.87 | 0.718 | 0.455 | 0.867 | 93.1 | -70.6 |
| wavelet-LLL_glszm_ZoneEntropy | 1.36 | 3.77 | 0.981 | 0.923 | 0.993 | 2.4 | -5.1 |
| wavelet-LLL_glszm_ZonePercentage | 2.06 | 5.71 | 0.964 | 0.915 | 0.985 | 6.9 | -4.6 |
| wavelet-LLL_glszm_ZoneVariance | 27.48 | 76.17 | 0.941 | 0.871 | 0.974 | 62.7 | -89.6 |
| wavelet-LLL_ngtdm_Busyness | 9.86 | 27.34 | 0.968 | 0.928 | 0.986 | 27.4 | -27.3 |
| wavelet-LLL_ngtdm_Coarseness | 5.09 | 14.11 | 0.965 | 0.909 | 0.985 | 18.7 | -9.6 |
| wavelet-LLL_ngtdm_Complexity | 14.61 | 40.50 | 0.993 | 0.981 | 0.997 | 29.7 | -51.3 |
| wavelet-LLL_ngtdm_Contrast | 8.63 | 23.91 | 0.993 | 0.983 | 0.997 | 26.2 | -21.6 |
| wavelet-LLL_ngtdm_Strength | 12.49 | 34.63 | 0.992 | 0.979 | 0.997 | 27.8 | -41.5 |
