# Supplementary material for: Prospective inter- and intra-tracer repeatability analysis of radiomics features in [68Ga]Ga-PSMA-11 and [18F]F-PSMA-1007 PET scans in metastatic prostate cancer
Source: Br J Radiol. 2023 Oct 24;96(1152):20221178. doi: 10.1259/bjr.20221178 (PMC10646662; doi:10.1259/bjr.20221178)
Supplement: Supplementary Table 2. [file bjr.20221178.suppl-04.docx]

**Supplementary Table S2**

Tabulated below are the raw repeatability metrics for the ^18^F-PSMA-1007 intra-tracer group, including: intraclass correlation coefficients (ICCs), symmetric repeatability coefficients (RCs), within-subject coefficient of variation (wCV) values, upper and lower limits of agreement (LOA).

| **Biomarker** | **wCV (%)** | **Symmetric RC (%)** | **ICC** | **ICC 95% Lower** | **ICC 95% Upper** | **Upper LOA** | **Lower LOA** |
| --- | --- | --- | --- | --- | --- | --- | --- |
| original_shape_Elongation | 6.64 | 18.42 | 0.885 | 0.7 | 0.959 | 19.3 | -17.5 |
| original_shape_Flatness | 6.57 | 18.22 | 0.957 | 0.88 | 0.985 | 18.5 | -17.9 |
| original_shape_LeastAxisLength | 4.84 | 13.43 | 0.973 | 0.919 | 0.991 | 16.7 | -10.2 |
| original_shape_MajorAxisLength | 5.82 | 16.14 | 0.991 | 0.973 | 0.997 | 19 | -13.2 |
| original_shape_Maximum2DDiameterColumn | 6.18 | 17.14 | 0.981 | 0.947 | 0.994 | 19 | -15.3 |
| original_shape_Maximum2DDiameterRow | 5.83 | 16.15 | 0.981 | 0.94 | 0.994 | 20.3 | -12 |
| original_shape_Maximum2DDiameterSlice | 4.72 | 13.08 | 0.991 | 0.973 | 0.997 | 15.6 | -10.6 |
| original_shape_Maximum3DDiameter | 4.86 | 13.47 | 0.989 | 0.957 | 0.997 | 18 | -9 |
| original_shape_MeshVolume | 12.26 | 33.98 | 0.996 | 0.99 | 0.999 | 46.1 | -21.9 |
| original_shape_MinorAxisLength | 5.95 | 16.50 | 0.987 | 0.959 | 0.996 | 20.3 | -12.7 |
| original_shape_Sphericity | 2.36 | 6.54 | 0.929 | 0.809 | 0.976 | 7.5 | -5.5 |
| original_shape_SurfaceArea | 8.74 | 24.24 | 0.995 | 0.984 | 0.998 | 31.4 | -17.1 |
| original_shape_SurfaceVolumeRatio | 4.60 | 12.74 | 0.935 | 0.689 | 0.981 | 7.6 | -17.8 |
| original_shape_VoxelVolume | 11.98 | 33.19 | 0.996 | 0.99 | 0.999 | 45 | -21.4 |
| exponential_firstorder_10Percentile | 2.79 | 7.72 | 0.529 | 0.081 | 0.808 | 9.6 | -5.9 |
| exponential_firstorder_90Percentile | 13.81 | 38.27 | 0.897 | 0.73 | 0.964 | 43.1 | -33.5 |
| exponential_firstorder_Energy | 24.92 | 69.09 | 0.981 | 0.945 | 0.993 | 88.3 | -49.9 |
| exponential_firstorder_Entropy | 37.89 | 105.02 | 0.943 | 0.843 | 0.98 | 92.5 | -117.5 |
| exponential_firstorder_InterquartileRange | 19.20 | 53.21 | 0.876 | 0.679 | 0.956 | 58.4 | -48 |
| exponential_firstorder_Kurtosis | 12.38 | 34.32 | 0.967 | 0.893 | 0.989 | 41.2 | -27.4 |
| exponential_firstorder_Maximum | 20.60 | 57.10 | 0.985 | 0.957 | 0.995 | 67 | -47.2 |
| exponential_firstorder_MeanAbsoluteDeviation | 23.17 | 64.22 | 0.945 | 0.849 | 0.981 | 71 | -57.5 |
| exponential_firstorder_Mean | 8.96 | 24.85 | 0.89 | 0.711 | 0.961 | 28 | -21.7 |
| exponential_firstorder_Median | 5.60 | 15.53 | 0.55 | 0.087 | 0.821 | 17.6 | -13.4 |
| exponential_firstorder_Minimum | 1.79 | 4.96 | 0.784 | 0.377 | 0.927 | 6.7 | -3.2 |
| exponential_firstorder_Range | 26.46 | 73.34 | 0.986 | 0.958 | 0.995 | 83.8 | -62.9 |
| exponential_firstorder_RobustMeanAbsoluteDeviation | 20.92 | 58.00 | 0.887 | 0.706 | 0.96 | 63.3 | -52.7 |
| exponential_firstorder_RootMeanSquared | 11.40 | 31.60 | 0.947 | 0.855 | 0.982 | 35.8 | -27.4 |
| exponential_firstorder_Skewness | 26.16 | 72.50 | 0.965 | 0.898 | 0.988 | 72.3 | -72.7 |
| exponential_firstorder_TotalEnergy | 24.92 | 69.09 | 0.981 | 0.945 | 0.993 | 88.3 | -49.9 |
| exponential_firstorder_Uniformity | 16.62 | 46.06 | 0.981 | 0.947 | 0.994 | 45.2 | -46.9 |
| exponential_firstorder_Variance | 40.97 | 113.58 | 0.954 | 0.872 | 0.984 | 125.4 | -101.7 |
| exponential_glcm_Autocorrelation | 45.42 | 125.89 | 0.948 | 0.856 | 0.982 | 120.8 | -131 |
| exponential_glcm_ClusterProminence | 72.14 | 199.95 | 0.905 | 0.749 | 0.967 | 200.1 | -199.9 |
| exponential_glcm_ClusterShade | 76.60 | 212.32 | 0.933 | 0.819 | 0.977 | 204.2 | -220.4 |
| exponential_glcm_ClusterTendency | 56.54 | 156.72 | 0.955 | 0.874 | 0.984 | 153.6 | -159.8 |
| exponential_glcm_Contrast | 54.78 | 151.85 | 0.943 | 0.843 | 0.98 | 146.6 | -157 |
| exponential_glcm_Correlation | 35.72 | 99.00 | 0 | -0.487 | 0.494 | 113 | -85 |
| exponential_glcm_DifferenceAverage | 44.04 | 122.07 | 0.92 | 0.784 | 0.972 | 112.5 | -131.6 |
| exponential_glcm_DifferenceEntropy | 38.81 | 107.57 | 0.919 | 0.783 | 0.972 | 96.4 | -118.8 |
| exponential_glcm_DifferenceVariance | 55.31 | 153.32 | 0.951 | 0.866 | 0.983 | 152 | -154.6 |
| exponential_glcm_Id | 9.81 | 27.19 | 0.931 | 0.812 | 0.976 | 25.9 | -28.4 |
| exponential_glcm_Idm | 12.99 | 36.02 | 0.929 | 0.809 | 0.976 | 34.1 | -37.9 |
| exponential_glcm_Idmn | 0.54 | 1.49 | 0.802 | 0.517 | 0.928 | 1.7 | -1.3 |
| exponential_glcm_Idn | 0.67 | 1.87 | 0.95 | 0.848 | 0.984 | 2.3 | -1.4 |
| exponential_glcm_Imc1 | 38.15 | 105.76 | 0.955 | 0.875 | 0.985 | 95.3 | -116.2 |
| exponential_glcm_Imc2 | 36.96 | 102.45 | 0.983 | 0.951 | 0.994 | 90.1 | -114.8 |
| exponential_glcm_InverseVariance | 37.02 | 102.61 | 0.93 | 0.805 | 0.976 | 85 | -120.2 |
| exponential_glcm_JointAverage | 24.40 | 67.62 | 0.892 | 0.716 | 0.962 | 64 | -71.2 |
| exponential_glcm_JointEnergy | 29.93 | 82.97 | 0.992 | 0.978 | 0.997 | 83.2 | -82.7 |
| exponential_glcm_JointEntropy | 37.69 | 104.48 | 0.946 | 0.851 | 0.981 | 91.7 | -117.3 |
| exponential_glcm_MCC | 33.53 | 92.93 | 0.069 | -0.413 | 0.538 | 107.8 | -78.1 |
| exponential_glcm_MaximumProbability | 29.32 | 81.27 | 0.974 | 0.926 | 0.991 | 81.5 | -81 |
| exponential_glcm_SumAverage | 24.40 | 67.62 | 0.892 | 0.716 | 0.962 | 64 | -71.2 |
| exponential_glcm_SumEntropy | 37.54 | 104.05 | 0.954 | 0.871 | 0.984 | 91.8 | -116.3 |
| exponential_glcm_SumSquares | 56.23 | 155.85 | 0.953 | 0.87 | 0.984 | 152.4 | -159.3 |
| exponential_gldm_DependenceEntropy | 3.66 | 10.15 | 0.947 | 0.854 | 0.982 | 11 | -9.3 |
| exponential_gldm_DependenceNonUniformity | 15.32 | 42.47 | 0.964 | 0.9 | 0.988 | 52.4 | -32.5 |
| exponential_gldm_DependenceNonUniformityNormalized | 14.22 | 39.42 | 0.462 | -0.033 | 0.778 | 37.5 | -41.3 |
| exponential_gldm_DependenceVariance | 27.23 | 75.49 | 0.852 | 0.58 | 0.95 | 85.3 | -65.7 |
| exponential_gldm_GrayLevelNonUniformity | 19.93 | 55.25 | 0.97 | 0.907 | 0.99 | 66.2 | -44.3 |
| exponential_gldm_GrayLevelVariance | 56.01 | 155.25 | 0.954 | 0.872 | 0.984 | 151.8 | -158.7 |
| exponential_gldm_HighGrayLevelEmphasis | 45.63 | 126.49 | 0.95 | 0.862 | 0.983 | 121.6 | -131.4 |
| exponential_gldm_LargeDependenceEmphasis | 26.87 | 74.49 | 0.952 | 0.85 | 0.984 | 80 | -69 |
| exponential_gldm_LargeDependenceHighGrayLevelEmphasis | 29.33 | 81.29 | 0.893 | 0.719 | 0.962 | 73.7 | -88.8 |
| exponential_gldm_LargeDependenceLowGrayLevelEmphasis | 53.17 | 147.37 | 0.477 | 0.002 | 0.784 | 167.7 | -127 |
| exponential_gldm_LowGrayLevelEmphasis | 43.27 | 119.95 | 0.466 | -0.015 | 0.778 | 130 | -109.9 |
| exponential_gldm_SmallDependenceEmphasis | 35.76 | 99.11 | 0.792 | 0.498 | 0.924 | 99 | -99.2 |
| exponential_gldm_SmallDependenceHighGrayLevelEmphasis | 67.80 | 187.93 | 0.929 | 0.807 | 0.975 | 186.3 | -189.5 |
| exponential_gldm_SmallDependenceLowGrayLevelEmphasis | 32.18 | 89.20 | 0.797 | 0.507 | 0.926 | 80.9 | -97.5 |
| exponential_glrlm_GrayLevelNonUniformity | 15.02 | 41.64 | 0.995 | 0.983 | 0.998 | 49.2 | -34.1 |
| exponential_glrlm_GrayLevelNonUniformityNormalized | 15.14 | 41.96 | 0.986 | 0.959 | 0.995 | 40.5 | -43.4 |
| exponential_glrlm_GrayLevelVariance | 55.91 | 154.97 | 0.963 | 0.898 | 0.987 | 152.8 | -157.2 |
| exponential_glrlm_HighGrayLevelRunEmphasis | 45.37 | 125.75 | 0.962 | 0.894 | 0.987 | 121.4 | -130.1 |
| exponential_glrlm_LongRunEmphasis | 15.17 | 42.05 | 0.824 | 0.563 | 0.936 | 49 | -35.1 |
| exponential_glrlm_LongRunHighGrayLevelEmphasis | 38.04 | 105.45 | 0.964 | 0.9 | 0.988 | 106.7 | -104.2 |
| exponential_glrlm_LongRunLowGrayLevelEmphasis | 50.62 | 140.30 | 0.308 | -0.204 | 0.695 | 156.6 | -124 |
| exponential_glrlm_LowGrayLevelRunEmphasis | 43.50 | 120.58 | 0.5 | 0.03 | 0.796 | 130.1 | -111 |
| exponential_glrlm_RunEntropy | 4.29 | 11.88 | 0.953 | 0.866 | 0.984 | 14.6 | -9.2 |
| exponential_glrlm_RunLengthNonUniformity | 15.54 | 43.07 | 0.963 | 0.896 | 0.987 | 48.5 | -37.7 |
| exponential_glrlm_RunLengthNonUniformityNormalized | 7.03 | 19.48 | 0.936 | 0.827 | 0.978 | 15.9 | -23.1 |
| exponential_glrlm_RunPercentage | 5.27 | 14.60 | 0.943 | 0.844 | 0.98 | 11.7 | -17.5 |
| exponential_glrlm_RunVariance | 28.00 | 77.61 | 0.768 | 0.452 | 0.915 | 85.3 | -69.9 |
| exponential_glrlm_ShortRunEmphasis | 4.32 | 11.98 | 0.97 | 0.916 | 0.99 | 9.8 | -14.1 |
| exponential_glrlm_ShortRunHighGrayLevelEmphasis | 47.68 | 132.15 | 0.961 | 0.893 | 0.987 | 126.2 | -138.1 |
| exponential_glrlm_ShortRunLowGrayLevelEmphasis | 41.58 | 115.27 | 0.442 | -0.058 | 0.768 | 122.5 | -108 |
| exponential_glszm_GrayLevelNonUniformity | 26.83 | 74.36 | 0.919 | 0.783 | 0.972 | 81.9 | -66.9 |
| exponential_glszm_GrayLevelNonUniformityNormalized | 33.89 | 93.93 | 0.645 | 0.23 | 0.864 | 91.5 | -96.4 |
| exponential_glszm_GrayLevelVariance | 55.84 | 154.77 | 0.979 | 0.939 | 0.993 | 153.2 | -156.3 |
| exponential_glszm_HighGrayLevelZoneEmphasis | 46.87 | 129.90 | 0.987 | 0.962 | 0.995 | 129.5 | -130.3 |
| exponential_glszm_LargeAreaEmphasis | 52.98 | 146.85 | 0.598 | 0.163 | 0.842 | 158.5 | -135.2 |
| exponential_glszm_LargeAreaHighGrayLevelEmphasis | 39.06 | 108.26 | 0.993 | 0.98 | 0.998 | 101.1 | -115.4 |
| exponential_glszm_LargeAreaLowGrayLevelEmphasis | 67.51 | 187.13 | 0.061 | -0.443 | 0.54 | 210.2 | -164.1 |
| exponential_glszm_LowGrayLevelZoneEmphasis | 42.38 | 117.46 | 0.688 | 0.302 | 0.882 | 103.3 | -131.6 |
| exponential_glszm_SizeZoneNonUniformity | 45.17 | 125.21 | 0.942 | 0.842 | 0.98 | 150.5 | -99.9 |
| exponential_glszm_SizeZoneNonUniformityNormalized | 27.11 | 75.13 | 0.507 | 0.046 | 0.798 | 95.3 | -55 |
| exponential_glszm_SmallAreaEmphasis | 73.54 | 203.84 | 0.491 | 0.005 | 0.793 | 193 | -214.6 |
| exponential_glszm_SmallAreaHighGrayLevelEmphasis | 93.95 | 260.41 | 0.981 | 0.947 | 0.994 | 266.6 | -254.2 |
| exponential_glszm_SmallAreaLowGrayLevelEmphasis | 81.54 | 226.01 | 0.581 | 0.138 | 0.835 | 172.3 | -279.7 |
| exponential_glszm_ZoneEntropy | 39.49 | 109.45 | 0.905 | 0.747 | 0.967 | 100.2 | -118.7 |
| exponential_glszm_ZonePercentage | 42.56 | 117.98 | 0.741 | 0.399 | 0.904 | 118 | -118 |
| exponential_glszm_ZoneVariance | 57.88 | 160.44 | 0.966 | 0.906 | 0.988 | 152.9 | -168 |
| exponential_ngtdm_Busyness | 57.79 | 160.18 | 0.533 | 0.062 | 0.813 | 144.9 | -175.4 |
| exponential_ngtdm_Coarseness | 40.61 | 112.57 | 0.004 | -0.494 | 0.5 | 113.7 | -111.4 |
| exponential_ngtdm_Complexity | 66.36 | 183.95 | 0.947 | 0.853 | 0.982 | 189.2 | -178.7 |
| exponential_ngtdm_Contrast | 46.52 | 128.96 | 0.883 | 0.695 | 0.959 | 112.9 | -145 |
| exponential_ngtdm_Strength | 60.41 | 167.45 | 0.983 | 0.95 | 0.994 | 170.4 | -164.5 |
| log-sigma-2-0-mm-3D_firstorder_10Percentile | 8.74 | 24.21 | 0.962 | 0.893 | 0.987 | 19 | -29.4 |
| log-sigma-2-0-mm-3D_firstorder_90Percentile | 69.43 | 192.44 | 0.97 | 0.915 | 0.99 | 198 | -186.9 |
| log-sigma-2-0-mm-3D_firstorder_Energy | 20.91 | 57.97 | 0.983 | 0.952 | 0.994 | 61.5 | -54.4 |
| log-sigma-2-0-mm-3D_firstorder_Entropy | 3.46 | 9.58 | 0.984 | 0.955 | 0.995 | 8.1 | -11.1 |
| log-sigma-2-0-mm-3D_firstorder_InterquartileRange | 10.32 | 28.62 | 0.935 | 0.808 | 0.978 | 21.4 | -35.9 |
| log-sigma-2-0-mm-3D_firstorder_Kurtosis | 10.76 | 29.84 | 0.938 | 0.83 | 0.978 | 32.4 | -27.2 |
| log-sigma-2-0-mm-3D_firstorder_Maximum | 108.53 | 300.83 | 0.973 | 0.924 | 0.991 | 245.9 | -355.8 |
| log-sigma-2-0-mm-3D_firstorder_MeanAbsoluteDeviation | 10.15 | 28.12 | 0.977 | 0.935 | 0.992 | 23.5 | -32.8 |
| log-sigma-2-0-mm-3D_firstorder_Mean | 6.38 | 17.68 | 0.94 | 0.83 | 0.98 | 12.7 | -22.7 |
| log-sigma-2-0-mm-3D_firstorder_Median | 132.85 | 368.24 | 0.946 | 0.852 | 0.981 | 312.1 | -424.4 |
| log-sigma-2-0-mm-3D_firstorder_Minimum | 11.98 | 33.20 | 0.992 | 0.977 | 0.997 | 32 | -34.4 |
| log-sigma-2-0-mm-3D_firstorder_Range | 11.79 | 32.69 | 0.992 | 0.976 | 0.997 | 31.6 | -33.7 |
| log-sigma-2-0-mm-3D_firstorder_RobustMeanAbsoluteDeviation | 9.82 | 27.21 | 0.959 | 0.885 | 0.986 | 21.9 | -32.5 |
| log-sigma-2-0-mm-3D_firstorder_RootMeanSquared | 7.40 | 20.52 | 0.981 | 0.946 | 0.994 | 16.5 | -24.6 |
| log-sigma-2-0-mm-3D_firstorder_Skewness | 93.62 | 259.49 | 0.917 | 0.777 | 0.971 | 292.5 | -226.4 |
| log-sigma-2-0-mm-3D_firstorder_TotalEnergy | 20.91 | 57.97 | 0.983 | 0.952 | 0.994 | 61.5 | -54.4 |
| log-sigma-2-0-mm-3D_firstorder_Uniformity | 7.50 | 20.79 | 0.992 | 0.976 | 0.997 | 23.2 | -18.4 |
| log-sigma-2-0-mm-3D_firstorder_Variance | 20.76 | 57.55 | 0.968 | 0.91 | 0.989 | 49.3 | -65.8 |
| log-sigma-2-0-mm-3D_glcm_Autocorrelation | 31.41 | 87.05 | 0.995 | 0.987 | 0.998 | 82.6 | -91.5 |
| log-sigma-2-0-mm-3D_glcm_ClusterProminence | 46.31 | 128.37 | 0.955 | 0.875 | 0.984 | 118.2 | -138.5 |
| log-sigma-2-0-mm-3D_glcm_ClusterShade | 49.58 | 137.44 | 0.97 | 0.917 | 0.99 | 134.4 | -140.4 |
| log-sigma-2-0-mm-3D_glcm_ClusterTendency | 22.73 | 62.99 | 0.969 | 0.914 | 0.989 | 55.4 | -70.6 |
| log-sigma-2-0-mm-3D_glcm_Contrast | 15.85 | 43.95 | 0.962 | 0.893 | 0.987 | 34.1 | -53.8 |
| log-sigma-2-0-mm-3D_glcm_Correlation | 6.42 | 17.78 | 0.871 | 0.667 | 0.954 | 18.3 | -17.3 |
| log-sigma-2-0-mm-3D_glcm_DifferenceAverage | 7.44 | 20.61 | 0.974 | 0.918 | 0.991 | 15.1 | -26.2 |
| log-sigma-2-0-mm-3D_glcm_DifferenceEntropy | 4.68 | 12.97 | 0.983 | 0.945 | 0.994 | 10.1 | -15.8 |
| log-sigma-2-0-mm-3D_glcm_DifferenceVariance | 18.28 | 50.66 | 0.975 | 0.929 | 0.991 | 42.1 | -59.2 |
| log-sigma-2-0-mm-3D_glcm_Id | 3.37 | 9.33 | 0.987 | 0.951 | 0.996 | 12.1 | -6.5 |
| log-sigma-2-0-mm-3D_glcm_Idm | 4.56 | 12.64 | 0.988 | 0.948 | 0.996 | 16.8 | -8.5 |
| log-sigma-2-0-mm-3D_glcm_Idmn | 0.43 | 1.20 | 0.834 | 0.584 | 0.94 | 1.3 | -1.1 |
| log-sigma-2-0-mm-3D_glcm_Idn | 0.86 | 2.40 | 0.896 | 0.725 | 0.963 | 2.6 | -2.2 |
| log-sigma-2-0-mm-3D_glcm_Imc1 | 8.68 | 24.05 | 0.814 | 0.534 | 0.933 | 19.2 | -28.9 |
| log-sigma-2-0-mm-3D_glcm_Imc2 | 3.12 | 8.66 | 0.924 | 0.791 | 0.974 | 7.1 | -10.2 |
| log-sigma-2-0-mm-3D_glcm_InverseVariance | 3.81 | 10.56 | 0.971 | 0.782 | 0.992 | 16.1 | -5 |
| log-sigma-2-0-mm-3D_glcm_JointAverage | 16.38 | 45.41 | 0.991 | 0.973 | 0.997 | 43 | -47.8 |
| log-sigma-2-0-mm-3D_glcm_JointEnergy | 12.34 | 34.20 | 0.996 | 0.989 | 0.999 | 37.3 | -31.1 |
| log-sigma-2-0-mm-3D_glcm_JointEntropy | 3.38 | 9.38 | 0.986 | 0.961 | 0.995 | 8.4 | -10.4 |
| log-sigma-2-0-mm-3D_glcm_MCC | 6.08 | 16.86 | 0.745 | 0.405 | 0.905 | 17.2 | -16.5 |
| log-sigma-2-0-mm-3D_glcm_MaximumProbability | 15.73 | 43.59 | 0.969 | 0.914 | 0.989 | 45.4 | -41.7 |
| log-sigma-2-0-mm-3D_glcm_SumAverage | 16.38 | 45.41 | 0.991 | 0.973 | 0.997 | 43 | -47.8 |
| log-sigma-2-0-mm-3D_glcm_SumEntropy | 3.42 | 9.49 | 0.982 | 0.947 | 0.994 | 8.4 | -10.5 |
| log-sigma-2-0-mm-3D_glcm_SumSquares | 21.07 | 58.40 | 0.969 | 0.912 | 0.989 | 50.6 | -66.2 |
| log-sigma-2-0-mm-3D_gldm_DependenceEntropy | 2.86 | 7.93 | 0.955 | 0.875 | 0.985 | 8 | -7.8 |
| log-sigma-2-0-mm-3D_gldm_DependenceNonUniformity | 11.90 | 32.99 | 0.989 | 0.968 | 0.996 | 42.1 | -23.8 |
| log-sigma-2-0-mm-3D_gldm_DependenceNonUniformityNormalized | 6.78 | 18.80 | 0.926 | 0.801 | 0.974 | 16.1 | -21.5 |
| log-sigma-2-0-mm-3D_gldm_DependenceVariance | 18.41 | 51.04 | 0.971 | 0.912 | 0.99 | 56.6 | -45.4 |
| log-sigma-2-0-mm-3D_gldm_GrayLevelNonUniformity | 10.59 | 29.36 | 0.995 | 0.981 | 0.999 | 43.6 | -15.1 |
| log-sigma-2-0-mm-3D_gldm_GrayLevelVariance | 19.62 | 54.39 | 0.967 | 0.909 | 0.989 | 46.8 | -62 |
| log-sigma-2-0-mm-3D_gldm_HighGrayLevelEmphasis | 29.69 | 82.29 | 0.996 | 0.989 | 0.999 | 77.7 | -86.9 |
| log-sigma-2-0-mm-3D_gldm_LargeDependenceEmphasis | 11.18 | 30.99 | 0.975 | 0.92 | 0.992 | 38.8 | -23.2 |
| log-sigma-2-0-mm-3D_gldm_LargeDependenceHighGrayLevelEmphasis | 35.45 | 98.27 | 0.934 | 0.816 | 0.977 | 99.4 | -97.1 |
| log-sigma-2-0-mm-3D_gldm_LargeDependenceLowGrayLevelEmphasis | 49.93 | 138.41 | 0.464 | -0.031 | 0.779 | 141 | -135.9 |
| log-sigma-2-0-mm-3D_gldm_LowGrayLevelEmphasis | 35.69 | 98.94 | 0.627 | 0.202 | 0.856 | 97.6 | -100.3 |
| log-sigma-2-0-mm-3D_gldm_SmallDependenceEmphasis | 12.50 | 34.64 | 0.936 | 0.821 | 0.978 | 24.5 | -44.7 |
| log-sigma-2-0-mm-3D_gldm_SmallDependenceHighGrayLevelEmphasis | 31.87 | 88.35 | 0.988 | 0.965 | 0.996 | 75.4 | -101.3 |
| log-sigma-2-0-mm-3D_gldm_SmallDependenceLowGrayLevelEmphasis | 34.82 | 96.50 | 0.736 | 0.391 | 0.902 | 84.8 | -108.2 |
| log-sigma-2-0-mm-3D_glrlm_GrayLevelNonUniformity | 10.30 | 28.55 | 0.997 | 0.987 | 0.999 | 42.1 | -15 |
| log-sigma-2-0-mm-3D_glrlm_GrayLevelNonUniformityNormalized | 7.73 | 21.43 | 0.983 | 0.952 | 0.994 | 24 | -18.8 |
| log-sigma-2-0-mm-3D_glrlm_GrayLevelVariance | 20.03 | 55.53 | 0.972 | 0.921 | 0.99 | 47.9 | -63.2 |
| log-sigma-2-0-mm-3D_glrlm_HighGrayLevelRunEmphasis | 29.16 | 80.83 | 0.996 | 0.989 | 0.999 | 76.3 | -85.3 |
| log-sigma-2-0-mm-3D_glrlm_LongRunEmphasis | 6.60 | 18.31 | 0.871 | 0.667 | 0.954 | 21.8 | -14.8 |
| log-sigma-2-0-mm-3D_glrlm_LongRunHighGrayLevelEmphasis | 28.34 | 78.56 | 0.989 | 0.969 | 0.996 | 77 | -80.1 |
| log-sigma-2-0-mm-3D_glrlm_LongRunLowGrayLevelEmphasis | 39.33 | 109.03 | 0.465 | -0.029 | 0.78 | 109.8 | -108.3 |
| log-sigma-2-0-mm-3D_glrlm_LowGrayLevelRunEmphasis | 34.27 | 95.00 | 0.652 | 0.242 | 0.867 | 94 | -96 |
| log-sigma-2-0-mm-3D_glrlm_RunEntropy | 2.68 | 7.43 | 0.978 | 0.939 | 0.993 | 7.1 | -7.7 |
| log-sigma-2-0-mm-3D_glrlm_RunLengthNonUniformity | 11.33 | 31.41 | 0.992 | 0.978 | 0.997 | 40.8 | -22 |
| log-sigma-2-0-mm-3D_glrlm_RunLengthNonUniformityNormalized | 1.71 | 4.75 | 0.981 | 0.922 | 0.994 | 3.2 | -6.3 |
| log-sigma-2-0-mm-3D_glrlm_RunPercentage | 1.29 | 3.57 | 0.978 | 0.924 | 0.993 | 2.6 | -4.5 |
| log-sigma-2-0-mm-3D_glrlm_RunVariance | 15.96 | 44.24 | 0.783 | 0.481 | 0.921 | 51.9 | -36.6 |
| log-sigma-2-0-mm-3D_glrlm_ShortRunEmphasis | 0.87 | 2.41 | 0.983 | 0.938 | 0.995 | 1.7 | -3.1 |
| log-sigma-2-0-mm-3D_glrlm_ShortRunHighGrayLevelEmphasis | 28.78 | 79.77 | 0.997 | 0.991 | 0.999 | 74.8 | -84.7 |
| log-sigma-2-0-mm-3D_glrlm_ShortRunLowGrayLevelEmphasis | 32.60 | 90.35 | 0.702 | 0.328 | 0.888 | 88.9 | -91.8 |
| log-sigma-2-0-mm-3D_glszm_GrayLevelNonUniformity | 26.34 | 73.02 | 0.915 | 0.772 | 0.97 | 74.1 | -72 |
| log-sigma-2-0-mm-3D_glszm_GrayLevelNonUniformityNormalized | 21.61 | 59.89 | 0.672 | 0.275 | 0.875 | 61 | -58.8 |
| log-sigma-2-0-mm-3D_glszm_GrayLevelVariance | 21.55 | 59.74 | 0.99 | 0.972 | 0.997 | 54.8 | -64.7 |
| log-sigma-2-0-mm-3D_glszm_HighGrayLevelZoneEmphasis | 23.99 | 66.50 | 0.996 | 0.988 | 0.999 | 62.5 | -70.5 |
| log-sigma-2-0-mm-3D_glszm_LargeAreaEmphasis | 29.92 | 82.95 | 0.982 | 0.945 | 0.994 | 111.2 | -54.7 |
| log-sigma-2-0-mm-3D_glszm_LargeAreaHighGrayLevelEmphasis | 35.19 | 97.54 | 0.76 | 0.435 | 0.911 | 118.8 | -76.3 |
| log-sigma-2-0-mm-3D_glszm_LargeAreaLowGrayLevelEmphasis | 50.08 | 138.82 | 0.336 | -0.183 | 0.712 | 161.5 | -116.2 |
| log-sigma-2-0-mm-3D_glszm_LowGrayLevelZoneEmphasis | 26.60 | 73.73 | 0.859 | 0.642 | 0.95 | 75.4 | -72.1 |
| log-sigma-2-0-mm-3D_glszm_SizeZoneNonUniformity | 33.20 | 92.02 | 0.956 | 0.879 | 0.985 | 77.6 | -106.5 |
| log-sigma-2-0-mm-3D_glszm_SizeZoneNonUniformityNormalized | 19.55 | 54.18 | 0.798 | 0.505 | 0.927 | 40 | -68.4 |
| log-sigma-2-0-mm-3D_glszm_SmallAreaEmphasis | 18.15 | 50.32 | 0.788 | 0.49 | 0.923 | 39.7 | -60.9 |
| log-sigma-2-0-mm-3D_glszm_SmallAreaHighGrayLevelEmphasis | 27.38 | 75.88 | 0.996 | 0.987 | 0.998 | 65.3 | -86.4 |
| log-sigma-2-0-mm-3D_glszm_SmallAreaLowGrayLevelEmphasis | 49.94 | 138.43 | 0.339 | -0.137 | 0.706 | 120.1 | -156.7 |
| log-sigma-2-0-mm-3D_glszm_ZoneEntropy | 5.40 | 14.98 | 0.969 | 0.914 | 0.989 | 15.6 | -14.3 |
| log-sigma-2-0-mm-3D_glszm_ZonePercentage | 15.87 | 44.00 | 0.943 | 0.83 | 0.981 | 31.7 | -56.4 |
| log-sigma-2-0-mm-3D_glszm_ZoneVariance | 30.40 | 84.26 | 0.988 | 0.964 | 0.996 | 113.5 | -55 |
| log-sigma-2-0-mm-3D_ngtdm_Busyness | 35.37 | 98.05 | 0.375 | -0.139 | 0.733 | 110.5 | -85.6 |
| log-sigma-2-0-mm-3D_ngtdm_Coarseness | 12.45 | 34.50 | 0.931 | 0.772 | 0.978 | 25.8 | -43.2 |
| log-sigma-2-0-mm-3D_ngtdm_Complexity | 32.26 | 89.43 | 0.985 | 0.956 | 0.995 | 79 | -99.9 |
| log-sigma-2-0-mm-3D_ngtdm_Contrast | 18.28 | 50.67 | 0.891 | 0.714 | 0.962 | 43.6 | -57.7 |
| log-sigma-2-0-mm-3D_ngtdm_Strength | 36.00 | 99.78 | 0.994 | 0.982 | 0.998 | 87.3 | -112.3 |
| log-sigma-3-0-mm-3D_firstorder_10Percentile | 7.98 | 22.11 | 0.975 | 0.93 | 0.991 | 19.4 | -24.9 |
| log-sigma-3-0-mm-3D_firstorder_90Percentile | 1934.74 | 5362.83 | 0.963 | 0.898 | 0.987 | 4852 | -5873.7 |
| log-sigma-3-0-mm-3D_firstorder_Energy | 21.11 | 58.50 | 0.984 | 0.953 | 0.994 | 64.6 | -52.4 |
| log-sigma-3-0-mm-3D_firstorder_Entropy | 3.11 | 8.61 | 0.985 | 0.957 | 0.995 | 7.9 | -9.3 |
| log-sigma-3-0-mm-3D_firstorder_InterquartileRange | 9.03 | 25.03 | 0.956 | 0.869 | 0.985 | 19.7 | -30.4 |
| log-sigma-3-0-mm-3D_firstorder_Kurtosis | 7.32 | 20.28 | 0.938 | 0.793 | 0.98 | 25.4 | -15.1 |
| log-sigma-3-0-mm-3D_firstorder_Maximum | 56.73 | 157.26 | 0.972 | 0.922 | 0.991 | 154.2 | -160.3 |
| log-sigma-3-0-mm-3D_firstorder_MeanAbsoluteDeviation | 9.90 | 27.45 | 0.981 | 0.945 | 0.993 | 25.1 | -29.8 |
| log-sigma-3-0-mm-3D_firstorder_Mean | 6.04 | 16.73 | 0.956 | 0.872 | 0.985 | 12.2 | -21.2 |
| log-sigma-3-0-mm-3D_firstorder_Median | 11.67 | 32.36 | 0.953 | 0.854 | 0.984 | 27.2 | -37.5 |
| log-sigma-3-0-mm-3D_firstorder_Minimum | 9.99 | 27.70 | 0.99 | 0.972 | 0.997 | 29.2 | -26.2 |
| log-sigma-3-0-mm-3D_firstorder_Range | 10.87 | 30.13 | 0.99 | 0.97 | 0.996 | 32.2 | -28.1 |
| log-sigma-3-0-mm-3D_firstorder_RobustMeanAbsoluteDeviation | 9.39 | 26.01 | 0.973 | 0.923 | 0.991 | 22.7 | -29.3 |
| log-sigma-3-0-mm-3D_firstorder_RootMeanSquared | 7.01 | 19.44 | 0.982 | 0.95 | 0.994 | 16.7 | -22.2 |
| log-sigma-3-0-mm-3D_firstorder_Skewness | 23.22 | 64.35 | 0.932 | 0.814 | 0.976 | 66.8 | -61.9 |
| log-sigma-3-0-mm-3D_firstorder_TotalEnergy | 21.11 | 58.50 | 0.984 | 0.953 | 0.994 | 64.6 | -52.4 |
| log-sigma-3-0-mm-3D_firstorder_Uniformity | 8.48 | 23.51 | 0.99 | 0.97 | 0.996 | 26.8 | -20.3 |
| log-sigma-3-0-mm-3D_firstorder_Variance | 20.34 | 56.37 | 0.973 | 0.924 | 0.991 | 52.7 | -60 |
| log-sigma-3-0-mm-3D_glcm_Autocorrelation | 27.36 | 75.84 | 0.995 | 0.985 | 0.998 | 82.6 | -69.1 |
| log-sigma-3-0-mm-3D_glcm_ClusterProminence | 44.27 | 122.71 | 0.964 | 0.9 | 0.988 | 123.4 | -122 |
| log-sigma-3-0-mm-3D_glcm_ClusterShade | 53.65 | 148.70 | 0.979 | 0.94 | 0.993 | 150.1 | -147.3 |
| log-sigma-3-0-mm-3D_glcm_ClusterTendency | 22.47 | 62.28 | 0.976 | 0.932 | 0.992 | 60 | -64.6 |
| log-sigma-3-0-mm-3D_glcm_Contrast | 15.84 | 43.90 | 0.965 | 0.903 | 0.988 | 39 | -48.8 |
| log-sigma-3-0-mm-3D_glcm_Correlation | 5.35 | 14.82 | 0.912 | 0.765 | 0.969 | 15.6 | -14.1 |
| log-sigma-3-0-mm-3D_glcm_DifferenceAverage | 7.62 | 21.11 | 0.978 | 0.936 | 0.992 | 17.6 | -24.6 |
| log-sigma-3-0-mm-3D_glcm_DifferenceEntropy | 3.70 | 10.25 | 0.986 | 0.961 | 0.995 | 9.2 | -11.3 |
| log-sigma-3-0-mm-3D_glcm_DifferenceVariance | 17.90 | 49.61 | 0.979 | 0.94 | 0.993 | 47.7 | -51.5 |
| log-sigma-3-0-mm-3D_glcm_Id | 4.15 | 11.49 | 0.988 | 0.962 | 0.996 | 14.4 | -8.5 |
| log-sigma-3-0-mm-3D_glcm_Idm | 5.93 | 16.45 | 0.989 | 0.964 | 0.996 | 21.1 | -11.8 |
| log-sigma-3-0-mm-3D_glcm_Idmn | 0.29 | 0.82 | 0.903 | 0.721 | 0.967 | 1 | -0.6 |
| log-sigma-3-0-mm-3D_glcm_Idn | 0.66 | 1.84 | 0.92 | 0.736 | 0.974 | 2.4 | -1.3 |
| log-sigma-3-0-mm-3D_glcm_Imc1 | 8.43 | 23.36 | 0.903 | 0.737 | 0.966 | 18.8 | -27.9 |
| log-sigma-3-0-mm-3D_glcm_Imc2 | 2.30 | 6.37 | 0.945 | 0.849 | 0.981 | 5.3 | -7.5 |
| log-sigma-3-0-mm-3D_glcm_InverseVariance | 5.64 | 15.64 | 0.979 | 0.912 | 0.994 | 21.3 | -10 |
| log-sigma-3-0-mm-3D_glcm_JointAverage | 14.06 | 38.97 | 0.989 | 0.968 | 0.996 | 42.7 | -35.3 |
| log-sigma-3-0-mm-3D_glcm_JointEnergy | 13.11 | 36.33 | 0.996 | 0.988 | 0.999 | 37.1 | -35.5 |
| log-sigma-3-0-mm-3D_glcm_JointEntropy | 2.79 | 7.73 | 0.987 | 0.963 | 0.996 | 7.8 | -7.6 |
| log-sigma-3-0-mm-3D_glcm_MCC | 4.78 | 13.26 | 0.853 | 0.627 | 0.948 | 12.8 | -13.7 |
| log-sigma-3-0-mm-3D_glcm_MaximumProbability | 15.03 | 41.66 | 0.974 | 0.925 | 0.991 | 41.2 | -42.2 |
| log-sigma-3-0-mm-3D_glcm_SumAverage | 14.06 | 38.97 | 0.989 | 0.968 | 0.996 | 42.7 | -35.3 |
| log-sigma-3-0-mm-3D_glcm_SumEntropy | 3.05 | 8.47 | 0.983 | 0.952 | 0.994 | 8.2 | -8.7 |
| log-sigma-3-0-mm-3D_glcm_SumSquares | 21.05 | 58.35 | 0.975 | 0.93 | 0.991 | 55.8 | -60.9 |
| log-sigma-3-0-mm-3D_gldm_DependenceEntropy | 2.52 | 6.98 | 0.956 | 0.877 | 0.985 | 8.1 | -5.9 |
| log-sigma-3-0-mm-3D_gldm_DependenceNonUniformity | 12.49 | 34.63 | 0.984 | 0.954 | 0.994 | 42.7 | -26.5 |
| log-sigma-3-0-mm-3D_gldm_DependenceNonUniformityNormalized | 6.57 | 18.21 | 0.943 | 0.84 | 0.98 | 14.5 | -22 |
| log-sigma-3-0-mm-3D_gldm_DependenceVariance | 16.74 | 46.40 | 0.988 | 0.963 | 0.996 | 53.2 | -39.6 |
| log-sigma-3-0-mm-3D_gldm_GrayLevelNonUniformity | 11.32 | 31.38 | 0.993 | 0.97 | 0.998 | 46.5 | -16.3 |
| log-sigma-3-0-mm-3D_gldm_GrayLevelVariance | 19.78 | 54.84 | 0.973 | 0.924 | 0.991 | 51.7 | -57.9 |
| log-sigma-3-0-mm-3D_gldm_HighGrayLevelEmphasis | 25.72 | 71.28 | 0.994 | 0.983 | 0.998 | 77.1 | -65.4 |
| log-sigma-3-0-mm-3D_gldm_LargeDependenceEmphasis | 12.31 | 34.12 | 0.974 | 0.925 | 0.991 | 41.8 | -26.4 |
| log-sigma-3-0-mm-3D_gldm_LargeDependenceHighGrayLevelEmphasis | 30.88 | 85.59 | 0.974 | 0.908 | 0.992 | 98.8 | -72.4 |
| log-sigma-3-0-mm-3D_gldm_LargeDependenceLowGrayLevelEmphasis | 53.17 | 147.38 | 0.832 | 0.581 | 0.94 | 127.9 | -166.8 |
| log-sigma-3-0-mm-3D_gldm_LowGrayLevelEmphasis | 29.19 | 80.91 | 0.928 | 0.806 | 0.975 | 62 | -99.8 |
| log-sigma-3-0-mm-3D_gldm_SmallDependenceEmphasis | 12.10 | 33.55 | 0.959 | 0.881 | 0.986 | 27.7 | -39.4 |
| log-sigma-3-0-mm-3D_gldm_SmallDependenceHighGrayLevelEmphasis | 30.55 | 84.68 | 0.98 | 0.943 | 0.993 | 84.1 | -85.3 |
| log-sigma-3-0-mm-3D_gldm_SmallDependenceLowGrayLevelEmphasis | 27.63 | 76.58 | 0.9 | 0.736 | 0.965 | 68.1 | -85.1 |
| log-sigma-3-0-mm-3D_glrlm_GrayLevelNonUniformity | 11.00 | 30.50 | 0.995 | 0.977 | 0.999 | 45 | -16 |
| log-sigma-3-0-mm-3D_glrlm_GrayLevelNonUniformityNormalized | 8.71 | 24.13 | 0.984 | 0.954 | 0.994 | 27.4 | -20.8 |
| log-sigma-3-0-mm-3D_glrlm_GrayLevelVariance | 20.02 | 55.48 | 0.975 | 0.93 | 0.992 | 52.5 | -58.4 |
| log-sigma-3-0-mm-3D_glrlm_HighGrayLevelRunEmphasis | 25.33 | 70.20 | 0.994 | 0.982 | 0.998 | 76.1 | -64.3 |
| log-sigma-3-0-mm-3D_glrlm_LongRunEmphasis | 4.66 | 12.92 | 0.93 | 0.811 | 0.976 | 14.9 | -10.9 |
| log-sigma-3-0-mm-3D_glrlm_LongRunHighGrayLevelEmphasis | 25.10 | 69.57 | 0.995 | 0.985 | 0.998 | 77.2 | -61.9 |
| log-sigma-3-0-mm-3D_glrlm_LongRunLowGrayLevelEmphasis | 32.60 | 90.36 | 0.882 | 0.692 | 0.958 | 72.5 | -108.2 |
| log-sigma-3-0-mm-3D_glrlm_LowGrayLevelRunEmphasis | 27.71 | 76.81 | 0.941 | 0.839 | 0.98 | 58.5 | -95.1 |
| log-sigma-3-0-mm-3D_glrlm_RunEntropy | 2.65 | 7.35 | 0.98 | 0.943 | 0.993 | 7.5 | -7.2 |
| log-sigma-3-0-mm-3D_glrlm_RunLengthNonUniformity | 11.68 | 32.37 | 0.993 | 0.979 | 0.997 | 42.4 | -22.3 |
| log-sigma-3-0-mm-3D_glrlm_RunLengthNonUniformityNormalized | 1.67 | 4.64 | 0.982 | 0.939 | 0.994 | 3.5 | -5.8 |
| log-sigma-3-0-mm-3D_glrlm_RunPercentage | 1.15 | 3.20 | 0.977 | 0.932 | 0.992 | 2.5 | -3.9 |
| log-sigma-3-0-mm-3D_glrlm_RunVariance | 15.95 | 44.20 | 0.908 | 0.754 | 0.968 | 50.2 | -38.2 |
| log-sigma-3-0-mm-3D_glrlm_ShortRunEmphasis | 0.89 | 2.45 | 0.979 | 0.935 | 0.993 | 1.9 | -3 |
| log-sigma-3-0-mm-3D_glrlm_ShortRunHighGrayLevelEmphasis | 25.17 | 69.76 | 0.994 | 0.982 | 0.998 | 75.2 | -64.3 |
| log-sigma-3-0-mm-3D_glrlm_ShortRunLowGrayLevelEmphasis | 26.32 | 72.95 | 0.943 | 0.844 | 0.98 | 54.8 | -91.1 |
| log-sigma-3-0-mm-3D_glszm_GrayLevelNonUniformity | 11.70 | 32.43 | 0.964 | 0.901 | 0.988 | 34.3 | -30.6 |
| log-sigma-3-0-mm-3D_glszm_GrayLevelNonUniformityNormalized | 12.39 | 34.34 | 0.903 | 0.742 | 0.966 | 31.4 | -37.2 |
| log-sigma-3-0-mm-3D_glszm_GrayLevelVariance | 20.57 | 57.01 | 0.993 | 0.979 | 0.998 | 56.6 | -57.5 |
| log-sigma-3-0-mm-3D_glszm_HighGrayLevelZoneEmphasis | 22.77 | 63.11 | 0.992 | 0.977 | 0.997 | 68 | -58.2 |
| log-sigma-3-0-mm-3D_glszm_LargeAreaEmphasis | 26.28 | 72.83 | 0.805 | 0.522 | 0.929 | 89.9 | -55.7 |
| log-sigma-3-0-mm-3D_glszm_LargeAreaHighGrayLevelEmphasis | 32.64 | 90.47 | 0.551 | 0.093 | 0.821 | 111.7 | -69.2 |
| log-sigma-3-0-mm-3D_glszm_LargeAreaLowGrayLevelEmphasis | 48.95 | 135.68 | 0.56 | 0.1 | 0.825 | 131.4 | -139.9 |
| log-sigma-3-0-mm-3D_glszm_LowGrayLevelZoneEmphasis | 19.47 | 53.97 | 0.97 | 0.909 | 0.99 | 43.6 | -64.3 |
| log-sigma-3-0-mm-3D_glszm_SizeZoneNonUniformity | 25.55 | 70.83 | 0.972 | 0.921 | 0.99 | 73.8 | -67.9 |
| log-sigma-3-0-mm-3D_glszm_SizeZoneNonUniformityNormalized | 13.39 | 37.10 | 0.899 | 0.734 | 0.965 | 35.3 | -38.9 |
| log-sigma-3-0-mm-3D_glszm_SmallAreaEmphasis | 10.81 | 29.96 | 0.844 | 0.606 | 0.944 | 30.6 | -29.3 |
| log-sigma-3-0-mm-3D_glszm_SmallAreaHighGrayLevelEmphasis | 28.35 | 78.59 | 0.987 | 0.963 | 0.996 | 83.2 | -74 |
| log-sigma-3-0-mm-3D_glszm_SmallAreaLowGrayLevelEmphasis | 40.17 | 111.34 | 0.547 | 0.081 | 0.819 | 115.3 | -107.4 |
| log-sigma-3-0-mm-3D_glszm_ZoneEntropy | 2.59 | 7.17 | 0.984 | 0.952 | 0.995 | 8.8 | -5.5 |
| log-sigma-3-0-mm-3D_glszm_ZonePercentage | 10.84 | 30.04 | 0.964 | 0.894 | 0.988 | 23 | -37.1 |
| log-sigma-3-0-mm-3D_glszm_ZoneVariance | 31.05 | 86.06 | 0.816 | 0.543 | 0.934 | 103.2 | -68.9 |
| log-sigma-3-0-mm-3D_ngtdm_Busyness | 27.58 | 76.45 | 0.741 | 0.399 | 0.904 | 77.1 | -75.8 |
| log-sigma-3-0-mm-3D_ngtdm_Coarseness | 10.85 | 30.07 | 0.941 | 0.741 | 0.982 | 20 | -40.2 |
| log-sigma-3-0-mm-3D_ngtdm_Complexity | 28.30 | 78.45 | 0.978 | 0.936 | 0.992 | 82 | -74.9 |
| log-sigma-3-0-mm-3D_ngtdm_Contrast | 13.02 | 36.09 | 0.91 | 0.659 | 0.972 | 22.2 | -50 |
| log-sigma-3-0-mm-3D_ngtdm_Strength | 27.42 | 76.01 | 0.992 | 0.977 | 0.997 | 77.6 | -74.4 |
| log-sigma-4-0-mm-3D_firstorder_10Percentile | 7.96 | 22.08 | 0.982 | 0.948 | 0.994 | 20.9 | -23.3 |
| log-sigma-4-0-mm-3D_firstorder_90Percentile | 129.05 | 357.70 | 0.977 | 0.936 | 0.992 | 306.7 | -408.7 |
| log-sigma-4-0-mm-3D_firstorder_Energy | 21.97 | 60.90 | 0.983 | 0.951 | 0.994 | 69 | -52.8 |
| log-sigma-4-0-mm-3D_firstorder_Entropy | 3.20 | 8.87 | 0.985 | 0.959 | 0.995 | 8.9 | -8.9 |
| log-sigma-4-0-mm-3D_firstorder_InterquartileRange | 10.80 | 29.92 | 0.971 | 0.917 | 0.99 | 27.7 | -32.1 |
| log-sigma-4-0-mm-3D_firstorder_Kurtosis | 4.68 | 12.97 | 0.927 | 0.532 | 0.981 | 19.5 | -6.4 |
| log-sigma-4-0-mm-3D_firstorder_Maximum | 44.30 | 122.78 | 0.933 | 0.818 | 0.977 | 129.7 | -115.9 |
| log-sigma-4-0-mm-3D_firstorder_MeanAbsoluteDeviation | 10.11 | 28.02 | 0.983 | 0.951 | 0.994 | 27.9 | -28.1 |
| log-sigma-4-0-mm-3D_firstorder_Mean | 6.20 | 17.19 | 0.964 | 0.897 | 0.988 | 13.5 | -20.9 |
| log-sigma-4-0-mm-3D_firstorder_Median | 12.21 | 33.84 | 0.887 | 0.668 | 0.962 | 23.9 | -43.8 |
| log-sigma-4-0-mm-3D_firstorder_Minimum | 9.44 | 26.16 | 0.984 | 0.955 | 0.995 | 29.5 | -22.8 |
| log-sigma-4-0-mm-3D_firstorder_Range | 11.14 | 30.88 | 0.981 | 0.945 | 0.993 | 35.2 | -26.5 |
| log-sigma-4-0-mm-3D_firstorder_RobustMeanAbsoluteDeviation | 9.57 | 26.53 | 0.981 | 0.947 | 0.994 | 25.3 | -27.8 |
| log-sigma-4-0-mm-3D_firstorder_RootMeanSquared | 7.05 | 19.53 | 0.982 | 0.95 | 0.994 | 17.8 | -21.2 |
| log-sigma-4-0-mm-3D_firstorder_Skewness | 20.22 | 56.05 | 0.95 | 0.828 | 0.984 | 61.8 | -50.3 |
| log-sigma-4-0-mm-3D_firstorder_TotalEnergy | 21.97 | 60.90 | 0.983 | 0.951 | 0.994 | 69 | -52.8 |
| log-sigma-4-0-mm-3D_firstorder_Uniformity | 8.74 | 24.23 | 0.989 | 0.969 | 0.996 | 25.5 | -23 |
| log-sigma-4-0-mm-3D_firstorder_Variance | 20.61 | 57.12 | 0.979 | 0.942 | 0.993 | 58.3 | -56 |
| log-sigma-4-0-mm-3D_glcm_Autocorrelation | 22.12 | 61.33 | 0.988 | 0.966 | 0.996 | 74 | -48.7 |
| log-sigma-4-0-mm-3D_glcm_ClusterProminence | 42.75 | 118.50 | 0.978 | 0.939 | 0.993 | 127.8 | -109.2 |
| log-sigma-4-0-mm-3D_glcm_ClusterShade | 50.92 | 141.15 | 0.988 | 0.965 | 0.996 | 149.7 | -132.6 |
| log-sigma-4-0-mm-3D_glcm_ClusterTendency | 22.55 | 62.51 | 0.983 | 0.95 | 0.994 | 65.1 | -60 |
| log-sigma-4-0-mm-3D_glcm_Contrast | 16.06 | 44.51 | 0.967 | 0.908 | 0.989 | 43.3 | -45.7 |
| log-sigma-4-0-mm-3D_glcm_Correlation | 5.02 | 13.91 | 0.921 | 0.788 | 0.973 | 15.2 | -12.7 |
| log-sigma-4-0-mm-3D_glcm_DifferenceAverage | 7.88 | 21.85 | 0.979 | 0.941 | 0.993 | 20.3 | -23.4 |
| log-sigma-4-0-mm-3D_glcm_DifferenceEntropy | 3.46 | 9.58 | 0.988 | 0.965 | 0.996 | 9.6 | -9.5 |
| log-sigma-4-0-mm-3D_glcm_DifferenceVariance | 17.30 | 47.96 | 0.983 | 0.95 | 0.994 | 49.8 | -46.1 |
| log-sigma-4-0-mm-3D_glcm_Id | 4.62 | 12.82 | 0.99 | 0.971 | 0.997 | 15 | -10.6 |
| log-sigma-4-0-mm-3D_glcm_Idm | 6.74 | 18.69 | 0.991 | 0.973 | 0.997 | 22.4 | -14.9 |
| log-sigma-4-0-mm-3D_glcm_Idmn | 0.20 | 0.55 | 0.941 | 0.8 | 0.981 | 0.7 | -0.4 |
| log-sigma-4-0-mm-3D_glcm_Idn | 0.49 | 1.36 | 0.94 | 0.755 | 0.982 | 1.8 | -0.9 |
| log-sigma-4-0-mm-3D_glcm_Imc1 | 7.33 | 20.31 | 0.938 | 0.831 | 0.979 | 17.2 | -23.4 |
| log-sigma-4-0-mm-3D_glcm_Imc2 | 2.36 | 6.54 | 0.951 | 0.864 | 0.983 | 5.7 | -7.4 |
| log-sigma-4-0-mm-3D_glcm_InverseVariance | 6.95 | 19.26 | 0.982 | 0.947 | 0.994 | 23.8 | -14.7 |
| log-sigma-4-0-mm-3D_glcm_JointAverage | 11.36 | 31.50 | 0.983 | 0.951 | 0.994 | 38.3 | -24.7 |
| log-sigma-4-0-mm-3D_glcm_JointEnergy | 14.05 | 38.94 | 0.993 | 0.981 | 0.998 | 37.2 | -40.7 |
| log-sigma-4-0-mm-3D_glcm_JointEntropy | 2.89 | 8.02 | 0.986 | 0.96 | 0.995 | 8.8 | -7.3 |
| log-sigma-4-0-mm-3D_glcm_MCC | 4.91 | 13.61 | 0.865 | 0.654 | 0.952 | 14.3 | -12.9 |
| log-sigma-4-0-mm-3D_glcm_MaximumProbability | 12.11 | 33.56 | 0.992 | 0.977 | 0.997 | 38.7 | -28.4 |
| log-sigma-4-0-mm-3D_glcm_SumAverage | 11.36 | 31.50 | 0.983 | 0.951 | 0.994 | 38.3 | -24.7 |
| log-sigma-4-0-mm-3D_glcm_SumEntropy | 3.11 | 8.62 | 0.985 | 0.957 | 0.995 | 8.7 | -8.5 |
| log-sigma-4-0-mm-3D_glcm_SumSquares | 21.26 | 58.92 | 0.982 | 0.947 | 0.994 | 61 | -56.8 |
| log-sigma-4-0-mm-3D_gldm_DependenceEntropy | 1.97 | 5.45 | 0.973 | 0.924 | 0.991 | 6.3 | -4.6 |
| log-sigma-4-0-mm-3D_gldm_DependenceNonUniformity | 14.72 | 40.81 | 0.983 | 0.951 | 0.994 | 51.8 | -29.8 |
| log-sigma-4-0-mm-3D_gldm_DependenceNonUniformityNormalized | 6.76 | 18.74 | 0.965 | 0.901 | 0.988 | 18 | -19.5 |
| log-sigma-4-0-mm-3D_gldm_DependenceVariance | 15.31 | 42.43 | 0.983 | 0.95 | 0.994 | 51.8 | -33.1 |
| log-sigma-4-0-mm-3D_gldm_GrayLevelNonUniformity | 9.58 | 26.55 | 0.989 | 0.959 | 0.996 | 39.7 | -13.4 |
| log-sigma-4-0-mm-3D_gldm_GrayLevelVariance | 20.24 | 56.11 | 0.979 | 0.941 | 0.993 | 57.5 | -54.7 |
| log-sigma-4-0-mm-3D_gldm_HighGrayLevelEmphasis | 21.18 | 58.72 | 0.987 | 0.962 | 0.995 | 70.2 | -47.2 |
| log-sigma-4-0-mm-3D_gldm_LargeDependenceEmphasis | 9.84 | 27.28 | 0.979 | 0.939 | 0.993 | 34.5 | -20.1 |
| log-sigma-4-0-mm-3D_gldm_LargeDependenceHighGrayLevelEmphasis | 22.28 | 61.76 | 0.937 | 0.799 | 0.979 | 82.3 | -41.2 |
| log-sigma-4-0-mm-3D_gldm_LargeDependenceLowGrayLevelEmphasis | 38.73 | 107.36 | 0.986 | 0.96 | 0.995 | 80.1 | -134.6 |
| log-sigma-4-0-mm-3D_gldm_LowGrayLevelEmphasis | 21.38 | 59.26 | 0.925 | 0.793 | 0.974 | 40.8 | -77.7 |
| log-sigma-4-0-mm-3D_gldm_SmallDependenceEmphasis | 7.63 | 21.15 | 0.973 | 0.923 | 0.991 | 17 | -25.3 |
| log-sigma-4-0-mm-3D_gldm_SmallDependenceHighGrayLevelEmphasis | 25.60 | 70.97 | 0.961 | 0.892 | 0.987 | 75.5 | -66.4 |
| log-sigma-4-0-mm-3D_gldm_SmallDependenceLowGrayLevelEmphasis | 25.54 | 70.79 | 0.779 | 0.474 | 0.919 | 61.1 | -80.5 |
| log-sigma-4-0-mm-3D_glrlm_GrayLevelNonUniformity | 9.25 | 25.64 | 0.993 | 0.969 | 0.998 | 38.5 | -12.8 |
| log-sigma-4-0-mm-3D_glrlm_GrayLevelNonUniformityNormalized | 8.74 | 24.23 | 0.987 | 0.963 | 0.996 | 25.6 | -22.8 |
| log-sigma-4-0-mm-3D_glrlm_GrayLevelVariance | 20.06 | 55.60 | 0.98 | 0.944 | 0.993 | 56.9 | -54.3 |
| log-sigma-4-0-mm-3D_glrlm_HighGrayLevelRunEmphasis | 21.04 | 58.33 | 0.986 | 0.961 | 0.995 | 69.7 | -47 |
| log-sigma-4-0-mm-3D_glrlm_LongRunEmphasis | 4.30 | 11.91 | 0.929 | 0.808 | 0.975 | 13.9 | -10 |
| log-sigma-4-0-mm-3D_glrlm_LongRunHighGrayLevelEmphasis | 21.19 | 58.74 | 0.99 | 0.971 | 0.997 | 72.6 | -44.9 |
| log-sigma-4-0-mm-3D_glrlm_LongRunLowGrayLevelEmphasis | 22.24 | 61.65 | 0.979 | 0.938 | 0.993 | 43.8 | -79.5 |
| log-sigma-4-0-mm-3D_glrlm_LowGrayLevelRunEmphasis | 20.93 | 58.01 | 0.928 | 0.8 | 0.975 | 40.3 | -75.8 |
| log-sigma-4-0-mm-3D_glrlm_RunEntropy | 2.50 | 6.92 | 0.984 | 0.953 | 0.994 | 7.3 | -6.5 |
| log-sigma-4-0-mm-3D_glrlm_RunLengthNonUniformity | 12.41 | 34.41 | 0.992 | 0.978 | 0.997 | 45.2 | -23.6 |
| log-sigma-4-0-mm-3D_glrlm_RunLengthNonUniformityNormalized | 1.14 | 3.15 | 0.991 | 0.974 | 0.997 | 2.6 | -3.7 |
| log-sigma-4-0-mm-3D_glrlm_RunPercentage | 0.91 | 2.51 | 0.986 | 0.958 | 0.995 | 2.1 | -3 |
| log-sigma-4-0-mm-3D_glrlm_RunVariance | 13.18 | 36.52 | 0.873 | 0.674 | 0.955 | 46.6 | -26.4 |
| log-sigma-4-0-mm-3D_glrlm_ShortRunEmphasis | 0.51 | 1.41 | 0.992 | 0.977 | 0.997 | 1.2 | -1.6 |
| log-sigma-4-0-mm-3D_glrlm_ShortRunHighGrayLevelEmphasis | 21.14 | 58.60 | 0.985 | 0.958 | 0.995 | 69.7 | -47.5 |
| log-sigma-4-0-mm-3D_glrlm_ShortRunLowGrayLevelEmphasis | 20.48 | 56.77 | 0.927 | 0.796 | 0.975 | 39.4 | -74.1 |
| log-sigma-4-0-mm-3D_glszm_GrayLevelNonUniformity | 15.47 | 42.88 | 0.964 | 0.899 | 0.988 | 45.5 | -40.3 |
| log-sigma-4-0-mm-3D_glszm_GrayLevelNonUniformityNormalized | 13.81 | 38.27 | 0.922 | 0.79 | 0.973 | 34 | -42.5 |
| log-sigma-4-0-mm-3D_glszm_GrayLevelVariance | 19.29 | 53.46 | 0.988 | 0.965 | 0.996 | 59.7 | -47.2 |
| log-sigma-4-0-mm-3D_glszm_HighGrayLevelZoneEmphasis | 19.39 | 53.74 | 0.981 | 0.945 | 0.993 | 62.3 | -45.2 |
| log-sigma-4-0-mm-3D_glszm_LargeAreaEmphasis | 22.72 | 62.99 | 0.857 | 0.635 | 0.949 | 81.1 | -44.9 |
| log-sigma-4-0-mm-3D_glszm_LargeAreaHighGrayLevelEmphasis | 27.26 | 75.55 | 0.653 | 0.251 | 0.866 | 108.3 | -42.8 |
| log-sigma-4-0-mm-3D_glszm_LargeAreaLowGrayLevelEmphasis | 33.57 | 93.06 | 0.928 | 0.806 | 0.975 | 82.1 | -104 |
| log-sigma-4-0-mm-3D_glszm_LowGrayLevelZoneEmphasis | 19.50 | 54.04 | 0.978 | 0.937 | 0.993 | 45.4 | -62.7 |
| log-sigma-4-0-mm-3D_glszm_SizeZoneNonUniformity | 27.77 | 76.96 | 0.955 | 0.876 | 0.985 | 82.7 | -71.2 |
| log-sigma-4-0-mm-3D_glszm_SizeZoneNonUniformityNormalized | 15.99 | 44.31 | 0.912 | 0.766 | 0.969 | 43.7 | -44.9 |
| log-sigma-4-0-mm-3D_glszm_SmallAreaEmphasis | 10.58 | 29.33 | 0.864 | 0.652 | 0.952 | 29.6 | -29 |
| log-sigma-4-0-mm-3D_glszm_SmallAreaHighGrayLevelEmphasis | 25.01 | 69.31 | 0.976 | 0.932 | 0.992 | 77 | -61.6 |
| log-sigma-4-0-mm-3D_glszm_SmallAreaLowGrayLevelEmphasis | 45.15 | 125.16 | 0.364 | -0.151 | 0.727 | 125.4 | -124.9 |
| log-sigma-4-0-mm-3D_glszm_ZoneEntropy | 3.95 | 10.94 | 0.975 | 0.928 | 0.991 | 12.5 | -9.3 |
| log-sigma-4-0-mm-3D_glszm_ZonePercentage | 9.06 | 25.12 | 0.974 | 0.927 | 0.991 | 20 | -30.2 |
| log-sigma-4-0-mm-3D_glszm_ZoneVariance | 28.29 | 78.41 | 0.813 | 0.542 | 0.933 | 103.2 | -53.6 |
| log-sigma-4-0-mm-3D_ngtdm_Busyness | 18.11 | 50.20 | 0.965 | 0.902 | 0.988 | 45.1 | -55.2 |
| log-sigma-4-0-mm-3D_ngtdm_Coarseness | 9.81 | 27.19 | 0.955 | 0.793 | 0.987 | 18.1 | -36.3 |
| log-sigma-4-0-mm-3D_ngtdm_Complexity | 25.39 | 70.39 | 0.975 | 0.929 | 0.991 | 78.4 | -62.4 |
| log-sigma-4-0-mm-3D_ngtdm_Contrast | 10.15 | 28.14 | 0.952 | 0.788 | 0.986 | 17.8 | -38.5 |
| log-sigma-4-0-mm-3D_ngtdm_Strength | 19.99 | 55.40 | 0.991 | 0.975 | 0.997 | 61.2 | -49.6 |
| log-sigma-5-0-mm-3D_firstorder_10Percentile | 8.32 | 23.05 | 0.982 | 0.949 | 0.994 | 23.2 | -22.9 |
| log-sigma-5-0-mm-3D_firstorder_90Percentile | 48.88 | 135.49 | 0.956 | 0.871 | 0.985 | 114.3 | -156.7 |
| log-sigma-5-0-mm-3D_firstorder_Energy | 23.25 | 64.43 | 0.98 | 0.944 | 0.993 | 74 | -54.9 |
| log-sigma-5-0-mm-3D_firstorder_Entropy | 3.46 | 9.60 | 0.986 | 0.959 | 0.995 | 10.2 | -9 |
| log-sigma-5-0-mm-3D_firstorder_InterquartileRange | 11.66 | 32.33 | 0.98 | 0.942 | 0.993 | 32.6 | -32.1 |
| log-sigma-5-0-mm-3D_firstorder_Kurtosis | 3.96 | 10.98 | 0.902 | 0.281 | 0.976 | 17.4 | -4.6 |
| log-sigma-5-0-mm-3D_firstorder_Maximum | 50.32 | 139.48 | 0.922 | 0.791 | 0.973 | 162.3 | -116.7 |
| log-sigma-5-0-mm-3D_firstorder_MeanAbsoluteDeviation | 10.81 | 29.96 | 0.982 | 0.948 | 0.994 | 31.7 | -28.2 |
| log-sigma-5-0-mm-3D_firstorder_Mean | 6.79 | 18.81 | 0.967 | 0.906 | 0.989 | 16 | -21.6 |
| log-sigma-5-0-mm-3D_firstorder_Median | 8.20 | 22.74 | 0.902 | 0.707 | 0.967 | 16.1 | -29.4 |
| log-sigma-5-0-mm-3D_firstorder_Minimum | 10.03 | 27.81 | 0.98 | 0.944 | 0.993 | 31.3 | -24.3 |
| log-sigma-5-0-mm-3D_firstorder_Range | 12.01 | 33.28 | 0.976 | 0.932 | 0.992 | 38 | -28.5 |
| log-sigma-5-0-mm-3D_firstorder_RobustMeanAbsoluteDeviation | 10.54 | 29.22 | 0.982 | 0.948 | 0.994 | 29.7 | -28.8 |
| log-sigma-5-0-mm-3D_firstorder_RootMeanSquared | 7.47 | 20.72 | 0.981 | 0.946 | 0.994 | 19.8 | -21.6 |
| log-sigma-5-0-mm-3D_firstorder_Skewness | 66.76 | 185.04 | 0.943 | 0.786 | 0.982 | 169.7 | -200.4 |
| log-sigma-5-0-mm-3D_firstorder_TotalEnergy | 23.25 | 64.43 | 0.98 | 0.944 | 0.993 | 74 | -54.9 |
| log-sigma-5-0-mm-3D_firstorder_Uniformity | 9.20 | 25.50 | 0.989 | 0.969 | 0.996 | 25.3 | -25.7 |
| log-sigma-5-0-mm-3D_firstorder_Variance | 21.49 | 59.55 | 0.985 | 0.956 | 0.995 | 64.7 | -54.4 |
| log-sigma-5-0-mm-3D_glcm_Autocorrelation | 24.69 | 68.45 | 0.988 | 0.967 | 0.996 | 83.9 | -53 |
| log-sigma-5-0-mm-3D_glcm_ClusterProminence | 43.02 | 119.25 | 0.992 | 0.978 | 0.997 | 135.5 | -103 |
| log-sigma-5-0-mm-3D_glcm_ClusterShade | 52.16 | 144.58 | 0.994 | 0.982 | 0.998 | 177 | -112.2 |
| log-sigma-5-0-mm-3D_glcm_ClusterTendency | 23.59 | 65.39 | 0.987 | 0.963 | 0.996 | 72 | -58.8 |
| log-sigma-5-0-mm-3D_glcm_Contrast | 17.71 | 49.10 | 0.969 | 0.913 | 0.989 | 50.3 | -47.9 |
| log-sigma-5-0-mm-3D_glcm_Correlation | 4.97 | 13.78 | 0.931 | 0.811 | 0.976 | 15.7 | -11.8 |
| log-sigma-5-0-mm-3D_glcm_DifferenceAverage | 8.85 | 24.54 | 0.978 | 0.939 | 0.993 | 24.4 | -24.7 |
| log-sigma-5-0-mm-3D_glcm_DifferenceEntropy | 4.14 | 11.47 | 0.985 | 0.956 | 0.995 | 12.3 | -10.6 |
| log-sigma-5-0-mm-3D_glcm_DifferenceVariance | 18.31 | 50.76 | 0.984 | 0.955 | 0.995 | 55.1 | -46.4 |
| log-sigma-5-0-mm-3D_glcm_Id | 5.39 | 14.93 | 0.989 | 0.968 | 0.996 | 16.3 | -13.6 |
| log-sigma-5-0-mm-3D_glcm_Idm | 8.18 | 22.69 | 0.989 | 0.968 | 0.996 | 25.2 | -20.1 |
| log-sigma-5-0-mm-3D_glcm_Idmn | 0.21 | 0.58 | 0.902 | 0.498 | 0.973 | 0.9 | -0.3 |
| log-sigma-5-0-mm-3D_glcm_Idn | 0.50 | 1.39 | 0.91 | 0.501 | 0.975 | 2 | -0.7 |
| log-sigma-5-0-mm-3D_glcm_Imc1 | 5.39 | 14.94 | 0.973 | 0.924 | 0.991 | 12.4 | -17.5 |
| log-sigma-5-0-mm-3D_glcm_Imc2 | 2.36 | 6.55 | 0.966 | 0.904 | 0.988 | 5.9 | -7.2 |
| log-sigma-5-0-mm-3D_glcm_InverseVariance | 7.94 | 22.00 | 0.977 | 0.934 | 0.992 | 25.2 | -18.8 |
| log-sigma-5-0-mm-3D_glcm_JointAverage | 13.06 | 36.20 | 0.977 | 0.934 | 0.992 | 44.6 | -27.8 |
| log-sigma-5-0-mm-3D_glcm_JointEnergy | 16.09 | 44.59 | 0.992 | 0.976 | 0.997 | 41.5 | -47.7 |
| log-sigma-5-0-mm-3D_glcm_JointEntropy | 3.34 | 9.26 | 0.984 | 0.955 | 0.995 | 10.3 | -8.2 |
| log-sigma-5-0-mm-3D_glcm_MCC | 3.97 | 11.00 | 0.927 | 0.801 | 0.975 | 12.5 | -9.5 |
| log-sigma-5-0-mm-3D_glcm_MaximumProbability | 12.63 | 35.01 | 0.989 | 0.969 | 0.996 | 40.3 | -29.8 |
| log-sigma-5-0-mm-3D_glcm_SumAverage | 13.06 | 36.20 | 0.977 | 0.934 | 0.992 | 44.6 | -27.8 |
| log-sigma-5-0-mm-3D_glcm_SumEntropy | 3.50 | 9.69 | 0.983 | 0.952 | 0.994 | 10.6 | -8.8 |
| log-sigma-5-0-mm-3D_glcm_SumSquares | 22.46 | 62.26 | 0.986 | 0.961 | 0.995 | 68.2 | -56.3 |
| log-sigma-5-0-mm-3D_gldm_DependenceEntropy | 2.70 | 7.47 | 0.964 | 0.898 | 0.988 | 8.4 | -6.5 |
| log-sigma-5-0-mm-3D_gldm_DependenceNonUniformity | 14.98 | 41.54 | 0.981 | 0.946 | 0.994 | 51.3 | -31.7 |
| log-sigma-5-0-mm-3D_gldm_DependenceNonUniformityNormalized | 6.40 | 17.73 | 0.953 | 0.869 | 0.984 | 15.8 | -19.7 |
| log-sigma-5-0-mm-3D_gldm_DependenceVariance | 19.24 | 53.32 | 0.993 | 0.979 | 0.998 | 60.3 | -46.3 |
| log-sigma-5-0-mm-3D_gldm_GrayLevelNonUniformity | 8.18 | 22.66 | 0.984 | 0.947 | 0.995 | 34.4 | -10.9 |
| log-sigma-5-0-mm-3D_gldm_GrayLevelVariance | 21.61 | 59.91 | 0.985 | 0.956 | 0.995 | 65 | -54.8 |
| log-sigma-5-0-mm-3D_gldm_HighGrayLevelEmphasis | 23.59 | 65.39 | 0.987 | 0.964 | 0.996 | 79.4 | -51.4 |
| log-sigma-5-0-mm-3D_gldm_LargeDependenceEmphasis | 12.14 | 33.64 | 0.991 | 0.974 | 0.997 | 38.8 | -28.5 |
| log-sigma-5-0-mm-3D_gldm_LargeDependenceHighGrayLevelEmphasis | 22.68 | 62.86 | 0.836 | 0.583 | 0.941 | 86.7 | -39 |
| log-sigma-5-0-mm-3D_gldm_LargeDependenceLowGrayLevelEmphasis | 55.89 | 154.93 | 0.99 | 0.971 | 0.997 | 130.1 | -179.8 |
| log-sigma-5-0-mm-3D_gldm_LowGrayLevelEmphasis | 31.27 | 86.69 | 0.96 | 0.878 | 0.987 | 64.9 | -108.5 |
| log-sigma-5-0-mm-3D_gldm_SmallDependenceEmphasis | 11.21 | 31.07 | 0.967 | 0.908 | 0.989 | 29.2 | -32.9 |
| log-sigma-5-0-mm-3D_gldm_SmallDependenceHighGrayLevelEmphasis | 29.82 | 82.67 | 0.96 | 0.888 | 0.986 | 92.7 | -72.7 |
| log-sigma-5-0-mm-3D_gldm_SmallDependenceLowGrayLevelEmphasis | 27.73 | 76.88 | 0.813 | 0.54 | 0.932 | 69.2 | -84.6 |
| log-sigma-5-0-mm-3D_glrlm_GrayLevelNonUniformity | 7.73 | 21.41 | 0.991 | 0.965 | 0.997 | 32.3 | -10.6 |
| log-sigma-5-0-mm-3D_glrlm_GrayLevelNonUniformityNormalized | 9.26 | 25.66 | 0.989 | 0.969 | 0.996 | 25 | -26.3 |
| log-sigma-5-0-mm-3D_glrlm_GrayLevelVariance | 21.44 | 59.42 | 0.985 | 0.958 | 0.995 | 65 | -53.9 |
| log-sigma-5-0-mm-3D_glrlm_HighGrayLevelRunEmphasis | 23.36 | 64.75 | 0.987 | 0.963 | 0.996 | 78.6 | -50.9 |
| log-sigma-5-0-mm-3D_glrlm_LongRunEmphasis | 3.70 | 10.25 | 0.951 | 0.863 | 0.983 | 12 | -8.5 |
| log-sigma-5-0-mm-3D_glrlm_LongRunHighGrayLevelEmphasis | 22.72 | 62.99 | 0.989 | 0.967 | 0.996 | 79.3 | -46.7 |
| log-sigma-5-0-mm-3D_glrlm_LongRunLowGrayLevelEmphasis | 34.91 | 96.76 | 0.985 | 0.956 | 0.995 | 74.9 | -118.6 |
| log-sigma-5-0-mm-3D_glrlm_LowGrayLevelRunEmphasis | 29.83 | 82.68 | 0.964 | 0.89 | 0.988 | 62 | -103.3 |
| log-sigma-5-0-mm-3D_glrlm_RunEntropy | 2.76 | 7.65 | 0.983 | 0.953 | 0.994 | 8.6 | -6.7 |
| log-sigma-5-0-mm-3D_glrlm_RunLengthNonUniformity | 12.90 | 35.76 | 0.993 | 0.978 | 0.997 | 46.8 | -24.7 |
| log-sigma-5-0-mm-3D_glrlm_RunLengthNonUniformityNormalized | 1.33 | 3.67 | 0.988 | 0.967 | 0.996 | 3.3 | -4.1 |
| log-sigma-5-0-mm-3D_glrlm_RunPercentage | 0.79 | 2.20 | 0.99 | 0.97 | 0.996 | 1.8 | -2.6 |
| log-sigma-5-0-mm-3D_glrlm_RunVariance | 13.87 | 38.44 | 0.878 | 0.685 | 0.957 | 46.5 | -30.4 |
| log-sigma-5-0-mm-3D_glrlm_ShortRunEmphasis | 0.57 | 1.58 | 0.991 | 0.975 | 0.997 | 1.5 | -1.7 |
| log-sigma-5-0-mm-3D_glrlm_ShortRunHighGrayLevelEmphasis | 23.57 | 65.33 | 0.986 | 0.96 | 0.995 | 79 | -51.7 |
| log-sigma-5-0-mm-3D_glrlm_ShortRunLowGrayLevelEmphasis | 28.26 | 78.34 | 0.953 | 0.861 | 0.984 | 58.6 | -98.1 |
| log-sigma-5-0-mm-3D_glszm_GrayLevelNonUniformity | 14.26 | 39.54 | 0.967 | 0.908 | 0.989 | 48.3 | -30.8 |
| log-sigma-5-0-mm-3D_glszm_GrayLevelNonUniformityNormalized | 12.72 | 35.25 | 0.979 | 0.94 | 0.993 | 36.5 | -34 |
| log-sigma-5-0-mm-3D_glszm_GrayLevelVariance | 20.68 | 57.31 | 0.988 | 0.964 | 0.996 | 65.4 | -49.2 |
| log-sigma-5-0-mm-3D_glszm_HighGrayLevelZoneEmphasis | 20.36 | 56.44 | 0.983 | 0.951 | 0.994 | 69.5 | -43.4 |
| log-sigma-5-0-mm-3D_glszm_LargeAreaEmphasis | 30.44 | 84.36 | 0.639 | 0.226 | 0.86 | 105.3 | -63.4 |
| log-sigma-5-0-mm-3D_glszm_LargeAreaHighGrayLevelEmphasis | 19.03 | 52.75 | 0.718 | 0.361 | 0.894 | 92.3 | -13.2 |
| log-sigma-5-0-mm-3D_glszm_LargeAreaLowGrayLevelEmphasis | 58.93 | 163.35 | 0.741 | 0.399 | 0.904 | 155.8 | -170.8 |
| log-sigma-5-0-mm-3D_glszm_LowGrayLevelZoneEmphasis | 21.12 | 58.54 | 0.98 | 0.941 | 0.993 | 46.8 | -70.3 |
| log-sigma-5-0-mm-3D_glszm_SizeZoneNonUniformity | 28.74 | 79.66 | 0.948 | 0.858 | 0.982 | 87.2 | -72.2 |
| log-sigma-5-0-mm-3D_glszm_SizeZoneNonUniformityNormalized | 9.87 | 27.37 | 0.952 | 0.867 | 0.983 | 28.1 | -26.6 |
| log-sigma-5-0-mm-3D_glszm_SmallAreaEmphasis | 14.65 | 40.61 | 0.906 | 0.751 | 0.967 | 45 | -36.2 |
| log-sigma-5-0-mm-3D_glszm_SmallAreaHighGrayLevelEmphasis | 26.70 | 74.01 | 0.975 | 0.93 | 0.992 | 89.2 | -58.8 |
| log-sigma-5-0-mm-3D_glszm_SmallAreaLowGrayLevelEmphasis | 47.51 | 131.69 | 0.528 | 0.055 | 0.811 | 138.9 | -124.5 |
| log-sigma-5-0-mm-3D_glszm_ZoneEntropy | 3.37 | 9.35 | 0.978 | 0.936 | 0.992 | 10.4 | -8.3 |
| log-sigma-5-0-mm-3D_glszm_ZonePercentage | 12.61 | 34.95 | 0.969 | 0.913 | 0.989 | 30.5 | -39.4 |
| log-sigma-5-0-mm-3D_glszm_ZoneVariance | 35.73 | 99.04 | 0.567 | 0.121 | 0.828 | 128.3 | -69.8 |
| log-sigma-5-0-mm-3D_ngtdm_Busyness | 19.52 | 54.12 | 0.942 | 0.841 | 0.98 | 44.1 | -64.1 |
| log-sigma-5-0-mm-3D_ngtdm_Coarseness | 10.52 | 29.16 | 0.952 | 0.833 | 0.985 | 20.9 | -37.4 |
| log-sigma-5-0-mm-3D_ngtdm_Complexity | 27.54 | 76.34 | 0.985 | 0.956 | 0.995 | 88.1 | -64.6 |
| log-sigma-5-0-mm-3D_ngtdm_Contrast | 9.13 | 25.30 | 0.956 | 0.834 | 0.986 | 16.5 | -34 |
| log-sigma-5-0-mm-3D_ngtdm_Strength | 19.64 | 54.44 | 0.988 | 0.963 | 0.996 | 62.8 | -46.1 |
| logarithm_firstorder_10Percentile | 11.86 | 32.86 | 0.307 | -0.214 | 0.696 | 30.8 | -34.9 |
| logarithm_firstorder_90Percentile | 11.61 | 32.18 | 0.769 | 0.451 | 0.915 | 28.4 | -36 |
| logarithm_firstorder_Energy | 28.29 | 78.43 | 0.98 | 0.943 | 0.993 | 83.5 | -73.3 |
| logarithm_firstorder_Entropy | 2.75 | 7.61 | 0.96 | 0.887 | 0.986 | 6.5 | -8.7 |
| logarithm_firstorder_InterquartileRange | 15.29 | 42.38 | 0.889 | 0.708 | 0.961 | 37.6 | -47.2 |
| logarithm_firstorder_Kurtosis | 4.52 | 12.52 | 0.688 | 0.303 | 0.882 | 13.8 | -11.3 |
| logarithm_firstorder_Maximum | 11.69 | 32.41 | 0.814 | 0.542 | 0.933 | 28.8 | -36 |
| logarithm_firstorder_MeanAbsoluteDeviation | 12.47 | 34.57 | 0.91 | 0.758 | 0.969 | 28.4 | -40.7 |
| logarithm_firstorder_Mean | 11.74 | 32.54 | 0.6 | 0.159 | 0.844 | 29 | -36.1 |
| logarithm_firstorder_Median | 11.68 | 32.38 | 0.557 | 0.096 | 0.824 | 28.6 | -36.2 |
| logarithm_firstorder_Minimum | 16.47 | 45.65 | 0.444 | -0.055 | 0.77 | 47.3 | -44 |
| logarithm_firstorder_Range | 11.97 | 33.17 | 0.913 | 0.76 | 0.97 | 26.5 | -39.9 |
| logarithm_firstorder_RobustMeanAbsoluteDeviation | 12.95 | 35.91 | 0.912 | 0.763 | 0.969 | 30.2 | -41.6 |
| logarithm_firstorder_RootMeanSquared | 11.71 | 32.46 | 0.632 | 0.211 | 0.858 | 28.8 | -36.1 |
| logarithm_firstorder_Skewness | 138.74 | 384.57 | 0.829 | 0.575 | 0.939 | 363.3 | -405.9 |
| logarithm_firstorder_TotalEnergy | 28.29 | 78.43 | 0.98 | 0.943 | 0.993 | 83.5 | -73.3 |
| logarithm_firstorder_Uniformity | 12.17 | 33.75 | 0.972 | 0.921 | 0.99 | 38.9 | -28.6 |
| logarithm_firstorder_Variance | 23.08 | 63.97 | 0.869 | 0.66 | 0.954 | 52 | -76 |
| logarithm_glcm_Autocorrelation | 21.25 | 58.91 | 0.777 | 0.444 | 0.92 | 43.1 | -74.7 |
| logarithm_glcm_ClusterProminence | 40.94 | 113.47 | 0.914 | 0.77 | 0.97 | 95.6 | -131.3 |
| logarithm_glcm_ClusterShade | 178.34 | 494.32 | 0.966 | 0.905 | 0.988 | 417.4 | -571.3 |
| logarithm_glcm_ClusterTendency | 24.73 | 68.56 | 0.895 | 0.724 | 0.963 | 57.7 | -79.4 |
| logarithm_glcm_Contrast | 21.80 | 60.42 | 0.754 | 0.415 | 0.91 | 45.1 | -75.7 |
| logarithm_glcm_Correlation | 3.06 | 8.48 | 0.968 | 0.902 | 0.989 | 10.2 | -6.7 |
| logarithm_glcm_DifferenceAverage | 11.58 | 32.09 | 0.855 | 0.608 | 0.949 | 23.8 | -40.3 |
| logarithm_glcm_DifferenceEntropy | 3.34 | 9.27 | 0.938 | 0.826 | 0.979 | 7.5 | -11.1 |
| logarithm_glcm_DifferenceVariance | 22.50 | 62.37 | 0.783 | 0.473 | 0.921 | 48.2 | -76.5 |
| logarithm_glcm_Id | 8.47 | 23.47 | 0.969 | 0.91 | 0.989 | 28.4 | -18.5 |
| logarithm_glcm_Idm | 12.13 | 33.62 | 0.978 | 0.938 | 0.993 | 38.6 | -28.7 |
| logarithm_glcm_Idmn | 0.20 | 0.57 | 0.943 | 0.835 | 0.981 | 0.7 | -0.4 |
| logarithm_glcm_Idn | 0.45 | 1.25 | 0.952 | 0.863 | 0.984 | 1.5 | -1 |
| logarithm_glcm_Imc1 | 6.57 | 18.20 | 0.898 | 0.505 | 0.971 | 10 | -26.4 |
| logarithm_glcm_Imc2 | 1.09 | 3.02 | 0.851 | 0.623 | 0.947 | 2.5 | -3.5 |
| logarithm_glcm_InverseVariance | 11.89 | 32.96 | 0.978 | 0.93 | 0.993 | 40.9 | -25.1 |
| logarithm_glcm_JointAverage | 11.29 | 31.29 | 0.864 | 0.612 | 0.954 | 22.6 | -40 |
| logarithm_glcm_JointEnergy | 12.99 | 35.99 | 0.951 | 0.846 | 0.984 | 27.2 | -44.8 |
| logarithm_glcm_JointEntropy | 2.10 | 5.81 | 0.976 | 0.923 | 0.992 | 7.4 | -4.2 |
| logarithm_glcm_MCC | 2.58 | 7.15 | 0.887 | 0.691 | 0.961 | 5.5 | -8.8 |
| logarithm_glcm_MaximumProbability | 18.32 | 50.78 | 0.927 | 0.801 | 0.975 | 44 | -57.6 |
| logarithm_glcm_SumAverage | 11.29 | 31.29 | 0.864 | 0.612 | 0.954 | 22.6 | -40 |
| logarithm_glcm_SumEntropy | 2.58 | 7.14 | 0.968 | 0.91 | 0.989 | 6.8 | -7.5 |
| logarithm_glcm_SumSquares | 24.06 | 66.69 | 0.882 | 0.692 | 0.958 | 55.2 | -78.2 |
| logarithm_gldm_DependenceEntropy | 2.22 | 6.15 | 0.961 | 0.891 | 0.987 | 6.1 | -6.2 |
| logarithm_gldm_DependenceNonUniformity | 15.32 | 42.46 | 0.992 | 0.977 | 0.997 | 54.2 | -30.8 |
| logarithm_gldm_DependenceNonUniformityNormalized | 8.08 | 22.40 | 0.901 | 0.737 | 0.965 | 22.3 | -22.5 |
| logarithm_gldm_DependenceVariance | 20.23 | 56.07 | 0.956 | 0.877 | 0.985 | 58.3 | -53.8 |
| logarithm_gldm_GrayLevelNonUniformity | 11.81 | 32.75 | 0.993 | 0.948 | 0.998 | 49.8 | -15.7 |
| logarithm_gldm_GrayLevelVariance | 23.02 | 63.81 | 0.869 | 0.66 | 0.954 | 51.7 | -75.9 |
| logarithm_gldm_HighGrayLevelEmphasis | 21.41 | 59.36 | 0.781 | 0.45 | 0.921 | 43.5 | -75.2 |
| logarithm_gldm_LargeDependenceEmphasis | 11.68 | 32.38 | 0.949 | 0.86 | 0.982 | 33.1 | -31.7 |
| logarithm_gldm_LargeDependenceHighGrayLevelEmphasis | 20.63 | 57.19 | 0.827 | 0.469 | 0.943 | 38 | -76.4 |
| logarithm_gldm_LargeDependenceLowGrayLevelEmphasis | 30.85 | 85.52 | 0.777 | 0.455 | 0.919 | 110 | -61.1 |
| logarithm_gldm_LowGrayLevelEmphasis | 21.70 | 60.14 | 0.912 | 0.766 | 0.969 | 62.9 | -57.4 |
| logarithm_gldm_SmallDependenceEmphasis | 6.52 | 18.09 | 0.912 | 0.765 | 0.969 | 17.6 | -18.5 |
| logarithm_gldm_SmallDependenceHighGrayLevelEmphasis | 25.76 | 71.39 | 0.78 | 0.467 | 0.92 | 57.1 | -85.7 |
| logarithm_gldm_SmallDependenceLowGrayLevelEmphasis | 23.13 | 64.12 | 0.854 | 0.627 | 0.948 | 57.1 | -71.1 |
| logarithm_glrlm_GrayLevelNonUniformity | 11.63 | 32.24 | 0.993 | 0.942 | 0.998 | 49.3 | -15.1 |
| logarithm_glrlm_GrayLevelNonUniformityNormalized | 12.06 | 33.43 | 0.973 | 0.923 | 0.991 | 38.7 | -28.2 |
| logarithm_glrlm_GrayLevelVariance | 22.88 | 63.43 | 0.87 | 0.663 | 0.954 | 51.2 | -75.6 |
| logarithm_glrlm_HighGrayLevelRunEmphasis | 21.46 | 59.48 | 0.783 | 0.454 | 0.922 | 43.7 | -75.3 |
| logarithm_glrlm_LongRunEmphasis | 1.30 | 3.61 | 0.921 | 0.788 | 0.973 | 3.8 | -3.4 |
| logarithm_glrlm_LongRunHighGrayLevelEmphasis | 21.04 | 58.32 | 0.783 | 0.45 | 0.923 | 42.4 | -74.2 |
| logarithm_glrlm_LongRunLowGrayLevelEmphasis | 21.96 | 60.87 | 0.912 | 0.766 | 0.969 | 64.7 | -57 |
| logarithm_glrlm_LowGrayLevelRunEmphasis | 21.69 | 60.12 | 0.91 | 0.761 | 0.969 | 62.5 | -57.8 |
| logarithm_glrlm_RunEntropy | 2.58 | 7.16 | 0.959 | 0.886 | 0.986 | 6.2 | -8.1 |
| logarithm_glrlm_RunLengthNonUniformity | 12.10 | 33.55 | 0.996 | 0.989 | 0.999 | 45.3 | -21.8 |
| logarithm_glrlm_RunLengthNonUniformityNormalized | 0.66 | 1.82 | 0.949 | 0.859 | 0.982 | 1.8 | -1.8 |
| logarithm_glrlm_RunPercentage | 0.37 | 1.02 | 0.944 | 0.847 | 0.981 | 1 | -1.1 |
| logarithm_glrlm_RunVariance | 17.38 | 48.19 | 0.883 | 0.696 | 0.959 | 50.9 | -45.4 |
| logarithm_glrlm_ShortRunEmphasis | 0.26 | 0.73 | 0.949 | 0.858 | 0.982 | 0.7 | -0.7 |
| logarithm_glrlm_ShortRunHighGrayLevelEmphasis | 21.60 | 59.88 | 0.782 | 0.455 | 0.922 | 44.2 | -75.6 |
| logarithm_glrlm_ShortRunLowGrayLevelEmphasis | 21.66 | 60.03 | 0.909 | 0.758 | 0.968 | 62 | -58 |
| logarithm_glszm_GrayLevelNonUniformity | 11.06 | 30.65 | 0.992 | 0.871 | 0.998 | 48.1 | -13.2 |
| logarithm_glszm_GrayLevelNonUniformityNormalized | 11.61 | 32.18 | 0.969 | 0.914 | 0.99 | 38.7 | -25.7 |
| logarithm_glszm_GrayLevelVariance | 22.16 | 61.41 | 0.885 | 0.694 | 0.96 | 47.9 | -74.9 |
| logarithm_glszm_HighGrayLevelZoneEmphasis | 22.45 | 62.23 | 0.806 | 0.503 | 0.931 | 47.4 | -77 |
| logarithm_glszm_LargeAreaEmphasis | 15.02 | 41.62 | 0.8 | 0.513 | 0.927 | 45.7 | -37.6 |
| logarithm_glszm_LargeAreaHighGrayLevelEmphasis | 23.41 | 64.89 | 0.803 | 0.507 | 0.929 | 50.4 | -79.4 |
| logarithm_glszm_LargeAreaLowGrayLevelEmphasis | 27.48 | 76.18 | 0.763 | 0.442 | 0.913 | 91.7 | -60.7 |
| logarithm_glszm_LowGrayLevelZoneEmphasis | 22.34 | 61.93 | 0.867 | 0.659 | 0.953 | 59.3 | -64.6 |
| logarithm_glszm_SizeZoneNonUniformity | 16.35 | 45.32 | 0.992 | 0.976 | 0.997 | 56.6 | -34 |
| logarithm_glszm_SizeZoneNonUniformityNormalized | 7.37 | 20.42 | 0.836 | 0.59 | 0.941 | 20.9 | -20 |
| logarithm_glszm_SmallAreaEmphasis | 3.56 | 9.87 | 0.859 | 0.639 | 0.95 | 10 | -9.7 |
| logarithm_glszm_SmallAreaHighGrayLevelEmphasis | 24.64 | 68.30 | 0.806 | 0.513 | 0.931 | 54.7 | -81.9 |
| logarithm_glszm_SmallAreaLowGrayLevelEmphasis | 25.53 | 70.77 | 0.823 | 0.563 | 0.936 | 62.4 | -79.2 |
| logarithm_glszm_ZoneEntropy | 2.48 | 6.87 | 0.952 | 0.867 | 0.983 | 6.3 | -7.5 |
| logarithm_glszm_ZonePercentage | 4.93 | 13.67 | 0.942 | 0.84 | 0.98 | 12.8 | -14.6 |
| logarithm_glszm_ZoneVariance | 26.97 | 74.76 | 0.757 | 0.428 | 0.91 | 85.7 | -63.9 |
| logarithm_ngtdm_Busyness | 21.40 | 59.32 | 0.853 | 0.597 | 0.949 | 85.6 | -33.1 |
| logarithm_ngtdm_Coarseness | 11.98 | 33.19 | 0.934 | 0.773 | 0.979 | 23.7 | -42.6 |
| logarithm_ngtdm_Complexity | 29.90 | 82.87 | 0.789 | 0.492 | 0.923 | 69.9 | -95.8 |
| logarithm_ngtdm_Contrast | 14.96 | 41.47 | 0.729 | 0.344 | 0.902 | 27.2 | -55.7 |
| logarithm_ngtdm_Strength | 19.42 | 53.82 | 0.823 | 0.53 | 0.938 | 38.4 | -69.2 |
| original_firstorder_10Percentile | 4.40 | 12.20 | 0.581 | 0.132 | 0.835 | 15.8 | -8.6 |
| original_firstorder_90Percentile | 7.05 | 19.54 | 0.978 | 0.937 | 0.992 | 20.1 | -19 |
| original_firstorder_Energy | 21.71 | 60.18 | 0.965 | 0.902 | 0.988 | 72.6 | -47.7 |
| original_firstorder_Entropy | 2.36 | 6.53 | 0.987 | 0.964 | 0.996 | 6.2 | -6.9 |
| original_firstorder_InterquartileRange | 11.33 | 31.39 | 0.971 | 0.92 | 0.99 | 30.7 | -32.1 |
| original_firstorder_Kurtosis | 6.68 | 18.51 | 0.947 | 0.853 | 0.982 | 21.2 | -15.8 |
| original_firstorder_Maximum | 8.17 | 22.63 | 0.988 | 0.965 | 0.996 | 24.2 | -21 |
| original_firstorder_MeanAbsoluteDeviation | 9.96 | 27.60 | 0.981 | 0.945 | 0.993 | 26.6 | -28.5 |
| original_firstorder_Mean | 5.77 | 15.98 | 0.966 | 0.903 | 0.988 | 16.7 | -15.3 |
| original_firstorder_Median | 5.20 | 14.42 | 0.932 | 0.816 | 0.977 | 14.9 | -14 |
| original_firstorder_Minimum | 13.17 | 36.50 | 0.704 | 0.313 | 0.89 | 45.2 | -27.8 |
| original_firstorder_Range | 10.49 | 29.07 | 0.989 | 0.97 | 0.996 | 29.3 | -28.8 |
| original_firstorder_RobustMeanAbsoluteDeviation | 9.99 | 27.70 | 0.977 | 0.934 | 0.992 | 26.6 | -28.8 |
| original_firstorder_RootMeanSquared | 6.35 | 17.60 | 0.974 | 0.926 | 0.991 | 18.2 | -17 |
| original_firstorder_Skewness | 34.71 | 96.21 | 0.936 | 0.826 | 0.978 | 91.4 | -101 |
| original_firstorder_TotalEnergy | 21.71 | 60.18 | 0.965 | 0.902 | 0.988 | 72.6 | -47.7 |
| original_firstorder_Uniformity | 9.03 | 25.03 | 0.988 | 0.966 | 0.996 | 28.7 | -21.4 |
| original_firstorder_Variance | 19.85 | 55.03 | 0.979 | 0.942 | 0.993 | 53.6 | -56.4 |
| original_glcm_Autocorrelation | 15.76 | 43.68 | 0.957 | 0.879 | 0.985 | 39 | -48.3 |
| original_glcm_ClusterProminence | 41.80 | 115.87 | 0.98 | 0.944 | 0.993 | 117.3 | -114.5 |
| original_glcm_ClusterShade | 131.59 | 364.74 | 0.988 | 0.965 | 0.996 | 328.5 | -401 |
| original_glcm_ClusterTendency | 21.35 | 59.18 | 0.982 | 0.95 | 0.994 | 58.7 | -59.7 |
| original_glcm_Contrast | 16.71 | 46.31 | 0.968 | 0.91 | 0.989 | 41.6 | -51 |
| original_glcm_Correlation | 3.14 | 8.71 | 0.971 | 0.913 | 0.99 | 10.4 | -7 |
| original_glcm_DifferenceAverage | 8.07 | 22.38 | 0.978 | 0.938 | 0.993 | 19.5 | -25.3 |
| original_glcm_DifferenceEntropy | 3.09 | 8.56 | 0.986 | 0.96 | 0.995 | 7.8 | -9.4 |
| original_glcm_DifferenceVariance | 18.60 | 51.56 | 0.983 | 0.95 | 0.994 | 48.7 | -54.4 |
| original_glcm_Id | 5.31 | 14.73 | 0.99 | 0.972 | 0.997 | 17.1 | -12.4 |
| original_glcm_Idm | 8.07 | 22.37 | 0.992 | 0.976 | 0.997 | 25.8 | -18.9 |
| original_glcm_Idmn | 0.22 | 0.61 | 0.952 | 0.868 | 0.984 | 0.7 | -0.5 |
| original_glcm_Idn | 0.52 | 1.44 | 0.955 | 0.874 | 0.984 | 1.7 | -1.2 |
| original_glcm_Imc1 | 4.71 | 13.05 | 0.962 | 0.814 | 0.989 | 8.3 | -17.8 |
| original_glcm_Imc2 | 1.10 | 3.06 | 0.985 | 0.956 | 0.995 | 2.5 | -3.6 |
| original_glcm_InverseVariance | 8.99 | 24.91 | 0.979 | 0.94 | 0.993 | 30.8 | -19 |
| original_glcm_JointAverage | 7.56 | 20.96 | 0.968 | 0.911 | 0.989 | 17.8 | -24.1 |
| original_glcm_JointEnergy | 14.88 | 41.24 | 0.986 | 0.96 | 0.995 | 38.8 | -43.7 |
| original_glcm_JointEntropy | 2.37 | 6.58 | 0.984 | 0.954 | 0.995 | 7.5 | -5.6 |
| original_glcm_MCC | 2.42 | 6.70 | 0.966 | 0.905 | 0.988 | 6.6 | -6.8 |
| original_glcm_MaximumProbability | 18.59 | 51.52 | 0.961 | 0.89 | 0.986 | 56.8 | -46.3 |
| original_glcm_SumAverage | 7.56 | 20.96 | 0.968 | 0.911 | 0.989 | 17.8 | -24.1 |
| original_glcm_SumEntropy | 2.44 | 6.75 | 0.985 | 0.958 | 0.995 | 6.9 | -6.6 |
| original_glcm_SumSquares | 20.51 | 56.84 | 0.981 | 0.947 | 0.994 | 55.7 | -57.9 |
| original_gldm_DependenceEntropy | 2.18 | 6.04 | 0.971 | 0.919 | 0.99 | 6.7 | -5.4 |
| original_gldm_DependenceNonUniformity | 13.90 | 38.52 | 0.987 | 0.962 | 0.996 | 51 | -26 |
| original_gldm_DependenceNonUniformityNormalized | 5.96 | 16.53 | 0.958 | 0.882 | 0.985 | 17.3 | -15.8 |
| original_gldm_DependenceVariance | 25.50 | 70.67 | 0.96 | 0.888 | 0.986 | 77.5 | -63.8 |
| original_gldm_GrayLevelNonUniformity | 9.87 | 27.36 | 0.981 | 0.915 | 0.994 | 42.9 | -11.9 |
| original_gldm_GrayLevelVariance | 19.63 | 54.40 | 0.98 | 0.942 | 0.993 | 53.1 | -55.7 |
| original_gldm_HighGrayLevelEmphasis | 15.88 | 44.02 | 0.958 | 0.884 | 0.986 | 39.3 | -48.7 |
| original_gldm_LargeDependenceEmphasis | 13.74 | 38.08 | 0.973 | 0.924 | 0.991 | 41.5 | -34.6 |
| original_gldm_LargeDependenceHighGrayLevelEmphasis | 15.20 | 42.13 | 0.906 | 0.748 | 0.967 | 33 | -51.3 |
| original_gldm_LargeDependenceLowGrayLevelEmphasis | 29.55 | 81.91 | 0.858 | 0.637 | 0.95 | 100.8 | -63.1 |
| original_gldm_LowGrayLevelEmphasis | 23.02 | 63.81 | 0.77 | 0.454 | 0.916 | 68 | -59.6 |
| original_gldm_SmallDependenceEmphasis | 9.19 | 25.49 | 0.971 | 0.918 | 0.99 | 24.3 | -26.7 |
| original_gldm_SmallDependenceHighGrayLevelEmphasis | 25.33 | 70.20 | 0.96 | 0.888 | 0.986 | 67.8 | -72.6 |
| original_gldm_SmallDependenceLowGrayLevelEmphasis | 22.06 | 61.14 | 0.812 | 0.538 | 0.932 | 55.6 | -66.6 |
| original_glrlm_GrayLevelNonUniformity | 9.48 | 26.27 | 0.984 | 0.922 | 0.995 | 41.3 | -11.2 |
| original_glrlm_GrayLevelNonUniformityNormalized | 8.64 | 23.95 | 0.99 | 0.972 | 0.997 | 27.3 | -20.6 |
| original_glrlm_GrayLevelVariance | 19.64 | 54.43 | 0.981 | 0.945 | 0.993 | 53 | -55.8 |
| original_glrlm_HighGrayLevelRunEmphasis | 15.98 | 44.29 | 0.961 | 0.893 | 0.987 | 39.8 | -48.8 |
| original_glrlm_LongRunEmphasis | 3.16 | 8.76 | 0.918 | 0.781 | 0.972 | 9.8 | -7.7 |
| original_glrlm_LongRunHighGrayLevelEmphasis | 14.99 | 41.56 | 0.96 | 0.889 | 0.986 | 37.3 | -45.8 |
| original_glrlm_LongRunLowGrayLevelEmphasis | 23.86 | 66.15 | 0.756 | 0.426 | 0.91 | 71.8 | -60.5 |
| original_glrlm_LowGrayLevelRunEmphasis | 23.24 | 64.41 | 0.77 | 0.454 | 0.916 | 68.3 | -60.5 |
| original_glrlm_RunEntropy | 2.16 | 5.98 | 0.986 | 0.96 | 0.995 | 5.8 | -6.1 |
| original_glrlm_RunLengthNonUniformity | 12.18 | 33.76 | 0.995 | 0.986 | 0.998 | 45.4 | -22.1 |
| original_glrlm_RunLengthNonUniformityNormalized | 0.71 | 1.97 | 0.989 | 0.97 | 0.996 | 1.9 | -2 |
| original_glrlm_RunPercentage | 0.59 | 1.62 | 0.982 | 0.947 | 0.994 | 1.5 | -1.8 |
| original_glrlm_RunVariance | 15.17 | 42.04 | 0.844 | 0.607 | 0.944 | 47.2 | -36.8 |
| original_glrlm_ShortRunEmphasis | 0.31 | 0.86 | 0.99 | 0.971 | 0.997 | 0.8 | -0.9 |
| original_glrlm_ShortRunHighGrayLevelEmphasis | 16.32 | 45.24 | 0.962 | 0.893 | 0.987 | 40.8 | -49.7 |
| original_glrlm_ShortRunLowGrayLevelEmphasis | 23.21 | 64.34 | 0.769 | 0.451 | 0.915 | 67.9 | -60.8 |
| original_glszm_GrayLevelNonUniformity | 8.63 | 23.91 | 0.996 | 0.968 | 0.999 | 34.4 | -13.4 |
| original_glszm_GrayLevelNonUniformityNormalized | 9.29 | 25.76 | 0.965 | 0.902 | 0.988 | 27.4 | -24.1 |
| original_glszm_GrayLevelVariance | 20.45 | 56.70 | 0.986 | 0.96 | 0.995 | 54.7 | -58.6 |
| original_glszm_HighGrayLevelZoneEmphasis | 19.30 | 53.49 | 0.976 | 0.934 | 0.992 | 51 | -56 |
| original_glszm_LargeAreaEmphasis | 19.32 | 53.56 | 0.849 | 0.619 | 0.946 | 69.3 | -37.8 |
| original_glszm_LargeAreaHighGrayLevelEmphasis | 15.03 | 41.65 | 0.927 | 0.801 | 0.974 | 41.6 | -41.7 |
| original_glszm_LargeAreaLowGrayLevelEmphasis | 32.81 | 90.95 | 0.756 | 0.428 | 0.91 | 114 | -67.9 |
| original_glszm_LowGrayLevelZoneEmphasis | 27.15 | 75.25 | 0.741 | 0.398 | 0.904 | 76.1 | -74.4 |
| original_glszm_SizeZoneNonUniformity | 22.05 | 61.11 | 0.977 | 0.934 | 0.992 | 69.5 | -52.7 |
| original_glszm_SizeZoneNonUniformityNormalized | 11.57 | 32.07 | 0.92 | 0.785 | 0.972 | 31.9 | -32.2 |
| original_glszm_SmallAreaEmphasis | 8.09 | 22.43 | 0.891 | 0.715 | 0.962 | 22.3 | -22.6 |
| original_glszm_SmallAreaHighGrayLevelEmphasis | 28.03 | 77.71 | 0.977 | 0.934 | 0.992 | 76.6 | -78.8 |
| original_glszm_SmallAreaLowGrayLevelEmphasis | 26.35 | 73.05 | 0.872 | 0.669 | 0.955 | 66.9 | -79.2 |
| original_glszm_ZoneEntropy | 1.94 | 5.37 | 0.983 | 0.953 | 0.994 | 6.1 | -4.6 |
| original_glszm_ZonePercentage | 8.10 | 22.45 | 0.985 | 0.956 | 0.995 | 19.4 | -25.5 |
| original_glszm_ZoneVariance | 27.97 | 77.54 | 0.869 | 0.663 | 0.954 | 102.8 | -52.3 |
| original_ngtdm_Busyness | 17.58 | 48.72 | 0.703 | 0.335 | 0.888 | 64.1 | -33.3 |
| original_ngtdm_Coarseness | 11.16 | 30.92 | 0.94 | 0.706 | 0.983 | 19.5 | -42.3 |
| original_ngtdm_Complexity | 25.97 | 72.00 | 0.969 | 0.912 | 0.989 | 71.8 | -72.2 |
| original_ngtdm_Contrast | 13.60 | 37.70 | 0.961 | 0.87 | 0.988 | 30.5 | -44.9 |
| original_ngtdm_Strength | 20.44 | 56.66 | 0.992 | 0.977 | 0.997 | 53 | -60.4 |
| square_firstorder_10Percentile | 15.89 | 44.06 | 0.585 | 0.144 | 0.836 | 56.5 | -31.6 |
| square_firstorder_90Percentile | 20.07 | 55.63 | 0.939 | 0.832 | 0.979 | 62 | -49.2 |
| square_firstorder_Energy | 39.57 | 109.68 | 0.988 | 0.964 | 0.996 | 131.5 | -87.9 |
| square_firstorder_Entropy | 7.58 | 21.00 | 0.949 | 0.86 | 0.983 | 22.3 | -19.7 |
| square_firstorder_InterquartileRange | 19.54 | 54.16 | 0.915 | 0.774 | 0.971 | 58.8 | -49.5 |
| square_firstorder_Kurtosis | 10.52 | 29.17 | 0.973 | 0.925 | 0.991 | 31.3 | -27 |
| square_firstorder_Maximum | 21.46 | 59.47 | 0.977 | 0.935 | 0.992 | 67.9 | -51.1 |
| square_firstorder_MeanAbsoluteDeviation | 21.14 | 58.60 | 0.956 | 0.878 | 0.985 | 64.2 | -53 |
| square_firstorder_Mean | 18.75 | 51.98 | 0.927 | 0.803 | 0.975 | 58.4 | -45.6 |
| square_firstorder_Median | 17.32 | 48.00 | 0.699 | 0.322 | 0.887 | 54.2 | -41.8 |
| square_firstorder_Minimum | 22.89 | 63.46 | 0.813 | 0.425 | 0.938 | 85.4 | -41.5 |
| square_firstorder_Range | 22.28 | 61.76 | 0.978 | 0.936 | 0.992 | 69.5 | -54 |
| square_firstorder_RobustMeanAbsoluteDeviation | 20.52 | 56.88 | 0.927 | 0.803 | 0.975 | 61.6 | -52.1 |
| square_firstorder_RootMeanSquared | 19.82 | 54.94 | 0.957 | 0.879 | 0.985 | 61.5 | -48.4 |
| square_firstorder_Skewness | 18.83 | 52.19 | 0.963 | 0.897 | 0.987 | 50.5 | -53.9 |
| square_firstorder_TotalEnergy | 39.57 | 109.68 | 0.988 | 0.964 | 0.996 | 131.5 | -87.9 |
| square_firstorder_Uniformity | 16.21 | 44.92 | 0.978 | 0.939 | 0.993 | 42.8 | -47.1 |
| square_firstorder_Variance | 39.31 | 108.95 | 0.97 | 0.916 | 0.99 | 119.3 | -98.6 |
| square_glcm_Autocorrelation | 34.25 | 94.94 | 0.963 | 0.897 | 0.987 | 106.6 | -83.3 |
| square_glcm_ClusterProminence | 64.07 | 177.59 | 0.955 | 0.874 | 0.984 | 198.7 | -156.5 |
| square_glcm_ClusterShade | 62.23 | 172.50 | 0.969 | 0.912 | 0.989 | 198.8 | -146.2 |
| square_glcm_ClusterTendency | 39.68 | 109.99 | 0.972 | 0.92 | 0.99 | 121.6 | -98.4 |
| square_glcm_Contrast | 37.16 | 103.00 | 0.957 | 0.879 | 0.985 | 110.2 | -95.8 |
| square_glcm_Correlation | 4.46 | 12.37 | 0.978 | 0.933 | 0.993 | 14.8 | -9.9 |
| square_glcm_DifferenceAverage | 20.41 | 56.58 | 0.935 | 0.823 | 0.977 | 59.7 | -53.4 |
| square_glcm_DifferenceEntropy | 8.81 | 24.43 | 0.955 | 0.875 | 0.984 | 25.4 | -23.5 |
| square_glcm_DifferenceVariance | 37.67 | 104.42 | 0.969 | 0.914 | 0.989 | 113.5 | -95.4 |
| square_glcm_Id | 9.48 | 26.29 | 0.957 | 0.88 | 0.985 | 25 | -27.6 |
| square_glcm_Idm | 12.47 | 34.57 | 0.96 | 0.89 | 0.986 | 33 | -36.2 |
| square_glcm_Idmn | 0.34 | 0.96 | 0.916 | 0.757 | 0.972 | 1.2 | -0.7 |
| square_glcm_Idn | 0.52 | 1.43 | 0.948 | 0.8 | 0.984 | 1.9 | -1 |
| square_glcm_Imc1 | 8.08 | 22.40 | 0.946 | 0.853 | 0.982 | 25.9 | -18.9 |
| square_glcm_Imc2 | 3.79 | 10.50 | 0.983 | 0.951 | 0.994 | 11.5 | -9.5 |
| square_glcm_InverseVariance | 10.17 | 28.20 | 0.923 | 0.793 | 0.973 | 25.9 | -30.5 |
| square_glcm_JointAverage | 17.77 | 49.26 | 0.924 | 0.795 | 0.974 | 55.2 | -43.3 |
| square_glcm_JointEnergy | 28.72 | 79.60 | 0.985 | 0.958 | 0.995 | 78.3 | -80.9 |
| square_glcm_JointEntropy | 7.02 | 19.46 | 0.964 | 0.9 | 0.988 | 20.2 | -18.7 |
| square_glcm_MCC | 3.41 | 9.45 | 0.983 | 0.937 | 0.995 | 12.4 | -6.5 |
| square_glcm_MaximumProbability | 25.78 | 71.45 | 0.981 | 0.947 | 0.994 | 72.6 | -70.3 |
| square_glcm_SumAverage | 17.77 | 49.26 | 0.924 | 0.795 | 0.974 | 55.2 | -43.3 |
| square_glcm_SumEntropy | 6.56 | 18.20 | 0.963 | 0.898 | 0.987 | 19.2 | -17.2 |
| square_glcm_SumSquares | 39.21 | 108.69 | 0.97 | 0.916 | 0.99 | 119.5 | -97.9 |
| square_gldm_DependenceEntropy | 2.91 | 8.06 | 0.958 | 0.878 | 0.986 | 9.7 | -6.5 |
| square_gldm_DependenceNonUniformity | 17.77 | 49.26 | 0.945 | 0.848 | 0.981 | 61.5 | -37 |
| square_gldm_DependenceNonUniformityNormalized | 14.15 | 39.23 | 0.743 | 0.402 | 0.905 | 39.8 | -38.7 |
| square_gldm_DependenceVariance | 30.28 | 83.93 | 0.885 | 0.698 | 0.96 | 96 | -71.8 |
| square_gldm_GrayLevelNonUniformity | 19.07 | 52.87 | 0.951 | 0.858 | 0.983 | 62.6 | -43.1 |
| square_gldm_GrayLevelVariance | 38.52 | 106.77 | 0.97 | 0.916 | 0.99 | 117.3 | -96.3 |
| square_gldm_HighGrayLevelEmphasis | 34.31 | 95.11 | 0.965 | 0.901 | 0.988 | 106.6 | -83.6 |
| square_gldm_LargeDependenceEmphasis | 25.49 | 70.66 | 0.95 | 0.86 | 0.983 | 76.2 | -65.1 |
| square_gldm_LargeDependenceHighGrayLevelEmphasis | 16.85 | 46.71 | 0.949 | 0.858 | 0.982 | 56.9 | -36.5 |
| square_gldm_LargeDependenceLowGrayLevelEmphasis | 38.17 | 105.79 | 0.948 | 0.856 | 0.982 | 98.2 | -113.3 |
| square_gldm_LowGrayLevelEmphasis | 20.01 | 55.47 | 0.778 | 0.454 | 0.92 | 42.6 | -68.3 |
| square_gldm_SmallDependenceEmphasis | 23.27 | 64.49 | 0.877 | 0.681 | 0.956 | 69.4 | -59.6 |
| square_gldm_SmallDependenceHighGrayLevelEmphasis | 50.91 | 141.12 | 0.966 | 0.905 | 0.988 | 152.6 | -129.7 |
| square_gldm_SmallDependenceLowGrayLevelEmphasis | 18.34 | 50.85 | 0.342 | -0.171 | 0.715 | 56.6 | -45.1 |
| square_glrlm_GrayLevelNonUniformity | 15.32 | 42.46 | 0.989 | 0.966 | 0.996 | 48.7 | -36.2 |
| square_glrlm_GrayLevelNonUniformityNormalized | 15.03 | 41.67 | 0.978 | 0.938 | 0.993 | 37.3 | -46 |
| square_glrlm_GrayLevelVariance | 37.95 | 105.19 | 0.976 | 0.932 | 0.992 | 117.3 | -93 |
| square_glrlm_HighGrayLevelRunEmphasis | 33.85 | 93.83 | 0.973 | 0.923 | 0.991 | 106 | -81.6 |
| square_glrlm_LongRunEmphasis | 11.24 | 31.16 | 0.872 | 0.67 | 0.955 | 34.7 | -27.6 |
| square_glrlm_LongRunHighGrayLevelEmphasis | 30.98 | 85.88 | 0.973 | 0.923 | 0.991 | 100.9 | -70.8 |
| square_glrlm_LongRunLowGrayLevelEmphasis | 28.87 | 80.03 | 0.904 | 0.745 | 0.966 | 69.5 | -90.6 |
| square_glrlm_LowGrayLevelRunEmphasis | 19.48 | 53.99 | 0.886 | 0.69 | 0.96 | 42.1 | -65.9 |
| square_glrlm_RunEntropy | 4.54 | 12.57 | 0.951 | 0.858 | 0.983 | 15.3 | -9.8 |
| square_glrlm_RunLengthNonUniformity | 14.19 | 39.34 | 0.972 | 0.922 | 0.991 | 48.8 | -29.9 |
| square_glrlm_RunLengthNonUniformityNormalized | 4.35 | 12.07 | 0.965 | 0.901 | 0.988 | 11.1 | -13 |
| square_glrlm_RunPercentage | 3.48 | 9.64 | 0.955 | 0.874 | 0.984 | 8.3 | -10.9 |
| square_glrlm_RunVariance | 26.95 | 74.71 | 0.79 | 0.496 | 0.924 | 77.9 | -71.5 |
| square_glrlm_ShortRunEmphasis | 1.99 | 5.52 | 0.977 | 0.935 | 0.992 | 5.1 | -5.9 |
| square_glrlm_ShortRunHighGrayLevelEmphasis | 35.24 | 97.69 | 0.973 | 0.923 | 0.991 | 110 | -85.4 |
| square_glrlm_ShortRunLowGrayLevelEmphasis | 18.19 | 50.43 | 0.85 | 0.601 | 0.948 | 38.4 | -62.4 |
| square_glszm_GrayLevelNonUniformity | 26.71 | 74.05 | 0.945 | 0.813 | 0.982 | 94.7 | -53.4 |
| square_glszm_GrayLevelNonUniformityNormalized | 23.28 | 64.53 | 0.948 | 0.856 | 0.982 | 66.4 | -62.6 |
| square_glszm_GrayLevelVariance | 38.69 | 107.25 | 0.982 | 0.948 | 0.994 | 119.2 | -95.3 |
| square_glszm_HighGrayLevelZoneEmphasis | 33.65 | 93.27 | 0.982 | 0.949 | 0.994 | 99.8 | -86.7 |
| square_glszm_LargeAreaEmphasis | 44.11 | 122.26 | 0.9 | 0.735 | 0.965 | 127.6 | -116.9 |
| square_glszm_LargeAreaHighGrayLevelEmphasis | 23.11 | 64.06 | 0.999 | 0.998 | 1 | 71.9 | -56.2 |
| square_glszm_LargeAreaLowGrayLevelEmphasis | 51.42 | 142.53 | 0.882 | 0.693 | 0.958 | 135.6 | -149.4 |
| square_glszm_LowGrayLevelZoneEmphasis | 16.13 | 44.70 | 0.948 | 0.847 | 0.982 | 53.3 | -36.1 |
| square_glszm_SizeZoneNonUniformity | 40.52 | 112.30 | 0.954 | 0.842 | 0.985 | 133 | -91.6 |
| square_glszm_SizeZoneNonUniformityNormalized | 19.87 | 55.08 | 0.936 | 0.823 | 0.978 | 58.5 | -51.7 |
| square_glszm_SmallAreaEmphasis | 39.91 | 110.63 | 0.869 | 0.66 | 0.954 | 131.9 | -89.4 |
| square_glszm_SmallAreaHighGrayLevelEmphasis | 64.05 | 177.54 | 0.982 | 0.948 | 0.994 | 195.8 | -159.2 |
| square_glszm_SmallAreaLowGrayLevelEmphasis | 39.73 | 110.11 | 0.607 | 0.174 | 0.846 | 141.8 | -78.4 |
| square_glszm_ZoneEntropy | 6.93 | 19.20 | 0.966 | 0.905 | 0.988 | 20.7 | -17.7 |
| square_glszm_ZonePercentage | 25.33 | 70.21 | 0.868 | 0.661 | 0.953 | 77.3 | -63.1 |
| square_glszm_ZoneVariance | 43.91 | 121.72 | 0.891 | 0.713 | 0.962 | 133 | -110.4 |
| square_ngtdm_Busyness | 29.95 | 83.02 | 0.987 | 0.962 | 0.996 | 73.6 | -92.4 |
| square_ngtdm_Coarseness | 12.92 | 35.82 | 0.92 | 0.759 | 0.973 | 25.7 | -45.9 |
| square_ngtdm_Complexity | 48.37 | 134.08 | 0.979 | 0.941 | 0.993 | 153.1 | -115 |
| square_ngtdm_Contrast | 27.21 | 75.42 | 0.858 | 0.638 | 0.95 | 70.4 | -80.5 |
| square_ngtdm_Strength | 40.06 | 111.05 | 0.989 | 0.965 | 0.996 | 124.7 | -97.5 |
| squareroot_firstorder_10Percentile | 8.38 | 23.22 | 0.343 | -0.175 | 0.716 | 22.2 | -24.2 |
| squareroot_firstorder_90Percentile | 8.57 | 23.74 | 0.902 | 0.742 | 0.966 | 21.3 | -26.2 |
| squareroot_firstorder_Energy | 24.22 | 67.13 | 0.979 | 0.941 | 0.993 | 74.2 | -60.1 |
| squareroot_firstorder_Entropy | 2.22 | 6.17 | 0.98 | 0.943 | 0.993 | 5.3 | -7 |
| squareroot_firstorder_InterquartileRange | 13.39 | 37.12 | 0.936 | 0.826 | 0.978 | 33.6 | -40.6 |
| squareroot_firstorder_Kurtosis | 4.99 | 13.83 | 0.85 | 0.62 | 0.946 | 16.1 | -11.6 |
| squareroot_firstorder_Maximum | 8.85 | 24.54 | 0.937 | 0.829 | 0.978 | 22.6 | -26.5 |
| squareroot_firstorder_MeanAbsoluteDeviation | 10.72 | 29.71 | 0.957 | 0.881 | 0.985 | 25.3 | -34.1 |
| squareroot_firstorder_Mean | 8.47 | 23.48 | 0.776 | 0.466 | 0.918 | 21.1 | -25.8 |
| squareroot_firstorder_Median | 8.37 | 23.21 | 0.696 | 0.317 | 0.886 | 20.7 | -25.8 |
| squareroot_firstorder_Minimum | 11.95 | 33.13 | 0.468 | -0.026 | 0.781 | 34.8 | -31.5 |
| squareroot_firstorder_Range | 10.45 | 28.97 | 0.968 | 0.909 | 0.989 | 24.9 | -33.1 |
| squareroot_firstorder_RobustMeanAbsoluteDeviation | 11.09 | 30.73 | 0.954 | 0.872 | 0.984 | 26.5 | -34.9 |
| squareroot_firstorder_RootMeanSquared | 8.49 | 23.54 | 0.814 | 0.542 | 0.933 | 21.1 | -26 |
| squareroot_firstorder_Skewness | 75.29 | 208.68 | 0.895 | 0.725 | 0.963 | 232.9 | -184.4 |
| squareroot_firstorder_TotalEnergy | 24.22 | 67.13 | 0.979 | 0.941 | 0.993 | 74.2 | -60.1 |
| squareroot_firstorder_Uniformity | 9.57 | 26.54 | 0.987 | 0.962 | 0.996 | 31.6 | -21.5 |
| squareroot_firstorder_Variance | 20.74 | 57.49 | 0.953 | 0.869 | 0.984 | 49 | -66 |
| squareroot_glcm_Autocorrelation | 17.81 | 49.35 | 0.883 | 0.681 | 0.96 | 38.4 | -60.3 |
| squareroot_glcm_ClusterProminence | 41.97 | 116.34 | 0.981 | 0.946 | 0.993 | 103.9 | -128.8 |
| squareroot_glcm_ClusterShade | 67.10 | 185.98 | 0.989 | 0.969 | 0.996 | 200.8 | -171.1 |
| squareroot_glcm_ClusterTendency | 22.56 | 62.53 | 0.963 | 0.895 | 0.987 | 54.8 | -70.3 |
| squareroot_glcm_Contrast | 18.58 | 51.49 | 0.908 | 0.743 | 0.968 | 39.4 | -63.6 |
| squareroot_glcm_Correlation | 3.01 | 8.36 | 0.971 | 0.912 | 0.991 | 10.1 | -6.6 |
| squareroot_glcm_DifferenceAverage | 9.29 | 25.76 | 0.935 | 0.806 | 0.978 | 19.1 | -32.4 |
| squareroot_glcm_DifferenceEntropy | 3.07 | 8.51 | 0.969 | 0.912 | 0.99 | 7 | -10 |
| squareroot_glcm_DifferenceVariance | 19.86 | 55.05 | 0.94 | 0.831 | 0.979 | 44.9 | -65.2 |
| squareroot_glcm_Id | 6.63 | 18.37 | 0.983 | 0.946 | 0.994 | 22.8 | -13.9 |
| squareroot_glcm_Idm | 9.92 | 27.49 | 0.988 | 0.964 | 0.996 | 32.7 | -22.3 |
| squareroot_glcm_Idmn | 0.15 | 0.41 | 0.965 | 0.847 | 0.989 | 0.6 | -0.3 |
| squareroot_glcm_Idn | 0.37 | 1.02 | 0.964 | 0.856 | 0.989 | 1.4 | -0.7 |
| squareroot_glcm_Imc1 | 4.97 | 13.78 | 0.935 | 0.541 | 0.983 | 6.7 | -20.9 |
| squareroot_glcm_Imc2 | 1.37 | 3.80 | 0.93 | 0.81 | 0.976 | 3.2 | -4.4 |
| squareroot_glcm_InverseVariance | 10.26 | 28.43 | 0.985 | 0.956 | 0.995 | 35.6 | -21.3 |
| squareroot_glcm_JointAverage | 9.07 | 25.14 | 0.925 | 0.772 | 0.975 | 18.9 | -31.4 |
| squareroot_glcm_JointEnergy | 13.03 | 36.11 | 0.978 | 0.935 | 0.993 | 30.3 | -41.9 |
| squareroot_glcm_JointEntropy | 2.13 | 5.90 | 0.98 | 0.943 | 0.993 | 7.1 | -4.7 |
| squareroot_glcm_MCC | 2.97 | 8.22 | 0.913 | 0.768 | 0.97 | 7.8 | -8.7 |
| squareroot_glcm_MaximumProbability | 17.48 | 48.45 | 0.964 | 0.901 | 0.988 | 48.4 | -48.5 |
| squareroot_glcm_SumAverage | 9.07 | 25.14 | 0.925 | 0.772 | 0.975 | 18.9 | -31.4 |
| squareroot_glcm_SumEntropy | 2.48 | 6.88 | 0.978 | 0.937 | 0.992 | 6.5 | -7.2 |
| squareroot_glcm_SumSquares | 21.76 | 60.32 | 0.959 | 0.885 | 0.986 | 52 | -68.7 |
| squareroot_gldm_DependenceEntropy | 1.86 | 5.16 | 0.974 | 0.926 | 0.991 | 5.5 | -4.8 |
| squareroot_gldm_DependenceNonUniformity | 14.09 | 39.05 | 0.99 | 0.973 | 0.997 | 49.5 | -28.6 |
| squareroot_gldm_DependenceNonUniformityNormalized | 7.47 | 20.70 | 0.929 | 0.807 | 0.975 | 19.4 | -22 |
| squareroot_gldm_DependenceVariance | 20.85 | 57.80 | 0.935 | 0.822 | 0.978 | 63 | -52.6 |
| squareroot_gldm_GrayLevelNonUniformity | 9.96 | 27.60 | 0.989 | 0.927 | 0.997 | 44.5 | -10.7 |
| squareroot_gldm_GrayLevelVariance | 20.71 | 57.42 | 0.953 | 0.868 | 0.984 | 48.8 | -66.1 |
| squareroot_gldm_HighGrayLevelEmphasis | 17.98 | 49.83 | 0.885 | 0.685 | 0.96 | 38.8 | -60.8 |
| squareroot_gldm_LargeDependenceEmphasis | 13.05 | 36.17 | 0.917 | 0.778 | 0.971 | 39.3 | -33 |
| squareroot_gldm_LargeDependenceHighGrayLevelEmphasis | 20.51 | 56.85 | 0.865 | 0.585 | 0.955 | 43.3 | -70.5 |
| squareroot_gldm_LargeDependenceLowGrayLevelEmphasis | 27.92 | 77.39 | 0.885 | 0.695 | 0.96 | 99.4 | -55.4 |
| squareroot_gldm_LowGrayLevelEmphasis | 21.08 | 58.42 | 0.926 | 0.8 | 0.974 | 68.6 | -48.3 |
| squareroot_gldm_SmallDependenceEmphasis | 9.43 | 26.14 | 0.93 | 0.81 | 0.976 | 23.9 | -28.4 |
| squareroot_gldm_SmallDependenceHighGrayLevelEmphasis | 23.41 | 64.89 | 0.896 | 0.723 | 0.964 | 53.7 | -76.1 |
| squareroot_gldm_SmallDependenceLowGrayLevelEmphasis | 23.25 | 64.45 | 0.88 | 0.689 | 0.958 | 68.1 | -60.8 |
| squareroot_glrlm_GrayLevelNonUniformity | 9.72 | 26.94 | 0.99 | 0.926 | 0.997 | 43.5 | -10.4 |
| squareroot_glrlm_GrayLevelNonUniformityNormalized | 9.45 | 26.19 | 0.988 | 0.965 | 0.996 | 31.1 | -21.3 |
| squareroot_glrlm_GrayLevelVariance | 20.53 | 56.91 | 0.953 | 0.87 | 0.984 | 48.3 | -65.6 |
| squareroot_glrlm_HighGrayLevelRunEmphasis | 18.00 | 49.89 | 0.888 | 0.693 | 0.961 | 39 | -60.8 |
| squareroot_glrlm_LongRunEmphasis | 2.34 | 6.49 | 0.871 | 0.668 | 0.954 | 7.3 | -5.7 |
| squareroot_glrlm_LongRunHighGrayLevelEmphasis | 17.96 | 49.79 | 0.886 | 0.683 | 0.961 | 39.3 | -60.3 |
| squareroot_glrlm_LongRunLowGrayLevelEmphasis | 21.08 | 58.42 | 0.929 | 0.808 | 0.975 | 69.7 | -47.1 |
| squareroot_glrlm_LowGrayLevelRunEmphasis | 21.19 | 58.73 | 0.923 | 0.792 | 0.973 | 68.7 | -48.8 |
| squareroot_glrlm_RunEntropy | 2.14 | 5.92 | 0.979 | 0.94 | 0.993 | 5.3 | -6.5 |
| squareroot_glrlm_RunLengthNonUniformity | 11.88 | 32.94 | 0.996 | 0.988 | 0.999 | 44.4 | -21.5 |
| squareroot_glrlm_RunLengthNonUniformityNormalized | 0.81 | 2.25 | 0.96 | 0.888 | 0.986 | 2 | -2.5 |
| squareroot_glrlm_RunPercentage | 0.56 | 1.54 | 0.939 | 0.834 | 0.979 | 1.4 | -1.7 |
| squareroot_glrlm_RunVariance | 19.60 | 54.34 | 0.796 | 0.506 | 0.926 | 59.4 | -49.3 |
| squareroot_glrlm_ShortRunEmphasis | 0.35 | 0.97 | 0.955 | 0.874 | 0.984 | 0.9 | -1.1 |
| squareroot_glrlm_ShortRunHighGrayLevelEmphasis | 18.08 | 50.12 | 0.888 | 0.695 | 0.961 | 39.2 | -61 |
| squareroot_glrlm_ShortRunLowGrayLevelEmphasis | 21.29 | 59.00 | 0.92 | 0.784 | 0.972 | 68.7 | -49.3 |
| squareroot_glszm_GrayLevelNonUniformity | 10.56 | 29.28 | 0.995 | 0.943 | 0.999 | 42.1 | -16.5 |
| squareroot_glszm_GrayLevelNonUniformityNormalized | 10.16 | 28.16 | 0.974 | 0.926 | 0.991 | 31.8 | -24.5 |
| squareroot_glszm_GrayLevelVariance | 20.18 | 55.94 | 0.961 | 0.89 | 0.987 | 47.5 | -64.4 |
| squareroot_glszm_HighGrayLevelZoneEmphasis | 19.24 | 53.34 | 0.915 | 0.764 | 0.971 | 43.7 | -63 |
| squareroot_glszm_LargeAreaEmphasis | 19.31 | 53.54 | 0.742 | 0.403 | 0.904 | 64.1 | -42.9 |
| squareroot_glszm_LargeAreaHighGrayLevelEmphasis | 24.58 | 68.13 | 0.895 | 0.724 | 0.963 | 62.3 | -73.9 |
| squareroot_glszm_LargeAreaLowGrayLevelEmphasis | 27.45 | 76.09 | 0.683 | 0.297 | 0.88 | 100.3 | -51.9 |
| squareroot_glszm_LowGrayLevelZoneEmphasis | 22.35 | 61.96 | 0.896 | 0.725 | 0.963 | 70.2 | -53.7 |
| squareroot_glszm_SizeZoneNonUniformity | 19.73 | 54.68 | 0.991 | 0.974 | 0.997 | 62.1 | -47.3 |
| squareroot_glszm_SizeZoneNonUniformityNormalized | 10.73 | 29.74 | 0.86 | 0.643 | 0.95 | 28.1 | -31.4 |
| squareroot_glszm_SmallAreaEmphasis | 5.76 | 15.98 | 0.83 | 0.576 | 0.939 | 15.1 | -16.8 |
| squareroot_glszm_SmallAreaHighGrayLevelEmphasis | 23.16 | 64.20 | 0.922 | 0.784 | 0.973 | 54.4 | -74 |
| squareroot_glszm_SmallAreaLowGrayLevelEmphasis | 24.15 | 66.94 | 0.883 | 0.695 | 0.959 | 71.6 | -62.3 |
| squareroot_glszm_ZoneEntropy | 2.30 | 6.38 | 0.966 | 0.905 | 0.988 | 6.6 | -6.2 |
| squareroot_glszm_ZonePercentage | 7.59 | 21.05 | 0.948 | 0.857 | 0.982 | 18.3 | -23.8 |
| squareroot_glszm_ZoneVariance | 30.63 | 84.91 | 0.745 | 0.41 | 0.906 | 102.8 | -67 |
| squareroot_ngtdm_Busyness | 18.12 | 50.24 | 0.859 | 0.631 | 0.95 | 71.7 | -28.8 |
| squareroot_ngtdm_Coarseness | 12.20 | 33.81 | 0.929 | 0.745 | 0.978 | 23.6 | -44 |
| squareroot_ngtdm_Complexity | 25.97 | 71.98 | 0.925 | 0.796 | 0.974 | 63.2 | -80.7 |
| squareroot_ngtdm_Contrast | 14.74 | 40.86 | 0.827 | 0.521 | 0.941 | 28.2 | -53.5 |
| squareroot_ngtdm_Strength | 15.88 | 44.03 | 0.957 | 0.874 | 0.986 | 33.3 | -54.7 |
| wavelet-LLH_firstorder_10Percentile | 6.06 | 16.80 | 0.965 | 0.823 | 0.99 | 7.9 | -25.7 |
| wavelet-LLH_firstorder_90Percentile | 27.31 | 75.70 | 0.984 | 0.953 | 0.994 | 79.6 | -71.8 |
| wavelet-LLH_firstorder_Energy | 14.05 | 38.94 | 0.982 | 0.95 | 0.994 | 37.3 | -40.6 |
| wavelet-LLH_firstorder_Entropy | 4.81 | 13.32 | 0.988 | 0.955 | 0.996 | 10.1 | -16.5 |
| wavelet-LLH_firstorder_InterquartileRange | 8.79 | 24.35 | 0.98 | 0.943 | 0.993 | 19.6 | -29.1 |
| wavelet-LLH_firstorder_Kurtosis | 6.45 | 17.88 | 0.961 | 0.89 | 0.986 | 17 | -18.7 |
| wavelet-LLH_firstorder_Maximum | 10.93 | 30.29 | 0.992 | 0.978 | 0.997 | 28.3 | -32.3 |
| wavelet-LLH_firstorder_MeanAbsoluteDeviation | 7.06 | 19.56 | 0.983 | 0.936 | 0.995 | 13.4 | -25.7 |
| wavelet-LLH_firstorder_Mean | 10.46 | 28.99 | 0.923 | 0.727 | 0.976 | 18.7 | -39.2 |
| wavelet-LLH_firstorder_Median | 25.19 | 69.83 | 0.776 | 0.467 | 0.918 | 55.9 | -83.8 |
| wavelet-LLH_firstorder_Minimum | 5.19 | 14.37 | 0.989 | 0.902 | 0.997 | 5.6 | -23.1 |
| wavelet-LLH_firstorder_Range | 5.74 | 15.90 | 0.992 | 0.956 | 0.998 | 9.7 | -22.1 |
| wavelet-LLH_firstorder_RobustMeanAbsoluteDeviation | 8.36 | 23.18 | 0.981 | 0.932 | 0.994 | 16.8 | -29.6 |
| wavelet-LLH_firstorder_RootMeanSquared | 6.02 | 16.70 | 0.982 | 0.922 | 0.994 | 9.9 | -23.5 |
| wavelet-LLH_firstorder_Skewness | 719.23 | 1993.60 | 0.889 | 0.711 | 0.961 | 1729.7 | -2257.5 |
| wavelet-LLH_firstorder_TotalEnergy | 14.05 | 38.94 | 0.982 | 0.95 | 0.994 | 37.3 | -40.6 |
| wavelet-LLH_firstorder_Uniformity | 7.38 | 20.47 | 0.938 | 0.828 | 0.979 | 26.2 | -14.7 |
| wavelet-LLH_firstorder_Variance | 13.32 | 36.91 | 0.964 | 0.888 | 0.988 | 24.9 | -48.9 |
| wavelet-LLH_glcm_Autocorrelation | 16.34 | 45.30 | 0.987 | 0.949 | 0.996 | 36.4 | -54.2 |
| wavelet-LLH_glcm_ClusterProminence | 27.13 | 75.21 | 0.904 | 0.743 | 0.966 | 56.3 | -94.1 |
| wavelet-LLH_glcm_ClusterShade | 246.26 | 682.59 | 0.913 | 0.754 | 0.97 | 779.5 | -585.7 |
| wavelet-LLH_glcm_ClusterTendency | 14.08 | 39.03 | 0.964 | 0.891 | 0.988 | 28.8 | -49.3 |
| wavelet-LLH_glcm_Contrast | 15.21 | 42.15 | 0.929 | 0.768 | 0.977 | 24.1 | -60.2 |
| wavelet-LLH_glcm_Correlation | 2.87 | 7.95 | 0.731 | 0.277 | 0.907 | 10.6 | -5.3 |
| wavelet-LLH_glcm_DifferenceAverage | 10.27 | 28.47 | 0.96 | 0.838 | 0.988 | 17.9 | -39 |
| wavelet-LLH_glcm_DifferenceEntropy | 3.69 | 10.24 | 0.984 | 0.901 | 0.996 | 5.8 | -14.7 |
| wavelet-LLH_glcm_DifferenceVariance | 12.10 | 33.54 | 0.941 | 0.81 | 0.981 | 18.4 | -48.7 |
| wavelet-LLH_glcm_Id | 4.32 | 11.97 | 0.966 | 0.878 | 0.989 | 15.7 | -8.3 |
| wavelet-LLH_glcm_Idm | 5.83 | 16.17 | 0.969 | 0.889 | 0.99 | 20.9 | -11.4 |
| wavelet-LLH_glcm_Idmn | 0.30 | 0.82 | 0.775 | 0.437 | 0.919 | 1 | -0.6 |
| wavelet-LLH_glcm_Idn | 0.84 | 2.32 | 0.8 | 0.5 | 0.928 | 2.9 | -1.8 |
| wavelet-LLH_glcm_Imc1 | 6.80 | 18.85 | 0.706 | 0.335 | 0.89 | 18.4 | -19.3 |
| wavelet-LLH_glcm_Imc2 | 1.89 | 5.24 | 0.961 | 0.89 | 0.986 | 4.8 | -5.6 |
| wavelet-LLH_glcm_InverseVariance | 8.37 | 23.21 | 0.89 | 0.711 | 0.961 | 24.2 | -22.3 |
| wavelet-LLH_glcm_JointAverage | 8.61 | 23.87 | 0.989 | 0.955 | 0.997 | 19.4 | -28.3 |
| wavelet-LLH_glcm_JointEnergy | 16.56 | 45.90 | 0.808 | 0.532 | 0.931 | 56.6 | -35.2 |
| wavelet-LLH_glcm_JointEntropy | 5.29 | 14.66 | 0.987 | 0.959 | 0.996 | 11.5 | -17.8 |
| wavelet-LLH_glcm_MCC | 2.51 | 6.96 | 0.738 | 0.261 | 0.911 | 9.6 | -4.4 |
| wavelet-LLH_glcm_MaximumProbability | 18.07 | 50.09 | 0.845 | 0.609 | 0.945 | 59.3 | -40.9 |
| wavelet-LLH_glcm_SumAverage | 8.61 | 23.87 | 0.989 | 0.955 | 0.997 | 19.4 | -28.3 |
| wavelet-LLH_glcm_SumEntropy | 4.38 | 12.14 | 0.986 | 0.956 | 0.996 | 9.6 | -14.7 |
| wavelet-LLH_glcm_SumSquares | 13.95 | 38.68 | 0.96 | 0.877 | 0.987 | 27.3 | -50 |
| wavelet-LLH_gldm_DependenceEntropy | 2.55 | 7.06 | 0.949 | 0.859 | 0.982 | 6.9 | -7.2 |
| wavelet-LLH_gldm_DependenceNonUniformity | 9.51 | 26.36 | 0.993 | 0.971 | 0.998 | 36.3 | -16.5 |
| wavelet-LLH_gldm_DependenceNonUniformityNormalized | 10.03 | 27.80 | 0.904 | 0.745 | 0.966 | 25.8 | -29.8 |
| wavelet-LLH_gldm_DependenceVariance | 28.49 | 78.97 | 0.763 | 0.441 | 0.913 | 87.7 | -70.3 |
| wavelet-LLH_gldm_GrayLevelNonUniformity | 16.06 | 44.53 | 0.994 | 0.979 | 0.998 | 61.9 | -27.2 |
| wavelet-LLH_gldm_GrayLevelVariance | 13.50 | 37.42 | 0.965 | 0.89 | 0.988 | 25.9 | -49 |
| wavelet-LLH_gldm_HighGrayLevelEmphasis | 15.54 | 43.08 | 0.986 | 0.945 | 0.996 | 33.4 | -52.7 |
| wavelet-LLH_gldm_LargeDependenceEmphasis | 19.88 | 55.12 | 0.831 | 0.574 | 0.94 | 68.2 | -42 |
| wavelet-LLH_gldm_LargeDependenceHighGrayLevelEmphasis | 28.09 | 77.87 | 0.912 | 0.766 | 0.969 | 82 | -73.7 |
| wavelet-LLH_gldm_LargeDependenceLowGrayLevelEmphasis | 32.07 | 88.89 | 0.939 | 0.833 | 0.979 | 94.1 | -83.7 |
| wavelet-LLH_gldm_LowGrayLevelEmphasis | 25.63 | 71.05 | 0.925 | 0.792 | 0.974 | 69.6 | -72.5 |
| wavelet-LLH_gldm_SmallDependenceEmphasis | 13.41 | 37.16 | 0.962 | 0.849 | 0.988 | 22.6 | -51.7 |
| wavelet-LLH_gldm_SmallDependenceHighGrayLevelEmphasis | 25.58 | 70.90 | 0.962 | 0.882 | 0.988 | 44.5 | -97.3 |
| wavelet-LLH_gldm_SmallDependenceLowGrayLevelEmphasis | 26.81 | 74.30 | 0.676 | 0.282 | 0.877 | 81.9 | -66.7 |
| wavelet-LLH_glrlm_GrayLevelNonUniformity | 12.33 | 34.18 | 0.998 | 0.987 | 0.999 | 47.6 | -20.8 |
| wavelet-LLH_glrlm_GrayLevelNonUniformityNormalized | 5.26 | 14.57 | 0.978 | 0.932 | 0.993 | 19.1 | -10.1 |
| wavelet-LLH_glrlm_GrayLevelVariance | 11.34 | 31.43 | 0.97 | 0.904 | 0.99 | 20.9 | -42 |
| wavelet-LLH_glrlm_HighGrayLevelRunEmphasis | 15.47 | 42.89 | 0.986 | 0.946 | 0.996 | 33 | -52.8 |
| wavelet-LLH_glrlm_LongRunEmphasis | 10.29 | 28.52 | 0.812 | 0.532 | 0.932 | 36.2 | -20.8 |
| wavelet-LLH_glrlm_LongRunHighGrayLevelEmphasis | 19.30 | 53.49 | 0.99 | 0.967 | 0.997 | 51.6 | -55.3 |
| wavelet-LLH_glrlm_LongRunLowGrayLevelEmphasis | 26.58 | 73.67 | 0.953 | 0.87 | 0.984 | 77.8 | -69.6 |
| wavelet-LLH_glrlm_LowGrayLevelRunEmphasis | 24.39 | 67.61 | 0.942 | 0.837 | 0.98 | 67.9 | -67.4 |
| wavelet-LLH_glrlm_RunEntropy | 1.86 | 5.15 | 0.99 | 0.972 | 0.997 | 5.2 | -5.1 |
| wavelet-LLH_glrlm_RunLengthNonUniformity | 11.67 | 32.34 | 0.993 | 0.98 | 0.998 | 37.7 | -27 |
| wavelet-LLH_glrlm_RunLengthNonUniformityNormalized | 5.55 | 15.39 | 0.941 | 0.822 | 0.98 | 11.7 | -19.1 |
| wavelet-LLH_glrlm_RunPercentage | 4.28 | 11.86 | 0.912 | 0.74 | 0.97 | 9 | -14.7 |
| wavelet-LLH_glrlm_RunVariance | 20.14 | 55.82 | 0.709 | 0.342 | 0.89 | 71.8 | -39.8 |
| wavelet-LLH_glrlm_ShortRunEmphasis | 2.97 | 8.24 | 0.938 | 0.815 | 0.979 | 6.3 | -10.2 |
| wavelet-LLH_glrlm_ShortRunHighGrayLevelEmphasis | 15.33 | 42.48 | 0.985 | 0.942 | 0.995 | 30.3 | -54.6 |
| wavelet-LLH_glrlm_ShortRunLowGrayLevelEmphasis | 23.82 | 66.03 | 0.932 | 0.813 | 0.977 | 66.1 | -65.9 |
| wavelet-LLH_glszm_GrayLevelNonUniformity | 18.00 | 49.91 | 0.929 | 0.807 | 0.975 | 54.2 | -45.6 |
| wavelet-LLH_glszm_GrayLevelNonUniformityNormalized | 10.18 | 28.21 | 0.953 | 0.859 | 0.984 | 36.2 | -20.2 |
| wavelet-LLH_glszm_GrayLevelVariance | 12.24 | 33.92 | 0.986 | 0.952 | 0.996 | 28.1 | -39.8 |
| wavelet-LLH_glszm_HighGrayLevelZoneEmphasis | 15.26 | 42.30 | 0.989 | 0.962 | 0.996 | 31.4 | -53.2 |
| wavelet-LLH_glszm_LargeAreaEmphasis | 32.75 | 90.77 | 0.98 | 0.941 | 0.993 | 129 | -52.5 |
| wavelet-LLH_glszm_LargeAreaHighGrayLevelEmphasis | 35.50 | 98.41 | 0.959 | 0.885 | 0.986 | 124.6 | -72.2 |
| wavelet-LLH_glszm_LargeAreaLowGrayLevelEmphasis | 35.06 | 97.19 | 0.731 | 0.38 | 0.9 | 137.1 | -57.3 |
| wavelet-LLH_glszm_LowGrayLevelZoneEmphasis | 16.29 | 45.17 | 0.973 | 0.916 | 0.991 | 61.1 | -29.2 |
| wavelet-LLH_glszm_SizeZoneNonUniformity | 27.59 | 76.46 | 0.943 | 0.845 | 0.98 | 67 | -85.9 |
| wavelet-LLH_glszm_SizeZoneNonUniformityNormalized | 19.52 | 54.09 | 0.817 | 0.549 | 0.934 | 47.8 | -60.3 |
| wavelet-LLH_glszm_SmallAreaEmphasis | 43.83 | 121.49 | 0.856 | 0.633 | 0.949 | 126.8 | -116.2 |
| wavelet-LLH_glszm_SmallAreaHighGrayLevelEmphasis | 56.35 | 156.19 | 0.973 | 0.92 | 0.991 | 146.9 | -165.5 |
| wavelet-LLH_glszm_SmallAreaLowGrayLevelEmphasis | 63.96 | 177.29 | 0.174 | -0.308 | 0.606 | 215.4 | -139.2 |
| wavelet-LLH_glszm_ZoneEntropy | 3.50 | 9.70 | 0.991 | 0.973 | 0.997 | 9.5 | -9.9 |
| wavelet-LLH_glszm_ZonePercentage | 14.32 | 39.69 | 0.941 | 0.789 | 0.981 | 24.2 | -55.1 |
| wavelet-LLH_glszm_ZoneVariance | 35.74 | 99.06 | 0.984 | 0.952 | 0.995 | 142.5 | -55.6 |
| wavelet-LLH_ngtdm_Busyness | 18.72 | 51.90 | 0.926 | 0.799 | 0.974 | 67.6 | -36.2 |
| wavelet-LLH_ngtdm_Coarseness | 13.96 | 38.70 | 0.924 | 0.794 | 0.974 | 31.3 | -46.1 |
| wavelet-LLH_ngtdm_Complexity | 18.70 | 51.84 | 0.941 | 0.83 | 0.98 | 35.9 | -67.8 |
| wavelet-LLH_ngtdm_Contrast | 18.84 | 52.23 | 0.87 | 0.634 | 0.956 | 39.5 | -64.9 |
| wavelet-LLH_ngtdm_Strength | 16.56 | 45.89 | 0.937 | 0.79 | 0.98 | 30.8 | -61 |
| wavelet-LHL_firstorder_10Percentile | 13.42 | 37.19 | 0.952 | 0.866 | 0.983 | 31.7 | -42.6 |
| wavelet-LHL_firstorder_90Percentile | 24.06 | 66.70 | 0.958 | 0.884 | 0.986 | 67.9 | -65.5 |
| wavelet-LHL_firstorder_Energy | 30.63 | 84.90 | 0.993 | 0.981 | 0.998 | 88.7 | -81.1 |
| wavelet-LHL_firstorder_Entropy | 6.31 | 17.49 | 0.964 | 0.899 | 0.988 | 15.9 | -19.1 |
| wavelet-LHL_firstorder_InterquartileRange | 17.79 | 49.30 | 0.915 | 0.774 | 0.971 | 46.1 | -52.5 |
| wavelet-LHL_firstorder_Kurtosis | 9.19 | 25.47 | 0.843 | 0.605 | 0.944 | 27.9 | -23 |
| wavelet-LHL_firstorder_Maximum | 19.18 | 53.16 | 0.986 | 0.961 | 0.995 | 54 | -52.3 |
| wavelet-LHL_firstorder_MeanAbsoluteDeviation | 15.08 | 41.80 | 0.963 | 0.897 | 0.987 | 38.9 | -44.7 |
| wavelet-LHL_firstorder_Mean | 11.35 | 31.47 | 0.914 | 0.753 | 0.971 | 22.2 | -40.7 |
| wavelet-LHL_firstorder_Median | 56.95 | 157.85 | 0.796 | 0.504 | 0.926 | 160.9 | -154.8 |
| wavelet-LHL_firstorder_Minimum | 17.18 | 47.62 | 0.969 | 0.912 | 0.989 | 46.9 | -48.4 |
| wavelet-LHL_firstorder_Range | 16.19 | 44.87 | 0.983 | 0.953 | 0.994 | 44.4 | -45.4 |
| wavelet-LHL_firstorder_RobustMeanAbsoluteDeviation | 15.81 | 43.83 | 0.934 | 0.82 | 0.977 | 40.7 | -47 |
| wavelet-LHL_firstorder_RootMeanSquared | 12.53 | 34.74 | 0.973 | 0.925 | 0.991 | 31.1 | -38.4 |
| wavelet-LHL_firstorder_Skewness | 91.85 | 254.60 | 0.809 | 0.533 | 0.931 | 218.5 | -290.7 |
| wavelet-LHL_firstorder_TotalEnergy | 30.63 | 84.90 | 0.993 | 0.981 | 0.998 | 88.7 | -81.1 |
| wavelet-LHL_firstorder_Uniformity | 13.17 | 36.51 | 0.962 | 0.893 | 0.987 | 41.3 | -31.7 |
| wavelet-LHL_firstorder_Variance | 28.88 | 80.05 | 0.98 | 0.944 | 0.993 | 75.4 | -84.7 |
| wavelet-LHL_glcm_Autocorrelation | 34.93 | 96.82 | 0.955 | 0.876 | 0.985 | 92.4 | -101.2 |
| wavelet-LHL_glcm_ClusterProminence | 50.75 | 140.67 | 0.998 | 0.995 | 0.999 | 133.5 | -147.8 |
| wavelet-LHL_glcm_ClusterShade | 334.72 | 927.79 | 0.99 | 0.971 | 0.997 | 1020.7 | -834.9 |
| wavelet-LHL_glcm_ClusterTendency | 28.07 | 77.82 | 0.982 | 0.948 | 0.994 | 73 | -82.7 |
| wavelet-LHL_glcm_Contrast | 29.46 | 81.67 | 0.981 | 0.947 | 0.994 | 69.9 | -93.4 |
| wavelet-LHL_glcm_Correlation | 6.02 | 16.68 | 0.883 | 0.682 | 0.96 | 20.5 | -12.8 |
| wavelet-LHL_glcm_DifferenceAverage | 16.01 | 44.37 | 0.955 | 0.873 | 0.985 | 37.7 | -51 |
| wavelet-LHL_glcm_DifferenceEntropy | 8.40 | 23.29 | 0.955 | 0.874 | 0.984 | 19.8 | -26.8 |
| wavelet-LHL_glcm_DifferenceVariance | 29.15 | 80.79 | 0.993 | 0.98 | 0.998 | 72.5 | -89.1 |
| wavelet-LHL_glcm_Id | 6.64 | 18.40 | 0.944 | 0.843 | 0.981 | 22.6 | -14.2 |
| wavelet-LHL_glcm_Idm | 9.03 | 25.03 | 0.947 | 0.847 | 0.982 | 31.1 | -18.9 |
| wavelet-LHL_glcm_Idmn | 0.45 | 1.25 | 0.885 | 0.701 | 0.96 | 1.4 | -1.1 |
| wavelet-LHL_glcm_Idn | 0.78 | 2.15 | 0.92 | 0.786 | 0.972 | 2.5 | -1.8 |
| wavelet-LHL_glcm_Imc1 | 7.90 | 21.90 | 0.74 | 0.397 | 0.904 | 21.1 | -22.7 |
| wavelet-LHL_glcm_Imc2 | 2.67 | 7.39 | 0.922 | 0.79 | 0.973 | 6.9 | -7.8 |
| wavelet-LHL_glcm_InverseVariance | 8.53 | 23.63 | 0.827 | 0.542 | 0.939 | 30 | -17.3 |
| wavelet-LHL_glcm_JointAverage | 18.61 | 51.60 | 0.969 | 0.914 | 0.99 | 49.2 | -54 |
| wavelet-LHL_glcm_JointEnergy | 23.45 | 65.01 | 0.932 | 0.814 | 0.976 | 75.3 | -54.8 |
| wavelet-LHL_glcm_JointEntropy | 6.64 | 18.42 | 0.965 | 0.902 | 0.988 | 17 | -19.8 |
| wavelet-LHL_glcm_MCC | 3.43 | 9.52 | 0.877 | 0.681 | 0.956 | 9.3 | -9.7 |
| wavelet-LHL_glcm_MaximumProbability | 23.58 | 65.37 | 0.91 | 0.76 | 0.968 | 79.5 | -51.2 |
| wavelet-LHL_glcm_SumAverage | 18.61 | 51.60 | 0.969 | 0.914 | 0.99 | 49.2 | -54 |
| wavelet-LHL_glcm_SumEntropy | 5.27 | 14.61 | 0.969 | 0.914 | 0.989 | 13.7 | -15.5 |
| wavelet-LHL_glcm_SumSquares | 28.05 | 77.76 | 0.982 | 0.948 | 0.994 | 71.5 | -84.1 |
| wavelet-LHL_gldm_DependenceEntropy | 3.17 | 8.78 | 0.933 | 0.818 | 0.977 | 9.4 | -8.2 |
| wavelet-LHL_gldm_DependenceNonUniformity | 17.37 | 48.14 | 0.98 | 0.943 | 0.993 | 51.4 | -44.9 |
| wavelet-LHL_gldm_DependenceNonUniformityNormalized | 13.43 | 37.23 | 0.794 | 0.471 | 0.927 | 28.7 | -45.7 |
| wavelet-LHL_gldm_DependenceVariance | 27.35 | 75.81 | 0.831 | 0.578 | 0.939 | 96.2 | -55.5 |
| wavelet-LHL_gldm_GrayLevelNonUniformity | 12.21 | 33.85 | 0.997 | 0.985 | 0.999 | 50.4 | -17.3 |
| wavelet-LHL_gldm_GrayLevelVariance | 27.03 | 74.93 | 0.98 | 0.944 | 0.993 | 69.3 | -80.5 |
| wavelet-LHL_gldm_HighGrayLevelEmphasis | 34.29 | 95.04 | 0.959 | 0.886 | 0.986 | 90 | -100.1 |
| wavelet-LHL_gldm_LargeDependenceEmphasis | 20.63 | 57.18 | 0.926 | 0.801 | 0.974 | 73.2 | -41.1 |
| wavelet-LHL_gldm_LargeDependenceHighGrayLevelEmphasis | 29.61 | 82.08 | 0.866 | 0.656 | 0.953 | 94.3 | -69.8 |
| wavelet-LHL_gldm_LargeDependenceLowGrayLevelEmphasis | 54.84 | 152.00 | 0.401 | -0.109 | 0.747 | 173 | -131 |
| wavelet-LHL_gldm_LowGrayLevelEmphasis | 37.37 | 103.58 | 0.615 | 0.183 | 0.851 | 110.7 | -96.5 |
| wavelet-LHL_gldm_SmallDependenceEmphasis | 21.33 | 59.13 | 0.902 | 0.723 | 0.966 | 43.2 | -75 |
| wavelet-LHL_gldm_SmallDependenceHighGrayLevelEmphasis | 46.22 | 128.13 | 0.998 | 0.995 | 0.999 | 107 | -149.3 |
| wavelet-LHL_gldm_SmallDependenceLowGrayLevelEmphasis | 23.17 | 64.22 | 0.829 | 0.576 | 0.939 | 52.3 | -76.1 |
| wavelet-LHL_glrlm_GrayLevelNonUniformity | 10.37 | 28.74 | 0.998 | 0.992 | 0.999 | 42.9 | -14.6 |
| wavelet-LHL_glrlm_GrayLevelNonUniformityNormalized | 13.70 | 37.96 | 0.954 | 0.873 | 0.984 | 41.6 | -34.3 |
| wavelet-LHL_glrlm_GrayLevelVariance | 27.81 | 77.07 | 0.983 | 0.952 | 0.994 | 72.4 | -81.8 |
| wavelet-LHL_glrlm_HighGrayLevelRunEmphasis | 34.07 | 94.45 | 0.96 | 0.888 | 0.986 | 88.8 | -100.1 |
| wavelet-LHL_glrlm_LongRunEmphasis | 10.91 | 30.23 | 0.874 | 0.669 | 0.955 | 39 | -21.5 |
| wavelet-LHL_glrlm_LongRunHighGrayLevelEmphasis | 28.11 | 77.91 | 0.94 | 0.837 | 0.979 | 82.7 | -73.2 |
| wavelet-LHL_glrlm_LongRunLowGrayLevelEmphasis | 44.65 | 123.77 | 0.499 | 0.016 | 0.797 | 136.8 | -110.7 |
| wavelet-LHL_glrlm_LowGrayLevelRunEmphasis | 36.05 | 99.92 | 0.673 | 0.277 | 0.876 | 108.3 | -91.5 |
| wavelet-LHL_glrlm_RunEntropy | 3.64 | 10.08 | 0.963 | 0.896 | 0.987 | 10.1 | -10.1 |
| wavelet-LHL_glrlm_RunLengthNonUniformity | 15.75 | 43.67 | 0.986 | 0.961 | 0.995 | 52.8 | -34.6 |
| wavelet-LHL_glrlm_RunLengthNonUniformityNormalized | 5.15 | 14.28 | 0.944 | 0.841 | 0.981 | 12.4 | -16.2 |
| wavelet-LHL_glrlm_RunPercentage | 3.36 | 9.30 | 0.941 | 0.834 | 0.98 | 7.9 | -10.7 |
| wavelet-LHL_glrlm_RunVariance | 21.52 | 59.66 | 0.851 | 0.592 | 0.949 | 80.9 | -38.5 |
| wavelet-LHL_glrlm_ShortRunEmphasis | 3.06 | 8.49 | 0.947 | 0.854 | 0.982 | 7.7 | -9.3 |
| wavelet-LHL_glrlm_ShortRunHighGrayLevelEmphasis | 35.67 | 98.87 | 0.964 | 0.898 | 0.987 | 92 | -105.8 |
| wavelet-LHL_glrlm_ShortRunLowGrayLevelEmphasis | 32.64 | 90.47 | 0.769 | 0.451 | 0.915 | 99.4 | -81.5 |
| wavelet-LHL_glszm_GrayLevelNonUniformity | 18.46 | 51.16 | 0.919 | 0.783 | 0.972 | 55.2 | -47.1 |
| wavelet-LHL_glszm_GrayLevelNonUniformityNormalized | 21.62 | 59.94 | 0.857 | 0.635 | 0.949 | 68.2 | -51.7 |
| wavelet-LHL_glszm_GrayLevelVariance | 29.38 | 81.43 | 0.995 | 0.984 | 0.998 | 78.3 | -84.5 |
| wavelet-LHL_glszm_HighGrayLevelZoneEmphasis | 28.64 | 79.38 | 0.974 | 0.926 | 0.991 | 72.7 | -86.1 |
| wavelet-LHL_glszm_LargeAreaEmphasis | 38.99 | 108.09 | 0.994 | 0.983 | 0.998 | 142.6 | -73.5 |
| wavelet-LHL_glszm_LargeAreaHighGrayLevelEmphasis | 28.76 | 79.72 | 0.994 | 0.982 | 0.998 | 109.9 | -49.5 |
| wavelet-LHL_glszm_LargeAreaLowGrayLevelEmphasis | 64.10 | 177.67 | 0.573 | 0.119 | 0.831 | 213 | -142.3 |
| wavelet-LHL_glszm_LowGrayLevelZoneEmphasis | 27.75 | 76.91 | 0.86 | 0.641 | 0.95 | 88 | -65.8 |
| wavelet-LHL_glszm_SizeZoneNonUniformity | 39.58 | 109.70 | 0.974 | 0.927 | 0.991 | 89.6 | -129.8 |
| wavelet-LHL_glszm_SizeZoneNonUniformityNormalized | 20.05 | 55.57 | 0.843 | 0.558 | 0.947 | 39.6 | -71.5 |
| wavelet-LHL_glszm_SmallAreaEmphasis | 32.39 | 89.79 | 0.821 | 0.547 | 0.936 | 68.4 | -111.2 |
| wavelet-LHL_glszm_SmallAreaHighGrayLevelEmphasis | 46.15 | 127.91 | 0.992 | 0.976 | 0.997 | 94.8 | -161 |
| wavelet-LHL_glszm_SmallAreaLowGrayLevelEmphasis | 37.30 | 103.39 | 0.905 | 0.748 | 0.967 | 96.9 | -109.9 |
| wavelet-LHL_glszm_ZoneEntropy | 7.24 | 20.06 | 0.946 | 0.851 | 0.981 | 19.1 | -21 |
| wavelet-LHL_glszm_ZonePercentage | 22.87 | 63.38 | 0.905 | 0.729 | 0.967 | 47.5 | -79.3 |
| wavelet-LHL_glszm_ZoneVariance | 38.25 | 106.02 | 0.994 | 0.982 | 0.998 | 141.5 | -70.5 |
| wavelet-LHL_ngtdm_Busyness | 34.71 | 96.21 | 0.596 | 0.154 | 0.842 | 106.9 | -85.5 |
| wavelet-LHL_ngtdm_Coarseness | 15.51 | 42.98 | 0.903 | 0.743 | 0.966 | 36.3 | -49.6 |
| wavelet-LHL_ngtdm_Complexity | 40.76 | 112.99 | 0.997 | 0.991 | 0.999 | 99.4 | -126.5 |
| wavelet-LHL_ngtdm_Contrast | 19.21 | 53.26 | 0.869 | 0.663 | 0.954 | 46.3 | -60.2 |
| wavelet-LHL_ngtdm_Strength | 29.05 | 80.51 | 0.989 | 0.968 | 0.996 | 68.1 | -92.9 |
| wavelet-LHH_firstorder_10Percentile | 18.22 | 50.50 | 0.953 | 0.87 | 0.984 | 47.2 | -53.8 |
| wavelet-LHH_firstorder_90Percentile | 14.85 | 41.16 | 0.868 | 0.661 | 0.953 | 35.2 | -47.1 |
| wavelet-LHH_firstorder_Energy | 31.31 | 86.78 | 0.972 | 0.922 | 0.991 | 82.3 | -91.3 |
| wavelet-LHH_firstorder_Entropy | 3.97 | 10.99 | 0.933 | 0.819 | 0.977 | 10.8 | -11.2 |
| wavelet-LHH_firstorder_InterquartileRange | 12.51 | 34.67 | 0.915 | 0.769 | 0.971 | 26.2 | -43.2 |
| wavelet-LHH_firstorder_Kurtosis | 14.69 | 40.73 | 0.773 | 0.46 | 0.917 | 37.4 | -44 |
| wavelet-LHH_firstorder_Maximum | 18.39 | 50.98 | 0.933 | 0.818 | 0.977 | 44.2 | -57.7 |
| wavelet-LHH_firstorder_MeanAbsoluteDeviation | 12.88 | 35.70 | 0.946 | 0.85 | 0.981 | 28.6 | -42.8 |
| wavelet-LHH_firstorder_Mean | 215.55 | 597.47 | 0.869 | 0.663 | 0.953 | 673.3 | -521.7 |
| wavelet-LHH_firstorder_Median | 127.85 | 354.38 | 0.805 | 0.523 | 0.929 | 299 | -409.7 |
| wavelet-LHH_firstorder_Minimum | 20.18 | 55.94 | 0.945 | 0.85 | 0.981 | 47.9 | -63.9 |
| wavelet-LHH_firstorder_Range | 17.82 | 49.38 | 0.94 | 0.836 | 0.979 | 42.2 | -56.5 |
| wavelet-LHH_firstorder_RobustMeanAbsoluteDeviation | 12.38 | 34.32 | 0.932 | 0.815 | 0.976 | 27.1 | -41.6 |
| wavelet-LHH_firstorder_RootMeanSquared | 13.84 | 38.36 | 0.952 | 0.867 | 0.983 | 30.4 | -46.3 |
| wavelet-LHH_firstorder_Skewness | 19.99 | 55.41 | 0.785 | 0.484 | 0.922 | 49.9 | -61 |
| wavelet-LHH_firstorder_TotalEnergy | 31.31 | 86.78 | 0.972 | 0.922 | 0.991 | 82.3 | -91.3 |
| wavelet-LHH_firstorder_Uniformity | 2.36 | 6.54 | 0.931 | 0.813 | 0.976 | 6.3 | -6.7 |
| wavelet-LHH_firstorder_Variance | 27.10 | 75.12 | 0.947 | 0.855 | 0.982 | 61.1 | -89.2 |
| wavelet-LHH_glcm_Autocorrelation | 26.93 | 74.65 | 0.589 | 0.144 | 0.839 | 76.2 | -73.1 |
| wavelet-LHH_glcm_ClusterProminence | 23.41 | 64.88 | 0.972 | 0.92 | 0.99 | 60.2 | -69.6 |
| wavelet-LHH_glcm_ClusterShade | 90.78 | 251.64 | 0.951 | 0.863 | 0.983 | 184.5 | -318.8 |
| wavelet-LHH_glcm_ClusterTendency | 8.24 | 22.85 | 0.931 | 0.813 | 0.976 | 24.5 | -21.2 |
| wavelet-LHH_glcm_Contrast | 7.06 | 19.58 | 0.945 | 0.848 | 0.981 | 16.8 | -22.4 |
| wavelet-LHH_glcm_Correlation | 4.53 | 12.56 | 0.867 | 0.597 | 0.956 | 16.5 | -8.6 |
| wavelet-LHH_glcm_DifferenceAverage | 6.33 | 17.56 | 0.911 | 0.757 | 0.969 | 14.4 | -20.7 |
| wavelet-LHH_glcm_DifferenceEntropy | 3.46 | 9.60 | 0.942 | 0.841 | 0.98 | 8.8 | -10.3 |
| wavelet-LHH_glcm_DifferenceVariance | 4.77 | 13.23 | 0.964 | 0.9 | 0.988 | 12.3 | -14.2 |
| wavelet-LHH_glcm_Id | 1.06 | 2.93 | 0.887 | 0.696 | 0.961 | 3.6 | -2.3 |
| wavelet-LHH_glcm_Idm | 1.09 | 3.01 | 0.896 | 0.719 | 0.964 | 3.6 | -2.4 |
| wavelet-LHH_glcm_Idmn | 0.77 | 2.13 | 0.804 | 0.521 | 0.929 | 2 | -2.3 |
| wavelet-LHH_glcm_Idn | 0.80 | 2.22 | 0.718 | 0.357 | 0.895 | 2.3 | -2.1 |
| wavelet-LHH_glcm_Imc1 | 7.70 | 21.33 | 0.804 | 0.51 | 0.93 | 26.4 | -16.3 |
| wavelet-LHH_glcm_Imc2 | 4.24 | 11.75 | 0.894 | 0.704 | 0.963 | 14.5 | -9 |
| wavelet-LHH_glcm_InverseVariance | 5.97 | 16.54 | 0.825 | 0.553 | 0.938 | 13.2 | -19.9 |
| wavelet-LHH_glcm_JointAverage | 14.65 | 40.62 | 0.608 | 0.172 | 0.847 | 41.4 | -39.9 |
| wavelet-LHH_glcm_JointEnergy | 5.37 | 14.88 | 0.936 | 0.826 | 0.978 | 14.4 | -15.4 |
| wavelet-LHH_glcm_JointEntropy | 4.80 | 13.31 | 0.936 | 0.826 | 0.978 | 13.3 | -13.3 |
| wavelet-LHH_glcm_MCC | 4.23 | 11.74 | 0.872 | 0.62 | 0.957 | 15.1 | -8.3 |
| wavelet-LHH_glcm_MaximumProbability | 5.90 | 16.36 | 0.904 | 0.73 | 0.967 | 13 | -19.8 |
| wavelet-LHH_glcm_SumAverage | 14.65 | 40.62 | 0.608 | 0.172 | 0.847 | 41.4 | -39.9 |
| wavelet-LHH_glcm_SumEntropy | 4.31 | 11.96 | 0.934 | 0.82 | 0.977 | 12.3 | -11.6 |
| wavelet-LHH_glcm_SumSquares | 7.61 | 21.10 | 0.938 | 0.83 | 0.978 | 21.5 | -20.7 |
| wavelet-LHH_gldm_DependenceEntropy | 1.47 | 4.06 | 0.864 | 0.643 | 0.952 | 5 | -3.2 |
| wavelet-LHH_gldm_DependenceNonUniformity | 10.20 | 28.27 | 0.995 | 0.986 | 0.998 | 37.7 | -18.8 |
| wavelet-LHH_gldm_DependenceNonUniformityNormalized | 5.25 | 14.56 | 0.567 | 0.122 | 0.827 | 12.1 | -17 |
| wavelet-LHH_gldm_DependenceVariance | 10.97 | 30.41 | 0.426 | -0.078 | 0.76 | 34.7 | -26.1 |
| wavelet-LHH_gldm_GrayLevelNonUniformity | 10.67 | 29.58 | 0.997 | 0.991 | 0.999 | 41.2 | -17.9 |
| wavelet-LHH_gldm_GrayLevelVariance | 6.25 | 17.32 | 0.94 | 0.837 | 0.979 | 16.8 | -17.8 |
| wavelet-LHH_gldm_HighGrayLevelEmphasis | 26.24 | 72.73 | 0.599 | 0.158 | 0.843 | 75 | -70.4 |
| wavelet-LHH_gldm_LargeDependenceEmphasis | 5.10 | 14.13 | 0.856 | 0.367 | 0.958 | 20.5 | -7.8 |
| wavelet-LHH_gldm_LargeDependenceHighGrayLevelEmphasis | 27.58 | 76.46 | 0.417 | -0.089 | 0.756 | 84.5 | -68.4 |
| wavelet-LHH_gldm_LargeDependenceLowGrayLevelEmphasis | 34.34 | 95.20 | 0.628 | 0.203 | 0.856 | 99.9 | -90.5 |
| wavelet-LHH_gldm_LowGrayLevelEmphasis | 33.06 | 91.64 | 0.575 | 0.123 | 0.833 | 88 | -95.3 |
| wavelet-LHH_gldm_SmallDependenceEmphasis | 16.08 | 44.57 | 0.67 | 0.234 | 0.878 | 32.9 | -56.3 |
| wavelet-LHH_gldm_SmallDependenceHighGrayLevelEmphasis | 34.93 | 96.83 | 0.603 | 0.164 | 0.845 | 94 | -99.7 |
| wavelet-LHH_gldm_SmallDependenceLowGrayLevelEmphasis | 31.43 | 87.13 | 0.578 | 0.139 | 0.833 | 67.7 | -106.6 |
| wavelet-LHH_glrlm_GrayLevelNonUniformity | 9.71 | 26.91 | 0.996 | 0.988 | 0.999 | 35.6 | -18.2 |
| wavelet-LHH_glrlm_GrayLevelNonUniformityNormalized | 2.90 | 8.05 | 0.91 | 0.761 | 0.969 | 8.6 | -7.5 |
| wavelet-LHH_glrlm_GrayLevelVariance | 8.08 | 22.41 | 0.951 | 0.865 | 0.983 | 21.4 | -23.4 |
| wavelet-LHH_glrlm_HighGrayLevelRunEmphasis | 25.97 | 71.99 | 0.63 | 0.207 | 0.857 | 74.7 | -69.2 |
| wavelet-LHH_glrlm_LongRunEmphasis | 5.84 | 16.17 | 0.925 | 0.493 | 0.98 | 24.3 | -8 |
| wavelet-LHH_glrlm_LongRunHighGrayLevelEmphasis | 26.35 | 73.03 | 0.441 | -0.06 | 0.768 | 83.3 | -62.8 |
| wavelet-LHH_glrlm_LongRunLowGrayLevelEmphasis | 34.22 | 94.85 | 0.723 | 0.365 | 0.897 | 99.6 | -90.1 |
| wavelet-LHH_glrlm_LowGrayLevelRunEmphasis | 32.73 | 90.72 | 0.621 | 0.193 | 0.853 | 86.2 | -95.2 |
| wavelet-LHH_glrlm_RunEntropy | 2.35 | 6.52 | 0.929 | 0.697 | 0.979 | 9.1 | -4 |
| wavelet-LHH_glrlm_RunLengthNonUniformity | 11.67 | 32.35 | 0.99 | 0.97 | 0.996 | 36.1 | -28.6 |
| wavelet-LHH_glrlm_RunLengthNonUniformityNormalized | 2.88 | 7.98 | 0.893 | 0.302 | 0.973 | 3.5 | -12.4 |
| wavelet-LHH_glrlm_RunPercentage | 2.61 | 7.24 | 0.868 | 0.322 | 0.964 | 3.6 | -10.9 |
| wavelet-LHH_glrlm_RunVariance | 9.12 | 25.27 | 0.934 | 0.719 | 0.98 | 34.5 | -16 |
| wavelet-LHH_glrlm_ShortRunEmphasis | 3.51 | 9.72 | 0.844 | 0.454 | 0.951 | 6.1 | -13.4 |
| wavelet-LHH_glrlm_ShortRunHighGrayLevelEmphasis | 26.73 | 74.10 | 0.718 | 0.356 | 0.895 | 74.7 | -73.5 |
| wavelet-LHH_glrlm_ShortRunLowGrayLevelEmphasis | 31.79 | 88.11 | 0.675 | 0.28 | 0.876 | 79.1 | -97.1 |
| wavelet-LHH_glszm_GrayLevelNonUniformity | 39.92 | 110.67 | 0.349 | -0.168 | 0.719 | 113 | -108.3 |
| wavelet-LHH_glszm_GrayLevelNonUniformityNormalized | 18.70 | 51.84 | 0.682 | 0.292 | 0.879 | 50.9 | -52.8 |
| wavelet-LHH_glszm_GrayLevelVariance | 35.54 | 98.51 | 0.717 | 0.354 | 0.894 | 98.4 | -98.7 |
| wavelet-LHH_glszm_HighGrayLevelZoneEmphasis | 35.29 | 97.82 | 0.521 | 0.046 | 0.807 | 102.3 | -93.4 |
| wavelet-LHH_glszm_LargeAreaEmphasis | 43.51 | 120.60 | 0.743 | 0.402 | 0.905 | 141.1 | -100.1 |
| wavelet-LHH_glszm_LargeAreaHighGrayLevelEmphasis | 47.28 | 131.05 | 0.753 | 0.421 | 0.909 | 153.2 | -108.9 |
| wavelet-LHH_glszm_LargeAreaLowGrayLevelEmphasis | 57.49 | 159.36 | 0.728 | 0.375 | 0.899 | 176.3 | -142.4 |
| wavelet-LHH_glszm_LowGrayLevelZoneEmphasis | 28.27 | 78.36 | 0.343 | -0.175 | 0.716 | 74.7 | -82 |
| wavelet-LHH_glszm_SizeZoneNonUniformity | 15.58 | 43.19 | 0.82 | 0.535 | 0.936 | 53.5 | -32.8 |
| wavelet-LHH_glszm_SizeZoneNonUniformityNormalized | 28.88 | 80.05 | 0.321 | -0.199 | 0.704 | 87.4 | -72.7 |
| wavelet-LHH_glszm_SmallAreaEmphasis | 101.35 | 280.92 | 0.393 | -0.118 | 0.743 | 272.4 | -289.4 |
| wavelet-LHH_glszm_SmallAreaHighGrayLevelEmphasis | 106.12 | 294.14 | 0.17 | -0.342 | 0.611 | 290.9 | -297.4 |
| wavelet-LHH_glszm_SmallAreaLowGrayLevelEmphasis | 106.70 | 295.77 | 0.131 | -0.386 | 0.589 | 277.4 | -314.2 |
| wavelet-LHH_glszm_ZoneEntropy | 27.13 | 75.19 | 0.494 | 0.009 | 0.794 | 72.5 | -77.9 |
| wavelet-LHH_glszm_ZonePercentage | 35.82 | 99.28 | 0.642 | 0.232 | 0.862 | 90.6 | -107.9 |
| wavelet-LHH_glszm_ZoneVariance | 60.07 | 166.51 | 0.854 | 0.629 | 0.948 | 182.1 | -150.9 |
| wavelet-LHH_ngtdm_Busyness | 46.48 | 128.84 | 0.984 | 0.953 | 0.994 | 154.3 | -103.3 |
| wavelet-LHH_ngtdm_Coarseness | 10.91 | 30.23 | 0.954 | 0.82 | 0.986 | 20.8 | -39.7 |
| wavelet-LHH_ngtdm_Complexity | 39.99 | 110.86 | 0.807 | 0.527 | 0.93 | 99.9 | -121.8 |
| wavelet-LHH_ngtdm_Contrast | 34.60 | 95.90 | 0.769 | 0.451 | 0.915 | 99.1 | -92.7 |
| wavelet-LHH_ngtdm_Strength | 36.20 | 100.35 | 0.651 | 0.241 | 0.866 | 85.3 | -115.4 |
| wavelet-HLL_firstorder_10Percentile | 13.00 | 36.05 | 0.972 | 0.922 | 0.991 | 31.8 | -40.3 |
| wavelet-HLL_firstorder_90Percentile | 23.65 | 65.55 | 0.971 | 0.918 | 0.99 | 72.9 | -58.2 |
| wavelet-HLL_firstorder_Energy | 28.11 | 77.92 | 0.971 | 0.917 | 0.99 | 82.9 | -73 |
| wavelet-HLL_firstorder_Entropy | 7.08 | 19.63 | 0.982 | 0.948 | 0.994 | 18.6 | -20.7 |
| wavelet-HLL_firstorder_InterquartileRange | 9.59 | 26.59 | 0.963 | 0.891 | 0.988 | 21.4 | -31.8 |
| wavelet-HLL_firstorder_Kurtosis | 13.74 | 38.10 | 0.777 | 0.468 | 0.919 | 36.7 | -39.5 |
| wavelet-HLL_firstorder_Maximum | 17.48 | 48.45 | 0.966 | 0.905 | 0.988 | 54.1 | -42.8 |
| wavelet-HLL_firstorder_MeanAbsoluteDeviation | 11.79 | 32.67 | 0.973 | 0.924 | 0.991 | 30 | -35.3 |
| wavelet-HLL_firstorder_Mean | 11.80 | 32.70 | 0.894 | 0.718 | 0.963 | 25.9 | -39.5 |
| wavelet-HLL_firstorder_Median | 21.28 | 59.00 | 0.843 | 0.604 | 0.944 | 61.6 | -56.3 |
| wavelet-HLL_firstorder_Minimum | 18.75 | 51.98 | 0.959 | 0.887 | 0.986 | 50.3 | -53.6 |
| wavelet-HLL_firstorder_Range | 17.00 | 47.12 | 0.975 | 0.929 | 0.991 | 47.5 | -46.7 |
| wavelet-HLL_firstorder_RobustMeanAbsoluteDeviation | 10.22 | 28.33 | 0.959 | 0.886 | 0.986 | 25.5 | -31.2 |
| wavelet-HLL_firstorder_RootMeanSquared | 11.95 | 33.11 | 0.977 | 0.936 | 0.992 | 29.8 | -36.4 |
| wavelet-HLL_firstorder_Skewness | 83.03 | 230.15 | 0.788 | 0.489 | 0.923 | 196 | -264.3 |
| wavelet-HLL_firstorder_TotalEnergy | 28.11 | 77.92 | 0.971 | 0.917 | 0.99 | 82.9 | -73 |
| wavelet-HLL_firstorder_Uniformity | 10.91 | 30.24 | 0.937 | 0.829 | 0.978 | 32.6 | -27.8 |
| wavelet-HLL_firstorder_Variance | 25.46 | 70.58 | 0.949 | 0.86 | 0.983 | 65.8 | -75.4 |
| wavelet-HLL_glcm_Autocorrelation | 37.84 | 104.90 | 0.939 | 0.834 | 0.979 | 98.3 | -111.5 |
| wavelet-HLL_glcm_ClusterProminence | 50.28 | 139.36 | 0.857 | 0.635 | 0.949 | 135.5 | -143.2 |
| wavelet-HLL_glcm_ClusterShade | 129.84 | 359.89 | 0.909 | 0.757 | 0.968 | 389.6 | -330.1 |
| wavelet-HLL_glcm_ClusterTendency | 24.71 | 68.48 | 0.944 | 0.846 | 0.981 | 66.4 | -70.6 |
| wavelet-HLL_glcm_Contrast | 24.96 | 69.18 | 0.935 | 0.82 | 0.978 | 63.8 | -74.5 |
| wavelet-HLL_glcm_Correlation | 3.58 | 9.92 | 0.867 | 0.658 | 0.953 | 10.7 | -9.1 |
| wavelet-HLL_glcm_DifferenceAverage | 13.43 | 37.22 | 0.965 | 0.899 | 0.988 | 33.9 | -40.6 |
| wavelet-HLL_glcm_DifferenceEntropy | 7.16 | 19.86 | 0.978 | 0.937 | 0.992 | 18.6 | -21.1 |
| wavelet-HLL_glcm_DifferenceVariance | 25.00 | 69.31 | 0.958 | 0.881 | 0.986 | 64.4 | -74.2 |
| wavelet-HLL_glcm_Id | 5.17 | 14.32 | 0.977 | 0.936 | 0.992 | 16.2 | -12.5 |
| wavelet-HLL_glcm_Idm | 7.19 | 19.94 | 0.978 | 0.937 | 0.992 | 22.7 | -17.2 |
| wavelet-HLL_glcm_Idmn | 0.51 | 1.40 | 0.535 | 0.065 | 0.814 | 1.4 | -1.4 |
| wavelet-HLL_glcm_Idn | 0.99 | 2.75 | 0.737 | 0.39 | 0.902 | 2.9 | -2.6 |
| wavelet-HLL_glcm_Imc1 | 5.30 | 14.69 | 0.881 | 0.692 | 0.958 | 13 | -16.4 |
| wavelet-HLL_glcm_Imc2 | 2.72 | 7.55 | 0.943 | 0.843 | 0.98 | 6.7 | -8.4 |
| wavelet-HLL_glcm_InverseVariance | 7.64 | 21.19 | 0.947 | 0.854 | 0.982 | 23.4 | -18.9 |
| wavelet-HLL_glcm_JointAverage | 20.37 | 56.45 | 0.957 | 0.88 | 0.985 | 52.8 | -60.1 |
| wavelet-HLL_glcm_JointEnergy | 19.43 | 53.84 | 0.903 | 0.744 | 0.966 | 59.1 | -48.6 |
| wavelet-HLL_glcm_JointEntropy | 6.63 | 18.37 | 0.984 | 0.954 | 0.994 | 17.8 | -19 |
| wavelet-HLL_glcm_MCC | 2.95 | 8.18 | 0.818 | 0.526 | 0.936 | 6.1 | -10.3 |
| wavelet-HLL_glcm_MaximumProbability | 21.21 | 58.79 | 0.939 | 0.834 | 0.979 | 69.3 | -48.3 |
| wavelet-HLL_glcm_SumAverage | 20.37 | 56.45 | 0.957 | 0.88 | 0.985 | 52.8 | -60.1 |
| wavelet-HLL_glcm_SumEntropy | 5.62 | 15.58 | 0.981 | 0.945 | 0.993 | 14.8 | -16.3 |
| wavelet-HLL_glcm_SumSquares | 24.58 | 68.12 | 0.943 | 0.844 | 0.98 | 65.6 | -70.7 |
| wavelet-HLL_gldm_DependenceEntropy | 2.27 | 6.30 | 0.966 | 0.905 | 0.988 | 6.7 | -5.9 |
| wavelet-HLL_gldm_DependenceNonUniformity | 16.14 | 44.74 | 0.98 | 0.943 | 0.993 | 56.2 | -33.3 |
| wavelet-HLL_gldm_DependenceNonUniformityNormalized | 12.04 | 33.37 | 0.905 | 0.748 | 0.967 | 33.1 | -33.7 |
| wavelet-HLL_gldm_DependenceVariance | 25.82 | 71.56 | 0.919 | 0.782 | 0.972 | 79.1 | -64 |
| wavelet-HLL_gldm_GrayLevelNonUniformity | 14.88 | 41.24 | 0.989 | 0.968 | 0.996 | 55.4 | -27.1 |
| wavelet-HLL_gldm_GrayLevelVariance | 23.30 | 64.57 | 0.949 | 0.859 | 0.982 | 61.5 | -67.6 |
| wavelet-HLL_gldm_HighGrayLevelEmphasis | 36.72 | 101.79 | 0.94 | 0.836 | 0.979 | 95.1 | -108.5 |
| wavelet-HLL_gldm_LargeDependenceEmphasis | 16.91 | 46.88 | 0.92 | 0.786 | 0.972 | 54.6 | -39.2 |
| wavelet-HLL_gldm_LargeDependenceHighGrayLevelEmphasis | 36.05 | 99.94 | 0.883 | 0.696 | 0.959 | 99.1 | -100.7 |
| wavelet-HLL_gldm_LargeDependenceLowGrayLevelEmphasis | 53.20 | 147.46 | 0.349 | -0.164 | 0.719 | 155.1 | -139.9 |
| wavelet-HLL_gldm_LowGrayLevelEmphasis | 41.31 | 114.51 | 0.559 | 0.101 | 0.825 | 118 | -111.1 |
| wavelet-HLL_gldm_SmallDependenceEmphasis | 14.58 | 40.42 | 0.969 | 0.911 | 0.989 | 32.4 | -48.4 |
| wavelet-HLL_gldm_SmallDependenceHighGrayLevelEmphasis | 41.28 | 114.42 | 0.864 | 0.652 | 0.952 | 103 | -125.9 |
| wavelet-HLL_gldm_SmallDependenceLowGrayLevelEmphasis | 24.83 | 68.82 | 0.772 | 0.46 | 0.916 | 58.2 | -79.4 |
| wavelet-HLL_glrlm_GrayLevelNonUniformity | 12.19 | 33.78 | 0.994 | 0.984 | 0.998 | 46.6 | -21 |
| wavelet-HLL_glrlm_GrayLevelNonUniformityNormalized | 10.74 | 29.78 | 0.93 | 0.811 | 0.976 | 31.7 | -27.9 |
| wavelet-HLL_glrlm_GrayLevelVariance | 23.84 | 66.08 | 0.957 | 0.88 | 0.985 | 63.2 | -68.9 |
| wavelet-HLL_glrlm_HighGrayLevelRunEmphasis | 36.53 | 101.24 | 0.94 | 0.836 | 0.979 | 94.5 | -108 |
| wavelet-HLL_glrlm_LongRunEmphasis | 9.00 | 24.96 | 0.89 | 0.712 | 0.961 | 28.3 | -21.6 |
| wavelet-HLL_glrlm_LongRunHighGrayLevelEmphasis | 34.04 | 94.35 | 0.965 | 0.902 | 0.988 | 90.7 | -98 |
| wavelet-HLL_glrlm_LongRunLowGrayLevelEmphasis | 44.58 | 123.58 | 0.397 | -0.111 | 0.745 | 127.8 | -119.4 |
| wavelet-HLL_glrlm_LowGrayLevelRunEmphasis | 40.17 | 111.36 | 0.558 | 0.098 | 0.824 | 114.9 | -107.8 |
| wavelet-HLL_glrlm_RunEntropy | 2.64 | 7.30 | 0.983 | 0.95 | 0.994 | 7.8 | -6.8 |
| wavelet-HLL_glrlm_RunLengthNonUniformity | 13.37 | 37.07 | 0.992 | 0.976 | 0.997 | 46.7 | -27.4 |
| wavelet-HLL_glrlm_RunLengthNonUniformityNormalized | 5.50 | 15.25 | 0.968 | 0.909 | 0.989 | 14.1 | -16.4 |
| wavelet-HLL_glrlm_RunPercentage | 3.48 | 9.64 | 0.956 | 0.878 | 0.985 | 8.6 | -10.7 |
| wavelet-HLL_glrlm_RunVariance | 20.39 | 56.52 | 0.872 | 0.67 | 0.955 | 68.9 | -44.1 |
| wavelet-HLL_glrlm_ShortRunEmphasis | 3.12 | 8.65 | 0.956 | 0.877 | 0.985 | 7.9 | -9.4 |
| wavelet-HLL_glrlm_ShortRunHighGrayLevelEmphasis | 37.62 | 104.28 | 0.935 | 0.824 | 0.978 | 97.2 | -111.3 |
| wavelet-HLL_glrlm_ShortRunLowGrayLevelEmphasis | 37.86 | 104.95 | 0.649 | 0.238 | 0.866 | 107.5 | -102.4 |
| wavelet-HLL_glszm_GrayLevelNonUniformity | 20.63 | 57.19 | 0.936 | 0.826 | 0.978 | 57.8 | -56.6 |
| wavelet-HLL_glszm_GrayLevelNonUniformityNormalized | 19.05 | 52.81 | 0.824 | 0.563 | 0.937 | 52.4 | -53.2 |
| wavelet-HLL_glszm_GrayLevelVariance | 25.63 | 71.04 | 0.984 | 0.955 | 0.995 | 70.4 | -71.7 |
| wavelet-HLL_glszm_HighGrayLevelZoneEmphasis | 31.38 | 86.98 | 0.939 | 0.834 | 0.979 | 82.3 | -91.7 |
| wavelet-HLL_glszm_LargeAreaEmphasis | 40.60 | 112.52 | 0.935 | 0.824 | 0.978 | 137.6 | -87.4 |
| wavelet-HLL_glszm_LargeAreaHighGrayLevelEmphasis | 34.15 | 94.66 | 0.934 | 0.821 | 0.977 | 113.4 | -75.9 |
| wavelet-HLL_glszm_LargeAreaLowGrayLevelEmphasis | 62.94 | 174.45 | 0.081 | -0.43 | 0.555 | 195.4 | -153.5 |
| wavelet-HLL_glszm_LowGrayLevelZoneEmphasis | 33.52 | 92.90 | 0.657 | 0.251 | 0.869 | 93.2 | -92.6 |
| wavelet-HLL_glszm_SizeZoneNonUniformity | 24.23 | 67.17 | 0.969 | 0.912 | 0.989 | 67.1 | -67.2 |
| wavelet-HLL_glszm_SizeZoneNonUniformityNormalized | 10.55 | 29.25 | 0.968 | 0.911 | 0.989 | 28.3 | -30.2 |
| wavelet-HLL_glszm_SmallAreaEmphasis | 24.64 | 68.29 | 0.909 | 0.759 | 0.968 | 69.6 | -67 |
| wavelet-HLL_glszm_SmallAreaHighGrayLevelEmphasis | 34.42 | 95.40 | 0.932 | 0.815 | 0.976 | 96.4 | -94.4 |
| wavelet-HLL_glszm_SmallAreaLowGrayLevelEmphasis | 45.99 | 127.47 | 0.692 | 0.309 | 0.884 | 114.2 | -140.8 |
| wavelet-HLL_glszm_ZoneEntropy | 7.50 | 20.80 | 0.961 | 0.89 | 0.986 | 22 | -19.6 |
| wavelet-HLL_glszm_ZonePercentage | 18.66 | 51.72 | 0.961 | 0.888 | 0.987 | 41.4 | -62.1 |
| wavelet-HLL_glszm_ZoneVariance | 44.31 | 122.83 | 0.953 | 0.87 | 0.984 | 149 | -96.7 |
| wavelet-HLL_ngtdm_Busyness | 40.37 | 111.89 | 0.207 | -0.316 | 0.637 | 119.2 | -104.6 |
| wavelet-HLL_ngtdm_Coarseness | 19.44 | 53.89 | 0.848 | 0.616 | 0.946 | 49.1 | -58.7 |
| wavelet-HLL_ngtdm_Complexity | 43.53 | 120.67 | 0.87 | 0.667 | 0.954 | 111.8 | -129.5 |
| wavelet-HLL_ngtdm_Contrast | 26.21 | 72.64 | 0.819 | 0.535 | 0.936 | 67.9 | -77.4 |
| wavelet-HLL_ngtdm_Strength | 38.50 | 106.71 | 0.954 | 0.874 | 0.984 | 96.1 | -117.3 |
| wavelet-HLH_firstorder_10Percentile | 15.11 | 41.89 | 0.961 | 0.89 | 0.987 | 40.2 | -43.6 |
| wavelet-HLH_firstorder_90Percentile | 8.35 | 23.13 | 0.974 | 0.923 | 0.991 | 17 | -29.3 |
| wavelet-HLH_firstorder_Energy | 23.37 | 64.77 | 0.931 | 0.812 | 0.976 | 59.8 | -69.8 |
| wavelet-HLH_firstorder_Entropy | 3.90 | 10.82 | 0.943 | 0.816 | 0.981 | 7.7 | -13.9 |
| wavelet-HLH_firstorder_InterquartileRange | 10.49 | 29.09 | 0.949 | 0.845 | 0.983 | 21.4 | -36.8 |
| wavelet-HLH_firstorder_Kurtosis | 14.52 | 40.25 | 0.761 | 0.421 | 0.913 | 31.2 | -49.3 |
| wavelet-HLH_firstorder_Maximum | 14.54 | 40.31 | 0.922 | 0.74 | 0.975 | 28.8 | -51.9 |
| wavelet-HLH_firstorder_MeanAbsoluteDeviation | 9.59 | 26.59 | 0.959 | 0.86 | 0.987 | 18.6 | -34.5 |
| wavelet-HLH_firstorder_Mean | 54.15 | 150.10 | 0.862 | 0.604 | 0.953 | 140.9 | -159.3 |
| wavelet-HLH_firstorder_Median | 122.57 | 339.75 | 0.686 | 0.3 | 0.881 | 368.2 | -311.3 |
| wavelet-HLH_firstorder_Minimum | 15.17 | 42.04 | 0.99 | 0.968 | 0.997 | 35.4 | -48.7 |
| wavelet-HLH_firstorder_Range | 12.88 | 35.71 | 0.964 | 0.863 | 0.989 | 25.9 | -45.5 |
| wavelet-HLH_firstorder_RobustMeanAbsoluteDeviation | 10.33 | 28.63 | 0.95 | 0.84 | 0.984 | 20.9 | -36.4 |
| wavelet-HLH_firstorder_RootMeanSquared | 9.37 | 25.98 | 0.962 | 0.86 | 0.988 | 17.5 | -34.5 |
| wavelet-HLH_firstorder_Skewness | 39.15 | 108.53 | 0.65 | 0.238 | 0.866 | 95 | -122.1 |
| wavelet-HLH_firstorder_TotalEnergy | 23.37 | 64.77 | 0.931 | 0.812 | 0.976 | 59.8 | -69.8 |
| wavelet-HLH_firstorder_Uniformity | 2.83 | 7.85 | 0.938 | 0.804 | 0.98 | 10 | -5.7 |
| wavelet-HLH_firstorder_Variance | 19.24 | 53.32 | 0.91 | 0.739 | 0.97 | 37 | -69.7 |
| wavelet-HLH_glcm_Autocorrelation | 2.54 | 7.03 | 1 | 0.999 | 1 | 5.7 | -8.3 |
| wavelet-HLH_glcm_ClusterProminence | 24.69 | 68.44 | 0.75 | 0.416 | 0.907 | 46.9 | -90 |
| wavelet-HLH_glcm_ClusterShade | 66.34 | 183.89 | 0.758 | 0.413 | 0.912 | 148.7 | -219.1 |
| wavelet-HLH_glcm_ClusterTendency | 9.47 | 26.24 | 0.86 | 0.633 | 0.951 | 19.4 | -33 |
| wavelet-HLH_glcm_Contrast | 7.97 | 22.11 | 0.878 | 0.643 | 0.959 | 14.5 | -29.7 |
| wavelet-HLH_glcm_Correlation | 2.62 | 7.27 | 0.937 | 0.828 | 0.978 | 8 | -6.6 |
| wavelet-HLH_glcm_DifferenceAverage | 6.03 | 16.71 | 0.904 | 0.667 | 0.969 | 10.7 | -22.7 |
| wavelet-HLH_glcm_DifferenceEntropy | 3.89 | 10.78 | 0.932 | 0.796 | 0.977 | 8.1 | -13.5 |
| wavelet-HLH_glcm_DifferenceVariance | 6.43 | 17.81 | 0.907 | 0.734 | 0.968 | 13.3 | -22.4 |
| wavelet-HLH_glcm_Id | 1.28 | 3.53 | 0.914 | 0.672 | 0.973 | 4.8 | -2.3 |
| wavelet-HLH_glcm_Idm | 1.42 | 3.93 | 0.911 | 0.672 | 0.972 | 5.3 | -2.6 |
| wavelet-HLH_glcm_Idmn | 0.58 | 1.61 | 0.879 | 0.686 | 0.957 | 1.3 | -1.9 |
| wavelet-HLH_glcm_Idn | 0.53 | 1.48 | 0.822 | 0.559 | 0.936 | 1.5 | -1.5 |
| wavelet-HLH_glcm_Imc1 | 5.92 | 16.41 | 0.93 | 0.811 | 0.976 | 17.1 | -15.8 |
| wavelet-HLH_glcm_Imc2 | 2.73 | 7.57 | 0.949 | 0.86 | 0.982 | 6.8 | -8.3 |
| wavelet-HLH_glcm_InverseVariance | 4.65 | 12.88 | 0.917 | 0.685 | 0.974 | 8.6 | -17.2 |
| wavelet-HLH_glcm_JointAverage | 1.30 | 3.59 | 0.999 | 0.998 | 1 | 3 | -4.2 |
| wavelet-HLH_glcm_JointEnergy | 6.46 | 17.90 | 0.942 | 0.836 | 0.98 | 22.5 | -13.3 |
| wavelet-HLH_glcm_JointEntropy | 4.77 | 13.23 | 0.943 | 0.819 | 0.981 | 9.8 | -16.6 |
| wavelet-HLH_glcm_MCC | 3.11 | 8.63 | 0.929 | 0.807 | 0.975 | 8.8 | -8.5 |
| wavelet-HLH_glcm_MaximumProbability | 5.34 | 14.79 | 0.941 | 0.831 | 0.98 | 18.7 | -10.9 |
| wavelet-HLH_glcm_SumAverage | 1.30 | 3.59 | 0.999 | 0.998 | 1 | 3 | -4.2 |
| wavelet-HLH_glcm_SumEntropy | 4.27 | 11.83 | 0.946 | 0.835 | 0.982 | 9.1 | -14.6 |
| wavelet-HLH_glcm_SumSquares | 9.01 | 24.99 | 0.865 | 0.638 | 0.953 | 18 | -32 |
| wavelet-HLH_gldm_DependenceEntropy | 1.28 | 3.56 | 0.93 | 0.81 | 0.976 | 3.8 | -3.3 |
| wavelet-HLH_gldm_DependenceNonUniformity | 10.82 | 29.98 | 0.995 | 0.986 | 0.998 | 40.2 | -19.7 |
| wavelet-HLH_gldm_DependenceNonUniformityNormalized | 7.57 | 20.98 | 0.211 | -0.313 | 0.639 | 19.4 | -22.6 |
| wavelet-HLH_gldm_DependenceVariance | 6.53 | 18.10 | 0.671 | 0.132 | 0.887 | 26 | -10.2 |
| wavelet-HLH_gldm_GrayLevelNonUniformity | 11.98 | 33.22 | 0.996 | 0.989 | 0.999 | 47.2 | -19.3 |
| wavelet-HLH_gldm_GrayLevelVariance | 7.69 | 21.31 | 0.869 | 0.649 | 0.954 | 15 | -27.6 |
| wavelet-HLH_gldm_HighGrayLevelEmphasis | 2.89 | 8.01 | 1 | 0.999 | 1 | 6.9 | -9.1 |
| wavelet-HLH_gldm_LargeDependenceEmphasis | 5.32 | 14.74 | 0.831 | 0.088 | 0.957 | 23.6 | -5.9 |
| wavelet-HLH_gldm_LargeDependenceHighGrayLevelEmphasis | 6.90 | 19.13 | 0.946 | 0.83 | 0.982 | 27.8 | -10.5 |
| wavelet-HLH_gldm_LargeDependenceLowGrayLevelEmphasis | 5.23 | 14.49 | 0.982 | 0.897 | 0.995 | 23.2 | -5.8 |
| wavelet-HLH_gldm_LowGrayLevelEmphasis | 3.34 | 9.27 | 0.995 | 0.985 | 0.998 | 9 | -9.6 |
| wavelet-HLH_gldm_SmallDependenceEmphasis | 14.47 | 40.11 | 0.449 | -0.049 | 0.772 | 37.2 | -43 |
| wavelet-HLH_gldm_SmallDependenceHighGrayLevelEmphasis | 14.74 | 40.87 | 0.951 | 0.861 | 0.983 | 33.6 | -48.1 |
| wavelet-HLH_gldm_SmallDependenceLowGrayLevelEmphasis | 17.50 | 48.50 | 0.832 | 0.581 | 0.94 | 49.4 | -47.6 |
| wavelet-HLH_glrlm_GrayLevelNonUniformity | 9.84 | 27.27 | 0.996 | 0.989 | 0.999 | 36.6 | -17.9 |
| wavelet-HLH_glrlm_GrayLevelNonUniformityNormalized | 3.26 | 9.03 | 0.945 | 0.837 | 0.982 | 11.1 | -6.9 |
| wavelet-HLH_glrlm_GrayLevelVariance | 8.69 | 24.08 | 0.891 | 0.699 | 0.962 | 17.2 | -30.9 |
| wavelet-HLH_glrlm_HighGrayLevelRunEmphasis | 4.32 | 11.96 | 0.999 | 0.997 | 1 | 10.1 | -13.8 |
| wavelet-HLH_glrlm_LongRunEmphasis | 6.86 | 19.03 | 0.931 | 0.645 | 0.98 | 27.8 | -10.3 |
| wavelet-HLH_glrlm_LongRunHighGrayLevelEmphasis | 7.33 | 20.33 | 0.936 | 0.814 | 0.978 | 28.8 | -11.8 |
| wavelet-HLH_glrlm_LongRunLowGrayLevelEmphasis | 7.00 | 19.39 | 0.982 | 0.941 | 0.994 | 27.3 | -11.5 |
| wavelet-HLH_glrlm_LowGrayLevelRunEmphasis | 4.23 | 11.73 | 0.991 | 0.975 | 0.997 | 12 | -11.5 |
| wavelet-HLH_glrlm_RunEntropy | 2.45 | 6.79 | 0.939 | 0.81 | 0.98 | 8.6 | -5 |
| wavelet-HLH_glrlm_RunLengthNonUniformity | 9.81 | 27.19 | 0.988 | 0.965 | 0.996 | 28.1 | -26.3 |
| wavelet-HLH_glrlm_RunLengthNonUniformityNormalized | 4.19 | 11.60 | 0.772 | 0.142 | 0.933 | 5.6 | -17.6 |
| wavelet-HLH_glrlm_RunPercentage | 2.68 | 7.42 | 0.826 | 0.098 | 0.955 | 2.9 | -12 |
| wavelet-HLH_glrlm_RunVariance | 9.85 | 27.30 | 0.946 | 0.796 | 0.983 | 37.4 | -17.2 |
| wavelet-HLH_glrlm_ShortRunEmphasis | 2.79 | 7.74 | 0.854 | 0.254 | 0.96 | 3.7 | -11.8 |
| wavelet-HLH_glrlm_ShortRunHighGrayLevelEmphasis | 8.32 | 23.05 | 0.988 | 0.96 | 0.996 | 17 | -29.2 |
| wavelet-HLH_glrlm_ShortRunLowGrayLevelEmphasis | 7.34 | 20.35 | 0.977 | 0.935 | 0.992 | 17.3 | -23.4 |
| wavelet-HLH_glszm_GrayLevelNonUniformity | 21.32 | 59.11 | 0.783 | 0.448 | 0.923 | 75.4 | -42.8 |
| wavelet-HLH_glszm_GrayLevelNonUniformityNormalized | 9.83 | 27.25 | 0.919 | 0.707 | 0.974 | 36.3 | -18.2 |
| wavelet-HLH_glszm_GrayLevelVariance | 22.08 | 61.21 | 0.961 | 0.852 | 0.988 | 41 | -81.4 |
| wavelet-HLH_glszm_HighGrayLevelZoneEmphasis | 15.97 | 44.28 | 0.957 | 0.864 | 0.986 | 26.8 | -61.7 |
| wavelet-HLH_glszm_LargeAreaEmphasis | 27.43 | 76.04 | 0.987 | 0.962 | 0.995 | 96.3 | -55.8 |
| wavelet-HLH_glszm_LargeAreaHighGrayLevelEmphasis | 28.29 | 78.42 | 0.982 | 0.949 | 0.994 | 99.3 | -57.5 |
| wavelet-HLH_glszm_LargeAreaLowGrayLevelEmphasis | 27.01 | 74.88 | 0.984 | 0.953 | 0.994 | 92.4 | -57.4 |
| wavelet-HLH_glszm_LowGrayLevelZoneEmphasis | 12.23 | 33.90 | 0.89 | 0.596 | 0.966 | 45.3 | -22.5 |
| wavelet-HLH_glszm_SizeZoneNonUniformity | 21.41 | 59.35 | 0.47 | -0.022 | 0.783 | 68.2 | -50.5 |
| wavelet-HLH_glszm_SizeZoneNonUniformityNormalized | 12.66 | 35.09 | 0.894 | 0.722 | 0.963 | 36.5 | -33.6 |
| wavelet-HLH_glszm_SmallAreaEmphasis | 82.80 | 229.50 | 0.433 | -0.07 | 0.764 | 238.9 | -220.1 |
| wavelet-HLH_glszm_SmallAreaHighGrayLevelEmphasis | 79.80 | 221.19 | 0.965 | 0.902 | 0.988 | 214.7 | -227.7 |
| wavelet-HLH_glszm_SmallAreaLowGrayLevelEmphasis | 85.91 | 238.12 | 0.153 | -0.366 | 0.603 | 253 | -223.2 |
| wavelet-HLH_glszm_ZoneEntropy | 7.66 | 21.24 | 0.94 | 0.837 | 0.979 | 22.3 | -20.2 |
| wavelet-HLH_glszm_ZonePercentage | 16.86 | 46.75 | 0.803 | 0.521 | 0.929 | 42.4 | -51.1 |
| wavelet-HLH_glszm_ZoneVariance | 37.89 | 105.03 | 0.99 | 0.971 | 0.997 | 136 | -74.1 |
| wavelet-HLH_ngtdm_Busyness | 30.63 | 84.91 | 0.996 | 0.988 | 0.999 | 116.7 | -53.1 |
| wavelet-HLH_ngtdm_Coarseness | 11.29 | 31.30 | 0.938 | 0.626 | 0.983 | 18.7 | -43.9 |
| wavelet-HLH_ngtdm_Complexity | 29.07 | 80.57 | 0.943 | 0.815 | 0.981 | 57.8 | -103.4 |
| wavelet-HLH_ngtdm_Contrast | 25.85 | 71.65 | 0.799 | 0.512 | 0.927 | 82.3 | -61 |
| wavelet-HLH_ngtdm_Strength | 27.36 | 75.84 | 0.817 | 0.347 | 0.943 | 42.5 | -109.2 |
| wavelet-HHL_firstorder_10Percentile | 22.98 | 63.69 | 0.982 | 0.948 | 0.994 | 64.8 | -62.6 |
| wavelet-HHL_firstorder_90Percentile | 17.31 | 47.99 | 0.957 | 0.878 | 0.985 | 43 | -52.9 |
| wavelet-HHL_firstorder_Energy | 36.77 | 101.93 | 0.985 | 0.958 | 0.995 | 104.8 | -99.1 |
| wavelet-HHL_firstorder_Entropy | 4.06 | 11.27 | 0.975 | 0.93 | 0.991 | 10.6 | -11.9 |
| wavelet-HHL_firstorder_InterquartileRange | 15.59 | 43.22 | 0.949 | 0.861 | 0.983 | 39.2 | -47.2 |
| wavelet-HHL_firstorder_Kurtosis | 15.61 | 43.27 | 0.76 | 0.435 | 0.912 | 41.5 | -45 |
| wavelet-HHL_firstorder_Maximum | 26.31 | 72.93 | 0.973 | 0.925 | 0.991 | 72.6 | -73.2 |
| wavelet-HHL_firstorder_MeanAbsoluteDeviation | 17.38 | 48.17 | 0.969 | 0.912 | 0.989 | 44.3 | -52 |
| wavelet-HHL_firstorder_Mean | 240.70 | 667.17 | 0.867 | 0.652 | 0.953 | 564.8 | -769.6 |
| wavelet-HHL_firstorder_Median | 591.28 | 1638.93 | 0.887 | 0.705 | 0.96 | 1412 | -1865.9 |
| wavelet-HHL_firstorder_Minimum | 16.93 | 46.93 | 0.975 | 0.928 | 0.992 | 37.4 | -56.4 |
| wavelet-HHL_firstorder_Range | 22.43 | 62.16 | 0.975 | 0.93 | 0.992 | 58.3 | -66 |
| wavelet-HHL_firstorder_RobustMeanAbsoluteDeviation | 16.50 | 45.75 | 0.956 | 0.878 | 0.985 | 42.6 | -48.9 |
| wavelet-HHL_firstorder_RootMeanSquared | 17.44 | 48.35 | 0.977 | 0.934 | 0.992 | 44.1 | -52.6 |
| wavelet-HHL_firstorder_Skewness | 38.38 | 106.38 | 0.69 | 0.307 | 0.883 | 113.2 | -99.5 |
| wavelet-HHL_firstorder_TotalEnergy | 36.77 | 101.93 | 0.985 | 0.958 | 0.995 | 104.8 | -99.1 |
| wavelet-HHL_firstorder_Uniformity | 3.62 | 10.05 | 0.948 | 0.857 | 0.982 | 10.4 | -9.7 |
| wavelet-HHL_firstorder_Variance | 34.99 | 96.98 | 0.963 | 0.897 | 0.987 | 90 | -103.9 |
| wavelet-HHL_glcm_Autocorrelation | 30.99 | 85.90 | 0.751 | 0.418 | 0.908 | 87.8 | -84 |
| wavelet-HHL_glcm_ClusterProminence | 21.36 | 59.20 | 0.951 | 0.864 | 0.983 | 53.6 | -64.8 |
| wavelet-HHL_glcm_ClusterShade | 404.26 | 1120.56 | 0.981 | 0.946 | 0.993 | 1244.7 | -996.5 |
| wavelet-HHL_glcm_ClusterTendency | 9.78 | 27.10 | 0.962 | 0.893 | 0.987 | 27.1 | -27.1 |
| wavelet-HHL_glcm_Contrast | 8.96 | 24.84 | 0.959 | 0.886 | 0.986 | 21.1 | -28.6 |
| wavelet-HHL_glcm_Correlation | 9.79 | 27.14 | 0.744 | 0.408 | 0.905 | 31.8 | -22.5 |
| wavelet-HHL_glcm_DifferenceAverage | 8.20 | 22.74 | 0.95 | 0.86 | 0.983 | 19.5 | -26 |
| wavelet-HHL_glcm_DifferenceEntropy | 4.17 | 11.55 | 0.972 | 0.921 | 0.99 | 10 | -13.1 |
| wavelet-HHL_glcm_DifferenceVariance | 5.86 | 16.23 | 0.98 | 0.944 | 0.993 | 14.1 | -18.4 |
| wavelet-HHL_glcm_Id | 1.70 | 4.70 | 0.93 | 0.808 | 0.976 | 5.5 | -3.9 |
| wavelet-HHL_glcm_Idm | 1.85 | 5.12 | 0.938 | 0.829 | 0.979 | 6 | -4.2 |
| wavelet-HHL_glcm_Idmn | 0.84 | 2.32 | 0.816 | 0.547 | 0.934 | 2.2 | -2.5 |
| wavelet-HHL_glcm_Idn | 0.93 | 2.57 | 0.697 | 0.319 | 0.886 | 2.7 | -2.5 |
| wavelet-HHL_glcm_Imc1 | 15.46 | 42.86 | 0.715 | 0.354 | 0.893 | 49.8 | -35.9 |
| wavelet-HHL_glcm_Imc2 | 8.87 | 24.60 | 0.85 | 0.621 | 0.947 | 28.2 | -20.9 |
| wavelet-HHL_glcm_InverseVariance | 7.52 | 20.83 | 0.81 | 0.535 | 0.931 | 18.4 | -23.2 |
| wavelet-HHL_glcm_JointAverage | 16.93 | 46.94 | 0.718 | 0.357 | 0.895 | 48.1 | -45.8 |
| wavelet-HHL_glcm_JointEnergy | 9.29 | 25.76 | 0.882 | 0.692 | 0.958 | 27 | -24.5 |
| wavelet-HHL_glcm_JointEntropy | 5.75 | 15.93 | 0.968 | 0.909 | 0.989 | 15.1 | -16.8 |
| wavelet-HHL_glcm_MCC | 9.05 | 25.07 | 0.821 | 0.558 | 0.935 | 29.2 | -20.9 |
| wavelet-HHL_glcm_MaximumProbability | 9.51 | 26.36 | 0.798 | 0.509 | 0.927 | 28.1 | -24.6 |
| wavelet-HHL_glcm_SumAverage | 16.93 | 46.94 | 0.718 | 0.357 | 0.895 | 48.1 | -45.8 |
| wavelet-HHL_glcm_SumEntropy | 5.34 | 14.79 | 0.963 | 0.895 | 0.987 | 14.6 | -15 |
| wavelet-HHL_glcm_SumSquares | 8.68 | 24.07 | 0.962 | 0.893 | 0.987 | 22.9 | -25.3 |
| wavelet-HHL_gldm_DependenceEntropy | 1.96 | 5.44 | 0.868 | 0.66 | 0.953 | 6 | -4.8 |
| wavelet-HHL_gldm_DependenceNonUniformity | 14.92 | 41.36 | 0.997 | 0.99 | 0.999 | 52.2 | -30.5 |
| wavelet-HHL_gldm_DependenceNonUniformityNormalized | 8.36 | 23.18 | 0.326 | -0.194 | 0.706 | 22.3 | -24.1 |
| wavelet-HHL_gldm_DependenceVariance | 10.37 | 28.74 | 0.604 | 0.166 | 0.846 | 30.7 | -26.8 |
| wavelet-HHL_gldm_GrayLevelNonUniformity | 12.18 | 33.77 | 0.997 | 0.99 | 0.999 | 45.9 | -21.6 |
| wavelet-HHL_gldm_GrayLevelVariance | 6.70 | 18.58 | 0.96 | 0.887 | 0.986 | 17 | -20.1 |
| wavelet-HHL_gldm_HighGrayLevelEmphasis | 30.32 | 84.04 | 0.766 | 0.445 | 0.914 | 85.9 | -82.2 |
| wavelet-HHL_gldm_LargeDependenceEmphasis | 7.60 | 21.08 | 0.893 | 0.658 | 0.965 | 27.7 | -14.4 |
| wavelet-HHL_gldm_LargeDependenceHighGrayLevelEmphasis | 30.98 | 85.87 | 0.448 | -0.05 | 0.772 | 95.7 | -76.1 |
| wavelet-HHL_gldm_LargeDependenceLowGrayLevelEmphasis | 40.71 | 112.84 | 0.447 | -0.051 | 0.771 | 116.3 | -109.3 |
| wavelet-HHL_gldm_LowGrayLevelEmphasis | 38.20 | 105.87 | 0.418 | -0.088 | 0.756 | 103.5 | -108.3 |
| wavelet-HHL_gldm_SmallDependenceEmphasis | 17.65 | 48.91 | 0.685 | 0.27 | 0.883 | 34.5 | -63.3 |
| wavelet-HHL_gldm_SmallDependenceHighGrayLevelEmphasis | 39.44 | 109.33 | 0.813 | 0.54 | 0.932 | 93.4 | -125.3 |
| wavelet-HHL_gldm_SmallDependenceLowGrayLevelEmphasis | 32.96 | 91.37 | 0.711 | 0.349 | 0.891 | 81.3 | -101.5 |
| wavelet-HHL_glrlm_GrayLevelNonUniformity | 10.28 | 28.50 | 0.994 | 0.984 | 0.998 | 38.1 | -18.9 |
| wavelet-HHL_glrlm_GrayLevelNonUniformityNormalized | 2.86 | 7.93 | 0.98 | 0.942 | 0.993 | 9.1 | -6.8 |
| wavelet-HHL_glrlm_GrayLevelVariance | 7.00 | 19.41 | 0.976 | 0.931 | 0.992 | 17.2 | -21.7 |
| wavelet-HHL_glrlm_HighGrayLevelRunEmphasis | 29.99 | 83.12 | 0.784 | 0.481 | 0.921 | 84.2 | -82.1 |
| wavelet-HHL_glrlm_LongRunEmphasis | 6.83 | 18.92 | 0.975 | 0.808 | 0.993 | 27.4 | -10.5 |
| wavelet-HHL_glrlm_LongRunHighGrayLevelEmphasis | 30.56 | 84.72 | 0.536 | 0.066 | 0.814 | 95.7 | -73.7 |
| wavelet-HHL_glrlm_LongRunLowGrayLevelEmphasis | 40.16 | 111.33 | 0.734 | 0.385 | 0.901 | 116.7 | -106 |
| wavelet-HHL_glrlm_LowGrayLevelRunEmphasis | 37.91 | 105.08 | 0.512 | 0.033 | 0.803 | 103.4 | -106.8 |
| wavelet-HHL_glrlm_RunEntropy | 2.50 | 6.92 | 0.948 | 0.814 | 0.983 | 9.1 | -4.8 |
| wavelet-HHL_glrlm_RunLengthNonUniformity | 11.94 | 33.09 | 0.992 | 0.977 | 0.997 | 37.2 | -29 |
| wavelet-HHL_glrlm_RunLengthNonUniformityNormalized | 5.33 | 14.77 | 0.866 | 0.574 | 0.956 | 10.1 | -19.5 |
| wavelet-HHL_glrlm_RunPercentage | 3.53 | 9.78 | 0.892 | 0.613 | 0.966 | 6.4 | -13.2 |
| wavelet-HHL_glrlm_RunVariance | 10.26 | 28.44 | 0.987 | 0.941 | 0.996 | 37.8 | -19.1 |
| wavelet-HHL_glrlm_ShortRunEmphasis | 3.91 | 10.84 | 0.885 | 0.567 | 0.964 | 6.9 | -14.8 |
| wavelet-HHL_glrlm_ShortRunHighGrayLevelEmphasis | 30.97 | 85.84 | 0.817 | 0.549 | 0.934 | 82.1 | -89.6 |
| wavelet-HHL_glrlm_ShortRunLowGrayLevelEmphasis | 37.46 | 103.85 | 0.586 | 0.138 | 0.837 | 99.7 | -108 |
| wavelet-HHL_glszm_GrayLevelNonUniformity | 31.27 | 86.68 | 0.612 | 0.178 | 0.849 | 79.3 | -94 |
| wavelet-HHL_glszm_GrayLevelNonUniformityNormalized | 17.77 | 49.26 | 0.808 | 0.53 | 0.93 | 51.1 | -47.5 |
| wavelet-HHL_glszm_GrayLevelVariance | 33.71 | 93.45 | 0.976 | 0.932 | 0.992 | 89.8 | -97.1 |
| wavelet-HHL_glszm_HighGrayLevelZoneEmphasis | 26.66 | 73.90 | 0.937 | 0.828 | 0.978 | 63.3 | -84.5 |
| wavelet-HHL_glszm_LargeAreaEmphasis | 42.90 | 118.90 | 0.916 | 0.775 | 0.971 | 149.5 | -88.3 |
| wavelet-HHL_glszm_LargeAreaHighGrayLevelEmphasis | 49.62 | 137.54 | 0.91 | 0.761 | 0.969 | 170.8 | -104.2 |
| wavelet-HHL_glszm_LargeAreaLowGrayLevelEmphasis | 56.20 | 155.77 | 0.921 | 0.788 | 0.973 | 177.3 | -134.2 |
| wavelet-HHL_glszm_LowGrayLevelZoneEmphasis | 21.68 | 60.09 | 0.798 | 0.468 | 0.929 | 74.5 | -45.7 |
| wavelet-HHL_glszm_SizeZoneNonUniformity | 23.83 | 66.06 | 0.512 | 0.033 | 0.803 | 63.2 | -69 |
| wavelet-HHL_glszm_SizeZoneNonUniformityNormalized | 27.55 | 76.37 | 0.56 | 0.1 | 0.826 | 82 | -70.7 |
| wavelet-HHL_glszm_SmallAreaEmphasis | 93.88 | 260.22 | 0 | -0.497 | 0.497 | 218.7 | -301.7 |
| wavelet-HHL_glszm_SmallAreaHighGrayLevelEmphasis | 94.88 | 262.98 | 0.753 | 0.421 | 0.909 | 202.5 | -323.5 |
| wavelet-HHL_glszm_SmallAreaLowGrayLevelEmphasis | 90.98 | 252.19 | 0 | -0.497 | 0.497 | 246.5 | -257.8 |
| wavelet-HHL_glszm_ZoneEntropy | 24.67 | 68.37 | 0.717 | 0.355 | 0.894 | 65.2 | -71.5 |
| wavelet-HHL_glszm_ZonePercentage | 31.73 | 87.94 | 0.596 | 0.166 | 0.841 | 68.2 | -107.7 |
| wavelet-HHL_glszm_ZoneVariance | 52.63 | 145.88 | 0.986 | 0.961 | 0.995 | 159.2 | -132.6 |
| wavelet-HHL_ngtdm_Busyness | 42.58 | 118.01 | 0.992 | 0.978 | 0.997 | 137.7 | -98.3 |
| wavelet-HHL_ngtdm_Coarseness | 10.70 | 29.65 | 0.943 | 0.775 | 0.982 | 19.2 | -40.1 |
| wavelet-HHL_ngtdm_Complexity | 38.72 | 107.33 | 0.961 | 0.89 | 0.987 | 94.1 | -120.5 |
| wavelet-HHL_ngtdm_Contrast | 34.31 | 95.09 | 0.784 | 0.48 | 0.921 | 99.5 | -90.6 |
| wavelet-HHL_ngtdm_Strength | 32.04 | 88.82 | 0.973 | 0.924 | 0.991 | 70.1 | -107.5 |
| wavelet-HHH_firstorder_10Percentile | 11.33 | 31.41 | 0.959 | 0.884 | 0.986 | 26.2 | -36.6 |
| wavelet-HHH_firstorder_90Percentile | 12.41 | 34.41 | 0.958 | 0.88 | 0.986 | 29.3 | -39.5 |
| wavelet-HHH_firstorder_Energy | 31.49 | 87.29 | 0.941 | 0.84 | 0.98 | 83.1 | -91.4 |
| wavelet-HHH_firstorder_Entropy | 0.28 | 0.78 | 0.763 | 0.44 | 0.913 | 0.8 | -0.8 |
| wavelet-HHH_firstorder_InterquartileRange | 12.43 | 34.45 | 0.937 | 0.816 | 0.979 | 24.8 | -44.1 |
| wavelet-HHH_firstorder_Kurtosis | 20.31 | 56.28 | 0.698 | 0.32 | 0.886 | 46.6 | -66 |
| wavelet-HHH_firstorder_Maximum | 17.29 | 47.92 | 0.981 | 0.945 | 0.993 | 42.1 | -53.8 |
| wavelet-HHH_firstorder_MeanAbsoluteDeviation | 12.03 | 33.36 | 0.949 | 0.855 | 0.983 | 25.8 | -40.9 |
| wavelet-HHH_firstorder_Mean | 83.39 | 231.16 | 0.883 | 0.695 | 0.959 | 251.3 | -211 |
| wavelet-HHH_firstorder_Median | 641.25 | 1777.45 | 0.802 | 0.517 | 0.928 | 2001.4 | -1553.5 |
| wavelet-HHH_firstorder_Minimum | 23.81 | 66.01 | 0.934 | 0.815 | 0.977 | 56.8 | -75.2 |
| wavelet-HHH_firstorder_Range | 19.64 | 54.43 | 0.964 | 0.895 | 0.988 | 46.1 | -62.8 |
| wavelet-HHH_firstorder_RobustMeanAbsoluteDeviation | 11.23 | 31.13 | 0.938 | 0.823 | 0.979 | 23.1 | -39.2 |
| wavelet-HHH_firstorder_RootMeanSquared | 13.24 | 36.70 | 0.953 | 0.864 | 0.984 | 28.7 | -44.7 |
| wavelet-HHH_firstorder_Skewness | 33.07 | 91.66 | 0.509 | 0.054 | 0.798 | 79.7 | -103.7 |
| wavelet-HHH_firstorder_TotalEnergy | 31.49 | 87.29 | 0.941 | 0.84 | 0.98 | 83.1 | -91.4 |
| wavelet-HHH_firstorder_Uniformity | 0.37 | 1.04 | 0.764 | 0.442 | 0.913 | 1 | -1 |
| wavelet-HHH_firstorder_Variance | 25.47 | 70.59 | 0.905 | 0.747 | 0.967 | 55.3 | -85.9 |
| wavelet-HHH_glcm_Autocorrelation | 2.78 | 7.70 | 0.805 | 0.524 | 0.929 | 9.1 | -6.3 |
| wavelet-HHH_glcm_ClusterProminence | 2.23 | 6.17 | 0.587 | 0.148 | 0.837 | 7.1 | -5.2 |
| wavelet-HHH_glcm_ClusterShade | 141.71 | 392.80 | 0.85 | 0.619 | 0.946 | 428.1 | -357.5 |
| wavelet-HHH_glcm_ClusterTendency | 2.94 | 8.15 | 0.225 | -0.243 | 0.635 | 9.9 | -6.4 |
| wavelet-HHH_glcm_Contrast | 3.87 | 10.72 | 0.558 | 0.098 | 0.825 | 9.3 | -12.2 |
| wavelet-HHH_glcm_Correlation | 10.70 | 29.65 | 0.463 | -0.013 | 0.776 | 34.5 | -24.8 |
| wavelet-HHH_glcm_DifferenceAverage | 3.87 | 10.72 | 0.558 | 0.098 | 0.825 | 9.3 | -12.2 |
| wavelet-HHH_glcm_DifferenceEntropy | 1.65 | 4.58 | 0.688 | 0.302 | 0.882 | 4.1 | -5 |
| wavelet-HHH_glcm_DifferenceVariance | 2.18 | 6.05 | 0.687 | 0.301 | 0.882 | 5.4 | -6.7 |
| wavelet-HHH_glcm_Id | 0.86 | 2.39 | 0.558 | 0.098 | 0.825 | 2.7 | -2.1 |
| wavelet-HHH_glcm_Idm | 0.86 | 2.39 | 0.558 | 0.098 | 0.825 | 2.7 | -2.1 |
| wavelet-HHH_glcm_Idmn | 0.30 | 0.84 | 0.558 | 0.098 | 0.825 | 1 | -0.7 |
| wavelet-HHH_glcm_Idn | 0.53 | 1.48 | 0.558 | 0.098 | 0.825 | 1.7 | -1.3 |
| wavelet-HHH_glcm_Imc1 | 17.38 | 48.19 | 0.676 | 0.287 | 0.877 | 55 | -41.3 |
| wavelet-HHH_glcm_Imc2 | 9.59 | 26.58 | 0.53 | 0.081 | 0.809 | 32 | -21.1 |
| wavelet-HHH_glcm_InverseVariance | 3.87 | 10.72 | 0.558 | 0.098 | 0.825 | 9.3 | -12.2 |
| wavelet-HHH_glcm_JointAverage | 1.35 | 3.74 | 0.814 | 0.543 | 0.933 | 4.4 | -3.1 |
| wavelet-HHH_glcm_JointEnergy | 3.22 | 8.92 | 0.587 | 0.141 | 0.838 | 8.6 | -9.3 |
| wavelet-HHH_glcm_JointEntropy | 1.45 | 4.03 | 0.603 | 0.164 | 0.845 | 4.2 | -3.9 |
| wavelet-HHH_glcm_MCC | 10.00 | 27.72 | 0.564 | 0.125 | 0.826 | 33.2 | -22.2 |
| wavelet-HHH_glcm_MaximumProbability | 4.96 | 13.76 | 0.71 | 0.342 | 0.891 | 13.3 | -14.2 |
| wavelet-HHH_glcm_SumAverage | 1.35 | 3.74 | 0.814 | 0.543 | 0.933 | 4.4 | -3.1 |
| wavelet-HHH_glcm_SumEntropy | 1.56 | 4.33 | 0.365 | -0.15 | 0.728 | 4.8 | -3.8 |
| wavelet-HHH_glcm_SumSquares | 1.42 | 3.94 | 0.384 | -0.117 | 0.736 | 4.5 | -3.3 |
| wavelet-HHH_gldm_DependenceEntropy | 1.74 | 4.82 | 0.382 | -0.113 | 0.734 | 5.6 | -4 |
| wavelet-HHH_gldm_DependenceNonUniformity | 12.73 | 35.29 | 0.997 | 0.992 | 0.999 | 46.6 | -24 |
| wavelet-HHH_gldm_DependenceNonUniformityNormalized | 4.26 | 11.81 | 0.603 | 0.164 | 0.845 | 11.3 | -12.3 |
| wavelet-HHH_gldm_DependenceVariance | 5.89 | 16.33 | 0.739 | 0.395 | 0.903 | 16.6 | -16.1 |
| wavelet-HHH_gldm_GrayLevelNonUniformity | 12.00 | 33.25 | 0.996 | 0.99 | 0.999 | 45.1 | -21.4 |
| wavelet-HHH_gldm_GrayLevelVariance | 0.39 | 1.08 | 0.764 | 0.442 | 0.913 | 1.1 | -1.1 |
| wavelet-HHH_gldm_HighGrayLevelEmphasis | 1.96 | 5.43 | 0.793 | 0.5 | 0.925 | 6.1 | -4.7 |
| wavelet-HHH_gldm_LargeDependenceEmphasis | 4.29 | 11.89 | 0.918 | 0.643 | 0.976 | 16.5 | -7.2 |
| wavelet-HHH_gldm_LargeDependenceHighGrayLevelEmphasis | 7.20 | 19.97 | 0.904 | 0.607 | 0.971 | 27.4 | -12.5 |
| wavelet-HHH_gldm_LargeDependenceLowGrayLevelEmphasis | 5.69 | 15.78 | 0.862 | 0.648 | 0.951 | 18.4 | -13.1 |
| wavelet-HHH_gldm_LowGrayLevelEmphasis | 1.95 | 5.41 | 0.793 | 0.5 | 0.925 | 4.7 | -6.1 |
| wavelet-HHH_gldm_SmallDependenceEmphasis | 17.88 | 49.56 | 0.487 | 0 | 0.791 | 47 | -52.2 |
| wavelet-HHH_gldm_SmallDependenceHighGrayLevelEmphasis | 13.66 | 37.87 | 0.765 | 0.444 | 0.914 | 37.4 | -38.3 |
| wavelet-HHH_gldm_SmallDependenceLowGrayLevelEmphasis | 24.96 | 69.18 | 0.079 | -0.432 | 0.553 | 65.1 | -73.3 |
| wavelet-HHH_glrlm_GrayLevelNonUniformity | 10.58 | 29.33 | 0.997 | 0.99 | 0.999 | 38.8 | -19.8 |
| wavelet-HHH_glrlm_GrayLevelNonUniformityNormalized | 0.61 | 1.70 | 0 | -0.497 | 0.497 | 1.7 | -1.7 |
| wavelet-HHH_glrlm_GrayLevelVariance | 0.63 | 1.74 | 0 | -0.497 | 0.497 | 1.7 | -1.8 |
| wavelet-HHH_glrlm_HighGrayLevelRunEmphasis | 2.38 | 6.59 | 0.505 | 0.024 | 0.8 | 6.7 | -6.5 |
| wavelet-HHH_glrlm_LongRunEmphasis | 5.00 | 13.85 | 0.944 | 0.75 | 0.983 | 19.1 | -8.6 |
| wavelet-HHH_glrlm_LongRunHighGrayLevelEmphasis | 5.42 | 15.04 | 0.946 | 0.676 | 0.985 | 21.7 | -8.3 |
| wavelet-HHH_glrlm_LongRunLowGrayLevelEmphasis | 6.20 | 17.18 | 0.915 | 0.764 | 0.971 | 21.3 | -13.1 |
| wavelet-HHH_glrlm_LowGrayLevelRunEmphasis | 2.52 | 6.97 | 0.505 | 0.024 | 0.8 | 6.9 | -7.1 |
| wavelet-HHH_glrlm_RunEntropy | 1.86 | 5.14 | 0.899 | 0.507 | 0.971 | 7.4 | -2.8 |
| wavelet-HHH_glrlm_RunLengthNonUniformity | 9.77 | 27.08 | 0.996 | 0.99 | 0.999 | 33.2 | -21 |
| wavelet-HHH_glrlm_RunLengthNonUniformityNormalized | 3.48 | 9.66 | 0.86 | 0.511 | 0.956 | 6.1 | -13.2 |
| wavelet-HHH_glrlm_RunPercentage | 2.27 | 6.31 | 0.907 | 0.618 | 0.972 | 3.9 | -8.7 |
| wavelet-HHH_glrlm_RunVariance | 7.44 | 20.61 | 0.951 | 0.799 | 0.985 | 27.9 | -13.4 |
| wavelet-HHH_glrlm_ShortRunEmphasis | 2.72 | 7.54 | 0.87 | 0.59 | 0.957 | 5.1 | -10 |
| wavelet-HHH_glrlm_ShortRunHighGrayLevelEmphasis | 3.59 | 9.94 | 0.866 | 0.614 | 0.955 | 7.2 | -12.7 |
| wavelet-HHH_glrlm_ShortRunLowGrayLevelEmphasis | 6.24 | 17.31 | 0.633 | 0.211 | 0.858 | 15.6 | -19 |
| wavelet-HHH_glszm_GrayLevelNonUniformity | 45.37 | 125.75 | 0.505 | 0.023 | 0.799 | 139.4 | -112.1 |
| wavelet-HHH_glszm_GrayLevelNonUniformityNormalized | 7.92 | 21.97 | 0 | -0.497 | 0.497 | 23.2 | -20.8 |
| wavelet-HHH_glszm_GrayLevelVariance | 9.80 | 27.17 | 0 | -0.497 | 0.497 | 26.6 | -27.8 |
| wavelet-HHH_glszm_HighGrayLevelZoneEmphasis | 15.19 | 42.11 | 0.311 | -0.197 | 0.696 | 48.1 | -36.1 |
| wavelet-HHH_glszm_LargeAreaEmphasis | 50.24 | 139.27 | 0.831 | 0.579 | 0.939 | 149.5 | -129 |
| wavelet-HHH_glszm_LargeAreaHighGrayLevelEmphasis | 49.09 | 136.08 | 0.837 | 0.593 | 0.942 | 147.1 | -125.1 |
| wavelet-HHH_glszm_LargeAreaLowGrayLevelEmphasis | 51.41 | 142.49 | 0.824 | 0.564 | 0.937 | 151.9 | -133.1 |
| wavelet-HHH_glszm_LowGrayLevelZoneEmphasis | 14.00 | 38.81 | 0.311 | -0.197 | 0.696 | 32.5 | -45.1 |
| wavelet-HHH_glszm_SizeZoneNonUniformity | 27.86 | 77.21 | 0.682 | 0.293 | 0.88 | 72.3 | -82.1 |
| wavelet-HHH_glszm_SizeZoneNonUniformityNormalized | 28.97 | 80.29 | 0 | -0.374 | 0.448 | 62.3 | -98.3 |
| wavelet-HHH_glszm_SmallAreaEmphasis | 129.51 | 358.99 | 0 | -0.497 | 0.497 | 402 | -316 |
| wavelet-HHH_glszm_SmallAreaHighGrayLevelEmphasis | 129.11 | 357.88 | 0 | -0.497 | 0.497 | 398.5 | -317.2 |
| wavelet-HHH_glszm_SmallAreaLowGrayLevelEmphasis | 130.30 | 361.18 | 0.003 | -0.495 | 0.499 | 409 | -313.4 |
| wavelet-HHH_glszm_ZoneEntropy | 34.65 | 96.05 | 0 | -0.445 | 0.479 | 112.9 | -79.2 |
| wavelet-HHH_glszm_ZonePercentage | 43.66 | 121.01 | 0.636 | 0.216 | 0.86 | 122.6 | -119.4 |
| wavelet-HHH_glszm_ZoneVariance | 108.30 | 300.19 | 0.897 | 0.728 | 0.964 | 362.1 | -238.3 |
| wavelet-HHH_ngtdm_Busyness | 18.71 | 51.85 | 0.992 | 0.976 | 0.997 | 58.6 | -45.1 |
| wavelet-HHH_ngtdm_Coarseness | 11.83 | 32.78 | 0.94 | 0.772 | 0.981 | 22.1 | -43.4 |
| wavelet-HHH_ngtdm_Complexity | 5.25 | 14.54 | 0.371 | -0.144 | 0.731 | 13.4 | -15.7 |
| wavelet-HHH_ngtdm_Contrast | 5.24 | 14.52 | 0.401 | -0.108 | 0.747 | 13.2 | -15.9 |
| wavelet-HHH_ngtdm_Strength | 11.84 | 32.83 | 0.94 | 0.778 | 0.981 | 22.3 | -43.3 |
| wavelet-LLL_firstorder_10Percentile | 4.34 | 12.02 | 0.587 | 0.149 | 0.837 | 15.2 | -8.8 |
| wavelet-LLL_firstorder_90Percentile | 7.13 | 19.77 | 0.977 | 0.936 | 0.992 | 20.4 | -19.2 |
| wavelet-LLL_firstorder_Energy | 21.75 | 60.29 | 0.965 | 0.901 | 0.988 | 72.8 | -47.8 |
| wavelet-LLL_firstorder_Entropy | 1.80 | 4.98 | 0.988 | 0.965 | 0.996 | 5 | -5 |
| wavelet-LLL_firstorder_InterquartileRange | 11.50 | 31.88 | 0.972 | 0.92 | 0.99 | 31.9 | -31.9 |
| wavelet-LLL_firstorder_Kurtosis | 6.25 | 17.33 | 0.947 | 0.846 | 0.982 | 20.6 | -14 |
| wavelet-LLL_firstorder_Maximum | 8.04 | 22.30 | 0.987 | 0.963 | 0.996 | 24.4 | -20.2 |
| wavelet-LLL_firstorder_MeanAbsoluteDeviation | 10.02 | 27.77 | 0.981 | 0.946 | 0.993 | 27.1 | -28.4 |
| wavelet-LLL_firstorder_Mean | 5.77 | 16.00 | 0.966 | 0.904 | 0.988 | 16.7 | -15.3 |
| wavelet-LLL_firstorder_Median | 5.14 | 14.25 | 0.936 | 0.826 | 0.978 | 14.7 | -13.8 |
| wavelet-LLL_firstorder_Minimum | 12.64 | 35.03 | 0.709 | 0.321 | 0.892 | 43.5 | -26.5 |
| wavelet-LLL_firstorder_Range | 10.21 | 28.30 | 0.988 | 0.967 | 0.996 | 29.3 | -27.3 |
| wavelet-LLL_firstorder_RobustMeanAbsoluteDeviation | 10.12 | 28.05 | 0.977 | 0.935 | 0.992 | 27.2 | -29 |
| wavelet-LLL_firstorder_RootMeanSquared | 6.36 | 17.63 | 0.974 | 0.926 | 0.991 | 18.3 | -17 |
| wavelet-LLL_firstorder_Skewness | 33.51 | 92.88 | 0.938 | 0.83 | 0.979 | 90.3 | -95.4 |
| wavelet-LLL_firstorder_TotalEnergy | 21.75 | 60.29 | 0.965 | 0.901 | 0.988 | 72.8 | -47.8 |
| wavelet-LLL_firstorder_Uniformity | 8.81 | 24.42 | 0.99 | 0.971 | 0.997 | 26.2 | -22.6 |
| wavelet-LLL_firstorder_Variance | 19.86 | 55.04 | 0.98 | 0.943 | 0.993 | 54.4 | -55.7 |
| wavelet-LLL_glcm_Autocorrelation | 16.42 | 45.53 | 0.957 | 0.881 | 0.985 | 42.4 | -48.6 |
| wavelet-LLL_glcm_ClusterProminence | 41.60 | 115.30 | 0.981 | 0.946 | 0.993 | 118.2 | -112.4 |
| wavelet-LLL_glcm_ClusterShade | 76.69 | 212.57 | 0.988 | 0.966 | 0.996 | 199.9 | -225.2 |
| wavelet-LLL_glcm_ClusterTendency | 21.54 | 59.71 | 0.983 | 0.951 | 0.994 | 60 | -59.5 |
| wavelet-LLL_glcm_Contrast | 16.95 | 46.97 | 0.968 | 0.91 | 0.989 | 42.7 | -51.2 |
| wavelet-LLL_glcm_Correlation | 3.17 | 8.79 | 0.97 | 0.906 | 0.99 | 10.7 | -6.9 |
| wavelet-LLL_glcm_DifferenceAverage | 8.18 | 22.66 | 0.978 | 0.938 | 0.993 | 19.9 | -25.4 |
| wavelet-LLL_glcm_DifferenceEntropy | 2.37 | 6.57 | 0.986 | 0.961 | 0.995 | 6.3 | -6.9 |
| wavelet-LLL_glcm_DifferenceVariance | 19.00 | 52.67 | 0.983 | 0.952 | 0.994 | 50.3 | -55 |
| wavelet-LLL_glcm_Id | 6.31 | 17.49 | 0.991 | 0.975 | 0.997 | 19.9 | -15.1 |
| wavelet-LLL_glcm_Idm | 9.82 | 27.22 | 0.992 | 0.978 | 0.997 | 29.4 | -25.1 |
| wavelet-LLL_glcm_Idmn | 0.21 | 0.57 | 0.956 | 0.851 | 0.986 | 0.7 | -0.4 |
| wavelet-LLL_glcm_Idn | 0.50 | 1.39 | 0.956 | 0.854 | 0.986 | 1.8 | -1 |
| wavelet-LLL_glcm_Imc1 | 5.09 | 14.11 | 0.974 | 0.805 | 0.993 | 9.1 | -19.2 |
| wavelet-LLL_glcm_Imc2 | 1.15 | 3.19 | 0.85 | 0.622 | 0.947 | 2.7 | -3.6 |
| wavelet-LLL_glcm_InverseVariance | 11.33 | 31.40 | 0.991 | 0.976 | 0.997 | 34.1 | -28.7 |
| wavelet-LLL_glcm_JointAverage | 7.90 | 21.89 | 0.969 | 0.912 | 0.989 | 19.5 | -24.3 |
| wavelet-LLL_glcm_JointEnergy | 13.43 | 37.23 | 0.957 | 0.82 | 0.987 | 27.5 | -47 |
| wavelet-LLL_glcm_JointEntropy | 2.06 | 5.70 | 0.978 | 0.926 | 0.993 | 7.5 | -3.9 |
| wavelet-LLL_glcm_MCC | 3.04 | 8.44 | 0.913 | 0.767 | 0.97 | 7.7 | -9.2 |
| wavelet-LLL_glcm_MaximumProbability | 20.00 | 55.45 | 0.942 | 0.84 | 0.98 | 56.9 | -54 |
| wavelet-LLL_glcm_SumAverage | 7.90 | 21.89 | 0.969 | 0.912 | 0.989 | 19.5 | -24.3 |
| wavelet-LLL_glcm_SumEntropy | 2.17 | 6.02 | 0.982 | 0.949 | 0.994 | 6.7 | -5.4 |
| wavelet-LLL_glcm_SumSquares | 20.71 | 57.41 | 0.982 | 0.948 | 0.994 | 57 | -57.8 |
| wavelet-LLL_gldm_DependenceEntropy | 1.64 | 4.53 | 0.98 | 0.943 | 0.993 | 5.2 | -3.9 |
| wavelet-LLL_gldm_DependenceNonUniformity | 14.78 | 40.96 | 0.993 | 0.98 | 0.998 | 52.7 | -29.2 |
| wavelet-LLL_gldm_DependenceNonUniformityNormalized | 6.97 | 19.33 | 0.941 | 0.837 | 0.979 | 19.3 | -19.4 |
| wavelet-LLL_gldm_DependenceVariance | 22.14 | 61.38 | 0.959 | 0.88 | 0.986 | 71.6 | -51.2 |
| wavelet-LLL_gldm_GrayLevelNonUniformity | 9.39 | 26.03 | 0.98 | 0.914 | 0.994 | 39.7 | -12.4 |
| wavelet-LLL_gldm_GrayLevelVariance | 19.89 | 55.12 | 0.98 | 0.943 | 0.993 | 54.5 | -55.8 |
| wavelet-LLL_gldm_HighGrayLevelEmphasis | 16.58 | 45.95 | 0.959 | 0.885 | 0.986 | 42.8 | -49.1 |
| wavelet-LLL_gldm_LargeDependenceEmphasis | 11.29 | 31.30 | 0.946 | 0.851 | 0.981 | 34.4 | -28.2 |
| wavelet-LLL_gldm_LargeDependenceHighGrayLevelEmphasis | 23.65 | 65.55 | 0.937 | 0.827 | 0.978 | 62.6 | -68.5 |
| wavelet-LLL_gldm_LargeDependenceLowGrayLevelEmphasis | 25.71 | 71.26 | 0.827 | 0.569 | 0.938 | 83.9 | -58.6 |
| wavelet-LLL_gldm_LowGrayLevelEmphasis | 20.09 | 55.68 | 0.844 | 0.607 | 0.944 | 62.4 | -48.9 |
| wavelet-LLL_gldm_SmallDependenceEmphasis | 7.59 | 21.04 | 0.931 | 0.813 | 0.976 | 20.9 | -21.2 |
| wavelet-LLL_gldm_SmallDependenceHighGrayLevelEmphasis | 21.07 | 58.41 | 0.961 | 0.892 | 0.987 | 55.3 | -61.5 |
| wavelet-LLL_gldm_SmallDependenceLowGrayLevelEmphasis | 19.98 | 55.37 | 0.871 | 0.667 | 0.954 | 58.5 | -52.3 |
| wavelet-LLL_glrlm_GrayLevelNonUniformity | 9.24 | 25.60 | 0.981 | 0.918 | 0.994 | 39 | -12.2 |
| wavelet-LLL_glrlm_GrayLevelNonUniformityNormalized | 8.67 | 24.02 | 0.99 | 0.972 | 0.997 | 25.7 | -22.4 |
| wavelet-LLL_glrlm_GrayLevelVariance | 19.80 | 54.88 | 0.98 | 0.944 | 0.993 | 54.2 | -55.6 |
| wavelet-LLL_glrlm_HighGrayLevelRunEmphasis | 16.55 | 45.87 | 0.96 | 0.888 | 0.986 | 42.8 | -49 |
| wavelet-LLL_glrlm_LongRunEmphasis | 1.39 | 3.85 | 0.913 | 0.768 | 0.97 | 4.2 | -3.5 |
| wavelet-LLL_glrlm_LongRunHighGrayLevelEmphasis | 16.51 | 45.77 | 0.959 | 0.887 | 0.986 | 42.9 | -48.6 |
| wavelet-LLL_glrlm_LongRunLowGrayLevelEmphasis | 20.26 | 56.15 | 0.843 | 0.606 | 0.944 | 63.4 | -48.9 |
| wavelet-LLL_glrlm_LowGrayLevelRunEmphasis | 19.93 | 55.23 | 0.849 | 0.618 | 0.946 | 61.9 | -48.5 |
| wavelet-LLL_glrlm_RunEntropy | 1.74 | 4.82 | 0.987 | 0.964 | 0.996 | 5 | -4.7 |
| wavelet-LLL_glrlm_RunLengthNonUniformity | 12.01 | 33.28 | 0.996 | 0.989 | 0.999 | 44.9 | -21.6 |
| wavelet-LLL_glrlm_RunLengthNonUniformityNormalized | 0.65 | 1.80 | 0.953 | 0.87 | 0.984 | 1.7 | -1.9 |
| wavelet-LLL_glrlm_RunPercentage | 0.38 | 1.05 | 0.945 | 0.848 | 0.981 | 1 | -1.1 |
| wavelet-LLL_glrlm_RunVariance | 19.54 | 54.17 | 0.878 | 0.684 | 0.957 | 58.8 | -49.6 |
| wavelet-LLL_glrlm_ShortRunEmphasis | 0.27 | 0.75 | 0.949 | 0.858 | 0.982 | 0.7 | -0.8 |
| wavelet-LLL_glrlm_ShortRunHighGrayLevelEmphasis | 16.59 | 45.98 | 0.96 | 0.889 | 0.986 | 42.8 | -49.1 |
| wavelet-LLL_glrlm_ShortRunLowGrayLevelEmphasis | 19.85 | 55.02 | 0.85 | 0.621 | 0.947 | 61.6 | -48.4 |
| wavelet-LLL_glszm_GrayLevelNonUniformity | 9.16 | 25.39 | 0.99 | 0.949 | 0.997 | 37.3 | -13.4 |
| wavelet-LLL_glszm_GrayLevelNonUniformityNormalized | 7.65 | 21.21 | 0.99 | 0.973 | 0.997 | 21.9 | -20.5 |
| wavelet-LLL_glszm_GrayLevelVariance | 19.03 | 52.76 | 0.984 | 0.954 | 0.995 | 51.2 | -54.3 |
| wavelet-LLL_glszm_HighGrayLevelZoneEmphasis | 16.95 | 46.99 | 0.969 | 0.913 | 0.989 | 43.7 | -50.3 |
| wavelet-LLL_glszm_LargeAreaEmphasis | 15.62 | 43.29 | 0.795 | 0.503 | 0.925 | 46.5 | -40.1 |
| wavelet-LLL_glszm_LargeAreaHighGrayLevelEmphasis | 23.31 | 64.61 | 0.955 | 0.874 | 0.984 | 63.2 | -66 |
| wavelet-LLL_glszm_LargeAreaLowGrayLevelEmphasis | 24.51 | 67.93 | 0.77 | 0.455 | 0.915 | 79.3 | -56.6 |
| wavelet-LLL_glszm_LowGrayLevelZoneEmphasis | 18.92 | 52.46 | 0.902 | 0.741 | 0.966 | 57.8 | -47.1 |
| wavelet-LLL_glszm_SizeZoneNonUniformity | 18.60 | 51.54 | 0.995 | 0.985 | 0.998 | 63.3 | -39.8 |
| wavelet-LLL_glszm_SizeZoneNonUniformityNormalized | 8.55 | 23.69 | 0.899 | 0.732 | 0.964 | 24.4 | -22.9 |
| wavelet-LLL_glszm_SmallAreaEmphasis | 4.38 | 12.13 | 0.879 | 0.685 | 0.957 | 12.4 | -11.9 |
| wavelet-LLL_glszm_SmallAreaHighGrayLevelEmphasis | 20.22 | 56.04 | 0.97 | 0.916 | 0.99 | 53.1 | -59 |
| wavelet-LLL_glszm_SmallAreaLowGrayLevelEmphasis | 21.17 | 58.68 | 0.901 | 0.739 | 0.965 | 61.2 | -56.1 |
| wavelet-LLL_glszm_ZoneEntropy | 1.55 | 4.31 | 0.983 | 0.953 | 0.994 | 4.8 | -3.9 |
| wavelet-LLL_glszm_ZonePercentage | 6.12 | 16.97 | 0.939 | 0.832 | 0.979 | 16.4 | -17.5 |
| wavelet-LLL_glszm_ZoneVariance | 30.05 | 83.28 | 0.785 | 0.484 | 0.922 | 93.2 | -73.4 |
| wavelet-LLL_ngtdm_Busyness | 12.66 | 35.10 | 0.868 | 0.657 | 0.953 | 48.7 | -21.5 |
| wavelet-LLL_ngtdm_Coarseness | 11.22 | 31.09 | 0.944 | 0.773 | 0.983 | 21.5 | -40.7 |
| wavelet-LLL_ngtdm_Complexity | 23.55 | 65.29 | 0.958 | 0.884 | 0.986 | 65.2 | -65.4 |
| wavelet-LLL_ngtdm_Contrast | 14.86 | 41.18 | 0.965 | 0.894 | 0.989 | 33.2 | -49.2 |
| wavelet-LLL_ngtdm_Strength | 15.80 | 43.80 | 0.987 | 0.964 | 0.996 | 40.5 | -47.1 |
