# Supplementary material for: Prospective inter- and intra-tracer repeatability analysis of radiomics features in [68Ga]Ga-PSMA-11 and [18F]F-PSMA-1007 PET scans in metastatic prostate cancer
Source: Br J Radiol. 2023 Oct 24;96(1152):20221178. doi: 10.1259/bjr.20221178 (PMC10646662; doi:10.1259/bjr.20221178)
Supplement: Supplementary Table 3. [file bjr.20221178.suppl-05.docx]

**Supplementary Table S3**

Tabulated below are the raw repeatability metrics for the inter-tracer group, including: intraclass correlation coefficients (ICCs), symmetric repeatability coefficients (RCs), within-subject coefficient of variation (wCV) values, upper and lower limits of agreement (LOA).

| **Biomarker** | **wCV (%)** | **Symmetric RC (%)** | **ICC** | **ICC 95% Lower** | **ICC 95% Upper** | **Upper LOA** | **Lower LOA** |
| --- | --- | --- | --- | --- | --- | --- | --- |
| original_shape_Elongation | 9.42 | 26.12 | 0.763 | 0.584 | 0.872 | 29.8 | -22.4 |
| original_shape_Flatness | 9.02 | 24.99 | 0.874 | 0.715 | 0.94 | 31.5 | -18.5 |
| original_shape_LeastAxisLength | 5.93 | 16.44 | 0.982 | 0.678 | 0.995 | 28.2 | -4.6 |
| original_shape_MajorAxisLength | 6.83 | 18.92 | 0.997 | 0.991 | 0.999 | 24.2 | -13.6 |
| original_shape_Maximum2DDiameterColumn | 9.10 | 25.24 | 0.994 | 0.984 | 0.997 | 31.5 | -18.9 |
| original_shape_Maximum2DDiameterRow | 7.17 | 19.87 | 0.993 | 0.969 | 0.998 | 28 | -11.8 |
| original_shape_Maximum2DDiameterSlice | 7.05 | 19.53 | 0.994 | 0.973 | 0.998 | 27.2 | -11.9 |
| original_shape_Maximum3DDiameter | 7.26 | 20.12 | 0.991 | 0.968 | 0.996 | 27.6 | -12.6 |
| original_shape_MeshVolume | 16.01 | 44.36 | 0.987 | 0.973 | 0.994 | 73.8 | -15 |
| original_shape_MinorAxisLength | 9.77 | 27.09 | 0.995 | 0.976 | 0.998 | 36.1 | -18 |
| original_shape_Sphericity | 4.49 | 12.45 | 0.907 | 0.827 | 0.952 | 13.9 | -11 |
| original_shape_SurfaceArea | 12.09 | 33.52 | 0.996 | 0.989 | 0.998 | 51.9 | -15.2 |
| original_shape_SurfaceVolumeRatio | 6.79 | 18.81 | 0.852 | 0.286 | 0.951 | 7.4 | -30.3 |
| original_shape_VoxelVolume | 15.82 | 43.86 | 0.987 | 0.974 | 0.994 | 72.8 | -14.9 |
| exponential_firstorder_10Percentile | 9.36 | 25.95 | 0 | -0.091 | 0.141 | 10.6 | -41.3 |
| exponential_firstorder_90Percentile | 26.41 | 73.19 | 0.514 | 0.08 | 0.755 | 39.8 | -106.6 |
| exponential_firstorder_Energy | 48.42 | 134.23 | 0.678 | 0.456 | 0.821 | 111.6 | -156.8 |
| exponential_firstorder_Entropy | 27.10 | 75.11 | 0.54 | 0.048 | 0.784 | 47.5 | -102.7 |
| exponential_firstorder_InterquartileRange | 41.86 | 116.02 | 0.448 | 0.043 | 0.705 | 60.6 | -171.4 |
| exponential_firstorder_Kurtosis | 21.50 | 59.59 | 0.795 | 0.629 | 0.891 | 66.6 | -52.6 |
| exponential_firstorder_Maximum | 39.33 | 109.03 | 0.782 | 0.616 | 0.882 | 77.5 | -140.5 |
| exponential_firstorder_MeanAbsoluteDeviation | 45.83 | 127.04 | 0.732 | 0.45 | 0.867 | 76.2 | -177.9 |
| exponential_firstorder_Mean | 18.80 | 52.12 | 0.385 | -0.035 | 0.67 | 26.2 | -78 |
| exponential_firstorder_Median | 14.69 | 40.73 | 0.033 | -0.097 | 0.214 | 16 | -65.4 |
| exponential_firstorder_Minimum | 6.72 | 18.64 | 0 | -0.124 | 0.178 | 8.7 | -28.6 |
| exponential_firstorder_Range | 52.72 | 146.14 | 0.785 | 0.62 | 0.884 | 101.2 | -191.1 |
| exponential_firstorder_RobustMeanAbsoluteDeviation | 42.83 | 118.72 | 0.514 | 0.102 | 0.75 | 64.5 | -173 |
| exponential_firstorder_RootMeanSquared | 22.46 | 62.24 | 0.659 | 0.305 | 0.832 | 35.8 | -88.6 |
| exponential_firstorder_Skewness | 22.02 | 61.03 | 0.877 | 0.767 | 0.936 | 65.9 | -56.1 |
| exponential_firstorder_TotalEnergy | 48.42 | 134.23 | 0.678 | 0.456 | 0.821 | 111.6 | -156.8 |
| exponential_firstorder_Uniformity | 32.58 | 90.32 | 0.527 | 0.134 | 0.754 | 139.7 | -41 |
| exponential_firstorder_Variance | 74.23 | 205.75 | 0.792 | 0.632 | 0.888 | 126.5 | -285 |
| exponential_glcm_Autocorrelation | 60.05 | 166.45 | 0.8 | 0.643 | 0.892 | 94 | -238.9 |
| exponential_glcm_ClusterProminence | 98.75 | 273.71 | 0.297 | -0.023 | 0.564 | 185.8 | -361.6 |
| exponential_glcm_ClusterShade | 94.29 | 261.36 | 0.51 | 0.227 | 0.714 | 173.1 | -349.6 |
| exponential_glcm_ClusterTendency | 74.12 | 205.45 | 0.799 | 0.643 | 0.892 | 128.2 | -282.7 |
| exponential_glcm_Contrast | 68.15 | 188.91 | 0.819 | 0.675 | 0.903 | 102.2 | -275.7 |
| exponential_glcm_Correlation | 19.69 | 54.57 | 0.795 | 0.636 | 0.89 | 54.2 | -55 |
| exponential_glcm_DifferenceAverage | 46.06 | 127.68 | 0.626 | 0.157 | 0.83 | 66.7 | -188.7 |
| exponential_glcm_DifferenceEntropy | 30.08 | 83.37 | 0.528 | 0.041 | 0.776 | 48.6 | -118.1 |
| exponential_glcm_DifferenceVariance | 67.90 | 188.20 | 0.778 | 0.608 | 0.88 | 112.3 | -264.1 |
| exponential_glcm_Id | 17.61 | 48.81 | 0.405 | -0.057 | 0.698 | 76.7 | -20.9 |
| exponential_glcm_Idm | 23.62 | 65.46 | 0.38 | -0.06 | 0.676 | 103.2 | -27.8 |
| exponential_glcm_Idmn | 0.57 | 1.57 | 0.701 | 0.415 | 0.849 | 2 | -1.1 |
| exponential_glcm_Idn | 1.36 | 3.78 | 0.706 | 0.216 | 0.877 | 5.5 | -2.1 |
| exponential_glcm_Imc1 | 29.26 | 81.10 | 0.529 | 0.254 | 0.727 | 82.7 | -79.5 |
| exponential_glcm_Imc2 | 24.64 | 68.29 | 0.635 | 0.387 | 0.796 | 58.2 | -78.4 |
| exponential_glcm_InverseVariance | 32.55 | 90.21 | 0.035 | -0.231 | 0.322 | 105.5 | -74.9 |
| exponential_glcm_JointAverage | 32.21 | 89.29 | 0.519 | 0.032 | 0.771 | 45.5 | -133.1 |
| exponential_glcm_JointEnergy | 53.07 | 147.11 | 0.385 | 0.083 | 0.626 | 239.9 | -54.3 |
| exponential_glcm_JointEntropy | 29.27 | 81.13 | 0.508 | 0.006 | 0.768 | 51 | -111.2 |
| exponential_glcm_MCC | 25.23 | 69.94 | 0.762 | 0.583 | 0.871 | 67.1 | -72.8 |
| exponential_glcm_MaximumProbability | 45.76 | 126.85 | 0.315 | -0.007 | 0.58 | 217.5 | -36.2 |
| exponential_glcm_SumAverage | 32.21 | 89.29 | 0.519 | 0.032 | 0.771 | 45.5 | -133.1 |
| exponential_glcm_SumEntropy | 26.66 | 73.90 | 0.582 | 0.104 | 0.806 | 50.4 | -97.4 |
| exponential_glcm_SumSquares | 73.12 | 202.68 | 0.802 | 0.647 | 0.894 | 124.3 | -281 |
| exponential_gldm_DependenceEntropy | 6.16 | 17.08 | 0.76 | 0.478 | 0.885 | 12.1 | -22.1 |
| exponential_gldm_DependenceNonUniformity | 27.83 | 77.14 | 0.911 | 0.832 | 0.953 | 60.1 | -94.2 |
| exponential_gldm_DependenceNonUniformityNormalized | 23.50 | 65.14 | 0.19 | -0.095 | 0.485 | 19.9 | -110.4 |
| exponential_gldm_DependenceVariance | 43.83 | 121.48 | 0.247 | -0.057 | 0.522 | 205.1 | -37.9 |
| exponential_gldm_GrayLevelNonUniformity | 31.38 | 86.97 | 0.641 | 0.404 | 0.799 | 161.8 | -12.1 |
| exponential_gldm_GrayLevelVariance | 72.25 | 200.26 | 0.792 | 0.631 | 0.888 | 123.9 | -276.6 |
| exponential_gldm_HighGrayLevelEmphasis | 60.82 | 168.58 | 0.795 | 0.636 | 0.89 | 97.9 | -239.3 |
| exponential_gldm_LargeDependenceEmphasis | 46.41 | 128.64 | 0.346 | -0.011 | 0.618 | 216.2 | -41.1 |
| exponential_gldm_LargeDependenceHighGrayLevelEmphasis | 21.51 | 59.61 | 0.839 | 0.708 | 0.914 | 66.7 | -52.6 |
| exponential_gldm_LargeDependenceLowGrayLevelEmphasis | 58.04 | 160.88 | 0.171 | -0.088 | 0.435 | 280.5 | -41.3 |
| exponential_gldm_LowGrayLevelEmphasis | 38.32 | 106.22 | 0.073 | -0.109 | 0.298 | 173.5 | -38.9 |
| exponential_gldm_SmallDependenceEmphasis | 50.43 | 139.78 | 0.331 | -0.053 | 0.622 | 65.5 | -214.1 |
| exponential_gldm_SmallDependenceHighGrayLevelEmphasis | 82.94 | 229.90 | 0.818 | 0.674 | 0.903 | 130.7 | -329.1 |
| exponential_gldm_SmallDependenceLowGrayLevelEmphasis | 33.71 | 93.44 | 0.229 | -0.101 | 0.514 | 91.9 | -95 |
| exponential_glrlm_GrayLevelNonUniformity | 23.41 | 64.89 | 0.841 | 0.707 | 0.916 | 120.8 | -9 |
| exponential_glrlm_GrayLevelNonUniformityNormalized | 32.52 | 90.13 | 0.602 | 0.217 | 0.801 | 133.1 | -47.1 |
| exponential_glrlm_GrayLevelVariance | 71.22 | 197.42 | 0.763 | 0.584 | 0.871 | 125.7 | -269.1 |
| exponential_glrlm_HighGrayLevelRunEmphasis | 62.73 | 173.89 | 0.78 | 0.612 | 0.881 | 104.8 | -242.9 |
| exponential_glrlm_LongRunEmphasis | 29.99 | 83.12 | 0.317 | -0.005 | 0.582 | 129.2 | -37 |
| exponential_glrlm_LongRunHighGrayLevelEmphasis | 47.11 | 130.59 | 0.775 | 0.604 | 0.878 | 91.5 | -169.7 |
| exponential_glrlm_LongRunLowGrayLevelEmphasis | 51.70 | 143.30 | 0.121 | -0.11 | 0.376 | 241.2 | -45.4 |
| exponential_glrlm_LowGrayLevelRunEmphasis | 41.31 | 114.50 | 0.09 | -0.111 | 0.329 | 184.5 | -44.5 |
| exponential_glrlm_RunEntropy | 9.74 | 27.01 | 0.718 | 0.444 | 0.857 | 19.8 | -34.3 |
| exponential_glrlm_RunLengthNonUniformity | 34.06 | 94.40 | 0.823 | 0.682 | 0.905 | 86.7 | -102.1 |
| exponential_glrlm_RunLengthNonUniformityNormalized | 18.15 | 50.32 | 0.369 | -0.054 | 0.661 | 28.2 | -72.4 |
| exponential_glrlm_RunPercentage | 12.37 | 34.30 | 0.344 | -0.066 | 0.642 | 18.3 | -50.3 |
| exponential_glrlm_RunVariance | 44.52 | 123.40 | 0.231 | -0.064 | 0.504 | 210.3 | -36.5 |
| exponential_glrlm_ShortRunEmphasis | 11.06 | 30.65 | 0.416 | -0.001 | 0.688 | 18.9 | -42.4 |
| exponential_glrlm_ShortRunHighGrayLevelEmphasis | 67.88 | 188.14 | 0.781 | 0.614 | 0.882 | 111.6 | -264.7 |
| exponential_glrlm_ShortRunLowGrayLevelEmphasis | 39.79 | 110.29 | 0.084 | -0.113 | 0.319 | 173.8 | -46.8 |
| exponential_glszm_GrayLevelNonUniformity | 47.62 | 132.00 | 0.979 | 0.959 | 0.989 | 128.2 | -135.8 |
| exponential_glszm_GrayLevelNonUniformityNormalized | 57.07 | 158.18 | 0.197 | -0.081 | 0.467 | 217.7 | -98.7 |
| exponential_glszm_GrayLevelVariance | 75.00 | 207.88 | 0.656 | 0.424 | 0.808 | 147.7 | -268.1 |
| exponential_glszm_HighGrayLevelZoneEmphasis | 73.33 | 203.27 | 0.707 | 0.498 | 0.838 | 138.2 | -268.3 |
| exponential_glszm_LargeAreaEmphasis | 61.38 | 170.13 | 0.052 | -0.27 | 0.367 | 301.5 | -38.7 |
| exponential_glszm_LargeAreaHighGrayLevelEmphasis | 51.33 | 142.28 | 0.187 | -0.139 | 0.48 | 246.6 | -38 |
| exponential_glszm_LargeAreaLowGrayLevelEmphasis | 66.62 | 184.66 | 0.013 | -0.303 | 0.331 | 326.6 | -42.8 |
| exponential_glszm_LowGrayLevelZoneEmphasis | 52.32 | 145.03 | 0.21 | -0.069 | 0.479 | 213.9 | -76.2 |
| exponential_glszm_SizeZoneNonUniformity | 74.86 | 207.50 | 0.418 | 0.116 | 0.652 | 141.7 | -273.3 |
| exponential_glszm_SizeZoneNonUniformityNormalized | 31.66 | 87.76 | 0.556 | 0.288 | 0.744 | 68.8 | -106.7 |
| exponential_glszm_SmallAreaEmphasis | 41.15 | 114.05 | 0.391 | 0.092 | 0.63 | 93.5 | -134.6 |
| exponential_glszm_SmallAreaHighGrayLevelEmphasis | 90.66 | 251.30 | 0.703 | 0.493 | 0.836 | 173.8 | -328.8 |
| exponential_glszm_SmallAreaLowGrayLevelEmphasis | 55.15 | 152.87 | 0.477 | 0.158 | 0.701 | 202.4 | -103.4 |
| exponential_glszm_ZoneEntropy | 23.20 | 64.32 | 0.619 | 0.248 | 0.81 | 44.6 | -84.1 |
| exponential_glszm_ZonePercentage | 57.00 | 158.00 | 0.291 | -0.049 | 0.572 | 75 | -241 |
| exponential_glszm_ZoneVariance | 58.12 | 161.10 | 0.053 | -0.271 | 0.369 | 295.2 | -27 |
| exponential_ngtdm_Busyness | 67.35 | 186.69 | 0.154 | -0.133 | 0.435 | 256.1 | -117.3 |
| exponential_ngtdm_Coarseness | 27.05 | 74.96 | 0.086 | -0.245 | 0.399 | 67.1 | -82.8 |
| exponential_ngtdm_Complexity | 82.01 | 227.33 | 0.821 | 0.679 | 0.904 | 132.9 | -321.7 |
| exponential_ngtdm_Contrast | 38.65 | 107.14 | 0.416 | 0.004 | 0.686 | 46.6 | -167.6 |
| exponential_ngtdm_Strength | 84.29 | 233.64 | 0.719 | 0.518 | 0.846 | 166 | -301.2 |
| log-sigma-2-0-mm-3D_firstorder_10Percentile | 17.47 | 48.41 | 0.801 | 0.439 | 0.917 | 65.9 | -31 |
| log-sigma-2-0-mm-3D_firstorder_90Percentile | 222.86 | 617.72 | 0.701 | 0.072 | 0.888 | 643.8 | -591.6 |
| log-sigma-2-0-mm-3D_firstorder_Energy | 31.20 | 86.48 | 0.608 | 0.34 | 0.782 | 148.3 | -24.7 |
| log-sigma-2-0-mm-3D_firstorder_Entropy | 6.57 | 18.22 | 0.874 | 0.509 | 0.953 | 24.3 | -12.1 |
| log-sigma-2-0-mm-3D_firstorder_InterquartileRange | 17.20 | 47.67 | 0.842 | 0.445 | 0.94 | 65 | -30.3 |
| log-sigma-2-0-mm-3D_firstorder_Kurtosis | 13.17 | 36.51 | 0.717 | 0.319 | 0.873 | 53.9 | -19.1 |
| log-sigma-2-0-mm-3D_firstorder_Maximum | 53.73 | 148.93 | 0.707 | 0.195 | 0.88 | 198.3 | -99.5 |
| log-sigma-2-0-mm-3D_firstorder_MeanAbsoluteDeviation | 15.89 | 44.06 | 0.806 | 0.163 | 0.935 | 69.1 | -19 |
| log-sigma-2-0-mm-3D_firstorder_Mean | 33.32 | 92.36 | 0.604 | 0.352 | 0.776 | 83.8 | -101 |
| log-sigma-2-0-mm-3D_firstorder_Median | 170.01 | 471.25 | 0.514 | 0.082 | 0.755 | 414.7 | -527.8 |
| log-sigma-2-0-mm-3D_firstorder_Minimum | 16.93 | 46.94 | 0.721 | 0.265 | 0.881 | 76.9 | -17 |
| log-sigma-2-0-mm-3D_firstorder_Range | 16.27 | 45.09 | 0.72 | 0.22 | 0.885 | 76.2 | -14 |
| log-sigma-2-0-mm-3D_firstorder_RobustMeanAbsoluteDeviation | 16.44 | 45.57 | 0.84 | 0.282 | 0.945 | 66.2 | -25 |
| log-sigma-2-0-mm-3D_firstorder_RootMeanSquared | 15.71 | 43.54 | 0.794 | 0.357 | 0.918 | 61.7 | -25.3 |
| log-sigma-2-0-mm-3D_firstorder_Skewness | 25.42 | 70.46 | 0.773 | 0.126 | 0.921 | 91.3 | -49.7 |
| log-sigma-2-0-mm-3D_firstorder_TotalEnergy | 31.20 | 86.48 | 0.608 | 0.34 | 0.782 | 148.3 | -24.7 |
| log-sigma-2-0-mm-3D_firstorder_Uniformity | 15.41 | 42.71 | 0.878 | 0.772 | 0.936 | 30.9 | -54.5 |
| log-sigma-2-0-mm-3D_firstorder_Variance | 30.07 | 83.35 | 0.64 | 0.269 | 0.823 | 135.8 | -30.9 |
| log-sigma-2-0-mm-3D_glcm_Autocorrelation | 29.38 | 81.43 | 0.474 | 0.173 | 0.694 | 149.9 | -12.9 |
| log-sigma-2-0-mm-3D_glcm_ClusterProminence | 53.23 | 147.56 | 0.214 | -0.096 | 0.495 | 254.8 | -40.3 |
| log-sigma-2-0-mm-3D_glcm_ClusterShade | 61.34 | 170.02 | 0.343 | 0.041 | 0.593 | 263.8 | -76.3 |
| log-sigma-2-0-mm-3D_glcm_ClusterTendency | 30.64 | 84.92 | 0.621 | 0.258 | 0.81 | 141.2 | -28.6 |
| log-sigma-2-0-mm-3D_glcm_Contrast | 29.92 | 82.93 | 0.735 | 0.376 | 0.879 | 121.6 | -44.3 |
| log-sigma-2-0-mm-3D_glcm_Correlation | 5.96 | 16.53 | 0.763 | 0.207 | 0.911 | 23.6 | -9.5 |
| log-sigma-2-0-mm-3D_glcm_DifferenceAverage | 17.29 | 47.92 | 0.859 | 0.491 | 0.946 | 64.3 | -31.6 |
| log-sigma-2-0-mm-3D_glcm_DifferenceEntropy | 8.21 | 22.76 | 0.867 | 0.462 | 0.952 | 31.2 | -14.4 |
| log-sigma-2-0-mm-3D_glcm_DifferenceVariance | 28.59 | 79.24 | 0.633 | 0.298 | 0.812 | 126.5 | -32 |
| log-sigma-2-0-mm-3D_glcm_Id | 9.15 | 25.36 | 0.837 | 0.702 | 0.913 | 21.5 | -29.2 |
| log-sigma-2-0-mm-3D_glcm_Idm | 13.32 | 36.91 | 0.824 | 0.684 | 0.906 | 32.6 | -41.2 |
| log-sigma-2-0-mm-3D_glcm_Idmn | 0.50 | 1.37 | 0.701 | 0.356 | 0.857 | 1.8 | -0.9 |
| log-sigma-2-0-mm-3D_glcm_Idn | 1.26 | 3.48 | 0.721 | 0.315 | 0.876 | 4.8 | -2.2 |
| log-sigma-2-0-mm-3D_glcm_Imc1 | 9.57 | 26.53 | 0.773 | 0.554 | 0.885 | 32.9 | -20.2 |
| log-sigma-2-0-mm-3D_glcm_Imc2 | 3.67 | 10.18 | 0.877 | 0.71 | 0.943 | 12.4 | -7.9 |
| log-sigma-2-0-mm-3D_glcm_InverseVariance | 10.88 | 30.17 | 0.828 | 0.653 | 0.914 | 23.6 | -36.7 |
| log-sigma-2-0-mm-3D_glcm_JointAverage | 16.07 | 44.55 | 0.698 | 0.227 | 0.87 | 80.9 | -8.2 |
| log-sigma-2-0-mm-3D_glcm_JointEnergy | 28.62 | 79.32 | 0.834 | 0.701 | 0.912 | 60.9 | -97.7 |
| log-sigma-2-0-mm-3D_glcm_JointEntropy | 6.62 | 18.34 | 0.875 | 0.52 | 0.953 | 24.2 | -12.5 |
| log-sigma-2-0-mm-3D_glcm_MCC | 4.98 | 13.81 | 0.745 | 0.241 | 0.898 | 19.3 | -8.3 |
| log-sigma-2-0-mm-3D_glcm_MaximumProbability | 31.34 | 86.88 | 0.734 | 0.54 | 0.855 | 81.6 | -92.2 |
| log-sigma-2-0-mm-3D_glcm_SumAverage | 16.07 | 44.55 | 0.698 | 0.227 | 0.87 | 80.9 | -8.2 |
| log-sigma-2-0-mm-3D_glcm_SumEntropy | 5.25 | 14.54 | 0.873 | 0.32 | 0.959 | 20.8 | -8.3 |
| log-sigma-2-0-mm-3D_glcm_SumSquares | 30.38 | 84.21 | 0.632 | 0.267 | 0.817 | 137.9 | -30.5 |
| log-sigma-2-0-mm-3D_gldm_DependenceEntropy | 2.08 | 5.76 | 0.885 | 0.046 | 0.97 | 10.2 | -1.3 |
| log-sigma-2-0-mm-3D_gldm_DependenceNonUniformity | 17.72 | 49.11 | 0.927 | 0.861 | 0.962 | 73.9 | -24.3 |
| log-sigma-2-0-mm-3D_gldm_DependenceNonUniformityNormalized | 16.18 | 44.84 | 0.725 | 0.526 | 0.849 | 40.5 | -49.2 |
| log-sigma-2-0-mm-3D_gldm_DependenceVariance | 36.61 | 101.49 | 0.584 | 0.322 | 0.763 | 117.7 | -85.3 |
| log-sigma-2-0-mm-3D_gldm_GrayLevelNonUniformity | 23.69 | 65.67 | 0.979 | 0.96 | 0.989 | 82.8 | -48.5 |
| log-sigma-2-0-mm-3D_gldm_GrayLevelVariance | 30.02 | 83.21 | 0.64 | 0.269 | 0.823 | 135.1 | -31.3 |
| log-sigma-2-0-mm-3D_gldm_HighGrayLevelEmphasis | 29.18 | 80.89 | 0.487 | 0.185 | 0.703 | 146.9 | -14.9 |
| log-sigma-2-0-mm-3D_gldm_LargeDependenceEmphasis | 29.11 | 80.68 | 0.753 | 0.568 | 0.865 | 85.5 | -75.8 |
| log-sigma-2-0-mm-3D_gldm_LargeDependenceHighGrayLevelEmphasis | 33.88 | 93.90 | 0.426 | 0.117 | 0.66 | 165.3 | -22.5 |
| log-sigma-2-0-mm-3D_gldm_LargeDependenceLowGrayLevelEmphasis | 51.93 | 143.93 | 0.898 | 0.81 | 0.946 | 80.1 | -207.8 |
| log-sigma-2-0-mm-3D_gldm_LowGrayLevelEmphasis | 27.93 | 77.42 | 0.862 | 0.689 | 0.934 | 20.8 | -134.1 |
| log-sigma-2-0-mm-3D_gldm_SmallDependenceEmphasis | 20.02 | 55.49 | 0.881 | 0.78 | 0.937 | 63.1 | -47.8 |
| log-sigma-2-0-mm-3D_gldm_SmallDependenceHighGrayLevelEmphasis | 38.54 | 106.84 | 0.482 | 0.19 | 0.697 | 171.9 | -41.8 |
| log-sigma-2-0-mm-3D_gldm_SmallDependenceLowGrayLevelEmphasis | 29.66 | 82.22 | 0.59 | 0.26 | 0.782 | 48.3 | -116.1 |
| log-sigma-2-0-mm-3D_glrlm_GrayLevelNonUniformity | 20.80 | 57.65 | 0.992 | 0.985 | 0.996 | 73.4 | -41.9 |
| log-sigma-2-0-mm-3D_glrlm_GrayLevelNonUniformityNormalized | 14.30 | 39.65 | 0.889 | 0.786 | 0.943 | 26.3 | -53 |
| log-sigma-2-0-mm-3D_glrlm_GrayLevelVariance | 29.65 | 82.20 | 0.63 | 0.263 | 0.816 | 134.8 | -29.6 |
| log-sigma-2-0-mm-3D_glrlm_HighGrayLevelRunEmphasis | 29.05 | 80.52 | 0.486 | 0.184 | 0.702 | 146.1 | -14.9 |
| log-sigma-2-0-mm-3D_glrlm_LongRunEmphasis | 9.35 | 25.92 | 0.763 | 0.585 | 0.871 | 26.1 | -25.7 |
| log-sigma-2-0-mm-3D_glrlm_LongRunHighGrayLevelEmphasis | 27.74 | 76.88 | 0.474 | 0.168 | 0.694 | 143.6 | -10.2 |
| log-sigma-2-0-mm-3D_glrlm_LongRunLowGrayLevelEmphasis | 31.20 | 86.47 | 0.932 | 0.861 | 0.966 | 28.3 | -144.7 |
| log-sigma-2-0-mm-3D_glrlm_LowGrayLevelRunEmphasis | 26.32 | 72.96 | 0.875 | 0.692 | 0.943 | 17.1 | -128.9 |
| log-sigma-2-0-mm-3D_glrlm_RunEntropy | 3.28 | 9.10 | 0.874 | 0.106 | 0.964 | 14.9 | -3.4 |
| log-sigma-2-0-mm-3D_glrlm_RunLengthNonUniformity | 15.98 | 44.28 | 0.953 | 0.909 | 0.976 | 73.6 | -14.9 |
| log-sigma-2-0-mm-3D_glrlm_RunLengthNonUniformityNormalized | 4.81 | 13.33 | 0.825 | 0.685 | 0.907 | 13.6 | -13 |
| log-sigma-2-0-mm-3D_glrlm_RunPercentage | 3.02 | 8.38 | 0.803 | 0.649 | 0.894 | 8.4 | -8.3 |
| log-sigma-2-0-mm-3D_glrlm_RunVariance | 27.41 | 75.98 | 0.717 | 0.514 | 0.845 | 82.5 | -69.4 |
| log-sigma-2-0-mm-3D_glrlm_ShortRunEmphasis | 2.29 | 6.33 | 0.821 | 0.678 | 0.904 | 6.4 | -6.2 |
| log-sigma-2-0-mm-3D_glrlm_ShortRunHighGrayLevelEmphasis | 29.71 | 82.36 | 0.488 | 0.187 | 0.704 | 147.7 | -17 |
| log-sigma-2-0-mm-3D_glrlm_ShortRunLowGrayLevelEmphasis | 25.02 | 69.34 | 0.848 | 0.604 | 0.933 | 14.1 | -124.6 |
| log-sigma-2-0-mm-3D_glszm_GrayLevelNonUniformity | 17.44 | 48.34 | 0.858 | 0.741 | 0.925 | 58.8 | -37.8 |
| log-sigma-2-0-mm-3D_glszm_GrayLevelNonUniformityNormalized | 13.81 | 38.27 | 0.819 | 0.61 | 0.912 | 10.7 | -65.8 |
| log-sigma-2-0-mm-3D_glszm_GrayLevelVariance | 29.57 | 81.95 | 0.556 | 0.215 | 0.762 | 133.7 | -30.2 |
| log-sigma-2-0-mm-3D_glszm_HighGrayLevelZoneEmphasis | 30.27 | 83.90 | 0.48 | 0.181 | 0.697 | 141.8 | -26 |
| log-sigma-2-0-mm-3D_glszm_LargeAreaEmphasis | 59.82 | 165.82 | 0.354 | 0.036 | 0.607 | 171.7 | -159.9 |
| log-sigma-2-0-mm-3D_glszm_LargeAreaHighGrayLevelEmphasis | 58.71 | 162.72 | 0.701 | 0.489 | 0.835 | 225.2 | -100.2 |
| log-sigma-2-0-mm-3D_glszm_LargeAreaLowGrayLevelEmphasis | 56.83 | 157.53 | 0.855 | 0.728 | 0.924 | 95.9 | -219.2 |
| log-sigma-2-0-mm-3D_glszm_LowGrayLevelZoneEmphasis | 23.11 | 64.05 | 0.933 | 0.805 | 0.972 | 17.1 | -111 |
| log-sigma-2-0-mm-3D_glszm_SizeZoneNonUniformity | 32.75 | 90.77 | 0.683 | 0.437 | 0.83 | 134.7 | -46.9 |
| log-sigma-2-0-mm-3D_glszm_SizeZoneNonUniformityNormalized | 17.66 | 48.96 | 0.724 | 0.513 | 0.851 | 56.6 | -41.3 |
| log-sigma-2-0-mm-3D_glszm_SmallAreaEmphasis | 11.44 | 31.72 | 0.718 | 0.505 | 0.847 | 36.4 | -27 |
| log-sigma-2-0-mm-3D_glszm_SmallAreaHighGrayLevelEmphasis | 36.50 | 101.18 | 0.435 | 0.137 | 0.663 | 159.7 | -42.7 |
| log-sigma-2-0-mm-3D_glszm_SmallAreaLowGrayLevelEmphasis | 45.04 | 124.85 | 0.294 | -0.032 | 0.564 | 97.8 | -151.9 |
| log-sigma-2-0-mm-3D_glszm_ZoneEntropy | 8.13 | 22.54 | 0.877 | 0.301 | 0.961 | 30.7 | -14.3 |
| log-sigma-2-0-mm-3D_glszm_ZonePercentage | 23.57 | 65.34 | 0.879 | 0.778 | 0.936 | 74 | -56.7 |
| log-sigma-2-0-mm-3D_glszm_ZoneVariance | 65.52 | 181.61 | 0.353 | 0.035 | 0.607 | 193.2 | -170 |
| log-sigma-2-0-mm-3D_ngtdm_Busyness | 26.13 | 72.43 | 0.765 | 0.552 | 0.879 | 20.6 | -124.3 |
| log-sigma-2-0-mm-3D_ngtdm_Coarseness | 14.05 | 38.94 | 0.847 | 0.564 | 0.936 | 18.7 | -59.1 |
| log-sigma-2-0-mm-3D_ngtdm_Complexity | 34.71 | 96.21 | 0.556 | 0.262 | 0.751 | 163.4 | -29 |
| log-sigma-2-0-mm-3D_ngtdm_Contrast | 26.59 | 73.72 | 0.871 | 0.763 | 0.932 | 73.2 | -74.3 |
| log-sigma-2-0-mm-3D_ngtdm_Strength | 27.76 | 76.95 | 0.599 | 0.284 | 0.786 | 135.9 | -18 |
| log-sigma-3-0-mm-3D_firstorder_10Percentile | 17.23 | 47.76 | 0.791 | 0.4 | 0.913 | 66.4 | -29.1 |
| log-sigma-3-0-mm-3D_firstorder_90Percentile | 141.58 | 392.45 | 0.654 | 0.119 | 0.854 | 398.6 | -386.3 |
| log-sigma-3-0-mm-3D_firstorder_Energy | 31.86 | 88.31 | 0.604 | 0.334 | 0.779 | 150.9 | -25.7 |
| log-sigma-3-0-mm-3D_firstorder_Entropy | 5.11 | 14.17 | 0.872 | 0.257 | 0.96 | 20.9 | -7.5 |
| log-sigma-3-0-mm-3D_firstorder_InterquartileRange | 15.87 | 43.98 | 0.812 | 0.157 | 0.938 | 67.4 | -20.6 |
| log-sigma-3-0-mm-3D_firstorder_Kurtosis | 10.78 | 29.88 | 0.737 | 0.359 | 0.882 | 45.4 | -14.4 |
| log-sigma-3-0-mm-3D_firstorder_Maximum | 393.93 | 1091.91 | 0.69 | 0.147 | 0.874 | 1241.9 | -941.9 |
| log-sigma-3-0-mm-3D_firstorder_MeanAbsoluteDeviation | 15.57 | 43.16 | 0.778 | 0.131 | 0.923 | 71.4 | -14.9 |
| log-sigma-3-0-mm-3D_firstorder_Mean | 30.85 | 85.50 | 0.626 | 0.381 | 0.789 | 82.6 | -88.4 |
| log-sigma-3-0-mm-3D_firstorder_Median | 658.12 | 1824.20 | 0.443 | 0.116 | 0.678 | 1880.1 | -1768.3 |
| log-sigma-3-0-mm-3D_firstorder_Minimum | 17.30 | 47.96 | 0.713 | 0.242 | 0.879 | 79.2 | -16.7 |
| log-sigma-3-0-mm-3D_firstorder_Range | 16.63 | 46.10 | 0.712 | 0.179 | 0.884 | 79.9 | -12.3 |
| log-sigma-3-0-mm-3D_firstorder_RobustMeanAbsoluteDeviation | 15.29 | 42.37 | 0.809 | 0.134 | 0.938 | 67.5 | -17.2 |
| log-sigma-3-0-mm-3D_firstorder_RootMeanSquared | 15.69 | 43.50 | 0.779 | 0.332 | 0.911 | 62.2 | -24.8 |
| log-sigma-3-0-mm-3D_firstorder_Skewness | 23.05 | 63.90 | 0.791 | 0.079 | 0.932 | 90.3 | -37.5 |
| log-sigma-3-0-mm-3D_firstorder_TotalEnergy | 31.86 | 88.31 | 0.604 | 0.334 | 0.779 | 150.9 | -25.7 |
| log-sigma-3-0-mm-3D_firstorder_Uniformity | 13.40 | 37.15 | 0.893 | 0.771 | 0.948 | 19.9 | -54.4 |
| log-sigma-3-0-mm-3D_firstorder_Variance | 30.20 | 83.71 | 0.598 | 0.24 | 0.793 | 141.3 | -26.1 |
| log-sigma-3-0-mm-3D_glcm_Autocorrelation | 30.93 | 85.74 | 0.458 | 0.155 | 0.682 | 158.4 | -13 |
| log-sigma-3-0-mm-3D_glcm_ClusterProminence | 53.04 | 147.03 | 0.184 | -0.126 | 0.471 | 260.5 | -33.6 |
| log-sigma-3-0-mm-3D_glcm_ClusterShade | 49.82 | 138.09 | 0.303 | 0 | 0.563 | 243.8 | -32.4 |
| log-sigma-3-0-mm-3D_glcm_ClusterTendency | 30.65 | 84.95 | 0.577 | 0.229 | 0.778 | 147 | -22.8 |
| log-sigma-3-0-mm-3D_glcm_Contrast | 29.35 | 81.35 | 0.703 | 0.326 | 0.863 | 125.5 | -37.2 |
| log-sigma-3-0-mm-3D_glcm_Correlation | 5.69 | 15.78 | 0.775 | 0.226 | 0.916 | 22.7 | -8.9 |
| log-sigma-3-0-mm-3D_glcm_DifferenceAverage | 16.09 | 44.60 | 0.841 | 0.307 | 0.945 | 64.6 | -24.6 |
| log-sigma-3-0-mm-3D_glcm_DifferenceEntropy | 7.15 | 19.80 | 0.861 | 0.294 | 0.954 | 28.6 | -11 |
| log-sigma-3-0-mm-3D_glcm_DifferenceVariance | 28.98 | 80.34 | 0.598 | 0.268 | 0.788 | 131.5 | -29.2 |
| log-sigma-3-0-mm-3D_glcm_Id | 9.41 | 26.09 | 0.846 | 0.685 | 0.923 | 18.9 | -33.2 |
| log-sigma-3-0-mm-3D_glcm_Idm | 14.06 | 38.98 | 0.831 | 0.683 | 0.912 | 30.3 | -47.6 |
| log-sigma-3-0-mm-3D_glcm_Idmn | 0.38 | 1.05 | 0.809 | 0.459 | 0.92 | 1.4 | -0.7 |
| log-sigma-3-0-mm-3D_glcm_Idn | 1.00 | 2.76 | 0.806 | 0.379 | 0.924 | 3.9 | -1.6 |
| log-sigma-3-0-mm-3D_glcm_Imc1 | 8.65 | 23.98 | 0.86 | 0.706 | 0.931 | 30.1 | -17.9 |
| log-sigma-3-0-mm-3D_glcm_Imc2 | 2.30 | 6.37 | 0.904 | 0.742 | 0.958 | 8.2 | -4.6 |
| log-sigma-3-0-mm-3D_glcm_InverseVariance | 13.17 | 36.51 | 0.842 | 0.681 | 0.92 | 27.4 | -45.7 |
| log-sigma-3-0-mm-3D_glcm_JointAverage | 16.80 | 46.56 | 0.691 | 0.218 | 0.867 | 85.4 | -7.7 |
| log-sigma-3-0-mm-3D_glcm_JointEnergy | 24.13 | 66.87 | 0.864 | 0.752 | 0.928 | 38 | -95.8 |
| log-sigma-3-0-mm-3D_glcm_JointEntropy | 4.88 | 13.52 | 0.875 | 0.247 | 0.962 | 19.9 | -7.1 |
| log-sigma-3-0-mm-3D_glcm_MCC | 4.31 | 11.94 | 0.768 | 0.289 | 0.908 | 16.9 | -7 |
| log-sigma-3-0-mm-3D_glcm_MaximumProbability | 26.36 | 73.07 | 0.777 | 0.607 | 0.88 | 63.2 | -83 |
| log-sigma-3-0-mm-3D_glcm_SumAverage | 16.80 | 46.56 | 0.691 | 0.218 | 0.867 | 85.4 | -7.7 |
| log-sigma-3-0-mm-3D_glcm_SumEntropy | 4.28 | 11.88 | 0.864 | 0.104 | 0.961 | 18.7 | -5 |
| log-sigma-3-0-mm-3D_glcm_SumSquares | 30.32 | 84.06 | 0.588 | 0.237 | 0.786 | 143.7 | -24.4 |
| log-sigma-3-0-mm-3D_gldm_DependenceEntropy | 1.98 | 5.49 | 0.886 | 0.063 | 0.97 | 9.9 | -1.1 |
| log-sigma-3-0-mm-3D_gldm_DependenceNonUniformity | 16.59 | 45.99 | 0.919 | 0.845 | 0.958 | 77.3 | -14.7 |
| log-sigma-3-0-mm-3D_gldm_DependenceNonUniformityNormalized | 13.03 | 36.13 | 0.838 | 0.706 | 0.914 | 38.5 | -33.8 |
| log-sigma-3-0-mm-3D_gldm_DependenceVariance | 30.43 | 84.35 | 0.64 | 0.401 | 0.798 | 90 | -78.7 |
| log-sigma-3-0-mm-3D_gldm_GrayLevelNonUniformity | 20.60 | 57.09 | 0.973 | 0.949 | 0.986 | 69 | -45.2 |
| log-sigma-3-0-mm-3D_gldm_GrayLevelVariance | 29.95 | 83.02 | 0.598 | 0.24 | 0.793 | 140.8 | -25.3 |
| log-sigma-3-0-mm-3D_gldm_HighGrayLevelEmphasis | 30.77 | 85.30 | 0.472 | 0.167 | 0.693 | 155.5 | -15.1 |
| log-sigma-3-0-mm-3D_gldm_LargeDependenceEmphasis | 24.39 | 67.61 | 0.773 | 0.601 | 0.877 | 63.9 | -71.3 |
| log-sigma-3-0-mm-3D_gldm_LargeDependenceHighGrayLevelEmphasis | 31.46 | 87.19 | 0.348 | 0.046 | 0.597 | 156.5 | -17.9 |
| log-sigma-3-0-mm-3D_gldm_LargeDependenceLowGrayLevelEmphasis | 58.06 | 160.93 | 0.918 | 0.846 | 0.957 | 112.3 | -209.5 |
| log-sigma-3-0-mm-3D_gldm_LowGrayLevelEmphasis | 29.07 | 80.57 | 0.893 | 0.734 | 0.951 | 34.8 | -126.4 |
| log-sigma-3-0-mm-3D_gldm_SmallDependenceEmphasis | 17.48 | 48.44 | 0.873 | 0.74 | 0.937 | 58.6 | -38.3 |
| log-sigma-3-0-mm-3D_gldm_SmallDependenceHighGrayLevelEmphasis | 37.17 | 103.03 | 0.497 | 0.198 | 0.71 | 176.4 | -29.6 |
| log-sigma-3-0-mm-3D_gldm_SmallDependenceLowGrayLevelEmphasis | 31.64 | 87.69 | 0.502 | 0.195 | 0.716 | 44.4 | -131 |
| log-sigma-3-0-mm-3D_glrlm_GrayLevelNonUniformity | 18.96 | 52.55 | 0.985 | 0.972 | 0.992 | 64.1 | -41 |
| log-sigma-3-0-mm-3D_glrlm_GrayLevelNonUniformityNormalized | 13.00 | 36.04 | 0.897 | 0.768 | 0.951 | 18 | -54.1 |
| log-sigma-3-0-mm-3D_glrlm_GrayLevelVariance | 29.80 | 82.59 | 0.592 | 0.237 | 0.789 | 140.5 | -24.7 |
| log-sigma-3-0-mm-3D_glrlm_HighGrayLevelRunEmphasis | 30.72 | 85.14 | 0.472 | 0.168 | 0.693 | 155 | -15.3 |
| log-sigma-3-0-mm-3D_glrlm_LongRunEmphasis | 5.96 | 16.53 | 0.78 | 0.612 | 0.881 | 15.3 | -17.8 |
| log-sigma-3-0-mm-3D_glrlm_LongRunHighGrayLevelEmphasis | 29.01 | 80.41 | 0.457 | 0.151 | 0.682 | 150.1 | -10.7 |
| log-sigma-3-0-mm-3D_glrlm_LongRunLowGrayLevelEmphasis | 32.77 | 90.84 | 0.92 | 0.826 | 0.961 | 44.1 | -137.6 |
| log-sigma-3-0-mm-3D_glrlm_LowGrayLevelRunEmphasis | 27.77 | 76.98 | 0.895 | 0.727 | 0.954 | 31 | -123 |
| log-sigma-3-0-mm-3D_glrlm_RunEntropy | 3.36 | 9.33 | 0.869 | 0.088 | 0.963 | 15.4 | -3.3 |
| log-sigma-3-0-mm-3D_glrlm_RunLengthNonUniformity | 15.60 | 43.24 | 0.964 | 0.929 | 0.981 | 73.5 | -12.9 |
| log-sigma-3-0-mm-3D_glrlm_RunLengthNonUniformityNormalized | 3.03 | 8.40 | 0.837 | 0.705 | 0.913 | 9.3 | -7.5 |
| log-sigma-3-0-mm-3D_glrlm_RunPercentage | 1.84 | 5.11 | 0.82 | 0.676 | 0.903 | 5.6 | -4.6 |
| log-sigma-3-0-mm-3D_glrlm_RunVariance | 23.54 | 65.26 | 0.741 | 0.551 | 0.859 | 64.5 | -66 |
| log-sigma-3-0-mm-3D_glrlm_ShortRunEmphasis | 1.33 | 3.69 | 0.832 | 0.696 | 0.91 | 4.1 | -3.3 |
| log-sigma-3-0-mm-3D_glrlm_ShortRunHighGrayLevelEmphasis | 31.20 | 86.49 | 0.477 | 0.172 | 0.696 | 156.4 | -16.6 |
| log-sigma-3-0-mm-3D_glrlm_ShortRunLowGrayLevelEmphasis | 26.45 | 73.32 | 0.885 | 0.679 | 0.951 | 27.4 | -119.2 |
| log-sigma-3-0-mm-3D_glszm_GrayLevelNonUniformity | 14.51 | 40.21 | 0.939 | 0.883 | 0.968 | 55 | -25.4 |
| log-sigma-3-0-mm-3D_glszm_GrayLevelNonUniformityNormalized | 13.18 | 36.54 | 0.915 | 0.774 | 0.962 | 10 | -63.1 |
| log-sigma-3-0-mm-3D_glszm_GrayLevelVariance | 28.19 | 78.15 | 0.549 | 0.215 | 0.755 | 135 | -21.3 |
| log-sigma-3-0-mm-3D_glszm_HighGrayLevelZoneEmphasis | 29.85 | 82.73 | 0.477 | 0.173 | 0.696 | 147.9 | -17.5 |
| log-sigma-3-0-mm-3D_glszm_LargeAreaEmphasis | 56.19 | 155.75 | 0.272 | -0.053 | 0.546 | 140.4 | -171.1 |
| log-sigma-3-0-mm-3D_glszm_LargeAreaHighGrayLevelEmphasis | 57.18 | 158.49 | 0.645 | 0.408 | 0.801 | 207.6 | -109.4 |
| log-sigma-3-0-mm-3D_glszm_LargeAreaLowGrayLevelEmphasis | 60.74 | 168.36 | 0.843 | 0.716 | 0.917 | 106.4 | -230.3 |
| log-sigma-3-0-mm-3D_glszm_LowGrayLevelZoneEmphasis | 23.72 | 65.75 | 0.891 | 0.736 | 0.95 | 15.4 | -116.1 |
| log-sigma-3-0-mm-3D_glszm_SizeZoneNonUniformity | 25.17 | 69.78 | 0.738 | 0.536 | 0.859 | 118.7 | -20.9 |
| log-sigma-3-0-mm-3D_glszm_SizeZoneNonUniformityNormalized | 13.43 | 37.24 | 0.821 | 0.579 | 0.917 | 46.6 | -27.9 |
| log-sigma-3-0-mm-3D_glszm_SmallAreaEmphasis | 8.20 | 22.72 | 0.804 | 0.609 | 0.902 | 27.4 | -18 |
| log-sigma-3-0-mm-3D_glszm_SmallAreaHighGrayLevelEmphasis | 31.37 | 86.96 | 0.464 | 0.162 | 0.686 | 153.3 | -20.6 |
| log-sigma-3-0-mm-3D_glszm_SmallAreaLowGrayLevelEmphasis | 44.75 | 124.03 | 0.26 | -0.069 | 0.538 | 76.5 | -171.6 |
| log-sigma-3-0-mm-3D_glszm_ZoneEntropy | 4.50 | 12.47 | 0.866 | 0.265 | 0.957 | 18.3 | -6.7 |
| log-sigma-3-0-mm-3D_glszm_ZonePercentage | 20.77 | 57.58 | 0.866 | 0.737 | 0.932 | 69.4 | -45.8 |
| log-sigma-3-0-mm-3D_glszm_ZoneVariance | 64.89 | 179.87 | 0.255 | -0.072 | 0.534 | 171.2 | -188.6 |
| log-sigma-3-0-mm-3D_ngtdm_Busyness | 28.15 | 78.03 | 0.757 | 0.555 | 0.872 | 23.9 | -132.1 |
| log-sigma-3-0-mm-3D_ngtdm_Coarseness | 13.26 | 36.75 | 0.851 | 0.505 | 0.941 | 17 | -56.5 |
| log-sigma-3-0-mm-3D_ngtdm_Complexity | 38.85 | 107.69 | 0.543 | 0.242 | 0.743 | 178.1 | -37.2 |
| log-sigma-3-0-mm-3D_ngtdm_Contrast | 21.72 | 60.21 | 0.876 | 0.762 | 0.936 | 67.7 | -52.7 |
| log-sigma-3-0-mm-3D_ngtdm_Strength | 30.50 | 84.53 | 0.555 | 0.233 | 0.757 | 144 | -25.1 |
| log-sigma-4-0-mm-3D_firstorder_10Percentile | 17.43 | 48.32 | 0.768 | 0.332 | 0.905 | 69.2 | -27.4 |
| log-sigma-4-0-mm-3D_firstorder_90Percentile | 637.09 | 1765.93 | 0.568 | 0.143 | 0.787 | 1647.7 | -1884.2 |
| log-sigma-4-0-mm-3D_firstorder_Energy | 32.80 | 90.93 | 0.608 | 0.34 | 0.782 | 154.4 | -27.4 |
| log-sigma-4-0-mm-3D_firstorder_Entropy | 4.86 | 13.47 | 0.86 | 0.105 | 0.959 | 21.1 | -5.8 |
| log-sigma-4-0-mm-3D_firstorder_InterquartileRange | 15.32 | 42.46 | 0.79 | 0.108 | 0.93 | 69.6 | -15.3 |
| log-sigma-4-0-mm-3D_firstorder_Kurtosis | 8.85 | 24.53 | 0.762 | 0.413 | 0.893 | 37.8 | -11.2 |
| log-sigma-4-0-mm-3D_firstorder_Maximum | 1320.16 | 3659.31 | 0.647 | 0.172 | 0.843 | 4066.9 | -3251.7 |
| log-sigma-4-0-mm-3D_firstorder_MeanAbsoluteDeviation | 15.86 | 43.96 | 0.755 | 0.129 | 0.912 | 75 | -12.9 |
| log-sigma-4-0-mm-3D_firstorder_Mean | 28.46 | 78.88 | 0.646 | 0.41 | 0.802 | 81.5 | -76.3 |
| log-sigma-4-0-mm-3D_firstorder_Median | 46.83 | 129.80 | 0.44 | 0.146 | 0.666 | 106.9 | -152.7 |
| log-sigma-4-0-mm-3D_firstorder_Minimum | 17.67 | 48.98 | 0.694 | 0.225 | 0.868 | 81.2 | -16.8 |
| log-sigma-4-0-mm-3D_firstorder_Range | 17.05 | 47.26 | 0.698 | 0.158 | 0.877 | 83.5 | -11 |
| log-sigma-4-0-mm-3D_firstorder_RobustMeanAbsoluteDeviation | 15.50 | 42.96 | 0.779 | 0.107 | 0.925 | 71.7 | -14.3 |
| log-sigma-4-0-mm-3D_firstorder_RootMeanSquared | 15.75 | 43.67 | 0.765 | 0.313 | 0.904 | 62.9 | -24.4 |
| log-sigma-4-0-mm-3D_firstorder_Skewness | 29.21 | 80.96 | 0.802 | 0.176 | 0.932 | 109.2 | -52.7 |
| log-sigma-4-0-mm-3D_firstorder_TotalEnergy | 32.80 | 90.93 | 0.608 | 0.34 | 0.782 | 154.4 | -27.4 |
| log-sigma-4-0-mm-3D_firstorder_Uniformity | 13.30 | 36.87 | 0.891 | 0.709 | 0.952 | 14.1 | -59.7 |
| log-sigma-4-0-mm-3D_firstorder_Variance | 30.55 | 84.68 | 0.554 | 0.212 | 0.761 | 146.8 | -22.5 |
| log-sigma-4-0-mm-3D_glcm_Autocorrelation | 33.69 | 93.38 | 0.41 | 0.106 | 0.647 | 168.8 | -17.9 |
| log-sigma-4-0-mm-3D_glcm_ClusterProminence | 53.71 | 148.88 | 0.154 | -0.155 | 0.446 | 265.6 | -32.2 |
| log-sigma-4-0-mm-3D_glcm_ClusterShade | 60.90 | 168.80 | 0.263 | -0.041 | 0.532 | 276.4 | -61.2 |
| log-sigma-4-0-mm-3D_glcm_ClusterTendency | 31.50 | 87.32 | 0.533 | 0.2 | 0.744 | 153.4 | -21.3 |
| log-sigma-4-0-mm-3D_glcm_Contrast | 28.98 | 80.32 | 0.669 | 0.291 | 0.842 | 128.8 | -31.8 |
| log-sigma-4-0-mm-3D_glcm_Correlation | 5.40 | 14.96 | 0.802 | 0.274 | 0.927 | 21.4 | -8.5 |
| log-sigma-4-0-mm-3D_glcm_DifferenceAverage | 15.35 | 42.55 | 0.816 | 0.205 | 0.937 | 65.7 | -19.4 |
| log-sigma-4-0-mm-3D_glcm_DifferenceEntropy | 6.68 | 18.51 | 0.85 | 0.151 | 0.954 | 28.2 | -8.8 |
| log-sigma-4-0-mm-3D_glcm_DifferenceVariance | 29.00 | 80.40 | 0.573 | 0.246 | 0.771 | 134 | -26.8 |
| log-sigma-4-0-mm-3D_glcm_Id | 8.90 | 24.68 | 0.855 | 0.6 | 0.938 | 14.2 | -35.1 |
| log-sigma-4-0-mm-3D_glcm_Idm | 13.16 | 36.47 | 0.847 | 0.652 | 0.928 | 22.7 | -50.3 |
| log-sigma-4-0-mm-3D_glcm_Idmn | 0.32 | 0.89 | 0.833 | 0.37 | 0.938 | 1.3 | -0.5 |
| log-sigma-4-0-mm-3D_glcm_Idn | 0.84 | 2.33 | 0.836 | 0.304 | 0.942 | 3.5 | -1.2 |
| log-sigma-4-0-mm-3D_glcm_Imc1 | 8.68 | 24.05 | 0.839 | 0.653 | 0.922 | 30.3 | -17.8 |
| log-sigma-4-0-mm-3D_glcm_Imc2 | 1.86 | 5.16 | 0.908 | 0.724 | 0.962 | 6.8 | -3.5 |
| log-sigma-4-0-mm-3D_glcm_InverseVariance | 12.70 | 35.21 | 0.847 | 0.556 | 0.936 | 20 | -50.4 |
| log-sigma-4-0-mm-3D_glcm_JointAverage | 18.39 | 50.99 | 0.664 | 0.205 | 0.85 | 91.6 | -10.3 |
| log-sigma-4-0-mm-3D_glcm_JointEnergy | 22.68 | 62.86 | 0.852 | 0.727 | 0.922 | 25.3 | -100.4 |
| log-sigma-4-0-mm-3D_glcm_JointEntropy | 4.63 | 12.84 | 0.866 | 0.11 | 0.962 | 20 | -5.7 |
| log-sigma-4-0-mm-3D_glcm_MCC | 4.42 | 12.26 | 0.78 | 0.314 | 0.913 | 17.2 | -7.3 |
| log-sigma-4-0-mm-3D_glcm_MaximumProbability | 25.84 | 71.62 | 0.796 | 0.637 | 0.89 | 50.7 | -92.6 |
| log-sigma-4-0-mm-3D_glcm_SumAverage | 18.39 | 50.99 | 0.664 | 0.205 | 0.85 | 91.6 | -10.3 |
| log-sigma-4-0-mm-3D_glcm_SumEntropy | 4.18 | 11.60 | 0.851 | 0.031 | 0.959 | 19.2 | -4 |
| log-sigma-4-0-mm-3D_glcm_SumSquares | 30.96 | 85.80 | 0.544 | 0.208 | 0.753 | 149.7 | -21.9 |
| log-sigma-4-0-mm-3D_gldm_DependenceEntropy | 2.16 | 5.99 | 0.874 | 0.019 | 0.967 | 11 | -1 |
| log-sigma-4-0-mm-3D_gldm_DependenceNonUniformity | 17.34 | 48.06 | 0.909 | 0.826 | 0.953 | 81.9 | -14.2 |
| log-sigma-4-0-mm-3D_gldm_DependenceNonUniformityNormalized | 10.95 | 30.35 | 0.871 | 0.753 | 0.933 | 35.4 | -25.3 |
| log-sigma-4-0-mm-3D_gldm_DependenceVariance | 27.74 | 76.89 | 0.647 | 0.41 | 0.802 | 75.2 | -78.5 |
| log-sigma-4-0-mm-3D_gldm_GrayLevelNonUniformity | 18.25 | 50.59 | 0.968 | 0.939 | 0.984 | 56.9 | -44.2 |
| log-sigma-4-0-mm-3D_gldm_GrayLevelVariance | 30.57 | 84.74 | 0.554 | 0.212 | 0.761 | 146.9 | -22.6 |
| log-sigma-4-0-mm-3D_gldm_HighGrayLevelEmphasis | 33.25 | 92.15 | 0.425 | 0.119 | 0.658 | 165.2 | -19.1 |
| log-sigma-4-0-mm-3D_gldm_LargeDependenceEmphasis | 21.75 | 60.29 | 0.772 | 0.598 | 0.876 | 51.3 | -69.3 |
| log-sigma-4-0-mm-3D_gldm_LargeDependenceHighGrayLevelEmphasis | 30.55 | 84.67 | 0.329 | 0.028 | 0.583 | 153.7 | -15.6 |
| log-sigma-4-0-mm-3D_gldm_LargeDependenceLowGrayLevelEmphasis | 61.57 | 170.67 | 0.855 | 0.736 | 0.923 | 91.8 | -249.5 |
| log-sigma-4-0-mm-3D_gldm_LowGrayLevelEmphasis | 32.25 | 89.40 | 0.845 | 0.647 | 0.927 | 29.6 | -149.2 |
| log-sigma-4-0-mm-3D_gldm_SmallDependenceEmphasis | 15.81 | 43.82 | 0.879 | 0.679 | 0.947 | 55.7 | -32 |
| log-sigma-4-0-mm-3D_gldm_SmallDependenceHighGrayLevelEmphasis | 39.95 | 110.74 | 0.449 | 0.146 | 0.675 | 189 | -32.4 |
| log-sigma-4-0-mm-3D_gldm_SmallDependenceLowGrayLevelEmphasis | 29.33 | 81.30 | 0.666 | 0.406 | 0.82 | 48.2 | -114.4 |
| log-sigma-4-0-mm-3D_glrlm_GrayLevelNonUniformity | 17.20 | 47.69 | 0.98 | 0.962 | 0.99 | 54.2 | -41.2 |
| log-sigma-4-0-mm-3D_glrlm_GrayLevelNonUniformityNormalized | 13.18 | 36.53 | 0.893 | 0.7 | 0.954 | 13.2 | -59.8 |
| log-sigma-4-0-mm-3D_glrlm_GrayLevelVariance | 30.62 | 84.88 | 0.55 | 0.209 | 0.758 | 147.1 | -22.6 |
| log-sigma-4-0-mm-3D_glrlm_HighGrayLevelRunEmphasis | 33.18 | 91.96 | 0.426 | 0.12 | 0.659 | 164.7 | -19.2 |
| log-sigma-4-0-mm-3D_glrlm_LongRunEmphasis | 5.06 | 14.03 | 0.783 | 0.616 | 0.883 | 12.1 | -15.9 |
| log-sigma-4-0-mm-3D_glrlm_LongRunHighGrayLevelEmphasis | 31.29 | 86.72 | 0.415 | 0.109 | 0.65 | 158.7 | -14.7 |
| log-sigma-4-0-mm-3D_glrlm_LongRunLowGrayLevelEmphasis | 36.37 | 100.83 | 0.862 | 0.727 | 0.93 | 38.4 | -163.3 |
| log-sigma-4-0-mm-3D_glrlm_LowGrayLevelRunEmphasis | 30.90 | 85.66 | 0.849 | 0.645 | 0.93 | 26.5 | -144.9 |
| log-sigma-4-0-mm-3D_glrlm_RunEntropy | 3.50 | 9.70 | 0.854 | 0.026 | 0.96 | 16.7 | -2.7 |
| log-sigma-4-0-mm-3D_glrlm_RunLengthNonUniformity | 16.18 | 44.84 | 0.966 | 0.932 | 0.983 | 75.4 | -14.3 |
| log-sigma-4-0-mm-3D_glrlm_RunLengthNonUniformityNormalized | 2.44 | 6.75 | 0.86 | 0.733 | 0.927 | 7.9 | -5.6 |
| log-sigma-4-0-mm-3D_glrlm_RunPercentage | 1.52 | 4.20 | 0.837 | 0.701 | 0.914 | 4.8 | -3.6 |
| log-sigma-4-0-mm-3D_glrlm_RunVariance | 22.00 | 60.97 | 0.739 | 0.548 | 0.857 | 52.4 | -69.6 |
| log-sigma-4-0-mm-3D_glrlm_ShortRunEmphasis | 1.06 | 2.95 | 0.851 | 0.723 | 0.922 | 3.4 | -2.5 |
| log-sigma-4-0-mm-3D_glrlm_ShortRunHighGrayLevelEmphasis | 33.64 | 93.25 | 0.428 | 0.123 | 0.661 | 166.2 | -20.3 |
| log-sigma-4-0-mm-3D_glrlm_ShortRunLowGrayLevelEmphasis | 29.39 | 81.47 | 0.846 | 0.603 | 0.932 | 23.2 | -139.7 |
| log-sigma-4-0-mm-3D_glszm_GrayLevelNonUniformity | 13.70 | 37.97 | 0.949 | 0.903 | 0.974 | 51.7 | -24.3 |
| log-sigma-4-0-mm-3D_glszm_GrayLevelNonUniformityNormalized | 14.66 | 40.63 | 0.912 | 0.729 | 0.964 | 12.6 | -68.7 |
| log-sigma-4-0-mm-3D_glszm_GrayLevelVariance | 29.36 | 81.39 | 0.512 | 0.182 | 0.729 | 144.6 | -18.2 |
| log-sigma-4-0-mm-3D_glszm_HighGrayLevelZoneEmphasis | 32.15 | 89.12 | 0.436 | 0.129 | 0.666 | 158.2 | -20.1 |
| log-sigma-4-0-mm-3D_glszm_LargeAreaEmphasis | 46.48 | 128.83 | 0.405 | 0.097 | 0.643 | 106.2 | -151.5 |
| log-sigma-4-0-mm-3D_glszm_LargeAreaHighGrayLevelEmphasis | 47.76 | 132.39 | 0.624 | 0.378 | 0.788 | 182.6 | -82.2 |
| log-sigma-4-0-mm-3D_glszm_LargeAreaLowGrayLevelEmphasis | 57.82 | 160.26 | 0.85 | 0.727 | 0.92 | 72 | -248.5 |
| log-sigma-4-0-mm-3D_glszm_LowGrayLevelZoneEmphasis | 24.70 | 68.47 | 0.883 | 0.75 | 0.943 | 16.6 | -120.3 |
| log-sigma-4-0-mm-3D_glszm_SizeZoneNonUniformity | 27.19 | 75.37 | 0.744 | 0.546 | 0.862 | 126.4 | -24.4 |
| log-sigma-4-0-mm-3D_glszm_SizeZoneNonUniformityNormalized | 11.00 | 30.49 | 0.841 | 0.473 | 0.937 | 42 | -19 |
| log-sigma-4-0-mm-3D_glszm_SmallAreaEmphasis | 6.43 | 17.83 | 0.825 | 0.492 | 0.927 | 24.1 | -11.6 |
| log-sigma-4-0-mm-3D_glszm_SmallAreaHighGrayLevelEmphasis | 34.25 | 94.94 | 0.432 | 0.127 | 0.663 | 167.2 | -22.7 |
| log-sigma-4-0-mm-3D_glszm_SmallAreaLowGrayLevelEmphasis | 41.71 | 115.62 | 0.274 | -0.054 | 0.549 | 83.8 | -147.4 |
| log-sigma-4-0-mm-3D_glszm_ZoneEntropy | 3.40 | 9.43 | 0.895 | 0.188 | 0.97 | 14.9 | -4 |
| log-sigma-4-0-mm-3D_glszm_ZonePercentage | 17.18 | 47.61 | 0.88 | 0.698 | 0.946 | 60.1 | -35.1 |
| log-sigma-4-0-mm-3D_glszm_ZoneVariance | 55.03 | 152.54 | 0.337 | 0.019 | 0.595 | 133.2 | -171.9 |
| log-sigma-4-0-mm-3D_ngtdm_Busyness | 29.59 | 82.01 | 0.75 | 0.53 | 0.87 | 21.1 | -142.9 |
| log-sigma-4-0-mm-3D_ngtdm_Coarseness | 12.94 | 35.87 | 0.859 | 0.521 | 0.945 | 18 | -53.8 |
| log-sigma-4-0-mm-3D_ngtdm_Complexity | 39.73 | 110.14 | 0.497 | 0.191 | 0.711 | 188.5 | -31.8 |
| log-sigma-4-0-mm-3D_ngtdm_Contrast | 19.26 | 53.38 | 0.852 | 0.715 | 0.924 | 61.6 | -45.2 |
| log-sigma-4-0-mm-3D_ngtdm_Strength | 31.02 | 85.97 | 0.486 | 0.164 | 0.708 | 149.9 | -22.1 |
| log-sigma-5-0-mm-3D_firstorder_10Percentile | 16.59 | 45.97 | 0.76 | 0.279 | 0.904 | 69 | -22.9 |
| log-sigma-5-0-mm-3D_firstorder_90Percentile | 93.50 | 259.17 | 0.436 | 0.108 | 0.673 | 267.2 | -251.2 |
| log-sigma-5-0-mm-3D_firstorder_Energy | 33.26 | 92.20 | 0.619 | 0.355 | 0.789 | 157.1 | -27.3 |
| log-sigma-5-0-mm-3D_firstorder_Entropy | 5.02 | 13.92 | 0.848 | 0.036 | 0.957 | 22.8 | -5.1 |
| log-sigma-5-0-mm-3D_firstorder_InterquartileRange | 16.59 | 45.98 | 0.765 | 0.114 | 0.918 | 76 | -15.9 |
| log-sigma-5-0-mm-3D_firstorder_Kurtosis | 8.37 | 23.19 | 0.773 | 0.474 | 0.895 | 34 | -12.4 |
| log-sigma-5-0-mm-3D_firstorder_Maximum | 264.80 | 733.99 | 0.577 | 0.18 | 0.788 | 913 | -554.9 |
| log-sigma-5-0-mm-3D_firstorder_MeanAbsoluteDeviation | 16.30 | 45.17 | 0.738 | 0.134 | 0.903 | 78.7 | -11.6 |
| log-sigma-5-0-mm-3D_firstorder_Mean | 25.88 | 71.75 | 0.67 | 0.444 | 0.817 | 79.3 | -64.2 |
| log-sigma-5-0-mm-3D_firstorder_Median | 31.76 | 88.02 | 0.512 | 0.227 | 0.716 | 82.6 | -93.5 |
| log-sigma-5-0-mm-3D_firstorder_Minimum | 17.91 | 49.63 | 0.69 | 0.23 | 0.865 | 81.8 | -17.4 |
| log-sigma-5-0-mm-3D_firstorder_Range | 17.59 | 48.77 | 0.689 | 0.162 | 0.872 | 86.5 | -11 |
| log-sigma-5-0-mm-3D_firstorder_RobustMeanAbsoluteDeviation | 16.09 | 44.61 | 0.759 | 0.106 | 0.915 | 76.1 | -13.1 |
| log-sigma-5-0-mm-3D_firstorder_RootMeanSquared | 15.56 | 43.13 | 0.756 | 0.287 | 0.901 | 63.2 | -23 |
| log-sigma-5-0-mm-3D_firstorder_Skewness | 30.68 | 85.03 | 0.8 | 0.37 | 0.921 | 110.8 | -59.3 |
| log-sigma-5-0-mm-3D_firstorder_TotalEnergy | 33.26 | 92.20 | 0.619 | 0.355 | 0.789 | 157.1 | -27.3 |
| log-sigma-5-0-mm-3D_firstorder_Uniformity | 13.90 | 38.53 | 0.878 | 0.598 | 0.951 | 10.5 | -66.6 |
| log-sigma-5-0-mm-3D_firstorder_Variance | 31.09 | 86.17 | 0.515 | 0.184 | 0.731 | 152.1 | -20.3 |
| log-sigma-5-0-mm-3D_glcm_Autocorrelation | 35.51 | 98.42 | 0.394 | 0.09 | 0.634 | 175.2 | -21.7 |
| log-sigma-5-0-mm-3D_glcm_ClusterProminence | 54.31 | 150.53 | 0.131 | -0.176 | 0.425 | 269.1 | -32 |
| log-sigma-5-0-mm-3D_glcm_ClusterShade | 71.82 | 199.06 | 0.23 | -0.074 | 0.505 | 306.2 | -91.9 |
| log-sigma-5-0-mm-3D_glcm_ClusterTendency | 32.31 | 89.55 | 0.496 | 0.172 | 0.715 | 158.8 | -20.3 |
| log-sigma-5-0-mm-3D_glcm_Contrast | 28.77 | 79.74 | 0.635 | 0.265 | 0.819 | 132 | -27.5 |
| log-sigma-5-0-mm-3D_glcm_Correlation | 4.88 | 13.53 | 0.833 | 0.309 | 0.941 | 19.5 | -7.5 |
| log-sigma-5-0-mm-3D_glcm_DifferenceAverage | 15.10 | 41.86 | 0.792 | 0.16 | 0.928 | 67.8 | -16 |
| log-sigma-5-0-mm-3D_glcm_DifferenceEntropy | 6.60 | 18.28 | 0.843 | 0.078 | 0.954 | 29 | -7.5 |
| log-sigma-5-0-mm-3D_glcm_DifferenceVariance | 28.63 | 79.36 | 0.559 | 0.232 | 0.761 | 135 | -23.8 |
| log-sigma-5-0-mm-3D_glcm_Id | 8.62 | 23.90 | 0.853 | 0.399 | 0.947 | 10.2 | -37.6 |
| log-sigma-5-0-mm-3D_glcm_Idm | 12.54 | 34.76 | 0.851 | 0.517 | 0.941 | 15.7 | -53.8 |
| log-sigma-5-0-mm-3D_glcm_Idmn | 0.36 | 1.00 | 0.81 | 0.512 | 0.916 | 1.3 | -0.7 |
| log-sigma-5-0-mm-3D_glcm_Idn | 0.88 | 2.45 | 0.836 | 0.47 | 0.935 | 3.4 | -1.5 |
| log-sigma-5-0-mm-3D_glcm_Imc1 | 9.68 | 26.82 | 0.761 | 0.499 | 0.883 | 34.1 | -19.6 |
| log-sigma-5-0-mm-3D_glcm_Imc2 | 2.29 | 6.36 | 0.856 | 0.634 | 0.936 | 8.2 | -4.5 |
| log-sigma-5-0-mm-3D_glcm_InverseVariance | 12.06 | 33.43 | 0.849 | 0.4 | 0.945 | 14.4 | -52.5 |
| log-sigma-5-0-mm-3D_glcm_JointAverage | 19.55 | 54.19 | 0.66 | 0.217 | 0.846 | 95.7 | -12.7 |
| log-sigma-5-0-mm-3D_glcm_JointEnergy | 22.14 | 61.36 | 0.851 | 0.71 | 0.924 | 15.7 | -107.1 |
| log-sigma-5-0-mm-3D_glcm_JointEntropy | 4.72 | 13.09 | 0.859 | 0.043 | 0.961 | 21.2 | -4.9 |
| log-sigma-5-0-mm-3D_glcm_MCC | 4.66 | 12.92 | 0.794 | 0.386 | 0.916 | 17.7 | -8.1 |
| log-sigma-5-0-mm-3D_glcm_MaximumProbability | 20.53 | 56.91 | 0.863 | 0.701 | 0.934 | 22.6 | -91.2 |
| log-sigma-5-0-mm-3D_glcm_SumAverage | 19.55 | 54.19 | 0.66 | 0.217 | 0.846 | 95.7 | -12.7 |
| log-sigma-5-0-mm-3D_glcm_SumEntropy | 4.40 | 12.19 | 0.845 | 0.011 | 0.958 | 20.6 | -3.8 |
| log-sigma-5-0-mm-3D_glcm_SumSquares | 31.60 | 87.59 | 0.506 | 0.18 | 0.723 | 154.9 | -20.3 |
| log-sigma-5-0-mm-3D_gldm_DependenceEntropy | 2.26 | 6.27 | 0.883 | 0.041 | 0.969 | 11.3 | -1.2 |
| log-sigma-5-0-mm-3D_gldm_DependenceNonUniformity | 19.41 | 53.79 | 0.898 | 0.805 | 0.947 | 93.2 | -14.4 |
| log-sigma-5-0-mm-3D_gldm_DependenceNonUniformityNormalized | 9.82 | 27.21 | 0.844 | 0.397 | 0.943 | 38.3 | -16.1 |
| log-sigma-5-0-mm-3D_gldm_DependenceVariance | 22.72 | 62.97 | 0.755 | 0.571 | 0.867 | 47 | -78.9 |
| log-sigma-5-0-mm-3D_gldm_GrayLevelNonUniformity | 15.84 | 43.90 | 0.963 | 0.93 | 0.981 | 44.9 | -42.9 |
| log-sigma-5-0-mm-3D_gldm_GrayLevelVariance | 31.19 | 86.45 | 0.515 | 0.184 | 0.731 | 152.3 | -20.6 |
| log-sigma-5-0-mm-3D_gldm_HighGrayLevelEmphasis | 34.86 | 96.63 | 0.408 | 0.103 | 0.645 | 171.1 | -22.1 |
| log-sigma-5-0-mm-3D_gldm_LargeDependenceEmphasis | 19.05 | 52.81 | 0.817 | 0.663 | 0.903 | 34 | -71.6 |
| log-sigma-5-0-mm-3D_gldm_LargeDependenceHighGrayLevelEmphasis | 29.35 | 81.35 | 0.414 | 0.11 | 0.65 | 144.6 | -18.1 |
| log-sigma-5-0-mm-3D_gldm_LargeDependenceLowGrayLevelEmphasis | 61.89 | 171.56 | 0.713 | 0.508 | 0.842 | 96.8 | -246.3 |
| log-sigma-5-0-mm-3D_gldm_LowGrayLevelEmphasis | 38.01 | 105.37 | 0.714 | 0.463 | 0.851 | 49.6 | -161.1 |
| log-sigma-5-0-mm-3D_gldm_SmallDependenceEmphasis | 15.05 | 41.71 | 0.856 | 0.467 | 0.946 | 56.1 | -27.3 |
| log-sigma-5-0-mm-3D_gldm_SmallDependenceHighGrayLevelEmphasis | 42.16 | 116.86 | 0.409 | 0.106 | 0.645 | 197.4 | -36.3 |
| log-sigma-5-0-mm-3D_gldm_SmallDependenceLowGrayLevelEmphasis | 26.81 | 74.31 | 0.701 | 0.463 | 0.84 | 43.6 | -105 |
| log-sigma-5-0-mm-3D_glrlm_GrayLevelNonUniformity | 15.22 | 42.18 | 0.976 | 0.954 | 0.988 | 44.1 | -40.2 |
| log-sigma-5-0-mm-3D_glrlm_GrayLevelNonUniformityNormalized | 14.08 | 39.02 | 0.877 | 0.611 | 0.95 | 10.9 | -67.1 |
| log-sigma-5-0-mm-3D_glrlm_GrayLevelVariance | 31.44 | 87.14 | 0.513 | 0.183 | 0.73 | 153 | -21.3 |
| log-sigma-5-0-mm-3D_glrlm_HighGrayLevelRunEmphasis | 34.77 | 96.39 | 0.409 | 0.103 | 0.646 | 170.6 | -22.2 |
| log-sigma-5-0-mm-3D_glrlm_LongRunEmphasis | 4.54 | 12.59 | 0.807 | 0.633 | 0.9 | 9.4 | -15.8 |
| log-sigma-5-0-mm-3D_glrlm_LongRunHighGrayLevelEmphasis | 32.91 | 91.23 | 0.406 | 0.101 | 0.644 | 163.7 | -18.7 |
| log-sigma-5-0-mm-3D_glrlm_LongRunLowGrayLevelEmphasis | 41.77 | 115.78 | 0.717 | 0.5 | 0.847 | 57.2 | -174.4 |
| log-sigma-5-0-mm-3D_glrlm_LowGrayLevelRunEmphasis | 36.75 | 101.88 | 0.724 | 0.468 | 0.859 | 46.3 | -157.5 |
| log-sigma-5-0-mm-3D_glrlm_RunEntropy | 3.78 | 10.49 | 0.846 | 0.011 | 0.958 | 18.2 | -2.8 |
| log-sigma-5-0-mm-3D_glrlm_RunLengthNonUniformity | 16.90 | 46.85 | 0.965 | 0.931 | 0.982 | 78.4 | -15.3 |
| log-sigma-5-0-mm-3D_glrlm_RunLengthNonUniformityNormalized | 2.32 | 6.42 | 0.862 | 0.655 | 0.938 | 8.2 | -4.6 |
| log-sigma-5-0-mm-3D_glrlm_RunPercentage | 1.37 | 3.81 | 0.85 | 0.661 | 0.929 | 4.8 | -2.8 |
| log-sigma-5-0-mm-3D_glrlm_RunVariance | 20.38 | 56.48 | 0.761 | 0.566 | 0.873 | 38.6 | -74.4 |
| log-sigma-5-0-mm-3D_glrlm_ShortRunEmphasis | 1.00 | 2.77 | 0.86 | 0.682 | 0.934 | 3.5 | -2 |
| log-sigma-5-0-mm-3D_glrlm_ShortRunHighGrayLevelEmphasis | 35.23 | 97.64 | 0.409 | 0.104 | 0.646 | 172.2 | -23.1 |
| log-sigma-5-0-mm-3D_glrlm_ShortRunLowGrayLevelEmphasis | 35.16 | 97.45 | 0.721 | 0.439 | 0.861 | 42.6 | -152.3 |
| log-sigma-5-0-mm-3D_glszm_GrayLevelNonUniformity | 10.64 | 29.48 | 0.954 | 0.912 | 0.976 | 42.6 | -16.4 |
| log-sigma-5-0-mm-3D_glszm_GrayLevelNonUniformityNormalized | 17.32 | 48.01 | 0.863 | 0.7 | 0.934 | 15.9 | -80.1 |
| log-sigma-5-0-mm-3D_glszm_GrayLevelVariance | 32.49 | 90.07 | 0.491 | 0.168 | 0.712 | 156.6 | -23.6 |
| log-sigma-5-0-mm-3D_glszm_HighGrayLevelZoneEmphasis | 33.62 | 93.18 | 0.419 | 0.113 | 0.654 | 162.4 | -23.9 |
| log-sigma-5-0-mm-3D_glszm_LargeAreaEmphasis | 40.15 | 111.30 | 0.454 | 0.161 | 0.676 | 68.5 | -154.1 |
| log-sigma-5-0-mm-3D_glszm_LargeAreaHighGrayLevelEmphasis | 42.22 | 117.01 | 0.606 | 0.352 | 0.777 | 153.5 | -80.5 |
| log-sigma-5-0-mm-3D_glszm_LargeAreaLowGrayLevelEmphasis | 59.14 | 163.92 | 0.724 | 0.525 | 0.849 | 72.8 | -255 |
| log-sigma-5-0-mm-3D_glszm_LowGrayLevelZoneEmphasis | 26.85 | 74.42 | 0.889 | 0.728 | 0.949 | 24.1 | -124.7 |
| log-sigma-5-0-mm-3D_glszm_SizeZoneNonUniformity | 28.42 | 78.78 | 0.722 | 0.512 | 0.85 | 129.4 | -28.1 |
| log-sigma-5-0-mm-3D_glszm_SizeZoneNonUniformityNormalized | 11.99 | 33.23 | 0.828 | 0.604 | 0.92 | 40.4 | -26 |
| log-sigma-5-0-mm-3D_glszm_SmallAreaEmphasis | 7.41 | 20.55 | 0.816 | 0.645 | 0.906 | 23.8 | -17.3 |
| log-sigma-5-0-mm-3D_glszm_SmallAreaHighGrayLevelEmphasis | 38.32 | 106.21 | 0.413 | 0.109 | 0.648 | 175.7 | -36.7 |
| log-sigma-5-0-mm-3D_glszm_SmallAreaLowGrayLevelEmphasis | 41.13 | 114.01 | 0.327 | 0.005 | 0.588 | 81.3 | -146.7 |
| log-sigma-5-0-mm-3D_glszm_ZoneEntropy | 4.63 | 12.83 | 0.856 | 0.155 | 0.956 | 19.9 | -5.8 |
| log-sigma-5-0-mm-3D_glszm_ZonePercentage | 15.44 | 42.80 | 0.86 | 0.458 | 0.948 | 58.9 | -26.7 |
| log-sigma-5-0-mm-3D_glszm_ZoneVariance | 47.88 | 132.71 | 0.378 | 0.071 | 0.622 | 82.6 | -182.8 |
| log-sigma-5-0-mm-3D_ngtdm_Busyness | 31.33 | 86.84 | 0.727 | 0.497 | 0.857 | 24.2 | -149.5 |
| log-sigma-5-0-mm-3D_ngtdm_Coarseness | 13.85 | 38.40 | 0.86 | 0.524 | 0.945 | 21.1 | -55.7 |
| log-sigma-5-0-mm-3D_ngtdm_Complexity | 42.51 | 117.83 | 0.434 | 0.131 | 0.664 | 200.6 | -35.1 |
| log-sigma-5-0-mm-3D_ngtdm_Contrast | 17.23 | 47.76 | 0.822 | 0.615 | 0.914 | 60.2 | -35.3 |
| log-sigma-5-0-mm-3D_ngtdm_Strength | 32.65 | 90.50 | 0.453 | 0.136 | 0.683 | 154.9 | -26.1 |
| logarithm_firstorder_10Percentile | 35.52 | 98.46 | 0 | -0.076 | 0.122 | 158.7 | -38.2 |
| logarithm_firstorder_90Percentile | 36.72 | 101.78 | 0 | -0.081 | 0.129 | 168.1 | -35.4 |
| logarithm_firstorder_Energy | 59.06 | 163.70 | 0.193 | -0.127 | 0.482 | 288.2 | -39.2 |
| logarithm_firstorder_Entropy | 9.83 | 27.25 | 0.204 | -0.095 | 0.501 | 43.9 | -10.6 |
| logarithm_firstorder_InterquartileRange | 40.50 | 112.27 | 0.138 | -0.088 | 0.402 | 186.4 | -38.2 |
| logarithm_firstorder_Kurtosis | 5.02 | 13.92 | 0.75 | 0.565 | 0.864 | 14.3 | -13.6 |
| logarithm_firstorder_Maximum | 36.74 | 101.84 | 0 | -0.085 | 0.134 | 169.7 | -34 |
| logarithm_firstorder_MeanAbsoluteDeviation | 39.71 | 110.07 | 0.134 | -0.087 | 0.396 | 183.9 | -36.2 |
| logarithm_firstorder_Mean | 36.25 | 100.49 | 0 | -0.076 | 0.122 | 164.1 | -36.9 |
| logarithm_firstorder_Median | 36.28 | 100.57 | 0 | -0.076 | 0.122 | 163.5 | -37.6 |
| logarithm_firstorder_Minimum | 34.79 | 96.43 | 0 | -0.11 | 0.162 | 152.1 | -40.8 |
| logarithm_firstorder_Range | 38.74 | 107.40 | 0.125 | -0.085 | 0.381 | 181.2 | -33.6 |
| logarithm_firstorder_RobustMeanAbsoluteDeviation | 40.01 | 110.90 | 0.138 | -0.088 | 0.403 | 185.1 | -36.7 |
| logarithm_firstorder_RootMeanSquared | 36.32 | 100.68 | 0 | -0.076 | 0.122 | 164.7 | -36.6 |
| logarithm_firstorder_Skewness | 799.50 | 2216.11 | 0.767 | 0.454 | 0.893 | 2352.7 | -2079.6 |
| logarithm_firstorder_TotalEnergy | 59.06 | 163.70 | 0.193 | -0.127 | 0.482 | 288.2 | -39.2 |
| logarithm_firstorder_Uniformity | 36.25 | 100.49 | 0.258 | -0.056 | 0.534 | 32.5 | -168.5 |
| logarithm_firstorder_Variance | 61.99 | 171.83 | 0.108 | -0.085 | 0.345 | 292.2 | -51.5 |
| logarithm_glcm_Autocorrelation | 60.70 | 168.26 | 0.054 | -0.078 | 0.238 | 284.2 | -52.3 |
| logarithm_glcm_ClusterProminence | 75.65 | 209.70 | 0.062 | -0.147 | 0.309 | 366 | -53.4 |
| logarithm_glcm_ClusterShade | 206.20 | 571.55 | 0.123 | -0.176 | 0.415 | 682.6 | -460.5 |
| logarithm_glcm_ClusterTendency | 61.76 | 171.18 | 0.132 | -0.089 | 0.386 | 293.5 | -48.9 |
| logarithm_glcm_Contrast | 62.23 | 172.49 | 0 | -0.113 | 0.166 | 283.6 | -61.3 |
| logarithm_glcm_Correlation | 5.21 | 14.45 | 0.814 | 0.198 | 0.937 | 21.5 | -7.4 |
| logarithm_glcm_DifferenceAverage | 39.87 | 110.51 | 0.017 | -0.098 | 0.184 | 177.1 | -43.9 |
| logarithm_glcm_DifferenceEntropy | 12.66 | 35.10 | 0.083 | -0.088 | 0.301 | 55.2 | -15 |
| logarithm_glcm_DifferenceVariance | 62.10 | 172.15 | 0 | -0.108 | 0.161 | 283.7 | -60.6 |
| logarithm_glcm_Id | 28.77 | 79.75 | 0.109 | -0.098 | 0.349 | 32.6 | -126.9 |
| logarithm_glcm_Idm | 38.44 | 106.54 | 0.118 | -0.104 | 0.367 | 44.5 | -168.6 |
| logarithm_glcm_Idmn | 0.32 | 0.90 | 0.814 | 0.464 | 0.922 | 1.2 | -0.6 |
| logarithm_glcm_Idn | 0.68 | 1.88 | 0.854 | 0.493 | 0.943 | 2.6 | -1.1 |
| logarithm_glcm_Imc1 | 23.32 | 64.63 | 0.296 | -0.041 | 0.574 | 92.7 | -36.6 |
| logarithm_glcm_Imc2 | 2.03 | 5.62 | 0.121 | -0.152 | 0.399 | 7 | -4.2 |
| logarithm_glcm_InverseVariance | 37.93 | 105.14 | 0.132 | -0.097 | 0.384 | 42.9 | -167.4 |
| logarithm_glcm_JointAverage | 38.56 | 106.88 | 0.073 | -0.077 | 0.275 | 176.3 | -37.4 |
| logarithm_glcm_JointEnergy | 33.31 | 92.32 | 0.617 | 0.152 | 0.824 | 26.6 | -158.1 |
| logarithm_glcm_JointEntropy | 5.24 | 14.52 | 0.656 | -0.031 | 0.876 | 24.1 | -5 |
| logarithm_glcm_MCC | 6.94 | 19.24 | 0.176 | -0.086 | 0.441 | 26.5 | -12 |
| logarithm_glcm_MaximumProbability | 35.36 | 98.02 | 0.435 | 0.054 | 0.689 | 31 | -165 |
| logarithm_glcm_SumAverage | 38.56 | 106.88 | 0.073 | -0.077 | 0.275 | 176.3 | -37.4 |
| logarithm_glcm_SumEntropy | 7.64 | 21.17 | 0.339 | -0.104 | 0.674 | 36 | -6.3 |
| logarithm_glcm_SumSquares | 61.81 | 171.33 | 0.116 | -0.087 | 0.358 | 292.3 | -50.3 |
| logarithm_gldm_DependenceEntropy | 4.09 | 11.33 | 0.584 | -0.09 | 0.852 | 19.8 | -2.9 |
| logarithm_gldm_DependenceNonUniformity | 25.64 | 71.06 | 0.668 | 0.441 | 0.815 | 131.8 | -10.3 |
| logarithm_gldm_DependenceNonUniformityNormalized | 24.28 | 67.31 | 0.064 | -0.102 | 0.278 | 101.1 | -33.5 |
| logarithm_gldm_DependenceVariance | 45.46 | 126.01 | 0.165 | -0.1 | 0.433 | 61.8 | -190.2 |
| logarithm_gldm_GrayLevelNonUniformity | 39.77 | 110.25 | 0.608 | 0.358 | 0.778 | 68.6 | -151.9 |
| logarithm_gldm_GrayLevelVariance | 62.01 | 171.87 | 0.108 | -0.085 | 0.345 | 292.2 | -51.6 |
| logarithm_gldm_HighGrayLevelEmphasis | 60.68 | 168.19 | 0.057 | -0.078 | 0.244 | 284.4 | -52 |
| logarithm_gldm_LargeDependenceEmphasis | 34.93 | 96.81 | 0.102 | -0.138 | 0.365 | 51.4 | -142.3 |
| logarithm_gldm_LargeDependenceHighGrayLevelEmphasis | 42.48 | 117.75 | 0.292 | -0.099 | 0.611 | 205.2 | -30.3 |
| logarithm_gldm_LargeDependenceLowGrayLevelEmphasis | 54.93 | 152.26 | 0.127 | -0.178 | 0.421 | 51.7 | -252.8 |
| logarithm_gldm_LowGrayLevelEmphasis | 40.32 | 111.76 | 0.483 | 0.14 | 0.711 | 36 | -187.5 |
| logarithm_gldm_SmallDependenceEmphasis | 19.63 | 54.42 | 0.066 | -0.119 | 0.294 | 76.1 | -32.8 |
| logarithm_gldm_SmallDependenceHighGrayLevelEmphasis | 67.14 | 186.11 | 0.039 | -0.081 | 0.211 | 312.7 | -59.5 |
| logarithm_gldm_SmallDependenceLowGrayLevelEmphasis | 34.56 | 95.79 | 0.616 | 0.198 | 0.816 | 29.5 | -162.1 |
| logarithm_glrlm_GrayLevelNonUniformity | 38.94 | 107.93 | 0.633 | 0.393 | 0.794 | 67.4 | -148.4 |
| logarithm_glrlm_GrayLevelNonUniformityNormalized | 36.27 | 100.52 | 0.258 | -0.056 | 0.535 | 32.5 | -168.5 |
| logarithm_glrlm_GrayLevelVariance | 62.09 | 172.10 | 0.108 | -0.085 | 0.343 | 292.4 | -51.8 |
| logarithm_glrlm_HighGrayLevelRunEmphasis | 60.78 | 168.47 | 0.056 | -0.078 | 0.243 | 284.8 | -52.1 |
| logarithm_glrlm_LongRunEmphasis | 4.08 | 11.30 | 0.094 | -0.134 | 0.349 | 7.1 | -15.5 |
| logarithm_glrlm_LongRunHighGrayLevelEmphasis | 59.31 | 164.39 | 0.063 | -0.078 | 0.256 | 278.5 | -50.3 |
| logarithm_glrlm_LongRunLowGrayLevelEmphasis | 41.68 | 115.52 | 0.456 | 0.126 | 0.689 | 37.6 | -193.4 |
| logarithm_glrlm_LowGrayLevelRunEmphasis | 40.52 | 112.30 | 0.486 | 0.143 | 0.713 | 36.1 | -188.5 |
| logarithm_glrlm_RunEntropy | 8.74 | 24.23 | 0.232 | -0.098 | 0.543 | 39.7 | -8.8 |
| logarithm_glrlm_RunLengthNonUniformity | 15.58 | 43.20 | 0.958 | 0.919 | 0.979 | 75.8 | -10.5 |
| logarithm_glrlm_RunLengthNonUniformityNormalized | 2.34 | 6.48 | 0.09 | -0.124 | 0.338 | 9 | -4 |
| logarithm_glrlm_RunPercentage | 1.28 | 3.54 | 0.097 | -0.124 | 0.348 | 4.9 | -2.2 |
| logarithm_glrlm_RunVariance | 44.89 | 124.44 | 0.101 | -0.136 | 0.362 | 60.3 | -188.6 |
| logarithm_glrlm_ShortRunEmphasis | 0.93 | 2.57 | 0.089 | -0.128 | 0.338 | 3.6 | -1.6 |
| logarithm_glrlm_ShortRunHighGrayLevelEmphasis | 61.14 | 169.47 | 0.055 | -0.078 | 0.24 | 286.4 | -52.6 |
| logarithm_glrlm_ShortRunLowGrayLevelEmphasis | 40.25 | 111.57 | 0.493 | 0.146 | 0.719 | 35.8 | -187.4 |
| logarithm_glszm_GrayLevelNonUniformity | 29.55 | 81.90 | 0.851 | 0.729 | 0.921 | 54.7 | -109.1 |
| logarithm_glszm_GrayLevelNonUniformityNormalized | 37.46 | 103.82 | 0.256 | -0.054 | 0.531 | 34.6 | -173 |
| logarithm_glszm_GrayLevelVariance | 62.74 | 173.91 | 0.098 | -0.085 | 0.326 | 294.8 | -53 |
| logarithm_glszm_HighGrayLevelZoneEmphasis | 62.08 | 172.09 | 0.051 | -0.078 | 0.233 | 290.6 | -53.5 |
| logarithm_glszm_LargeAreaEmphasis | 47.62 | 131.98 | 0.017 | -0.287 | 0.329 | 78.1 | -185.9 |
| logarithm_glszm_LargeAreaHighGrayLevelEmphasis | 47.83 | 132.58 | 0.455 | 0.156 | 0.678 | 204.6 | -60.5 |
| logarithm_glszm_LargeAreaLowGrayLevelEmphasis | 56.45 | 156.46 | 0.097 | -0.221 | 0.402 | 55.5 | -257.4 |
| logarithm_glszm_LowGrayLevelZoneEmphasis | 43.69 | 121.10 | 0.493 | 0.163 | 0.715 | 39.5 | -202.7 |
| logarithm_glszm_SizeZoneNonUniformity | 33.05 | 91.62 | 0.606 | 0.356 | 0.777 | 155.7 | -27.5 |
| logarithm_glszm_SizeZoneNonUniformityNormalized | 18.34 | 50.83 | 0.039 | -0.13 | 0.256 | 71.7 | -29.9 |
| logarithm_glszm_SmallAreaEmphasis | 9.12 | 25.27 | 0.049 | -0.141 | 0.283 | 34.9 | -15.6 |
| logarithm_glszm_SmallAreaHighGrayLevelEmphasis | 65.45 | 181.41 | 0.044 | -0.08 | 0.219 | 305 | -57.8 |
| logarithm_glszm_SmallAreaLowGrayLevelEmphasis | 40.98 | 113.59 | 0.564 | 0.198 | 0.773 | 35.9 | -191.3 |
| logarithm_glszm_ZoneEntropy | 5.03 | 13.93 | 0.439 | -0.102 | 0.763 | 24.5 | -3.4 |
| logarithm_glszm_ZonePercentage | 17.00 | 47.13 | 0.078 | -0.122 | 0.317 | 64.6 | -29.7 |
| logarithm_glszm_ZoneVariance | 64.64 | 179.16 | 0.016 | -0.295 | 0.33 | 87.2 | -271.1 |
| logarithm_ngtdm_Busyness | 59.16 | 163.97 | 0.145 | -0.154 | 0.433 | 63.2 | -264.7 |
| logarithm_ngtdm_Coarseness | 14.75 | 40.89 | 0.787 | 0.322 | 0.916 | 17.7 | -64.1 |
| logarithm_ngtdm_Complexity | 70.29 | 194.82 | 0.051 | -0.096 | 0.248 | 332.7 | -56.9 |
| logarithm_ngtdm_Contrast | 47.27 | 131.02 | 0 | -0.166 | 0.219 | 198.7 | -63.3 |
| logarithm_ngtdm_Strength | 58.95 | 163.39 | 0.028 | -0.12 | 0.229 | 272.3 | -54.5 |
| original_firstorder_10Percentile | 5.96 | 16.52 | 0.758 | 0.577 | 0.869 | 18.3 | -14.8 |
| original_firstorder_90Percentile | 12.66 | 35.10 | 0.728 | 0.124 | 0.898 | 58.3 | -11.9 |
| original_firstorder_Energy | 29.85 | 82.73 | 0.73 | 0.527 | 0.854 | 146.4 | -19.1 |
| original_firstorder_Entropy | 4.34 | 12.02 | 0.859 | 0.201 | 0.956 | 18.2 | -5.8 |
| original_firstorder_InterquartileRange | 17.91 | 49.64 | 0.708 | 0.158 | 0.884 | 77.5 | -21.8 |
| original_firstorder_Kurtosis | 10.21 | 28.31 | 0.765 | 0.421 | 0.895 | 41.1 | -15.5 |
| original_firstorder_Maximum | 15.50 | 42.97 | 0.675 | 0.153 | 0.864 | 74.3 | -11.6 |
| original_firstorder_MeanAbsoluteDeviation | 17.40 | 48.23 | 0.713 | 0.116 | 0.89 | 79.7 | -16.7 |
| original_firstorder_Mean | 9.36 | 25.94 | 0.761 | 0.147 | 0.914 | 41.5 | -10.4 |
| original_firstorder_Median | 7.58 | 21.00 | 0.809 | 0.344 | 0.927 | 30.8 | -11.2 |
| original_firstorder_Minimum | 10.48 | 29.04 | 0.751 | 0.531 | 0.871 | 22.1 | -36 |
| original_firstorder_Range | 17.87 | 49.52 | 0.679 | 0.144 | 0.867 | 85 | -14 |
| original_firstorder_RobustMeanAbsoluteDeviation | 17.44 | 48.33 | 0.713 | 0.134 | 0.889 | 77.6 | -19 |
| original_firstorder_RootMeanSquared | 11.09 | 30.75 | 0.728 | 0.136 | 0.897 | 50.2 | -11.3 |
| original_firstorder_Skewness | 26.90 | 74.56 | 0.809 | 0.15 | 0.937 | 100.1 | -49 |
| original_firstorder_TotalEnergy | 29.85 | 82.73 | 0.73 | 0.527 | 0.854 | 146.4 | -19.1 |
| original_firstorder_Uniformity | 14.85 | 41.15 | 0.895 | 0.773 | 0.949 | 20.2 | -62.1 |
| original_firstorder_Variance | 32.86 | 91.08 | 0.512 | 0.159 | 0.735 | 153.5 | -28.6 |
| original_glcm_Autocorrelation | 27.44 | 76.05 | 0.589 | 0.159 | 0.802 | 125.1 | -27 |
| original_glcm_ClusterProminence | 55.98 | 155.16 | 0.144 | -0.163 | 0.436 | 271.4 | -39 |
| original_glcm_ClusterShade | 54.79 | 151.88 | 0.25 | -0.053 | 0.521 | 263.2 | -40.6 |
| original_glcm_ClusterTendency | 33.58 | 93.09 | 0.502 | 0.158 | 0.725 | 159.5 | -26.7 |
| original_glcm_Contrast | 27.80 | 77.06 | 0.659 | 0.253 | 0.84 | 123.9 | -30.2 |
| original_glcm_Correlation | 5.16 | 14.31 | 0.824 | 0.188 | 0.942 | 21.3 | -7.3 |
| original_glcm_DifferenceAverage | 14.47 | 40.10 | 0.808 | 0.159 | 0.936 | 62.4 | -17.8 |
| original_glcm_DifferenceEntropy | 5.22 | 14.48 | 0.855 | 0.142 | 0.956 | 22.4 | -6.5 |
| original_glcm_DifferenceVariance | 28.51 | 79.02 | 0.577 | 0.229 | 0.778 | 132.1 | -26 |
| original_glcm_Id | 9.14 | 25.33 | 0.873 | 0.656 | 0.945 | 13.9 | -36.7 |
| original_glcm_Idm | 13.21 | 36.63 | 0.868 | 0.723 | 0.935 | 22.1 | -51.2 |
| original_glcm_Idmn | 0.34 | 0.93 | 0.818 | 0.267 | 0.936 | 1.4 | -0.5 |
| original_glcm_Idn | 0.79 | 2.19 | 0.83 | 0.183 | 0.944 | 3.4 | -0.9 |
| original_glcm_Imc1 | 9.23 | 25.59 | 0.892 | 0.79 | 0.945 | 30.7 | -20.5 |
| original_glcm_Imc2 | 1.61 | 4.46 | 0.881 | 0.771 | 0.939 | 5.2 | -3.7 |
| original_glcm_InverseVariance | 12.39 | 34.33 | 0.876 | 0.703 | 0.943 | 19 | -49.7 |
| original_glcm_JointAverage | 13.37 | 37.07 | 0.768 | 0.097 | 0.921 | 59 | -15.1 |
| original_glcm_JointEnergy | 20.56 | 56.99 | 0.878 | 0.769 | 0.937 | 22 | -92 |
| original_glcm_JointEntropy | 3.50 | 9.71 | 0.882 | 0.151 | 0.966 | 15.5 | -4 |
| original_glcm_MCC | 4.81 | 13.33 | 0.748 | 0.51 | 0.871 | 16.4 | -10.2 |
| original_glcm_MaximumProbability | 20.74 | 57.49 | 0.825 | 0.685 | 0.906 | 34.1 | -80.9 |
| original_glcm_SumAverage | 13.37 | 37.07 | 0.768 | 0.097 | 0.921 | 59 | -15.1 |
| original_glcm_SumEntropy | 3.87 | 10.73 | 0.856 | 0.088 | 0.958 | 17.2 | -4.3 |
| original_glcm_SumSquares | 32.74 | 90.76 | 0.513 | 0.165 | 0.734 | 154.8 | -26.7 |
| original_gldm_DependenceEntropy | 2.23 | 6.17 | 0.881 | 0.101 | 0.967 | 10.5 | -1.9 |
| original_gldm_DependenceNonUniformity | 18.25 | 50.60 | 0.906 | 0.821 | 0.951 | 85.6 | -15.6 |
| original_gldm_DependenceNonUniformityNormalized | 10.05 | 27.85 | 0.84 | 0.663 | 0.921 | 34.2 | -21.5 |
| original_gldm_DependenceVariance | 23.52 | 65.20 | 0.637 | 0.396 | 0.796 | 57.2 | -73.2 |
| original_gldm_GrayLevelNonUniformity | 18.87 | 52.29 | 0.96 | 0.924 | 0.979 | 60.4 | -44.2 |
| original_gldm_GrayLevelVariance | 32.75 | 90.77 | 0.512 | 0.159 | 0.735 | 153.2 | -28.4 |
| original_gldm_HighGrayLevelEmphasis | 28.25 | 78.30 | 0.568 | 0.153 | 0.785 | 128 | -28.6 |
| original_gldm_LargeDependenceEmphasis | 18.07 | 50.10 | 0.763 | 0.585 | 0.871 | 41.4 | -58.8 |
| original_gldm_LargeDependenceHighGrayLevelEmphasis | 21.05 | 58.34 | 0.675 | 0.224 | 0.855 | 93.8 | -22.9 |
| original_gldm_LargeDependenceLowGrayLevelEmphasis | 48.67 | 134.90 | 0.585 | 0.324 | 0.764 | 110.4 | -159.4 |
| original_gldm_LowGrayLevelEmphasis | 28.67 | 79.46 | 0.605 | 0.346 | 0.777 | 56.6 | -102.4 |
| original_gldm_SmallDependenceEmphasis | 11.71 | 32.45 | 0.853 | 0.699 | 0.926 | 37.9 | -27 |
| original_gldm_SmallDependenceHighGrayLevelEmphasis | 37.68 | 104.45 | 0.552 | 0.173 | 0.767 | 162 | -46.9 |
| original_gldm_SmallDependenceLowGrayLevelEmphasis | 29.06 | 80.55 | 0.49 | 0.203 | 0.701 | 56.6 | -104.5 |
| original_glrlm_GrayLevelNonUniformity | 18.31 | 50.76 | 0.967 | 0.936 | 0.983 | 58.9 | -42.7 |
| original_glrlm_GrayLevelNonUniformityNormalized | 14.67 | 40.66 | 0.897 | 0.77 | 0.951 | 19.4 | -61.9 |
| original_glrlm_GrayLevelVariance | 32.76 | 90.81 | 0.511 | 0.159 | 0.734 | 153.3 | -28.3 |
| original_glrlm_HighGrayLevelRunEmphasis | 28.29 | 78.42 | 0.567 | 0.153 | 0.784 | 128.3 | -28.5 |
| original_glrlm_LongRunEmphasis | 2.66 | 7.36 | 0.812 | 0.664 | 0.899 | 6.4 | -8.3 |
| original_glrlm_LongRunHighGrayLevelEmphasis | 26.97 | 74.75 | 0.568 | 0.15 | 0.786 | 123.1 | -26.4 |
| original_glrlm_LongRunLowGrayLevelEmphasis | 29.89 | 82.84 | 0.626 | 0.377 | 0.79 | 59.8 | -105.9 |
| original_glrlm_LowGrayLevelRunEmphasis | 28.43 | 78.80 | 0.615 | 0.36 | 0.784 | 55.6 | -102 |
| original_glrlm_RunEntropy | 3.51 | 9.73 | 0.858 | 0.113 | 0.959 | 15.7 | -3.8 |
| original_glrlm_RunLengthNonUniformity | 16.36 | 45.35 | 0.976 | 0.952 | 0.988 | 75.1 | -15.6 |
| original_glrlm_RunLengthNonUniformityNormalized | 1.55 | 4.28 | 0.846 | 0.717 | 0.919 | 4.9 | -3.7 |
| original_glrlm_RunPercentage | 0.84 | 2.34 | 0.839 | 0.707 | 0.915 | 2.6 | -2 |
| original_glrlm_RunVariance | 20.15 | 55.86 | 0.776 | 0.606 | 0.879 | 46.3 | -65.4 |
| original_glrlm_ShortRunEmphasis | 0.63 | 1.75 | 0.842 | 0.713 | 0.916 | 2 | -1.5 |
| original_glrlm_ShortRunHighGrayLevelEmphasis | 28.75 | 79.68 | 0.567 | 0.154 | 0.784 | 129.9 | -29.4 |
| original_glrlm_ShortRunLowGrayLevelEmphasis | 28.13 | 77.98 | 0.614 | 0.357 | 0.784 | 54.8 | -101.2 |
| original_glszm_GrayLevelNonUniformity | 14.12 | 39.13 | 0.999 | 0.998 | 1 | 48.5 | -29.8 |
| original_glszm_GrayLevelNonUniformityNormalized | 15.60 | 43.25 | 0.897 | 0.778 | 0.95 | 18.7 | -67.8 |
| original_glszm_GrayLevelVariance | 31.77 | 88.07 | 0.496 | 0.153 | 0.72 | 151.1 | -25 |
| original_glszm_HighGrayLevelZoneEmphasis | 29.84 | 82.71 | 0.551 | 0.157 | 0.77 | 135.4 | -30 |
| original_glszm_LargeAreaEmphasis | 32.25 | 89.41 | 0.543 | 0.268 | 0.737 | 72.7 | -106.1 |
| original_glszm_LargeAreaHighGrayLevelEmphasis | 29.33 | 81.30 | 0.594 | 0.337 | 0.77 | 108.1 | -54.5 |
| original_glszm_LargeAreaLowGrayLevelEmphasis | 47.79 | 132.45 | 0.695 | 0.481 | 0.832 | 103.7 | -161.2 |
| original_glszm_LowGrayLevelZoneEmphasis | 29.59 | 82.01 | 0.681 | 0.455 | 0.824 | 53.4 | -110.7 |
| original_glszm_SizeZoneNonUniformity | 26.53 | 73.54 | 0.852 | 0.726 | 0.922 | 111.6 | -35.4 |
| original_glszm_SizeZoneNonUniformityNormalized | 10.66 | 29.55 | 0.824 | 0.656 | 0.911 | 34.5 | -24.6 |
| original_glszm_SmallAreaEmphasis | 5.99 | 16.61 | 0.826 | 0.678 | 0.908 | 18.8 | -14.4 |
| original_glszm_SmallAreaHighGrayLevelEmphasis | 36.01 | 99.81 | 0.54 | 0.164 | 0.759 | 155.4 | -44.2 |
| original_glszm_SmallAreaLowGrayLevelEmphasis | 32.52 | 90.14 | 0.647 | 0.405 | 0.804 | 61 | -119.3 |
| original_glszm_ZoneEntropy | 2.82 | 7.81 | 0.865 | 0.113 | 0.961 | 12.8 | -2.8 |
| original_glszm_ZonePercentage | 10.74 | 29.76 | 0.868 | 0.73 | 0.934 | 34.6 | -24.9 |
| original_glszm_ZoneVariance | 41.62 | 115.35 | 0.443 | 0.141 | 0.67 | 93 | -137.7 |
| original_ngtdm_Busyness | 27.83 | 77.15 | 0.719 | 0.505 | 0.848 | 43.1 | -111.2 |
| original_ngtdm_Coarseness | 13.77 | 38.18 | 0.864 | 0.57 | 0.945 | 19 | -57.3 |
| original_ngtdm_Complexity | 39.26 | 108.82 | 0.467 | 0.159 | 0.69 | 184.8 | -32.9 |
| original_ngtdm_Contrast | 17.55 | 48.65 | 0.855 | 0.648 | 0.934 | 59.7 | -37.6 |
| original_ngtdm_Strength | 32.39 | 89.78 | 0.473 | 0.139 | 0.702 | 152.8 | -26.8 |
| square_firstorder_10Percentile | 43.71 | 121.17 | 0 | -0.109 | 0.161 | 48.3 | -194.1 |
| square_firstorder_90Percentile | 41.49 | 114.99 | 0.784 | 0.61 | 0.885 | 79.5 | -150.5 |
| square_firstorder_Energy | 77.94 | 216.03 | 0.544 | 0.269 | 0.738 | 181.1 | -251 |
| square_firstorder_Entropy | 18.96 | 52.55 | 0.672 | 0.224 | 0.853 | 34.1 | -71 |
| square_firstorder_InterquartileRange | 39.27 | 108.86 | 0.685 | 0.43 | 0.833 | 68.3 | -149.4 |
| square_firstorder_Kurtosis | 12.97 | 35.95 | 0.764 | 0.35 | 0.901 | 58.9 | -13 |
| square_firstorder_Maximum | 44.54 | 123.45 | 0.777 | 0.606 | 0.879 | 103.6 | -143.3 |
| square_firstorder_MeanAbsoluteDeviation | 41.29 | 114.44 | 0.821 | 0.678 | 0.904 | 85.1 | -143.8 |
| square_firstorder_Mean | 42.41 | 117.56 | 0.696 | 0.407 | 0.845 | 75.3 | -159.8 |
| square_firstorder_Median | 40.74 | 112.92 | 0.3 | -0.082 | 0.604 | 53.2 | -172.7 |
| square_firstorder_Minimum | 47.86 | 132.67 | 0 | -0.161 | 0.214 | 48.3 | -217 |
| square_firstorder_Range | 44.29 | 122.78 | 0.778 | 0.608 | 0.88 | 103.8 | -141.7 |
| square_firstorder_RobustMeanAbsoluteDeviation | 39.80 | 110.33 | 0.753 | 0.555 | 0.868 | 73.7 | -147 |
| square_firstorder_RootMeanSquared | 43.40 | 120.29 | 0.79 | 0.629 | 0.887 | 86.4 | -154.1 |
| square_firstorder_Skewness | 13.60 | 37.70 | 0.816 | 0.132 | 0.941 | 57.6 | -17.8 |
| square_firstorder_TotalEnergy | 77.94 | 216.03 | 0.544 | 0.269 | 0.738 | 181.1 | -251 |
| square_firstorder_Uniformity | 31.93 | 88.52 | 0.513 | 0.124 | 0.744 | 136.7 | -40.3 |
| square_firstorder_Variance | 70.23 | 194.68 | 0.666 | 0.44 | 0.814 | 149.7 | -239.6 |
| square_glcm_Autocorrelation | 65.24 | 180.84 | 0.718 | 0.515 | 0.845 | 126.7 | -235 |
| square_glcm_ClusterProminence | 103.30 | 286.33 | 0.208 | -0.112 | 0.494 | 242 | -330.7 |
| square_glcm_ClusterShade | 94.55 | 262.08 | 0.371 | 0.063 | 0.617 | 220.9 | -303.3 |
| square_glcm_ClusterTendency | 67.98 | 188.43 | 0.666 | 0.439 | 0.814 | 148.5 | -228.3 |
| square_glcm_Contrast | 62.64 | 173.62 | 0.769 | 0.594 | 0.875 | 118.8 | -228.5 |
| square_glcm_Correlation | 9.18 | 25.45 | 0.83 | 0.579 | 0.923 | 30.3 | -20.6 |
| square_glcm_DifferenceAverage | 37.19 | 103.09 | 0.8 | 0.57 | 0.904 | 65 | -141.2 |
| square_glcm_DifferenceEntropy | 18.92 | 52.44 | 0.76 | 0.436 | 0.89 | 36 | -68.8 |
| square_glcm_DifferenceVariance | 62.37 | 172.89 | 0.698 | 0.486 | 0.833 | 130.7 | -215 |
| square_glcm_Id | 16.88 | 46.79 | 0.582 | 0.061 | 0.813 | 71.9 | -21.7 |
| square_glcm_Idm | 23.04 | 63.86 | 0.564 | 0.061 | 0.8 | 99.5 | -28.2 |
| square_glcm_Idmn | 0.56 | 1.56 | 0.747 | 0.513 | 0.87 | 1.9 | -1.2 |
| square_glcm_Idn | 0.77 | 2.14 | 0.789 | -0.034 | 0.939 | 3.8 | -0.4 |
| square_glcm_Imc1 | 19.67 | 54.52 | 0.578 | 0.315 | 0.76 | 52.9 | -56.1 |
| square_glcm_Imc2 | 11.14 | 30.87 | 0.611 | 0.356 | 0.781 | 25.6 | -36.1 |
| square_glcm_InverseVariance | 21.38 | 59.25 | 0.504 | 0.159 | 0.727 | 82.6 | -35.9 |
| square_glcm_JointAverage | 36.23 | 100.44 | 0.713 | 0.417 | 0.857 | 63.1 | -137.8 |
| square_glcm_JointEnergy | 47.28 | 131.07 | 0.521 | 0.207 | 0.731 | 213.4 | -48.7 |
| square_glcm_JointEntropy | 16.55 | 45.89 | 0.702 | 0.238 | 0.872 | 29.6 | -62.2 |
| square_glcm_MCC | 10.17 | 28.19 | 0.77 | 0.594 | 0.876 | 30.2 | -26.1 |
| square_glcm_MaximumProbability | 40.60 | 112.55 | 0.496 | 0.075 | 0.741 | 188.9 | -36.2 |
| square_glcm_SumAverage | 36.23 | 100.44 | 0.713 | 0.417 | 0.857 | 63.1 | -137.8 |
| square_glcm_SumEntropy | 14.54 | 40.30 | 0.743 | 0.388 | 0.884 | 27.5 | -53.1 |
| square_glcm_SumSquares | 67.30 | 186.55 | 0.676 | 0.453 | 0.82 | 144.8 | -228.3 |
| square_gldm_DependenceEntropy | 5.53 | 15.34 | 0.841 | 0.685 | 0.92 | 12.1 | -18.6 |
| square_gldm_DependenceNonUniformity | 27.13 | 75.21 | 0.936 | 0.879 | 0.967 | 66.5 | -83.9 |
| square_gldm_DependenceNonUniformityNormalized | 19.78 | 54.82 | 0.296 | -0.1 | 0.617 | 17.5 | -92.2 |
| square_gldm_DependenceVariance | 33.13 | 91.84 | 0.393 | -0.009 | 0.667 | 162.1 | -21.6 |
| square_gldm_GrayLevelNonUniformity | 30.89 | 85.64 | 0.732 | 0.534 | 0.854 | 159.3 | -11.9 |
| square_gldm_GrayLevelVariance | 68.03 | 188.57 | 0.666 | 0.44 | 0.814 | 146.1 | -231 |
| square_gldm_HighGrayLevelEmphasis | 65.92 | 182.72 | 0.699 | 0.486 | 0.834 | 130.1 | -235.4 |
| square_gldm_LargeDependenceEmphasis | 40.73 | 112.91 | 0.439 | 0.056 | 0.692 | 187.6 | -38.2 |
| square_gldm_LargeDependenceHighGrayLevelEmphasis | 35.27 | 97.76 | 0.671 | 0.445 | 0.817 | 83.3 | -112.3 |
| square_gldm_LargeDependenceLowGrayLevelEmphasis | 65.15 | 180.59 | 0.026 | -0.181 | 0.276 | 309.5 | -51.7 |
| square_gldm_LowGrayLevelEmphasis | 49.95 | 138.45 | 0 | -0.157 | 0.21 | 219.8 | -57.1 |
| square_gldm_SmallDependenceEmphasis | 50.15 | 139.02 | 0.524 | -0.007 | 0.784 | 76 | -202.1 |
| square_gldm_SmallDependenceHighGrayLevelEmphasis | 82.67 | 229.15 | 0.693 | 0.478 | 0.83 | 160 | -298.3 |
| square_gldm_SmallDependenceLowGrayLevelEmphasis | 33.27 | 92.21 | 0.122 | -0.122 | 0.384 | 58.2 | -126.3 |
| square_glrlm_GrayLevelNonUniformity | 26.47 | 73.37 | 0.827 | 0.683 | 0.909 | 136.5 | -10.3 |
| square_glrlm_GrayLevelNonUniformityNormalized | 32.17 | 89.17 | 0.537 | 0.188 | 0.75 | 133.1 | -45.2 |
| square_glrlm_GrayLevelVariance | 68.41 | 189.63 | 0.646 | 0.41 | 0.801 | 149 | -230.3 |
| square_glrlm_HighGrayLevelRunEmphasis | 66.10 | 183.22 | 0.679 | 0.456 | 0.822 | 133.6 | -232.8 |
| square_glrlm_LongRunEmphasis | 19.94 | 55.28 | 0.394 | 0.023 | 0.658 | 84 | -26.5 |
| square_glrlm_LongRunHighGrayLevelEmphasis | 58.59 | 162.40 | 0.677 | 0.454 | 0.821 | 122.7 | -202.1 |
| square_glrlm_LongRunLowGrayLevelEmphasis | 58.62 | 162.49 | 0.054 | -0.15 | 0.298 | 266 | -59 |
| square_glrlm_LowGrayLevelRunEmphasis | 49.65 | 137.63 | 0.085 | -0.117 | 0.324 | 215.5 | -59.7 |
| square_glrlm_RunEntropy | 9.66 | 26.77 | 0.785 | 0.545 | 0.895 | 19.5 | -34.1 |
| square_glrlm_RunLengthNonUniformity | 25.54 | 70.79 | 0.988 | 0.976 | 0.994 | 79.2 | -62.4 |
| square_glrlm_RunLengthNonUniformityNormalized | 11.61 | 32.19 | 0.496 | 0.032 | 0.752 | 19.5 | -44.9 |
| square_glrlm_RunPercentage | 7.29 | 20.19 | 0.452 | -0.011 | 0.724 | 11.4 | -29 |
| square_glrlm_RunVariance | 39.47 | 109.40 | 0.346 | -0.047 | 0.634 | 187.4 | -31.4 |
| square_glrlm_ShortRunEmphasis | 6.13 | 16.99 | 0.488 | 0.085 | 0.731 | 11 | -23 |
| square_glrlm_ShortRunHighGrayLevelEmphasis | 68.67 | 190.34 | 0.679 | 0.457 | 0.822 | 138.1 | -242.6 |
| square_glrlm_ShortRunLowGrayLevelEmphasis | 47.45 | 131.51 | 0.076 | -0.118 | 0.31 | 203.1 | -59.9 |
| square_glszm_GrayLevelNonUniformity | 52.30 | 144.96 | 0.744 | 0.555 | 0.86 | 101.6 | -188.4 |
| square_glszm_GrayLevelNonUniformityNormalized | 37.57 | 104.15 | 0.639 | 0.399 | 0.797 | 115.9 | -92.4 |
| square_glszm_GrayLevelVariance | 65.67 | 182.03 | 0.59 | 0.334 | 0.767 | 147.5 | -216.6 |
| square_glszm_HighGrayLevelZoneEmphasis | 57.15 | 158.40 | 0.622 | 0.377 | 0.787 | 142.5 | -174.3 |
| square_glszm_LargeAreaEmphasis | 68.89 | 190.94 | 0.053 | -0.254 | 0.36 | 332.4 | -49.5 |
| square_glszm_LargeAreaHighGrayLevelEmphasis | 50.73 | 140.63 | 0.258 | -0.06 | 0.533 | 230.8 | -50.4 |
| square_glszm_LargeAreaLowGrayLevelEmphasis | 75.45 | 209.13 | 0 | -0.296 | 0.309 | 364.4 | -53.8 |
| square_glszm_LowGrayLevelZoneEmphasis | 52.81 | 146.37 | 0.583 | 0.321 | 0.763 | 133.9 | -158.8 |
| square_glszm_SizeZoneNonUniformity | 69.36 | 192.26 | 0.494 | 0.204 | 0.704 | 133.3 | -251.2 |
| square_glszm_SizeZoneNonUniformityNormalized | 31.12 | 86.25 | 0.67 | 0.444 | 0.816 | 70.2 | -102.3 |
| square_glszm_SmallAreaEmphasis | 53.10 | 147.18 | 0.652 | 0.375 | 0.814 | 116.5 | -177.9 |
| square_glszm_SmallAreaHighGrayLevelEmphasis | 79.40 | 220.08 | 0.618 | 0.371 | 0.784 | 187 | -253.2 |
| square_glszm_SmallAreaLowGrayLevelEmphasis | 65.53 | 181.65 | 0 | -0.295 | 0.309 | 121.8 | -241.5 |
| square_glszm_ZoneEntropy | 22.20 | 61.54 | 0.743 | 0.541 | 0.862 | 50.5 | -72.6 |
| square_glszm_ZonePercentage | 57.20 | 158.55 | 0.461 | -0.033 | 0.74 | 84.4 | -232.7 |
| square_glszm_ZoneVariance | 77.72 | 215.44 | 0.07 | -0.246 | 0.378 | 350.8 | -80.1 |
| square_ngtdm_Busyness | 62.96 | 174.52 | 0.177 | -0.124 | 0.46 | 239.9 | -109.1 |
| square_ngtdm_Coarseness | 14.48 | 40.14 | 0.855 | 0.504 | 0.943 | 18.6 | -61.7 |
| square_ngtdm_Complexity | 79.42 | 220.13 | 0.648 | 0.414 | 0.803 | 158.9 | -281.4 |
| square_ngtdm_Contrast | 41.82 | 115.91 | 0.586 | 0.193 | 0.792 | 66.2 | -165.6 |
| square_ngtdm_Strength | 69.03 | 191.35 | 0.622 | 0.376 | 0.787 | 164.6 | -218.1 |
| squareroot_firstorder_10Percentile | 25.18 | 69.80 | 0 | -0.081 | 0.128 | 111.7 | -27.9 |
| squareroot_firstorder_90Percentile | 27.35 | 75.81 | 0.132 | -0.087 | 0.391 | 127.6 | -24 |
| squareroot_firstorder_Energy | 47.61 | 131.97 | 0.335 | 0.024 | 0.591 | 239.5 | -24.5 |
| squareroot_firstorder_Entropy | 8.19 | 22.71 | 0.424 | -0.097 | 0.739 | 36.7 | -8.7 |
| squareroot_firstorder_InterquartileRange | 32.82 | 90.97 | 0.334 | -0.103 | 0.663 | 151.8 | -30.1 |
| squareroot_firstorder_Kurtosis | 7.69 | 21.32 | 0.764 | 0.53 | 0.881 | 26.8 | -15.8 |
| squareroot_firstorder_Maximum | 27.79 | 77.03 | 0.231 | -0.099 | 0.55 | 132.7 | -21.4 |
| squareroot_firstorder_MeanAbsoluteDeviation | 31.83 | 88.22 | 0.352 | -0.104 | 0.684 | 150 | -26.4 |
| squareroot_firstorder_Mean | 26.35 | 73.04 | 0 | -0.093 | 0.143 | 120.3 | -25.8 |
| squareroot_firstorder_Median | 26.26 | 72.78 | 0 | -0.09 | 0.14 | 118.4 | -27.2 |
| squareroot_firstorder_Minimum | 24.48 | 67.86 | 0 | -0.115 | 0.168 | 105.7 | -30.1 |
| squareroot_firstorder_Range | 30.81 | 85.39 | 0.362 | -0.104 | 0.694 | 148.5 | -22.3 |
| squareroot_firstorder_RobustMeanAbsoluteDeviation | 32.22 | 89.31 | 0.342 | -0.103 | 0.672 | 150.7 | -27.9 |
| squareroot_firstorder_RootMeanSquared | 26.50 | 73.46 | 0.021 | -0.081 | 0.173 | 121.8 | -25.2 |
| squareroot_firstorder_Skewness | 1352.19 | 3748.07 | 0.788 | 0.243 | 0.922 | 4084 | -3412.2 |
| squareroot_firstorder_TotalEnergy | 47.61 | 131.97 | 0.335 | 0.024 | 0.591 | 239.5 | -24.5 |
| squareroot_firstorder_Uniformity | 29.73 | 82.40 | 0.491 | 0.076 | 0.736 | 27.2 | -137.6 |
| squareroot_firstorder_Variance | 53.21 | 147.48 | 0.283 | -0.077 | 0.58 | 255 | -39.9 |
| squareroot_glcm_Autocorrelation | 50.80 | 140.82 | 0.225 | -0.097 | 0.531 | 240.8 | -40.8 |
| squareroot_glcm_ClusterProminence | 70.87 | 196.45 | 0.098 | -0.177 | 0.381 | 348.2 | -44.7 |
| squareroot_glcm_ClusterShade | 65.71 | 182.13 | 0.16 | -0.123 | 0.438 | 341.7 | -22.5 |
| squareroot_glcm_ClusterTendency | 53.19 | 147.45 | 0.291 | -0.066 | 0.583 | 257.6 | -37.3 |
| squareroot_glcm_Contrast | 52.97 | 146.83 | 0.234 | -0.092 | 0.533 | 242.7 | -51 |
| squareroot_glcm_Correlation | 5.28 | 14.63 | 0.818 | 0.182 | 0.939 | 21.8 | -7.5 |
| squareroot_glcm_DifferenceAverage | 31.62 | 87.64 | 0.275 | -0.1 | 0.594 | 141.5 | -33.8 |
| squareroot_glcm_DifferenceEntropy | 10.52 | 29.15 | 0.337 | -0.097 | 0.657 | 46.2 | -12.1 |
| squareroot_glcm_DifferenceVariance | 52.39 | 145.22 | 0.234 | -0.091 | 0.532 | 242.6 | -47.8 |
| squareroot_glcm_Id | 22.14 | 61.38 | 0.35 | -0.047 | 0.639 | 24.9 | -97.9 |
| squareroot_glcm_Idm | 30.53 | 84.62 | 0.359 | -0.007 | 0.631 | 35.6 | -133.6 |
| squareroot_glcm_Idmn | 0.32 | 0.89 | 0.822 | 0.391 | 0.932 | 1.3 | -0.5 |
| squareroot_glcm_Idn | 0.72 | 2.00 | 0.845 | 0.376 | 0.944 | 2.9 | -1.1 |
| squareroot_glcm_Imc1 | 18.71 | 51.87 | 0.482 | 0.056 | 0.733 | 74.1 | -29.6 |
| squareroot_glcm_Imc2 | 2.25 | 6.23 | 0.335 | 0.032 | 0.588 | 7.8 | -4.7 |
| squareroot_glcm_InverseVariance | 29.06 | 80.55 | 0.389 | -0.007 | 0.663 | 31.1 | -130 |
| squareroot_glcm_JointAverage | 29.79 | 82.58 | 0.263 | -0.102 | 0.591 | 137.8 | -27.3 |
| squareroot_glcm_JointEnergy | 31.91 | 88.44 | 0.666 | 0.315 | 0.836 | 27.1 | -149.8 |
| squareroot_glcm_JointEntropy | 5.19 | 14.39 | 0.699 | -0.016 | 0.897 | 23.5 | -5.3 |
| squareroot_glcm_MCC | 5.23 | 14.51 | 0.493 | 0.065 | 0.74 | 20.4 | -8.6 |
| squareroot_glcm_MaximumProbability | 31.57 | 87.52 | 0.602 | 0.26 | 0.794 | 31.3 | -143.8 |
| squareroot_glcm_SumAverage | 29.79 | 82.58 | 0.263 | -0.102 | 0.591 | 137.8 | -27.3 |
| squareroot_glcm_SumEntropy | 6.63 | 18.39 | 0.512 | -0.097 | 0.809 | 31.1 | -5.7 |
| squareroot_glcm_SumSquares | 53.14 | 147.29 | 0.289 | -0.071 | 0.583 | 255.8 | -38.8 |
| squareroot_gldm_DependenceEntropy | 3.20 | 8.87 | 0.703 | -0.074 | 0.911 | 16.2 | -1.6 |
| squareroot_gldm_DependenceNonUniformity | 23.92 | 66.30 | 0.738 | 0.544 | 0.857 | 121.2 | -11.4 |
| squareroot_gldm_DependenceNonUniformityNormalized | 20.97 | 58.12 | 0.265 | -0.073 | 0.555 | 85.6 | -30.6 |
| squareroot_gldm_DependenceVariance | 40.40 | 111.99 | 0.259 | -0.035 | 0.524 | 62.9 | -161.1 |
| squareroot_gldm_GrayLevelNonUniformity | 33.40 | 92.57 | 0.724 | 0.525 | 0.848 | 64.9 | -120.2 |
| squareroot_gldm_GrayLevelVariance | 53.24 | 147.58 | 0.283 | -0.077 | 0.58 | 255 | -40.2 |
| squareroot_gldm_HighGrayLevelEmphasis | 50.82 | 140.85 | 0.224 | -0.096 | 0.527 | 241.1 | -40.6 |
| squareroot_gldm_LargeDependenceEmphasis | 31.98 | 88.65 | 0.25 | -0.041 | 0.516 | 51.1 | -126.2 |
| squareroot_gldm_LargeDependenceHighGrayLevelEmphasis | 31.50 | 87.32 | 0.445 | -0.092 | 0.752 | 157.4 | -17.2 |
| squareroot_gldm_LargeDependenceLowGrayLevelEmphasis | 54.52 | 151.12 | 0.322 | 0.018 | 0.578 | 55.1 | -247.2 |
| squareroot_gldm_LowGrayLevelEmphasis | 36.36 | 100.78 | 0.617 | 0.184 | 0.819 | 25.7 | -175.9 |
| squareroot_gldm_SmallDependenceEmphasis | 19.12 | 53.00 | 0.276 | -0.053 | 0.555 | 72.5 | -33.5 |
| squareroot_gldm_SmallDependenceHighGrayLevelEmphasis | 59.89 | 166.00 | 0.216 | -0.093 | 0.511 | 278.8 | -53.2 |
| squareroot_gldm_SmallDependenceLowGrayLevelEmphasis | 32.26 | 89.43 | 0.655 | 0.244 | 0.838 | 25.6 | -153.3 |
| squareroot_glrlm_GrayLevelNonUniformity | 32.47 | 90.01 | 0.746 | 0.559 | 0.862 | 63.4 | -116.6 |
| squareroot_glrlm_GrayLevelNonUniformityNormalized | 29.73 | 82.40 | 0.491 | 0.075 | 0.736 | 27.2 | -137.6 |
| squareroot_glrlm_GrayLevelVariance | 53.34 | 147.86 | 0.283 | -0.077 | 0.58 | 255.3 | -40.4 |
| squareroot_glrlm_HighGrayLevelRunEmphasis | 50.92 | 141.15 | 0.224 | -0.096 | 0.527 | 241.6 | -40.7 |
| squareroot_glrlm_LongRunEmphasis | 4.04 | 11.20 | 0.264 | -0.032 | 0.529 | 7.5 | -14.9 |
| squareroot_glrlm_LongRunHighGrayLevelEmphasis | 48.89 | 135.52 | 0.228 | -0.097 | 0.533 | 233.4 | -37.7 |
| squareroot_glrlm_LongRunLowGrayLevelEmphasis | 37.91 | 105.07 | 0.598 | 0.2 | 0.801 | 27.8 | -182.3 |
| squareroot_glrlm_LowGrayLevelRunEmphasis | 36.47 | 101.09 | 0.621 | 0.192 | 0.821 | 25.7 | -176.5 |
| squareroot_glrlm_RunEntropy | 7.02 | 19.46 | 0.45 | -0.099 | 0.763 | 32.3 | -6.6 |
| squareroot_glrlm_RunLengthNonUniformity | 15.81 | 43.83 | 0.963 | 0.926 | 0.981 | 76 | -11.6 |
| squareroot_glrlm_RunLengthNonUniformityNormalized | 2.33 | 6.47 | 0.304 | -0.014 | 0.571 | 8.7 | -4.3 |
| squareroot_glrlm_RunPercentage | 1.28 | 3.54 | 0.289 | -0.02 | 0.555 | 4.7 | -2.4 |
| squareroot_glrlm_RunVariance | 39.17 | 108.57 | 0.242 | -0.047 | 0.508 | 58.1 | -159 |
| squareroot_glrlm_ShortRunEmphasis | 0.94 | 2.59 | 0.301 | -0.012 | 0.566 | 3.5 | -1.7 |
| squareroot_glrlm_ShortRunHighGrayLevelEmphasis | 51.43 | 142.57 | 0.223 | -0.096 | 0.526 | 243.7 | -41.5 |
| squareroot_glrlm_ShortRunLowGrayLevelEmphasis | 36.14 | 100.17 | 0.626 | 0.19 | 0.825 | 25.2 | -175.2 |
| squareroot_glszm_GrayLevelNonUniformity | 22.50 | 62.38 | 0.936 | 0.878 | 0.967 | 48.1 | -76.7 |
| squareroot_glszm_GrayLevelNonUniformityNormalized | 30.68 | 85.05 | 0.491 | 0.089 | 0.732 | 28 | -142.1 |
| squareroot_glszm_GrayLevelVariance | 53.84 | 149.23 | 0.28 | -0.076 | 0.576 | 257.3 | -41.1 |
| squareroot_glszm_HighGrayLevelZoneEmphasis | 52.32 | 145.01 | 0.228 | -0.096 | 0.533 | 248.3 | -41.8 |
| squareroot_glszm_LargeAreaEmphasis | 46.38 | 128.55 | 0.045 | -0.265 | 0.355 | 79.6 | -177.5 |
| squareroot_glszm_LargeAreaHighGrayLevelEmphasis | 40.12 | 111.22 | 0.435 | 0.131 | 0.665 | 164.3 | -58.2 |
| squareroot_glszm_LargeAreaLowGrayLevelEmphasis | 55.23 | 153.10 | 0.221 | -0.106 | 0.507 | 53.2 | -253 |
| squareroot_glszm_LowGrayLevelZoneEmphasis | 39.09 | 108.35 | 0.625 | 0.296 | 0.806 | 29.2 | -187.5 |
| squareroot_glszm_SizeZoneNonUniformity | 32.33 | 89.62 | 0.633 | 0.392 | 0.794 | 150.4 | -28.8 |
| squareroot_glszm_SizeZoneNonUniformityNormalized | 17.10 | 47.39 | 0.247 | -0.064 | 0.526 | 65.9 | -28.9 |
| squareroot_glszm_SmallAreaEmphasis | 8.66 | 24.01 | 0.269 | -0.045 | 0.542 | 32.7 | -15.3 |
| squareroot_glszm_SmallAreaHighGrayLevelEmphasis | 56.91 | 157.75 | 0.224 | -0.094 | 0.524 | 266.8 | -48.6 |
| squareroot_glszm_SmallAreaLowGrayLevelEmphasis | 38.00 | 105.33 | 0.621 | 0.305 | 0.801 | 32 | -178.7 |
| squareroot_glszm_ZoneEntropy | 4.05 | 11.23 | 0.619 | -0.087 | 0.874 | 20.2 | -2.3 |
| squareroot_glszm_ZonePercentage | 17.13 | 47.48 | 0.292 | -0.038 | 0.568 | 63.6 | -31.4 |
| squareroot_glszm_ZoneVariance | 60.60 | 167.96 | 0.03 | -0.283 | 0.344 | 88.1 | -247.8 |
| squareroot_ngtdm_Busyness | 49.38 | 136.89 | 0.257 | -0.045 | 0.526 | 51.2 | -222.6 |
| squareroot_ngtdm_Coarseness | 13.93 | 38.62 | 0.831 | 0.416 | 0.935 | 18 | -59.2 |
| squareroot_ngtdm_Complexity | 63.06 | 174.80 | 0.241 | -0.065 | 0.518 | 302.1 | -47.5 |
| squareroot_ngtdm_Contrast | 38.01 | 105.35 | 0.19 | -0.079 | 0.458 | 154.4 | -56.3 |
| squareroot_ngtdm_Strength | 50.00 | 138.61 | 0.272 | -0.07 | 0.561 | 238.8 | -38.4 |
| wavelet-LLH_firstorder_10Percentile | 16.36 | 45.36 | 0.795 | 0.567 | 0.9 | 58.9 | -31.8 |
| wavelet-LLH_firstorder_90Percentile | 50.70 | 140.53 | 0.755 | 0.272 | 0.901 | 156.7 | -124.3 |
| wavelet-LLH_firstorder_Energy | 31.66 | 87.77 | 0.7 | 0.464 | 0.839 | 149.2 | -26.3 |
| wavelet-LLH_firstorder_Entropy | 6.99 | 19.39 | 0.862 | 0.419 | 0.951 | 26.7 | -12.1 |
| wavelet-LLH_firstorder_InterquartileRange | 16.94 | 46.94 | 0.837 | 0.641 | 0.922 | 58.7 | -35.2 |
| wavelet-LLH_firstorder_Kurtosis | 14.70 | 40.74 | 0.639 | 0.287 | 0.819 | 56.8 | -24.7 |
| wavelet-LLH_firstorder_Maximum | 16.99 | 47.10 | 0.706 | 0.173 | 0.881 | 81.1 | -13.1 |
| wavelet-LLH_firstorder_MeanAbsoluteDeviation | 15.38 | 42.63 | 0.815 | 0.435 | 0.925 | 60.1 | -25.2 |
| wavelet-LLH_firstorder_Mean | 153.95 | 426.72 | 0.289 | -0.028 | 0.557 | 386.7 | -466.7 |
| wavelet-LLH_firstorder_Median | 326.99 | 906.36 | 0.221 | -0.078 | 0.496 | 959.5 | -853.2 |
| wavelet-LLH_firstorder_Minimum | 16.72 | 46.34 | 0.687 | 0.245 | 0.861 | 73.8 | -18.9 |
| wavelet-LLH_firstorder_Range | 15.85 | 43.92 | 0.707 | 0.197 | 0.879 | 73.8 | -14 |
| wavelet-LLH_firstorder_RobustMeanAbsoluteDeviation | 16.49 | 45.71 | 0.845 | 0.631 | 0.929 | 58.1 | -33.3 |
| wavelet-LLH_firstorder_RootMeanSquared | 14.08 | 39.04 | 0.795 | 0.39 | 0.917 | 57 | -21.1 |
| wavelet-LLH_firstorder_Skewness | 132.33 | 366.80 | 0.585 | 0.248 | 0.781 | 386.5 | -347.1 |
| wavelet-LLH_firstorder_TotalEnergy | 31.66 | 87.77 | 0.7 | 0.464 | 0.839 | 149.2 | -26.3 |
| wavelet-LLH_firstorder_Uniformity | 15.63 | 43.32 | 0.816 | 0.662 | 0.903 | 28.4 | -58.2 |
| wavelet-LLH_firstorder_Variance | 28.50 | 79.00 | 0.662 | 0.306 | 0.834 | 119.6 | -38.4 |
| wavelet-LLH_glcm_Autocorrelation | 29.17 | 80.87 | 0.464 | 0.143 | 0.692 | 133.7 | -28.1 |
| wavelet-LLH_glcm_ClusterProminence | 49.61 | 137.50 | 0.268 | -0.03 | 0.533 | 222.7 | -52.3 |
| wavelet-LLH_glcm_ClusterShade | 235.42 | 652.55 | 0.247 | -0.046 | 0.515 | 779.3 | -525.8 |
| wavelet-LLH_glcm_ClusterTendency | 31.00 | 85.92 | 0.649 | 0.297 | 0.826 | 126.7 | -45.1 |
| wavelet-LLH_glcm_Contrast | 26.70 | 74.00 | 0.729 | 0.435 | 0.868 | 110.2 | -37.8 |
| wavelet-LLH_glcm_Correlation | 5.90 | 16.36 | 0.658 | 0.427 | 0.809 | 17.1 | -15.6 |
| wavelet-LLH_glcm_DifferenceAverage | 15.07 | 41.78 | 0.836 | 0.509 | 0.932 | 59 | -24.6 |
| wavelet-LLH_glcm_DifferenceEntropy | 8.21 | 22.77 | 0.843 | 0.392 | 0.943 | 32.5 | -13 |
| wavelet-LLH_glcm_DifferenceVariance | 27.46 | 76.13 | 0.653 | 0.314 | 0.826 | 116.3 | -36 |
| wavelet-LLH_glcm_Id | 6.45 | 17.89 | 0.865 | 0.624 | 0.942 | 11.4 | -24.4 |
| wavelet-LLH_glcm_Idm | 8.76 | 24.27 | 0.867 | 0.632 | 0.943 | 15.5 | -33.1 |
| wavelet-LLH_glcm_Idmn | 0.26 | 0.73 | 0.799 | 0.46 | 0.913 | 1 | -0.5 |
| wavelet-LLH_glcm_Idn | 0.82 | 2.29 | 0.792 | 0.509 | 0.905 | 3 | -1.5 |
| wavelet-LLH_glcm_Imc1 | 6.53 | 18.09 | 0.741 | 0.537 | 0.861 | 14.9 | -21.3 |
| wavelet-LLH_glcm_Imc2 | 3.16 | 8.76 | 0.824 | 0.684 | 0.906 | 9.5 | -8 |
| wavelet-LLH_glcm_InverseVariance | 7.42 | 20.57 | 0.806 | 0.557 | 0.909 | 14.5 | -26.6 |
| wavelet-LLH_glcm_JointAverage | 16.04 | 44.47 | 0.68 | 0.232 | 0.858 | 72.2 | -16.7 |
| wavelet-LLH_glcm_JointEnergy | 27.34 | 75.79 | 0.748 | 0.561 | 0.863 | 47.1 | -104.5 |
| wavelet-LLH_glcm_JointEntropy | 7.25 | 20.09 | 0.86 | 0.333 | 0.953 | 27.9 | -12.2 |
| wavelet-LLH_glcm_MCC | 4.08 | 11.30 | 0.732 | 0.537 | 0.854 | 11.9 | -10.7 |
| wavelet-LLH_glcm_MaximumProbability | 27.64 | 76.62 | 0.726 | 0.529 | 0.85 | 60.3 | -93 |
| wavelet-LLH_glcm_SumAverage | 16.04 | 44.47 | 0.68 | 0.232 | 0.858 | 72.2 | -16.7 |
| wavelet-LLH_glcm_SumEntropy | 6.28 | 17.41 | 0.855 | 0.399 | 0.948 | 23.8 | -11 |
| wavelet-LLH_glcm_SumSquares | 30.02 | 83.21 | 0.659 | 0.31 | 0.832 | 123.6 | -42.8 |
| wavelet-LLH_gldm_DependenceEntropy | 2.52 | 6.99 | 0.874 | 0.435 | 0.956 | 10.2 | -3.8 |
| wavelet-LLH_gldm_DependenceNonUniformity | 16.67 | 46.20 | 0.929 | 0.86 | 0.964 | 80.2 | -12.2 |
| wavelet-LLH_gldm_DependenceNonUniformityNormalized | 12.72 | 35.26 | 0.878 | 0.774 | 0.936 | 40.5 | -30.1 |
| wavelet-LLH_gldm_DependenceVariance | 28.76 | 79.71 | 0.721 | 0.518 | 0.847 | 68.3 | -91.2 |
| wavelet-LLH_gldm_GrayLevelNonUniformity | 19.96 | 55.34 | 0.986 | 0.974 | 0.993 | 69.6 | -41.1 |
| wavelet-LLH_gldm_GrayLevelVariance | 28.70 | 79.56 | 0.663 | 0.307 | 0.835 | 119.7 | -39.4 |
| wavelet-LLH_gldm_HighGrayLevelEmphasis | 28.78 | 79.76 | 0.474 | 0.151 | 0.7 | 131.8 | -27.8 |
| wavelet-LLH_gldm_LargeDependenceEmphasis | 21.24 | 58.87 | 0.826 | 0.674 | 0.909 | 45 | -72.7 |
| wavelet-LLH_gldm_LargeDependenceHighGrayLevelEmphasis | 31.28 | 86.71 | 0.507 | 0.213 | 0.715 | 124 | -49.4 |
| wavelet-LLH_gldm_LargeDependenceLowGrayLevelEmphasis | 42.09 | 116.67 | 0.924 | 0.842 | 0.962 | 57.3 | -176.1 |
| wavelet-LLH_gldm_LowGrayLevelEmphasis | 27.90 | 77.34 | 0.925 | 0.734 | 0.97 | 32.8 | -121.9 |
| wavelet-LLH_gldm_SmallDependenceEmphasis | 19.26 | 53.40 | 0.861 | 0.684 | 0.934 | 70.7 | -36.1 |
| wavelet-LLH_gldm_SmallDependenceHighGrayLevelEmphasis | 36.42 | 100.95 | 0.437 | 0.131 | 0.668 | 168.5 | -33.3 |
| wavelet-LLH_gldm_SmallDependenceLowGrayLevelEmphasis | 27.60 | 76.51 | 0.592 | 0.241 | 0.789 | 46.9 | -106.2 |
| wavelet-LLH_glrlm_GrayLevelNonUniformity | 16.55 | 45.89 | 0.999 | 0.999 | 1 | 61.1 | -30.7 |
| wavelet-LLH_glrlm_GrayLevelNonUniformityNormalized | 14.25 | 39.50 | 0.852 | 0.693 | 0.927 | 23.3 | -55.7 |
| wavelet-LLH_glrlm_GrayLevelVariance | 27.02 | 74.89 | 0.652 | 0.287 | 0.829 | 116.7 | -33.1 |
| wavelet-LLH_glrlm_HighGrayLevelRunEmphasis | 28.70 | 79.55 | 0.474 | 0.15 | 0.7 | 131.7 | -27.4 |
| wavelet-LLH_glrlm_LongRunEmphasis | 10.73 | 29.74 | 0.833 | 0.678 | 0.914 | 22.6 | -36.9 |
| wavelet-LLH_glrlm_LongRunHighGrayLevelEmphasis | 27.44 | 76.07 | 0.466 | 0.15 | 0.692 | 120.7 | -31.5 |
| wavelet-LLH_glrlm_LongRunLowGrayLevelEmphasis | 31.49 | 87.28 | 0.947 | 0.861 | 0.976 | 35.9 | -138.7 |
| wavelet-LLH_glrlm_LowGrayLevelRunEmphasis | 27.30 | 75.66 | 0.938 | 0.722 | 0.978 | 31.4 | -119.9 |
| wavelet-LLH_glrlm_RunEntropy | 3.46 | 9.60 | 0.844 | 0.21 | 0.95 | 14.9 | -4.3 |
| wavelet-LLH_glrlm_RunLengthNonUniformity | 15.63 | 43.34 | 0.927 | 0.858 | 0.962 | 77.2 | -9.5 |
| wavelet-LLH_glrlm_RunLengthNonUniformityNormalized | 5.20 | 14.40 | 0.873 | 0.72 | 0.939 | 17.9 | -10.9 |
| wavelet-LLH_glrlm_RunPercentage | 3.41 | 9.46 | 0.866 | 0.723 | 0.934 | 11.6 | -7.4 |
| wavelet-LLH_glrlm_RunVariance | 19.49 | 54.02 | 0.809 | 0.645 | 0.9 | 41.9 | -66.1 |
| wavelet-LLH_glrlm_ShortRunEmphasis | 2.90 | 8.04 | 0.861 | 0.71 | 0.932 | 9.9 | -6.2 |
| wavelet-LLH_glrlm_ShortRunHighGrayLevelEmphasis | 29.35 | 81.36 | 0.477 | 0.153 | 0.702 | 135.2 | -27.5 |
| wavelet-LLH_glrlm_ShortRunLowGrayLevelEmphasis | 26.57 | 73.66 | 0.936 | 0.628 | 0.979 | 31.6 | -115.7 |
| wavelet-LLH_glszm_GrayLevelNonUniformity | 17.16 | 47.56 | 0.902 | 0.814 | 0.949 | 65.8 | -29.3 |
| wavelet-LLH_glszm_GrayLevelNonUniformityNormalized | 13.77 | 38.16 | 0.841 | 0.283 | 0.946 | 10.2 | -66.1 |
| wavelet-LLH_glszm_GrayLevelVariance | 24.56 | 68.07 | 0.591 | 0.19 | 0.797 | 119.1 | -17.1 |
| wavelet-LLH_glszm_HighGrayLevelZoneEmphasis | 27.38 | 75.91 | 0.472 | 0.151 | 0.697 | 127.9 | -24 |
| wavelet-LLH_glszm_LargeAreaEmphasis | 47.77 | 132.41 | 0.697 | 0.484 | 0.832 | 117.1 | -147.7 |
| wavelet-LLH_glszm_LargeAreaHighGrayLevelEmphasis | 44.66 | 123.80 | 0.844 | 0.716 | 0.917 | 157.7 | -89.9 |
| wavelet-LLH_glszm_LargeAreaLowGrayLevelEmphasis | 50.65 | 140.38 | 0.734 | 0.532 | 0.856 | 76.5 | -204.2 |
| wavelet-LLH_glszm_LowGrayLevelZoneEmphasis | 25.89 | 71.75 | 0.911 | 0.627 | 0.968 | 27.9 | -115.6 |
| wavelet-LLH_glszm_SizeZoneNonUniformity | 36.97 | 102.47 | 0.645 | 0.374 | 0.808 | 158.4 | -46.6 |
| wavelet-LLH_glszm_SizeZoneNonUniformityNormalized | 20.09 | 55.68 | 0.659 | 0.397 | 0.816 | 67.7 | -43.7 |
| wavelet-LLH_glszm_SmallAreaEmphasis | 33.17 | 91.94 | 0.672 | 0.41 | 0.825 | 106 | -77.9 |
| wavelet-LLH_glszm_SmallAreaHighGrayLevelEmphasis | 44.60 | 123.64 | 0.421 | 0.113 | 0.656 | 186.5 | -60.8 |
| wavelet-LLH_glszm_SmallAreaLowGrayLevelEmphasis | 53.53 | 148.37 | 0.135 | -0.161 | 0.424 | 111.2 | -185.5 |
| wavelet-LLH_glszm_ZoneEntropy | 4.46 | 12.37 | 0.899 | 0.164 | 0.972 | 20.7 | -4.1 |
| wavelet-LLH_glszm_ZonePercentage | 24.42 | 67.69 | 0.861 | 0.727 | 0.929 | 84.8 | -50.6 |
| wavelet-LLH_glszm_ZoneVariance | 52.89 | 146.59 | 0.698 | 0.486 | 0.833 | 138.9 | -154.3 |
| wavelet-LLH_ngtdm_Busyness | 30.31 | 84.02 | 0.875 | 0.715 | 0.941 | 41.8 | -126.3 |
| wavelet-LLH_ngtdm_Coarseness | 12.04 | 33.36 | 0.854 | 0.467 | 0.945 | 12.4 | -54.3 |
| wavelet-LLH_ngtdm_Complexity | 33.71 | 93.45 | 0.503 | 0.178 | 0.721 | 157.7 | -29.2 |
| wavelet-LLH_ngtdm_Contrast | 24.53 | 68.00 | 0.84 | 0.71 | 0.915 | 65.5 | -70.5 |
| wavelet-LLH_ngtdm_Strength | 29.46 | 81.65 | 0.669 | 0.418 | 0.821 | 118.7 | -44.6 |
| wavelet-LHL_firstorder_10Percentile | 19.63 | 54.41 | 0.867 | 0.616 | 0.944 | 70.1 | -38.7 |
| wavelet-LHL_firstorder_90Percentile | 24.83 | 68.82 | 0.822 | 0.312 | 0.936 | 98 | -39.6 |
| wavelet-LHL_firstorder_Energy | 36.86 | 102.17 | 0.529 | 0.251 | 0.728 | 167.4 | -37 |
| wavelet-LHL_firstorder_Entropy | 8.82 | 24.44 | 0.872 | 0.482 | 0.953 | 33.1 | -15.8 |
| wavelet-LHL_firstorder_InterquartileRange | 19.05 | 52.80 | 0.873 | 0.645 | 0.946 | 68.5 | -37.1 |
| wavelet-LHL_firstorder_Kurtosis | 12.96 | 35.91 | 0.767 | 0.42 | 0.897 | 52.1 | -19.7 |
| wavelet-LHL_firstorder_Maximum | 18.50 | 51.29 | 0.759 | 0.247 | 0.906 | 84.5 | -18.1 |
| wavelet-LHL_firstorder_MeanAbsoluteDeviation | 18.04 | 50.01 | 0.856 | 0.43 | 0.948 | 71.1 | -28.9 |
| wavelet-LHL_firstorder_Mean | 47.01 | 130.31 | 0.742 | 0.552 | 0.859 | 118.1 | -142.5 |
| wavelet-LHL_firstorder_Median | 519.02 | 1438.66 | 0.607 | 0.31 | 0.788 | 1262.4 | -1614.9 |
| wavelet-LHL_firstorder_Minimum | 19.08 | 52.88 | 0.743 | 0.34 | 0.888 | 84.4 | -21.3 |
| wavelet-LHL_firstorder_Range | 18.15 | 50.31 | 0.751 | 0.31 | 0.896 | 82.4 | -18.2 |
| wavelet-LHL_firstorder_RobustMeanAbsoluteDeviation | 18.53 | 51.37 | 0.874 | 0.612 | 0.948 | 68.3 | -34.5 |
| wavelet-LHL_firstorder_RootMeanSquared | 18.19 | 50.43 | 0.837 | 0.428 | 0.938 | 70.9 | -29.9 |
| wavelet-LHL_firstorder_Skewness | 65.22 | 180.79 | 0.807 | 0.257 | 0.931 | 228.9 | -132.7 |
| wavelet-LHL_firstorder_TotalEnergy | 36.86 | 102.17 | 0.529 | 0.251 | 0.728 | 167.4 | -37 |
| wavelet-LHL_firstorder_Uniformity | 17.55 | 48.65 | 0.837 | 0.674 | 0.918 | 30.8 | -66.5 |
| wavelet-LHL_firstorder_Variance | 33.79 | 93.66 | 0.705 | 0.406 | 0.853 | 139.2 | -48.1 |
| wavelet-LHL_glcm_Autocorrelation | 33.33 | 92.38 | 0.482 | 0.188 | 0.697 | 156.3 | -28.5 |
| wavelet-LHL_glcm_ClusterProminence | 54.00 | 149.69 | 0.247 | -0.067 | 0.523 | 242.8 | -56.6 |
| wavelet-LHL_glcm_ClusterShade | 161.57 | 447.84 | 0.358 | 0.054 | 0.606 | 538.1 | -357.6 |
| wavelet-LHL_glcm_ClusterTendency | 32.68 | 90.58 | 0.701 | 0.403 | 0.85 | 138.6 | -42.6 |
| wavelet-LHL_glcm_Contrast | 31.92 | 88.49 | 0.753 | 0.488 | 0.878 | 129 | -48 |
| wavelet-LHL_glcm_Correlation | 3.89 | 10.78 | 0.836 | 0.644 | 0.921 | 13.2 | -8.3 |
| wavelet-LHL_glcm_DifferenceAverage | 19.05 | 52.81 | 0.878 | 0.553 | 0.953 | 72.2 | -33.4 |
| wavelet-LHL_glcm_DifferenceEntropy | 10.23 | 28.35 | 0.874 | 0.445 | 0.955 | 39.5 | -17.2 |
| wavelet-LHL_glcm_DifferenceVariance | 29.78 | 82.53 | 0.661 | 0.39 | 0.819 | 128.2 | -36.9 |
| wavelet-LHL_glcm_Id | 7.90 | 21.90 | 0.865 | 0.693 | 0.936 | 15.1 | -28.7 |
| wavelet-LHL_glcm_Idm | 10.62 | 29.43 | 0.864 | 0.7 | 0.935 | 20.4 | -38.4 |
| wavelet-LHL_glcm_Idmn | 0.40 | 1.10 | 0.746 | 0.482 | 0.874 | 1.4 | -0.8 |
| wavelet-LHL_glcm_Idn | 1.00 | 2.78 | 0.778 | 0.489 | 0.897 | 3.7 | -1.9 |
| wavelet-LHL_glcm_Imc1 | 5.66 | 15.70 | 0.794 | 0.635 | 0.889 | 14.8 | -16.6 |
| wavelet-LHL_glcm_Imc2 | 2.75 | 7.62 | 0.888 | 0.772 | 0.944 | 9 | -6.3 |
| wavelet-LHL_glcm_InverseVariance | 7.37 | 20.43 | 0.874 | 0.715 | 0.941 | 14.4 | -26.5 |
| wavelet-LHL_glcm_JointAverage | 18.24 | 50.57 | 0.725 | 0.307 | 0.88 | 85 | -16.1 |
| wavelet-LHL_glcm_JointEnergy | 29.99 | 83.14 | 0.79 | 0.624 | 0.887 | 51.8 | -114.4 |
| wavelet-LHL_glcm_JointEntropy | 8.93 | 24.76 | 0.873 | 0.444 | 0.955 | 33.9 | -15.6 |
| wavelet-LHL_glcm_MCC | 2.94 | 8.14 | 0.825 | 0.647 | 0.912 | 9.9 | -6.4 |
| wavelet-LHL_glcm_MaximumProbability | 29.34 | 81.32 | 0.769 | 0.593 | 0.875 | 63.7 | -98.9 |
| wavelet-LHL_glcm_SumAverage | 18.24 | 50.57 | 0.725 | 0.307 | 0.88 | 85 | -16.1 |
| wavelet-LHL_glcm_SumEntropy | 7.02 | 19.46 | 0.875 | 0.426 | 0.957 | 27.1 | -11.9 |
| wavelet-LHL_glcm_SumSquares | 32.43 | 89.88 | 0.707 | 0.411 | 0.854 | 136.9 | -42.8 |
| wavelet-LHL_gldm_DependenceEntropy | 2.29 | 6.34 | 0.869 | 0.049 | 0.964 | 10.8 | -1.9 |
| wavelet-LHL_gldm_DependenceNonUniformity | 17.89 | 49.59 | 0.928 | 0.854 | 0.964 | 79.9 | -19.3 |
| wavelet-LHL_gldm_DependenceNonUniformityNormalized | 14.74 | 40.87 | 0.893 | 0.801 | 0.944 | 42.4 | -39.4 |
| wavelet-LHL_gldm_DependenceVariance | 30.80 | 85.36 | 0.791 | 0.63 | 0.887 | 83 | -87.7 |
| wavelet-LHL_gldm_GrayLevelNonUniformity | 24.90 | 69.01 | 0.979 | 0.959 | 0.989 | 80.2 | -57.8 |
| wavelet-LHL_gldm_GrayLevelVariance | 32.27 | 89.45 | 0.705 | 0.405 | 0.853 | 135.4 | -43.5 |
| wavelet-LHL_gldm_HighGrayLevelEmphasis | 33.11 | 91.78 | 0.491 | 0.198 | 0.704 | 154.3 | -29.3 |
| wavelet-LHL_gldm_LargeDependenceEmphasis | 26.75 | 74.14 | 0.799 | 0.634 | 0.893 | 62.3 | -86 |
| wavelet-LHL_gldm_LargeDependenceHighGrayLevelEmphasis | 27.72 | 76.84 | 0.536 | 0.24 | 0.737 | 129.2 | -24.5 |
| wavelet-LHL_gldm_LargeDependenceLowGrayLevelEmphasis | 47.23 | 130.90 | 0.597 | 0.341 | 0.771 | 46 | -215.8 |
| wavelet-LHL_gldm_LowGrayLevelEmphasis | 31.58 | 87.54 | 0.671 | 0.447 | 0.817 | 20.3 | -154.7 |
| wavelet-LHL_gldm_SmallDependenceEmphasis | 24.69 | 68.42 | 0.898 | 0.727 | 0.955 | 86.8 | -50 |
| wavelet-LHL_gldm_SmallDependenceHighGrayLevelEmphasis | 46.78 | 129.67 | 0.436 | 0.141 | 0.663 | 202.5 | -56.8 |
| wavelet-LHL_gldm_SmallDependenceLowGrayLevelEmphasis | 22.25 | 61.67 | 0.781 | 0.544 | 0.892 | 40.1 | -83.2 |
| wavelet-LHL_glrlm_GrayLevelNonUniformity | 20.51 | 56.84 | 0.999 | 0.998 | 0.999 | 69.8 | -43.9 |
| wavelet-LHL_glrlm_GrayLevelNonUniformityNormalized | 16.82 | 46.61 | 0.846 | 0.686 | 0.923 | 27.9 | -65.4 |
| wavelet-LHL_glrlm_GrayLevelVariance | 31.88 | 88.36 | 0.694 | 0.392 | 0.846 | 135.3 | -41.4 |
| wavelet-LHL_glrlm_HighGrayLevelRunEmphasis | 33.10 | 91.75 | 0.49 | 0.197 | 0.703 | 154.2 | -29.3 |
| wavelet-LHL_glrlm_LongRunEmphasis | 13.59 | 37.67 | 0.791 | 0.606 | 0.892 | 29.5 | -45.8 |
| wavelet-LHL_glrlm_LongRunHighGrayLevelEmphasis | 29.23 | 81.02 | 0.493 | 0.197 | 0.706 | 136.4 | -25.7 |
| wavelet-LHL_glrlm_LongRunLowGrayLevelEmphasis | 36.38 | 100.85 | 0.647 | 0.411 | 0.802 | 26.1 | -175.6 |
| wavelet-LHL_glrlm_LowGrayLevelRunEmphasis | 31.15 | 86.35 | 0.696 | 0.483 | 0.832 | 20.4 | -152.3 |
| wavelet-LHL_glrlm_RunEntropy | 3.79 | 10.50 | 0.873 | 0.224 | 0.961 | 16.3 | -4.7 |
| wavelet-LHL_glrlm_RunLengthNonUniformity | 16.21 | 44.93 | 0.898 | 0.805 | 0.947 | 80 | -9.9 |
| wavelet-LHL_glrlm_RunLengthNonUniformityNormalized | 7.53 | 20.86 | 0.857 | 0.719 | 0.927 | 25.1 | -16.6 |
| wavelet-LHL_glrlm_RunPercentage | 4.81 | 13.33 | 0.845 | 0.705 | 0.92 | 15.9 | -10.7 |
| wavelet-LHL_glrlm_RunVariance | 24.87 | 68.95 | 0.767 | 0.575 | 0.877 | 56.1 | -81.8 |
| wavelet-LHL_glrlm_ShortRunEmphasis | 4.14 | 11.47 | 0.83 | 0.674 | 0.913 | 13.8 | -9.2 |
| wavelet-LHL_glrlm_ShortRunHighGrayLevelEmphasis | 35.02 | 97.08 | 0.489 | 0.197 | 0.702 | 161 | -33.2 |
| wavelet-LHL_glrlm_ShortRunLowGrayLevelEmphasis | 29.76 | 82.49 | 0.733 | 0.527 | 0.856 | 19.8 | -145.2 |
| wavelet-LHL_glszm_GrayLevelNonUniformity | 18.84 | 52.21 | 0.902 | 0.797 | 0.951 | 70.1 | -34.3 |
| wavelet-LHL_glszm_GrayLevelNonUniformityNormalized | 19.91 | 55.20 | 0.82 | 0.534 | 0.92 | 25.9 | -84.5 |
| wavelet-LHL_glszm_GrayLevelVariance | 30.24 | 83.83 | 0.604 | 0.29 | 0.789 | 138.4 | -29.3 |
| wavelet-LHL_glszm_HighGrayLevelZoneEmphasis | 32.91 | 91.21 | 0.465 | 0.171 | 0.684 | 148.7 | -33.7 |
| wavelet-LHL_glszm_LargeAreaEmphasis | 56.31 | 156.09 | 0.735 | 0.541 | 0.855 | 142.3 | -169.9 |
| wavelet-LHL_glszm_LargeAreaHighGrayLevelEmphasis | 49.57 | 137.41 | 0.966 | 0.934 | 0.982 | 182.2 | -92.7 |
| wavelet-LHL_glszm_LargeAreaLowGrayLevelEmphasis | 61.82 | 171.35 | 0.606 | 0.353 | 0.777 | 93.6 | -249.1 |
| wavelet-LHL_glszm_LowGrayLevelZoneEmphasis | 30.12 | 83.50 | 0.824 | 0.683 | 0.906 | 35 | -132 |
| wavelet-LHL_glszm_SizeZoneNonUniformity | 38.65 | 107.14 | 0.756 | 0.521 | 0.876 | 168.8 | -45.5 |
| wavelet-LHL_glszm_SizeZoneNonUniformityNormalized | 19.93 | 55.23 | 0.739 | 0.346 | 0.885 | 73.5 | -37 |
| wavelet-LHL_glszm_SmallAreaEmphasis | 42.05 | 116.57 | 0.778 | 0.599 | 0.882 | 114.3 | -118.9 |
| wavelet-LHL_glszm_SmallAreaHighGrayLevelEmphasis | 62.01 | 171.88 | 0.426 | 0.129 | 0.656 | 223.3 | -120.5 |
| wavelet-LHL_glszm_SmallAreaLowGrayLevelEmphasis | 53.74 | 148.96 | 0.349 | 0.031 | 0.605 | 129.5 | -168.4 |
| wavelet-LHL_glszm_ZoneEntropy | 10.13 | 28.07 | 0.861 | 0.53 | 0.945 | 35.3 | -20.8 |
| wavelet-LHL_glszm_ZonePercentage | 30.97 | 85.84 | 0.903 | 0.794 | 0.953 | 103.6 | -68 |
| wavelet-LHL_glszm_ZoneVariance | 59.11 | 163.85 | 0.737 | 0.545 | 0.856 | 153.7 | -174 |
| wavelet-LHL_ngtdm_Busyness | 36.15 | 100.19 | 0.709 | 0.502 | 0.84 | 50 | -150.4 |
| wavelet-LHL_ngtdm_Coarseness | 12.82 | 35.54 | 0.888 | 0.57 | 0.958 | 16.9 | -54.2 |
| wavelet-LHL_ngtdm_Complexity | 42.16 | 116.86 | 0.471 | 0.182 | 0.688 | 184.8 | -48.9 |
| wavelet-LHL_ngtdm_Contrast | 25.03 | 69.38 | 0.869 | 0.76 | 0.931 | 67.7 | -71.1 |
| wavelet-LHL_ngtdm_Strength | 34.06 | 94.42 | 0.609 | 0.288 | 0.794 | 141.4 | -47.5 |
| wavelet-LHH_firstorder_10Percentile | 20.90 | 57.92 | 0.833 | 0.307 | 0.941 | 82.4 | -33.5 |
| wavelet-LHH_firstorder_90Percentile | 18.94 | 52.51 | 0.874 | 0.738 | 0.938 | 63.3 | -41.7 |
| wavelet-LHH_firstorder_Energy | 37.95 | 105.21 | 0.616 | 0.347 | 0.788 | 169.5 | -40.9 |
| wavelet-LHH_firstorder_Entropy | 5.69 | 15.77 | 0.668 | 0.366 | 0.829 | 20.4 | -11.1 |
| wavelet-LHH_firstorder_InterquartileRange | 22.53 | 62.46 | 0.83 | 0.634 | 0.918 | 76.5 | -48.4 |
| wavelet-LHH_firstorder_Kurtosis | 15.89 | 44.06 | 0.711 | 0.388 | 0.86 | 59 | -29.1 |
| wavelet-LHH_firstorder_Maximum | 21.56 | 59.77 | 0.714 | 0.33 | 0.87 | 87.6 | -31.9 |
| wavelet-LHH_firstorder_MeanAbsoluteDeviation | 19.60 | 54.33 | 0.844 | 0.511 | 0.937 | 72 | -36.6 |
| wavelet-LHH_firstorder_Mean | 208.68 | 578.43 | 0.623 | 0.309 | 0.802 | 590.9 | -565.9 |
| wavelet-LHH_firstorder_Median | 2501.61 | 6934.10 | 0.491 | 0.192 | 0.706 | 7661.2 | -6206.9 |
| wavelet-LHH_firstorder_Minimum | 21.79 | 60.39 | 0.76 | 0.273 | 0.904 | 90.9 | -29.9 |
| wavelet-LHH_firstorder_Range | 20.93 | 58.01 | 0.735 | 0.306 | 0.886 | 86.9 | -29.1 |
| wavelet-LHH_firstorder_RobustMeanAbsoluteDeviation | 20.91 | 57.96 | 0.854 | 0.644 | 0.933 | 72.1 | -43.8 |
| wavelet-LHH_firstorder_RootMeanSquared | 19.01 | 52.71 | 0.818 | 0.428 | 0.927 | 72.6 | -32.8 |
| wavelet-LHH_firstorder_Skewness | 44.92 | 124.53 | 0.656 | 0.425 | 0.808 | 127.3 | -121.8 |
| wavelet-LHH_firstorder_TotalEnergy | 37.95 | 105.21 | 0.616 | 0.347 | 0.788 | 169.5 | -40.9 |
| wavelet-LHH_firstorder_Uniformity | 3.11 | 8.63 | 0.68 | 0.411 | 0.831 | 6.4 | -10.9 |
| wavelet-LHH_firstorder_Variance | 34.92 | 96.79 | 0.713 | 0.382 | 0.862 | 136.4 | -57.2 |
| wavelet-LHH_glcm_Autocorrelation | 24.71 | 68.49 | 0.537 | 0.247 | 0.736 | 82.9 | -54.1 |
| wavelet-LHH_glcm_ClusterProminence | 34.73 | 96.26 | 0.266 | -0.036 | 0.533 | 124.5 | -68.1 |
| wavelet-LHH_glcm_ClusterShade | 3531.49 | 9788.79 | 0.483 | 0.177 | 0.702 | 9043.5 | -10534.1 |
| wavelet-LHH_glcm_ClusterTendency | 12.70 | 35.20 | 0.555 | 0.259 | 0.751 | 44.7 | -25.7 |
| wavelet-LHH_glcm_Contrast | 10.21 | 28.31 | 0.565 | 0.267 | 0.758 | 36.3 | -20.4 |
| wavelet-LHH_glcm_Correlation | 6.18 | 17.14 | 0.71 | 0.503 | 0.84 | 18.1 | -16.2 |
| wavelet-LHH_glcm_DifferenceAverage | 7.62 | 21.13 | 0.657 | 0.371 | 0.819 | 27.2 | -15.1 |
| wavelet-LHH_glcm_DifferenceEntropy | 5.15 | 14.28 | 0.639 | 0.341 | 0.81 | 18.4 | -10.2 |
| wavelet-LHH_glcm_DifferenceVariance | 8.29 | 22.97 | 0.544 | 0.24 | 0.745 | 29.4 | -16.6 |
| wavelet-LHH_glcm_Id | 1.30 | 3.60 | 0.691 | 0.415 | 0.84 | 2.7 | -4.5 |
| wavelet-LHH_glcm_Idm | 1.39 | 3.86 | 0.679 | 0.398 | 0.833 | 2.9 | -4.9 |
| wavelet-LHH_glcm_Idmn | 0.86 | 2.38 | 0.641 | 0.336 | 0.812 | 3 | -1.7 |
| wavelet-LHH_glcm_Idn | 0.76 | 2.10 | 0.668 | 0.392 | 0.825 | 2.6 | -1.6 |
| wavelet-LHH_glcm_Imc1 | 9.92 | 27.49 | 0.701 | 0.489 | 0.835 | 26.3 | -28.7 |
| wavelet-LHH_glcm_Imc2 | 5.49 | 15.22 | 0.737 | 0.542 | 0.857 | 17.1 | -13.3 |
| wavelet-LHH_glcm_InverseVariance | 5.72 | 15.87 | 0.737 | 0.49 | 0.866 | 20.1 | -11.7 |
| wavelet-LHH_glcm_JointAverage | 13.35 | 37.01 | 0.565 | 0.283 | 0.754 | 44.7 | -29.3 |
| wavelet-LHH_glcm_JointEnergy | 6.56 | 18.18 | 0.729 | 0.464 | 0.864 | 13 | -23.4 |
| wavelet-LHH_glcm_JointEntropy | 6.31 | 17.49 | 0.692 | 0.39 | 0.845 | 22.9 | -12.1 |
| wavelet-LHH_glcm_MCC | 5.65 | 15.66 | 0.734 | 0.54 | 0.854 | 17.2 | -14.1 |
| wavelet-LHH_glcm_MaximumProbability | 7.24 | 20.07 | 0.648 | 0.406 | 0.804 | 16.2 | -24 |
| wavelet-LHH_glcm_SumAverage | 13.35 | 37.01 | 0.565 | 0.283 | 0.754 | 44.7 | -29.3 |
| wavelet-LHH_glcm_SumEntropy | 5.61 | 15.54 | 0.717 | 0.415 | 0.861 | 20.3 | -10.8 |
| wavelet-LHH_glcm_SumSquares | 11.76 | 32.61 | 0.554 | 0.256 | 0.75 | 41.8 | -23.4 |
| wavelet-LHH_gldm_DependenceEntropy | 1.85 | 5.11 | 0.587 | 0.316 | 0.767 | 6.1 | -4.1 |
| wavelet-LHH_gldm_DependenceNonUniformity | 14.02 | 38.86 | 0.985 | 0.97 | 0.993 | 67.6 | -10.1 |
| wavelet-LHH_gldm_DependenceNonUniformityNormalized | 4.69 | 13.01 | 0.498 | 0.209 | 0.707 | 12.7 | -13.3 |
| wavelet-LHH_gldm_DependenceVariance | 6.31 | 17.49 | 0.595 | 0.338 | 0.77 | 18.7 | -16.3 |
| wavelet-LHH_gldm_GrayLevelNonUniformity | 15.00 | 41.59 | 0.988 | 0.975 | 0.994 | 68.3 | -14.8 |
| wavelet-LHH_gldm_GrayLevelVariance | 10.60 | 29.38 | 0.533 | 0.231 | 0.736 | 37.5 | -21.3 |
| wavelet-LHH_gldm_HighGrayLevelEmphasis | 24.42 | 67.70 | 0.532 | 0.242 | 0.733 | 82.2 | -53.2 |
| wavelet-LHH_gldm_LargeDependenceEmphasis | 6.39 | 17.71 | 0.845 | 0.718 | 0.918 | 20 | -15.4 |
| wavelet-LHH_gldm_LargeDependenceHighGrayLevelEmphasis | 23.55 | 65.28 | 0.535 | 0.245 | 0.735 | 80 | -50.5 |
| wavelet-LHH_gldm_LargeDependenceLowGrayLevelEmphasis | 33.25 | 92.16 | 0.526 | 0.246 | 0.726 | 79.8 | -104.5 |
| wavelet-LHH_gldm_LowGrayLevelEmphasis | 28.57 | 79.19 | 0.535 | 0.26 | 0.731 | 62.7 | -95.6 |
| wavelet-LHH_gldm_SmallDependenceEmphasis | 15.84 | 43.90 | 0.547 | 0.275 | 0.739 | 35.7 | -52 |
| wavelet-LHH_gldm_SmallDependenceHighGrayLevelEmphasis | 36.19 | 100.32 | 0.374 | 0.072 | 0.618 | 114.7 | -85.9 |
| wavelet-LHH_gldm_SmallDependenceLowGrayLevelEmphasis | 21.62 | 59.92 | 0.73 | 0.224 | 0.891 | 31.6 | -88.2 |
| wavelet-LHH_glrlm_GrayLevelNonUniformity | 13.97 | 38.72 | 0.99 | 0.978 | 0.995 | 63.2 | -14.3 |
| wavelet-LHH_glrlm_GrayLevelNonUniformityNormalized | 4.30 | 11.93 | 0.682 | 0.393 | 0.837 | 8.7 | -15.2 |
| wavelet-LHH_glrlm_GrayLevelVariance | 13.62 | 37.74 | 0.501 | 0.199 | 0.714 | 48.5 | -27 |
| wavelet-LHH_glrlm_HighGrayLevelRunEmphasis | 24.17 | 66.99 | 0.555 | 0.261 | 0.75 | 83.2 | -50.8 |
| wavelet-LHH_glrlm_LongRunEmphasis | 9.55 | 26.47 | 0.899 | 0.8 | 0.949 | 31.9 | -21 |
| wavelet-LHH_glrlm_LongRunHighGrayLevelEmphasis | 22.37 | 62.01 | 0.581 | 0.276 | 0.771 | 80.7 | -43.3 |
| wavelet-LHH_glrlm_LongRunLowGrayLevelEmphasis | 34.29 | 95.06 | 0.613 | 0.363 | 0.781 | 85.3 | -104.8 |
| wavelet-LHH_glrlm_LowGrayLevelRunEmphasis | 27.58 | 76.46 | 0.594 | 0.326 | 0.771 | 58.9 | -94 |
| wavelet-LHH_glrlm_RunEntropy | 2.69 | 7.47 | 0.848 | 0.314 | 0.948 | 11.2 | -3.7 |
| wavelet-LHH_glrlm_RunLengthNonUniformity | 15.50 | 42.96 | 0.989 | 0.972 | 0.995 | 68.7 | -17.2 |
| wavelet-LHH_glrlm_RunLengthNonUniformityNormalized | 5.72 | 15.86 | 0.811 | 0.662 | 0.898 | 14 | -17.7 |
| wavelet-LHH_glrlm_RunPercentage | 4.19 | 11.62 | 0.842 | 0.714 | 0.916 | 10.1 | -13.1 |
| wavelet-LHH_glrlm_RunVariance | 10.89 | 30.20 | 0.92 | 0.797 | 0.964 | 39.8 | -20.6 |
| wavelet-LHH_glrlm_ShortRunEmphasis | 4.72 | 13.08 | 0.794 | 0.634 | 0.889 | 12.6 | -13.6 |
| wavelet-LHH_glrlm_ShortRunHighGrayLevelEmphasis | 26.94 | 74.67 | 0.537 | 0.243 | 0.738 | 92.6 | -56.7 |
| wavelet-LHH_glrlm_ShortRunLowGrayLevelEmphasis | 24.59 | 68.16 | 0.701 | 0.41 | 0.85 | 48.4 | -87.9 |
| wavelet-LHH_glszm_GrayLevelNonUniformity | 30.52 | 84.58 | 0.965 | 0.933 | 0.982 | 73.1 | -96 |
| wavelet-LHH_glszm_GrayLevelNonUniformityNormalized | 15.66 | 43.42 | 0.639 | 0.232 | 0.828 | 25.5 | -61.3 |
| wavelet-LHH_glszm_GrayLevelVariance | 35.95 | 99.64 | 0.576 | 0.25 | 0.772 | 138.2 | -61.1 |
| wavelet-LHH_glszm_HighGrayLevelZoneEmphasis | 26.87 | 74.49 | 0.558 | 0.189 | 0.769 | 106.3 | -42.7 |
| wavelet-LHH_glszm_LargeAreaEmphasis | 34.86 | 96.64 | 0.913 | 0.838 | 0.955 | 146.2 | -47.1 |
| wavelet-LHH_glszm_LargeAreaHighGrayLevelEmphasis | 34.00 | 94.24 | 0.921 | 0.85 | 0.959 | 153.6 | -34.9 |
| wavelet-LHH_glszm_LargeAreaLowGrayLevelEmphasis | 53.58 | 148.51 | 0.903 | 0.819 | 0.949 | 184.1 | -112.9 |
| wavelet-LHH_glszm_LowGrayLevelZoneEmphasis | 20.90 | 57.93 | 0.582 | 0.217 | 0.784 | 38.6 | -77.3 |
| wavelet-LHH_glszm_SizeZoneNonUniformity | 26.33 | 72.97 | 0.644 | 0.406 | 0.8 | 76.1 | -69.8 |
| wavelet-LHH_glszm_SizeZoneNonUniformityNormalized | 33.66 | 93.30 | 0.261 | -0.068 | 0.539 | 91.1 | -95.6 |
| wavelet-LHH_glszm_SmallAreaEmphasis | 86.07 | 238.57 | 0.159 | -0.173 | 0.459 | 228.3 | -248.9 |
| wavelet-LHH_glszm_SmallAreaHighGrayLevelEmphasis | 91.02 | 252.30 | 0.353 | 0.042 | 0.605 | 273.3 | -231.3 |
| wavelet-LHH_glszm_SmallAreaLowGrayLevelEmphasis | 88.33 | 244.83 | 0.124 | -0.179 | 0.418 | 215.4 | -274.2 |
| wavelet-LHH_glszm_ZoneEntropy | 23.88 | 66.18 | 0.55 | 0.28 | 0.74 | 72.9 | -59.5 |
| wavelet-LHH_glszm_ZonePercentage | 29.53 | 81.87 | 0.539 | 0.233 | 0.742 | 59.2 | -104.5 |
| wavelet-LHH_glszm_ZoneVariance | 51.35 | 142.33 | 0.915 | 0.841 | 0.956 | 192.1 | -92.5 |
| wavelet-LHH_ngtdm_Busyness | 49.93 | 138.40 | 0.527 | 0.247 | 0.726 | 122.9 | -153.9 |
| wavelet-LHH_ngtdm_Coarseness | 16.36 | 45.35 | 0.85 | 0.522 | 0.939 | 17.5 | -73.2 |
| wavelet-LHH_ngtdm_Complexity | 45.00 | 124.74 | 0.503 | 0.192 | 0.717 | 167.9 | -81.6 |
| wavelet-LHH_ngtdm_Contrast | 33.94 | 94.08 | 0.583 | 0.289 | 0.77 | 63.7 | -124.4 |
| wavelet-LHH_ngtdm_Strength | 44.25 | 122.65 | 0.482 | 0.191 | 0.696 | 139.7 | -105.6 |
| wavelet-HLL_firstorder_10Percentile | 16.20 | 44.91 | 0.806 | 0.35 | 0.925 | 63.7 | -26.1 |
| wavelet-HLL_firstorder_90Percentile | 28.44 | 78.84 | 0.795 | 0.237 | 0.926 | 113.8 | -43.9 |
| wavelet-HLL_firstorder_Energy | 29.31 | 81.25 | 0.625 | 0.358 | 0.794 | 146.8 | -15.7 |
| wavelet-HLL_firstorder_Entropy | 8.31 | 23.03 | 0.848 | 0.364 | 0.946 | 32 | -14 |
| wavelet-HLL_firstorder_InterquartileRange | 21.31 | 59.06 | 0.82 | 0.467 | 0.926 | 77.1 | -41.1 |
| wavelet-HLL_firstorder_Kurtosis | 12.77 | 35.40 | 0.767 | 0.544 | 0.881 | 44.8 | -26 |
| wavelet-HLL_firstorder_Maximum | 25.61 | 70.97 | 0.785 | 0.28 | 0.918 | 102.4 | -39.5 |
| wavelet-HLL_firstorder_MeanAbsoluteDeviation | 18.03 | 49.98 | 0.807 | 0.252 | 0.931 | 72.4 | -27.5 |
| wavelet-HLL_firstorder_Mean | 14.05 | 38.93 | 0.858 | 0.741 | 0.925 | 37.1 | -40.8 |
| wavelet-HLL_firstorder_Median | 40.98 | 113.60 | 0.607 | 0.284 | 0.793 | 85.5 | -141.7 |
| wavelet-HLL_firstorder_Minimum | 17.92 | 49.68 | 0.745 | 0.269 | 0.896 | 77.9 | -21.5 |
| wavelet-HLL_firstorder_Range | 17.98 | 49.83 | 0.771 | 0.25 | 0.912 | 79.3 | -20.4 |
| wavelet-HLL_firstorder_RobustMeanAbsoluteDeviation | 19.29 | 53.47 | 0.827 | 0.392 | 0.934 | 72.9 | -34.1 |
| wavelet-HLL_firstorder_RootMeanSquared | 15.19 | 42.10 | 0.796 | 0.299 | 0.923 | 62.3 | -21.9 |
| wavelet-HLL_firstorder_Skewness | 111.91 | 310.20 | 0.723 | 0.408 | 0.867 | 373.1 | -247.3 |
| wavelet-HLL_firstorder_TotalEnergy | 29.31 | 81.25 | 0.625 | 0.358 | 0.794 | 146.8 | -15.7 |
| wavelet-HLL_firstorder_Uniformity | 17.43 | 48.31 | 0.852 | 0.709 | 0.925 | 28.6 | -68 |
| wavelet-HLL_firstorder_Variance | 29.92 | 82.93 | 0.654 | 0.313 | 0.827 | 128.7 | -37.1 |
| wavelet-HLL_glcm_Autocorrelation | 31.66 | 87.76 | 0.547 | 0.253 | 0.745 | 146.1 | -29.5 |
| wavelet-HLL_glcm_ClusterProminence | 47.43 | 131.47 | 0.222 | -0.093 | 0.503 | 221.1 | -41.9 |
| wavelet-HLL_glcm_ClusterShade | 205.18 | 568.72 | 0.297 | -0.01 | 0.56 | 651.1 | -486.3 |
| wavelet-HLL_glcm_ClusterTendency | 30.99 | 85.91 | 0.643 | 0.31 | 0.819 | 133.9 | -37.9 |
| wavelet-HLL_glcm_Contrast | 29.77 | 82.51 | 0.709 | 0.361 | 0.862 | 122.5 | -42.5 |
| wavelet-HLL_glcm_Correlation | 9.26 | 25.68 | 0.534 | 0.257 | 0.731 | 29.4 | -21.9 |
| wavelet-HLL_glcm_DifferenceAverage | 17.60 | 48.78 | 0.833 | 0.426 | 0.936 | 68.6 | -29 |
| wavelet-HLL_glcm_DifferenceEntropy | 9.20 | 25.51 | 0.849 | 0.363 | 0.947 | 36.2 | -14.8 |
| wavelet-HLL_glcm_DifferenceVariance | 29.02 | 80.43 | 0.65 | 0.313 | 0.824 | 125.2 | -35.6 |
| wavelet-HLL_glcm_Id | 7.67 | 21.27 | 0.873 | 0.677 | 0.943 | 13.9 | -28.6 |
| wavelet-HLL_glcm_Idm | 10.39 | 28.80 | 0.869 | 0.685 | 0.94 | 19 | -38.6 |
| wavelet-HLL_glcm_Idmn | 0.41 | 1.12 | 0.791 | 0.566 | 0.897 | 1.4 | -0.8 |
| wavelet-HLL_glcm_Idn | 0.89 | 2.46 | 0.853 | 0.633 | 0.934 | 3.2 | -1.7 |
| wavelet-HLL_glcm_Imc1 | 11.11 | 30.80 | 0.668 | 0.44 | 0.815 | 32.1 | -29.5 |
| wavelet-HLL_glcm_Imc2 | 6.63 | 18.38 | 0.603 | 0.349 | 0.775 | 21.1 | -15.7 |
| wavelet-HLL_glcm_InverseVariance | 7.44 | 20.62 | 0.851 | 0.581 | 0.936 | 13.2 | -28.1 |
| wavelet-HLL_glcm_JointAverage | 17.33 | 48.03 | 0.742 | 0.256 | 0.895 | 79.2 | -16.9 |
| wavelet-HLL_glcm_JointEnergy | 26.47 | 73.38 | 0.929 | 0.858 | 0.965 | 38.9 | -107.9 |
| wavelet-HLL_glcm_JointEntropy | 7.72 | 21.41 | 0.858 | 0.255 | 0.954 | 31.1 | -11.7 |
| wavelet-HLL_glcm_MCC | 7.35 | 20.38 | 0.43 | 0.129 | 0.66 | 22.6 | -18.2 |
| wavelet-HLL_glcm_MaximumProbability | 25.92 | 71.84 | 0.901 | 0.813 | 0.949 | 51.3 | -92.3 |
| wavelet-HLL_glcm_SumAverage | 17.33 | 48.03 | 0.742 | 0.256 | 0.895 | 79.2 | -16.9 |
| wavelet-HLL_glcm_SumEntropy | 6.99 | 19.37 | 0.846 | 0.262 | 0.949 | 27.9 | -10.8 |
| wavelet-HLL_glcm_SumSquares | 30.40 | 84.27 | 0.651 | 0.315 | 0.824 | 131.2 | -37.3 |
| wavelet-HLL_gldm_DependenceEntropy | 2.03 | 5.63 | 0.882 | 0.045 | 0.969 | 9.9 | -1.3 |
| wavelet-HLL_gldm_DependenceNonUniformity | 15.73 | 43.59 | 0.924 | 0.851 | 0.961 | 78.3 | -8.9 |
| wavelet-HLL_gldm_DependenceNonUniformityNormalized | 14.83 | 41.12 | 0.862 | 0.747 | 0.927 | 46.8 | -35.4 |
| wavelet-HLL_gldm_DependenceVariance | 27.14 | 75.22 | 0.821 | 0.672 | 0.906 | 64.8 | -85.7 |
| wavelet-HLL_gldm_GrayLevelNonUniformity | 25.87 | 71.71 | 0.981 | 0.963 | 0.99 | 81.2 | -62.2 |
| wavelet-HLL_gldm_GrayLevelVariance | 29.59 | 82.01 | 0.654 | 0.313 | 0.827 | 127.6 | -36.5 |
| wavelet-HLL_gldm_HighGrayLevelEmphasis | 31.15 | 86.34 | 0.554 | 0.259 | 0.75 | 143.4 | -29.3 |
| wavelet-HLL_gldm_LargeDependenceEmphasis | 23.43 | 64.95 | 0.875 | 0.753 | 0.937 | 51.3 | -78.6 |
| wavelet-HLL_gldm_LargeDependenceHighGrayLevelEmphasis | 28.73 | 79.63 | 0.598 | 0.313 | 0.779 | 124.9 | -34.4 |
| wavelet-HLL_gldm_LargeDependenceLowGrayLevelEmphasis | 47.85 | 132.62 | 0.458 | 0.161 | 0.68 | 62 | -203.2 |
| wavelet-HLL_gldm_LowGrayLevelEmphasis | 27.79 | 77.02 | 0.643 | 0.405 | 0.8 | 22.9 | -131.2 |
| wavelet-HLL_gldm_SmallDependenceEmphasis | 21.01 | 58.24 | 0.868 | 0.665 | 0.941 | 76 | -40.4 |
| wavelet-HLL_gldm_SmallDependenceHighGrayLevelEmphasis | 40.64 | 112.65 | 0.485 | 0.197 | 0.698 | 182.1 | -43.2 |
| wavelet-HLL_gldm_SmallDependenceLowGrayLevelEmphasis | 22.06 | 61.13 | 0.805 | 0.577 | 0.906 | 36 | -86.2 |
| wavelet-HLL_glrlm_GrayLevelNonUniformity | 22.56 | 62.53 | 0.997 | 0.994 | 0.998 | 73.6 | -51.5 |
| wavelet-HLL_glrlm_GrayLevelNonUniformityNormalized | 16.30 | 45.17 | 0.85 | 0.683 | 0.927 | 24.7 | -65.6 |
| wavelet-HLL_glrlm_GrayLevelVariance | 28.68 | 79.50 | 0.646 | 0.304 | 0.822 | 126.1 | -32.9 |
| wavelet-HLL_glrlm_HighGrayLevelRunEmphasis | 30.80 | 85.37 | 0.555 | 0.26 | 0.75 | 142.2 | -28.5 |
| wavelet-HLL_glrlm_LongRunEmphasis | 11.92 | 33.05 | 0.868 | 0.75 | 0.932 | 26.8 | -39.3 |
| wavelet-HLL_glrlm_LongRunHighGrayLevelEmphasis | 27.67 | 76.71 | 0.553 | 0.253 | 0.75 | 128.7 | -24.7 |
| wavelet-HLL_glrlm_LongRunLowGrayLevelEmphasis | 33.73 | 93.48 | 0.489 | 0.201 | 0.701 | 33.3 | -153.6 |
| wavelet-HLL_glrlm_LowGrayLevelRunEmphasis | 26.95 | 74.71 | 0.709 | 0.497 | 0.841 | 21.4 | -128.1 |
| wavelet-HLL_glrlm_RunEntropy | 3.91 | 10.85 | 0.835 | 0.09 | 0.95 | 17.2 | -4.5 |
| wavelet-HLL_glrlm_RunLengthNonUniformity | 13.24 | 36.70 | 0.932 | 0.869 | 0.965 | 71.4 | -2 |
| wavelet-HLL_glrlm_RunLengthNonUniformityNormalized | 5.66 | 15.69 | 0.889 | 0.757 | 0.947 | 19.5 | -11.8 |
| wavelet-HLL_glrlm_RunPercentage | 3.78 | 10.48 | 0.886 | 0.765 | 0.943 | 12.8 | -8.1 |
| wavelet-HLL_glrlm_RunVariance | 24.53 | 67.98 | 0.816 | 0.668 | 0.902 | 58.8 | -77.2 |
| wavelet-HLL_glrlm_ShortRunEmphasis | 3.08 | 8.53 | 0.895 | 0.77 | 0.949 | 10.6 | -6.5 |
| wavelet-HLL_glrlm_ShortRunHighGrayLevelEmphasis | 31.56 | 87.47 | 0.554 | 0.26 | 0.75 | 145.7 | -29.3 |
| wavelet-HLL_glrlm_ShortRunLowGrayLevelEmphasis | 25.32 | 70.17 | 0.818 | 0.625 | 0.91 | 19.6 | -120.8 |
| wavelet-HLL_glszm_GrayLevelNonUniformity | 17.60 | 48.78 | 0.848 | 0.72 | 0.92 | 68.7 | -28.8 |
| wavelet-HLL_glszm_GrayLevelNonUniformityNormalized | 15.25 | 42.28 | 0.884 | 0.539 | 0.957 | 14.5 | -70.1 |
| wavelet-HLL_glszm_GrayLevelVariance | 26.38 | 73.13 | 0.615 | 0.259 | 0.804 | 125.6 | -20.7 |
| wavelet-HLL_glszm_HighGrayLevelZoneEmphasis | 28.07 | 77.81 | 0.568 | 0.279 | 0.758 | 132.5 | -23.1 |
| wavelet-HLL_glszm_LargeAreaEmphasis | 52.30 | 144.96 | 0.601 | 0.348 | 0.774 | 124.9 | -165 |
| wavelet-HLL_glszm_LargeAreaHighGrayLevelEmphasis | 44.92 | 124.51 | 0.756 | 0.573 | 0.867 | 159.3 | -89.7 |
| wavelet-HLL_glszm_LargeAreaLowGrayLevelEmphasis | 60.68 | 168.20 | 0.37 | 0.057 | 0.619 | 95.3 | -241.1 |
| wavelet-HLL_glszm_LowGrayLevelZoneEmphasis | 24.97 | 69.20 | 0.873 | 0.725 | 0.938 | 23 | -115.4 |
| wavelet-HLL_glszm_SizeZoneNonUniformity | 35.29 | 97.83 | 0.642 | 0.363 | 0.808 | 156.1 | -39.6 |
| wavelet-HLL_glszm_SizeZoneNonUniformityNormalized | 18.02 | 49.96 | 0.668 | 0.349 | 0.832 | 63.1 | -36.8 |
| wavelet-HLL_glszm_SmallAreaEmphasis | 23.30 | 64.59 | 0.741 | 0.422 | 0.878 | 80.1 | -49.1 |
| wavelet-HLL_glszm_SmallAreaHighGrayLevelEmphasis | 36.52 | 101.23 | 0.515 | 0.227 | 0.72 | 166.6 | -35.8 |
| wavelet-HLL_glszm_SmallAreaLowGrayLevelEmphasis | 46.04 | 127.61 | 0.343 | 0.023 | 0.6 | 108.6 | -146.6 |
| wavelet-HLL_glszm_ZoneEntropy | 5.82 | 16.13 | 0.898 | 0.384 | 0.968 | 23.7 | -8.6 |
| wavelet-HLL_glszm_ZonePercentage | 25.88 | 71.75 | 0.879 | 0.727 | 0.942 | 90.3 | -53.2 |
| wavelet-HLL_glszm_ZoneVariance | 55.20 | 153.00 | 0.603 | 0.35 | 0.775 | 139.3 | -166.7 |
| wavelet-HLL_ngtdm_Busyness | 33.99 | 94.22 | 0.537 | 0.264 | 0.732 | 45.7 | -142.7 |
| wavelet-HLL_ngtdm_Coarseness | 15.89 | 44.05 | 0.865 | 0.601 | 0.944 | 26.6 | -61.5 |
| wavelet-HLL_ngtdm_Complexity | 39.25 | 108.79 | 0.54 | 0.255 | 0.737 | 174.9 | -42.6 |
| wavelet-HLL_ngtdm_Contrast | 23.81 | 66.01 | 0.786 | 0.621 | 0.884 | 67 | -65.1 |
| wavelet-HLL_ngtdm_Strength | 31.83 | 88.24 | 0.657 | 0.374 | 0.819 | 129.6 | -46.8 |
| wavelet-HLH_firstorder_10Percentile | 21.41 | 59.36 | 0.82 | 0.467 | 0.926 | 78.4 | -40.3 |
| wavelet-HLH_firstorder_90Percentile | 16.90 | 46.83 | 0.849 | 0.713 | 0.922 | 55.8 | -37.9 |
| wavelet-HLH_firstorder_Energy | 32.82 | 90.97 | 0.72 | 0.491 | 0.851 | 146.8 | -35.1 |
| wavelet-HLH_firstorder_Entropy | 6.02 | 16.70 | 0.752 | 0.502 | 0.876 | 21.2 | -12.2 |
| wavelet-HLH_firstorder_InterquartileRange | 19.51 | 54.07 | 0.86 | 0.739 | 0.927 | 60.9 | -47.3 |
| wavelet-HLH_firstorder_Kurtosis | 18.53 | 51.37 | 0.557 | 0.282 | 0.747 | 61.8 | -40.9 |
| wavelet-HLH_firstorder_Maximum | 21.05 | 58.33 | 0.706 | 0.359 | 0.86 | 80 | -36.7 |
| wavelet-HLH_firstorder_MeanAbsoluteDeviation | 17.44 | 48.34 | 0.851 | 0.631 | 0.932 | 61.1 | -35.6 |
| wavelet-HLH_firstorder_Mean | 87.18 | 241.65 | 0.588 | 0.237 | 0.786 | 218.5 | -264.8 |
| wavelet-HLH_firstorder_Median | 567.04 | 1571.75 | 0.433 | 0.105 | 0.672 | 1548.2 | -1595.3 |
| wavelet-HLH_firstorder_Minimum | 21.36 | 59.21 | 0.749 | 0.397 | 0.887 | 81.6 | -36.8 |
| wavelet-HLH_firstorder_Range | 19.86 | 55.04 | 0.729 | 0.369 | 0.876 | 77 | -33.1 |
| wavelet-HLH_firstorder_RobustMeanAbsoluteDeviation | 18.54 | 51.40 | 0.864 | 0.733 | 0.931 | 60.3 | -42.5 |
| wavelet-HLH_firstorder_RootMeanSquared | 17.05 | 47.25 | 0.827 | 0.54 | 0.925 | 62 | -32.5 |
| wavelet-HLH_firstorder_Skewness | 994.67 | 2757.08 | 0.374 | 0.061 | 0.622 | 2998.4 | -2515.8 |
| wavelet-HLH_firstorder_TotalEnergy | 32.82 | 90.97 | 0.72 | 0.491 | 0.851 | 146.8 | -35.1 |
| wavelet-HLH_firstorder_Uniformity | 3.52 | 9.75 | 0.733 | 0.518 | 0.858 | 7.6 | -11.9 |
| wavelet-HLH_firstorder_Variance | 31.10 | 86.20 | 0.772 | 0.463 | 0.895 | 116 | -56.4 |
| wavelet-HLH_glcm_Autocorrelation | 29.55 | 81.91 | 0.523 | 0.221 | 0.73 | 101.6 | -62.2 |
| wavelet-HLH_glcm_ClusterProminence | 35.55 | 98.55 | 0.44 | 0.14 | 0.668 | 128.1 | -69 |
| wavelet-HLH_glcm_ClusterShade | 713.96 | 1978.99 | 0.569 | 0.269 | 0.762 | 1860.1 | -2097.9 |
| wavelet-HLH_glcm_ClusterTendency | 12.94 | 35.86 | 0.679 | 0.404 | 0.832 | 45.3 | -26.4 |
| wavelet-HLH_glcm_Contrast | 11.67 | 32.34 | 0.687 | 0.448 | 0.831 | 39.1 | -25.6 |
| wavelet-HLH_glcm_Correlation | 8.31 | 23.04 | 0.45 | 0.152 | 0.674 | 25.4 | -20.6 |
| wavelet-HLH_glcm_DifferenceAverage | 9.50 | 26.33 | 0.716 | 0.507 | 0.845 | 30.8 | -21.9 |
| wavelet-HLH_glcm_DifferenceEntropy | 5.68 | 15.75 | 0.698 | 0.421 | 0.845 | 20 | -11.5 |
| wavelet-HLH_glcm_DifferenceVariance | 8.66 | 24.02 | 0.66 | 0.365 | 0.823 | 30.8 | -17.3 |
| wavelet-HLH_glcm_Id | 1.68 | 4.65 | 0.722 | 0.519 | 0.847 | 4 | -5.3 |
| wavelet-HLH_glcm_Idm | 1.75 | 4.85 | 0.72 | 0.516 | 0.847 | 4.1 | -5.6 |
| wavelet-HLH_glcm_Idmn | 0.97 | 2.69 | 0.621 | 0.37 | 0.788 | 3.2 | -2.2 |
| wavelet-HLH_glcm_Idn | 1.06 | 2.93 | 0.456 | 0.165 | 0.677 | 3.4 | -2.4 |
| wavelet-HLH_glcm_Imc1 | 12.50 | 34.64 | 0.519 | 0.237 | 0.721 | 32.7 | -36.5 |
| wavelet-HLH_glcm_Imc2 | 7.54 | 20.91 | 0.555 | 0.287 | 0.743 | 23.4 | -18.5 |
| wavelet-HLH_glcm_InverseVariance | 7.98 | 22.12 | 0.717 | 0.515 | 0.845 | 24.5 | -19.8 |
| wavelet-HLH_glcm_JointAverage | 15.99 | 44.33 | 0.545 | 0.248 | 0.744 | 54.8 | -33.9 |
| wavelet-HLH_glcm_JointEnergy | 7.82 | 21.67 | 0.765 | 0.573 | 0.875 | 17.2 | -26.1 |
| wavelet-HLH_glcm_JointEntropy | 6.91 | 19.15 | 0.758 | 0.518 | 0.878 | 24.2 | -14.1 |
| wavelet-HLH_glcm_MCC | 7.78 | 21.57 | 0.524 | 0.245 | 0.724 | 23.8 | -19.4 |
| wavelet-HLH_glcm_MaximumProbability | 8.26 | 22.90 | 0.723 | 0.522 | 0.848 | 21.4 | -24.3 |
| wavelet-HLH_glcm_SumAverage | 15.99 | 44.33 | 0.545 | 0.248 | 0.744 | 54.8 | -33.9 |
| wavelet-HLH_glcm_SumEntropy | 5.98 | 16.58 | 0.77 | 0.525 | 0.887 | 21.1 | -12 |
| wavelet-HLH_glcm_SumSquares | 12.07 | 33.47 | 0.687 | 0.418 | 0.836 | 42.2 | -24.8 |
| wavelet-HLH_gldm_DependenceEntropy | 1.60 | 4.43 | 0.653 | 0.299 | 0.828 | 5.9 | -3 |
| wavelet-HLH_gldm_DependenceNonUniformity | 16.21 | 44.94 | 0.986 | 0.972 | 0.993 | 71.3 | -18.6 |
| wavelet-HLH_gldm_DependenceNonUniformityNormalized | 5.22 | 14.46 | 0.633 | 0.39 | 0.794 | 11.8 | -17.1 |
| wavelet-HLH_gldm_DependenceVariance | 7.07 | 19.60 | 0.602 | 0.308 | 0.784 | 24.4 | -14.8 |
| wavelet-HLH_gldm_GrayLevelNonUniformity | 14.83 | 41.11 | 0.988 | 0.976 | 0.994 | 68 | -14.2 |
| wavelet-HLH_gldm_GrayLevelVariance | 10.68 | 29.60 | 0.672 | 0.394 | 0.828 | 37.6 | -21.6 |
| wavelet-HLH_gldm_HighGrayLevelEmphasis | 28.99 | 80.35 | 0.52 | 0.217 | 0.727 | 99.5 | -61.2 |
| wavelet-HLH_gldm_LargeDependenceEmphasis | 7.52 | 20.85 | 0.819 | 0.667 | 0.905 | 24.4 | -17.3 |
| wavelet-HLH_gldm_LargeDependenceHighGrayLevelEmphasis | 27.41 | 75.98 | 0.484 | 0.164 | 0.706 | 97.5 | -54.4 |
| wavelet-HLH_gldm_LargeDependenceLowGrayLevelEmphasis | 38.27 | 106.09 | 0.446 | 0.151 | 0.67 | 87.3 | -124.9 |
| wavelet-HLH_gldm_LowGrayLevelEmphasis | 35.70 | 98.96 | 0.508 | 0.223 | 0.714 | 77 | -120.9 |
| wavelet-HLH_gldm_SmallDependenceEmphasis | 18.59 | 51.52 | 0.535 | 0.258 | 0.731 | 46.9 | -56.1 |
| wavelet-HLH_gldm_SmallDependenceHighGrayLevelEmphasis | 38.24 | 106.00 | 0.456 | 0.161 | 0.678 | 121 | -91.1 |
| wavelet-HLH_gldm_SmallDependenceLowGrayLevelEmphasis | 39.05 | 108.23 | 0.497 | 0.213 | 0.705 | 82.7 | -133.7 |
| wavelet-HLH_glrlm_GrayLevelNonUniformity | 14.23 | 39.44 | 0.99 | 0.979 | 0.995 | 63.4 | -15.5 |
| wavelet-HLH_glrlm_GrayLevelNonUniformityNormalized | 4.24 | 11.75 | 0.746 | 0.481 | 0.874 | 8.5 | -15 |
| wavelet-HLH_glrlm_GrayLevelVariance | 13.10 | 36.32 | 0.65 | 0.355 | 0.817 | 46.9 | -25.7 |
| wavelet-HLH_glrlm_HighGrayLevelRunEmphasis | 29.54 | 81.87 | 0.531 | 0.226 | 0.736 | 101.7 | -62.1 |
| wavelet-HLH_glrlm_LongRunEmphasis | 8.70 | 24.13 | 0.909 | 0.824 | 0.954 | 28.5 | -19.7 |
| wavelet-HLH_glrlm_LongRunHighGrayLevelEmphasis | 27.13 | 75.21 | 0.5 | 0.182 | 0.717 | 97.8 | -52.7 |
| wavelet-HLH_glrlm_LongRunLowGrayLevelEmphasis | 38.59 | 106.97 | 0.446 | 0.151 | 0.67 | 89.2 | -124.7 |
| wavelet-HLH_glrlm_LowGrayLevelRunEmphasis | 35.94 | 99.63 | 0.522 | 0.241 | 0.723 | 77.7 | -121.5 |
| wavelet-HLH_glrlm_RunEntropy | 2.57 | 7.12 | 0.825 | 0.112 | 0.945 | 11.5 | -2.8 |
| wavelet-HLH_glrlm_RunLengthNonUniformity | 18.06 | 50.05 | 0.988 | 0.97 | 0.994 | 74.1 | -26 |
| wavelet-HLH_glrlm_RunLengthNonUniformityNormalized | 6.09 | 16.88 | 0.797 | 0.631 | 0.892 | 14.1 | -19.7 |
| wavelet-HLH_glrlm_RunPercentage | 4.48 | 12.42 | 0.818 | 0.67 | 0.903 | 10.5 | -14.3 |
| wavelet-HLH_glrlm_RunVariance | 8.92 | 24.74 | 0.928 | 0.736 | 0.972 | 35.2 | -14.3 |
| wavelet-HLH_glrlm_ShortRunEmphasis | 5.15 | 14.26 | 0.771 | 0.598 | 0.876 | 14.3 | -14.3 |
| wavelet-HLH_glrlm_ShortRunHighGrayLevelEmphasis | 31.81 | 88.17 | 0.564 | 0.27 | 0.757 | 108.9 | -67.5 |
| wavelet-HLH_glrlm_ShortRunLowGrayLevelEmphasis | 36.00 | 99.78 | 0.572 | 0.302 | 0.756 | 77.9 | -121.7 |
| wavelet-HLH_glszm_GrayLevelNonUniformity | 34.88 | 96.67 | 0.968 | 0.937 | 0.983 | 106.7 | -86.7 |
| wavelet-HLH_glszm_GrayLevelNonUniformityNormalized | 23.72 | 65.74 | 0.545 | 0.271 | 0.738 | 57.8 | -73.7 |
| wavelet-HLH_glszm_GrayLevelVariance | 40.70 | 112.80 | 0.636 | 0.367 | 0.802 | 132.3 | -93.3 |
| wavelet-HLH_glszm_HighGrayLevelZoneEmphasis | 38.89 | 107.79 | 0.543 | 0.261 | 0.739 | 125.7 | -89.9 |
| wavelet-HLH_glszm_LargeAreaEmphasis | 39.80 | 110.33 | 0.995 | 0.989 | 0.997 | 143.4 | -77.2 |
| wavelet-HLH_glszm_LargeAreaHighGrayLevelEmphasis | 39.44 | 109.31 | 0.728 | 0.532 | 0.851 | 159.4 | -59.2 |
| wavelet-HLH_glszm_LargeAreaLowGrayLevelEmphasis | 58.56 | 162.33 | 0.493 | 0.203 | 0.704 | 171.2 | -153.5 |
| wavelet-HLH_glszm_LowGrayLevelZoneEmphasis | 34.91 | 96.78 | 0.418 | 0.116 | 0.651 | 82.8 | -110.7 |
| wavelet-HLH_glszm_SizeZoneNonUniformity | 21.97 | 60.90 | 0.972 | 0.946 | 0.986 | 56.3 | -65.5 |
| wavelet-HLH_glszm_SizeZoneNonUniformityNormalized | 30.05 | 83.30 | 0.372 | 0.072 | 0.616 | 60.1 | -106.5 |
| wavelet-HLH_glszm_SmallAreaEmphasis | 88.87 | 246.32 | 0.269 | -0.055 | 0.544 | 229.6 | -263 |
| wavelet-HLH_glszm_SmallAreaHighGrayLevelEmphasis | 93.13 | 258.15 | 0.303 | -0.022 | 0.571 | 266.7 | -249.6 |
| wavelet-HLH_glszm_SmallAreaLowGrayLevelEmphasis | 99.14 | 274.79 | 0 | -0.319 | 0.322 | 239.5 | -310.1 |
| wavelet-HLH_glszm_ZoneEntropy | 23.82 | 66.03 | 0.696 | 0.467 | 0.835 | 75.4 | -56.6 |
| wavelet-HLH_glszm_ZonePercentage | 32.41 | 89.82 | 0.612 | 0.361 | 0.781 | 80.3 | -99.3 |
| wavelet-HLH_glszm_ZoneVariance | 54.23 | 150.32 | 0.995 | 0.991 | 0.998 | 189.6 | -111.1 |
| wavelet-HLH_ngtdm_Busyness | 48.56 | 134.60 | 0.694 | 0.48 | 0.831 | 127.2 | -142 |
| wavelet-HLH_ngtdm_Coarseness | 17.05 | 47.25 | 0.852 | 0.518 | 0.941 | 19.6 | -74.9 |
| wavelet-HLH_ngtdm_Complexity | 43.69 | 121.10 | 0.527 | 0.23 | 0.731 | 153.2 | -89 |
| wavelet-HLH_ngtdm_Contrast | 38.69 | 107.25 | 0.614 | 0.366 | 0.781 | 86.9 | -127.6 |
| wavelet-HLH_ngtdm_Strength | 44.12 | 122.29 | 0.548 | 0.274 | 0.74 | 128.4 | -116.2 |
| wavelet-HHL_firstorder_10Percentile | 20.22 | 56.05 | 0.853 | 0.346 | 0.949 | 82.1 | -30 |
| wavelet-HHL_firstorder_90Percentile | 19.80 | 54.89 | 0.905 | 0.792 | 0.954 | 67 | -42.8 |
| wavelet-HHL_firstorder_Energy | 35.43 | 98.20 | 0.581 | 0.316 | 0.762 | 162.1 | -34.3 |
| wavelet-HHL_firstorder_Entropy | 5.85 | 16.21 | 0.85 | 0.474 | 0.942 | 23.2 | -9.2 |
| wavelet-HHL_firstorder_InterquartileRange | 20.41 | 56.57 | 0.905 | 0.79 | 0.954 | 69.5 | -43.7 |
| wavelet-HHL_firstorder_Kurtosis | 17.52 | 48.58 | 0.678 | 0.402 | 0.832 | 64.1 | -33.1 |
| wavelet-HHL_firstorder_Maximum | 23.40 | 64.87 | 0.75 | 0.328 | 0.894 | 94.2 | -35.6 |
| wavelet-HHL_firstorder_MeanAbsoluteDeviation | 19.35 | 53.65 | 0.889 | 0.623 | 0.956 | 71.4 | -35.9 |
| wavelet-HHL_firstorder_Mean | 120.00 | 332.62 | 0.685 | 0.356 | 0.845 | 303.6 | -361.6 |
| wavelet-HHL_firstorder_Median | 431.63 | 1196.43 | 0.726 | 0.526 | 0.85 | 1149.3 | -1243.6 |
| wavelet-HHL_firstorder_Minimum | 19.74 | 54.72 | 0.761 | 0.383 | 0.896 | 84.7 | -24.7 |
| wavelet-HHL_firstorder_Range | 20.85 | 57.79 | 0.77 | 0.323 | 0.907 | 87.5 | -28.1 |
| wavelet-HHL_firstorder_RobustMeanAbsoluteDeviation | 20.16 | 55.88 | 0.902 | 0.762 | 0.955 | 69.8 | -41.9 |
| wavelet-HHL_firstorder_RootMeanSquared | 18.94 | 52.50 | 0.867 | 0.514 | 0.949 | 72.1 | -32.9 |
| wavelet-HHL_firstorder_Skewness | 32.58 | 90.31 | 0.651 | 0.391 | 0.81 | 108.8 | -71.8 |
| wavelet-HHL_firstorder_TotalEnergy | 35.43 | 98.20 | 0.581 | 0.316 | 0.762 | 162.1 | -34.3 |
| wavelet-HHL_firstorder_Uniformity | 3.76 | 10.43 | 0.831 | 0.498 | 0.931 | 6.3 | -14.6 |
| wavelet-HHL_firstorder_Variance | 34.36 | 95.24 | 0.799 | 0.52 | 0.908 | 134.8 | -55.7 |
| wavelet-HHL_glcm_Autocorrelation | 31.62 | 87.66 | 0.57 | 0.295 | 0.756 | 108.5 | -66.8 |
| wavelet-HHL_glcm_ClusterProminence | 36.66 | 101.62 | 0.386 | 0.085 | 0.627 | 139.4 | -63.9 |
| wavelet-HHL_glcm_ClusterShade | 234.58 | 650.21 | 0.614 | 0.342 | 0.787 | 661.8 | -638.6 |
| wavelet-HHL_glcm_ClusterTendency | 13.82 | 38.31 | 0.754 | 0.487 | 0.879 | 53.5 | -23.1 |
| wavelet-HHL_glcm_Contrast | 12.10 | 33.55 | 0.811 | 0.57 | 0.911 | 44.5 | -22.6 |
| wavelet-HHL_glcm_Correlation | 7.25 | 20.11 | 0.736 | 0.527 | 0.859 | 24.1 | -16.1 |
| wavelet-HHL_glcm_DifferenceAverage | 9.89 | 27.41 | 0.833 | 0.614 | 0.922 | 34.9 | -19.9 |
| wavelet-HHL_glcm_DifferenceEntropy | 5.82 | 16.14 | 0.816 | 0.522 | 0.919 | 21.5 | -10.8 |
| wavelet-HHL_glcm_DifferenceVariance | 9.99 | 27.70 | 0.746 | 0.482 | 0.874 | 37.1 | -18.3 |
| wavelet-HHL_glcm_Id | 1.71 | 4.73 | 0.809 | 0.61 | 0.905 | 3.5 | -5.9 |
| wavelet-HHL_glcm_Idm | 1.77 | 4.91 | 0.822 | 0.619 | 0.913 | 3.6 | -6.2 |
| wavelet-HHL_glcm_Idmn | 1.22 | 3.38 | 0.408 | 0.106 | 0.644 | 4.1 | -2.6 |
| wavelet-HHL_glcm_Idn | 1.32 | 3.65 | 0.313 | 0.008 | 0.571 | 4.3 | -3 |
| wavelet-HHL_glcm_Imc1 | 10.64 | 29.48 | 0.667 | 0.439 | 0.814 | 31.3 | -27.6 |
| wavelet-HHL_glcm_Imc2 | 6.25 | 17.32 | 0.821 | 0.632 | 0.911 | 21.5 | -13.2 |
| wavelet-HHL_glcm_InverseVariance | 9.11 | 25.26 | 0.688 | 0.467 | 0.828 | 29.5 | -21 |
| wavelet-HHL_glcm_JointAverage | 17.20 | 47.67 | 0.603 | 0.322 | 0.781 | 58.8 | -36.6 |
| wavelet-HHL_glcm_JointEnergy | 8.80 | 24.38 | 0.739 | 0.465 | 0.871 | 16.1 | -32.7 |
| wavelet-HHL_glcm_JointEntropy | 7.33 | 20.31 | 0.84 | 0.493 | 0.936 | 28.1 | -12.5 |
| wavelet-HHL_glcm_MCC | 6.38 | 17.67 | 0.812 | 0.649 | 0.902 | 21 | -14.3 |
| wavelet-HHL_glcm_MaximumProbability | 8.43 | 23.36 | 0.709 | 0.473 | 0.845 | 17.9 | -28.8 |
| wavelet-HHL_glcm_SumAverage | 17.20 | 47.67 | 0.603 | 0.322 | 0.781 | 58.8 | -36.6 |
| wavelet-HHL_glcm_SumEntropy | 6.61 | 18.32 | 0.829 | 0.458 | 0.931 | 25.4 | -11.2 |
| wavelet-HHL_glcm_SumSquares | 12.98 | 35.99 | 0.768 | 0.503 | 0.888 | 50.1 | -21.9 |
| wavelet-HHL_gldm_DependenceEntropy | 1.83 | 5.06 | 0.725 | 0.302 | 0.88 | 7 | -3.1 |
| wavelet-HHL_gldm_DependenceNonUniformity | 14.75 | 40.89 | 0.986 | 0.972 | 0.993 | 67.5 | -14.3 |
| wavelet-HHL_gldm_DependenceNonUniformityNormalized | 6.89 | 19.10 | 0.43 | 0.128 | 0.661 | 16.7 | -21.5 |
| wavelet-HHL_gldm_DependenceVariance | 10.67 | 29.57 | 0.474 | 0.18 | 0.691 | 30 | -29.1 |
| wavelet-HHL_gldm_GrayLevelNonUniformity | 16.47 | 45.66 | 0.988 | 0.976 | 0.994 | 70.6 | -20.8 |
| wavelet-HHL_gldm_GrayLevelVariance | 11.32 | 31.39 | 0.755 | 0.488 | 0.88 | 43.9 | -18.8 |
| wavelet-HHL_gldm_HighGrayLevelEmphasis | 31.11 | 86.22 | 0.567 | 0.292 | 0.755 | 106.7 | -65.7 |
| wavelet-HHL_gldm_LargeDependenceEmphasis | 8.41 | 23.31 | 0.851 | 0.729 | 0.921 | 24.5 | -22.1 |
| wavelet-HHL_gldm_LargeDependenceHighGrayLevelEmphasis | 30.81 | 85.40 | 0.453 | 0.154 | 0.677 | 106.1 | -64.7 |
| wavelet-HHL_gldm_LargeDependenceLowGrayLevelEmphasis | 40.33 | 111.78 | 0.442 | 0.149 | 0.667 | 90.7 | -132.9 |
| wavelet-HHL_gldm_LowGrayLevelEmphasis | 38.12 | 105.66 | 0.438 | 0.143 | 0.664 | 83.3 | -128 |
| wavelet-HHL_gldm_SmallDependenceEmphasis | 16.64 | 46.11 | 0.716 | 0.512 | 0.844 | 43.7 | -48.5 |
| wavelet-HHL_gldm_SmallDependenceHighGrayLevelEmphasis | 40.69 | 112.78 | 0.559 | 0.292 | 0.746 | 136.2 | -89.3 |
| wavelet-HHL_gldm_SmallDependenceLowGrayLevelEmphasis | 30.83 | 85.47 | 0.728 | 0.48 | 0.86 | 59.3 | -111.6 |
| wavelet-HHL_glrlm_GrayLevelNonUniformity | 13.75 | 38.10 | 0.99 | 0.979 | 0.995 | 61.5 | -14.7 |
| wavelet-HHL_glrlm_GrayLevelNonUniformityNormalized | 4.47 | 12.39 | 0.853 | 0.477 | 0.943 | 7.3 | -17.4 |
| wavelet-HHL_glrlm_GrayLevelVariance | 14.12 | 39.13 | 0.707 | 0.429 | 0.851 | 54.3 | -24 |
| wavelet-HHL_glrlm_HighGrayLevelRunEmphasis | 31.21 | 86.52 | 0.582 | 0.308 | 0.765 | 108.1 | -64.9 |
| wavelet-HHL_glrlm_LongRunEmphasis | 10.33 | 28.62 | 0.904 | 0.82 | 0.949 | 31.6 | -25.7 |
| wavelet-HHL_glrlm_LongRunHighGrayLevelEmphasis | 29.05 | 80.53 | 0.484 | 0.175 | 0.703 | 103.3 | -57.7 |
| wavelet-HHL_glrlm_LongRunLowGrayLevelEmphasis | 41.70 | 115.58 | 0.471 | 0.182 | 0.687 | 96.1 | -135 |
| wavelet-HHL_glrlm_LowGrayLevelRunEmphasis | 37.82 | 104.83 | 0.489 | 0.2 | 0.701 | 82.3 | -127.4 |
| wavelet-HHL_glrlm_RunEntropy | 2.88 | 8.00 | 0.816 | 0.141 | 0.94 | 12.7 | -3.3 |
| wavelet-HHL_glrlm_RunLengthNonUniformity | 15.00 | 41.58 | 0.985 | 0.963 | 0.993 | 68.8 | -14.4 |
| wavelet-HHL_glrlm_RunLengthNonUniformityNormalized | 6.62 | 18.34 | 0.858 | 0.741 | 0.925 | 17.3 | -19.3 |
| wavelet-HHL_glrlm_RunPercentage | 4.88 | 13.52 | 0.861 | 0.746 | 0.926 | 12.8 | -14.3 |
| wavelet-HHL_glrlm_RunVariance | 12.99 | 36.00 | 0.916 | 0.805 | 0.961 | 44.1 | -27.9 |
| wavelet-HHL_glrlm_ShortRunEmphasis | 5.28 | 14.64 | 0.851 | 0.729 | 0.921 | 15.2 | -14.1 |
| wavelet-HHL_glrlm_ShortRunHighGrayLevelEmphasis | 33.55 | 93.00 | 0.624 | 0.368 | 0.791 | 116.9 | -69.1 |
| wavelet-HHL_glrlm_ShortRunLowGrayLevelEmphasis | 36.13 | 100.15 | 0.593 | 0.321 | 0.772 | 77.6 | -122.7 |
| wavelet-HHL_glszm_GrayLevelNonUniformity | 23.72 | 65.74 | 0.949 | 0.902 | 0.973 | 72.3 | -59.2 |
| wavelet-HHL_glszm_GrayLevelNonUniformityNormalized | 21.89 | 60.67 | 0.533 | 0.228 | 0.737 | 43 | -78.3 |
| wavelet-HHL_glszm_GrayLevelVariance | 43.87 | 121.59 | 0.665 | 0.341 | 0.831 | 163.4 | -79.8 |
| wavelet-HHL_glszm_HighGrayLevelZoneEmphasis | 35.00 | 97.03 | 0.533 | 0.229 | 0.737 | 129.5 | -64.5 |
| wavelet-HHL_glszm_LargeAreaEmphasis | 37.13 | 102.92 | 0.96 | 0.924 | 0.98 | 133 | -72.8 |
| wavelet-HHL_glszm_LargeAreaHighGrayLevelEmphasis | 40.13 | 111.23 | 0.533 | 0.257 | 0.73 | 158.9 | -63.5 |
| wavelet-HHL_glszm_LargeAreaLowGrayLevelEmphasis | 58.43 | 161.96 | 0.669 | 0.443 | 0.816 | 168.2 | -155.7 |
| wavelet-HHL_glszm_LowGrayLevelZoneEmphasis | 30.97 | 85.83 | 0.549 | 0.275 | 0.741 | 69.5 | -102.2 |
| wavelet-HHL_glszm_SizeZoneNonUniformity | 18.88 | 52.33 | 0.978 | 0.957 | 0.988 | 56.2 | -48.5 |
| wavelet-HHL_glszm_SizeZoneNonUniformityNormalized | 25.25 | 70.00 | 0.511 | 0.22 | 0.718 | 49.9 | -90.1 |
| wavelet-HHL_glszm_SmallAreaEmphasis | 88.51 | 245.32 | 0.329 | 0.007 | 0.59 | 242.1 | -248.5 |
| wavelet-HHL_glszm_SmallAreaHighGrayLevelEmphasis | 97.56 | 270.42 | 0.174 | -0.128 | 0.458 | 293.4 | -247.4 |
| wavelet-HHL_glszm_SmallAreaLowGrayLevelEmphasis | 94.24 | 261.21 | 0.237 | -0.075 | 0.514 | 237.5 | -284.9 |
| wavelet-HHL_glszm_ZoneEntropy | 18.04 | 50.01 | 0.752 | 0.444 | 0.884 | 62.1 | -37.9 |
| wavelet-HHL_glszm_ZonePercentage | 26.15 | 72.49 | 0.781 | 0.613 | 0.881 | 68 | -77 |
| wavelet-HHL_glszm_ZoneVariance | 44.50 | 123.35 | 0.96 | 0.924 | 0.98 | 165.6 | -81.1 |
| wavelet-HHL_ngtdm_Busyness | 56.85 | 157.57 | 0.683 | 0.464 | 0.824 | 134.6 | -180.6 |
| wavelet-HHL_ngtdm_Coarseness | 15.41 | 42.72 | 0.817 | 0.433 | 0.927 | 15.4 | -70.1 |
| wavelet-HHL_ngtdm_Complexity | 50.65 | 140.39 | 0.556 | 0.271 | 0.749 | 190.8 | -89.9 |
| wavelet-HHL_ngtdm_Contrast | 44.50 | 123.35 | 0.309 | 0.006 | 0.568 | 92.5 | -154.2 |
| wavelet-HHL_ngtdm_Strength | 50.20 | 139.14 | 0.619 | 0.371 | 0.785 | 162 | -116.3 |
| wavelet-HHH_firstorder_10Percentile | 19.49 | 54.03 | 0.895 | 0.75 | 0.952 | 65.9 | -42.2 |
| wavelet-HHH_firstorder_90Percentile | 17.36 | 48.12 | 0.877 | 0.33 | 0.96 | 69.9 | -26.3 |
| wavelet-HHH_firstorder_Energy | 35.09 | 97.26 | 0.656 | 0.401 | 0.813 | 157.1 | -37.4 |
| wavelet-HHH_firstorder_Entropy | 0.31 | 0.85 | 0.249 | -0.081 | 0.53 | 0.8 | -0.9 |
| wavelet-HHH_firstorder_InterquartileRange | 17.72 | 49.12 | 0.892 | 0.585 | 0.96 | 64.6 | -33.6 |
| wavelet-HHH_firstorder_Kurtosis | 17.17 | 47.59 | 0.747 | 0.56 | 0.862 | 51.4 | -43.7 |
| wavelet-HHH_firstorder_Maximum | 22.12 | 61.33 | 0.766 | 0.386 | 0.899 | 87.7 | -34.9 |
| wavelet-HHH_firstorder_MeanAbsoluteDeviation | 17.85 | 49.49 | 0.885 | 0.552 | 0.957 | 66.1 | -32.9 |
| wavelet-HHH_firstorder_Mean | 137.06 | 379.92 | 0.541 | 0.218 | 0.748 | 341 | -418.9 |
| wavelet-HHH_firstorder_Median | 647.92 | 1795.94 | 0.37 | 0.054 | 0.619 | 1691.3 | -1900.6 |
| wavelet-HHH_firstorder_Minimum | 21.62 | 59.94 | 0.805 | 0.498 | 0.914 | 80.5 | -39.4 |
| wavelet-HHH_firstorder_Range | 20.97 | 58.14 | 0.797 | 0.44 | 0.914 | 80.9 | -35.4 |
| wavelet-HHH_firstorder_RobustMeanAbsoluteDeviation | 18.12 | 50.22 | 0.89 | 0.582 | 0.959 | 66 | -34.5 |
| wavelet-HHH_firstorder_RootMeanSquared | 17.99 | 49.85 | 0.871 | 0.546 | 0.95 | 67.1 | -32.6 |
| wavelet-HHH_firstorder_Skewness | 31.88 | 88.36 | 0.582 | 0.32 | 0.762 | 72.8 | -103.9 |
| wavelet-HHH_firstorder_TotalEnergy | 35.09 | 97.26 | 0.656 | 0.401 | 0.813 | 157.1 | -37.4 |
| wavelet-HHH_firstorder_Uniformity | 0.42 | 1.15 | 0.249 | -0.08 | 0.53 | 1.2 | -1.1 |
| wavelet-HHH_firstorder_Variance | 32.46 | 89.98 | 0.833 | 0.534 | 0.929 | 123.5 | -56.5 |
| wavelet-HHH_glcm_Autocorrelation | 2.93 | 8.13 | 0.397 | 0.086 | 0.638 | 8.3 | -7.9 |
| wavelet-HHH_glcm_ClusterProminence | 2.00 | 5.55 | 0.252 | -0.077 | 0.532 | 5.5 | -5.6 |
| wavelet-HHH_glcm_ClusterShade | 772.24 | 2140.55 | 0.372 | 0.057 | 0.621 | 1977.4 | -2303.7 |
| wavelet-HHH_glcm_ClusterTendency | 2.14 | 5.94 | 0.289 | -0.037 | 0.56 | 5.9 | -6 |
| wavelet-HHH_glcm_Contrast | 4.03 | 11.17 | 0.223 | -0.108 | 0.51 | 11.2 | -11.2 |
| wavelet-HHH_glcm_Correlation | 7.97 | 22.09 | 0.255 | -0.074 | 0.534 | 22.1 | -22.1 |
| wavelet-HHH_glcm_DifferenceAverage | 4.03 | 11.17 | 0.223 | -0.108 | 0.51 | 11.2 | -11.2 |
| wavelet-HHH_glcm_DifferenceEntropy | 1.85 | 5.13 | 0.633 | 0.393 | 0.793 | 5.7 | -4.5 |
| wavelet-HHH_glcm_DifferenceVariance | 2.43 | 6.73 | 0.635 | 0.395 | 0.794 | 7.5 | -5.9 |
| wavelet-HHH_glcm_Id | 0.79 | 2.20 | 0.223 | -0.108 | 0.51 | 2.2 | -2.2 |
| wavelet-HHH_glcm_Idm | 0.79 | 2.20 | 0.223 | -0.108 | 0.51 | 2.2 | -2.2 |
| wavelet-HHH_glcm_Idmn | 0.28 | 0.79 | 0.223 | -0.108 | 0.51 | 0.8 | -0.8 |
| wavelet-HHH_glcm_Idn | 0.50 | 1.38 | 0.223 | -0.108 | 0.51 | 1.4 | -1.4 |
| wavelet-HHH_glcm_Imc1 | 12.28 | 34.05 | 0.633 | 0.393 | 0.793 | 30.2 | -37.9 |
| wavelet-HHH_glcm_Imc2 | 6.49 | 17.99 | 0.481 | 0.188 | 0.696 | 17 | -18.9 |
| wavelet-HHH_glcm_InverseVariance | 4.03 | 11.17 | 0.223 | -0.108 | 0.51 | 11.2 | -11.2 |
| wavelet-HHH_glcm_JointAverage | 1.50 | 4.16 | 0.376 | 0.061 | 0.623 | 4.3 | -4 |
| wavelet-HHH_glcm_JointEnergy | 1.99 | 5.50 | 0.558 | 0.288 | 0.747 | 5.1 | -6 |
| wavelet-HHH_glcm_JointEntropy | 0.94 | 2.61 | 0.585 | 0.325 | 0.764 | 2.9 | -2.4 |
| wavelet-HHH_glcm_MCC | 6.97 | 19.31 | 0.515 | 0.231 | 0.718 | 18.1 | -20.6 |
| wavelet-HHH_glcm_MaximumProbability | 5.06 | 14.03 | 0.172 | -0.16 | 0.469 | 13.5 | -14.5 |
| wavelet-HHH_glcm_SumAverage | 1.50 | 4.16 | 0.376 | 0.061 | 0.623 | 4.3 | -4 |
| wavelet-HHH_glcm_SumEntropy | 0.90 | 2.48 | 0.641 | 0.404 | 0.798 | 2.8 | -2.2 |
| wavelet-HHH_glcm_SumSquares | 0.59 | 1.65 | 0.207 | -0.125 | 0.497 | 1.6 | -1.7 |
| wavelet-HHH_gldm_DependenceEntropy | 1.08 | 3.00 | 0.457 | 0.158 | 0.68 | 3.2 | -2.8 |
| wavelet-HHH_gldm_DependenceNonUniformity | 15.50 | 42.98 | 0.985 | 0.969 | 0.992 | 71.4 | -14.6 |
| wavelet-HHH_gldm_DependenceNonUniformityNormalized | 2.96 | 8.20 | 0.606 | 0.353 | 0.777 | 7.6 | -8.8 |
| wavelet-HHH_gldm_DependenceVariance | 5.03 | 13.95 | 0.621 | 0.374 | 0.786 | 14.3 | -13.6 |
| wavelet-HHH_gldm_GrayLevelNonUniformity | 15.70 | 43.51 | 0.987 | 0.974 | 0.994 | 72.5 | -14.5 |
| wavelet-HHH_gldm_GrayLevelVariance | 0.43 | 1.18 | 0.249 | -0.08 | 0.53 | 1.1 | -1.3 |
| wavelet-HHH_gldm_HighGrayLevelEmphasis | 2.61 | 7.24 | 0.414 | 0.106 | 0.65 | 7.5 | -7 |
| wavelet-HHH_gldm_LargeDependenceEmphasis | 4.26 | 11.82 | 0.851 | 0.351 | 0.948 | 17.3 | -6.3 |
| wavelet-HHH_gldm_LargeDependenceHighGrayLevelEmphasis | 7.02 | 19.45 | 0.817 | 0.529 | 0.919 | 25.1 | -13.7 |
| wavelet-HHH_gldm_LargeDependenceLowGrayLevelEmphasis | 6.05 | 16.77 | 0.726 | 0.41 | 0.869 | 22 | -11.5 |
| wavelet-HHH_gldm_LowGrayLevelEmphasis | 2.58 | 7.16 | 0.414 | 0.106 | 0.65 | 6.9 | -7.4 |
| wavelet-HHH_gldm_SmallDependenceEmphasis | 18.03 | 49.97 | 0.469 | 0.172 | 0.689 | 36.4 | -63.6 |
| wavelet-HHH_gldm_SmallDependenceHighGrayLevelEmphasis | 20.69 | 57.35 | 0.44 | 0.146 | 0.666 | 44.2 | -70.5 |
| wavelet-HHH_gldm_SmallDependenceLowGrayLevelEmphasis | 17.91 | 49.64 | 0.452 | 0.151 | 0.677 | 36.3 | -63 |
| wavelet-HHH_glrlm_GrayLevelNonUniformity | 14.45 | 40.06 | 0.989 | 0.977 | 0.995 | 65.6 | -14.5 |
| wavelet-HHH_glrlm_GrayLevelNonUniformityNormalized | 0.26 | 0.72 | 0.388 | 0.076 | 0.632 | 0.7 | -0.8 |
| wavelet-HHH_glrlm_GrayLevelVariance | 0.26 | 0.73 | 0.388 | 0.076 | 0.632 | 0.8 | -0.7 |
| wavelet-HHH_glrlm_HighGrayLevelRunEmphasis | 2.23 | 6.17 | 0.4 | 0.09 | 0.641 | 6.2 | -6.1 |
| wavelet-HHH_glrlm_LongRunEmphasis | 5.70 | 15.79 | 0.866 | 0.381 | 0.954 | 23.4 | -8.2 |
| wavelet-HHH_glrlm_LongRunHighGrayLevelEmphasis | 7.24 | 20.08 | 0.857 | 0.454 | 0.947 | 28 | -12.1 |
| wavelet-HHH_glrlm_LongRunLowGrayLevelEmphasis | 6.47 | 17.94 | 0.831 | 0.455 | 0.933 | 25.1 | -10.8 |
| wavelet-HHH_glrlm_LowGrayLevelRunEmphasis | 2.26 | 6.26 | 0.4 | 0.09 | 0.641 | 6.2 | -6.3 |
| wavelet-HHH_glrlm_RunEntropy | 1.74 | 4.81 | 0.873 | 0.325 | 0.959 | 7.4 | -2.3 |
| wavelet-HHH_glrlm_RunLengthNonUniformity | 13.43 | 37.22 | 0.991 | 0.982 | 0.996 | 58.3 | -16.2 |
| wavelet-HHH_glrlm_RunLengthNonUniformityNormalized | 3.51 | 9.73 | 0.835 | 0.433 | 0.936 | 5.4 | -14.1 |
| wavelet-HHH_glrlm_RunPercentage | 2.58 | 7.15 | 0.854 | 0.39 | 0.948 | 3.6 | -10.7 |
| wavelet-HHH_glrlm_RunVariance | 7.28 | 20.17 | 0.885 | 0.334 | 0.963 | 31.1 | -9.3 |
| wavelet-HHH_glrlm_ShortRunEmphasis | 3.20 | 8.86 | 0.794 | 0.469 | 0.909 | 5.6 | -12.1 |
| wavelet-HHH_glrlm_ShortRunHighGrayLevelEmphasis | 4.70 | 13.03 | 0.709 | 0.456 | 0.849 | 9.5 | -16.5 |
| wavelet-HHH_glrlm_ShortRunLowGrayLevelEmphasis | 4.63 | 12.84 | 0.675 | 0.422 | 0.825 | 9.9 | -15.7 |
| wavelet-HHH_glszm_GrayLevelNonUniformity | 46.73 | 129.52 | 0.946 | 0.898 | 0.972 | 121.1 | -137.9 |
| wavelet-HHH_glszm_GrayLevelNonUniformityNormalized | 11.21 | 31.07 | 0.272 | -0.027 | 0.537 | 24.4 | -37.7 |
| wavelet-HHH_glszm_GrayLevelVariance | 19.40 | 53.78 | 0.272 | -0.027 | 0.537 | 64.3 | -43.2 |
| wavelet-HHH_glszm_HighGrayLevelZoneEmphasis | 19.25 | 53.35 | 0.313 | -0.011 | 0.578 | 54.5 | -52.2 |
| wavelet-HHH_glszm_LargeAreaEmphasis | 39.90 | 110.60 | 0.904 | 0.822 | 0.95 | 166.3 | -54.9 |
| wavelet-HHH_glszm_LargeAreaHighGrayLevelEmphasis | 40.50 | 112.27 | 0.901 | 0.815 | 0.948 | 168.3 | -56.3 |
| wavelet-HHH_glszm_LargeAreaLowGrayLevelEmphasis | 39.98 | 110.82 | 0.909 | 0.829 | 0.952 | 166 | -55.6 |
| wavelet-HHH_glszm_LowGrayLevelZoneEmphasis | 19.37 | 53.70 | 0.313 | -0.011 | 0.578 | 55.6 | -51.8 |
| wavelet-HHH_glszm_SizeZoneNonUniformity | 38.62 | 107.06 | 0.936 | 0.88 | 0.967 | 113.2 | -100.9 |
| wavelet-HHH_glszm_SizeZoneNonUniformityNormalized | 26.79 | 74.26 | 0.04 | -0.276 | 0.354 | 83.2 | -65.3 |
| wavelet-HHH_glszm_SmallAreaEmphasis | 83.85 | 232.41 | 0.361 | 0.044 | 0.613 | 201 | -263.8 |
| wavelet-HHH_glszm_SmallAreaHighGrayLevelEmphasis | 90.11 | 249.78 | 0.188 | -0.144 | 0.482 | 220.4 | -279.1 |
| wavelet-HHH_glszm_SmallAreaLowGrayLevelEmphasis | 90.51 | 250.87 | 0.466 | 0.169 | 0.686 | 221.2 | -280.5 |
| wavelet-HHH_glszm_ZoneEntropy | 27.51 | 76.25 | 0.088 | -0.243 | 0.401 | 71.7 | -80.8 |
| wavelet-HHH_glszm_ZonePercentage | 39.01 | 108.14 | 0.355 | 0.054 | 0.603 | 78.3 | -138 |
| wavelet-HHH_glszm_ZoneVariance | 86.83 | 240.67 | 0.907 | 0.826 | 0.951 | 254.9 | -226.4 |
| wavelet-HHH_ngtdm_Busyness | 18.93 | 52.47 | 0.991 | 0.98 | 0.995 | 80.6 | -24.3 |
| wavelet-HHH_ngtdm_Coarseness | 16.30 | 45.19 | 0.839 | 0.479 | 0.936 | 16.3 | -74.1 |
| wavelet-HHH_ngtdm_Complexity | 4.54 | 12.59 | 0.096 | -0.235 | 0.408 | 12.6 | -12.6 |
| wavelet-HHH_ngtdm_Contrast | 4.64 | 12.86 | 0.071 | -0.259 | 0.386 | 12.8 | -12.9 |
| wavelet-HHH_ngtdm_Strength | 16.25 | 45.04 | 0.839 | 0.472 | 0.937 | 16.1 | -74 |
| wavelet-LLL_firstorder_10Percentile | 5.77 | 16.00 | 0.767 | 0.591 | 0.874 | 17.7 | -14.3 |
| wavelet-LLL_firstorder_90Percentile | 12.62 | 34.99 | 0.729 | 0.123 | 0.898 | 58.3 | -11.7 |
| wavelet-LLL_firstorder_Energy | 29.94 | 82.98 | 0.73 | 0.527 | 0.854 | 146.8 | -19.2 |
| wavelet-LLL_firstorder_Entropy | 3.28 | 9.08 | 0.864 | 0.179 | 0.959 | 14.1 | -4 |
| wavelet-LLL_firstorder_InterquartileRange | 18.05 | 50.02 | 0.705 | 0.158 | 0.882 | 78.2 | -21.9 |
| wavelet-LLL_firstorder_Kurtosis | 9.99 | 27.70 | 0.764 | 0.423 | 0.894 | 40.3 | -15 |
| wavelet-LLL_firstorder_Maximum | 15.53 | 43.06 | 0.675 | 0.152 | 0.864 | 74.5 | -11.6 |
| wavelet-LLL_firstorder_MeanAbsoluteDeviation | 17.49 | 48.48 | 0.711 | 0.116 | 0.889 | 80.2 | -16.7 |
| wavelet-LLL_firstorder_Mean | 9.39 | 26.03 | 0.761 | 0.145 | 0.914 | 41.7 | -10.3 |
| wavelet-LLL_firstorder_Median | 7.68 | 21.28 | 0.809 | 0.341 | 0.927 | 31.2 | -11.3 |
| wavelet-LLL_firstorder_Minimum | 10.69 | 29.64 | 0.743 | 0.519 | 0.866 | 22.6 | -36.7 |
| wavelet-LLL_firstorder_Range | 18.03 | 49.97 | 0.678 | 0.144 | 0.867 | 85.7 | -14.3 |
| wavelet-LLL_firstorder_RobustMeanAbsoluteDeviation | 17.50 | 48.50 | 0.711 | 0.133 | 0.887 | 78.2 | -18.8 |
| wavelet-LLL_firstorder_RootMeanSquared | 11.12 | 30.82 | 0.728 | 0.136 | 0.897 | 50.3 | -11.3 |
| wavelet-LLL_firstorder_Skewness | 25.91 | 71.81 | 0.81 | 0.134 | 0.938 | 98 | -45.6 |
| wavelet-LLL_firstorder_TotalEnergy | 29.94 | 82.98 | 0.73 | 0.527 | 0.854 | 146.8 | -19.2 |
| wavelet-LLL_firstorder_Uniformity | 14.29 | 39.61 | 0.884 | 0.74 | 0.945 | 17.5 | -61.7 |
| wavelet-LLL_firstorder_Variance | 33.00 | 91.47 | 0.509 | 0.157 | 0.732 | 154.3 | -28.6 |
| wavelet-LLL_glcm_Autocorrelation | 28.52 | 79.04 | 0.583 | 0.155 | 0.797 | 129.4 | -28.7 |
| wavelet-LLL_glcm_ClusterProminence | 56.30 | 156.04 | 0.142 | -0.164 | 0.435 | 272.5 | -39.6 |
| wavelet-LLL_glcm_ClusterShade | 56.52 | 156.67 | 0.247 | -0.056 | 0.518 | 268.3 | -45 |
| wavelet-LLL_glcm_ClusterTendency | 33.77 | 93.61 | 0.498 | 0.156 | 0.722 | 160.4 | -26.8 |
| wavelet-LLL_glcm_Contrast | 27.84 | 77.16 | 0.656 | 0.249 | 0.838 | 124.6 | -29.7 |
| wavelet-LLL_glcm_Correlation | 5.16 | 14.29 | 0.825 | 0.197 | 0.942 | 21.2 | -7.4 |
| wavelet-LLL_glcm_DifferenceAverage | 14.41 | 39.93 | 0.805 | 0.148 | 0.935 | 62.6 | -17.2 |
| wavelet-LLL_glcm_DifferenceEntropy | 3.84 | 10.64 | 0.854 | 0.1 | 0.957 | 17 | -4.3 |
| wavelet-LLL_glcm_DifferenceVariance | 28.72 | 79.60 | 0.575 | 0.227 | 0.776 | 133.1 | -26.1 |
| wavelet-LLL_glcm_Id | 10.43 | 28.92 | 0.867 | 0.678 | 0.939 | 16 | -41.8 |
| wavelet-LLL_glcm_Idm | 14.76 | 40.92 | 0.849 | 0.709 | 0.922 | 25.3 | -56.5 |
| wavelet-LLL_glcm_Idmn | 0.34 | 0.95 | 0.829 | 0.308 | 0.939 | 1.4 | -0.5 |
| wavelet-LLL_glcm_Idn | 0.79 | 2.20 | 0.838 | 0.202 | 0.947 | 3.5 | -0.9 |
| wavelet-LLL_glcm_Imc1 | 7.10 | 19.68 | 0.934 | 0.875 | 0.966 | 21.8 | -17.5 |
| wavelet-LLL_glcm_Imc2 | 0.32 | 0.89 | 0.856 | 0.738 | 0.924 | 1 | -0.8 |
| wavelet-LLL_glcm_InverseVariance | 13.94 | 38.64 | 0.871 | 0.734 | 0.936 | 23 | -54.2 |
| wavelet-LLL_glcm_JointAverage | 13.92 | 38.60 | 0.765 | 0.086 | 0.92 | 61.3 | -15.9 |
| wavelet-LLL_glcm_JointEnergy | 18.05 | 50.03 | 0.85 | 0.619 | 0.933 | 15 | -85 |
| wavelet-LLL_glcm_JointEntropy | 2.72 | 7.54 | 0.916 | 0.211 | 0.977 | 12.6 | -2.5 |
| wavelet-LLL_glcm_MCC | 3.88 | 10.74 | 0.767 | 0.592 | 0.874 | 12 | -9.5 |
| wavelet-LLL_glcm_MaximumProbability | 17.15 | 47.54 | 0.85 | 0.696 | 0.925 | 18.3 | -76.8 |
| wavelet-LLL_glcm_SumAverage | 13.92 | 38.60 | 0.765 | 0.086 | 0.92 | 61.3 | -15.9 |
| wavelet-LLL_glcm_SumEntropy | 3.01 | 8.36 | 0.865 | 0.048 | 0.963 | 14.1 | -2.6 |
| wavelet-LLL_glcm_SumSquares | 32.91 | 91.21 | 0.51 | 0.163 | 0.731 | 155.7 | -26.7 |
| wavelet-LLL_gldm_DependenceEntropy | 2.02 | 5.60 | 0.886 | 0.06 | 0.97 | 9.9 | -1.3 |
| wavelet-LLL_gldm_DependenceNonUniformity | 17.72 | 49.12 | 0.93 | 0.866 | 0.964 | 82.5 | -15.8 |
| wavelet-LLL_gldm_DependenceNonUniformityNormalized | 8.40 | 23.27 | 0.814 | 0.635 | 0.905 | 27.9 | -18.6 |
| wavelet-LLL_gldm_DependenceVariance | 20.74 | 57.48 | 0.734 | 0.541 | 0.855 | 44.3 | -70.7 |
| wavelet-LLL_gldm_GrayLevelNonUniformity | 17.51 | 48.53 | 0.96 | 0.923 | 0.979 | 55.6 | -41.5 |
| wavelet-LLL_gldm_GrayLevelVariance | 32.91 | 91.22 | 0.509 | 0.157 | 0.732 | 154.1 | -28.3 |
| wavelet-LLL_gldm_HighGrayLevelEmphasis | 29.44 | 81.59 | 0.562 | 0.149 | 0.781 | 132.5 | -30.7 |
| wavelet-LLL_gldm_LargeDependenceEmphasis | 11.93 | 33.08 | 0.754 | 0.572 | 0.866 | 27 | -39.1 |
| wavelet-LLL_gldm_LargeDependenceHighGrayLevelEmphasis | 25.09 | 69.54 | 0.607 | 0.161 | 0.815 | 111.1 | -28 |
| wavelet-LLL_gldm_LargeDependenceLowGrayLevelEmphasis | 41.95 | 116.28 | 0.534 | 0.258 | 0.73 | 83.7 | -148.9 |
| wavelet-LLL_gldm_LowGrayLevelEmphasis | 31.68 | 87.80 | 0.708 | 0.487 | 0.842 | 57 | -118.6 |
| wavelet-LLL_gldm_SmallDependenceEmphasis | 6.10 | 16.89 | 0.799 | 0.639 | 0.893 | 18.8 | -14.9 |
| wavelet-LLL_gldm_SmallDependenceHighGrayLevelEmphasis | 33.24 | 92.12 | 0.549 | 0.151 | 0.769 | 146.4 | -37.9 |
| wavelet-LLL_gldm_SmallDependenceLowGrayLevelEmphasis | 31.03 | 86.01 | 0.693 | 0.469 | 0.832 | 56.4 | -115.6 |
| wavelet-LLL_glrlm_GrayLevelNonUniformity | 17.30 | 47.95 | 0.962 | 0.927 | 0.98 | 55 | -40.9 |
| wavelet-LLL_glrlm_GrayLevelNonUniformityNormalized | 14.22 | 39.42 | 0.885 | 0.739 | 0.946 | 17.3 | -61.6 |
| wavelet-LLL_glrlm_GrayLevelVariance | 32.91 | 91.22 | 0.508 | 0.157 | 0.732 | 154.1 | -28.3 |
| wavelet-LLL_glrlm_HighGrayLevelRunEmphasis | 29.43 | 81.58 | 0.561 | 0.149 | 0.781 | 132.6 | -30.6 |
| wavelet-LLL_glrlm_LongRunEmphasis | 1.02 | 2.81 | 0.769 | 0.595 | 0.875 | 2.5 | -3.2 |
| wavelet-LLL_glrlm_LongRunHighGrayLevelEmphasis | 28.92 | 80.16 | 0.563 | 0.148 | 0.782 | 130.6 | -29.7 |
| wavelet-LLL_glrlm_LongRunLowGrayLevelEmphasis | 32.04 | 88.80 | 0.709 | 0.49 | 0.842 | 57.8 | -119.8 |
| wavelet-LLL_glrlm_LowGrayLevelRunEmphasis | 31.60 | 87.58 | 0.714 | 0.496 | 0.845 | 56.7 | -118.5 |
| wavelet-LLL_glrlm_RunEntropy | 3.03 | 8.40 | 0.867 | 0.145 | 0.961 | 13.4 | -3.4 |
| wavelet-LLL_glrlm_RunLengthNonUniformity | 16.01 | 44.38 | 0.983 | 0.966 | 0.992 | 73.6 | -15.1 |
| wavelet-LLL_glrlm_RunLengthNonUniformityNormalized | 0.62 | 1.73 | 0.8 | 0.643 | 0.893 | 1.9 | -1.5 |
| wavelet-LLL_glrlm_RunPercentage | 0.33 | 0.91 | 0.788 | 0.625 | 0.886 | 1 | -0.8 |
| wavelet-LLL_glrlm_RunVariance | 20.77 | 57.57 | 0.741 | 0.551 | 0.858 | 45.5 | -69.6 |
| wavelet-LLL_glrlm_ShortRunEmphasis | 0.24 | 0.67 | 0.795 | 0.636 | 0.89 | 0.8 | -0.6 |
| wavelet-LLL_glrlm_ShortRunHighGrayLevelEmphasis | 29.58 | 81.99 | 0.561 | 0.149 | 0.78 | 133.1 | -30.8 |
| wavelet-LLL_glrlm_ShortRunLowGrayLevelEmphasis | 31.49 | 87.29 | 0.715 | 0.497 | 0.846 | 56.4 | -118.1 |
| wavelet-LLL_glszm_GrayLevelNonUniformity | 15.52 | 43.03 | 0.984 | 0.968 | 0.992 | 50.4 | -35.7 |
| wavelet-LLL_glszm_GrayLevelNonUniformityNormalized | 13.54 | 37.54 | 0.898 | 0.725 | 0.956 | 14.2 | -60.9 |
| wavelet-LLL_glszm_GrayLevelVariance | 32.71 | 90.67 | 0.499 | 0.152 | 0.724 | 154 | -27.3 |
| wavelet-LLL_glszm_HighGrayLevelZoneEmphasis | 29.29 | 81.19 | 0.553 | 0.149 | 0.774 | 133.5 | -28.9 |
| wavelet-LLL_glszm_LargeAreaEmphasis | 13.28 | 36.81 | 0.679 | 0.457 | 0.822 | 31 | -42.6 |
| wavelet-LLL_glszm_LargeAreaHighGrayLevelEmphasis | 25.25 | 69.99 | 0.585 | 0.163 | 0.798 | 113.2 | -26.8 |
| wavelet-LLL_glszm_LargeAreaLowGrayLevelEmphasis | 37.46 | 103.84 | 0.645 | 0.408 | 0.801 | 70 | -137.7 |
| wavelet-LLL_glszm_LowGrayLevelZoneEmphasis | 31.20 | 86.47 | 0.777 | 0.596 | 0.882 | 55 | -118 |
| wavelet-LLL_glszm_SizeZoneNonUniformity | 20.63 | 57.17 | 0.932 | 0.87 | 0.965 | 89.4 | -24.9 |
| wavelet-LLL_glszm_SizeZoneNonUniformityNormalized | 6.91 | 19.16 | 0.782 | 0.613 | 0.882 | 21.2 | -17.2 |
| wavelet-LLL_glszm_SmallAreaEmphasis | 3.33 | 9.23 | 0.765 | 0.588 | 0.872 | 10 | -8.4 |
| wavelet-LLL_glszm_SmallAreaHighGrayLevelEmphasis | 31.53 | 87.38 | 0.546 | 0.15 | 0.768 | 141.2 | -33.5 |
| wavelet-LLL_glszm_SmallAreaLowGrayLevelEmphasis | 32.04 | 88.82 | 0.768 | 0.583 | 0.876 | 58 | -119.6 |
| wavelet-LLL_glszm_ZoneEntropy | 1.97 | 5.46 | 0.875 | 0.003 | 0.968 | 10.2 | -0.7 |
| wavelet-LLL_glszm_ZonePercentage | 4.50 | 12.47 | 0.799 | 0.639 | 0.893 | 14 | -10.9 |
| wavelet-LLL_glszm_ZoneVariance | 29.78 | 82.55 | 0.616 | 0.367 | 0.783 | 63 | -102.1 |
| wavelet-LLL_ngtdm_Busyness | 27.35 | 75.82 | 0.75 | 0.56 | 0.864 | 45.7 | -105.9 |
| wavelet-LLL_ngtdm_Coarseness | 13.14 | 36.42 | 0.875 | 0.581 | 0.951 | 17.8 | -55 |
| wavelet-LLL_ngtdm_Complexity | 38.56 | 106.89 | 0.498 | 0.191 | 0.713 | 179 | -34.8 |
| wavelet-LLL_ngtdm_Contrast | 19.08 | 52.88 | 0.778 | 0.511 | 0.894 | 71 | -34.8 |
| wavelet-LLL_ngtdm_Strength | 30.99 | 85.89 | 0.49 | 0.141 | 0.718 | 143.9 | -27.9 |
