# Supplementary material for: Prospective inter- and intra-tracer repeatability analysis of radiomics features in [68Ga]Ga-PSMA-11 and [18F]F-PSMA-1007 PET scans in metastatic prostate cancer
Source: Br J Radiol. 2023 Oct 24;96(1152):20221178. doi: 10.1259/bjr.20221178 (PMC10646662; doi:10.1259/bjr.20221178)
Supplement: Supplementary Figure 2. [file bjr.20221178.suppl-02.docx]

| 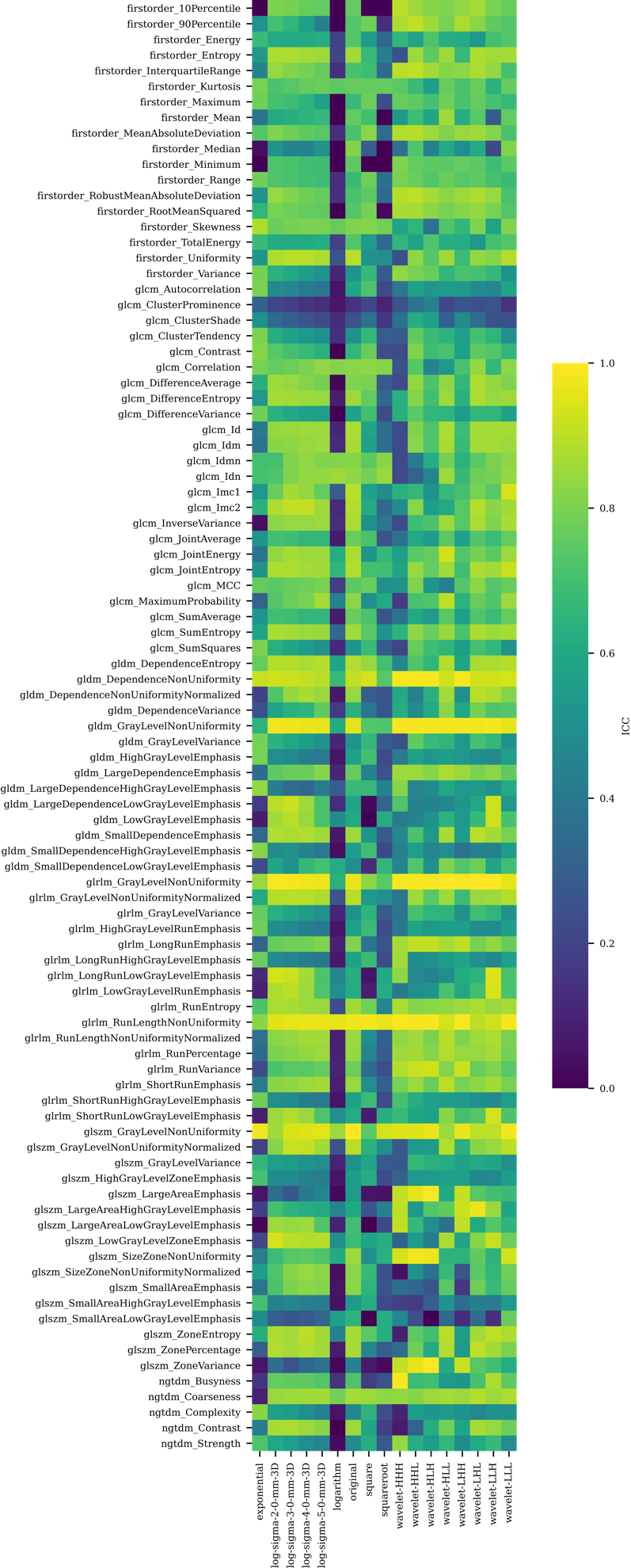 | |
| --- | --- |
|  |  |

**Supplementary Fig. 2** Heatmap showing how the ICC values of features change with the application of different imaging filters for the inter-tracer group. Shape based features are excluded since they are insensitive to the application of filters.
